# Rupturing aromaticity by periphery overcrowding

**DOI:** 10.1038/s41557-023-01149-6

**Published:** 2023-03-06

**Authors:** Promeet K. Saha, Abhijit Mallick, Andrew T. Turley, Aisha N. Bismillah, Andrew Danos, Andrew P. Monkman, Alyssa-Jennifer Avestro, Dmitry S. Yufit, Paul R. McGonigal

**Affiliations:** 1 Department of Chemistry, Durham University, Durham, UK; 2 Department of Physics, Durham University, Durham, UK; 3 Department of Chemistry, University of York, York, UK

**Keywords:** Reaction mechanisms, Structure elucidation

## Abstract

The balance between strain relief and aromatic stabilization dictates the form and function of non-planar π-aromatics. Overcrowded systems are known to undergo geometric deformations, but the energetically favourable π-electron delocalization of their aromatic ring(s) is typically preserved. In this study we incremented the strain energy of an aromatic system beyond its aromatic stabilization energy, causing it to rearrange and its aromaticity to be ruptured. We noted that increasing the steric bulk around the periphery of π-extended tropylium rings leads them to deviate from planarity to form contorted conformations in which aromatic stabilization and strain are close in energy. Under increasing strain, the aromatic π-electron delocalization of the system is broken, leading to the formation of a non-aromatic, bicyclic analogue referred to as ‘Dewar tropylium’. The aromatic and non-aromatic isomers have been found to exist in rapid equilibrium with one another. This investigation demarcates the extent of steric deformation tolerated by an aromatic carbocycle and thus provides direct experimental insights into the fundamental nature of aromaticity.

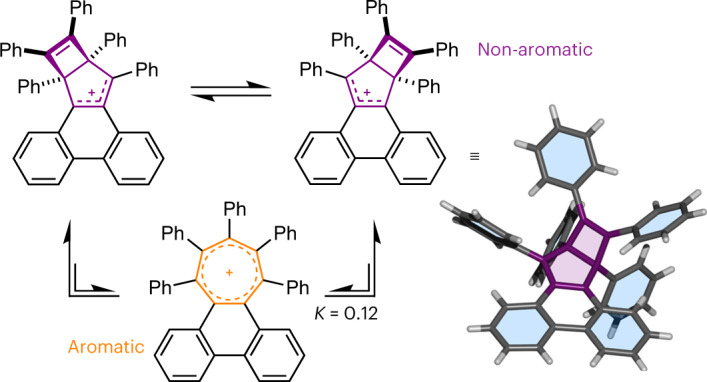

## Abstract (web summary)

Stabilization from aromatic electron delocalization is highly favourable so it is typically preserved in even grossly distorted molecules. Now, peripheral overcrowding of an aromatic tropylium has been shown to cause sufficient geometric strain to rupture aromaticity, forming a non-aromatic bicyclic system that is in rapid equilibrium with its aromatic counterpart.

## Main

**Fig. 1:**
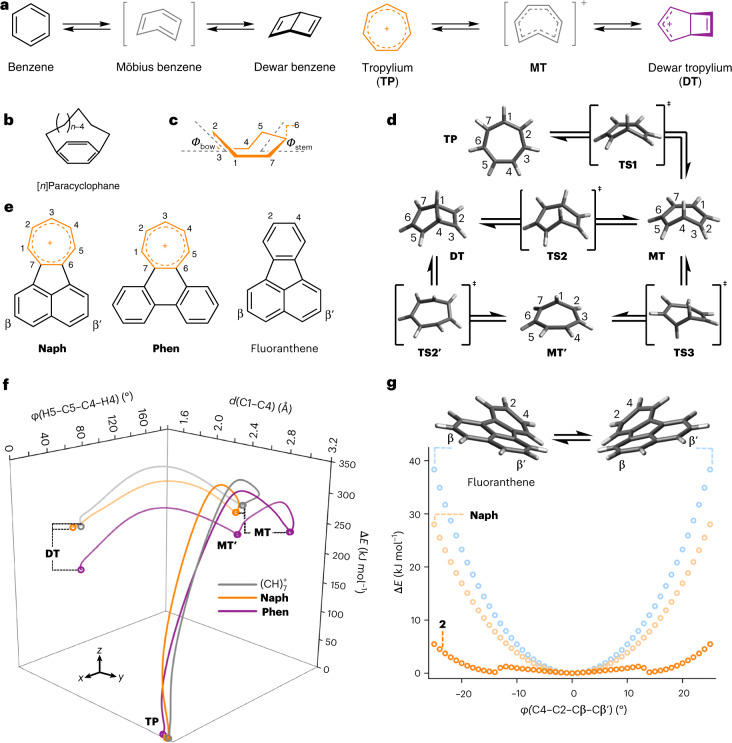
Aromatic ring systems undergo geometric and valence isomerism. **a**, The aromatic, planar isomers of (CH)_6_ and (CH)_7_^+^ are linked to non-aromatic, bicyclic (Dewar) isomers by geometric deformation. **b**, This has been investigated in the distorted benzene rings of [*n*]paracyclophanes. **c**,**d**, Seven-membered ring boat conformations **(c)** are accessed via DFT-optimized energy minima and transition states in the isomerization pathway between tropylium and Dewar tropylium (**d**). **e**, Structural formulae of 1,2-acenaphthylene-annulated tropylium (**Naph**), 9,10-phenanthene-annulated tropylium (**Phen**) and fluoranthene. **f**, The IRCs for the rearrangement of (CH)_7_^+^, **Naph** and **Phen** plotted against C1–C4 internuclear distance, *d*, and H5–C5–C4–H4 dihedral angle, *φ*, show that the tropylium rings must become twisted to access the high-energy **MT** and **DT** isomers, which is facilitated by steric overcrowding. **g**, Plots of the energy of the twisted aromatic systems of fluoranthene, **Naph** and pentaphenyl-substituted **Naph** (**2**) relative to their planar geometries, Δ*E*, obtained by performing a relaxed potential energy scan as the C4–C2–Cβ–Cβ′ torsion angle is varied in 1° increments. See Fig. 2a for the structural formula of **2**. Two-dimensional plots of the data in **f** are shown in Supplementary Fig. 79. All DFT calculations were performed at the B3LYP(GD3BJ)/6-31G(d)/CH_2_Cl_2_ level of theory. Source data

Aromaticity has been one of the fundamental tenets of chemical structure for over a century^1–3^. One school of thought defines it as the ability of a molecule to maintain a diatropic ring current when placed in a magnetic field^4–6^. The cyclic electron delocalization in aromatic systems gives rise to extraordinary stability, equal bond lengths, modified chemical reactivity and unique electronic properties^4,7^. For example, benzene, the archetypal aromatic compound, exhibits exceptionally high stability relative to other (CH)_6_ isomers, such as prismane, benzvalene, Möbius benzene and Dewar benzene (Fig. 1a)^8,9^.

The extraordinary electronic stabilization brought about by aromatic electron delocalization is also evident in the properties of tropylium—an aromatic homologue of benzene that exists as a stable *C*_7_-symmetric carbocation (Fig. 1a). Since Doering and Knox’s seminal work^10^ on its isolation and structural elucidation, its electronic properties have been exploited in organocatalysis^11^, stimuli-responsive dyes^12^ and redox-active polycyclic aromatic hydrocarbons (PAHs)^13–15^. Relative to benzene, its larger ring size, increased conformational flexibility and the smaller angles subtended by its substituents (51.4° versus 60.0° for benzene) allow tropylium to undergo more facile strain-induced deformations^16,17^. Consequently, it has the potential to form highly twisted structures and to serve as a versatile subunit to probe aromaticity in geometrically deformed systems^18^.

In recent years, there has been increasing interest in non-planar aromatic structures, including helicenes^19^, twistacenes^20,21^, Möbius aromatics^22,23^, nanohoops^24–26^, nanobelts^27,28^ and warped nanographenes^29,30^. Relatively large deviations from planarity induced by strain are accommodated in these structures while maintaining substantial π-electron delocalization. However, it is challenging to probe the limits of such deformations experimentally. At what point is the aromatic stabilization energy^26^ (ASE) of a ring system outweighed by its strain?

Certain annulated aromatic rings, such as Siegel’s trisbicyclo[2.1.1]hexabenzene^31^, experience large amounts of bond angle strain, which causes them to undergo substantial geometric distortion^32,33^. Yet, despite the distorted bond lengths present in these π-systems, effective delocalization still occurs and the system remains aromatic^32^. Similarly, bending of a benzene ring into a boat conformation with an out-of-plane angle of 23.2°, enforced by the pentamethylene ‘strap’ in [5]paracyclophanes (Fig. 1b), diminishes the magnitude of its π-ring current by only 17% (refs. ^34,35^). Altering the length of the strap gives some crude control of the strain energy. But a decrease of just one methylene group causes a large jump in strain. For example, [4]paracyclophanes are enormously strained structures that have tentatively been assigned^36–38^ to undergo thermally irreversible isomerization to the corresponding Dewar benzenes. However, the inherent instability and reactivity of these short-lived systems have limited their analysis to spectroscopic studies at low temperatures. So far, it has not been possible to fine-tune the level of strain present in an aromatic system, titrating it to the point at which the ASE is overcome. The consequences of precisely offsetting the ASE through strain have not been investigated.

Here we report the use of periphery overcrowding^20,39^, that is, the introduction of sterically demanding substituents around the exterior of a molecule, to tune the geometric deformations experienced by π-extended tropylium ring systems beyond the point at which strain exceeds ASE. On one side of this energetic balance point, we identify structures that exhibit twisted geometries while retaining aromaticity. In the most extreme case, single-crystal X-ray diffraction (XRD) analysis shows (1) an end-to-end twist angle along the π-extended ring system of 45.2° and (2) that the tropylium is in a distorted boat conformation (*Φ*_bow_ = 13.0° and *Φ*_stern_ = 29.0°; Fig. 1c), while computational modelling of its electronic properties indicates that these large geometric distortions reduce its aromatic character by only ~13%. On the other side of the energetic balance point, the π-extended ring systems sacrifice aromaticity in favour of relieving strain, that is, by collapse of their tropylium rings into bicyclic Dewar tropylium structures^40^. Dynamic nuclear magnetic resonance (NMR) spectroscopic analysis shows that an overcrowded Dewar tropylium undergoes reversible exchange between degenerate structures, passing through a twisted tropylium intermediate. This exchange is indicative of a dynamic intramolecular aromatic-to-non-aromatic equilibrium process that arises by counterbalancing aromaticity against substantial ring strain. Understanding these competing energetics in sterically strained systems is integral to designing and exploiting non-planar PAHs that exist at the limits of aromaticity^41^.

## Results and discussion

**Fig. 2:**
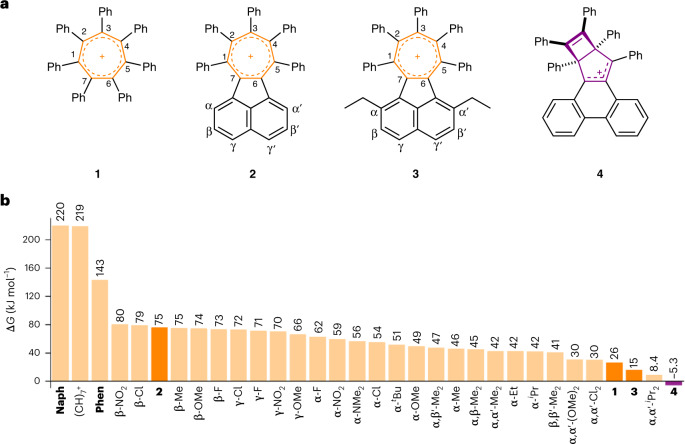
Periphery overcrowding tunes the balance of steric strain and aromatic stabilization. **a**,**b**, Cations **1**–**4** synthesized in this work (**a**) were selected on the basis of the calculated Gibbs energy differences, Δ*G*, between tropylium and the Dewar tropylium forms of (CH)_7_^+^, **Naph**, **Phen**, **1**–**4** and a series of derivatives of **2** (B3LYP(GD3BJ)/6-31G(d)/CH_2_Cl_2_) (**b**). The most overcrowded derivative, **4**, is predicted to lose aromaticity in favour of relieving strain by rearranging to its Dewar tropylium form. In **b**, the indicated positions of the different substituents of the derivatives of **2** correspond to the labelling shown in the structure of **2** in **a**. Positive Δ*G* values (orange bars) indicate an energetically favourable tropylium isomer, while negative Δ*G* values (purple bar) denote a bias towards Dewar tropylium. For asymmetric substitution patterns that break the *C*_2_ symmetry, the lower energy of the two possible Dewar tropylium isomers has been used to estimate Δ*G*.

Previous computational studies^42^ in conjunction with kinetic measurements^43^ have shown that Dewar benzene-to-benzene isomerization proceeds (Fig. 1a) through a conrotatory electrocyclic ring opening. Initially, a highly strained *cis*,*cis*,*trans*-cyclohexatriene (Möbius benzene) intermediate is formed, before a π-bond rotation produces benzene. Relative to benzene, Dewar benzene is destabilized by 326 kJ mol^−1^, while the largest activation energy barrier along the isomerization pathway lies at 443 kJ mol^−1^ (ref. ^44^). With this benchmark in mind, we used density functional theory (DFT) to establish the energetic characteristics of deforming and isomerizing (CH)_7_^+^ through its tropylium (**TP**), Möbius-like tropylium (**MT**) and Dewar tropylium (**DT**) isomers (Fig. 1), as well as isomerizing our subsequent synthetic targets **1**–**4** (Fig. 2). Note that **MT** is named by analogy to Möbius benzene, but lacks the *C*_2_ symmetry of a genuine Möbius topology.

### Aromatic-to-non-aromatic (CH)_7_^+^ isomerization

A screen of functionals and basis sets (Supplementary Table 15) indicated that the B3LYP functional^45^ with the D3 version of Grimme’s dispersion correction and Becke–Johnson damping^46^, the 6-31G(d) basis set^47^ and a CH_2_Cl_2_ polarizable continuum solvent model (using the integral equation formalism variant)^48^ is suitable for modelling the energetics of these systems. Using this level of theory, we identified (Fig. 1d) a series of possible transition states and Möbius-like tropylium intermediates that could link tropylium and Dewar tropylium. We also modelled (Fig. 1e) the same processes for two π-extended tropylium derivatives containing *ortho*-fused 1,2-acenaphthylene (**Naph**) or 9,10-phenanthrene (**Phen**) ring systems—the parent compounds of our subsequent targets **2**–**4** (Fig. 2a).

We visualized the isomerization pathways (Fig. 1f) through intrinsic reaction coordinate (IRC) calculations^49^ using the optimized transition-state geometries. Transition state **TS1** connects the high-energy **MT** intermediate to the aromatic **TP** isomer. During this transformation, the most important geometric change occurs in the torsion angle *φ*(H5–C5–C4–H4) as it varies (Fig. 1f) from 0° (**TP**) to approximately 180° (**MT**). Starting from transition state **TS2** (the conrotatory ring-opening transition state), the IRC paths for (CH)_7_^+^ and **Naph** provide the aforementioned Möbius-like intermediates in one direction and the Dewar tropylium isomers in the other, completing the tropylium-to-Dewar tropylium isomerization pathway. This structural evolution is best visualized by following (Fig. 1f) the change in the internuclear distance *d*(C1–C4), which is shortest (~1.6 Å) when C1 and C4 share a single bond in a Dewar tropylium structure and longest (~3.1 Å) when they are separated across an aromatic tropylium ring. Our calculations suggest that a slightly modified isomerization pathway is preferred for **Phen**. The **MT** state evolves first to another Möbius-like intermediate (**MT′**), in which C1 rather than C4 is in the out-of-plane position, before shortening of *d*(C1–C4) leads to the **DT** structure. **TS1** corresponds to the highest energy point along the IRCs of (CH)_7_^+^ and **Phen**, presenting rate-limiting activation energy barriers, Δ*G*^‡^, of 302 and 297 kJ mol^−1^, respectively (Supplementary Tables 16–21). **TS2** is the highest energy point along the IRC of **Naph**, with Δ*G*^‡^ = 316 kJ mol^−1^. Further details, including the full structures of all energy minima and transition states, are included in Supplementary Figs. 71–73.

Overall, the aromatic-to-non-aromatic isomerization of tropylium generally mirrors that of benzene (Fig. 1). Similar distortions of the aromatic ring geometry occur, albeit with the increased flexibility and reduced ASE^26^ of the seven-membered ring contributing to a lower overall activation energy barrier, Δ*G*^‡^ (302 kJ mol^−1^ for tropylium versus 443 kJ mol^−1^ for benzene). We quantified the magnitude of this reduced ASE by calculating the energetics of hyperhomodesmotic^50^ reactions (Supplementary Fig. 76) according to von Ragué Schleyer and co-workers’ isomerization stabilization energy method^51^. Using the same level of theory outlined above, we found (CH)_7_^+^, **Naph** and **Phen** to have ASEs of approximately −50 kJ mol^−1^ (Table 1), which are approximately half the ASE of benzene (−98.5 kJ mol^−1^). As the B3LYP functional is known to overestimate aromatic stabilization in larger aromatic circuits^52^, we also estimated ASEs using the M06-2X functional and found them to be comparable to our B3LYP predictions (Supplementary Tables 24 and 25).

**Table 1:** Torsion angles and tropylium aromaticity

	(CH)_7_^+^	Naph	Phen	1	2	3	4-TP
*φ*(C4–C2–Cβ–Cβ′) calc.^a^ (°)	–	0	31.6	–	14.3, 0.4^e^	45.4	61.7
*φ*(C4–C2–Cβ–Cβ′) exp.^b^ (°)	–	0	–	–	18.4, 0.4^e^	45.2	–
*Φ*_bow_ calc.^a^ (°)	0	0	5.3	16.3	2.8^f^	15.0	16.3
*Φ*_bow_ exp.^b^ (°)	0	–	–	13.6	6.7^f^	13.0	–
*Φ*_stern_ calc.^a^ (°)	0	0	14.8	18.4	7.3^f^	28.8	31.8
*Φ*_stern_ exp.^b^ (°)	0	–	–	12.2	13.7^f^	29.0	-
ASE (kJ mol^–1^)	−50.3	−55.1	−50.7	–	–	–	–
NICS_*zz*_(1)^a,c^	−29.11	−18.38	−19.22	–18.91	−15.59^f^	−14.01	−16.90
NICS_*zz*_(−1)^a,c^	−29.11	−18.38	−19.22	−18.90	−15.57^f^	−14.07	−16.90
EDDB^*k* d^	4.94	3.08, 6.00	3.01, 7.05	4.26	3.09, 5.97^f^	2.69, 5.89	2.84, 6.66

^a^Calculated (calc.) using DFT-optimized geometries (B3LYP(GD3BJ)/6-31G(d)/CH_2_Cl_2_). ^b^Experimentally measured (exp.) by single-crystal XRD analysis. ^c^NICS_*zz*_(±) values were calculated 1 Å above and below the averaged plane of the tropylium rings. ^d^Where two values are given, they correspond to the number of delocalized electrons in the tropylium ring circuit and the number delocalized over the entire annulated framework, respectively. ^e^Energy minima with two different torsion angles are predicted to be similar in energy by DFT (5.6 kJ mol^−1^), matching closely the conformers of **2** observed in the crystal lattice. ^f^Values are given for the twisted conformer of **2**.

### Thermodynamic tuning by periphery overcrowding

We postulated that the introduction of sterically bulky groups around the periphery of (CH)_7_^+^ and its π-extended derivatives would decrease the aromatic-to-non-aromatic Δ*G* by favouring twisted geometries that more closely resemble the non-planar Möbius-like and Dewar structures. To test this hypothesis, we performed a series of geometry optimizations for fluoranthene, **Naph** and **2** (Fig. 1g). The torsion angle *φ*(C4–C2–Cβ–Cβ′) was scanned in increments of 1° to determine the energetic cost of twisting deformations. This end-to-end twisting in **Naph** carries a lower energy penalty than its six-membered ring counterpart, fluoranthene, as would be expected given the greater degrees of freedom of its seven-membered ring. The additional peripheral overcrowding caused by the phenyl groups of **2** destabilizes the fully coplanar tropylium structure, dramatically flattening (Fig. 1g) the energy well for twisting deformation. Indeed, through subsequent unrestrained geometry optimizations, we identified two local energy minima for structures with *φ*(C4–C2–Cβ–Cβ′) of 14.3° and 0.4° (Table 1). These two conformations strike different balances between the optimal π-electron delocalization of the planar structure and the reduced steric strain of the non-planar structure. In keeping with this phenomenon, our calculations also show that this peripheral overcrowding makes the Möbius-like isomers much more energetically accessible. The Δ*G* between tropylium **2** and its Möbius-like form, **2-MT**, was calculated to be only 96 kJ mol^−1^ (compared with a gap of 259 kJ mol^−1^ for the model compound **Naph**, which lacks Ph groups). Similarly for compound **4**, the two Möbius-like isomers **4-MT** and **4-MT′** are only 67 and 28 kJ mol^−1^ higher in energy than the tropylium form **4-TP** (again showing a substantial decrease relative to the analogous structures **Phen-MT** and **Phen-MT′** with Δ*G* = 232 and 209 kJ mol^−1^, respectively).

We further investigated the impacts of steric and electronic factors on the aromatic-to-non-aromatic Gibbs energy gap by modelling an extended series of compounds. Starting from the previously reported heptaphenyltropylium cation **1** (ref. ^17^), which exhibits a 26 kJ mol^–1^ preference for the aromatic tropylium isomer, we sought to increase the steric bulk in the plane of the tropylium core. Cation **2** provides an ideal scaffold for this purpose^53^. Despite its overcrowding, this species has an increased preference for the tropylium isomer (Δ*G* = 75 kJ mol^–1^) on account of the extensive π-electron delocalization between the tropylium and the annulated naphthyl rings^54^. By contrast, the phenyl groups of **1** and **2** lie almost orthogonal to the central rings to minimize steric strain, so they have minimal π-overlap with the tropylium rings.

Our calculations suggest (Fig. 2) that while electron-donating or -withdrawing substituents at the β- and γ-positions of the acenaphthyl ring system tune the energetic balance of isomers by a few kilojoules per mole, increasing the steric bulk at the α- and α′-positions (which abut the phenyl rings) tunes the relative isomer energies over a larger range. For example, α,α′-dimethyl substitution reduces the energy gap to 42 kJ mol^−1^. The gap is reduced to 15 kJ mol^−1^ by the α,α′-diethyl substitution of compound **3** and further still to 8.4 kJ mol^−1^ by α,α′-diisopropyl substitution. The low thermodynamic bias in favour of the aromatic isomer for these overcrowded systems approaches the critical point where the ASE and strain energy are evenly balanced. Consequently, the tropylium ring geometries would be expected to be among the most distorted possible. Indeed, increasing the size of the appended moiety to a phenanthrene-annulated system pushes the thermodynamic preference towards the Dewar tropylium isomer. The tropylium form of the parent **Phen** is already destabilized^55^ (Fig. 2b) relative to (CH)_7_^+^ and **Naph** on account of a more sterically congested tropylium-to-PAH bay region. The added strain caused by the five proximal phenyl rings of **4** is sufficient to tune the energetic balance in favour of the Dewar tropylium by 5.3 kJ mol^–1^.

### Synthesis of twisted and Dewar tropyliums

**Fig. 3:**
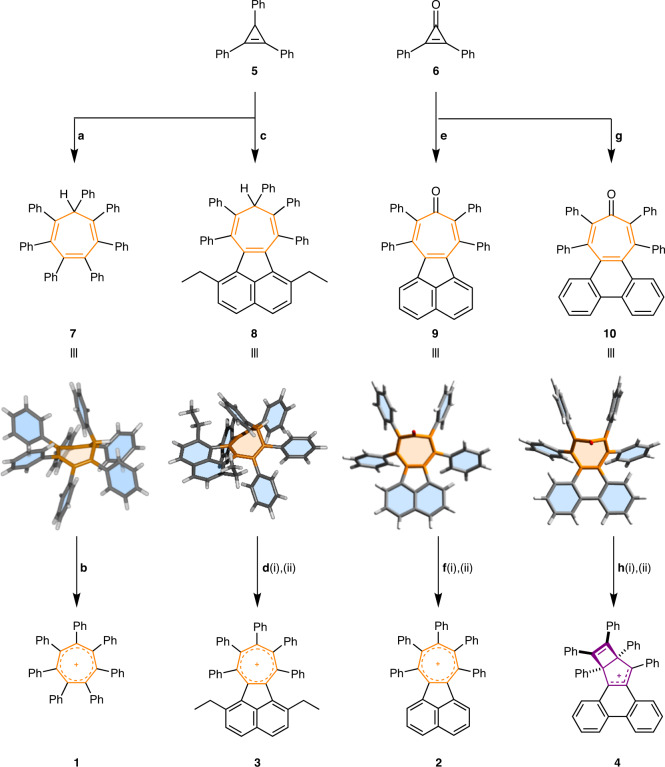
Synthesis of sterically overcrowded tropyliums **1**–**4**. **a**–**h**, Reagents and conditions: **a**, **5**, tetracyclone, *p*-xylene, 140 °C, 36 h, 82%; **b**, ICl (1.0 M in CH_2_Cl_2_), rt, 24 h, 60%; **c**, **5**, diethylacecyclone, *p*-xylene, 190 °C, 84 h, 22%; **d**(i) *m*CPBA, CHCl_3_, saturated aqueous NaHCO_3_, reflux, 20 h, 12%; (ii) BBr_3_, CH_2_Cl_2_, rt, 10 min; **e**, **6**, acecyclone, PhMe−CHCl_3_ (5:3), 130 °C, 24 h, 34%; **f**(i) PhMgBr, THF, 0 °C → rt, 3 h, 68%; (ii) Et_3_O·SbCl_6_, CDCl_3_, rt, 6 h, quantitative; **g**, **6**, phencyclone, PhMe, 130 °C, 24 h, 40%; **h**(i) PhMgBr, THF, 0 °C → rt, 3 h, 60%; (ii) Et_3_O·SbCl_6_, CDCl_3_, rt, 6 h, quantitative. rt, room temperature; *m*CPBA = *m*-chloroperbenzoic acid. Solid-state structures determined by XRD analysis are shown for the four key intermediates **7**–**10**.

Cations **1**–**4** were selected as synthetic targets that span a wide range of calculated Δ*G* values: **1** and **2** favour an aromatic tropylium, **4** is biased towards a Dewar tropylium isomer and **3** is close to the border between the two. Their syntheses are outlined in Fig. 3.

Firstly, dienophiles **5** and **6** were both prepared from diphenylacetylene (Supplementary Fig. 1). As a precursor to **1**, *sym*-heptaphenylcycloheptatriene, **7**, was formed (Fig. 3) by the Diels**–**Alder cycloaddition of **5** with tetraphenylcyclopentadienone (tetracyclone) and subsequent cheletropic elimination of CO (ref. ^56^). Oxidation of **7** using an excess of ICl then yielded **1** as its iodine dichloride salt (**1**·ICl_2_). The analogous phenanthrene- and acenaphthene-annulated cycloheptatrienes, as well as an α,α′-diethyl derivative, **8**, were also accessed through the same approach by treating **5** with an appropriate cyclopentadienone reagent (phencyclone, acecyclone and diethylacecyclone, respectively). However, attempts to oxidize these compounds with ICl or Br_2_ led to unwanted halogenation of the electron-rich annulated ring systems in preference to oxidation of the cycloheptatrienes. Hydride abstraction reactions were also unsuccessful.

Instead, the target cations **2** and **4** were prepared successfully (Fig. 3) by first treating acecyclone and phencyclone with **6** to afford tropones **9** and **10**, respectively. Subsequent nucleophilic attack by PhMgBr at the carbonyl introduced a final phenyl ring as well as a tertiary alcohol group, which was readily eliminated by treatment with triethyloxonium hexachloroantimonate to give **2**·SbCl_6_ and **4**·SbCl_6_, respectively.

We attempted a similar strategy to prepare **3**. However, cycloaddition between **6** and diethylacecyclone did not yield the desired tropone. We observed that the high temperature (190 °C) required for the reaction of this sterically crowded diene caused the rapid decomposition of **6**. Instead, we employed a stepwise oxidation protocol to convert cycloheptatriene **8** into tropylium **3**. Oxidation of **8** using *m*CPBA in refluxing CHCl_3_ led to a mixture of products, from which we isolated an epoxide intermediate (**S1**). By treating the epoxide with a stoichiometric amount of BBr_3_ under the inert conditions of a glove box, we were able to form **3** in trace amounts and isolate single crystals of its tetrabromoborate salt **3**·BBr_4_ by slow evaporation of the reaction mixture. The apparently high reactivity of the contorted cation **3** and associated low yield prevented us from obtaining solution-state spectroscopic data. However, XRD analysis of the crystals confirmed its identity and allowed us to measure its geometric parameters (vide infra).

### Geometric distortions of tropylium rings

**Fig. 4:**
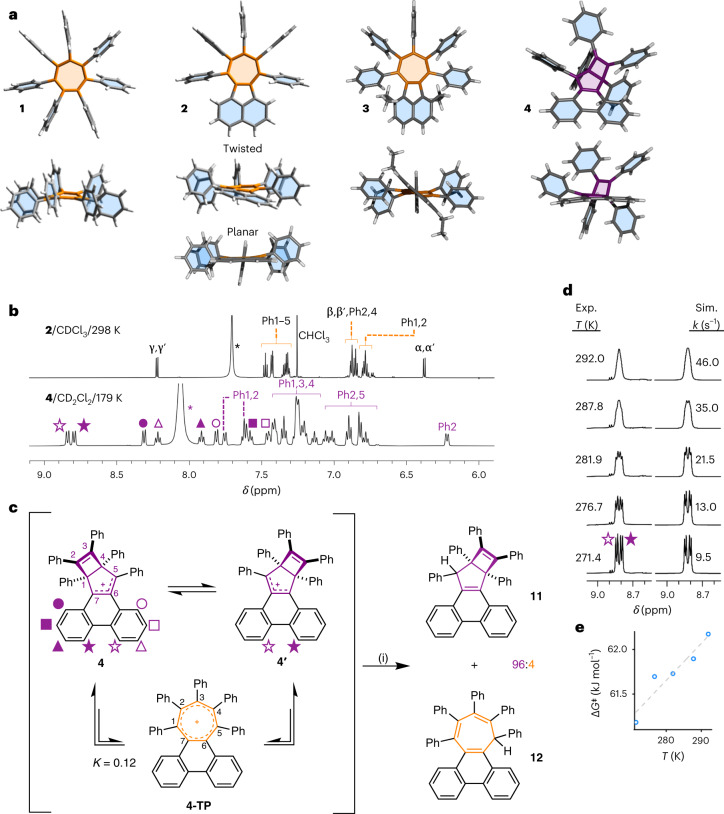
Highly twisted tropyliums are found to undergo a non-aromatic-to-aromatic equilibrium. **a**–**e**, Single-crystal XRD analysis and NMR spectroscopy show the increasingly twisted geometries of **1**–**3** and the preferred Dewar tropylium structure of **4**, which is in equilibrium with **4**-**TP**. **a**, Plan and side views of single-crystal X-ray structures of cations **1**–**4**. Counterions and solvent molecules have been omitted for clarity. Both of the conformers present in the unit cell of **2** are shown in side view. **b**, Partial ^1^H NMR spectra of **2**·SbCl_6_ (top, 700 MHz) and **4**·SbCl_6_ (bottom, 500 MHz). Full spectra are shown in Supplementary Figs. 26 and 32, respectively. The peaks of **2** are labelled according to the numbering scheme shown in Fig. 2. Asterisks denote signals corresponding to R_2_OH^+^ (R = H or Et), formed as a byproduct from the reaction of the Et_3_O^+^ reagent. Filled and hollow shape symbols represent pairs of inequivalent resonances that arise by breaking the tropylium *C*_2_ symmetry upon forming the Dewar tropylium. **c**, The dynamic exchange between two degenerate Dewar tropylium isomers of **4** and its tropylium form was intercepted by reduction to give **11** and **12** in a ratio of 96:4. Reagents and conditions: (i) NaBH_4_, THF, rt, 30 min. **d**, This dynamic rearrangement causes broadening of NMR resonance lineshapes as the temperature is raised from 271.4 to 292.0 K. Full NMR spectra are shown in Supplementary Fig. 41. The rate data were obtained by comparing the experimental (Exp.) and simulated (Sim.) lineshapes. **e**, Fitting the rate data to the Eyring equation gives an Eyring plot of Δ*G*^‡^ versus *T* for the exchange. The linear fit (dashed grey line) indicates Δ*H*^‡^ = 50.0 kJ mol^−1^ and Δ*S*^‡^ = −41.5 J K^−1^ mol^−1^. Source data

We determined the solid-state structures of **1**–**4** (Fig. 4a) by single-crystal XRD analysis. While the geometries of the cations are influenced by crystal packing effects and the enforced proximity to their anions, we found that the crystal structures of **1**–**4** are in good agreement with our calculated solution-state cation geometries^57^. The geometry of **1** in crystals of **1**·ICl_2_ is similar to that found previously for its trifluoroacetate salt^17^. Its tropylium ring adopts a shallow boat conformation to minimize steric interactions between adjacent phenyl rings, giving interplanar angles (Table 1) at the bow and stern (Fig. 1c) of *Φ*_bow_ = 13.6° and *Φ*_stern_ = 12.2°.

Orange crystals of **2**·SbCl_6_ were grown by slow evaporation of a CDCl_3_ solution. Pleasingly, the facile twisting of **2** predicted by DFT (Fig. 1g) is evident in the solid-state structure determined by XRD analysis (Fig. 4a). We found two non-degenerate conformers present in the unit cell—a near-planar structure with *φ*(C4–C2–Cβ–Cβ′) = 0.4° and a twisted conformer with *φ*(C4–C2–Cβ–Cβ′) = 18.4°, which match the two minima predicted by DFT calculations.

Taking the twisting deformation to its extreme, the solid-state structure of **3**·BBr_4_ (Fig. 4a) shows that its cation adopts an extremely distorted geometry on account of the severe peripheral overcrowding, while remaining as the aromatic tropylium isomer (rather than a Dewar tropylium). It has a large end-to-end twist with *φ*(C4–C2–Cβ–Cβ′) = 45.2°, which matches closely the predicted twist of *φ*(C4–C2–Cβ–Cβ′) = 45.4° (Table 1). Examination of the crystal packing showed that the twisted acenaphthyltropylium units all have the same helical screw sense as one another, that is, the crystal has formed as a conglomerate^58^. The seven-membered ring of **3** is distorted from planarity to a much greater extent than that of **1** or **2** (Table 1). It has a boat conformation characterized by interplanar angles of *Φ*_bow_ = 13.0° and *Φ*_stern_ = 29.0°. An informative comparison can also be made with the neutral, benzenoid homologue of this tropylium, 1,6-diethyl-7,8,9,10-tetraphenylfluoranthene (**S5**), which was isolated as a side-product during the synthesis of **3**. The benzene-centred molecule is significantly less distorted than **3** (Supplementary Fig. 51). Its torsion angle of *φ*(C4–C2–Cβ–Cβ′) = 22.7° and interplanar angles of *Φ*_bow_ = 4.4° and *Φ*_stern_ = 13.2° are smaller than those measured for **3**, reflecting the greater inherent flexibility and increased propensity for peripheral overcrowding of tropylium compared with benzene.

### Non-aromatic-to-aromatic dynamics of a Dewar tropylium

The ^1^H NMR spectrum of **2**·SbCl_6_ (Fig. 4b) shows that, as expected for the tropylium structure, the cation retains the *C*_2_ symmetry of its cycloheptatriene precursor. The ^1^H NMR spectrum of **4**·SbCl_6_, however, exhibits a larger number of resonances, indicating that **4** lacks *C*_2_ symmetry. Its lower symmetry is consistent with the formation of a Dewar tropylium structure in solution. As an illustrative example, the NMR signal arising from the two *peri* positions of **2** (γ and γ′), which appears as a sharp doublet (that is, a single resonance), can be contrasted with the two distinct doublets arising from the phenanthrene bay region of **4** (open and filled star symbols, Fig. 4b) that appear in the ^1^H NMR spectrum recorded at 179 K. These solution-state NMR data are complemented by the XRD analysis of dark-purple single crystals grown by the slow evaporation of **4**·SbCl_6_ in CH_2_Cl_2_ (Fig. 4a), which confirmed the presence of the Dewar tropylium ring system in the solid state. To our knowledge, this is the first non-aromatic valence isomer of a tropylium derivative that has been isolated.

Close to room temperature, several of the ^1^H NMR peaks of **4** broaden and merge (Supplementary Fig. 41), which we attribute to dynamic exchange between its two degenerate valence isomers (Fig. 4c). Taking the phenanthrene bay protons as an example, these nuclei trade magnetic environments as **4** rearranges to **4′**. A ^1^H–^1^H exchange NMR spectroscopy experiment (Supplementary Fig. 42) confirmed that these proton environments are in exchange. We performed NMR lineshape analysis (Fig. 4d) to derive exchange rates for this process at a series of temperatures close to the signal coalescence point. An Eyring plot based on these data (Fig. 4e) gives an enthalpy of activation, Δ*H*^‡^, of 50.0 kJ mol^−1^ and an entropy of activation, Δ*S*^‡^, of −41.5 J K^−1^ mol^−1^, corresponding to a Δ*G*^‡^ of 62.4 kJ mol^−1^ at 298 K.

By analogy to the isomerization pathways identified (Fig. 1f) for (CH)_7_^+^, **Naph** and **Phen**, the exchange between **4** and **4′** presumably involves cleavage of the C1–C4 bond to form an aromatic intermediate, **4-TP** (Fig. 4c). Indeed, DFT modelling of **4-TP** indicates that, despite it having a large end-to-end twist angle of *φ*(C4–C2–Cβ–Cβ′) = 61.7°, it is an energetically viable intermediate. It lies only 5.3 kJ mol^−1^ higher in energy than **4**. Consequently, it should exist as a minor, but detectable, species at equilibrium. With the thermal energy available at 298 K, the Boltzmann distribution of isomers (Supplementary Table 26) for this Gibbs energy gap gives an ~10% probability of any given cation occupying the **4-TP** state, that is, the dynamic rearrangement of **4** to **4-TP** has an equilibrium constant *K* = 0.12. To test this prediction, we performed a hydride ‘trapping’ experiment by treating a solution of **4** with NaBH_4_ (Fig. 4c). ^1^H NMR spectroscopic analysis of the crude mixture obtained after aqueous work-up showed that two bicyclo[3.2.0]heptadiene diastereoisomers, *anti*-**11** and *syn*-**11**, were produced along with a cycloheptatriene product^59^, **12**, in a 38:58:4 ratio (Fig. 4c and Supplementary Figs. 38 and 39). The structure of *anti*-**11** was confirmed by XRD analysis (Supplementary Fig. 68). The observation of **12** is consistent with its tropylium precursor, **4-TP**, being present at equilibrium in solution.

Overall, therefore, the peripheral overcrowding dictates the energy gap between the non-aromatic isomer **4** and its aromatic form **4-TP**, and tunes the kinetics of their interconversion. It does so by selectively destabilizing the aromatic tropylium isomer relative to its Dewar and Möbius-like tropylium isomers, as well as the transition-state structure(s) that bridge(s) them. The **4** and **4-TP** isomers have been brought to within a few kilojoules per mole of one another, establishing a non-aromatic-to-aromatic equilibrium that is weighted in a ratio of ~90:10 towards the non-aromatic form. The experimentally measured Δ*G*^‡^ of 62.4 kJ mol^−1^ for this rearrangement is significantly lower than the Δ*G*^‡^ of the parent compound (**Phen**) lacking the bulky phenyl groups, which is predicted (Fig. 1f) to be 297 kJ mol^−1^. Consequently, the equilibrium is established rapidly with the thermal energy available at room temperature, which is reflected experimentally in the broadening of the NMR peaks and the formation of a cycloheptatriene product following reaction with NaBH_4_.

### Aromaticity probes

**Fig. 5:**
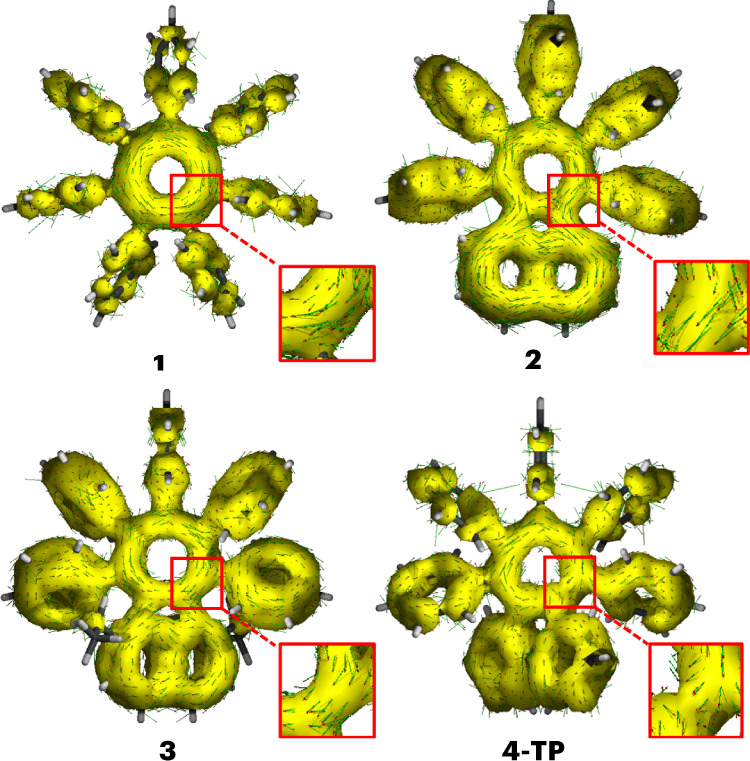
ACID plots. The DFT-optimized geometries (B3LYP(GD3BJ)/6-31G(d)/CH_2_Cl_2_) of **1–3** and **4-TP** overlaid with the ACID isosurfaces (isovalue = 0.01) for their respective π-orbitals (see Supplementary Section 6.8 for orbital numbers). The red-headed arrows demonstrate clockwise current density vectors for the seven-membered rings (see the magnified areas), which are indicative of aromatic electron delocalization.

Our experimental results led us to the following question: are the large geometric distortions of **3** and the ruptured aromaticity observed for **4** best attributed to them having increased strain relative to the other derivatives (that is, **1** and **2**), or to them having reduced aromatic character? To investigate the aromaticity of cations **1**–**3** and **4**-**TP**, we characterized their tropylium units using both magnetic and electronic criteria. Anisotropy of the induced current density (ACID) plots (Fig. 5) show the presence of a clockwise ring current in all four species, which is indicative of aromaticity^60^. The aromatic ring current in **2** extends to the appended naphthalene moiety, corroborating its stabilizing effect through extensive charge delocalization. The extent of this π-electron delocalization is reduced in **3** and **4-TP**, as the annulated acenaphthyl and phenanthrenyl groups are twisted further from the plane of the tropylium.

We calculated the *zz* components of the nucleus-independent chemical shifts (NICS_*zz*_(±1); refs. ^61,62^), to gain insights into the local aromaticity of the tropylium rings (Table 1). Negative and positive NICS values are characteristic of aromaticity and antiaromaticity, respectively, whereas a NICS value of around zero suggests non-aromatic character^63^. The NICS_*zz*_(±1) values of −14.01 to −18.91 calculated for **1**–**3** and **4**-**TP** (Table 1) are consistent with the aromatic character predicted by their ACID plots.

To complement this magnetic assessment, we also determined electron density of delocalized bond (EDDB^*k*^) values (Table 1) for the cyclic delocalization of π-electrons through pathways that can be represented by Kekulean resonance forms. EDDB^*k*^ is a quantitative electronic index that estimates the number of electrons delocalized in an aromatic circuit^64^. We assessed electron delocalization involving the tropylium ring alone (local aromaticity) and, for the π-extended systems **2**, **3**, **4-TP**, **Naph** and **Phen**, we also assessed electron delocalization involving the entire polycyclic framework of the molecule (global aromaticity, shown in Supplementary Figs. 77 and 78)^65^. A direct comparison is most easily drawn between **Naph**, **2** and **3**, which share the same cyclic framework. The EDDB^*k*^ values of **2** are almost identical to those of **Naph**, suggesting perphenylation has little impact on the effectiveness of electron delocalization along both pathways. Even with the severe deviation from planarity imposed by the additional ethyl groups of **3**, the EDDB^*k*^ value for the local aromaticity of the tropylium drops by just 13% compared with **Naph** (from 3.08 to 2.69), suggesting a modest decrease in aromatic character. Similarly, the tropylium moiety in **1** suffers a 13% drop in aromatic character compared with (CH)_7_^+^, while **4-TP** experiences a very small 6% reduction compared with **Phen**.

Together, these analyses demonstrate that cyclic π-electron delocalization persists despite the steric deformation of these tropyliums. The aromaticity of the tropylium isomer **4-TP** is comparable to that of **1**–**3**. Therefore, its rearrangement to the Dewar tropylium isomer and associated rupture of aromaticity occur primarily as a consequence of its greatly increased strain (relative to **Phen**) exceeding the ASE, rather than the geometric changes causing any substantial decrease in the aromatic character of the tropylium.

## Conclusions

Peripheral overcrowding causes considerable structural deformations in a series of π-extended aromatic cations. These deformations manifest as boat-type conformations of their tropylium cores and helical twists along the length of their π-extended polycyclic frameworks, giving experimentally measured torsion angles of up to 45.2° in **3**. The Gibbs energy gap between the aromatic and non-aromatic isomers of **3** is only 15 kJ mol^–1^, placing it among the most overcrowded and twisted tropylium structures that can feasibly be synthesized. By imposing even greater peripheral overcrowding, the balance between strain and aromatic stabilization tips in favour of a bicyclic Dewar tropylium structure, as observed in **4**. Spectroscopic and DFT evidence show that **4** exists as a dynamic mixture in solution. At room temperature, it passes back and forth rapidly between non-aromatic (**4** and **4′**) and aromatic (**4-TP**) isomers in an ~90:10 ratio, establishing a non-aromatic-to-aromatic equilibrium. Our calculations suggest that even the most deformed aromatic tropylium isomers **3** and **4-TP** retain the electronic and magnetic hallmarks of aromaticity. Yet, when bulky groups are introduced in a manner that raises steric overcrowding for the aromatic isomer preferentially, the ASE can be outweighed by the strain energy, and the equilibrium shifts towards a non-aromatic species.

## Online content

Any methods, additional references, Nature Portfolio reporting summaries, source data, extended data, supplementary information, acknowledgements, peer review information; details of author contributions and competing interests; and statements of data and code availability are available at 10.1038/s41557-023-01149-6.

## Supplementary information

Supplementary Information Supplementary Figs. 1–78, Tables 1–141, Discussion and Experimental procedures.

Supplementary Data 1 Crystallographic data for compound **1**·ICl_2_; CCDC reference 2141786.

Supplementary Data 2 Structure factors for compound **1**·ICl_2_; CCDC reference 2141786.

Supplementary Data 3 Crystallographic data for compound **2**·SbCl_6_ at 120 K; CCDC reference 2141787.

Supplementary Data 4 Structure factors for compound **2**·SbCl_6_ at 120 K; CCDC reference 2141787.

Supplementary Data 5 Crystallographic data for compound **2**·SbCl_6_ at 270 K; CCDC reference 2173731.

Supplementary Data 6 Structure factors for compound **2**·SbCl_6_ at 270 K; CCDC reference 2173731.

Supplementary Data 7 Crystallographic data for compound **3**·BBr_4_; CCDC reference 2141788.

Supplementary Data 8 Structure factors for compound **3**·BBr_4_; CCDC reference 2141788.

Supplementary Data 9 Crystallographic data for compound **4**·SbCl_6_; CCDC reference 2141789.

Supplementary Data 10 Structure factors for compound **4**·SbCl_6_; CCDC reference 2141789.

Supplementary Data 11 Crystallographic data for compound **8**; CCDC reference 2141790.

Supplementary Data 12 Structure factors for compound **8**; CCDC reference 2141790.

Supplementary Data 13 Crystallographic data for compound **9**; CCDC reference 2141791.

Supplementary Data 14 Structure factors for compound **9**; CCDC reference 2141791.

Supplementary Data 15 Crystallographic data for compound **10**; CCDC reference 2141792.

Supplementary Data 16 Structure factors for compound **10**; CCDC reference 2141792.

Supplementary Data 17 Crystallographic data for compound *anti*-**11**; CCDC reference 2182241.

Supplementary Data 18 Structure factors for compound *anti*-**11**; CCDC reference 2182241.

Supplementary Data 19 Crystallographic data for compound **S2**; CCDC reference 2141793.

Supplementary Data 20 Structure factors for compound **S2**; CCDC reference 2141793.

Supplementary Data 21 Crystallographic data for compound **S3**; CCDC reference 2141794.

Supplementary Data 22 Structure factors for compound **S3**; CCDC reference 2141794.

Supplementary Data 23 Crystallographic data for compound **S4**; CCDC reference 2141795.

Supplementary Data 24 Structure factors for compound **S4**; CCDC reference 2141795.

Supplementary Data 25 Crystallographic data for compound **S5**; CCDC reference 2141796.

Supplementary Data 26 Structure factors for compound **S5**; CCDC reference 2141796.

## Data Availability

The crystallographic data for the structures reported in this Article have been deposited at the Cambridge Crystallographic Data Centre, under deposition numbers CCDC 2141786 (**1**·ICl_2_), 2141787 (**2**·SbCl_6_ (120 K)), 2173731 (**2**·SbCl_6_ (270 K)), 2141788 (**3**·BBr_4_), 2141789 (**4**·SbCl_6_), 2141790 (**8**), 2141791 (**9**), 2141792 (**10**), 2182241 (*anti*-**11**), 2141793 (**S2**), 2141794 (**S3**), 2141795 (**S4**) and 2141796 (**S5**). Copies of the data can be obtained free of charge via https://www.ccdc.cam.ac.uk/structures/. All other data supporting the findings of this study are available within the paper and its Supplementary Information. Source data are provided with this paper.

## Source data

Source Data Fig. 1f Bond lengths, bond angles and energies of IRC calculations.
Source Data Fig. 1g Torsion angles and energies of relaxed potential energy scans.
Source Data Fig. 4e NMR lineshape analysis data for Fig. 4e.
