# Supplementary material for: Rupturing aromaticity by periphery overcrowding
Source: Nat Chem. 2023 Mar 6;15(4):516–25. doi: 10.1038/s41557-023-01149-6 (PMC10070187; doi:10.1038/s41557-023-01149-6)
Supplement: Supplementary file 1 — Supplementary Figs. 1–78, Tables 1–141, Discussion and Experimental procedures. [file 41557_2023_1149_MOESM1_ESM.pdf]

nature chemistry

Article

https://doi.org/10.1038/s41557-023-01149-6

Rupturing aromaticity by periphery overcrowding

In the format provided by the authors and unedited

Rupturing Aromaticity by Periphery Overcrowding

Promeet K. Saha,† Abhijit Mallick,† Andrew T. Turley,† Aisha N. Bismillah,† Andrew Danos,‡ Andrew P. Monkman,‡ Alyssa-Jennifer Avestro,# Dmitry S. Yufit,† Paul R. McGonigal†,*

†Department of Chemistry and ‡Department of Physics, Durham University, Lower Mountjoy, Stockton Road, Durham, DH1 3LE, United Kingdom

#Department of Chemistry, University of York, Heslington, York, YO10 5DD, United Kingdom

Table of Contents

1. General Methods	S2
2. Synthetic Procedures	S4
3. ^{1}H and ^{13}C NMR Spectroscopic Characterization of Synthesized Compounds	S20
3.1. Variable-Temperature (VT) NMR Spectroscopy	S58
3.2. Exchange NMR Spectroscopy	S60
4. X-Ray Crystallographic Analysis	S61
4.1. **1**·ICl_2	S61
4.2. **8**	S64
4.3. **3**·BBr_4	S66
4.4. Diels-Alder Intermediate (**S4**)	S68
4.5. **S5**	S70
4.6. **9**	S72
4.7. **S2**	S74
4.8. **2**·$SbCl_6$ (120 K)	S76
4.9. **2**·$SbCl_6$ (270 K)	S78
4.9. **10**	S81
4.10. **S3**	S83
4.11. **4**·$SbCl_6$	S85
4.12. *anti*-**11**	S87
5. UV-Vis Absorption Spectroscopy	S89
6. *In Silico* Modelling	S91
6.1. General Methods	S91
6.2. Basis Set and Functional Screen	S92
6.3. Intrinsic Reaction Coordinate (IRC) Calculations	S92
6.4. Potential Energy Surface (PES) Scan	S113
6.5. Relative Stabilities of Tropylium Isomers	S114
6.6. Aromatic Stabilization Energy (ASE) Calculations	S116
6.7. Nucleus-Independent Chemical Shift (NICS) Values	S117
6.8. Anisotropy of Induced Current (ACID) Plots	S118
6.9. Electron Density of Delocalized Bonds (EDDB) Values and Plots	S118
6.10. Boltzmann Distribution of **4**/**4-TP**	S121
6.11. Optimized Structures	S121
6.12. Additional Data	S321
7. References	S322

Supporting Information

General Methods

Materials: All reagents were purchased from commercial suppliers (Sigma-Aldrich, Acros Organics, or Alfa Aesar) and used without further purification.

Instrumentation and Analytical Techniques: Microwave reactions were performed using an automated Biotage reactor, Robot-Sixty, model: Initiator EXP EU, 355301, 11327-36W. Analytical thin-layer chromatography (TLC) was performed on neutral aluminum sheet silica gel plates and visualized under UV irradiation (254 nm). Nuclear magnetic resonance (NMR) spectra were recorded using a Bruker Advance (III)-400 (^{1}H 400.130 MHz and ^{13}C 100.613 MHz), Varian Inova-500 (^{1}H 500.130 MHz and ^{13}C 125.758 MHz), Varian VNMRS-600 (^{1}H 600.130 MHz and ^{13}C 150.903 MHz) or a Varian VNMRS-700 (^{1}H 700.130 MHz and ^{13}C 176.048 MHz) spectrometer, at a constant temperature of 298 K unless otherwise stated. For variable-temperature measurements, operating temperatures were calibrated using an internal calibration solution of MeOH and glycerol. Chemical shifts (δ) are reported in parts per million (ppm) relative to the signals corresponding to residual non-deuterated solvents [$CDCl_3$: δ = 7.26 or 77.16. CD_2Cl_2: δ = 5.32 or 54.00]. Coupling constants (J) are reported in Hertz (Hz). ^{13}C NMR Experiments were proton decoupled. Assignment of ^{1}H and ^{13}C NMR signals were accomplished by two-dimensional NMR spectroscopy (COSY, NOESY, HSQC, HMBC). NMR spectra were processed using MestReNova version 14.2. Data are reported as follows: chemical shift; multiplicity; coupling constants; integral and assignment. Low-resolution ASAP-MS were performed using a Waters Xevo QTOF equipped with an Atmospheric Solids Analysis Probe (ASAP). High-resolution electrospray (HRESI) and ASAP (HRASAP) mass spectra were measured using a Waters LCT Premier XE high resolution, accurate mass UPLC ES MS (also with ASAP ion source). Melting points were recorded using a Gallenkanp (Sanyo) apparatus and are uncorrected. X The X-ray single crystal data have been collected at the temperature 120.0(2)K on the Bruker D8Venture (Photon100 CMOS or Photon

III MM C7 CPAD detectors, IμS-microsource, focusing mirrors, 3-circle goniometer) and Agilent XCalibur (compound **13**; Sapphire-3 CCD detector, fine-focus sealed tube, graphite monochromator, 4-circle κ-goniometer) diffractometers equipped with a Cryostream (Oxford Cryosystems) open-flow nitrogen cryostats. Data were collected using λMoKα or λCuKα (compounds **12** and **14**) radiation (λ =0.71073 and 1.54178Å respectively). All structures were solved by direct method and refined by full-matrix least squares on F^2 for all data using Olex2[1] and SHELXTL[2] software. . All non-disordered non-hydrogen atoms were refined in anisotropic approximation, the hydrogen atoms were placed in the calculated positions and refined in riding mode in all structures except the **14** one, where they were freely refined isotropically. Crystal data and parameters of refinement are listed in Tables S1–S11. Crystallographic data for the structures have been deposited with the Cambridge Crystallographic Data Centre as supplementary publication CCDC 2141786–2141796, 2173731, and 2182241.

2. Synthetic Procedures

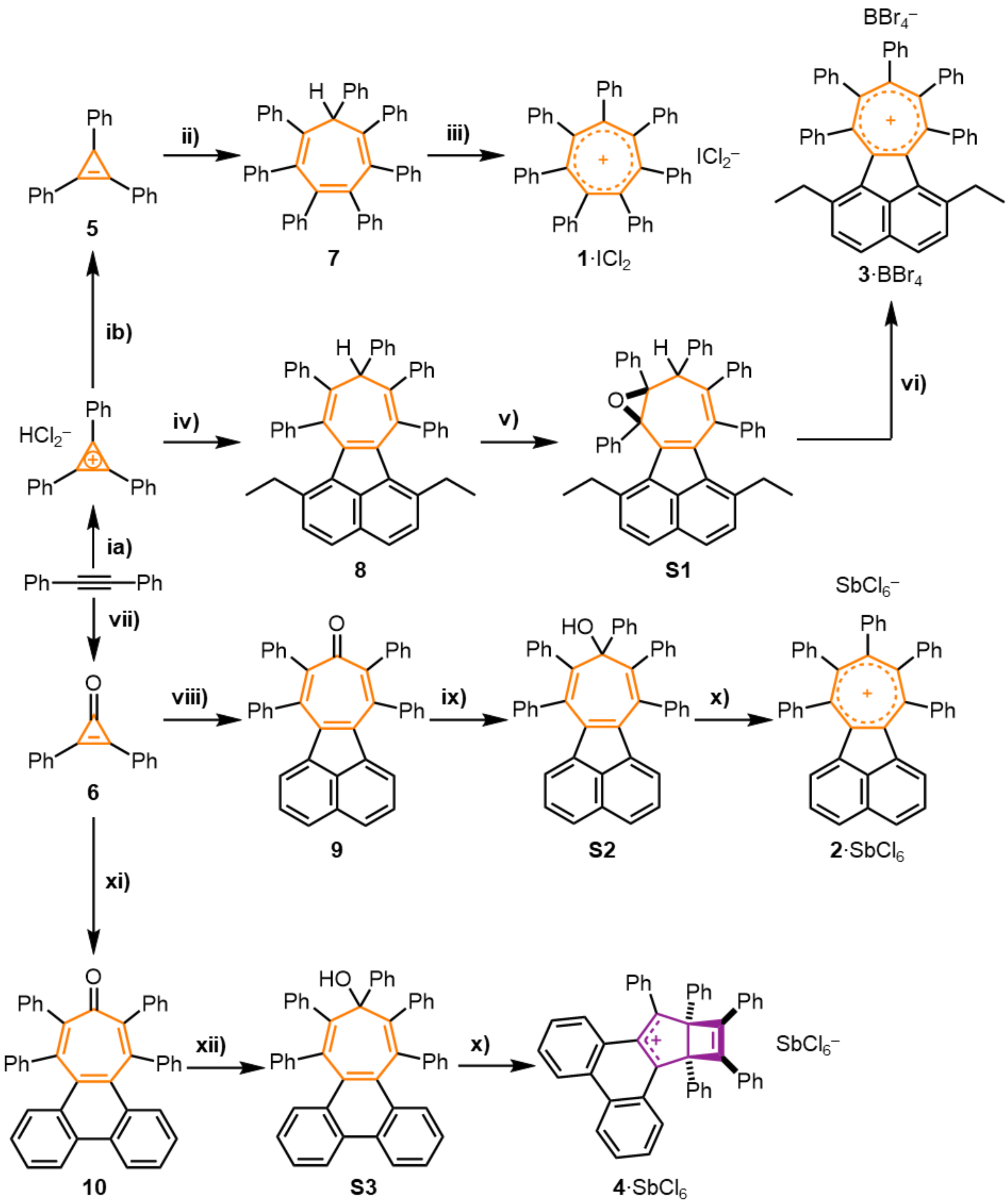

Figure S1. Synthesis of tropylium cations **1** – **4**. Reagents and conditions: (ia) 1. KO*t*Bu, *α*,*α*-dichlorotoluene, C_6H_6, reflux, 3 h; 2. $HCl_{(g)}$, Et_2O–CH_2Cl_2 (2:1), rt. (ib) $NaBH_4$, EtOH, 0 °C → rt, 16 h, 97%. (ii) tetracyclone, *p*-xylene, 140 °C, 24 h, 82%. (iii) ICl, CH_2Cl_2, rt, 24 h, 60%. (iv) diethylacecyclone, *p*-xylene, 190 °C, 84 h, 22%. (v) *m*CPBA, $CHCl_3$, sat. $NaHCO_{3\,(aq)}$, reflux, 20 h, 12%. (vi) BBr_3, $CDCl_3$, rt, 10 min, trace amounts. (vii) 1. diphenylacetylene, KO*t*Bu, hexane, $CHBr_3$, –20 °C → rt, 18 h; 2. H_2O, rt, 30 min, 65%. (viii) acecyclone, PhMe–$CHCl_3$ (5:3), 130 °C, 24 h, 34%. (ix) PhMgBr, THF, 0 °C → rt, 3 h, 68%. (x) $Et_3O{\cdot}SbCl_6^-$,$CDCl_3$, rt, 10 min, quant. (xi) phencyclone, PhMe, 130 °C, 24 h, 40%. (xii) PhMgBr, THF, 0 °C / 3.5 h / 60%. *m*CPBA = *m*-chloroperbenzoic acid.

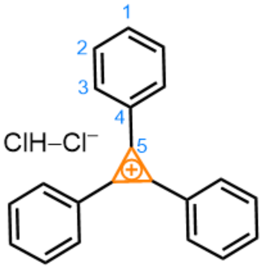

$Ph_3C_3{\cdot}HCl_2$

Triphenylcyclopropenylium hydrogen dichloride ($Ph_3C_3{\cdot}HCl_2$): Anhydrous benzene (200 mL) was added to diphenylacetylene (18.5 g, 104 mmol) and KO^{t}Bu (35.0 g, 312 mmol) in an oven-dried two-necked 500 mL round-bottomed flask fitted with a septum under an inert atmosphere. α,α-Dichlorotoluene (25.0 g, 155 mmol) was added uniformly over a period of 30 min using a syringe pump, and the reaction mixture was then heated to reflux for 3 h. After cooling, H_2O (200 mL) was added to dissolve the inorganic salts. The layers were separated and the aqueous layer was extracted with Et_2O (2 × 200 mL). The combined organic extracts were dried over $MgSO_4$, and the solvent was removed under reduced pressure to yield orange residue. This was dissolved in a mixture of 2:1 Et_2O–CH_2Cl_2 (200 mL) and sparged with gaseous HCl, leading to the formation of a colorless precipitate. Sparging was continued until no more precipitation was observed. The precipitate was collected by filtration, and the solid was washed with Et_2O, then dried under vacuum to yield the title compound as cream-colored powder (18.6 g, 61.4 mmol, 43%). **M.P.** 186 – 188 °C. **^{1}H NMR** (600 MHz, CD_3CN) δ 8.83 – 8.39 (m, 6H, H_3), 8.20 – 8.00 (m, 3H, H_1), 7.97 – 7.86 (m, 6H, H_2). **^{13}C NMR** (151 MHz, CD_3CN) δ 154.1 (C_5), 139.1 (C_1), 136.6 (C_2), 131.4 (C_3), 121.6 (C_4). **HRESI-MS** *m/z* = 267.1169 $[M]^+$ (calculated for $C_{21}H_{15}^+$ = 267.1168).

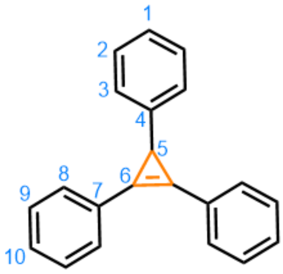

5

***sym*-Triphenylcyclopropene (5):** $NaBH_4$ (9.30 g, 246 mmol) was added to a solution of **$Ph_3C_3{\cdot}HCl_2$** (18.6 g, 61.4 mmol) in EtOH (460 mL) at 0°C. The mixture was allowed to stir overnight at rt. The reaction was quenched with H_2O (500 mL) then extracted with Et_2O (3 × 200 mL). The combined organic extracts were washed with H_2O (500 mL), then brine (500 mL), and dried over $MgSO_4$. The solvent was removed under reduced pressure to give **5** as a colorless solid (14.2 g, 53.1 mmol, 97%). **M.P.** 113 –115 °C

(lit.[3] 114 – 116 °C). **^{1}H NMR** (400 MHz, $CDCl_3$) δ 7.71 – 7.66 (m, 4H, H_8), 7.47 – 7.40 (m, 4H, H_9), 7.38 – 7.32 (m, 2H, H_{10}), 7.24 (br s, 2H, H_2), 7.23 – 7.22 (m, 2H, H_3), 7.17 – 7.10 (m, 1H, H_1), 3.27 (s, 1H, H_5). **^{13}C NMR** (101 MHz, $CDCl_3$) δ 144.6 (C_4), 130.0 (C_8), 128.9 (C_9), 128.8 (C_{10}), 128.7 (C_7), 128.3 (C_3), 126.0 (C_2), 125.6 (C_1), 112.7 (C_6), 24.5 (C_5). **HR-ESI MS** *m/z* = 267.1172 $[M-H]^+$ (calculated for $C_{21}H_{15}^+$ = 267.1168).

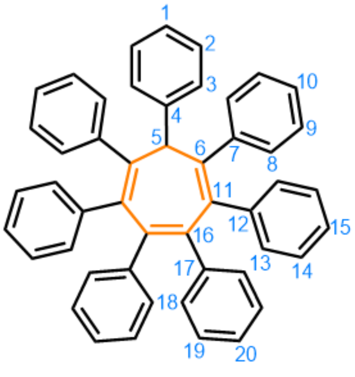

7

sym-**Heptaphenylcycloheptatriene** (**7**): Anhydrous *p*-xylene (13.5 mL) was added to **5** (1.20 g, 4.47 mmol) and tetraphenylcyclopentadienone (1.79 g, 4.47 mmol) in a sealed, oven-dried microwave vial under an inert atmosphere. The mixture was deoxygenated (3 × freeze–pump–thaw cycles under N_2) then stirred for 36 h at 140 °C in a microwave reactor. Upon cooling to rt, a crystalline solid formed, which was isolated by filtration, and washed with Et_2O (3 × 10 mL). The solid was dried under vacuum to yield **7** as a pale pink solid (2.30 g, 3.68 mmol, 82%) in high purity (no observable impurities by ^{1}H NMR spectroscopy). Samples for analytical measurements were further purified by recrystallization. A saturated solution of **7** in $CHCl_3$–EtOH (1:1) was allowed to evaporate slowly, yielding colorless crystals of analytically pure **7**. **M.P.** 291 – 292 °C (lit.[4] 285 – 288 °C). **^{1}H NMR** (700 MHz, $(CD_3)_2CO$) δ 8.17 – 7.99 (m, 2H, H_3), 7.62 – 7.48 (m, 2H, H_2), 7.44 – 7.34 (m, 1H, H_1), 7.26 – 7.18 (m, 4H, H_8), 7.15 (br s, 4H, H_{13}), 7.05 – 7.02 (m, 4H, H_{14}), 7.02 – 6.98 (m, 4H, H_9), 6.98 – 6.96 (m, 2H, H_{15}), 6.96 – 6.93 (m, 2H, H_{10}), 6.63 – 6.59 (m, 2H, H_{20}), 6.60 – 6.55 (m, 4H, H_{19}), 6.36 – 6.32 (m, 4H, H_{18}), 5.35 (s, 1H, H_5). **^{13}C NMR** (176 MHz, $(CD_3)_2CO$) δ 144.8 (C_{16}), 144.4 (C_7), 144.1 (C_4), 141.9 (C_{12}), 141.6 (C_{17}), 140.2 (C_6), 138.0 (C_{11}), 132.7 (C_{13}), 132.3 (C_{18}), 130.7 (C_8), 129.4 (C_2), 128.5 (C_9), 128.1 (C_{14}), 127.9 (C_3), 127.7 (C_1), 127.3

(C_{10}), 126.93 (C_{15}), 126.87 (C_{19}), 126.0 (C_{20}), 59.0 (C_5). **HR-ESI MS** *m/z* = 625.2892 $[M+H]^+$ (calculated for $C_{47}H_{37}^+$ = 625.2890).

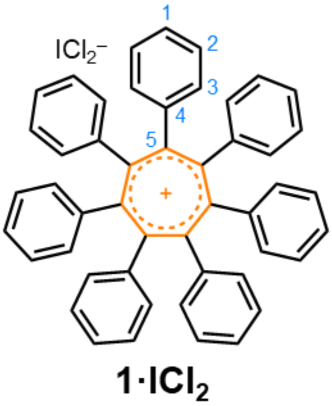

Heptaphenyltropylium iodine dichloride (1·ICl2): ICl (1.0 M in CH_2Cl_2, 1 mL, 1.0 mmol) was added to a solution of **7** (100 mg, 0.16 mmol) in anhydrous CH_2Cl_2 (10 mL), and the mixture was stirred at rt for 24 h. The solvent was removed under reduced pressure to give a crude dark purple solid, which was dissolved in anhydrous MeCN (15 mL) and triturated with anhydrous Et_2O (25 mL). The resulting precipitate was collected by vacuum filtration to yield the title compound as an orange solid (97 mg, 0.096 mmol, 60%) **M.P.** 280 – 282 °C. **^{1}H NMR** (400 MHz, CD_3CN) δ 6.92 – 6.88 (m, 14 H, H_3), 6.87 – 6.78 (m, 21H, H_{1+2}). **^{13}C NMR** (101 MHz, CD_3CN) δ 167.3 (C_5), 141.6 (C_4), 130.5 (C_3), 127.8 (C_2), 127.6 (C_1). **HR-ASAP MS** *m/z*= 623.2712 $[M-ICl_2]^+$ (calculated for $C_{49}H_{35}^+$ = 623.2739).

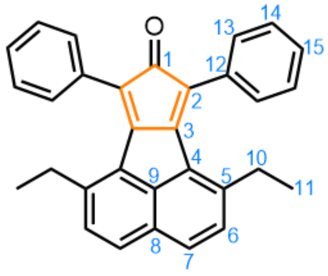

Diethylacecyclone

Diethylacecyclone: A solution of NaOH (0.31 g, 7.7 mmol) in EtOH (20 mL) was added dropwise to a refluxing suspension of 1,3-diphenyl-2-propanone (0.90 g, 4.5 mmol) and 3,8-diethylacenaphthenequinone (0.93 g, 4.1 mmol) in EtOH (20 mL). The reaction mixture was refluxed for 30 min, then immediately placed onto an ice-bath. Once cooled, the resulting precipitate was collected *via* vacuum filtration and washed with cold EtOH (10 mL) to give the title compound as a black solid (0.50 g, 1.2 mmol, 30%). **M.P.** 140 – 142 °C. **^{1}H NMR** (700 MHz, $CDCl_3$) δ 7.74 (d, J = 8.3 Hz, 2H, H_7), 7.45 – 7.37 (m, 10H, $H_{13,14,15}$),

7.35 (d, J = 8.3 Hz, 2H, H_6), 2.40 (q, J = 7.5 Hz, 4H, H_{10}), 0.85 (t, J = 7.6 Hz, 6H, H_{11}). **^{13}C NMR** (176 MHz, $CDCl_3$) δ 202.9 (C_1), 156.2 (C_3), 146.6 (C_9), 140.5 (C_5), 133.1 (C_{12}), 130.4 (C_4), 129.4 (C_{13}), 128.7 (C_{14}), 128.4 (C_{15}), 128.0 (C_7), 127.6 (C_8), 127.6 (C_6), 121.8 (C_2), 28.2 (C_{10}), 14.9 (C_{11}). **HR-ESI MS** *m/z* = 413.1894 $[M+H]^+$ (calculated for $C_{31}H_{25}O^+$ = 413.1900).

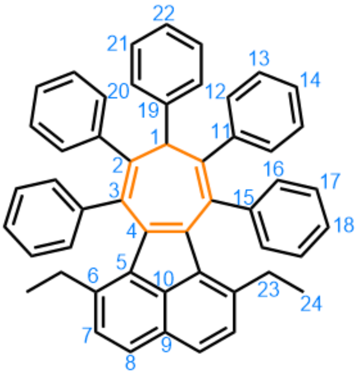

8

8: Anhydrous *p*-xylene (10 mL) was added to **5** (0.13 g, 0.49 mmol) and diethylacecyclone (0.20 g, 0.49 mmol) in a sealed, oven-dried microwave vial under an inert atmosphere. The mixture was deoxygenated (3 × freeze–pump–thaw cycles under N_2), then stirred for 84 h at 190 °C. The solvent was removed under reduced pressure, leaving a brown residue, which was purified by column chromatography (Teledyne Isco CombiFlash Rf+ system, 40 g SiO_2, hexanes−CH_2Cl_2, 0 – 20% gradient elution), to yield **8** as a yellow solid (0.071 g, 0.11 mmol, 22%). **M.P.** 198 – 200 °C. **^{1}H NMR** (400 MHz, $CDCl_3$) δ 7.52 (m, 4H, $H_{8,20}$), 7.16 – 7.10 (m, 4H, $H_{18,21}$), 7.08 – 7.03 (m, 10H, $H_{12,13,14}$), 7.01 – 6.84 (m, 8H, $H_{16,17}$), 6.83 – 6.76 (m, 2H, H_7), 6.66 (t, J = 7.4 Hz, 1H, H_{22}), 5.48 (s, 1H, H_1), 2.27 (m, 4H, H_{23}), 0.68 (t, J = 7.5 Hz, 6H, H_{24}). **^{13}C NMR** (101 MHz, $CDCl_3$) δ 143.4 (C_{19}), 142.9 (C_{15}), 141.5 (C_{11}), 141.1 (C_6), 140.6 (C_9), 137.8 (C_3), 134.7 (C_{10}), 132.4 (C_{16}), 131.9 (C_{20}), 130.6 (C_2), 129.0 (C_5), 128.5 (C_{21}), 127.8 (C_{12}), 127.6 (C_{17}), 127.2 (C_{22}), 126.8 (C_{13}), 126.5 (C_{14}), 126.2 (C_{18}), 125.6 (C_7), 125.5 (C_4), 125.4 (C_8), 60.6 (C_1), 27.1 (C_{23}), 14.2 (C_{24}). **HR-ESI MS** *m/z* = 653.3223 $[M+H]^+$ (calculated for $C_{51}H_{41}^+$ = 653.3208).

In addition to **8**, *trace amounts of the Diels-Alder intermediate* (**S4**) (*identified by single-crystal X-ray crystallography, see section* **4.4**) *as well as 1,6-diethyl-7,8,9,10-tetraphenylfluoranthene* (**S5**) (0.020 g, 0.071 mmol, 7%) (*section* **4.5**) *were isolated after column chromatography.*

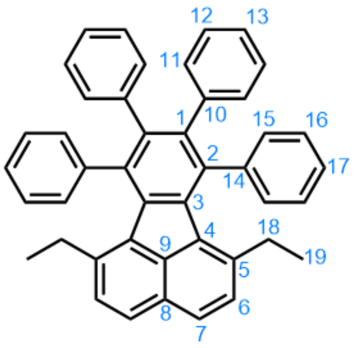

S5

S5: **^{1}H NMR** (600 MHz, $CDCl_3$) δ 7.71 (d, *J* = 8.3 Hz, 2H, H_7), 7.26 (d, *J* = 8.3 Hz, 2H, H_6), 7.14 – 7.07 (m, 10 H, $H_{15,16,117}$), 6.90 – 6.85 (m, 6H, $H_{12,13}$), 6.6.73 – 6.69 (m, 4H, H_{11}), 1.75 (q, 4H, *J* = 7.5 Hz, H_{18}), 0.81 (t, 6H, *J* = 7.5 Hz, H_{19}). **^{13}C NMR** (151 MHz, $CDCl_3$) δ 142.4 (C_{14}), 141.0 (C_{10}), 140.8 (C_5), 140.2 (C_1), 139.6 (C_3), 136.4 (C_2), 134.4 (C_9), 133.1 (C_4), 131.8 (C_{15}), 131.7 (C_{11}), 129.9 (C_6), 127.7 ($C_{16/17}$), 126.8 (C_7), 126.7 (C_8), 126.6 (C_{12}), 126.5 ($C_{17/16}$), 125.2 (C_{12}), 27.6 (C_{18}), 16.0 (C_{19}).

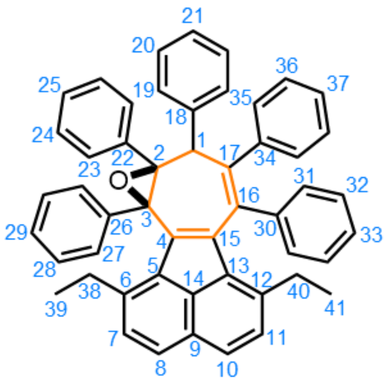

S1

S1: *m*CPBA (≤77% by wt., 38 mg, 150 μmol) was added to a solution of **8** (59 mg, 77 μmol) in $CHCl_3$ (2.5 mL). One drop of a saturated aqueous solution of $NaHCO_3$ was added, and the biphasic mixture was heated to reflux for 20 h. Upon cooling to rt, a saturated aqueous solution of $NaHCO_3$ (10 mL) was added, and the layers were separated. The aqueous layer was extracted with CH_2Cl_2 (2 × 10 mL). The combined organic extracts were dried over $MgSO_4$, and the solvent was removed under reduced pressure, leaving an orange residue, which was purified by column chromatography (Teledyne Isco CombiFlash Rf+ system, 12 g SiO_2, hexanes−CH_2Cl_2, 0 – 20% gradient elution), to yield **11** as a yellow solid (5.9 mg, 8.3 μmol, 12%). **^{1}H NMR** (600 MHz, $CDCl_3$) δ 7.60 (d, *J* = 8.3 Hz, 2H, H_{19}), 7.57 (d, *J* = 8.4 Hz, 1H, H_{10}), 7.53 (d, *J* = 8.2 Hz, 1H, H_8), 7.25 – 7.21 (m, 3H, $H_{25/29}$+H_{31}), 7.21 – 7.17 (m, 2H, H_{36}), 7.16 (d, *J* = 8.4 Hz, 1H, H_{11}), 7.14 – 7.10 (m, 4H, $H_{24/28}$+H_{32}), 7.09 (d, *J* = 8.3 Hz, 1H, H_7), 7.08 – 6.99 (m, 9H, $H_{23,24/28,27,29/25,35}$), 6.97 – 6.92 (m, 4H, $H_{20,33,37}$), 6.81 (t, *J* = 7.3 Hz 1H, H_{21}), 4.90 (s, 1H, H_1), 2.78 – 2.69 (m, 1H, $H_{38'}$), 2.66 – 2.57 (m, 2H, $H_{38,40}$), 2.57 – 2.50 (m, 1H, $H_{40'}$), 0.61 (t, *J* = 7.4

Hz, 3H, H_{41}), 0.50 (t, J = 7.4 Hz, 3H, H_{39}). **^{13}C NMR** (151 MHz, $CDCl_3$) δ 144.0 (C_{16}), 142.5 (C_6), 142.2 (C_{12}), 141.30 ($C_{15/26}$), 141.29 ($C_{26/15}$), 140.7 ($C_{22/34}$), 140.6 ($C_{34/22}$), 139.1 (C_{17}), 137.5 (C_{30}), 137.0 (C_{18}), 136.8 (C_4), 134.2 (C_{13}), 133.5 (C_5), 131.7 (C_{31}), 129.6 (C_{14}), 129.3 (C_7), 129.0 (C_{19}), 128.7 (C_{27}), 128.4 (C_{11}), 127.9 (C_{36}), 127.58 ($C_{23/24/25/28/29}$), 127.56 (C_{10}), 127.52 (C_{37}), 127.5 (C_8), 127.2 ($C_{20,23/24/25/28/29}$), 127.1 ($C_{35,23/24/25/28/29}$), 126.92 (C_{33}), 126.90 ($C_{32,23/24/25/28/29}$), 126.6 ($C_{23/24/25/28/29}$), 125.5 (C_{21}), 125.2 (C_9), 77.0 (C_2), 64.2 (C_3), 59.0 (C_1), 27.3 (C_{40}), 26.6 (C_{38}), 15.0 (C_{39}), 14.1 (C_{41}). **HR-ASAP MS** m/z = 669.3154 $[M+H]^+$ (calculated for $C_{51}H_{41}O^+$ = 669.3157).

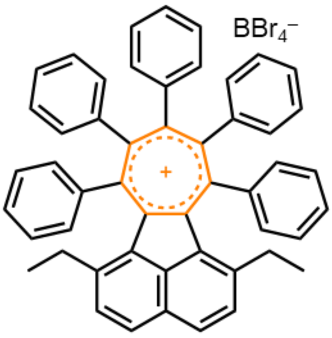

3·BBr₄

3·BBr_4: A solution of BBr_3 (3.3 mg, 13 μmol) in anhydrous CH_2Cl_2 (0.11 mL) was added to a solution of **S1** (5.9 mg, 8.8 μmol) in anhydrous CH_2Cl_2 (0.4 mL) in an oven-dried vial under an inert atmosphere, and the solution was left to stir at rt for 6 h. Anhydrous hexane (0.5 mL) was added, and the vial was placed in the freezer to crystallize. Upon slow evaporation of the solvent, **3·BBr_4** was obtained as an orange, crystalline solid. *Due to the small quantity of crystalline material obtained, yields and NMR spectroscopic data could not be acquired for this compound. The identity of the compound was confirmed by single-crystal X-ray diffraction and mass spectrometry.* **HR-ESI MS** m/z = 651.3055 $[M]^+$ (calculated for $C_{51}H_{39}^+$ = 651.3052).

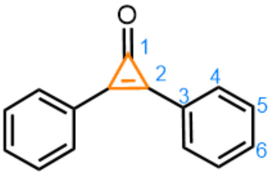

6

Diphenylcyclopropenone (6): Anhydrous hexane (150 mL) was added to diphenylacetylene (6.02 g, 33.8 mmol) and KO^{t}Bu (10.6 g, 94.5 mmol) under an inert atmosphere, and the mixture was cooled to −20 °C. $CHBr_3$ (2.68 mL, 30.6 mmol) was added slowly over 3 h at this

temperature, and the reaction mixture was allowed to warm to rt overnight. The reaction was quenched with H_2O (50 mL) and stirred for 30 min. The layers were separated, and the aqueous layer was extracted with EtOAc (3 × 30 mL). The combined organic extracts were washed with a saturated aqueous solution of $Na_2S_2O_3$ (30 mL) and brine (30 mL), then dried over $MgSO_4$. The solvent was removed under reduced pressure give a dark red viscous oil, which was purified by column chromatography (Teledyne Isco CombiFlash Rf+ system, 80 g SiO_2, hexanes−EtOAc, gradient elution) to yield the title compound as a pale-yellow solid (4.54 g, 21.9 mmol, 65%). **M.P.** 92 – 94 °C. **^{1}H NMR** (400 MHz, $CDCl_3$) δ 8.08 – 7.91 (m, 4H, H_4), 7.71 – 7.52 (m, 6H, $H_{5,6}$). **^{13}C NMR** (101 MHz, $CDCl_3$) δ 156.0 (C_1), 148.5 (C_3), 132.9 (C_4), 131.7 (C_5), 129.5 (C_6), 124.2 (C_2). **HR-ESI MS** *m/z* = 207.0787 $[M+H]^+$ (calculated for $C_{15}H_{11}O^+$ = 207.0810).

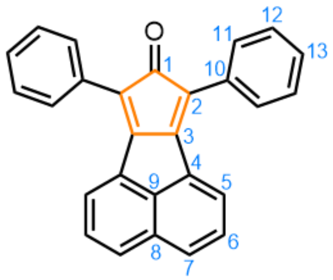

Acecyclone

Acecyclone: A solution of NaOH (790 mg, 19.8 mmol) in EtOH (25 mL) was added dropwise to a refluxing suspension of 1,3-diphenyl-2-propanone (2.60 g, 12.4 mmol) and acenaphthenequinone (2.00 g, 11.0 mmol) in EtOH (50 mL). The reaction mixture was refluxed for 15 min, then immediately placed onto an ice-bath. Once cooled, the resulting precipitate was collected *via* vacuum filtration and washed with cold EtOH (10 mL) to give the title compound as a black solid (2.95 g, 8.24 mmol, 75%). **M.P.** 280 – 282 °C. **^{1}H NMR** (700 MHz, $CDCl_3$) δ 8.07 (dd, J = 7.2, 0.6 Hz, 2H, H_7), 7.90 – 7.85 (m, 2H, H_5), 7.85 – 7.80 (m, 4H, H_{11}), 7.59 (dd, J = 8.2, 7.2 Hz, 2H, H_6), 7.54 – 7.51 (m, 4H, H_{12}), 7.41 (ddt, J = 7.9, 7.0, 1.3 Hz, 2H, H_{13}). **^{13}C NMR** (176 MHz, $CDCl_3$) δ 202.0 (C_1), 154.4 (C_3), 144.9 (C_4), 132.3 (C_2), 131.7 (C_{10}), 131.6 (C_8), 129.2 (C_{11}), 128.7 (C_{12}), 128.6 (C_9), 128.4 (C_6), 127.9 (C_5), 121.8 (C_{13}), 121.1 (C_7). **HR-ESI MS** *m/z* = 357.1256 $[M+H]^+$ (calculated for $C_{27}H_{17}O^+$ = 357.1279).

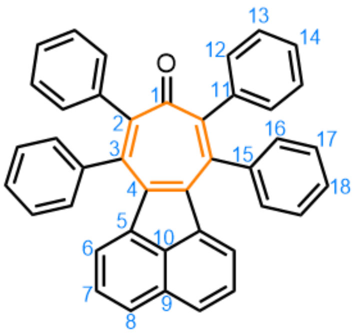

9

9: Anhydrous PhMe (2.5 mL) and anhydrous $CHCl_3$ (1.5 mL) were added to **6** (0.20 g, 0.97 mmol) and acecyclone (0.23 g, 0.65 mmol) in a sealed, oven-dried microwave vial under an inert atmosphere. The mixture was deoxygenated (3 × freeze-pump-thaw cycles under N_2), then stirred for 24 h at 130 °C in a microwave reactor. Upon cooling to rt, the solvent was removed under reduced pressure and the crude solid was purified by column chromatography (Teledyne Isco CombiFlash Rf+ system, 12 g SiO_2, hexanes−EtOAc, 0 – 10% gradient elution). The title compound was isolated as a pale-yellow solid (0.12 g, 0.18 mmol, 34%). **M.P.** 245 – 247 °C. **^{1}H NMR** (700 MHz, $CDCl_3$) δ 7.65 (d, J = 8.1 Hz, H_8), 7.28 – 7.33 (m, 8H, $H_{13,17}$), 7.32 – 7.28 (m, 2H, H_{18}), 7.28 – 7.23 (m, 4H, H_{16}), 7.17 (dd, J = 8.2, 7.2 Hz, H_7), 7.07 – 7.01 (m, 4H, H_{12}), 6.36 (d, J = 7.2 Hz, 2H, H_6). **^{13}C NMR** (176 MHz, $CDCl_3$) δ 197.5 (C_1), 140.2 (C_3), 140.0 (C_{15}), 139.0 (C_2), 138.7 (C_{11}), 136.5 (C_4), 134.1 (C_6), 131.9 (C_{13}), 130.6 (C_{12}), 128.6 (C_5), 128.5 (C_{10}), 128.3 (C_{17}), 127.9 (C_{18}), 127.7 (C_9), 127.6 (C_{16}), 127.5 (C_{14}), 127.4 (C_7), 126.1 (C_8). **HR-ESI MS** m/z = 535.1953 $[M+H]^+$ (calculated for $C_{41}H_{27}O^+$ = 535.1948).

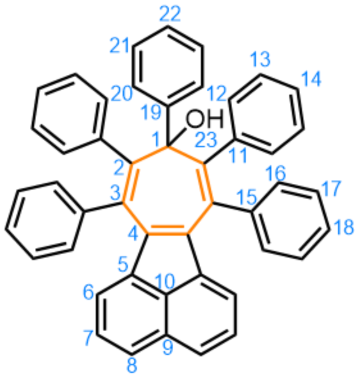

S2

S2: 9 (0.10 g, 0.19 mmol) was placed in a flame-dried two-necked round-bottomed flask under an inert atmosphere. Anhydrous THF (3 mL) was added, and the solution was cooled to 0 °C. PhMgBr (1 M in THF, 0.20 mL, 0.20 mmol) was added slowly, and the mixture was stirred at this temperature for 30 min, then at rt for 3 h. The reaction was quenched with a saturated aqueous solution of NH_4Cl (5 mL) and extracted with

Et_2O (2 ×10 mL). The combined organic extracts were dried over $MgSO_4$, and the solvent was removed under reduced pressure. The crude product was purified by column chromatography (Teledyne Isco CombiFlash Rf+ system, 12 g SiO_2, hexanes−EtOAc, 0 – 10% gradient elution), yielding **12** as a yellow solid (0.080 g, 0.13 mmol, 68%). **M.P.** 205 – 207 °C. **^{1}H NMR** (700 MHz, $CDCl_3$) δ 7.80 – 7.75 (m, 2H, H_{20}), 7.44 (d, *J* = 8.2 Hz, 2H, H_8), 7.30 – 7.27 (m, 4H, H_{16}), 7.30 – 6.90 (br, 8 H, $H_{12,13}$) 7.16 – 7.13 (m, 6H, $H_{17,18}$), 7.08 (t, 2H, *J* = 7.4 Hz, H_{14}), 7.04 (dd, 2H, *J* = 8.2, 7.1 Hz, H_7), 6.91 – 6.87 (m, 2H, H_{21}), 6.70 – 6.55 (m, 1H, H_{22}), 6.24 (d, 2H, *J* = 7.2 Hz, H_6), 1.84 (s, 1H, H_{23}). **^{13}C NMR** (176 MHz, $CDCl_3$) δ 144.2 (C_{19}), 142.5 (C_4), 141.9 (C_{15}), 140.6 (C_2), 138.8 (C_9), 137.2 ($C_{12/13}$**)**, 133.5 (C_3), 133.2 (C_{14}), 131.9 (C_{16}), 129.8 ($C_{13/12}$) 128.4 (C_{10}), 128.0 (C_5), 127.9 (C_{17}), 127.4 (C_{14}), 127.3, ($C_{7,21,22}$), 126.9 (C_{18}), 126.6 (C_{20}), 126.5 (C_8), 124.4 (C_6), 80.5 (C_1). **HR-ESI MS** *m/z* = 595.2440 [M–OH]$^+$ (calculated for $C_{47}H_{31}^+$ = 595.2426).

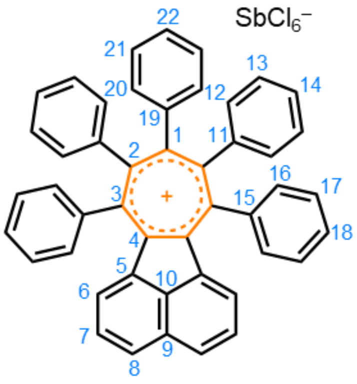

2·SbCl6

2·$SbCl_6$: $Et_3O·SbCl_6$ (5.0 mg, 11 μmol) was added to a solution of **S2** (3.6 mg, 5.9 μmol) in anhydrous $CDCl_3$ (0.6 mL) under an inert atmosphere at rt, causing the initially yellow solution to turn orange immediately. NMR spectroscopic analysis indicated quantitative conversion of the starting material to **2**·$SbCl_6$. **^{1}H NMR** (700 MHz, $CDCl_3$) δ 8.22 (d, *J* = 8.0 Hz, 2H, H_8), 7.48 (t, *J* = 7.9 Hz, 2H, H_7), 7.45 – 7.42 (m, 4H, H_{16}), 7.37 – 7.30 (m, 6H, $H_{17,18}$), 6.90 – 6.84 (m, 8H, $H_{13,14,20}$), 6.82 – 6.76 (m, 6H, $H_{12,21}$), 6.75 – 6.71 (m, 1H, H_{22}), 6.38 (d, *J* = 7.8 Hz, 2H, H_6). **^{13}C NMR** (176 MHz, $CDCl_3$) δ 162.2 (C_1), 162.1 (C_2), 159.6 (C_3), 157.2 (C_4), 141.1 ($C_{15/19}$), 141.1 ($C_{19/15}$), 140.4 (C_{11}), 135.4 (C_5), 135.3 (C_8), 135.2 (C_{10}), 134.5 (C_6), 129.9 (C_{20}), 129.8 (C_{21}), 129.8 (C_{16}), 129.6 (C_7), 129.4 (C_9), 129.0

(C_{18}), 128.7 (C_{17}), 127.1 (C_{13}), 126.9 (C_{14}), 126.9 (C_{12}), 126.8 (C_{22}). **HR-ESI MS** *m/z* = 595.2440 $[M]^+$ (calculated for $C_{47}H_{31}^+$= 595.2426).

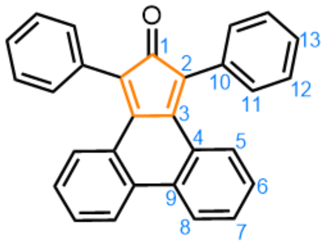

Phencyclone

Phencyclone: A solution of NaOH (3.30 g, 81.9 mmol) in EtOH (90 mL) was added dropwise to a refluxing suspension of 1,3-diphenyl-2-propanone (9.91 g, 46.0 mmol) and phenanthrenequinone (9.06 g, 43.5 mmol) in EtOH (300 mL). The reaction mixture was refluxed for 15 min, then immediately placed onto an ice-bath. Once cooled, the resulting precipitate was collected *via* vacuum filtration and washed with cold EtOH (10 mL) to give the title compound as a black solid (14.8 g, 38.7 mmol, 89%). **M.P.** 255 – 257 °C. ^{1}**H NMR** (400 MHz, $CDCl_3$) δ 6.95 (t, *J* = 7 Hz, 2H), 7.28–7.30 (m, 2H), 7.36 – 7.45 (m, 10H), 7.55 (dd, *J* = 8, 1 Hz, 2H), 7.81 (d, *J* = 7.5 Hz, 2H). The spectroscopic data was consistent with literature reports.[5]

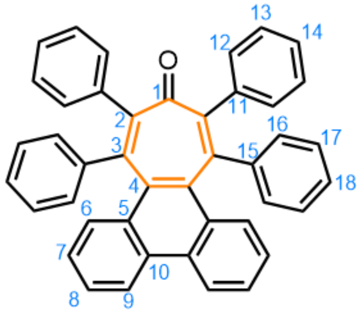

10

10: Anhydrous PhMe (10 mL) was added to phencyclone (0.49 g, 1.2 mmol) and **6** (0.40 g, 1.9 mmol) in a sealed, oven-dried microwave vial under an inert atmosphere. The mixture was deoxygenated (3 × freeze-pump-thaw cycles under N_2), then stirred for 24 h at 130 °C in a microwave reactor. Upon cooling to rt, the solvent was removed under reduced pressure, and the crude solid was purified by column chromatography (Teledyne Isco CombiFlash Rf+ system, 12 g SiO_2, hexanes−CH_2Cl_2, 0 – 30% gradient elution), to yield **10** as a colorless solid (0.31 g, 0.48 mmol, 40%). **M.P.** 248 – 250 °C. ^{1}**H NMR** (700 MHz, $CDCl_3$) δ 8.59 (dd, *J* = 8.4, 1.3 Hz, 2H, H_9), 7.94 (dd, *J* = 8.5, 1.3 Hz, 2H, H_6), 7.47 (ddd, *J* = 8.3, 6.9, 1.2 Hz, 2H,

H$_{8}$), 7.20 (ddd, J = 8.3, 6.9, 1.2 Hz, 2H, H$_{7}$), 7.15 – 7.13 (m, 4H, H$_{17}$), 7.13 – 7.09 (m, 10H, H$_{12,13,14}$), 7.03 – 6.97 (m, 6H, H$_{18,16}$). **^{13}C NMR** (176 MHz, CDCl$_{3}$) δ 199.9 (C$_{1}$), 146.2 (C$_{3}$), 140.6 (C$_{15}$), 135.8 (C$_{11}$), 134.9 (C$_{10}$), 133.6 (C$_{2}$), 131.1 (C$_{4}$), 130.7 (C$_{6}$), 130.4 (C$_{13}$), 129.8 (C$_{5}$), 129.7 (C$_{12}$), 128.3 (C$_{17}$), 128.0 (C$_{16}$), 127.8 (C$_{18}$), 127.2 (C$_{14}$), 126.9 (C$_{8}$), 125.9 (C$_{7}$), 122.4 (C$_{9}$). **HR-ESI MS** m/z = 561.2222 [M+H]$^{+}$ (calculated for $C_{43}H_{29}O^{+}$ = 561.2218).

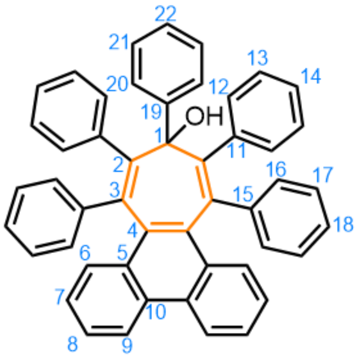

S3

S3: 10 (0.10 g, 0.18 mmol) was placed in an oven-dried two-necked round-bottomed flask under an inert atmosphere. Anhydrous THF (5 mL) was added, and the solution was cooled to 0 °C. PhMgBr (1 M in THF, 0.2 mL, 0.2 mmol) was added slowly, and the mixture was stirred at this temperature for 30 min, then at rt for 3 h. The reaction was quenched with a saturated aqueous solution of NH_4Cl (5 mL) and extracted with Et_2O (2 ×10 mL). The combined organic extracts were dried over $MgSO_4$, and the solvent was removed under reduced pressure. The crude solid was purified by column chromatography (Teledyne Isco CombiFlash Rf+ system, 12 g SiO_2, hexanes−EtOAc, 0 – 5% gradient elution). The title product was isolated as a colorless solid (0.070 g, 0.11 mmol, 60%). **M.P.** 340 – 342 °C. **^{1}H NMR** (700 MHz, CDCl$_{3}$) δ 8.32 (d, J = 8.3 Hz, 2H, H$_{9}$), 7.85 (d, J = 8.3 Hz, 2H, H$_{6}$), 7.78 (d, J = 7.7 Hz, 2H, H$_{16}$), 7.62 – 7.57 (m, 2H, H$_{20}$), 7.41 – 7.36 (m, 2H, H$_{17}$), 7.34 – 7.28 (m, 2H, H$_{8}$), 7.19 – 7.08 (m, 4H, H$_{7,17'}$), 7.04 – 6.95 (m, 10H, H$_{12,13,14}$), 6.94 – 6.87 (m, 2H, H$_{18}$), 6.84 – 6.79 (m, 2H, H$_{16'}$), 6.57 – 6.49 (m, 3H, H$_{21,22}$). **^{13}C NMR** (176 MHz, CDCl$_{3}$) δ 151.4 (C$_{15}$), 143.0 (C$_{3}$), 142.3 (C$_{19}$), 137.6 (C$_{2}$), 136.7 (C$_{11}$), 133.9 (C$_{10}$), 132.6 (C$_{16}$), 132.2 (C$_{16'}$), 130.9 (C$_{12}$), 130.1 (C$_{4}$), 129.9 (C$_{6}$), 128.9 (C$_{5}$), 127.6 (C$_{18}$), 127.3 (C$_{13}$), 127.2 (C$_{17}$), 127.2 (C$_{17'}$), 126.7 (C$_{21}$), 126.4 (C$_{22}$), 125.8 (C$_{8}$), 125.6 (C$_{14}$), 125.3 (C$_{20}$), 125.1 (C$_{7}$), 121.7 (C$_{9}$), 81.1 (C$_{1}$). **HR-ESI MS** m/z = 621.2560 [M-OH]$^{+}$ (calculated for $C_{49}H_{33}^{+}$ = 621.2582).

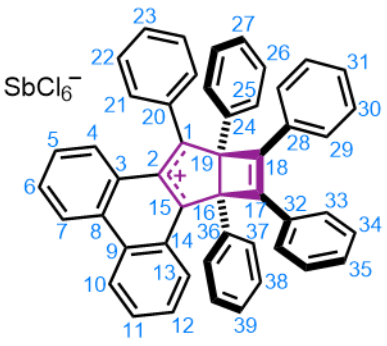

4·SbCl$_6$

4·SbCl$_6$: $Et_3O·SbCl_6$ (5.0 mg, 11 µmol) was added to a solution of **S3** (3.5 mg, 5.5 µmol) in anhydrous CD_2Cl_2 (0.6 mL) under an inert atmosphere at rt, causing the initially colorless solution to turn purple. The solution was left to stand for 4 h. NMR spectroscopic analysis indicated quantitative conversion of the starting material to **4**·$SbCl_6$. **^{1}H NMR** (500MHz, CD_2Cl_2, 179 K) δ 8.84 (d, J = 8.6 Hz, 1H, H_7), 8.79 (d, J = 8.5 Hz, 1H, H_{10}), 8.33 (d, J = 8.2 Hz, 1H, H_{13}), 8.25 – 8.18 (m, 1H, H_6), 7.95 – 7.87 (m, 1H, H_{11}), 7.81 (d, 1H, J = 8.4 Hz, H_4), 7.78 – 7.72 (m, 1H, H_{29}), 7.67 – 7.56 (m, 3H, $H_{12,37,39}$), 7.50 – 7.39 (m, 3H, $H_{5,29',37'}$), 7.38 – 7.32 (m, 2H, $H_{27,33'}$), 7.31 – 7.17 (m, 6H, $H_{26,30,38}$), 7.16 – 7.09 (m, 1H, H_{31}), 7.05 (t, J = 7.4 Hz, 1H, H_{23}), 7.00 (t, J = 7.6 Hz, 1H, H_{35}), 6.89 (t, J = 7.7 Hz, 2H, H_{22}), 6.84 – 6.79 (m, 2H, H_{21}), 6.76 (t, J = 7.7 Hz, 2H, H_{34}), 6.20 (m, 1H, H_{33}). **^{13}C NMR** (126 MHz, CD_2Cl_2, 179 K) δ 185.2 (C_1), 152.6 (C_{15}), 142.7 (C_2), 141.5 (C_6), 141.1 (C_{36}), 139.7 (C_{32}), 139.7 (C_{20}), 139.0 (C_{28}), 138.9 (C_{24}), 138.8 (C_{12}), 136.4 (C_9), 135.7 (C_4), 134.2 (C_8), 134.2 (C_3), 133.6 (C_{39}), 132.9 (C_{14}), 131.7 (C_{21}), 131.0 ($C_{11,33'}$), 129.9 (C_{37}), 129.7 (C_{25}), 129.6 (C_{26}), 129.2 (C_{31}), 129.1 (C_{23}), 128.9 (C_{27}), 128.9 (C_5), 128.8 (C_{30}), 128.8 (C_{38}), 128.6 (C_{19}), 128.4 (C_{22}), 128.4 (C_{33}), 128.0 (C_{35}), 128.0 (C_{34}), 127.6 (C_{16}), 127.5 (C_{29}), 127.35 (C_{18}), 127.0 (C_{13}), 126.75 (C_{17}), 125.0 (C_7), 124.8 (C_{10}). **HR-ESI MS** m/z = 621.2560 $[M]^+$ (calculated for $C_{49}H_{33}^+$ = 621.2582).

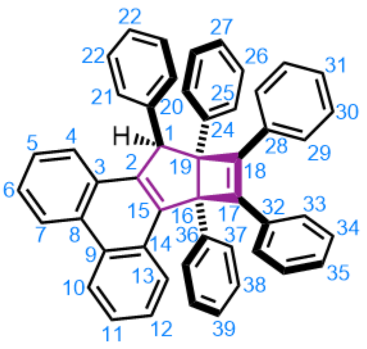

anti-**11**

anti-**11**: A solution of $LiAlH_4$ (1 M in THF, 0.15 mL, 150 µmol) was added to a solution of **4**·OTf (12 mg, 13 µmol) in anhydrous dioxane (1 mL), and the mixture was stirred at rt for 20 min. The reaction was quenched with water (5 mL) and extracted with EtOAc (3 × 5 mL). The combined organic extracts were washed

with brine (10 mL), then dried over $MgSO_4$. The solvent was removed *in vacuo*, leaving a pale-yellow residue, which was purified by preparative TLC (SiO_2, hexanes–CH_2Cl_2, 7:3) to yield the title compound as a colorless solid (5.6 mg, 9.0 µmol, 72%). **^{1}H NMR** (600 MHz, $CDCl_3$) δ 8.75 (d, J = 8.4 Hz, 1H, H_7), 8.72 (d, J = 8.4 Hz, 1H, H_{10}), 7.63 (d, J = 8.2 Hz, 1H, H_4), 7.59 – 7.56 (m, 1H, H_6), 7.52 – 7.48 (m, 1H, H_{11}), 7.48 – 7.42 (m, 4H, $H_{21,29,30'}$), 7.38 – 7.35 (m, 1H, H_5), 7.35 – 7.32 (m, 2H, H_{25}), 7.29 (d, J = 8.3 Hz, 1H, H_{13}), 7.27 – 7.24 (m, 2H, $H_{29',30}$), 7.11 – 7.07 (m, 3H, $H_{12,22}$), 7.04 – 6.95 (m, 4H, $H_{26,27,31}$), 6.87 – 6.81 (m, 2H, $H_{35,39}$), 6.80 – 6.76 (m, 3H, $H_{23,37}$), 6.74 – 6.70 (m, 3H, $H_{34,38}$), 6.68 – 6.85 (m, 1H, $H_{33'}$), 6.52 – 6.48 (m, 1H, $H_{34'}$), 6.47 – 6.44 (m, 1H, H_{33}), 5.95 (s, 1H, H_1). **^{13}C NMR** (151 MHz, $CDCl_3$) δ 148.1 (C_{18}), 142.4 (C_{32}), 142.1 (C_{20}), 141.9 (C_{24}), 141.5 (C_2), 140.5 (C_{17}), 139.0 (C_{15}), 136.7 (C_{28}), 133.7 (C_{36}), 131.7 (C_9), 131.7 (C_8), 130.0 ($C_{33'}$), 130.0 (C_{21}), 129.9 (C_3), 129.8 (C_{33}), 129.4 (C_{25}), 129.3 (C_{29}), 129.2 (C_{13}), 128.6 (C_{30}), 128.2 ($C_{34'}$), 128.0 (C_{37}), 127.9 ($C_{22/29'}$), 127.9 $C_{29'/22}$), 127.8 ($C_{30'}$), 127.5 (C_{26}), 127.5 (C_{34}), 127.3 (C_{31}), 126.9 (C_{38}), 126.8 (C_{23}), 126.5 (C_5), 126.4 (C_4), 126.3 ($C_{35/39}$), 126.1 (C_6), 126.1 (C_{27}), 126.1 (C_{11}) 125.9 ($C_{39/35}$), 125.6 (C_{12}), 123.2 (C_7), 122.8 (C_{10}), 76.5 (C_{16}), 71.5 (C_{19}), 57.2 (C_1).

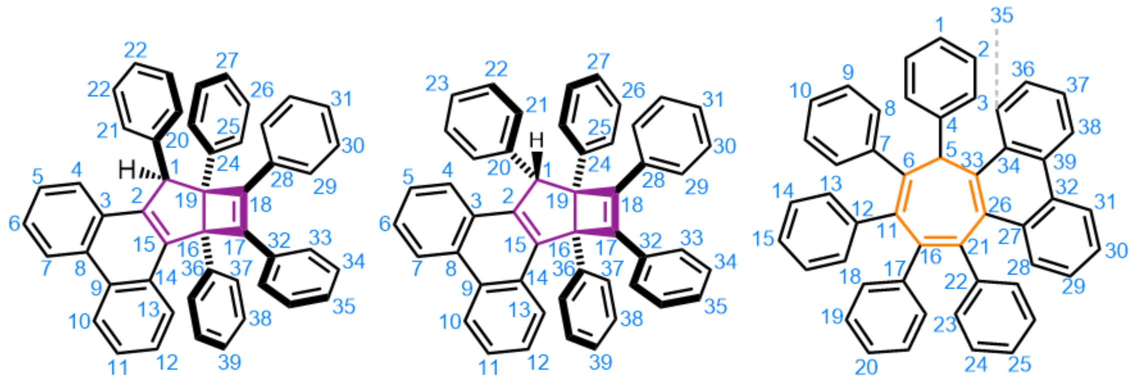

anti-**11** *syn*-**11** **12**

$NaBH_4$ (20 mg, 530 μmol) was added to a solution of **4**·$SbCl_6$ (7.2 mg, 7.8 μmol) in CD_2Cl_2 (0.5 mL), and the solvent was removed *in vacuo*. Anhydrous THF (0.5 mL) was added, and the mixture was stirred for 20 min. Water (10 mL) was added, and the aqueous layer was extracted with EtOAc (3 × 5 mL). The combined organic extracts were washed with brine (10 mL) and dried over $MgSO_4$. The solvent was removed *in vacuo*, leaving a colorless oil, which was passed through a silica plug, eluting in hexanes–CH_2Cl_2 (1:1). Evaporation to dryness afforded a mixture of *anti*-**11**, *syn*-**11**, and **12** as a colorless solid. **^{1}H NMR** spectroscopic analysis (Figure S37 and S38) showed *anti*-**11**, *syn*-**11**, and **12** were present in a 38:58:4 ratio (96:4 ratio of bicycloheptadiene:cycloheptatriene isomers). The signals in the mixture corresponding to **12** could be identified unambiguously by comparison with NMR data from a pure sample which we have previously synthesized by an alternative synthetic route.[6] For comparison, the characterization data for **12** are reproduced below:

^{1}H NMR (700 MHz, $CDCl_3$) δ 8.76 (dd, J = 8.4, 1.2 Hz, 2H, H_{28}), 8.64 (dd, J = 8.4, 1.2 Hz, 1H, H_{38}), 8.16 (d, J = 8.4 Hz, 2H, H_{31}), 7.96 – 7.92 (m, 1H, H_{35}), 7.66 (ddd, J = 8.2, 6.9, 1.2 Hz, 1H, H_{29}), 7.58 (d, J = 7.6 Hz, 2H, H_{18}), 7.55 (m, 1H, H_{30}), 7.46 (m, 1H, H_{37}), 7.28 (m, 2H, H_{19}), 7.25 (m, 1H, H_{36}), 7.23 (m, 1H, H_{20}), 7.11 (m, 3H, $H_{13,15}$), 7.02 – 6.95 (m, 5H, $H_{8–10}$), 6.86 (m, 1H, H_1), 6.78 (m, 1H, H_{25}), 6.73 (m, 2H, H_{24}), 6.71 (m, 2H, H_{23}), 6.68 (m, 2H, H_2), 6.36 (s, 1H, H_5), 5.70 (d, J = 7.7 Hz, 2H, H_3). **^{13}C NMR** (176 MHz, $CDCl_3$) δ 143.8 (C_6), 143.5 (C_{26}), 143.4

(C_{16}), 143.1 (C_{11}), 142.7 (C_{21}), 142.5 (C_{33}), 142.1 (C_4), 140.7 (C_{22}), 140.2 (C_{17}), 139.5 (C_{12}), 138.4 (C_7), 138.0 (C_{39}), 132.2 (C_{32}), 131.7 (C_{23}), 131.5 (C_{34}), 131.5 (C_{27}), 131.2 (C_3), 129.5 (C_{35}), 129.43 (C_{14}), 129.0 ($C_{8/9/10}$), 128.7 (C_{36}), 127.7 ($C_{9/8/10}$), 127.67 (C_{15}), 127.6 (C_{30}), 127.1 (C_{18}), 126.9 (C_{29}), 126.79 (C_{24}), 126.7 (C_{13}), 126.5 (C_{20}), 126.5 (C_2), 126.3 ($C_{10/9/8}$), 125.9 (C_{19}), 125.8 (C_{37}), 125.7 (C_{25}), 125.7 (C_1), 124.4 (C_{31}), 123.2 (C_{28}), 122.7 (C_{38}), 50.3 (C_5). **HR-ASAP MS** *m/z* = 622.2662 $[M]^+$ (calculated for $C_{49}H_{34}^+$: 622.2655).

3. ^{1}H and ^{13}C NMR Spectroscopic Characterization of Synthesized Compounds

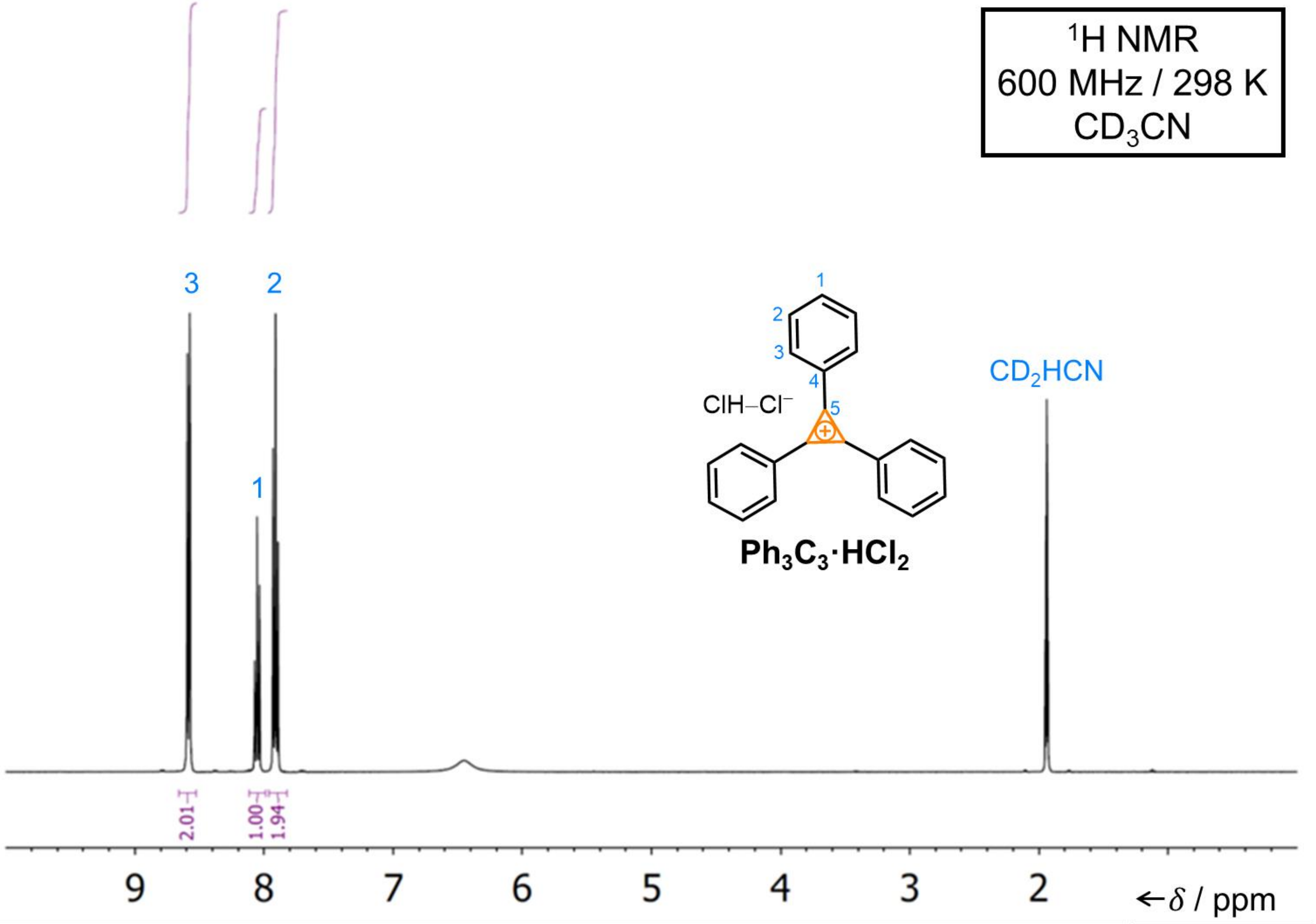

Fig. S2. ^{1}H NMR spectrum of **Ph_3C_3**·HCl_2.

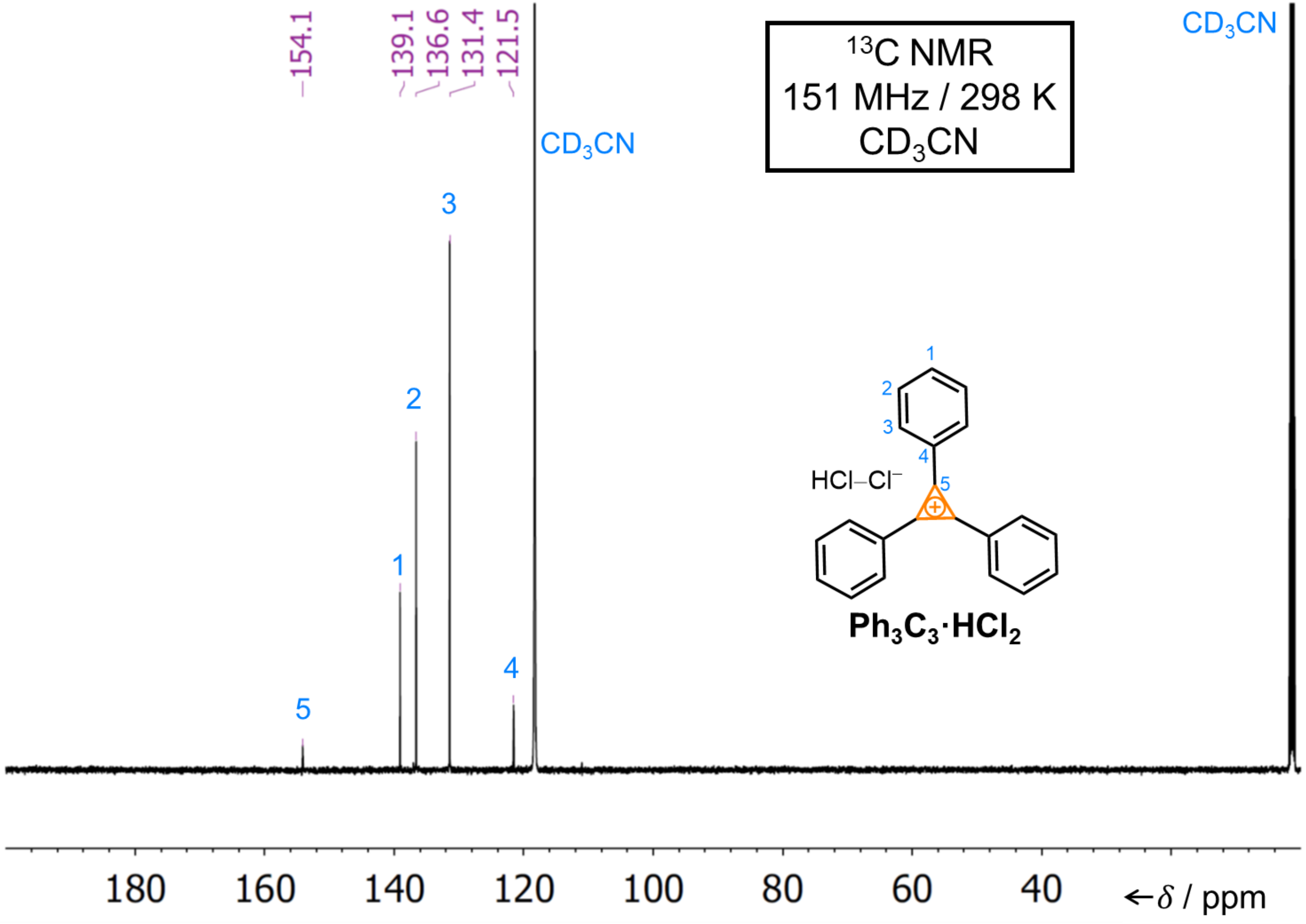

Fig. S3. ^{13}C NMR spectrum of **Ph$_3$C$_3$**·HCl$_2$.

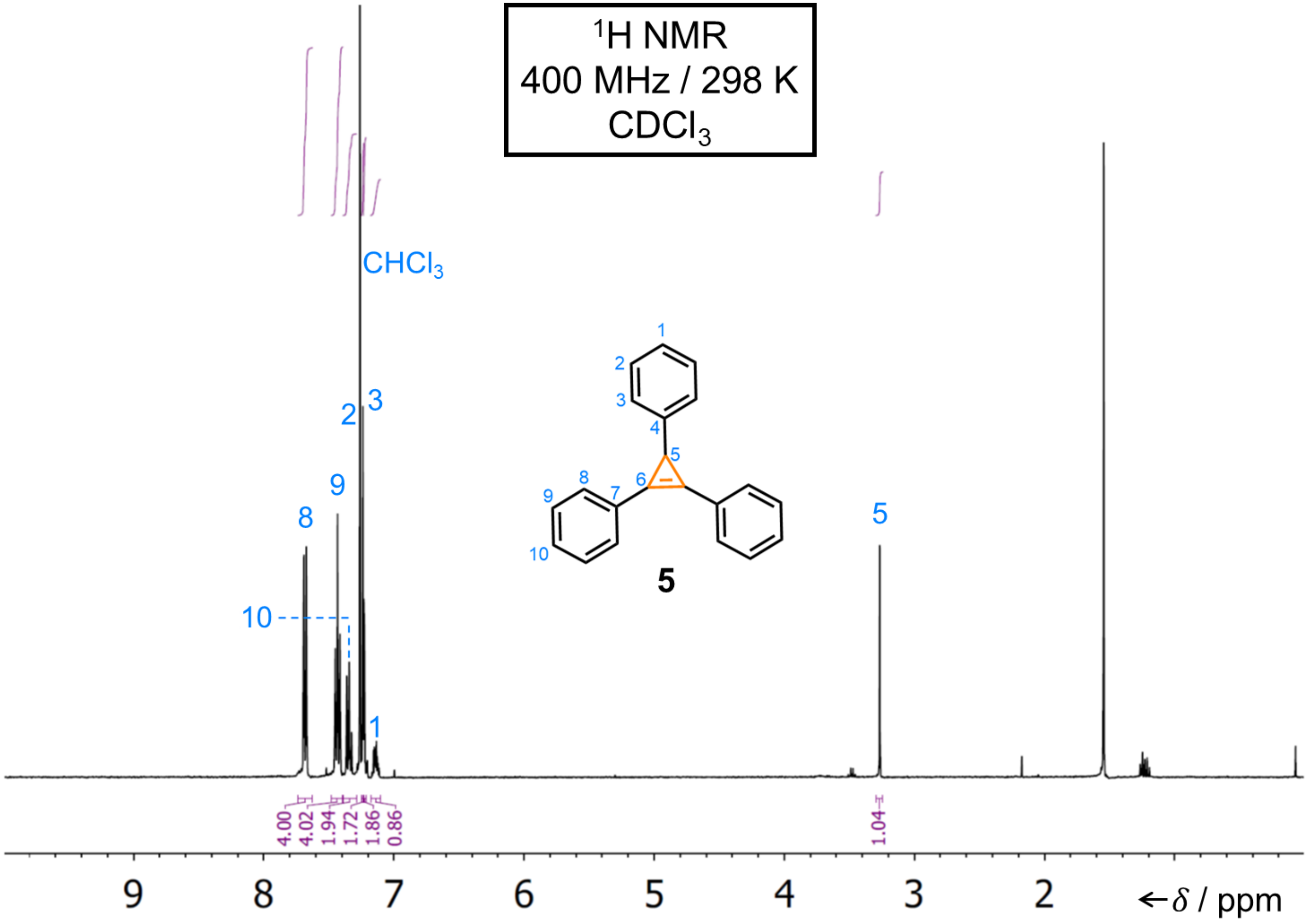

Fig. S4. ^{1}H NMR spectrum of **5**.

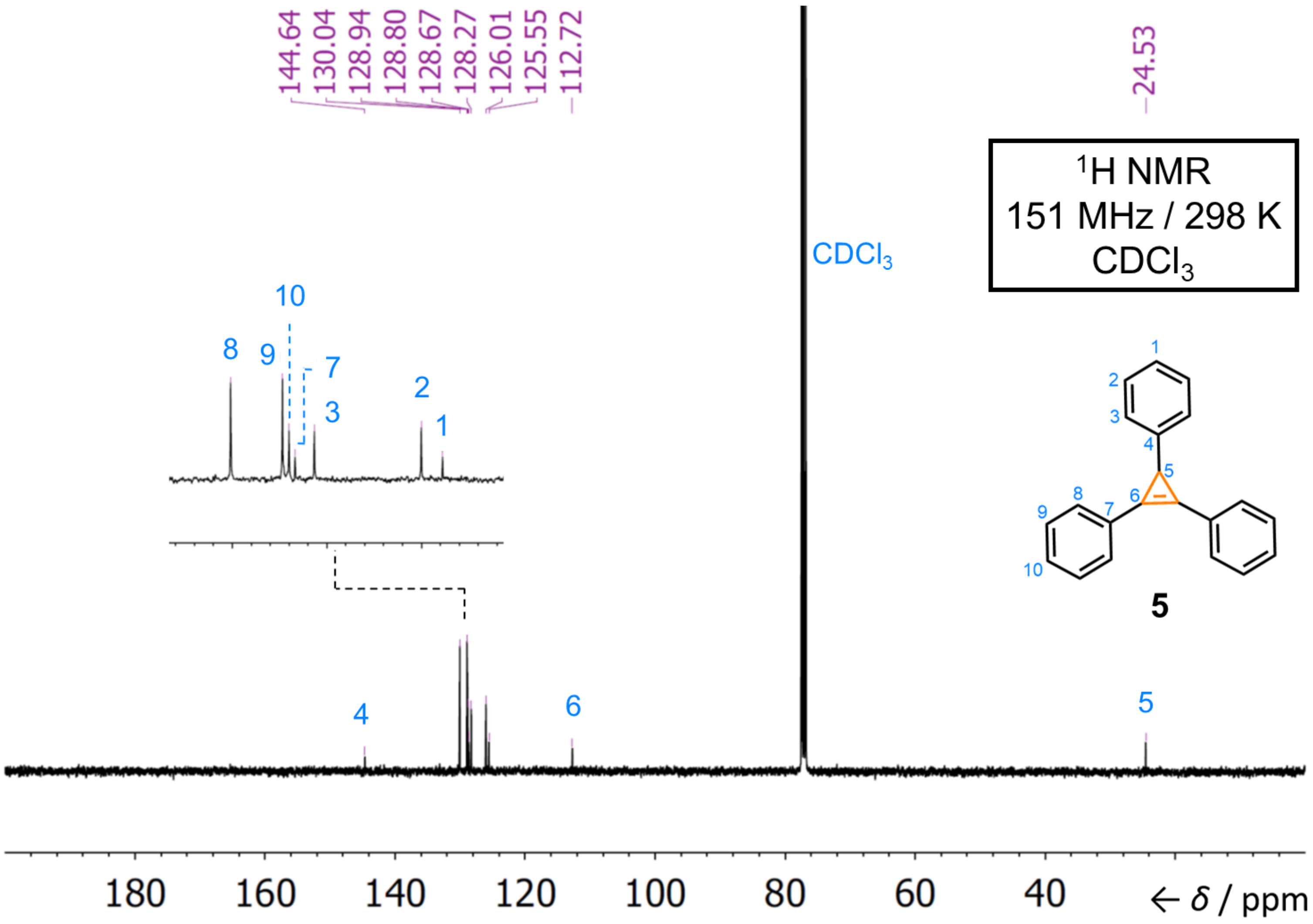

Fig. S5. ^{13}C NMR spectrum of **5**.

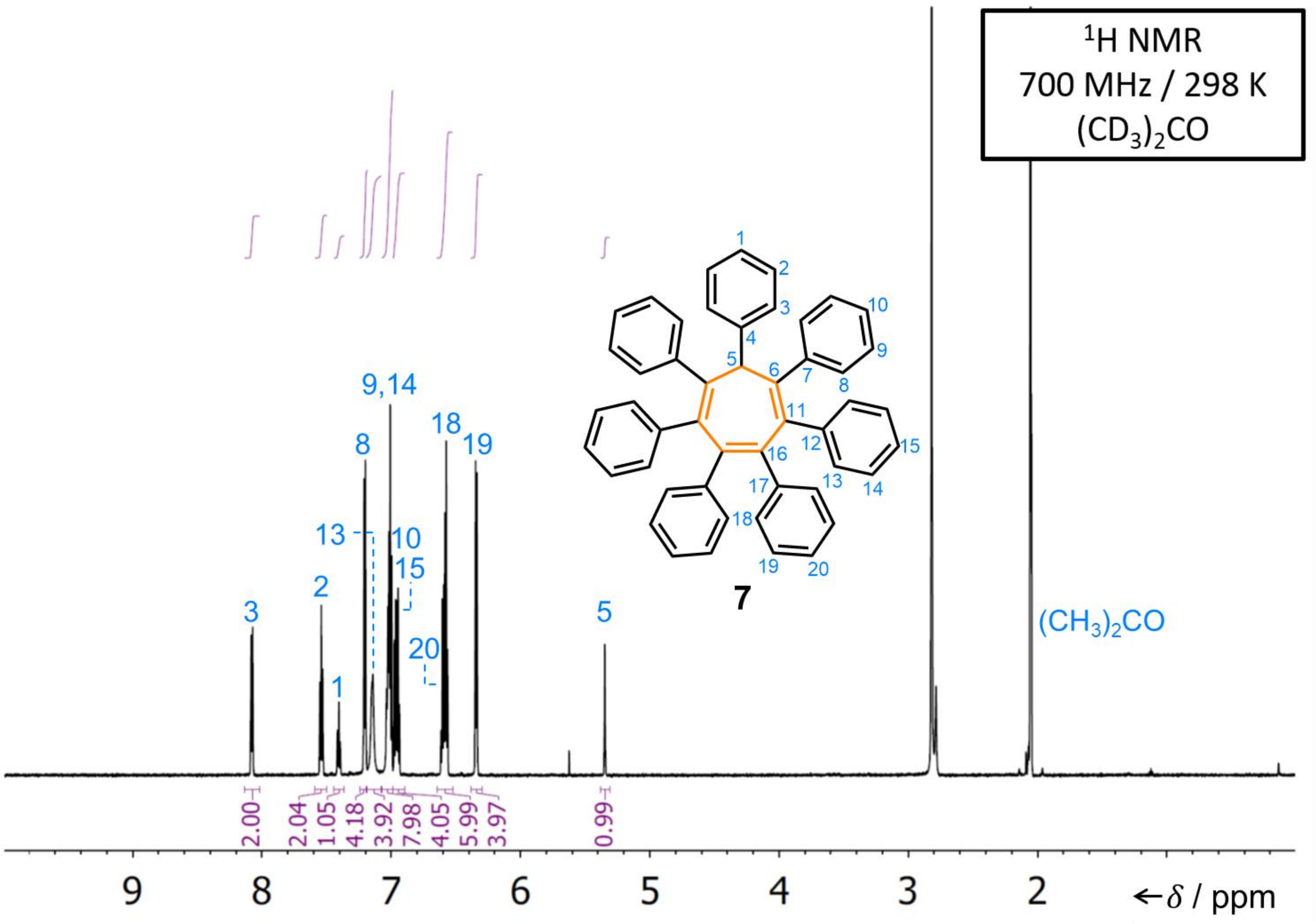

Fig. S6. ^{1}H NMR spectrum of **7.**

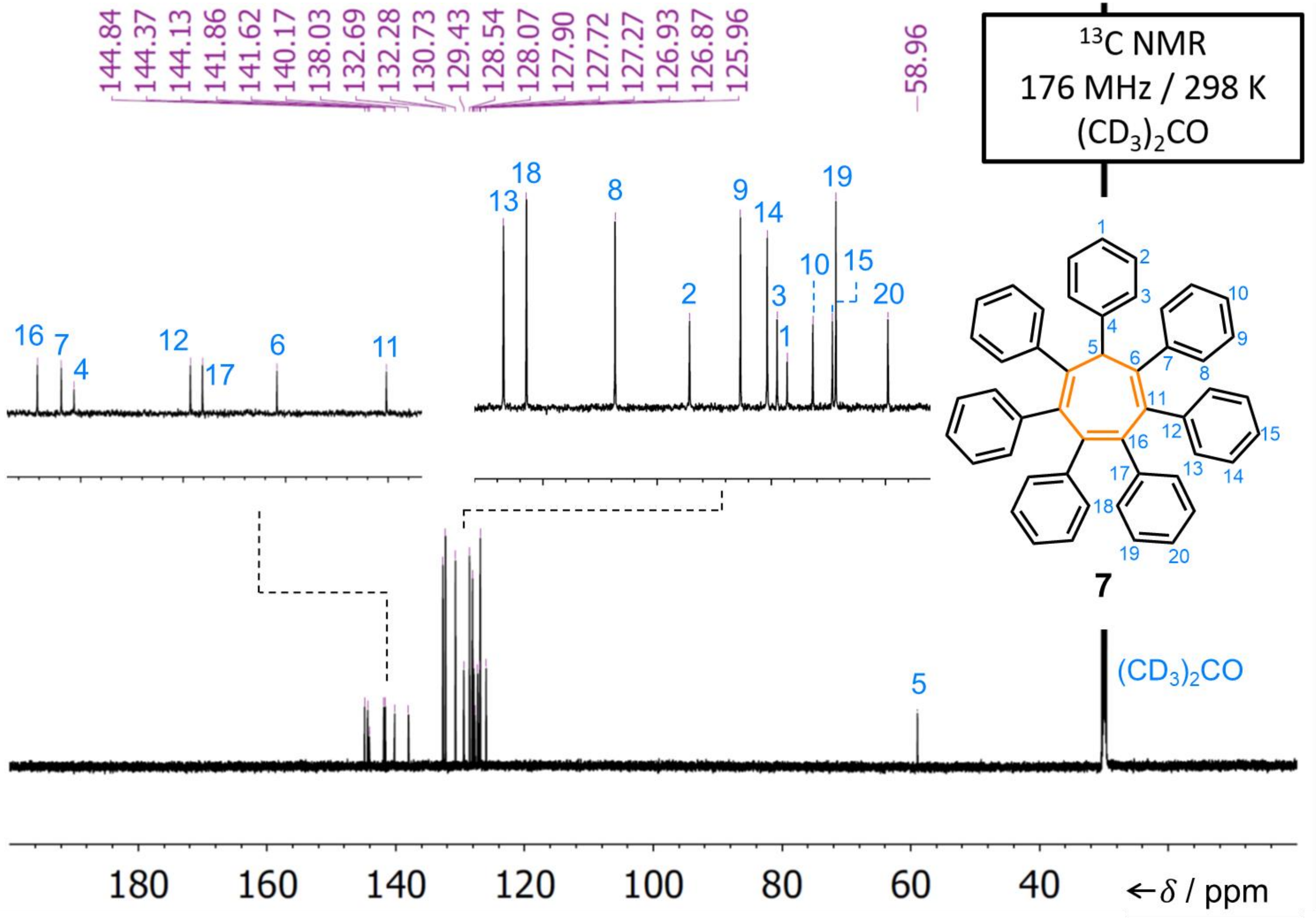

Fig. S7. ^{13}C NMR spectrum of **7.**

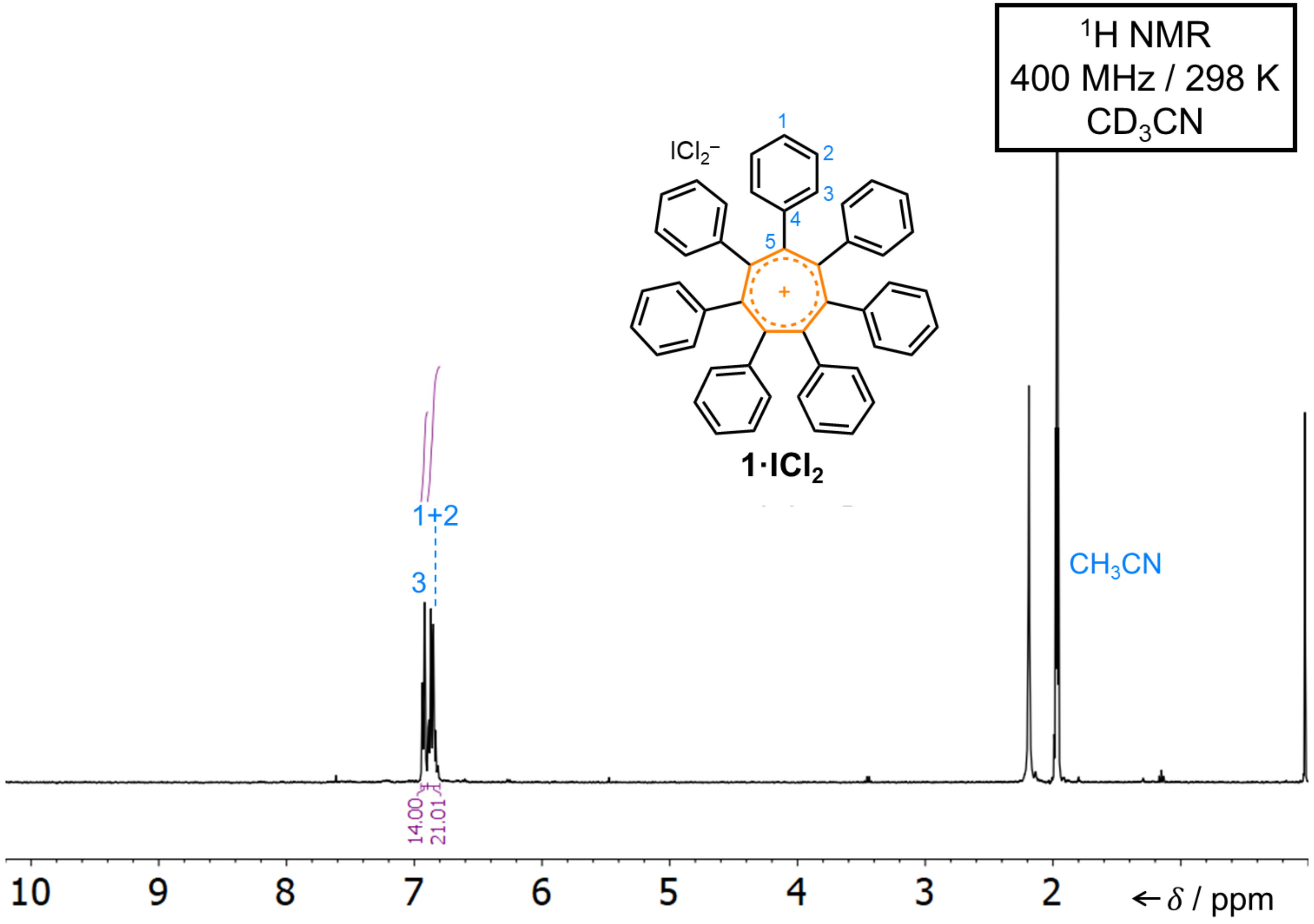

Fig. S8. ^{1}H NMR spectrum of **1**·ICl_2.

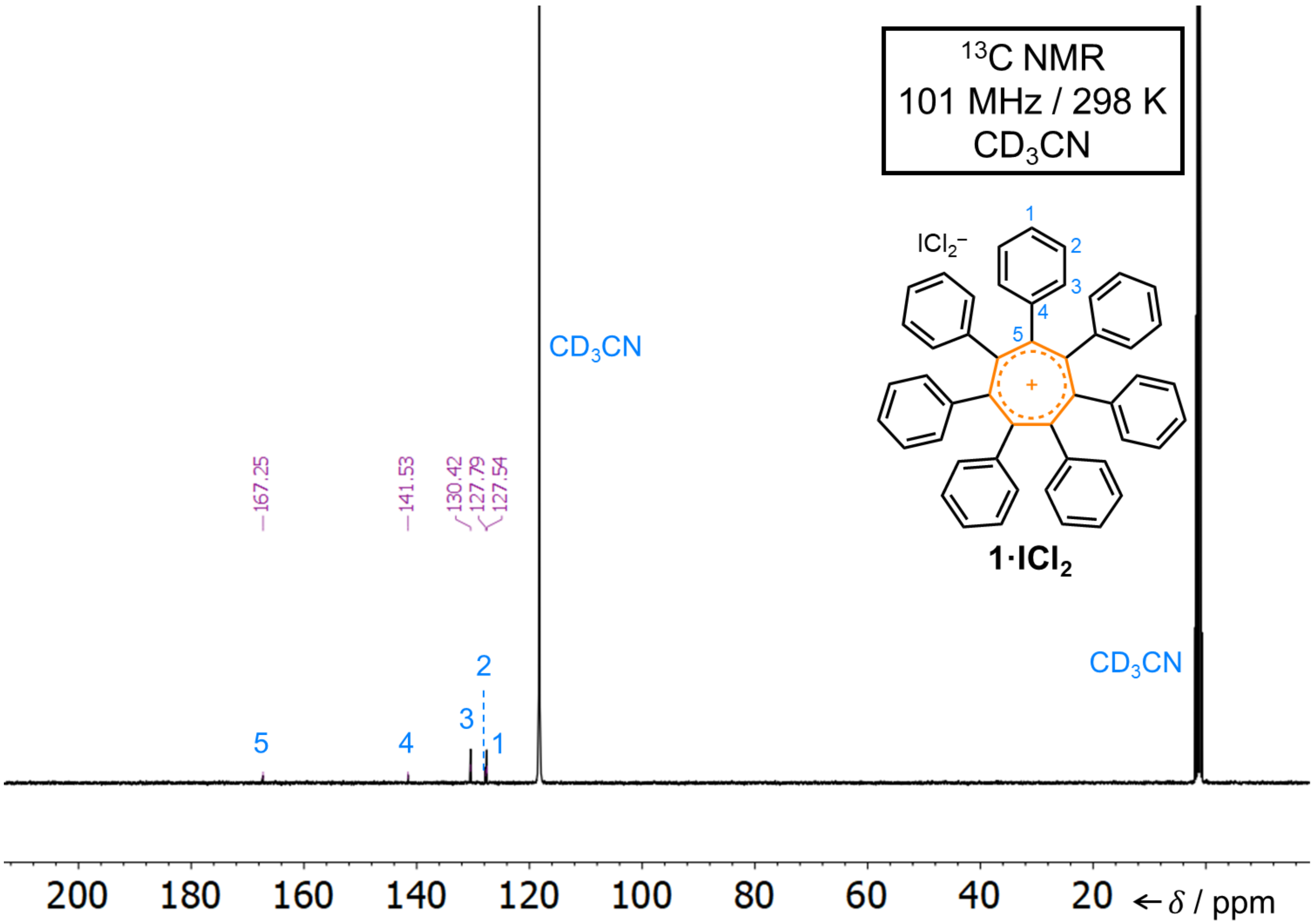

Fig. S9. ^{13}C NMR spectrum of **1**·ICl_2.

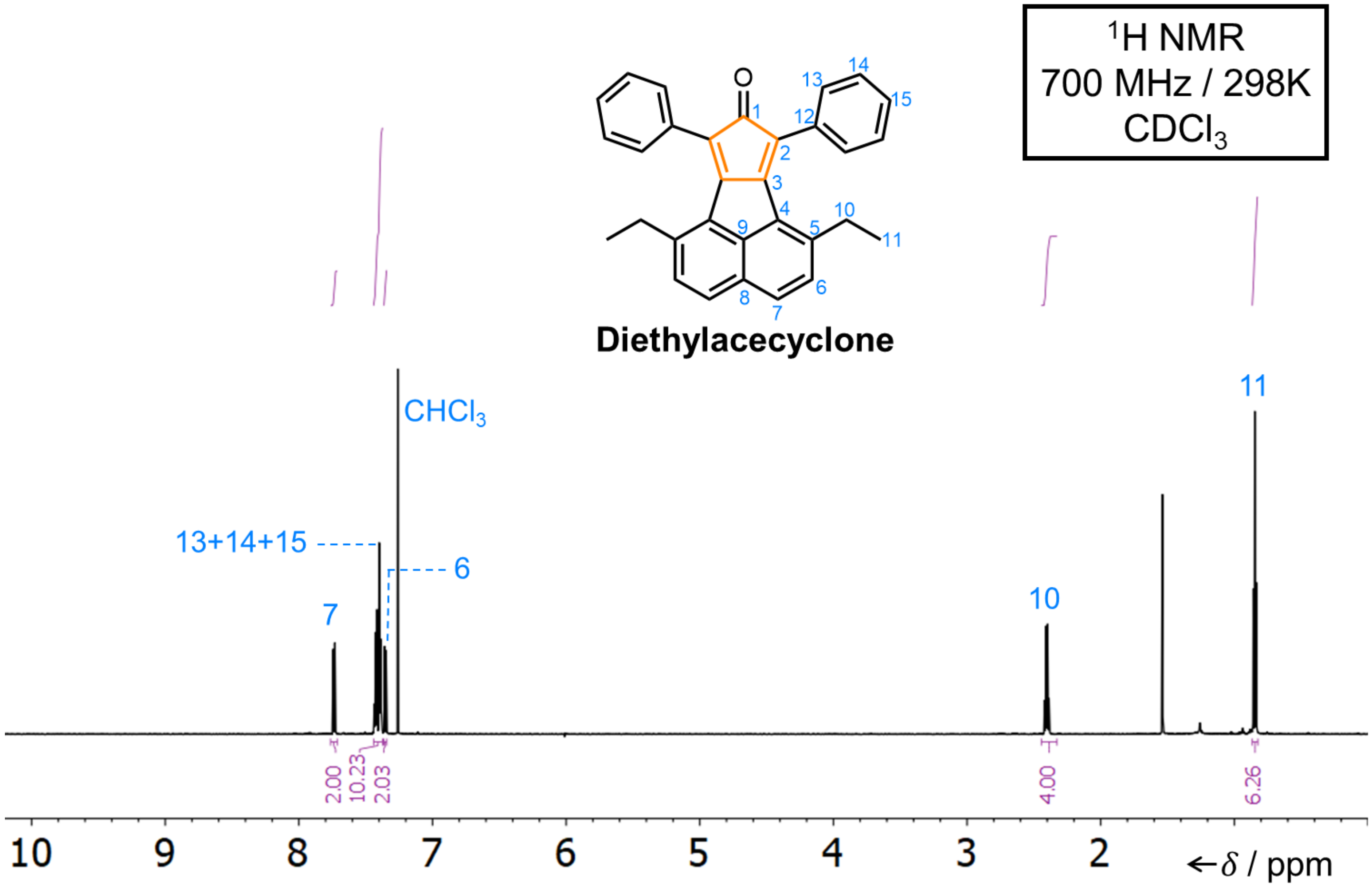

Fig. S10. ^{1}H NMR spectrum of **diethylacecyclone**.

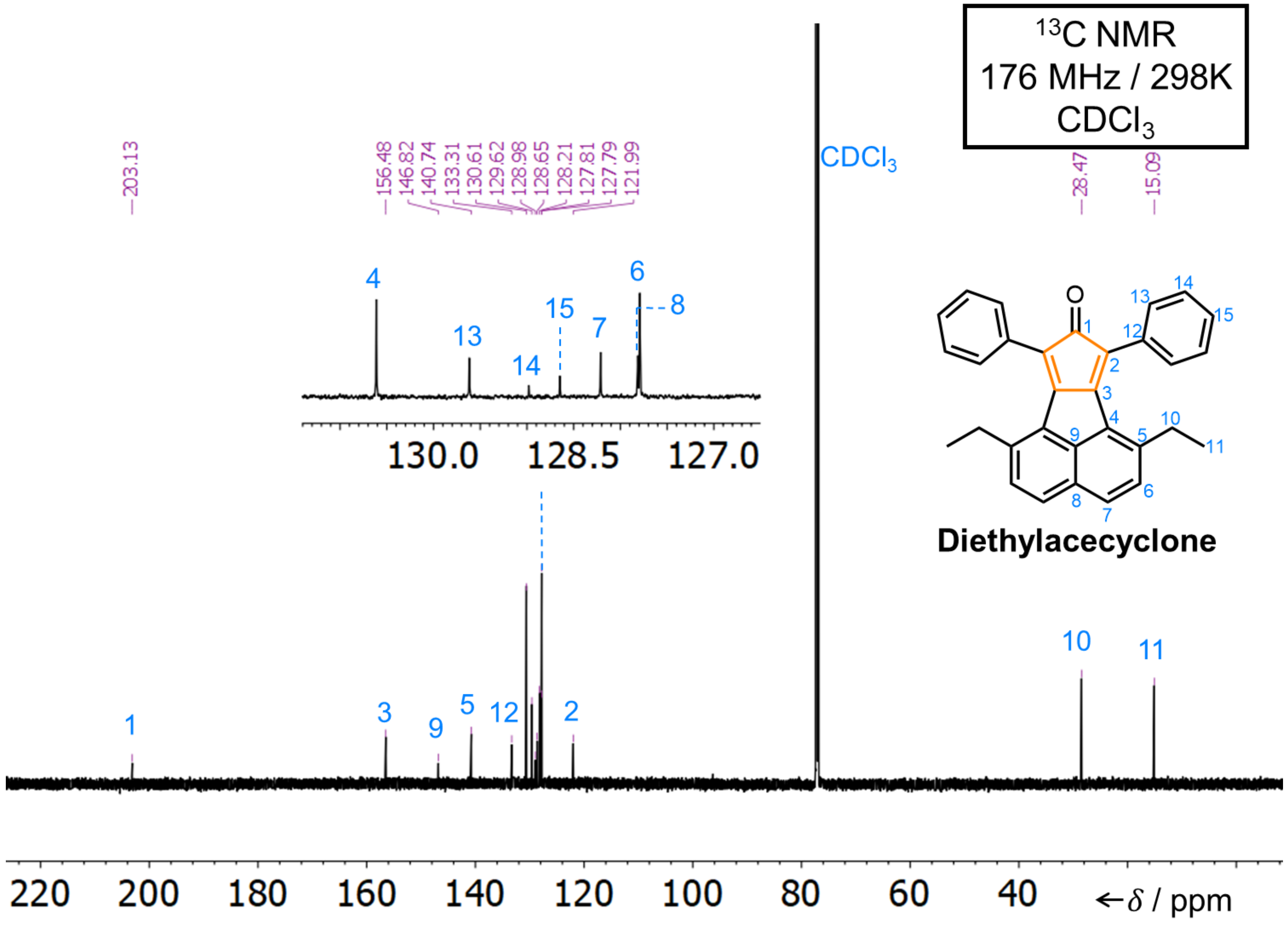

Fig. S11. ^{13}C NMR spectrum of **diethylacecyclone**.

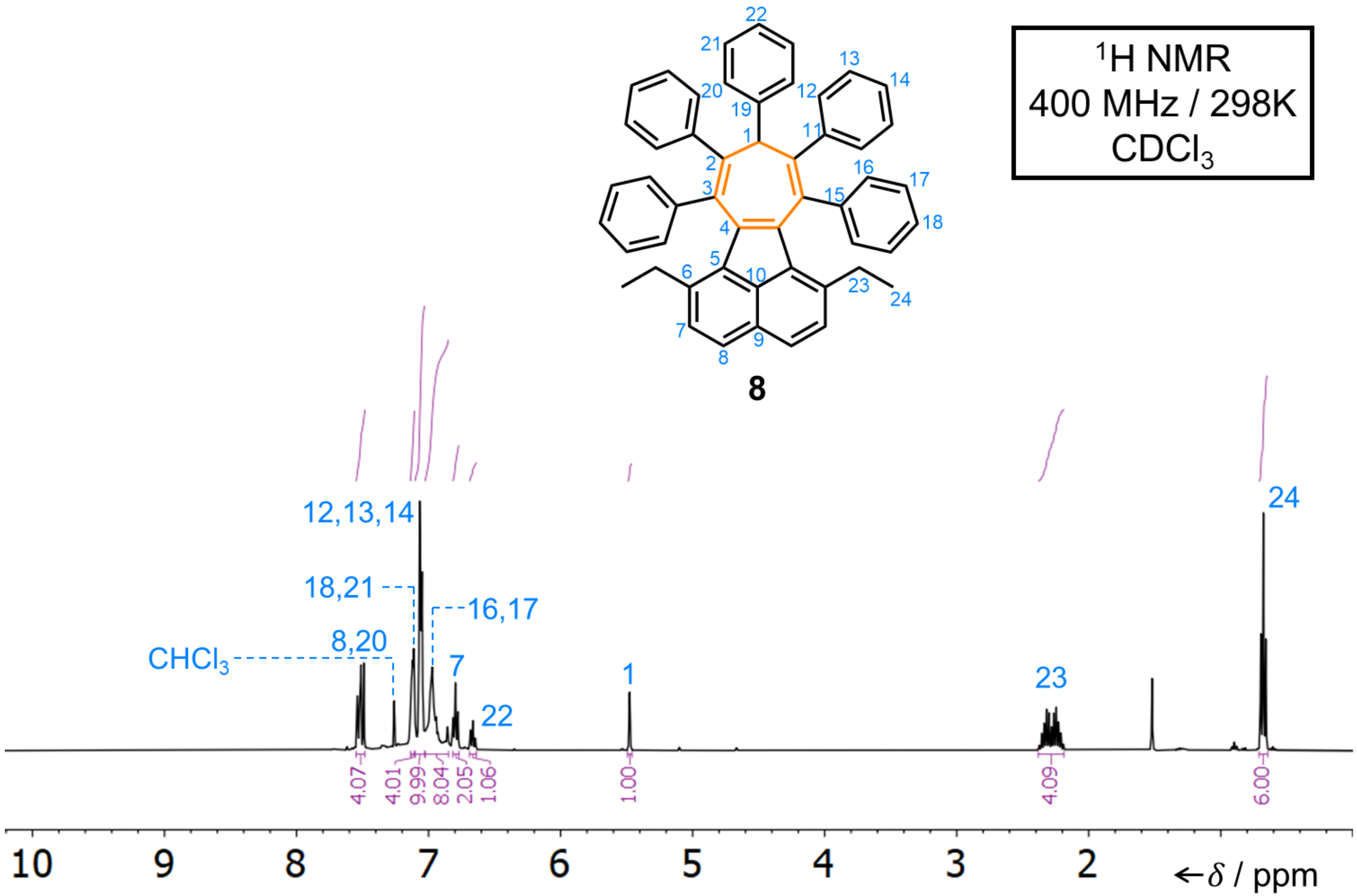

Fig. S12. ^{1}H NMR spectrum of **8**.

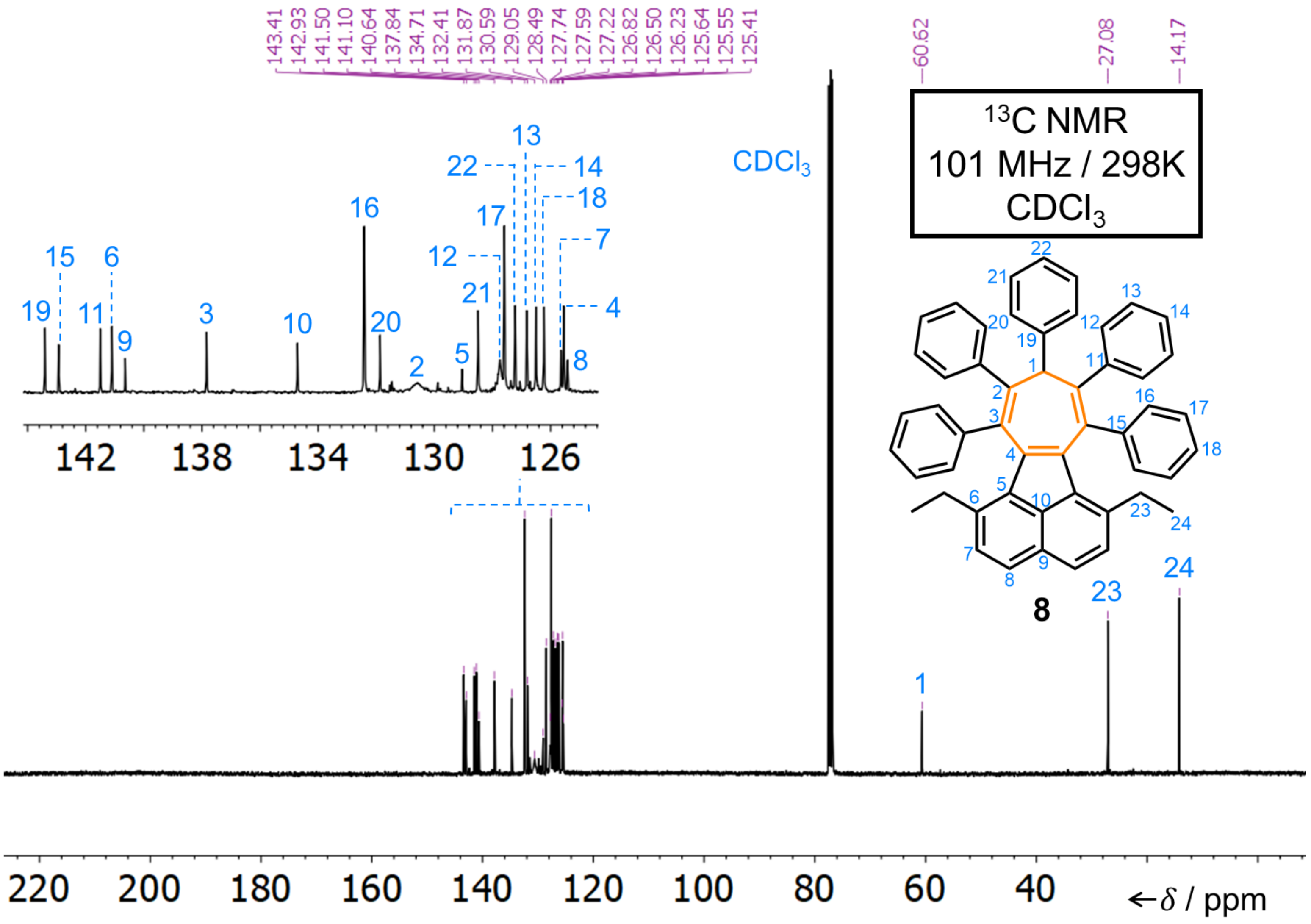

Fig. S13. ^{13}C NMR spectrum of **8**.

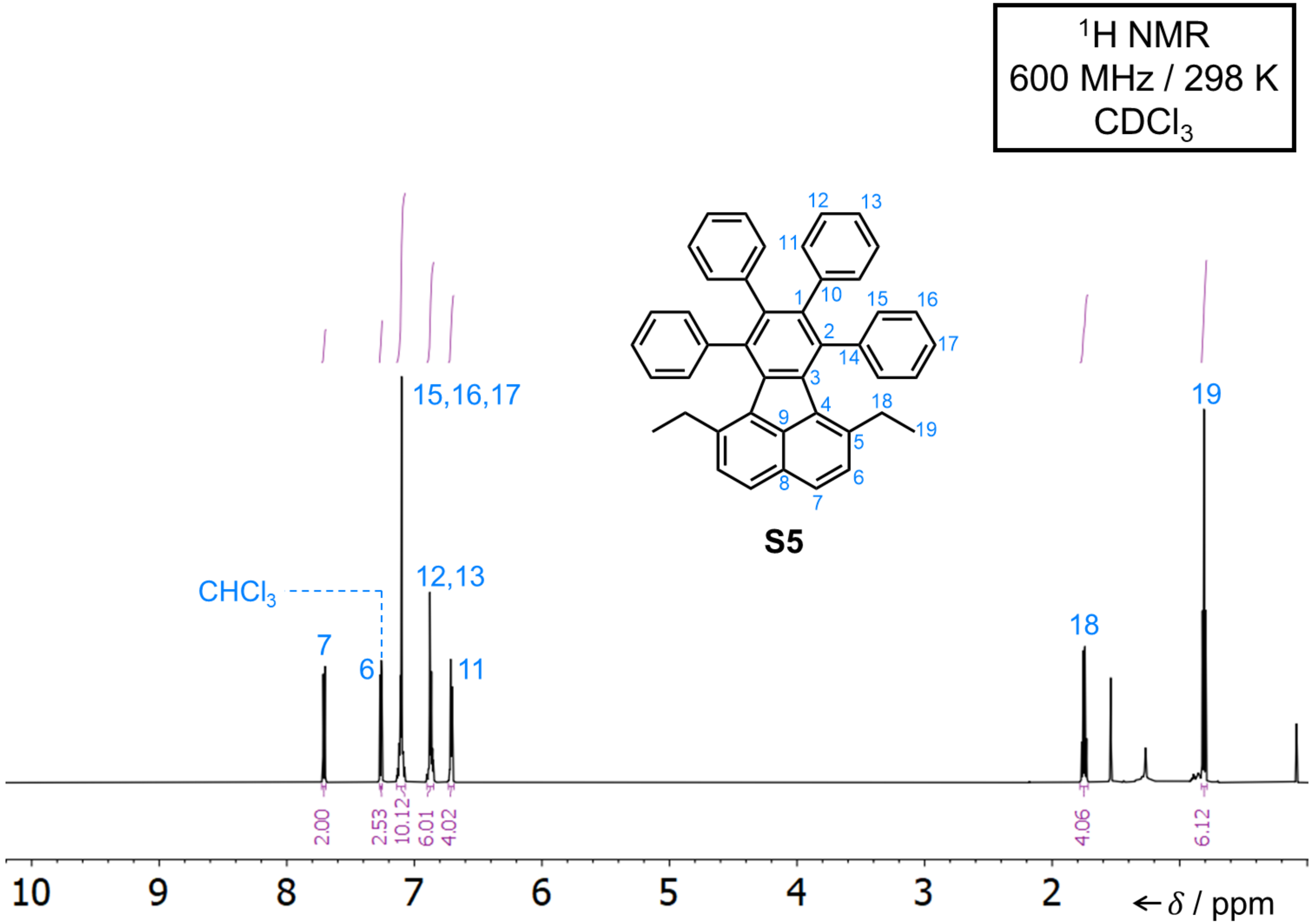

Fig. S14. ^{1}H NMR spectrum of **S5**.

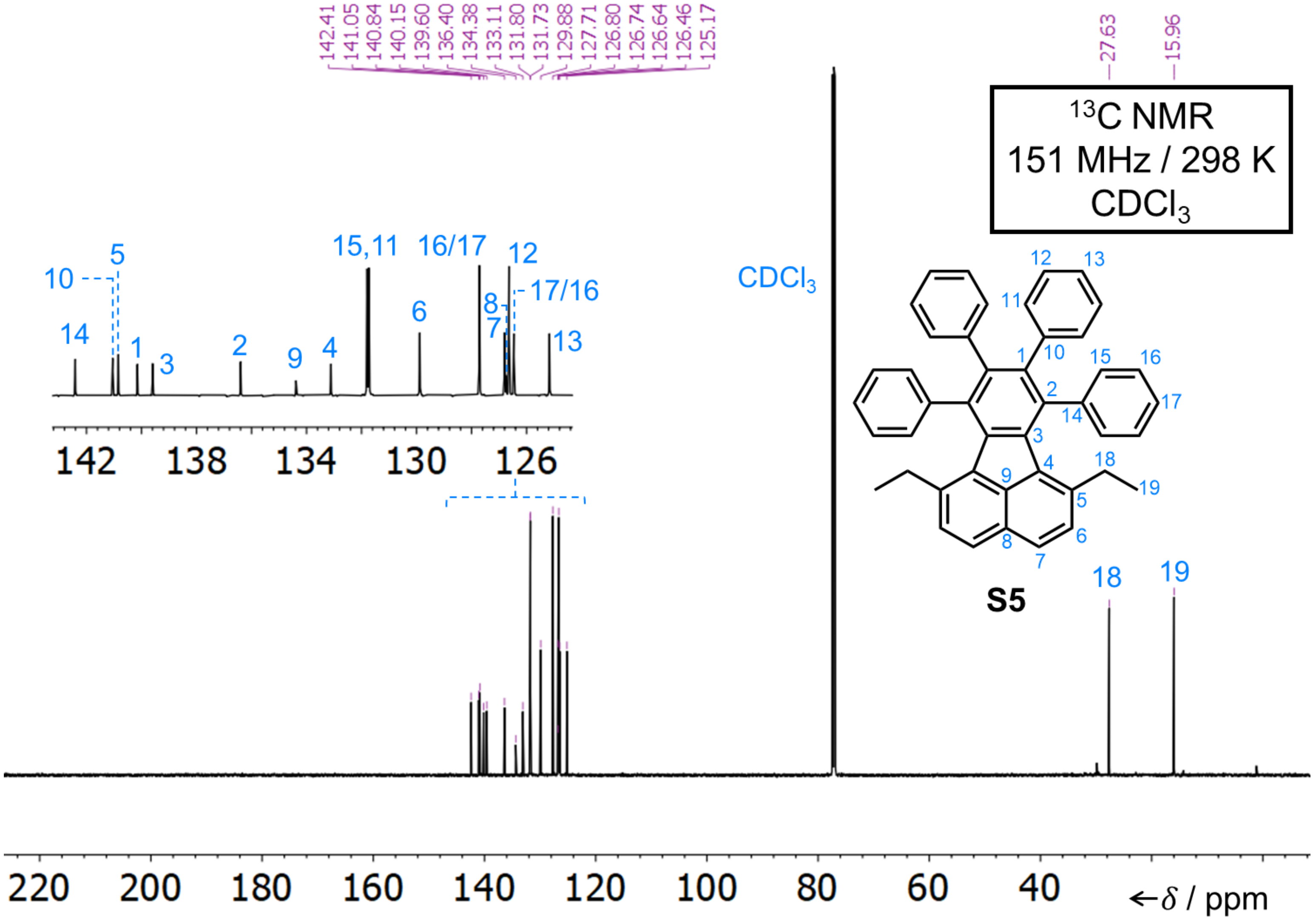

Fig. S15. ^{13}C NMR spectrum of **S5**.

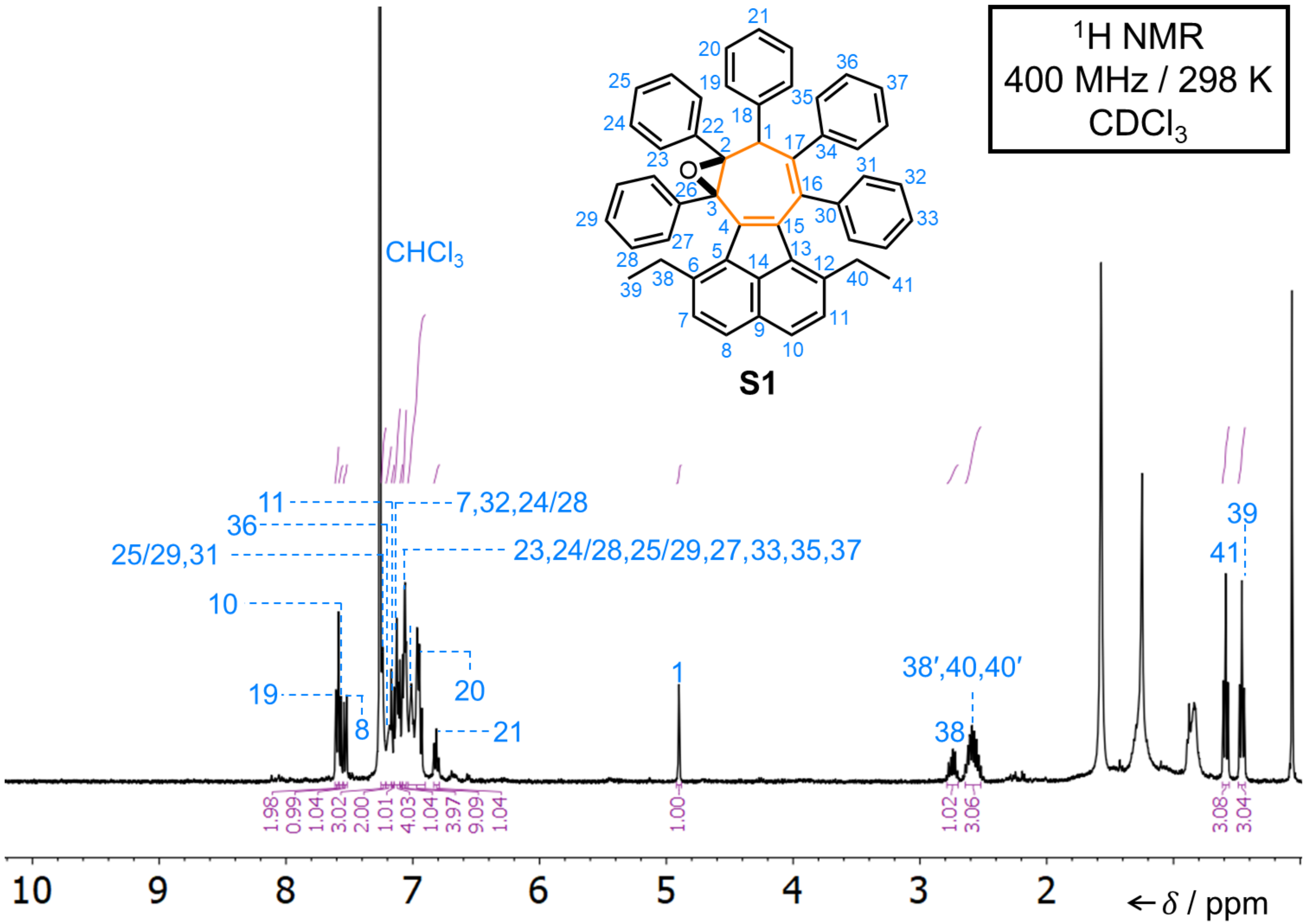

Fig. S16. ^{1}H NMR spectrum of **S1**.

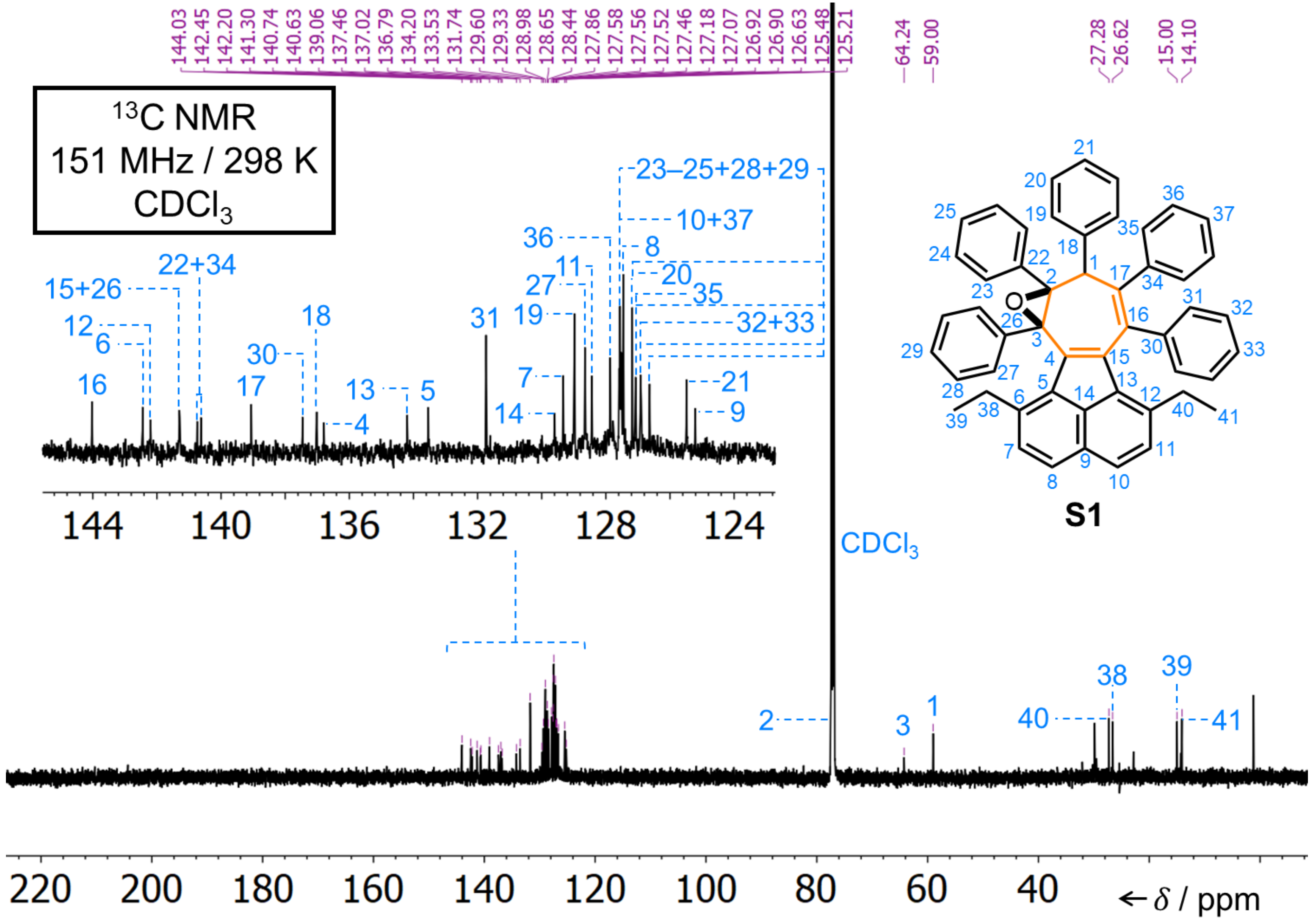

Fig. S17. ^{13}C NMR spectrum of **S1**.

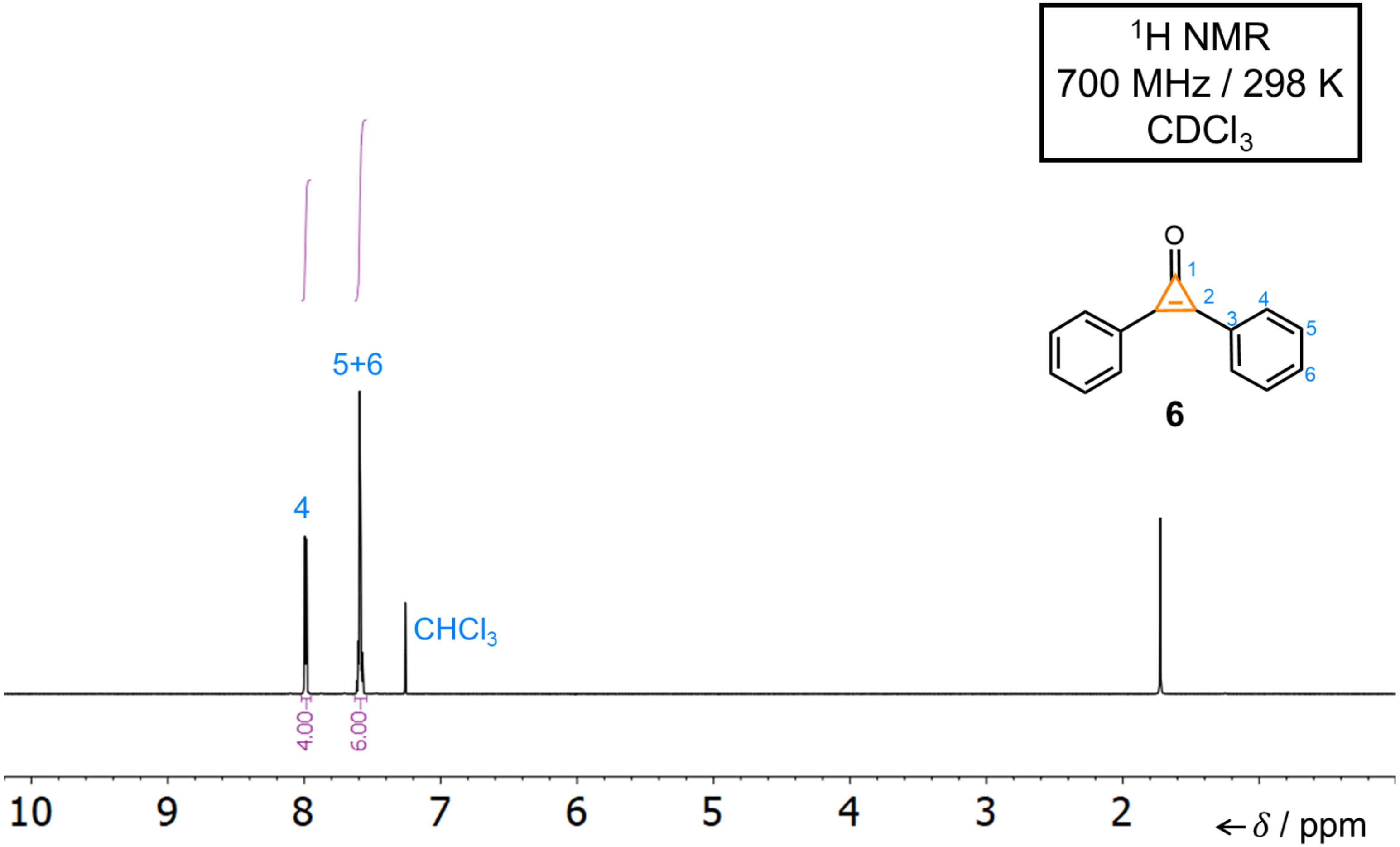

Fig. S18. ^{1}H NMR spectrum of **6**.

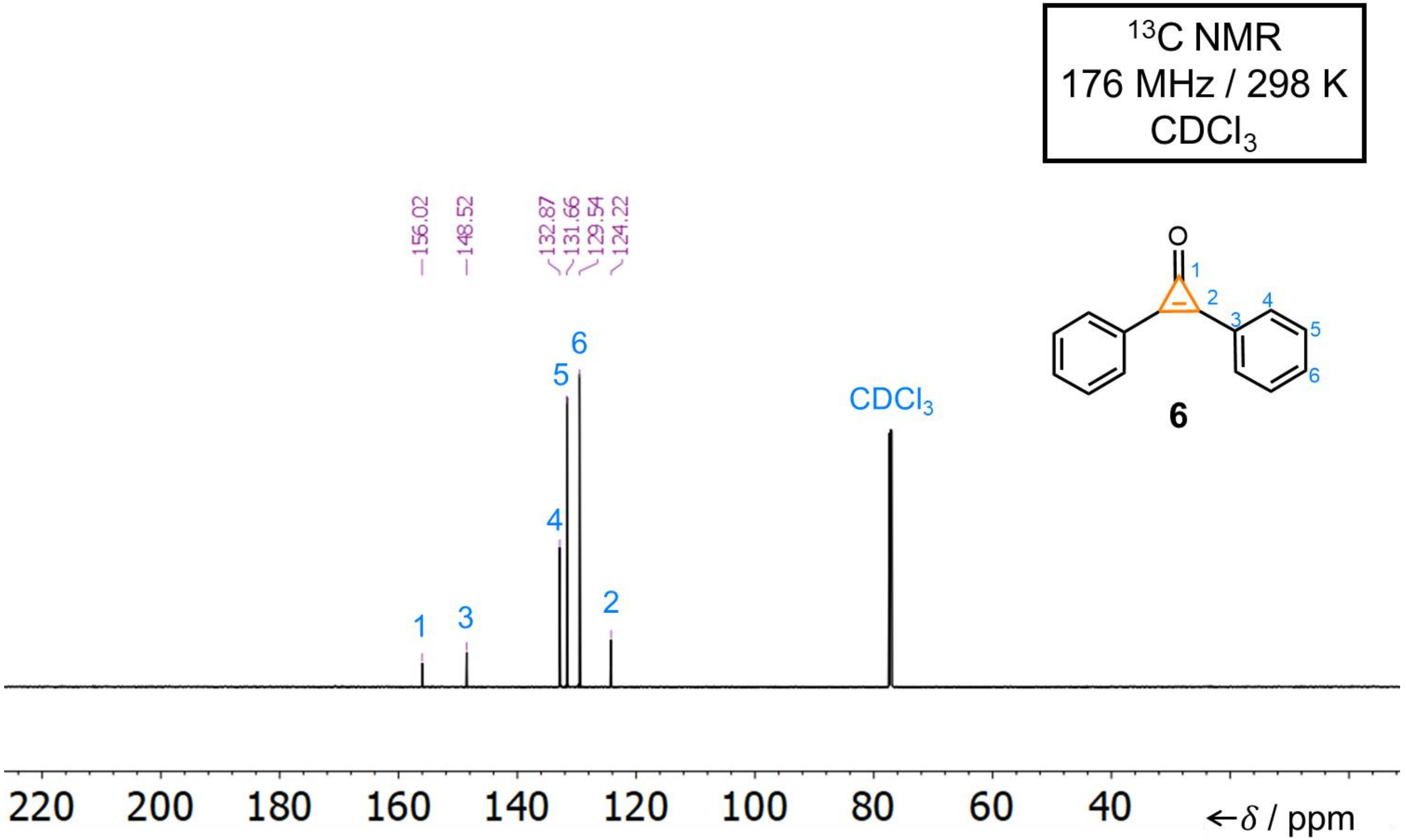

Fig. S19. ^{13}C NMR spectrum of **6**.

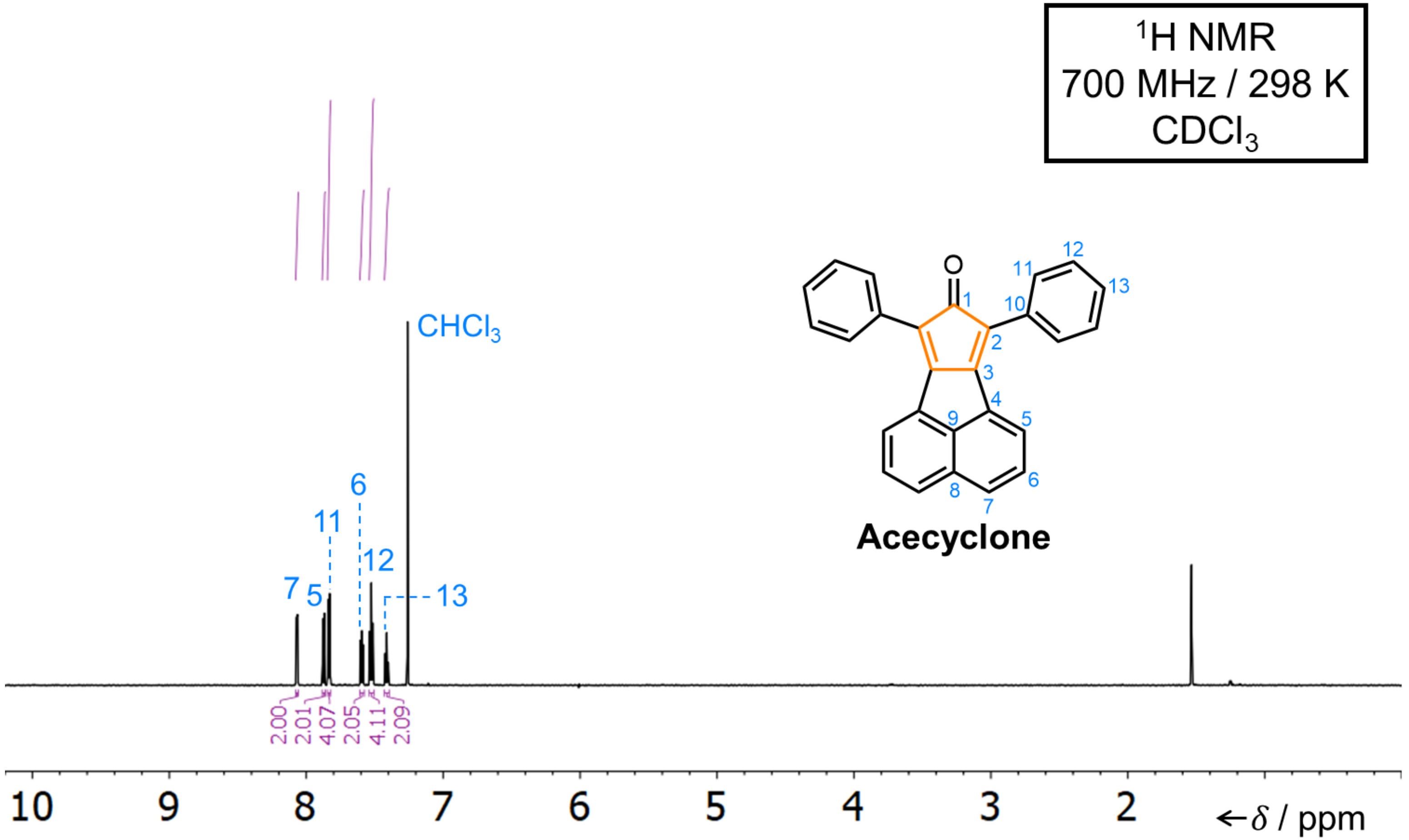

Fig. S20. ^{1}H NMR spectrum of **acecyclone**.

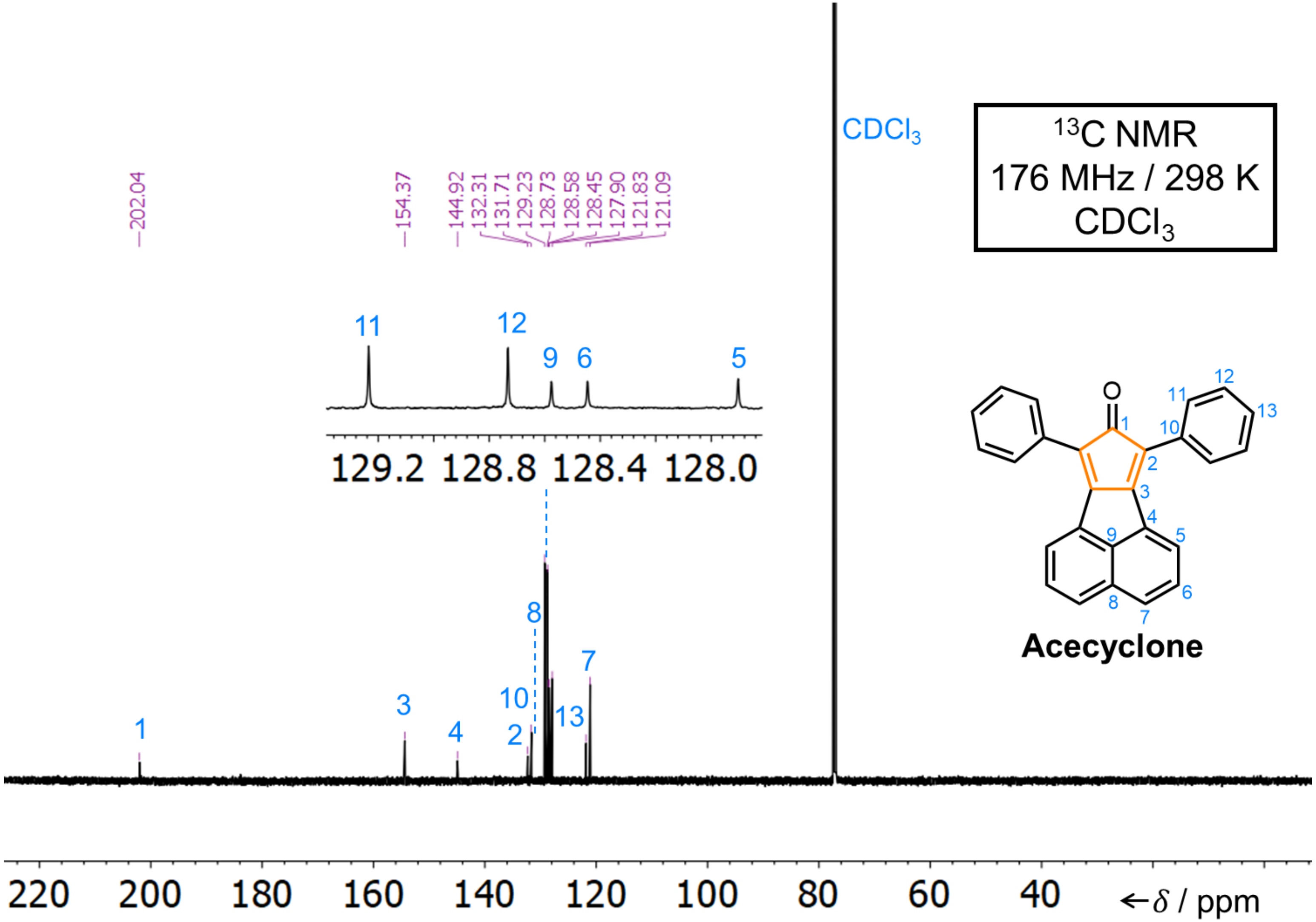

Fig. S21. ^{13}C NMR spectrum of **acecyclone**.

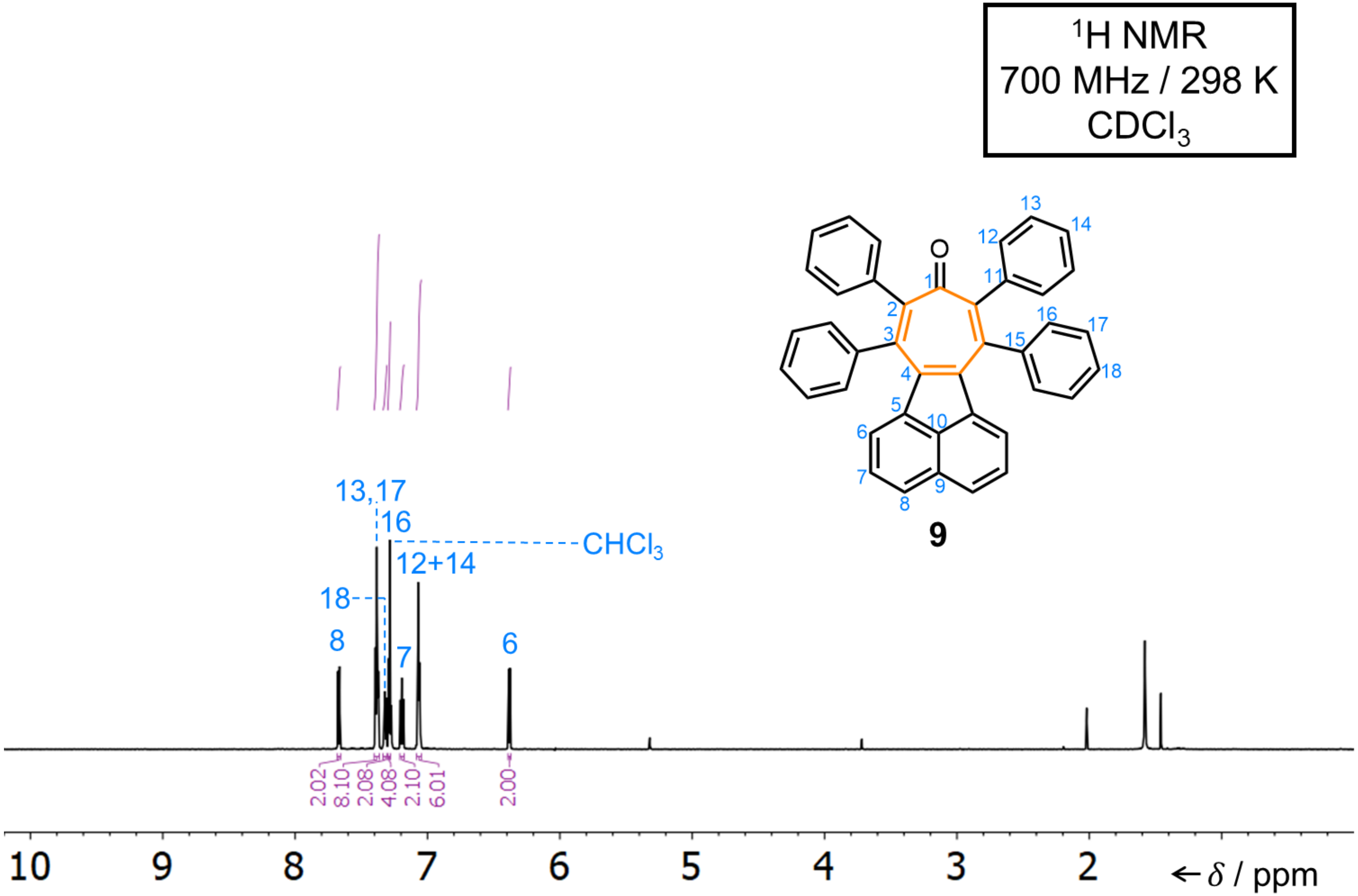

Fig. S22. ^{1}H NMR spectrum of **9**.

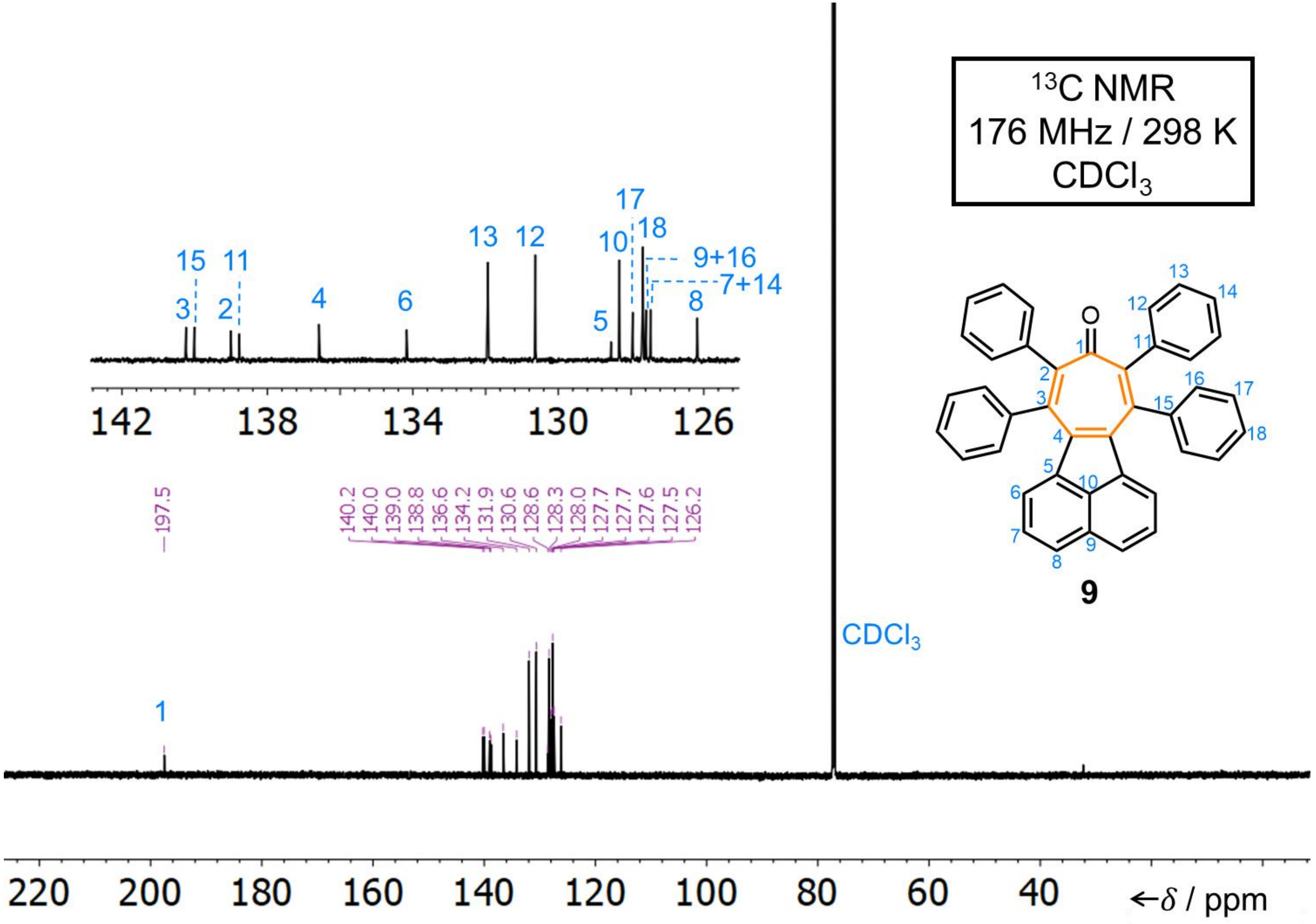

Fig. S23. ^{13}C NMR spectrum of **9**.

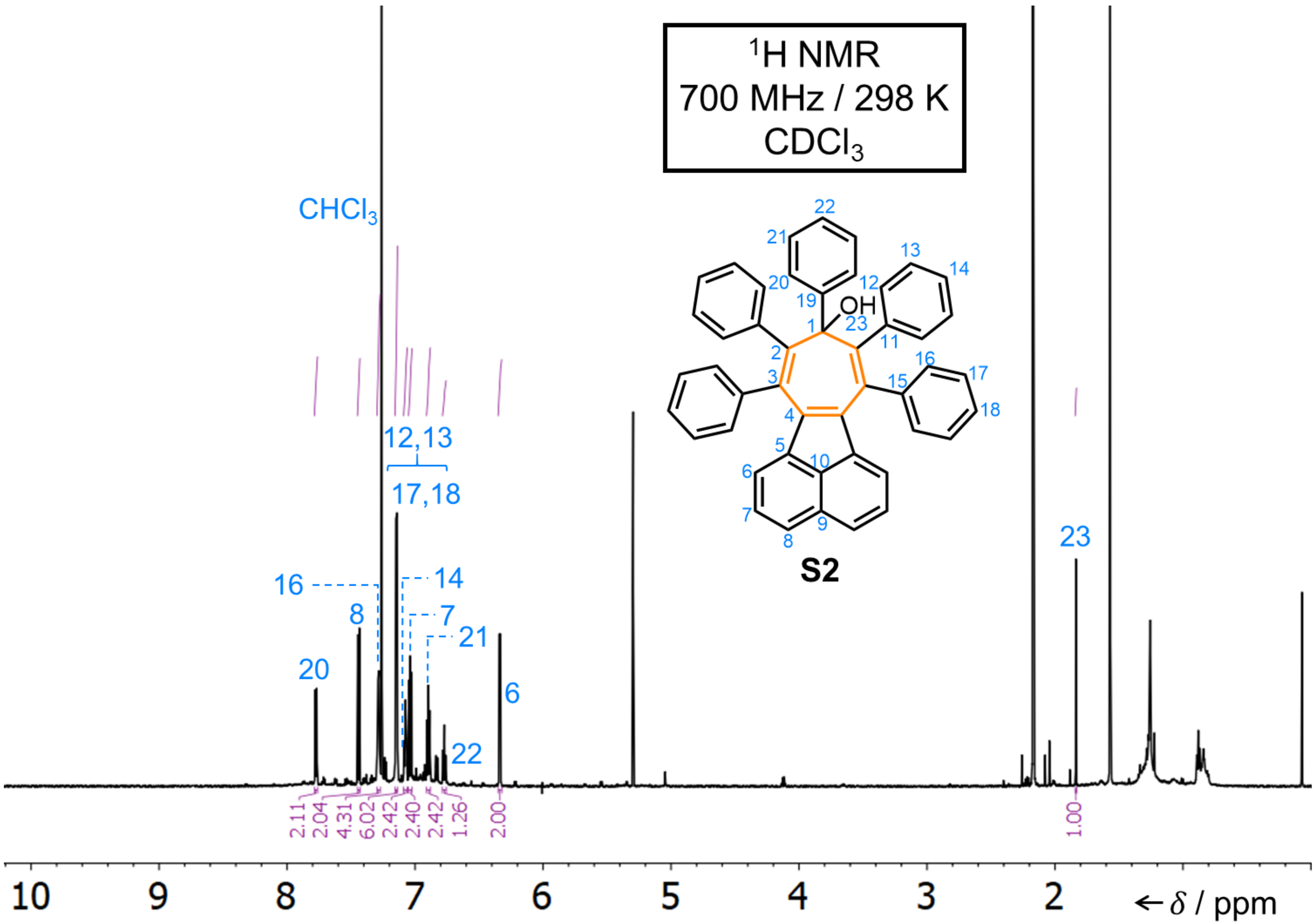

Fig. S24. ^{1}H NMR spectrum of **S2**.

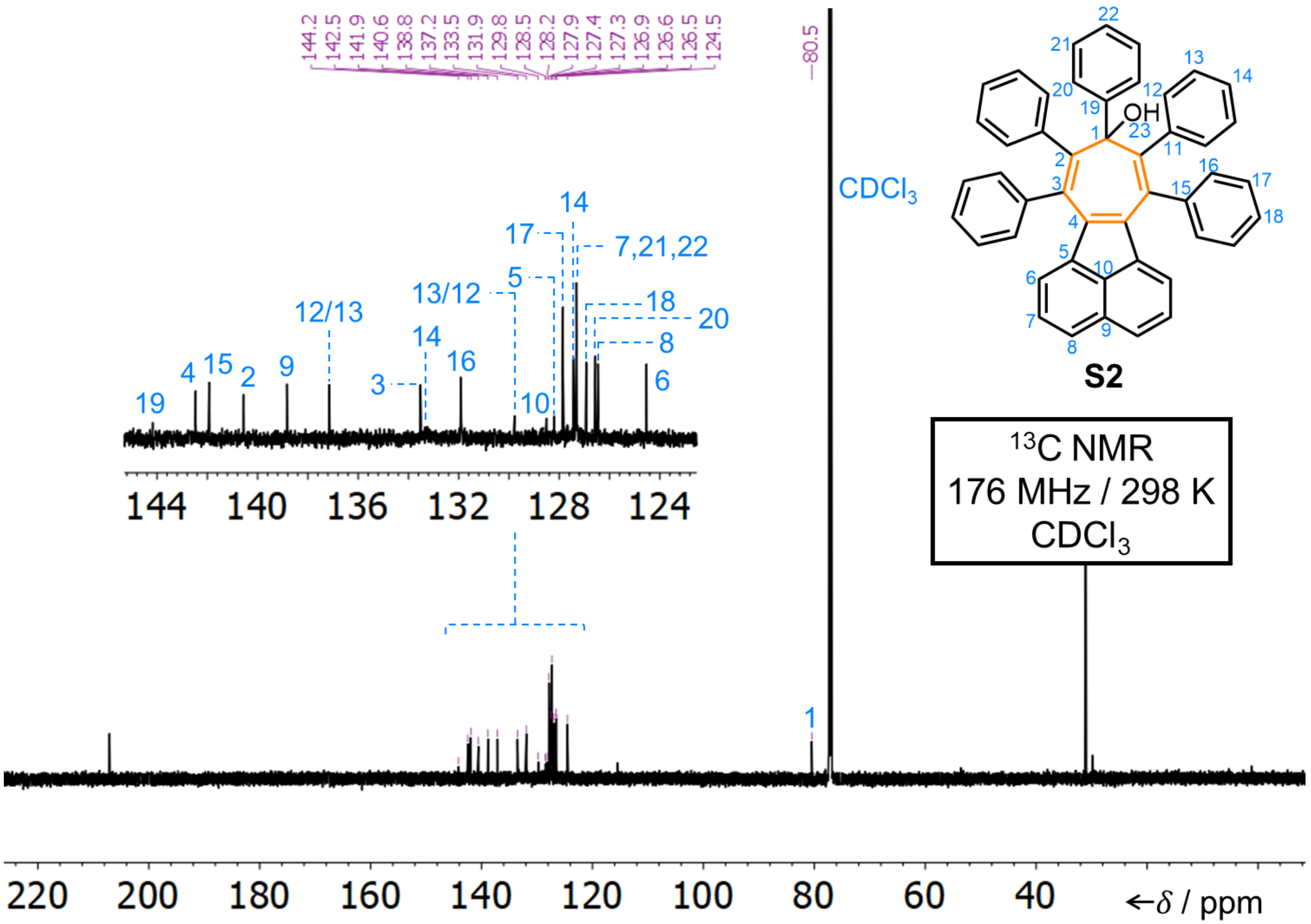

Fig. S25. ^{13}C NMR spectrum of **S2**.

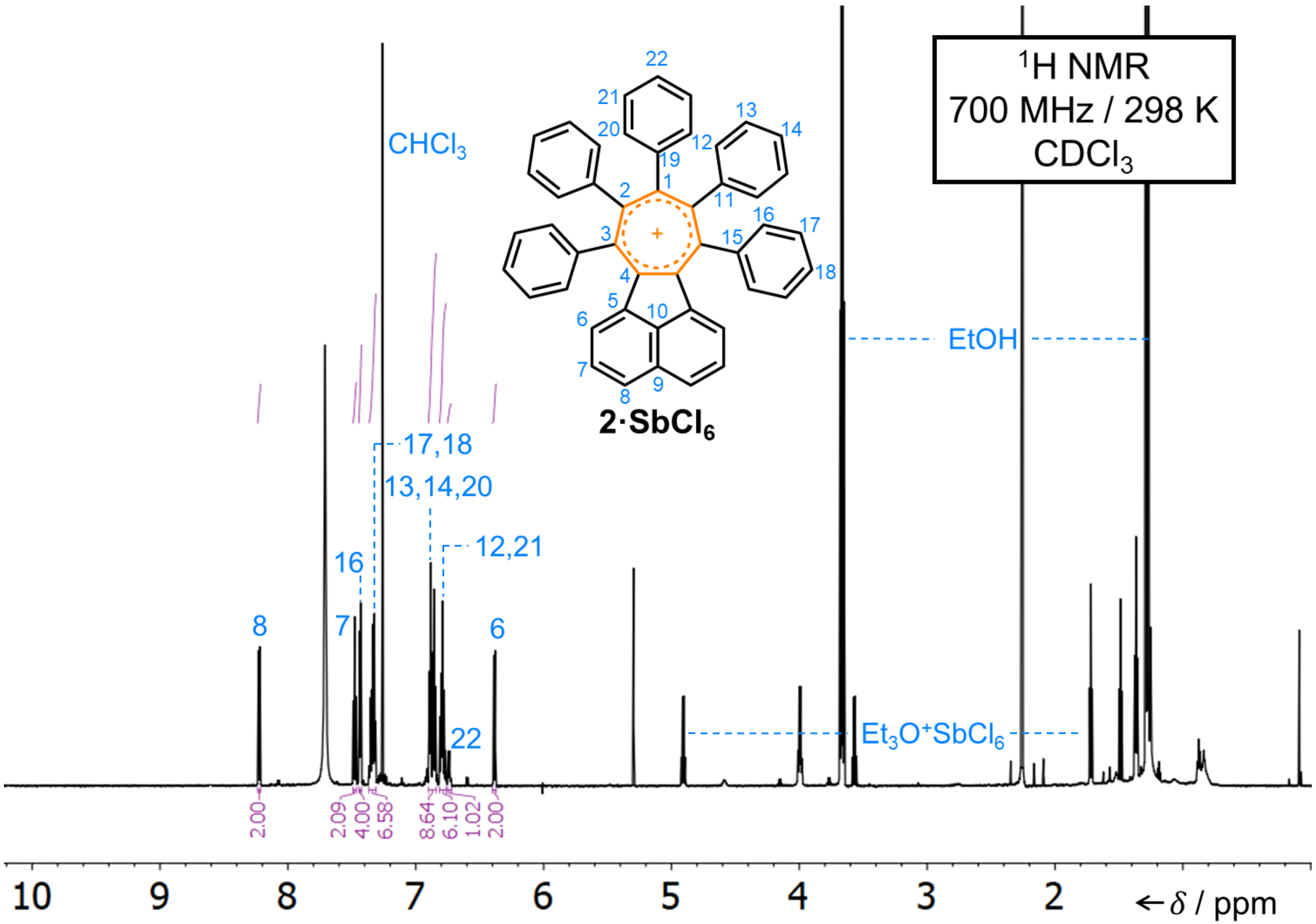

Fig. S26. ^{1}H NMR spectrum of **2**·SbCl$_6$.

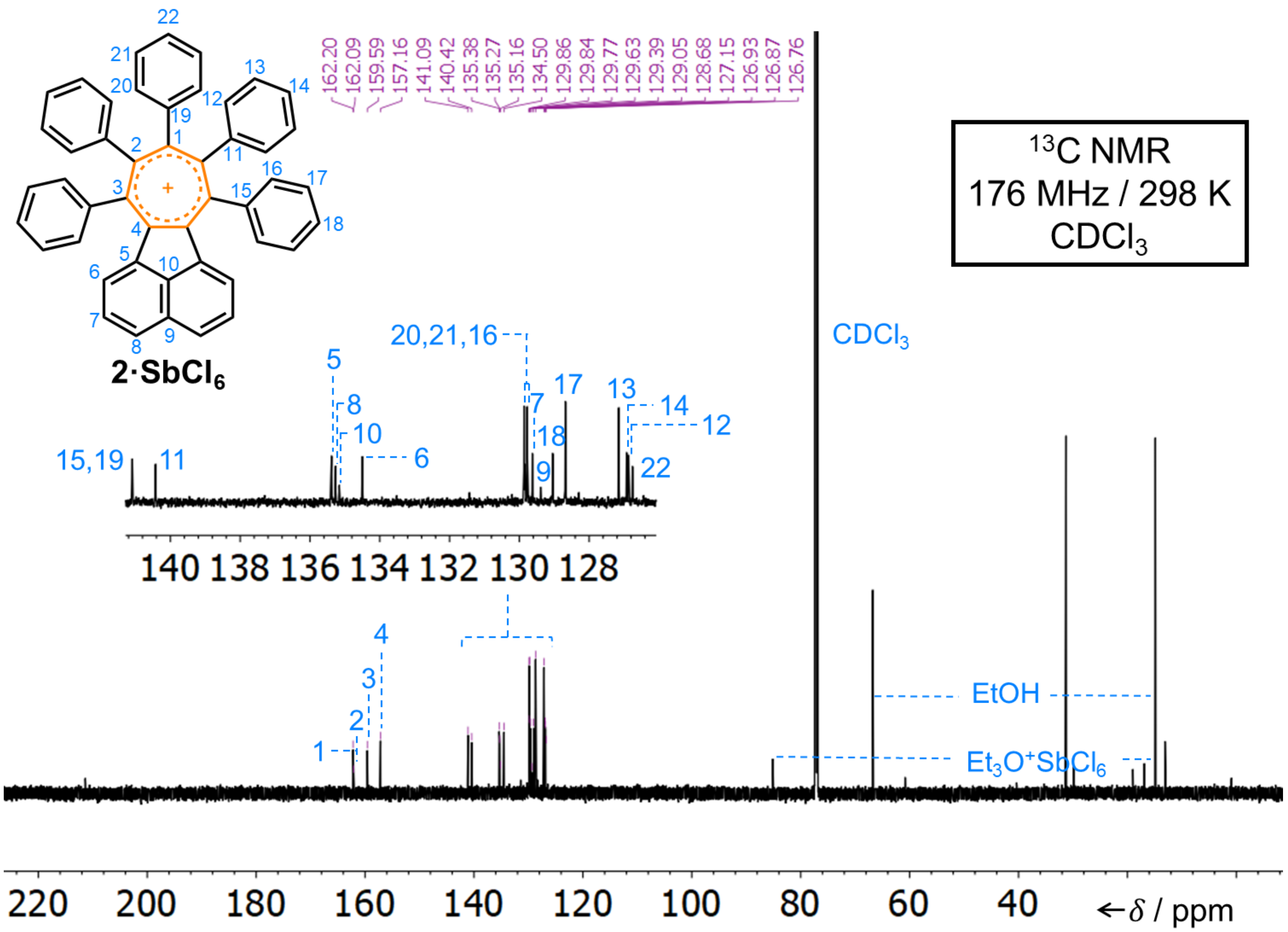

Fig. S27. ^{13}C NMR spectrum of **2**·SbCl$_6$.

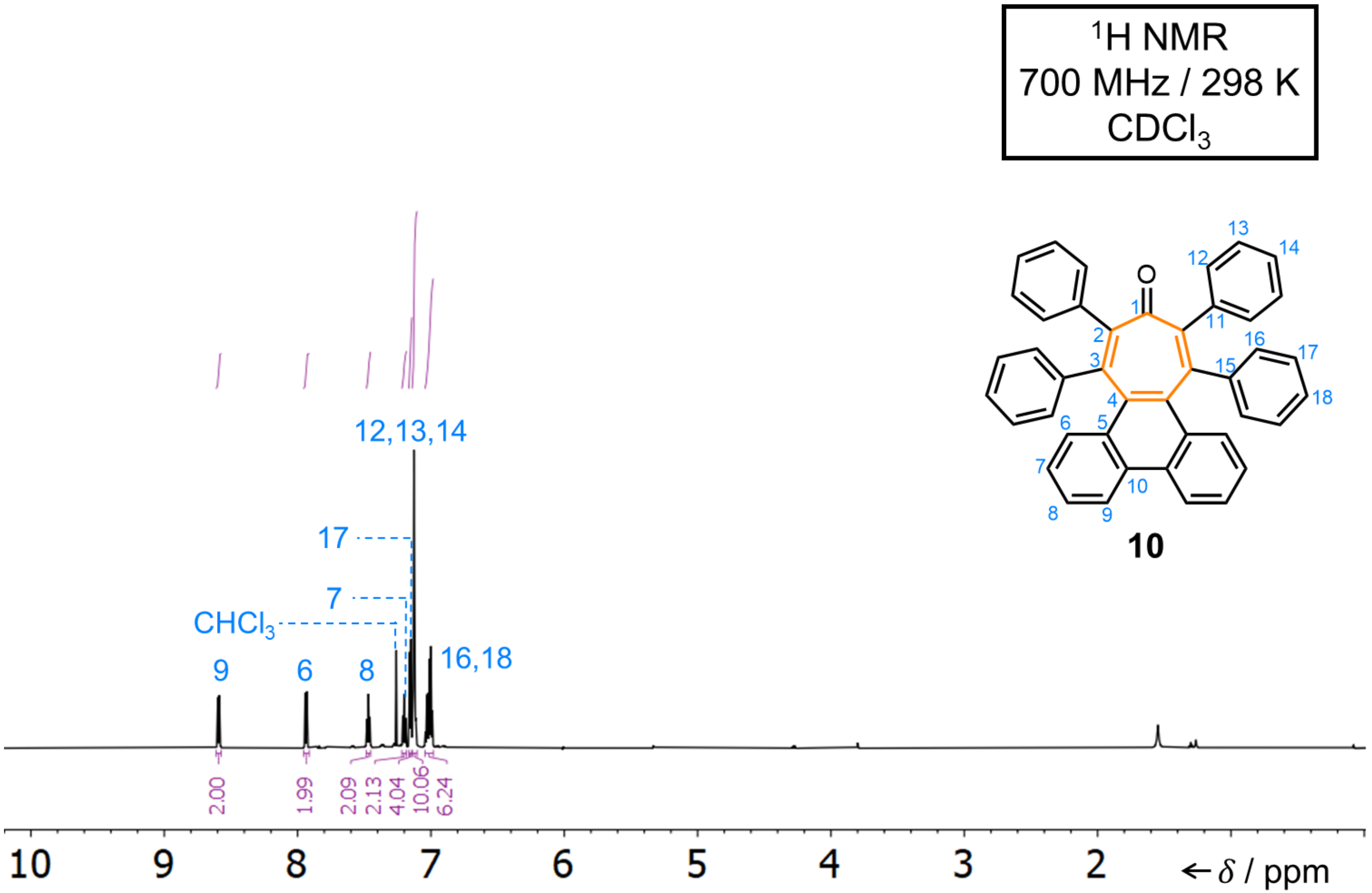

Fig. S28. ^{1}H NMR spectrum of **10**.

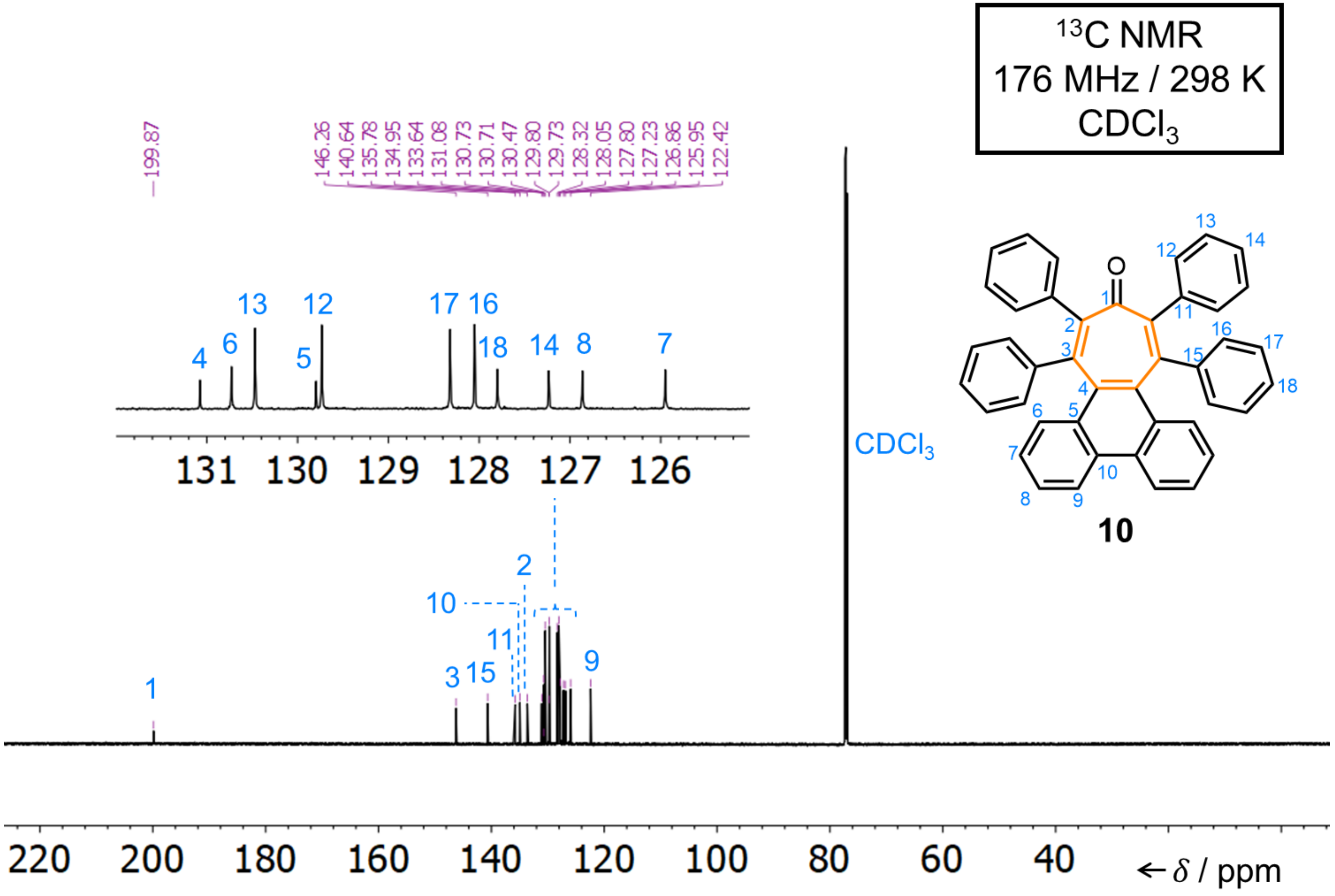

Fig. S29. ^{13}C NMR spectrum of **10**.

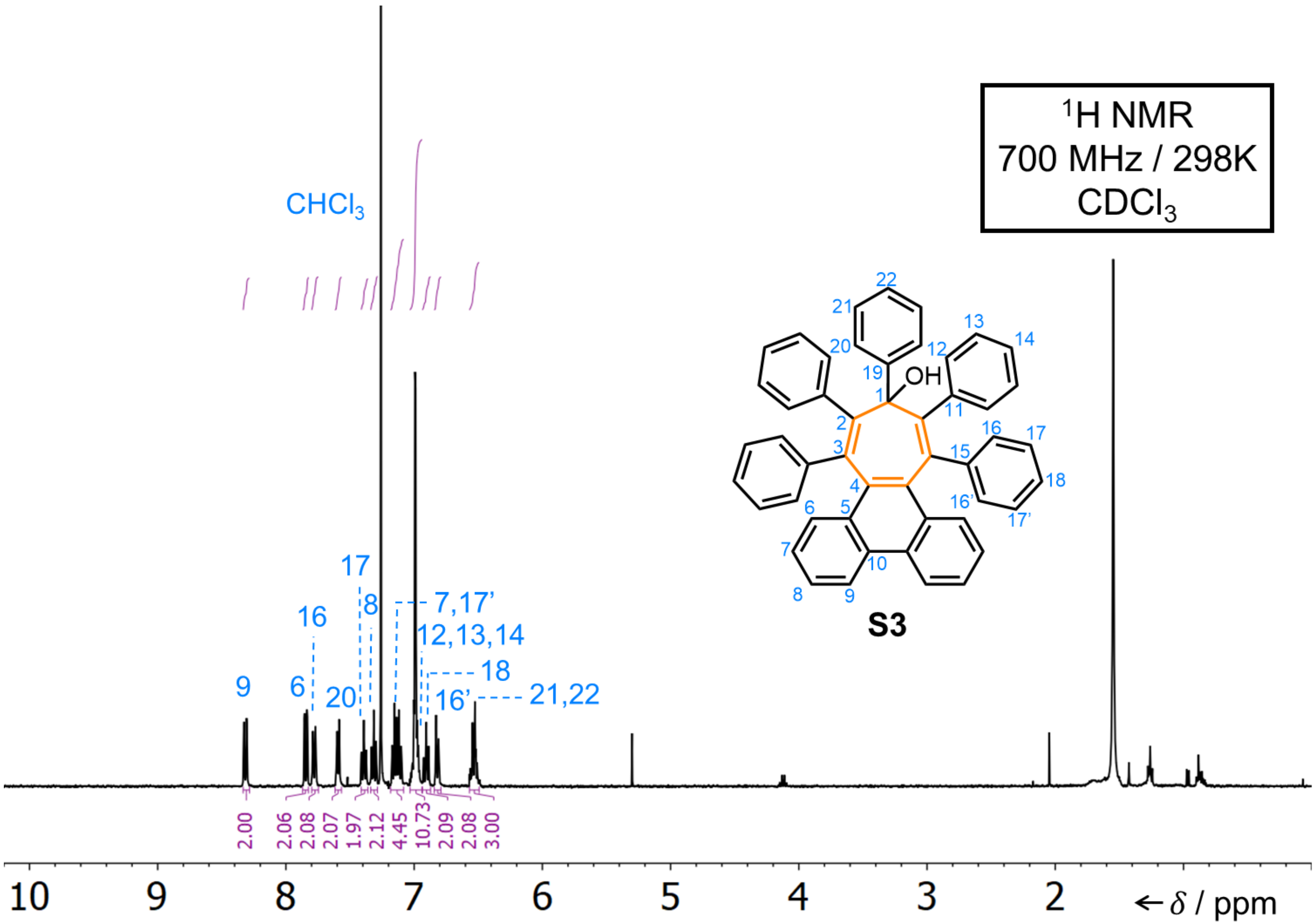

Fig. S30. ^{1}H NMR spectrum of **S3**.

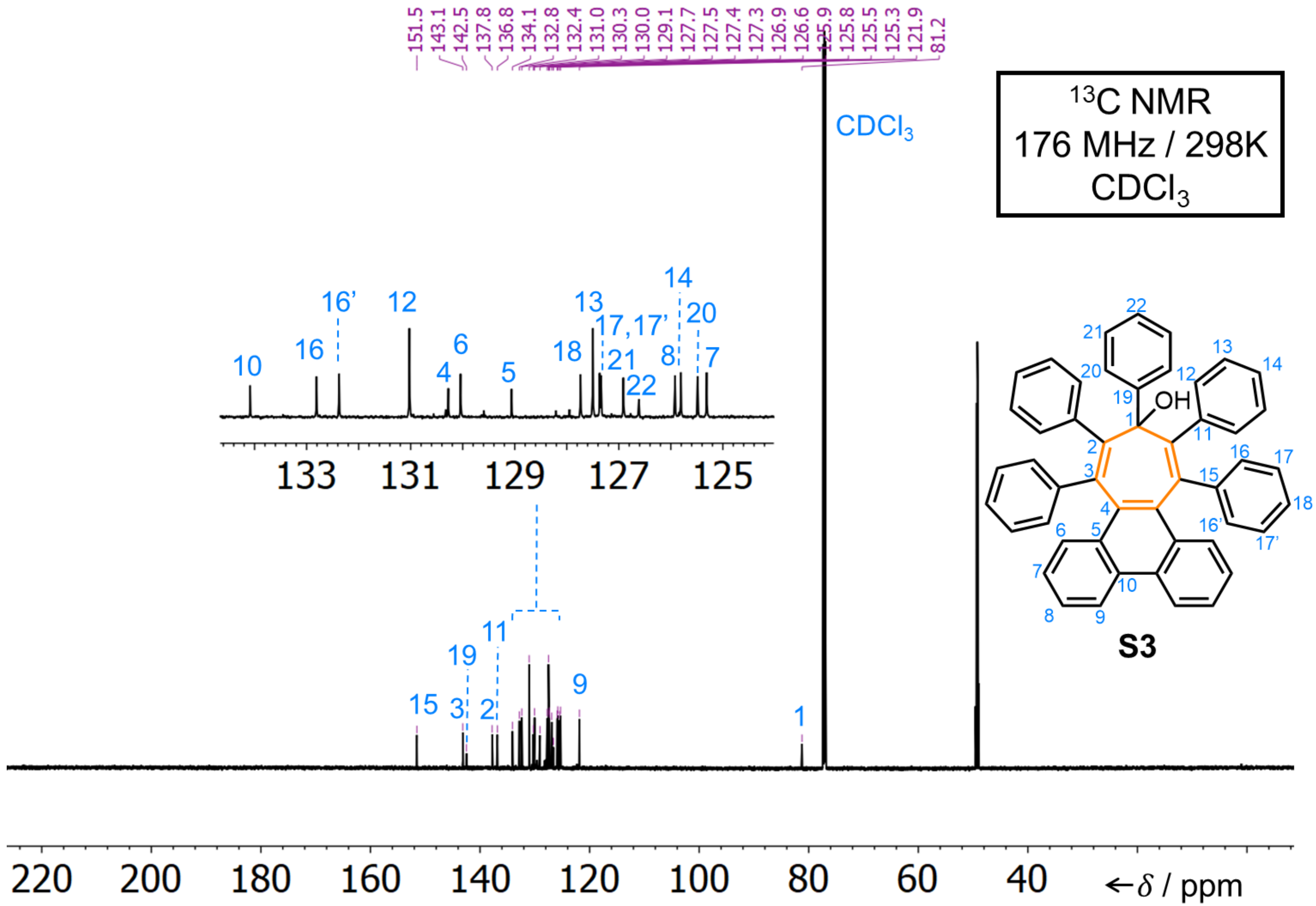

Fig. S31. ^{13}C NMR spectrum of **S3**.

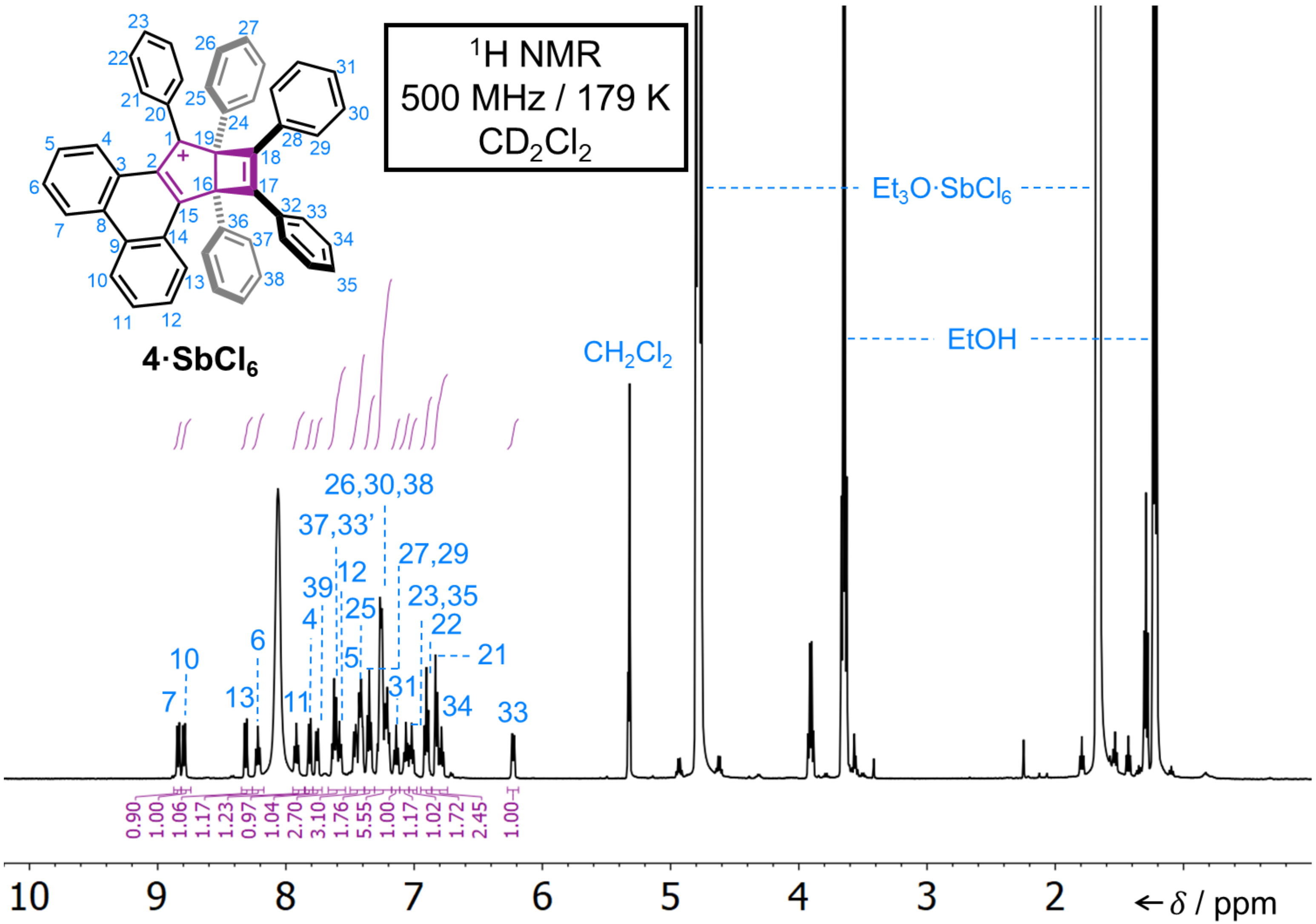

Fig. S32. ^{1}H NMR spectrum of **4**·$SbCl_6$.

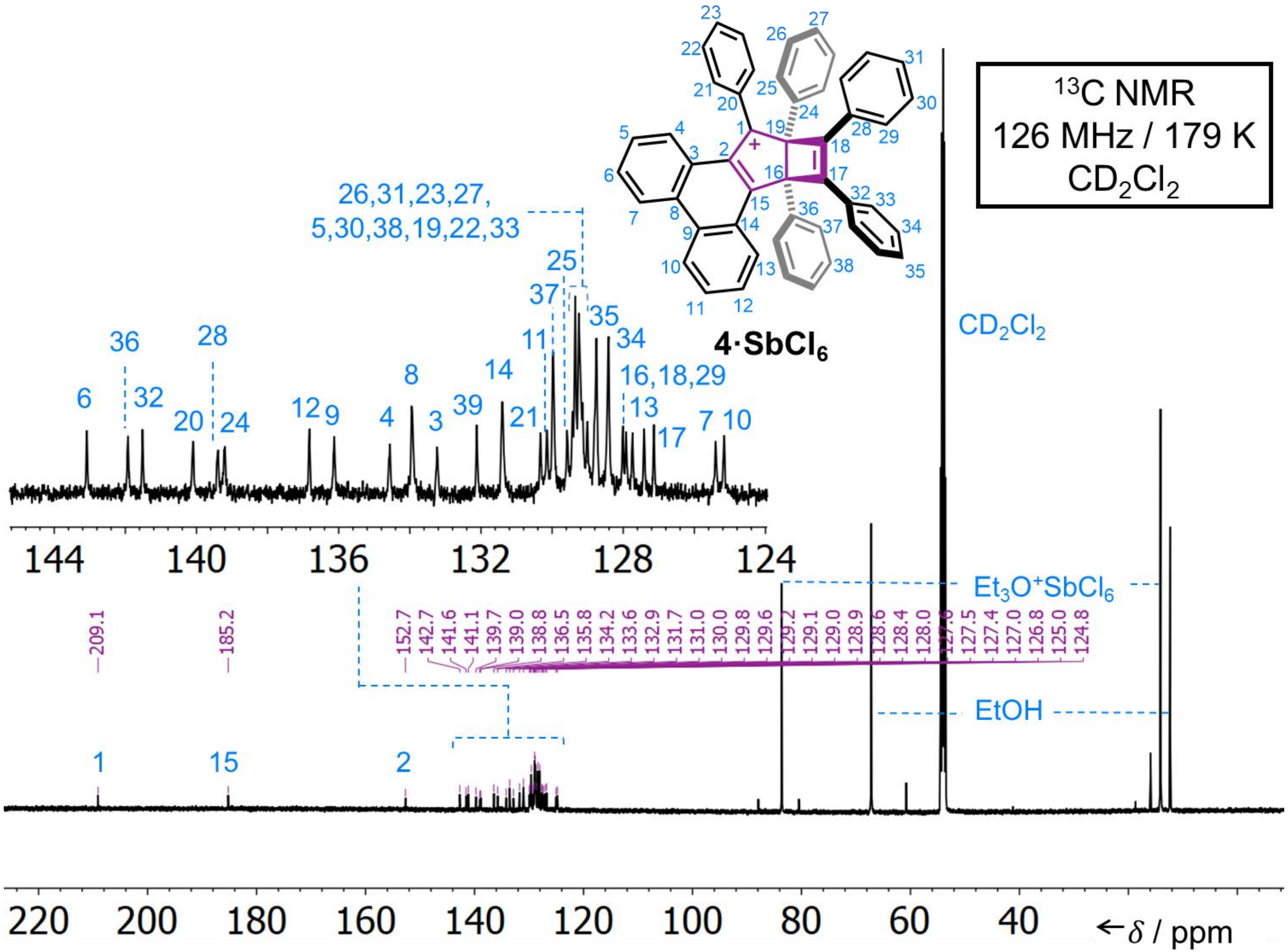

Fig. S33. ^{13}C NMR spectrum of **4**·SbCl$_6$.

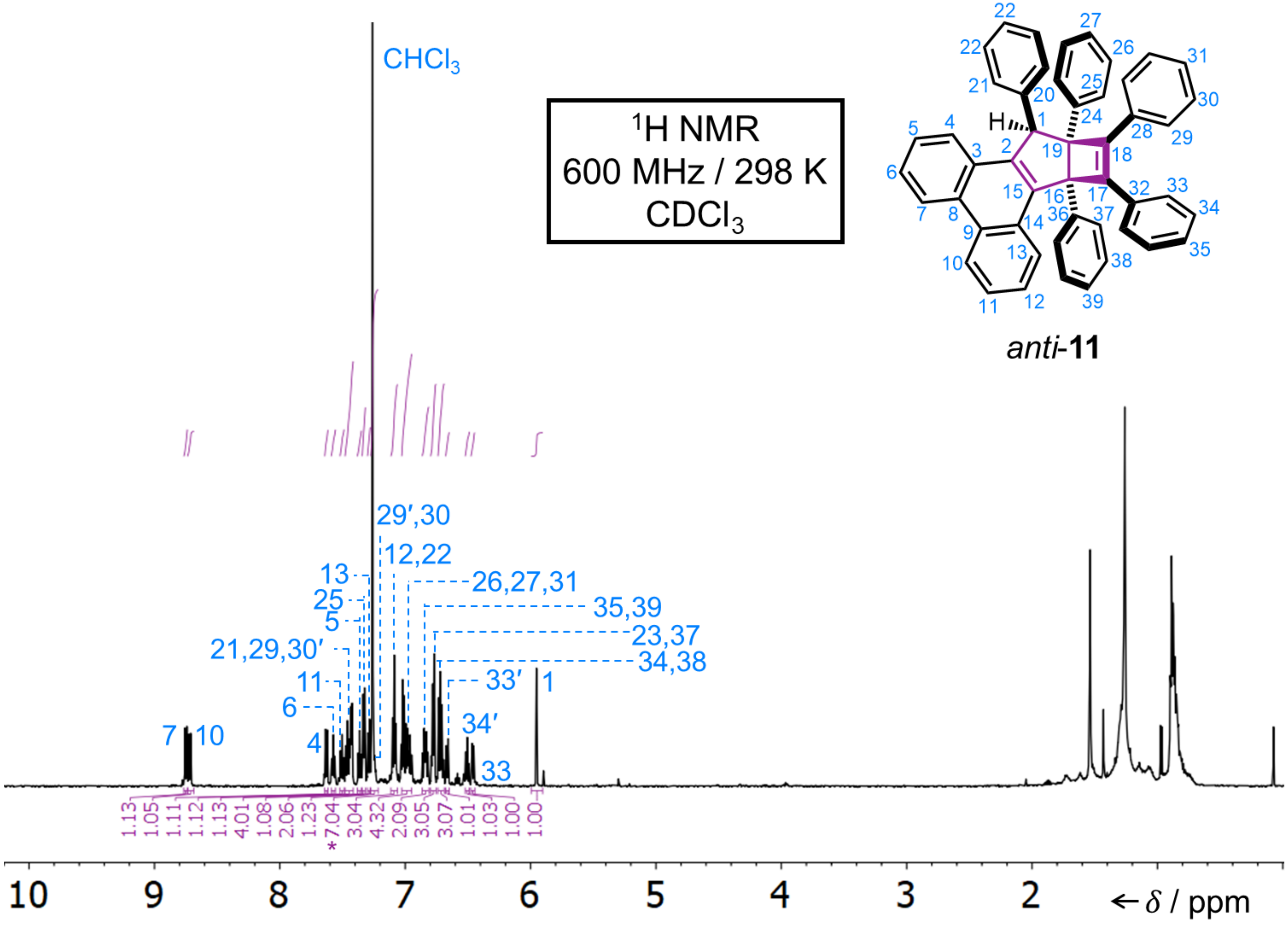

Fig. S34. ^{1}H NMR spectrum of *anti*-**11**. (*Signals overlapping with solvent peak)

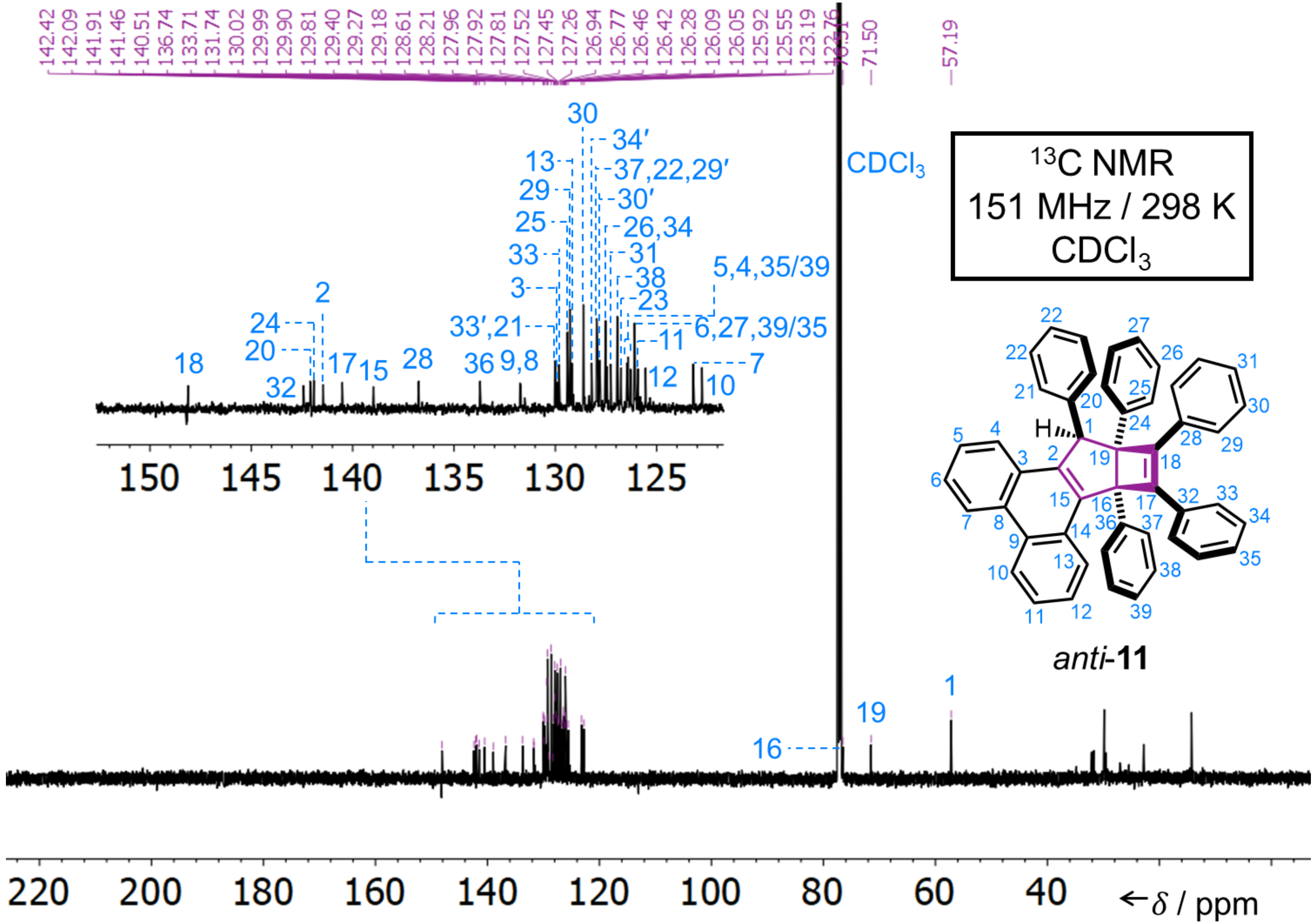

Fig. S35. ^{13}C NMR spectrum of *anti*-**11**.

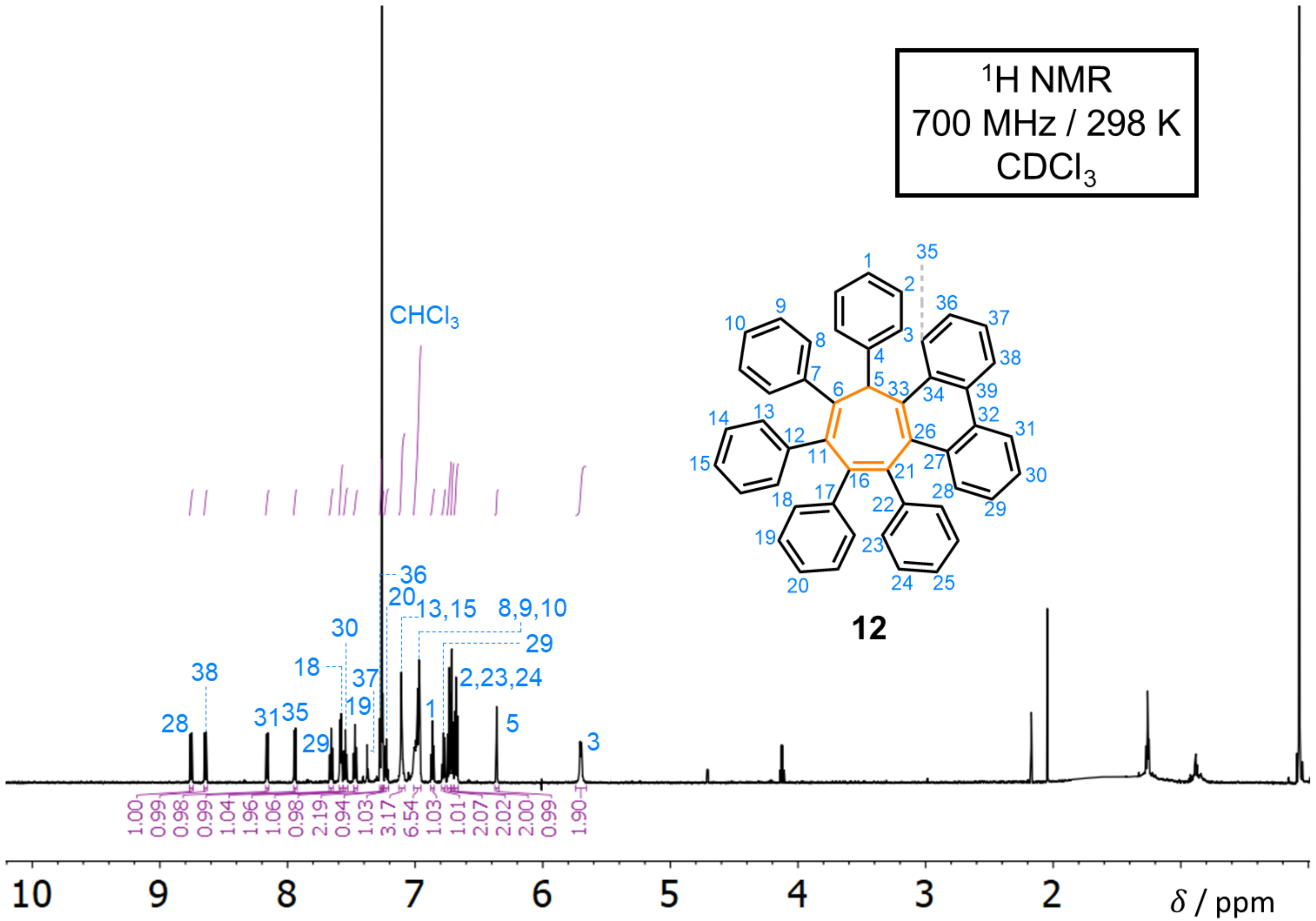

Fig. S36. ¹H NMR spectrum of **12**.

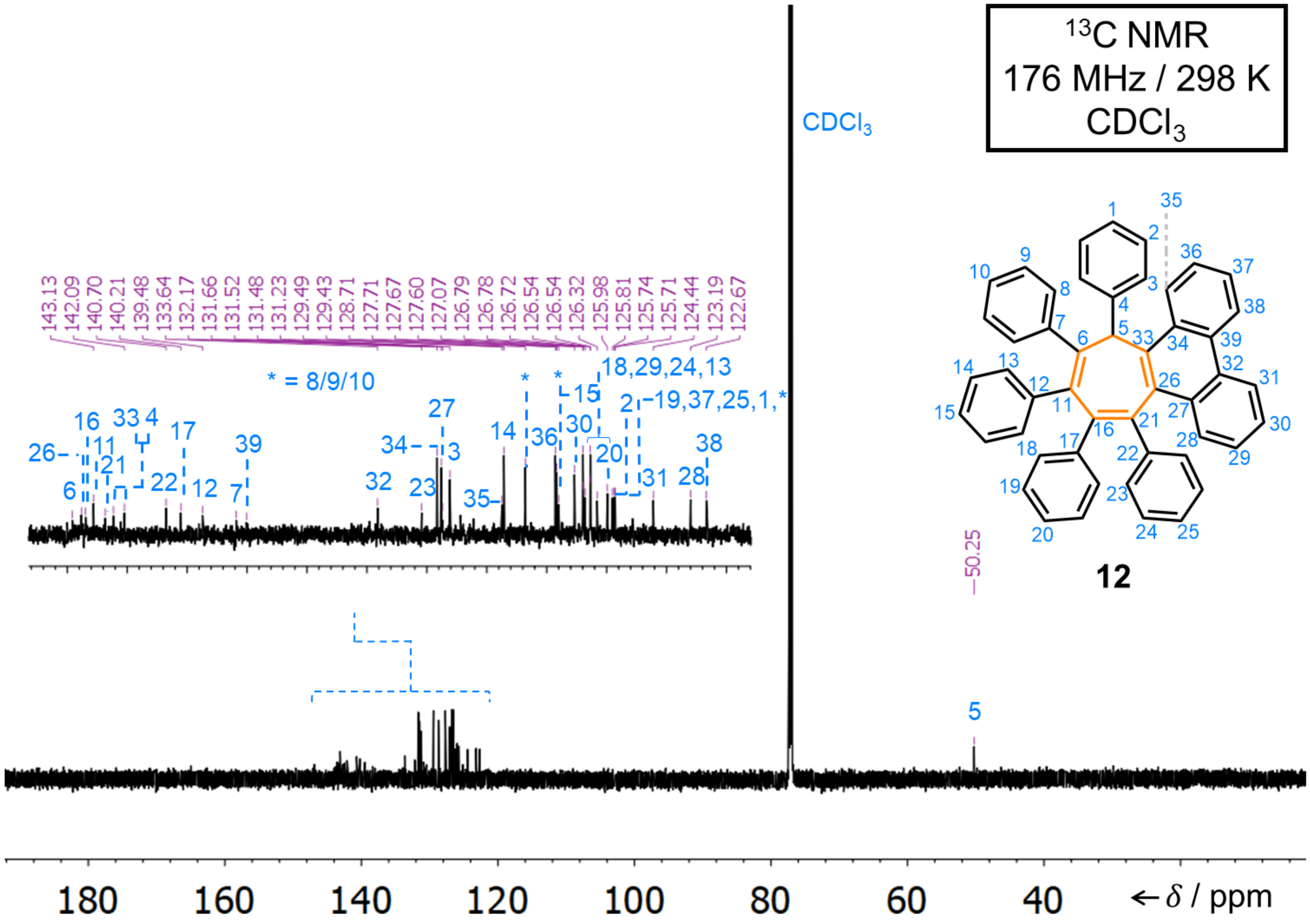

Fig. S37. ^{13}C NMR spectrum of **12**.

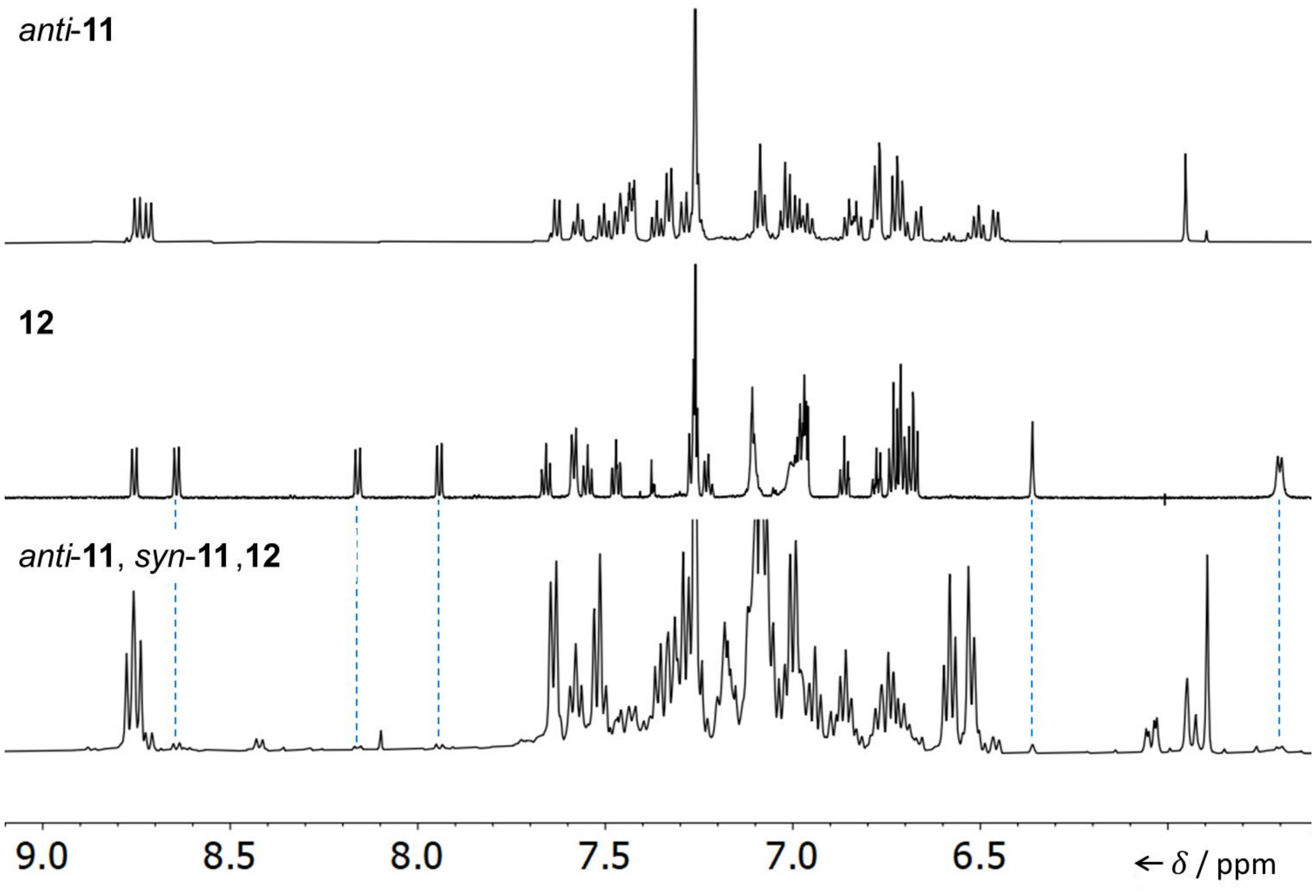

Fig. S38. Comparison of partial ^{1}H NMR spectra stack of (top) *anti*-**11** (600 MHz / 298 K / $CDCl_3$), (b) **12** (700 MHz / 298 K / $CDCl_3$), and (c) the crude mixture of *anti*-**11**, *syn*-**11**, and **12** (500 MHz / 298 K / $CDCl_3$) obtained from the $NaBH_4$ trapping experiment.

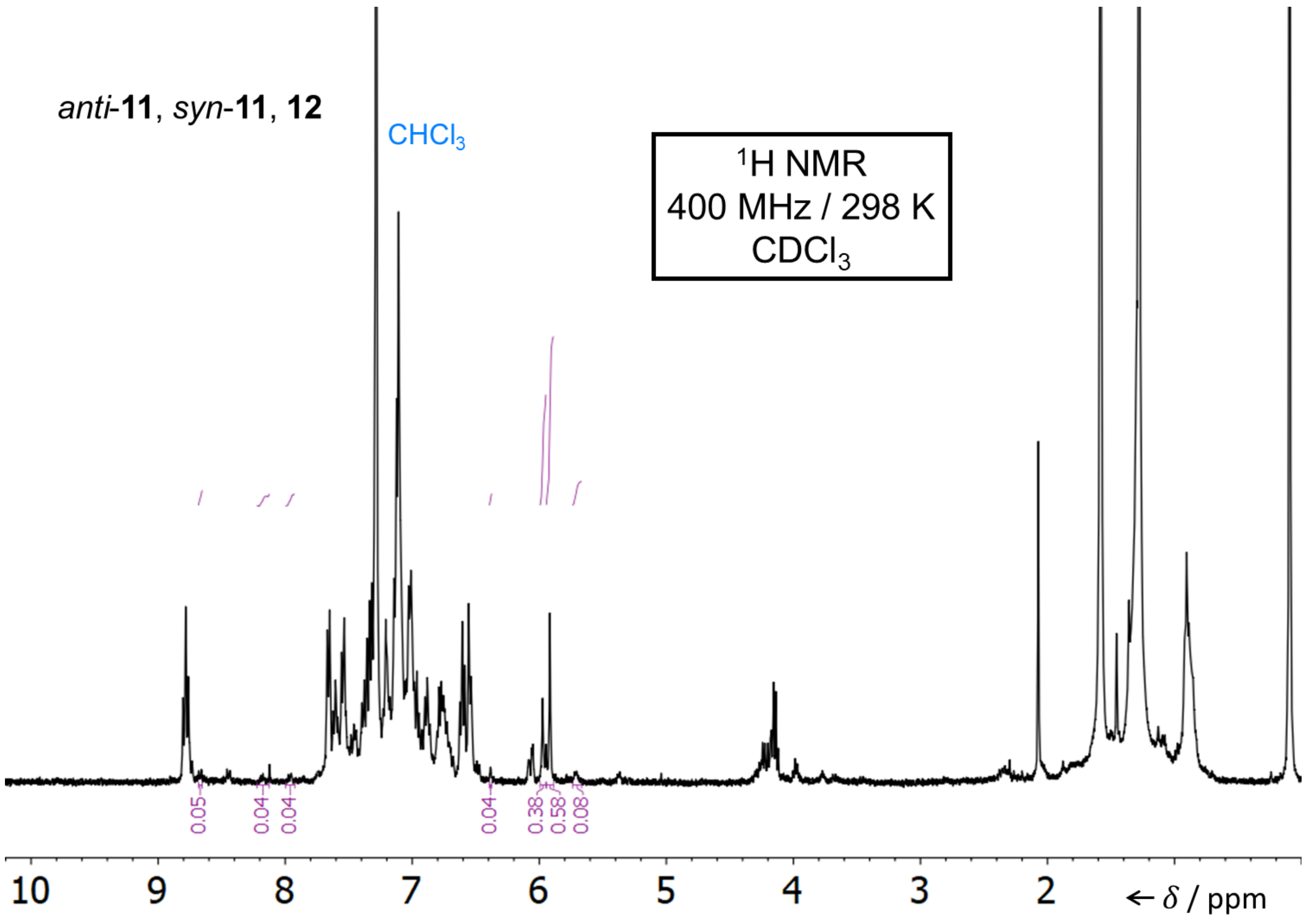

Fig. S39. ¹H NMR spectrum of the crude mixture of *anti*-**11**, *syn*-**11**, and **12** obtained from the $NaBH_4$ trapping experiment, showing the presence of 38:58:4 ratio of products (96:4 ratio of bicycloheptadiene:cycloheptatriene isomers).

3.1. Variable-Temperature (VT) NMR Spectroscopy

The ^{1}H NMR spectrum of **4**·$SbCl_6$ at room temperature exhibits broad peaks, indicating interconversion between the two isomers of **4** (see Fig. S34).

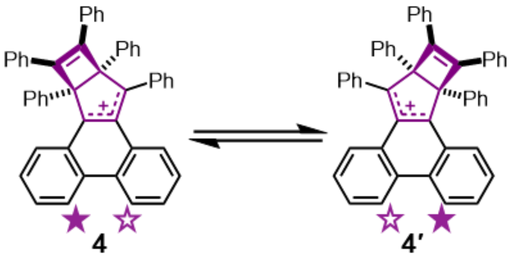

Fig. S40. Two degenerate isomers of **4**.

Cooling below the coalescence temperature of the broad peaks separates the interconverting environments into the slow-exchange regime, such that the NMR spectrum exhibits sharp peaks. A variable temperature ^{1}H NMR stack of **4**·$SbCl_6$ is shown below in Fig. S34.

Line shape analysis of the VT ^{1}H NMR spectrum of **4**·$SbCl_6$ was carried out using WinDNMR v. 7.1.

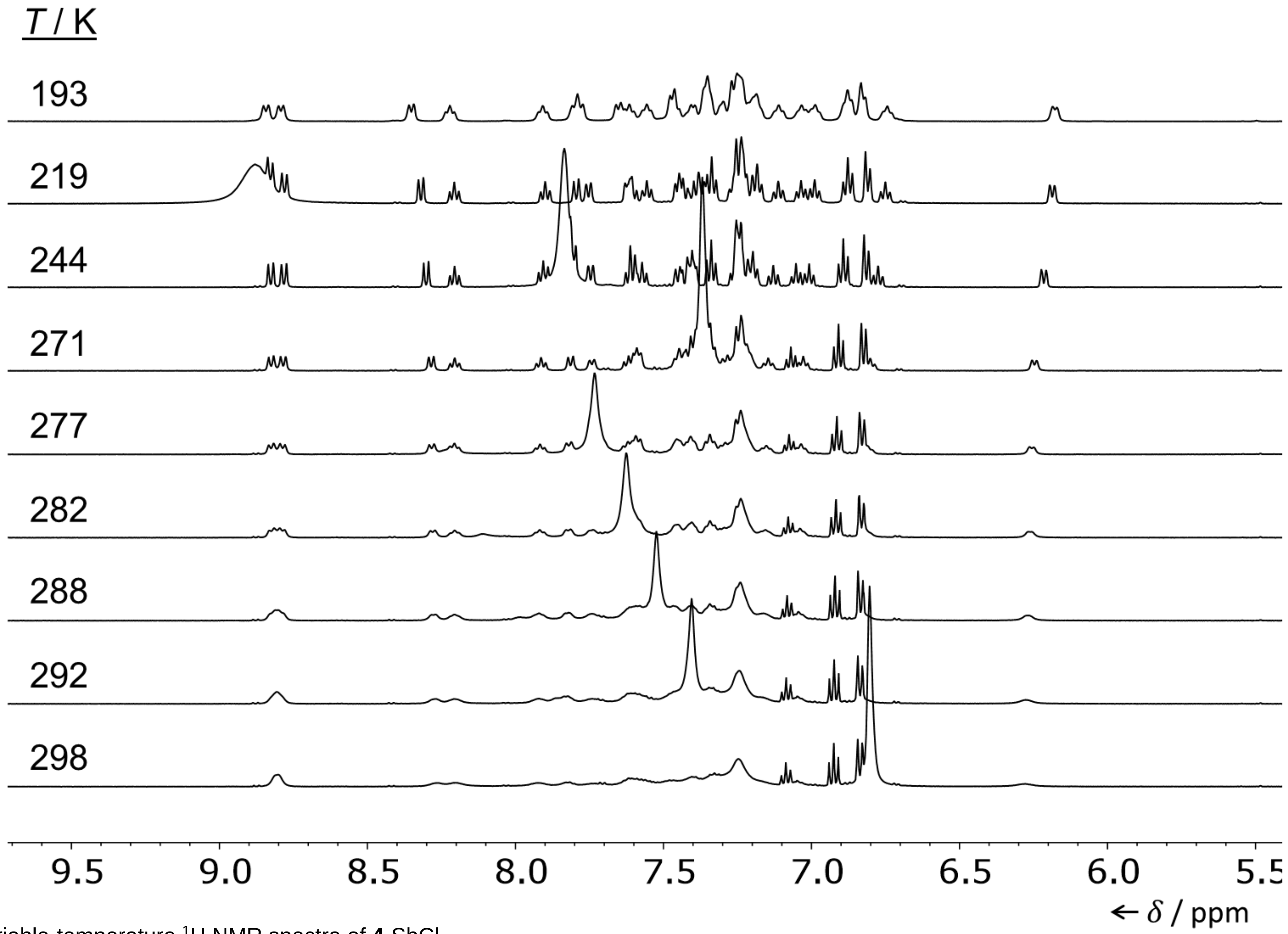

Fig. S41. Variable-temperature ^{1}H NMR spectra of **4**·$SbCl_6$

3.2. Exchange NMR Spectroscopy

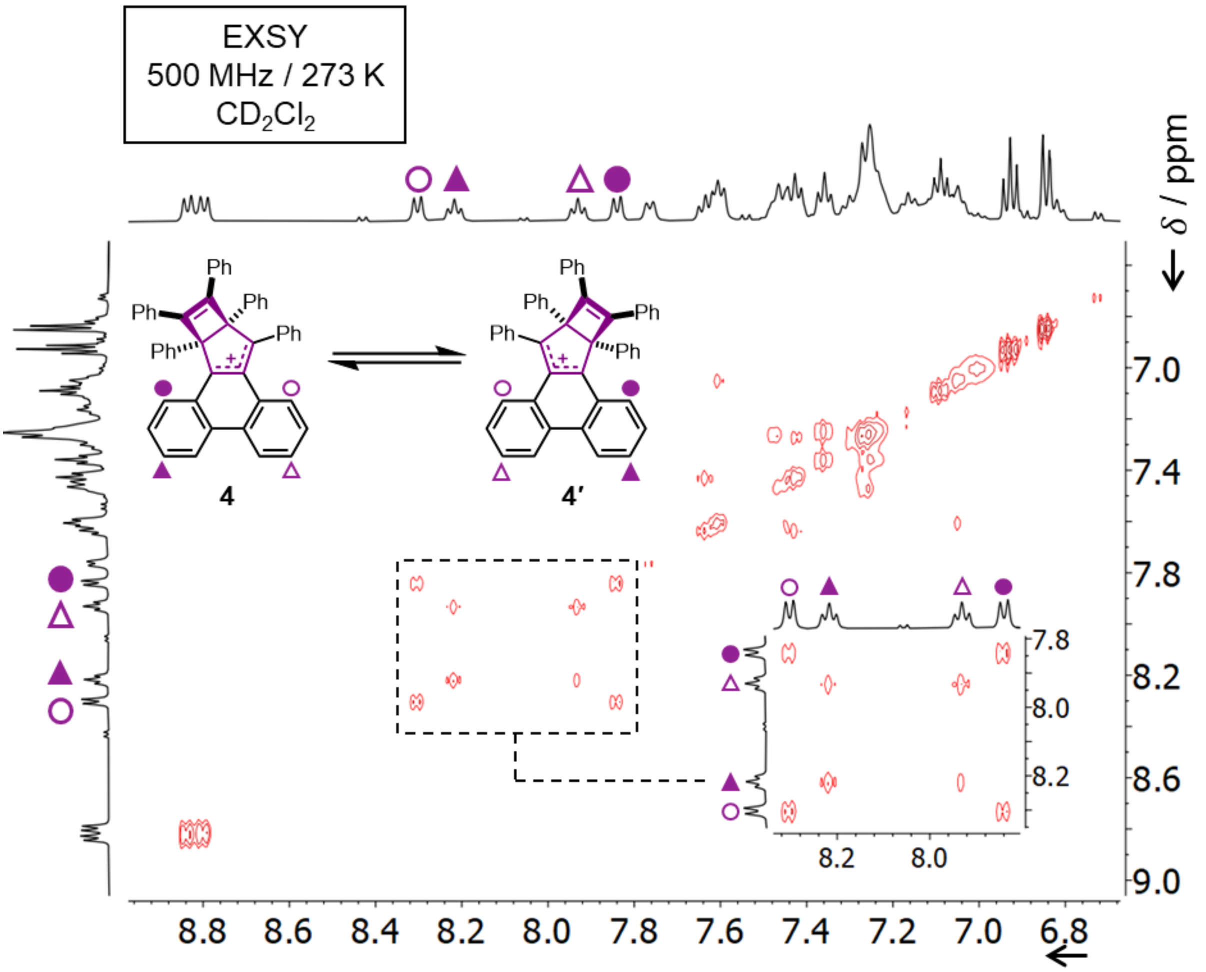

Fig. S42. Partial EXSY NMR spectrum of **4** and **4′** with a mixing time τ_m = 200 ms at 270 K. The spectrum shows exchange correlations between ^{1}H environments 4-● and 4′-○, as well as 4-▲ and 4′- Δ (see inset).

As the chosen proton environments are too far apart to exhibit through-space coupling, the possibility that the observed correlations arise from the nuclear Overhauser effect (NOE) can be ruled out. The correlations arise through dynamic exchange of isomers **4** and **4′**, which is consistent with our lineshape analysis of the ^{1}H NMR spectra at different temperatures.

4. X-ray Crystallographic Analysis

4.1. $1 \cdot ICl_2$

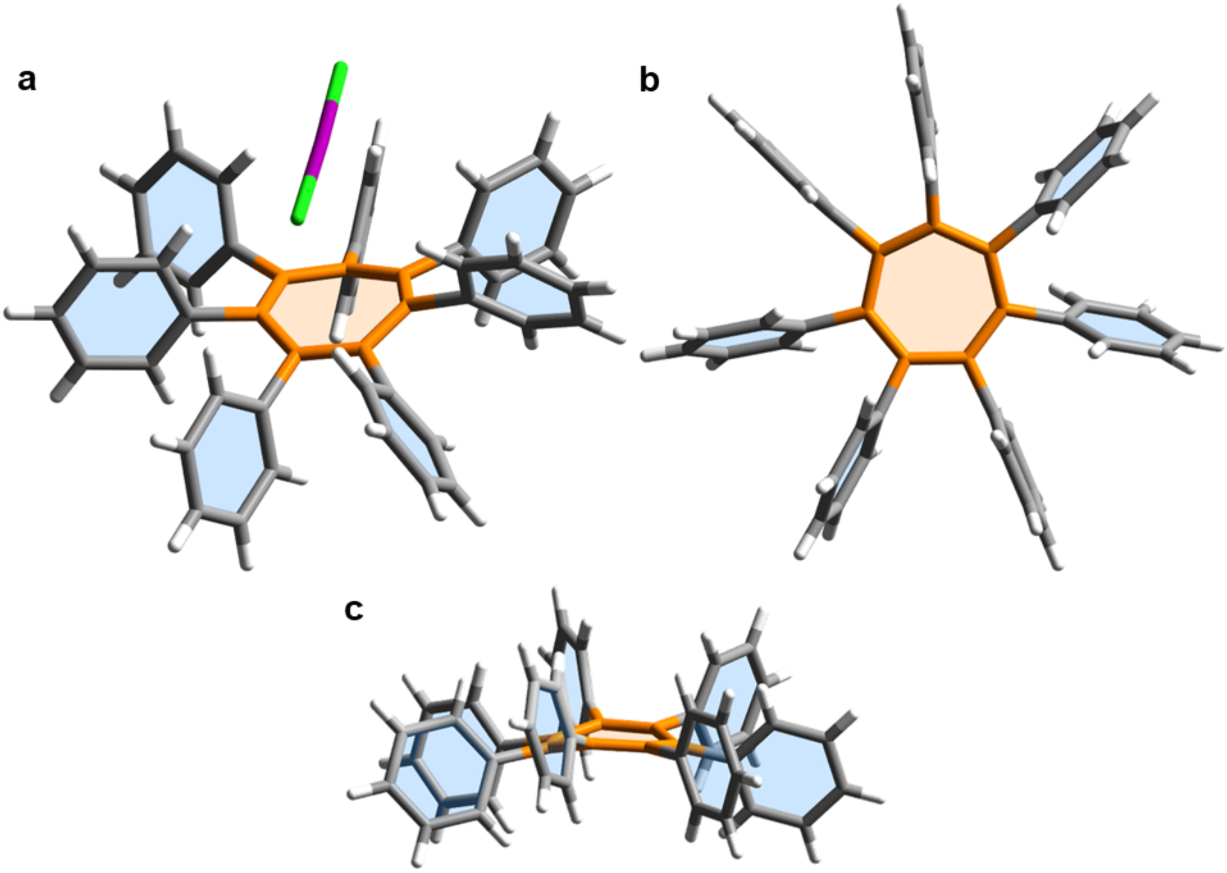

Fig. S43. Solid-state structure of **1**·ICl_2 viewed (**a**) face-on to the tropylium with ICl_2 counter-ion, (**b**) from above the tropylium and (**c**) side-on to the tropylium. The counter-ion is omitted for clarity from (**b**) and (**c**).

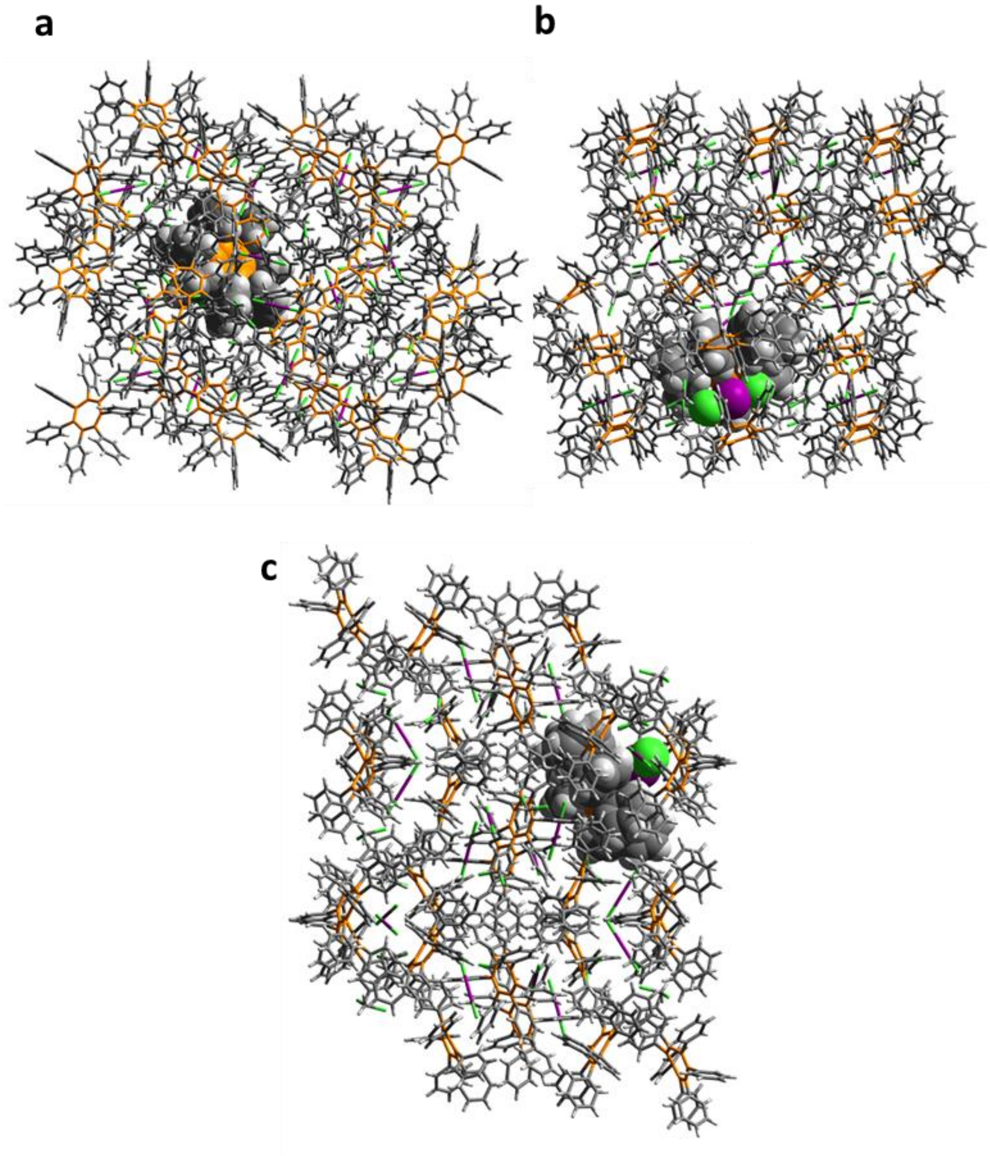

Fig. S44. Solid-state superstructure of **1**·ICl_2**.** A central molecule (space filling representation) is shown embedded in a section of the lattice made up of 2×2×2 unit cells in order to illustrate the crystal packing. Projections are viewed along the crystallographic (**a**) *a*-, (**b**) *b*-, and (**c**) *c*-axes.

Table S1. Crystal data and structure refinement for **1**·ICl_2.

	**1**·ICl_2
CCDC Number	2141786
Empirical formula	$C_{50}H_{37}Cl_4I$
Formula weight	906.49
Temperature/K	120.0
Crystal system	monoclinic
Space group	$P2_1/c$
a/Å	26.964(2)

b/Å	27.059(2)
c/Å	22.8873(17)
α/°	90
β/°	94.601(3)
γ/°	90
Volume/Å^3	16645(2)
Z	16
ρ_{calc}g/cm^3	1.447
μ/mm^{-1}	1.061
F(000)	7328.0
Crystal size/mm^3	0.3 × 0.21 × 0.11
Radiation	MoKα (λ = 0.71073)
2Θ range for data collection/°	3.874 to 57
Index ranges	-36 ≤ h ≤ 36, -36 ≤ k ≤ 36, -30 ≤ l ≤ 30
Reflections collected	314988
Independent reflections	42199 [R_{int} = 0.0636, R_{sigma} = 0.0452]
Data/restraints/parameters	42199/6/1978
Goodness-of-fit on F^2	1.033
Final R indexes [I>=2σ (I)]	R_1 = 0.0531, wR_2 = 0.1305
Final R indexes [all data]	R_1 = 0.0867, wR_2 = 0.1495
Largest diff. peak/hole / e Å^{-3}	3.07/-1.32

Crystals of **1**·ICl_2 suitable for X-ray diffraction were grown by slow evaporation of a saturated CH_2Cl_2 solution.

4.2. 8

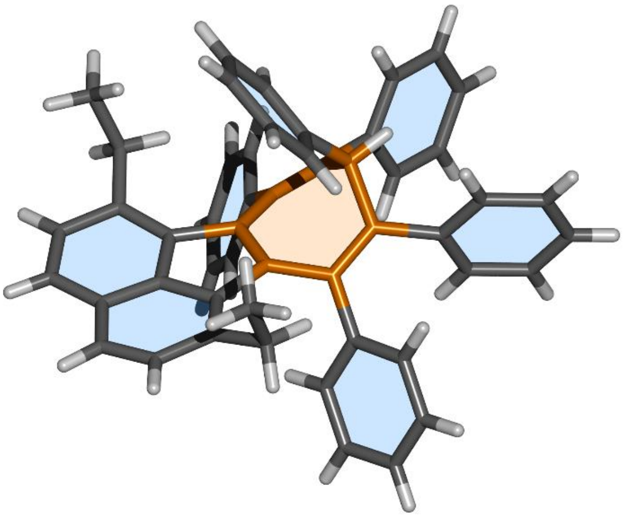

Fig. S45. Solid-state structure of **8**.

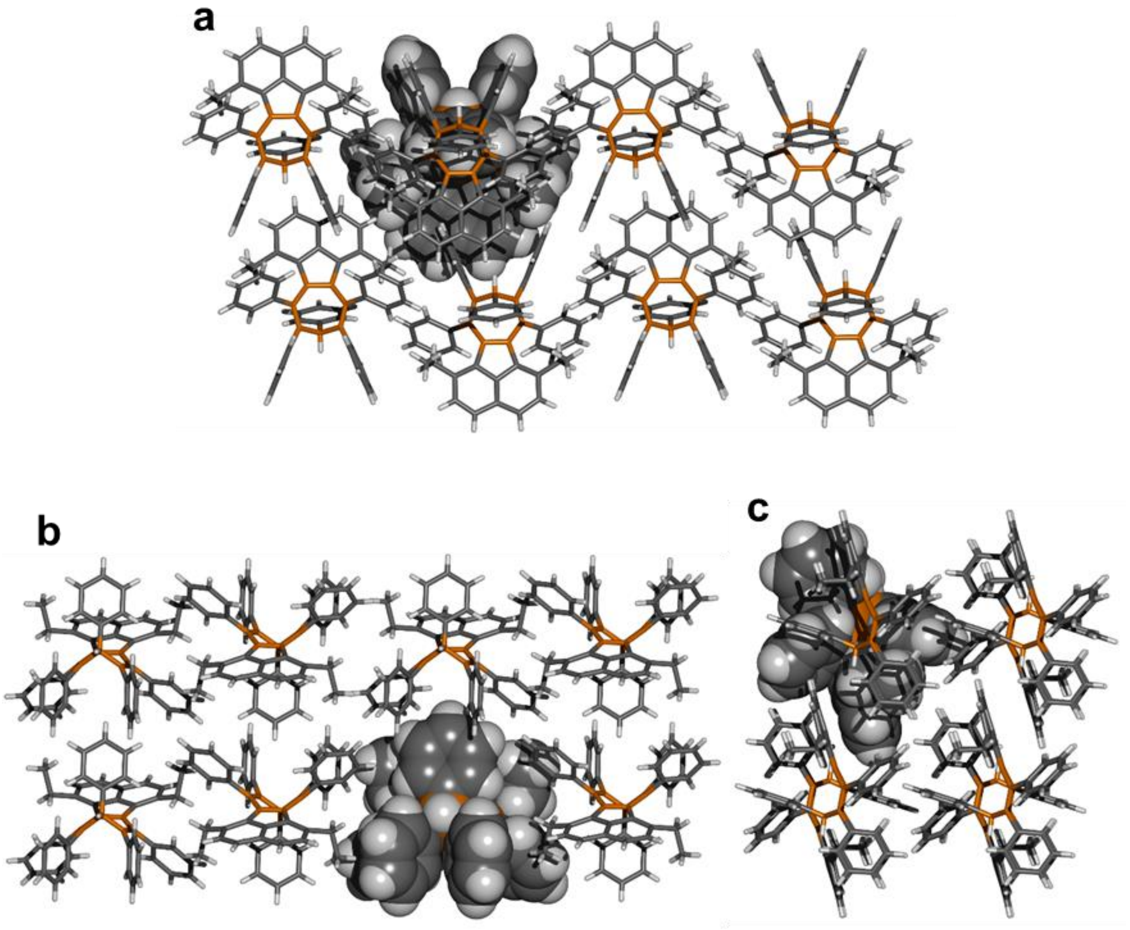

Fig. S46. Solid-state superstructure of **8.** A central molecule (space filling representation) is shown embedded in a section of the lattice made up of 2×2×2 unit cells in order to illustrate the crystal packing. Projections are viewed along the crystallographic (**a**) *a*-, (**b**) *b*-, and (**c**) *c*-axes.

Table S2. Crystal data and structure refinement for **8**.

	**8**
CCDC Number	2141790
Empirical formula	$C_{51}H_{40}$
Formula weight	652.83
Temperature/K	120.0
Crystal system	triclinic
Space group	P-1
a/Å	9.549(2)
b/Å	9.987(2)
c/Å	19.991(5)
α/°	78.699(7)
β/°	88.107(6)
γ/°	79.154(7)
Volume/Å^3	1836.1(8)
Z	2
ρ_{calc}g/cm^3	1.181
μ/mm^{-1}	0.067
F(000)	692.0
Crystal size/mm^3	0.25 × 0.05 × 0.02
Radiation	MoKα (λ = 0.71073)
2Θ range for data collection/°	4.232 to 51.994
Index ranges	-11 ≤ h ≤ 11, -12 ≤ k ≤ 11, -24 ≤ l ≤ 24
Reflections collected	30569
Independent reflections	7231 [R_{int} = 0.2068, R_{sigma} = 0.2472]
Data/restraints/parameters	7231/0/463
Goodness-of-fit on F^2	0.959
Final R indexes [I>=2σ (I)]	R_1 = 0.0769, wR_2 = 0.1307
Final R indexes [all data]	R_1 = 0.2207, wR_2 = 0.1704
Largest diff. peak/hole / e Å^{-3}	0.26/-0.24

Crystals of **8** suitable for X-ray diffraction were grown by slow evaporation of a saturated CH_2Cl_2 solution.

4.3. 3·BBr_4

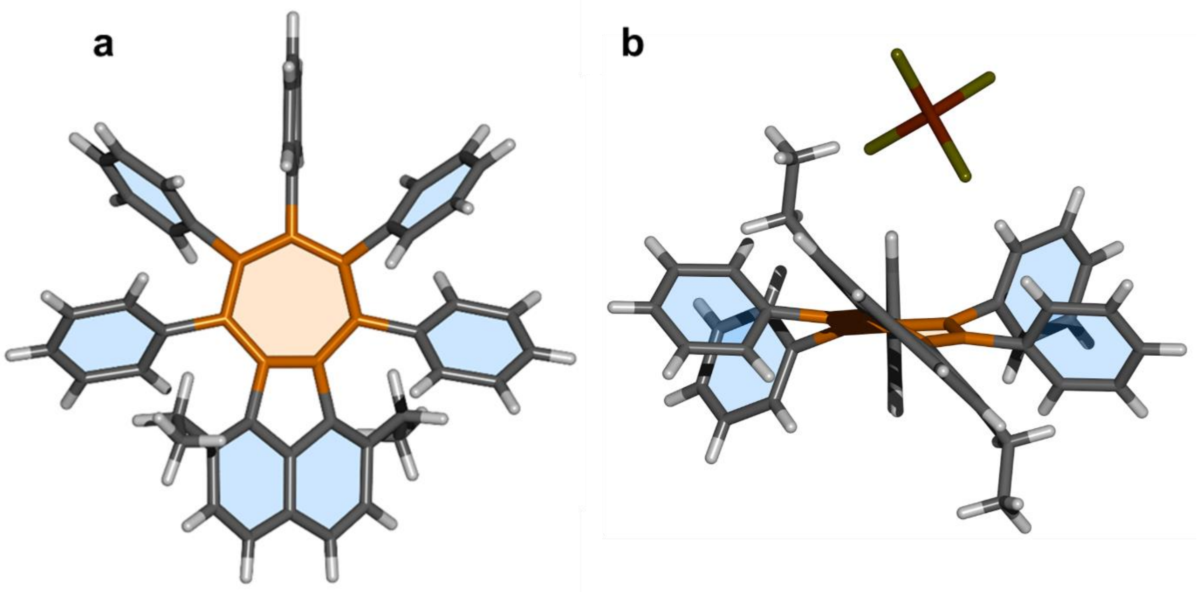

Fig. S47. Solid-state structure of **3**·BBr_4 viewed (**a**) face-on to the tropylium, omitting the counterion, and (**b**) side-on to the tropylium, including the BBr_4. The CH_2Cl_2 solvent has been omitted for clarity.

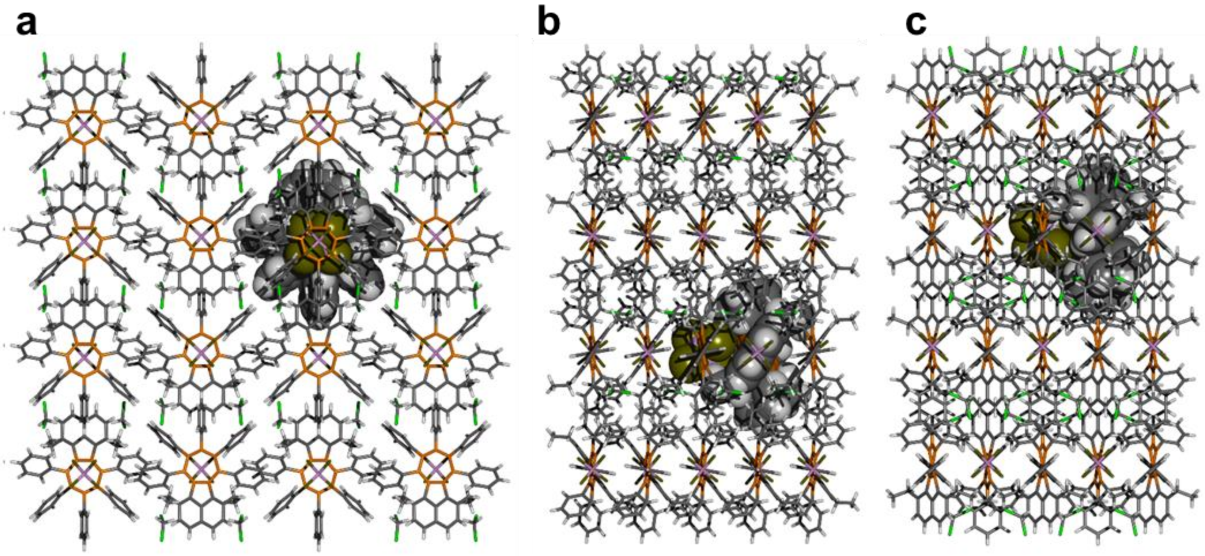

Fig. S48. Solid-state superstructure of **3**·BBr_4 (as a CH_2Cl_2 solvate). A central molecule (space filling representation) is shown embedded in a section of the lattice made up of 2×2×2 unit cells in order to illustrate the crystal packing. Projections are viewed along the crystallographic (**a**) *a*-, (**b**) *b*-, and (**c**) *c*-axes. Two solvent molecules are present for each **3** cations.

Table S3. Crystal data and structure refinement for **3**·BBr_4.

	**3**·BBr_4
CCDC Number	2141788
Empirical formula	$C_{51}H_{39}$×BBr_4×2 CH_2Cl_2
Formula weight	1152.12
Temperature/K	120.0
Crystal system	orthorhombic
Space group	$C222_1$
a/Å	10.1647(10)
b/Å	21.424(2)
c/Å	21.601(2)
α/°	90
β/°	90
γ/°	90
Volume/Å^3	4703.8(8)
Z	4
ρ_{calc}g/cm^3	1.627
μ/mm^{-1}	3.687
F(000)	2296.0
Crystal size/mm^3	0.11 × 0.06 × 0.02
Radiation	MoKα (λ = 0.71073)
2Θ range for data collection/°	1.886 to 49.998
Index ranges	-12 ≤ h ≤ 12, -25 ≤ k ≤ 25, -25 ≤ l ≤ 25
Reflections collected	23165
Independent reflections	4141 [R_{int} = 0.0979, R_{sigma} = 0.0801]
Data/restraints/parameters	4141/7/286
Goodness-of-fit on F^2	1.040
Final R indexes [I>=2σ (I)]	R_1 = 0.0876, wR_2 = 0.2249
Final R indexes [all data]	R_1 = 0.1404, wR_2 = 0.2639
Largest diff. peak/hole / e Å^{-3}	1.01/-0.94
Flack parameter	-0.008(10)

Crystals of **3**·BBr_4 suitable for X-ray diffraction were grown by slow evaporation of a solution of **3**·BBr_4 generated *in situ* from **11** and BBr_3 in anhydrous CH_2Cl_2, then layering with anhydrous hexanes in a freezer.

4.4. Diels-Alder Intermediate (S4)

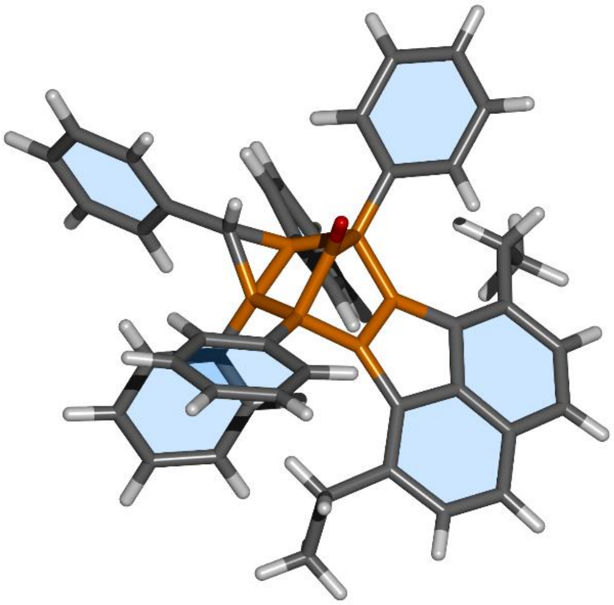

Fig. S49. Solid-state structure of **S4**.

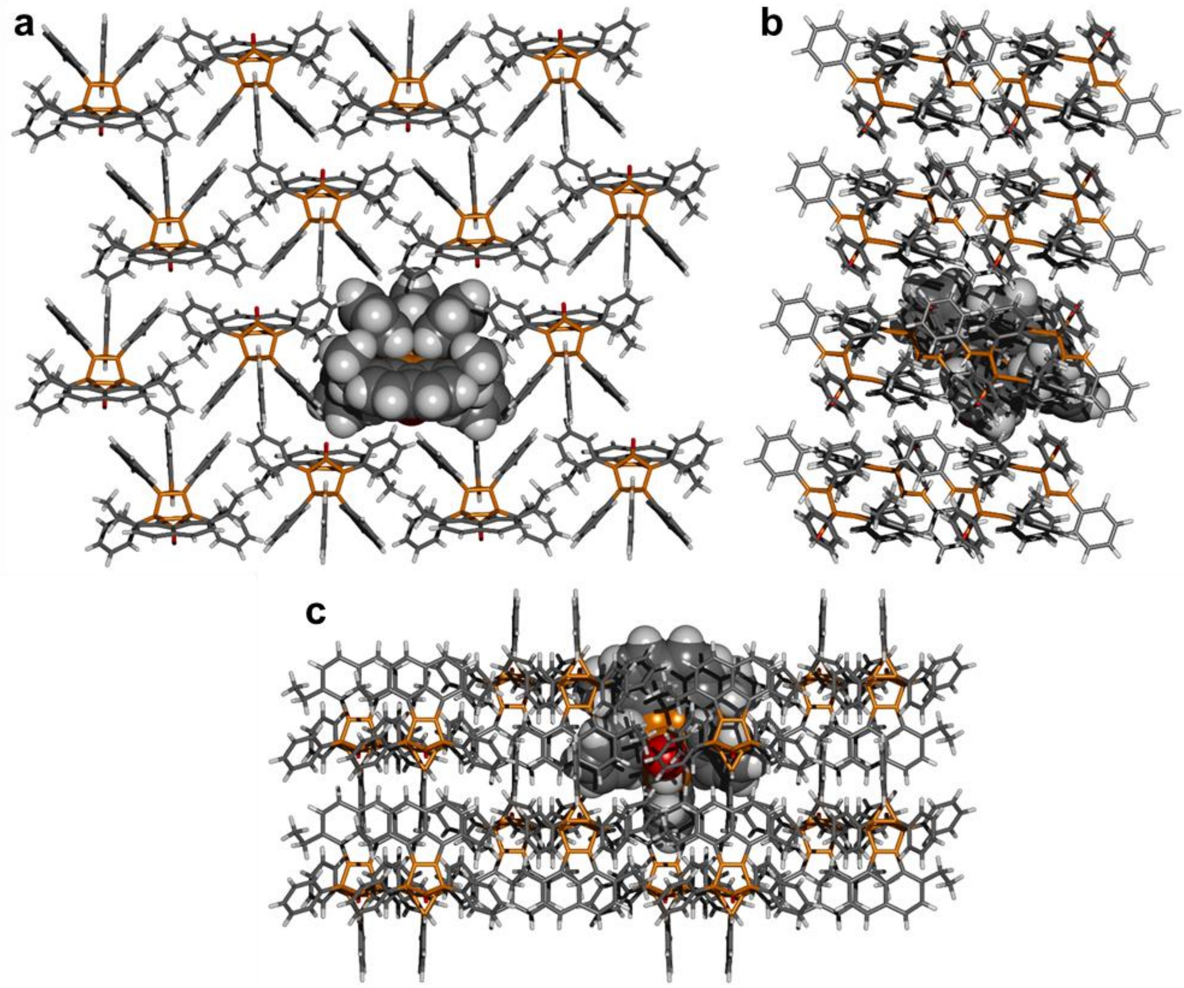

Fig. S50. Solid-state superstructure of **S4.** A central molecule (space filling representation) is shown embedded in a section of the lattice made up of 2×2×2 unit cells in order to illustrate the crystal packing. Projections are viewed along the crystallographic (**a**) *a*-, (**b**) *b*-, and (**c**) *c*-axes.

Table S4. Crystal data and structure refinement for **S4**.

	**S4**
CCDC Number	2141795
Empirical formula	$C_{52}H_{40}O$
Formula weight	680.84
Temperature/K	120.0
Crystal system	monoclinic
Space group	$P2_1/c$
a/Å	9.6957(2)
b/Å	20.4272(5)
c/Å	18.3325(4)
α/°	90
β/°	96.6351(13)
γ/°	90
Volume/Å^3	3606.54(14)
Z	4
ρ_{calc}g/cm^3	1.254
μ/mm^{-1}	0.555
F(000)	1440.0
Crystal size/mm^3	0.29 × 0.19 × 0.13
Radiation	CuKα (λ = 1.54178)
2Θ range for data collection/°	6.502 to 141.986
Index ranges	-11 ≤ h ≤ 11, -24 ≤ k ≤ 25, -21 ≤ l ≤ 22
Reflections collected	44694
Independent reflections	6951 [R_{int} = 0.0492, R_{sigma} = 0.0292]
Data/restraints/parameters	6951/0/638
Goodness-of-fit on F^2	1.022
Final R indexes [I>=2σ (I)]	R_1 = 0.0424, wR_2 = 0.1080
Final R indexes [all data]	R_1 = 0.0577, wR_2 = 0.1170
Largest diff. peak/hole / e Å^{-3}	0.30/-0.24

Crystals of **S4** suitable for X-ray diffraction were grown by slow evaporation of a saturated CH_2Cl_2 solution of **S4** layered with hexanes

4.5. 1,6-Diethyl-7,8,9,10-tetraphenylfluoranthene (S5)

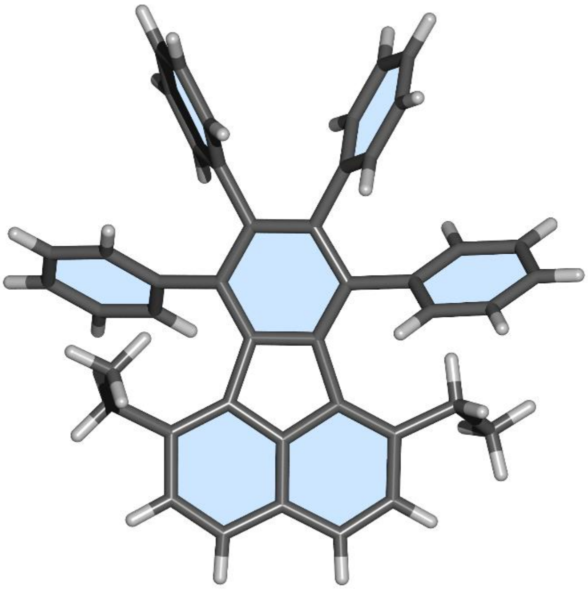

Fig. S51. Solid-state structure of **S5.**

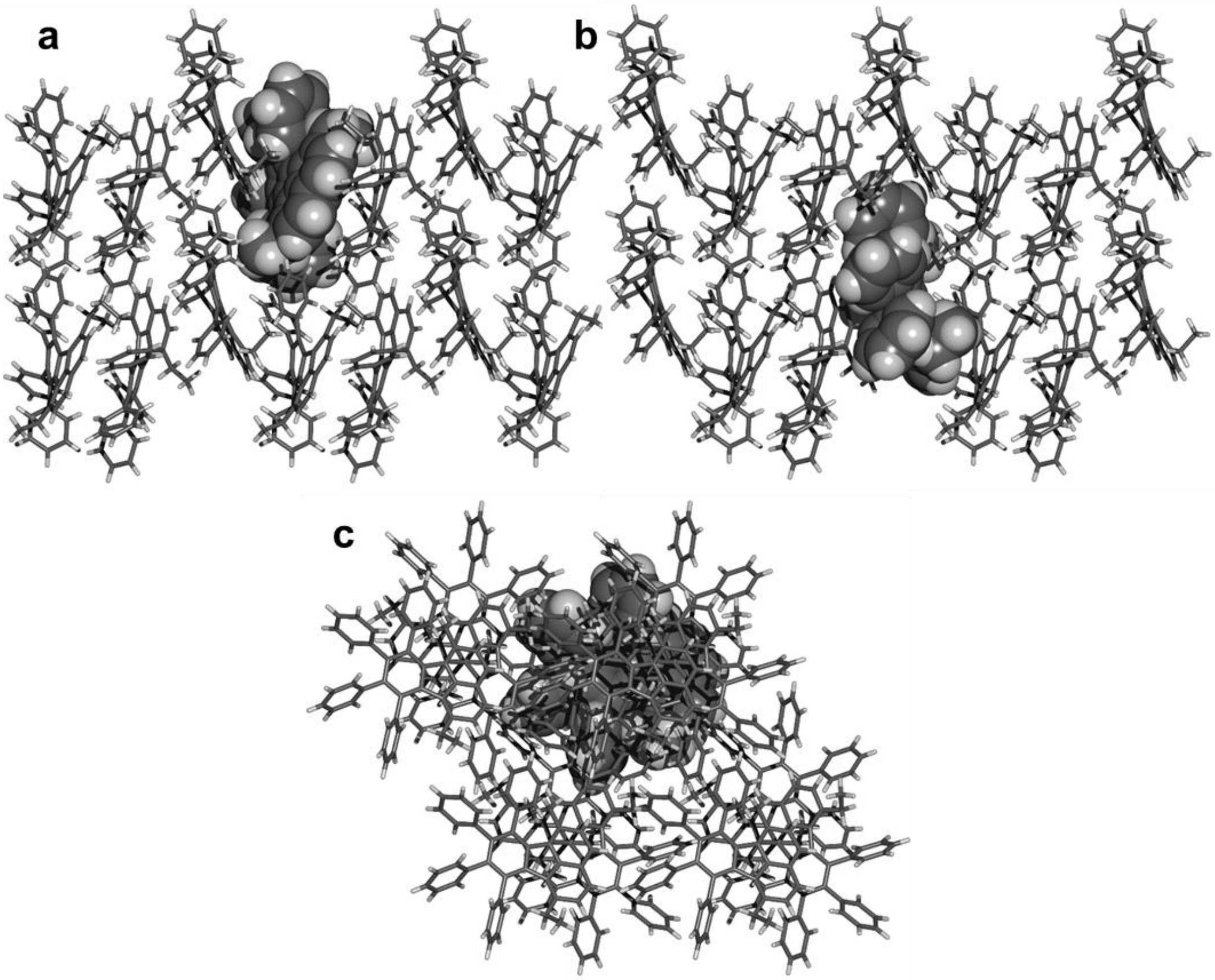

Fig. S52. Solid-state superstructure of **S5.** A central molecule (space filling representation) is shown embedded in a section of the lattice made up of 2×2×2 unit cells in order to illustrate the crystal packing. Projections are viewed along the crystallographic (**a**) *a*-, (**b**) *b*-, and (**c**) *c*-axes.

Table S5. Crystal data and structure refinement for **S5**.

	**S5**
CCDC Number	2141796
Empirical formula	$C_{44}H_{34}$
Formula weight	562.71
Temperature/K	120.0
Crystal system	trigonal
Space group	$P3_2$
a/Å	13.1637(2)
b/Å	13.1637(2)
c/Å	15.4431(4)
α/°	90
β/°	90
γ/°	120
Volume/Å^3	2317.51(9)
Z	3
ρ_{calc}g/cm^3	1.210
μ/mm^{-1}	0.068
F(000)	894.0
Crystal size/mm^3	0.32 × 0.16 × 0.07
Radiation	Mo Kα (λ = 0.71073)
2Θ range for data collection/°	4.442 to 57.966
Index ranges	-17 ≤ h ≤ 17, -17 ≤ k ≤ 17, -21 ≤ l ≤ 21
Reflections collected	39603
Independent reflections	8165 [R_{int} = 0.0461, R_{sigma} = 0.0402]
Data/restraints/parameters	8165/2/404
Goodness-of-fit on F^2	1.032
Final R indexes [I>=2σ (I)]	R_1 = 0.0542, wR_2 = 0.1169
Final R indexes [all data]	R_1 = 0.0633, wR_2 = 0.1217
Largest diff. peak/hole / e Å^{-3}	0.60/-0.44
Flack parameter	-0.1(10)

Crystals of **S5** suitable for X-ray diffraction were grown by slow evaporation of a saturated solution of **S5** in hexanes–CH_2Cl_2.

4.6. 9

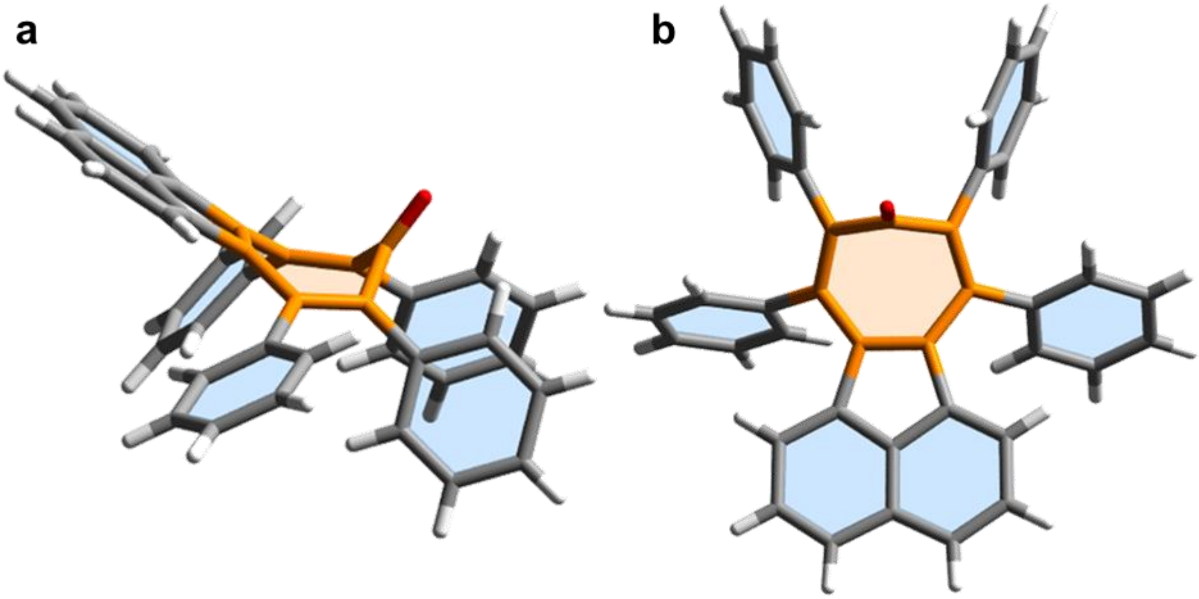

Fig. S53. Solid-state structure of **9** viewed (**a**) side-on to the tropone and (**b**) face-on to the tropone.

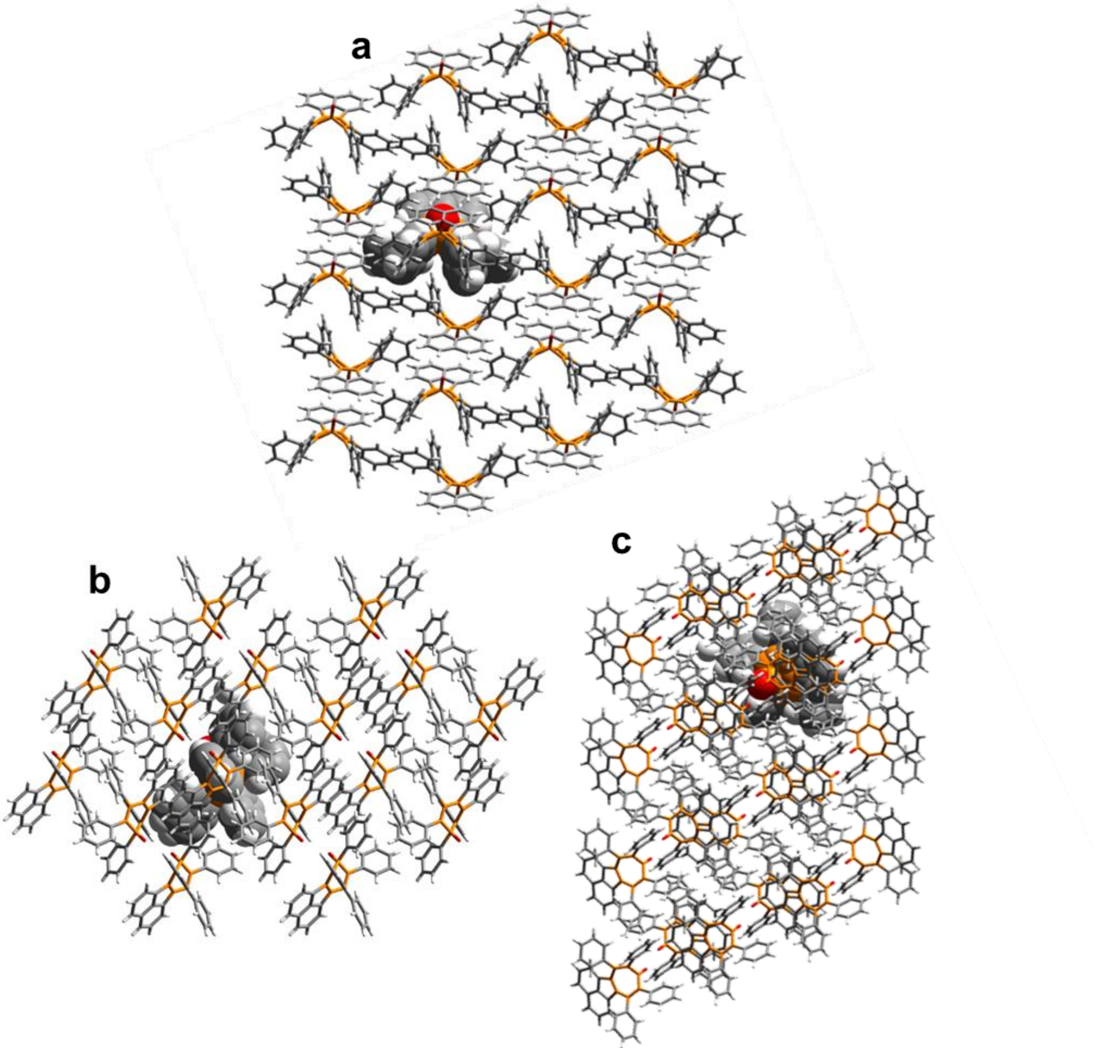

Fig. S54. Solid-state superstructure of **9.** A central molecule (space filling representation) is shown embedded in a section of the lattice made up of 2×2×2 unit cells in order to illustrate the crystal packing. Projections are viewed along the crystallographic (**a**) *a*-, (**b**) *b*-, and (**c**) *c*-axes.

Table S6. Crystal data and structure refinement for **9**.

	**9**
CCDC Number	2141791
Empirical formula	$C_{41}H_{26}O$
Formula weight	534.62
Temperature/K	120.0
Crystal system	triclinic
Space group	P-1
a/Å	10.3889(6)
b/Å	11.1459(7)
c/Å	14.2952(9)
α/°	103.530(2)
β/°	103.151(2)
γ/°	112.377(2)
Volume/$Å^3$	1392.85(15)
Z	2
$\rho_{calc}g/cm^3$	1.275
μ/mm^{-1}	0.075
F(000)	560.0
Crystal size/mm^3	0.31 × 0.13 × 0.08
Radiation	MoKα (λ = 0.71073)
2Θ range for data collection/°	4.23 to 58
Index ranges	-14 ≤ h ≤ 14, -15 ≤ k ≤ 15, -19 ≤ l ≤ 19
Reflections collected	29172
Independent reflections	7415 [R_{int} = 0.0557, R_{sigma} = 0.0678]
Data/restraints/parameters	7415/0/379
Goodness-of-fit on F^2	1.030
Final R indexes [I>=2σ (I)]	R_1 = 0.0523, wR_2 = 0.1043
Final R indexes [all data]	R_1 = 0.1002, wR_2 = 0.1190
Largest diff. peak/hole / e $Å^{-3}$	0.28/-0.28

Crystals of **9** suitable for X-ray diffraction were grown by slow evaporation of a saturated solution of **9** in CH_2Cl_2–hexanes.

4.7. S2

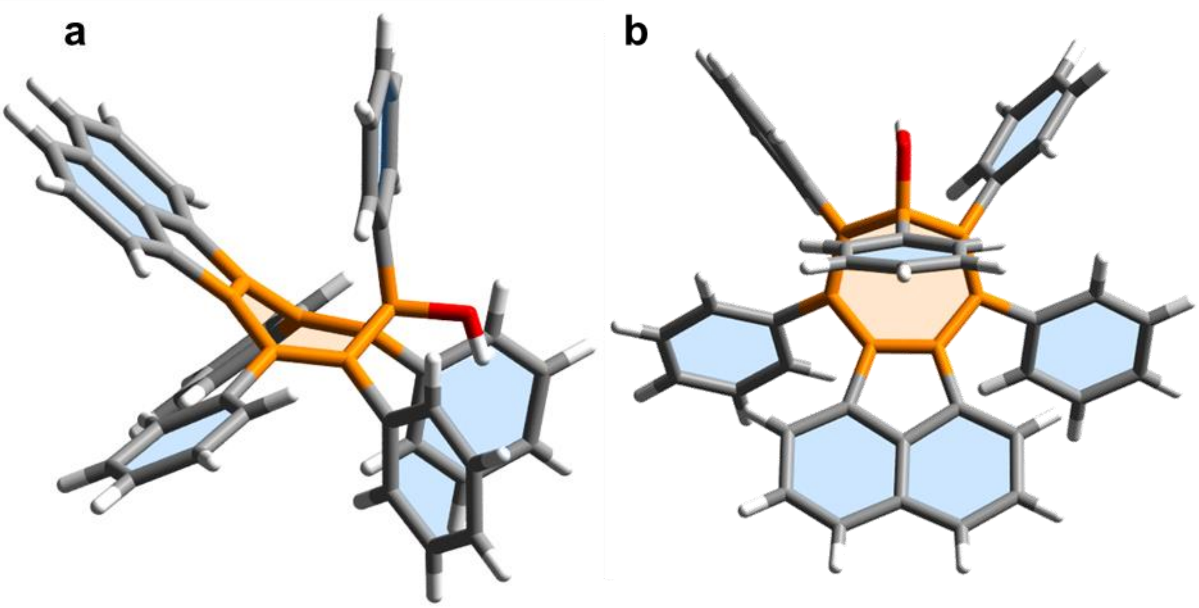

Fig. S55. Solid-state structure of **S2** viewed (**a**) side-on and (**b**) face-on to the seven-membered ring.

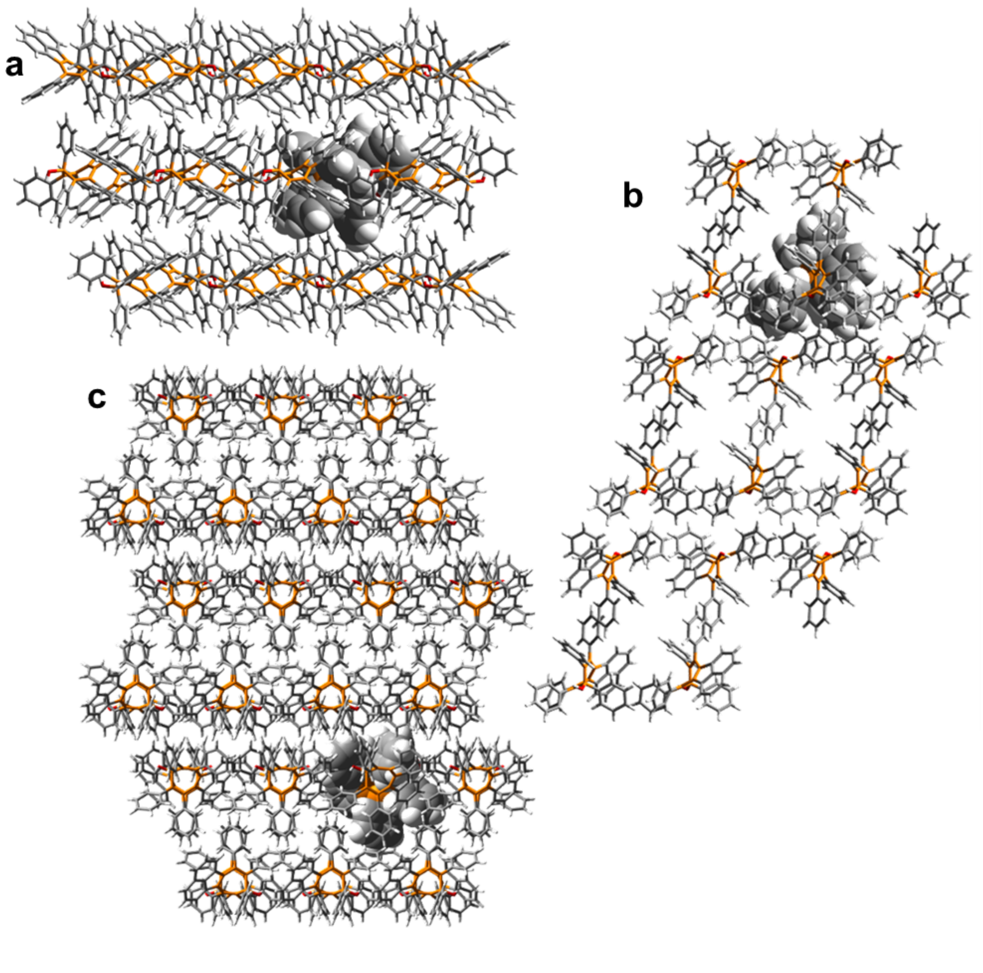

Fig. S56. Solid-state superstructure of **S2.** A central molecule (space filling representation) is shown embedded in a section of the lattice made up of 2×2×2 unit cells in order to illustrate the crystal packing. Projections are viewed along the crystallographic (**a**) *a*-, (**b**) *b*-, and (**c**) *c*-axes.

Table S7. Crystal data and structure refinement for **S2**.

	**S2**
CCDC Number	2141793
Empirical formula	$C_{51}H_{40}O_2$
Formula weight	684.83
Temperature/K	120.0
Crystal system	monoclinic
Space group	$P2_1/c$
a/Å	19.5114(12)
b/Å	9.7395(6)
c/Å	19.9893(12)
α/°	90
β/°	107.714(4)
γ/°	90
Volume/$Å^3$	3618.5(4)
Z	4
$\rho_{calc}g/cm^3$	1.257
μ/mm^{-1}	0.577
F(000)	1448.0
Crystal size/mm^3	0.08 × 0.05 × 0.02
Radiation	CuKα (λ = 1.54178)
2Θ range for data collection/°	4.754 to 134.988
Index ranges	-23 ≤ h ≤ 23, -11 ≤ k ≤ 11, -22 ≤ l ≤ 23
Reflections collected	46456
Independent reflections	6478 [R_{int} = 0.3108, R_{sigma} = 0.2019]
Data/restraints/parameters	6478/13/475
Goodness-of-fit on F^2	0.987
Final R indexes [I>=2σ (I)]	R_1 = 0.1092, wR_2 = 0.2279
Final R indexes [all data]	R_1 = 0.2278, wR_2 = 0.2929
Largest diff. peak/hole / e $Å^{-3}$	0.39/-0.31

Crystals of **S2** suitable for X-ray diffraction were grown by slow evaporation of a saturated solution of Et_2O layered with hexanes.

4.8. 2·$SbCl_6$ (120 K)

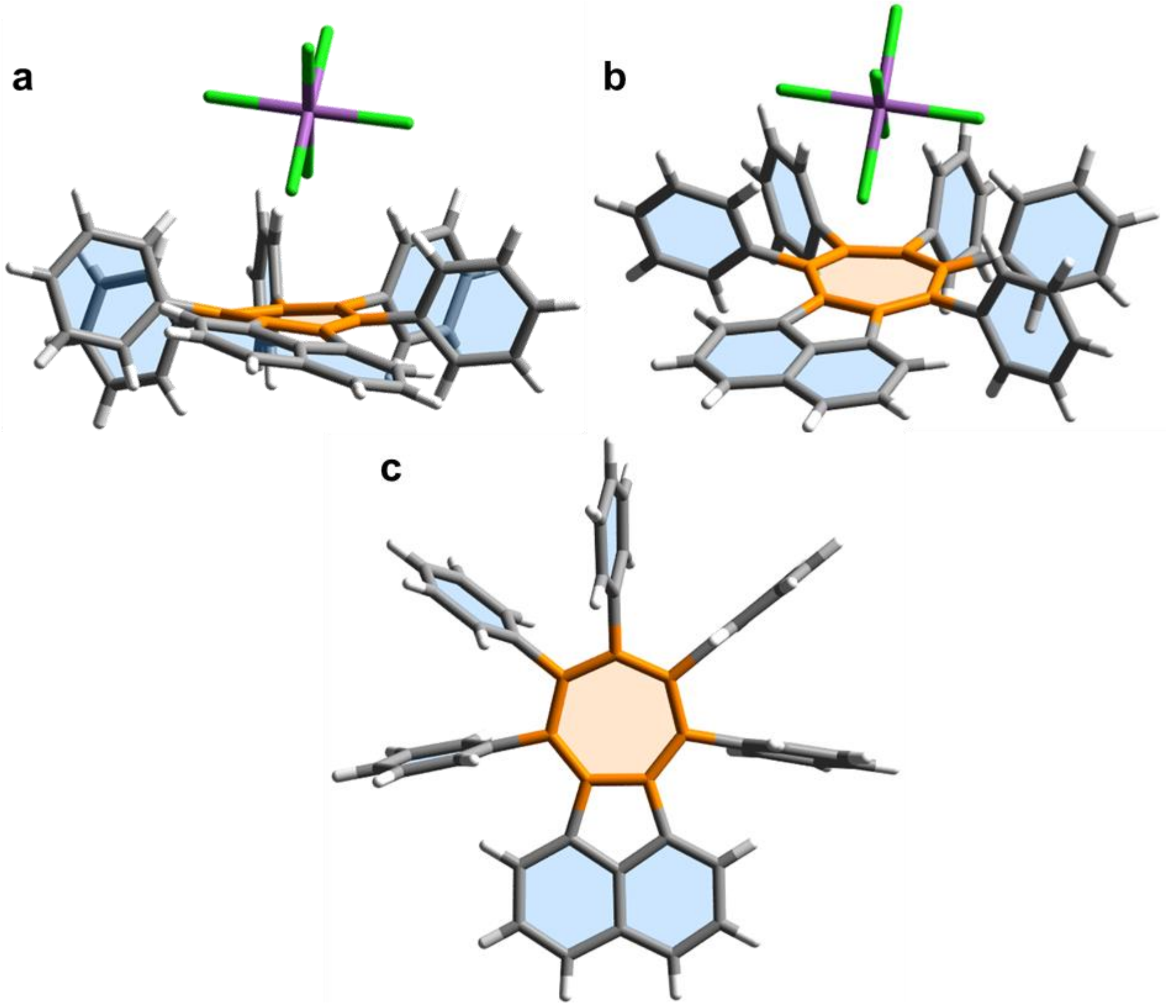

Fig. S57. Solid-state structure of **2**·$SbCl_6$ (120 K) in its (**a**) twisted and (**b**) shallow boat conformations, and (**c**) viewed face-on. The counter-ion is omitted for clarity from (**c**). $CHCl_3$ present in the unit cell is also omitted for clarity.

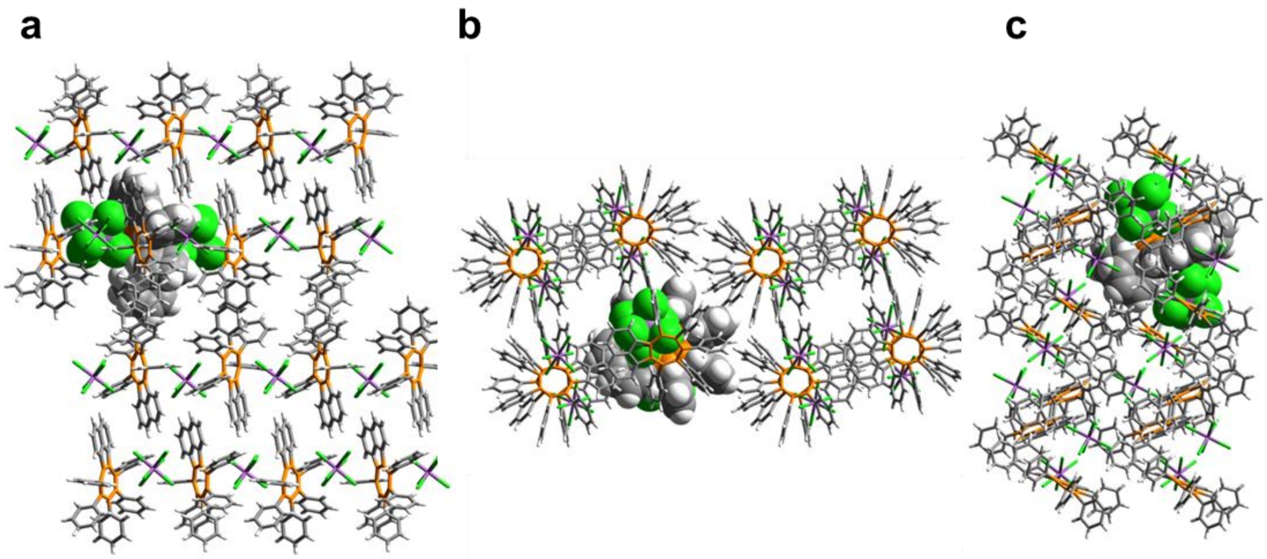

Fig. S58. Solid-state superstructure of **2**·$SbCl_6$ (120 K)**.** A central molecule (space filling representation) is shown embedded in a section of the lattice made up of 2×2×2 unit cells in order to illustrate the crystal packing. Projections are viewed along the crystallographic (**a**) *a*-, (**b**) *b*-, and (**c**) *c*-axes.

Table S8. Crystal data and structure refinement for **2**·$SbCl_6$ at 120 K.

	**2**·$SbCl_6$
CCDC Number	2141787
Empirical formula	$C_{47}H_{31}$ × $SbCl_6$ × 1.5 $CHCl_3$
Formula weight	1109.22
Temperature/K	120.0
Crystal system	triclinic
Space group	P-1
a/Å	11.853(2)
b/Å	17.680(3)
c/Å	23.961(4)
α/°	76.709(5)
β/°	76.366(5)
γ/°	89.443(6)
Volume/Å^3	4743.8(14)
Z	4
ρ_{calc}g/cm^3	1.553
μ/mm^{-1}	1.207
F(000)	2212.0
Crystal size/mm^3	0.27 × 0.24 × 0.02
Radiation	MoKα (λ = 0.71073)
2Θ range for data collection/°	3.818 to 54
Index ranges	-15 ≤ h ≤ 14, -22 ≤ k ≤ 22, -30 ≤ l ≤ 30
Reflections collected	73608
Independent reflections	20630 [R_{int} = 0.0645, R_{sigma} = 0.0751]
Data/restraints/parameters	20630/6/1082
Goodness-of-fit on F^2	1.060
Final R indexes [I>=2σ (I)]	R_1 = 0.0890, wR_2 = 0.2404
Final R indexes [all data]	R_1 = 0.1134, wR_2 = 0.2664
Largest diff. peak/hole / e Å^{-3}	2.21/-2.99

Crystals of **2**·$SbCl_6$ suitable for X-ray diffraction were grown by slow evaporation of a saturated CH_2Cl_2 solution.

4.8. 2·$SbCl_6$ (270 K)

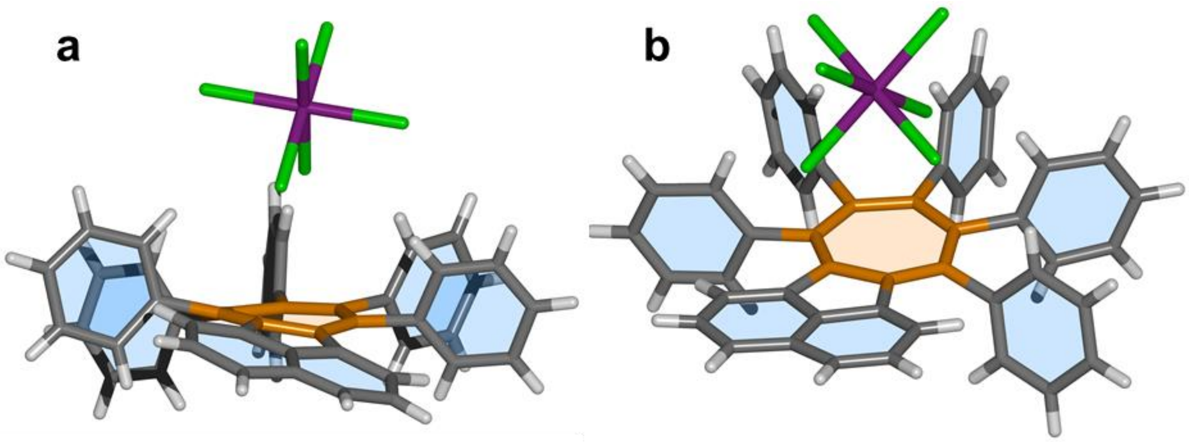

Fig. S59. Solid-state structure of **2**·$SbCl_6$ **(**270 K) in its (**a**) twisted and (**b**) shallow boat conformations. $CHCl_3$ present in the unit cell is also omitted for clarity

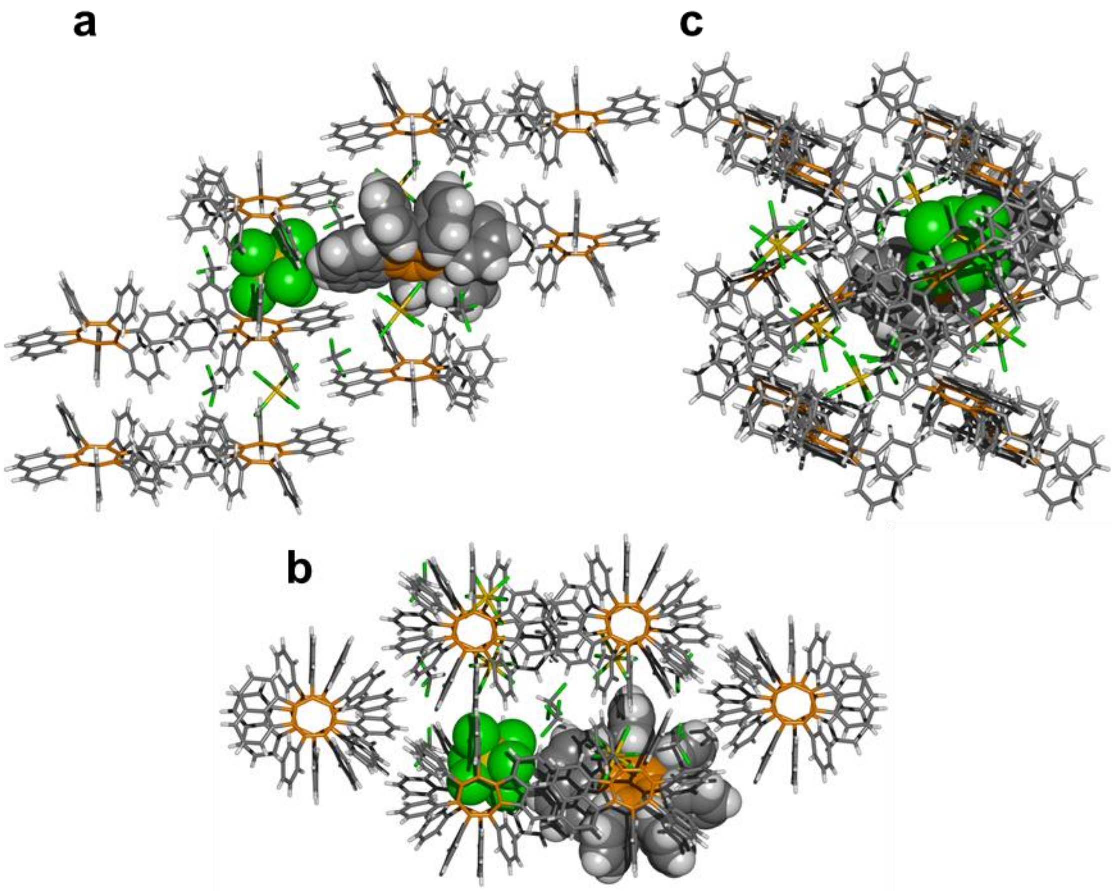

Fig. S60. Solid-state superstructure of **2**·$SbCl_6$ (270 K)**.** A central molecule (space filling representation) is shown embedded in a section of the lattice made up of 2×2×2 unit cells in order to illustrate the crystal packing. Projections are viewed along the crystallographic (**a**) *a*-, (**b**) *b*-, and (**c**) *c*-axes.

Table S9. Crystal data and structure refinement for **2**·$SbCl_6$ (270 K).

**2**·$SbCl_6$	
CCDC Number	2173731
Empirical formula	$C_{47}H_{31}$ × $SbCl_6$ × $CHCl_3$
Formula weight	1049.53
Temperature/K	270.00
Crystal system	triclinic
Space group	P-1
a/Å	11.953(4)
b/Å	17.868(5)
c/Å	24.504(7)
α/°	104.594(9)
β/°	103.956(9)
γ/°	90.055(10)
Volume/Å3	4905(2)
Z	4
ρcalcg/cm3	1.421
μ/mm 1	1.084
F(000)	2096.0
Crystal size/mm3	0.28 × 0.09 × 0.06
Radiation	Mo Kα (λ = 0.71073)
2Θ range for data collection/°	4.106 to 54.998
Index ranges	-15 ≤ h ≤ 15, -23 ≤ k ≤ 23, -31 ≤ l ≤ 31
Reflections collected	93109
Independent reflections	22495 [Rint = 0.1009, Rsigma = 0.1095]
Data/restraints/parameters	22495/274/1100
Goodness-of-fit on F2	1.064
Final R indexes [I>=2σ (I)]	R1 = 0.0998, wR2 = 0.2292
Final R indexes [all data]	R1 = 0.1612, wR2 = 0.2645
Largest diff. peak/hole / e Å-3	1.94/-0.91

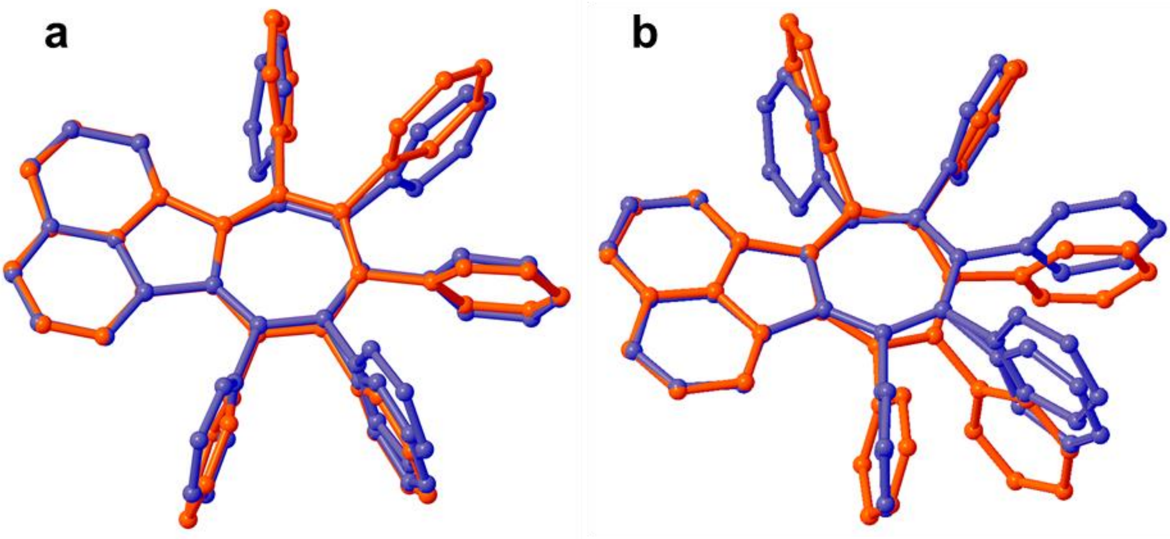

Fig. S61. Overlays of the solid-state structures of **2**·$SbCl_6$ at 120 K (orange) and 270 K (blue). Panel (**a**) shows the twisted conformer and (**b**) shows the planar conformer.

Table S10. Comparison of key structural data for the twisted conformer of **2**·$SbCl_6$ at low temperatures (T = 120 K) and high temperatures (T = 270 K).

T / K	120	270
Φ_{bow} / °	6.7	2.7
Φ_{stern} / °	13.7	14.0
$\varphi(C_4\text{-}C_2\text{-}C_\beta\text{-}C_{\beta'})$ / °	18.4	17.6

4.10. 10

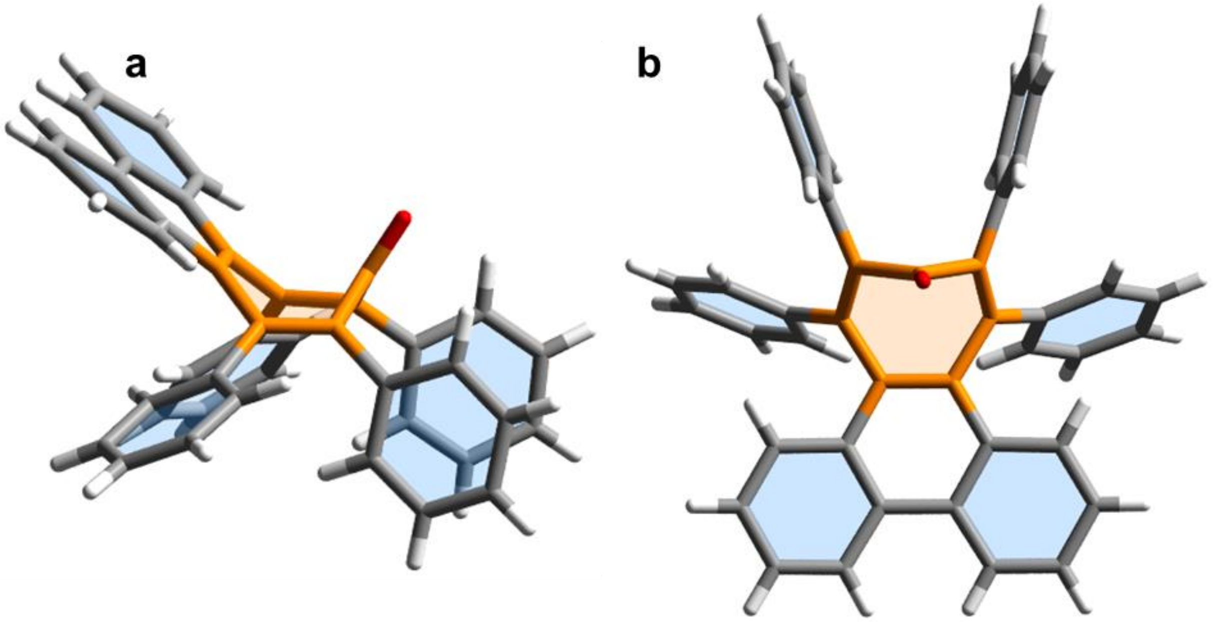

Fig. S62. Solid-state structure of **10** viewed (**a**) side-on and (**b**) face-on to the tropone.

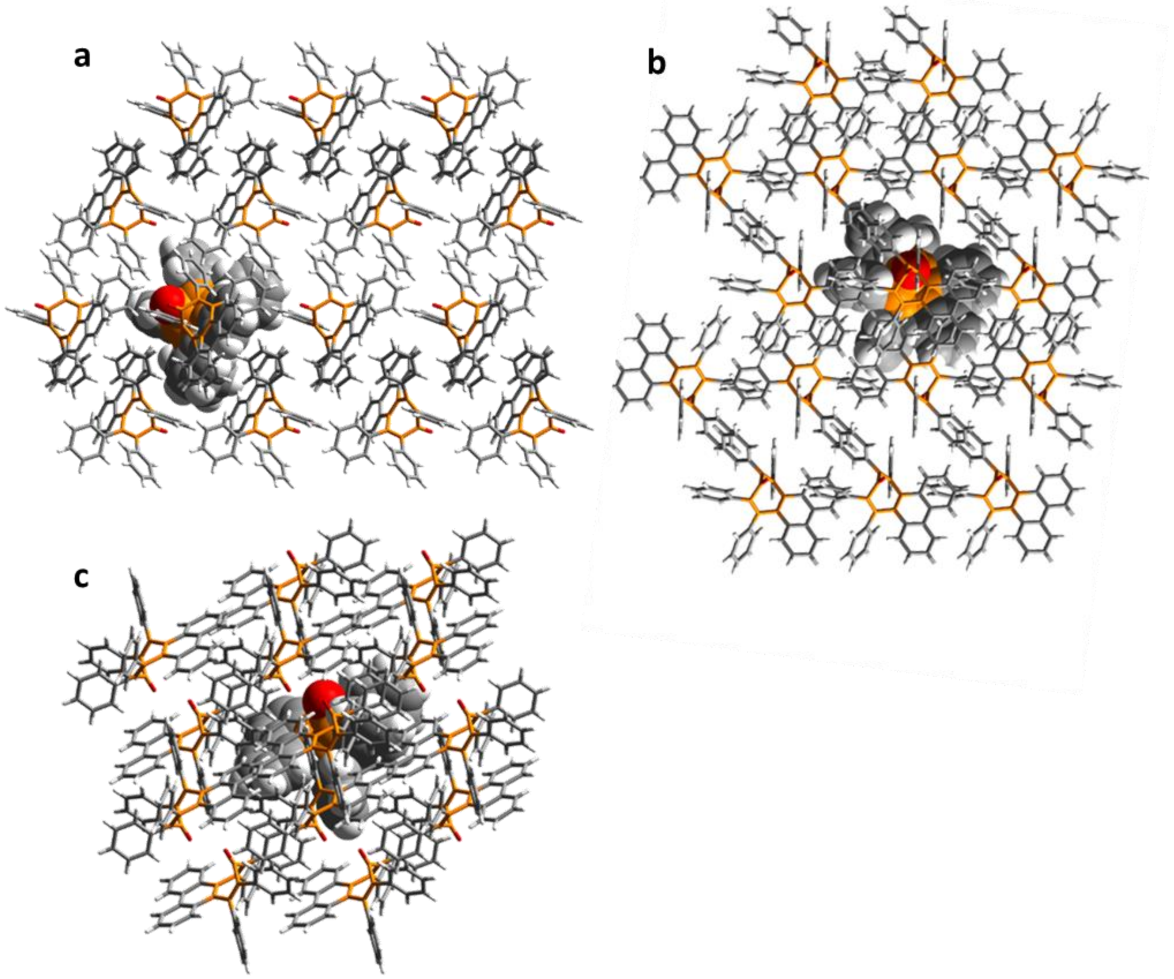

Fig. S63. Solid-state superstructure of **10.** A central molecule (space filling representation) is shown embedded in a section of the lattice made up of 2×2×2 unit cells in order to illustrate the crystal packing. Projections are viewed along the crystallographic (**a**) *a*-, (**b**) *b*-, and (**c**) *c*-axes.

Table S11. Crystal data and structure refinement for **10**.

	10
CCDC Number	2141792
Empirical formula	$C_{43}H_{28}O$
Formula weight	560.65
Temperature/K	120.0
Crystal system	triclinic
Space group	P-1
a/Å	9.3113(6)
b/Å	10.5095(7)
c/Å	16.2093(10)
α/°	84.875(3)
β/°	80.864(3)
γ/°	75.921(3)
Volume/Å^3	1516.90(17)
Z	2
ρ_{calc}g/cm^3	1.227
μ/mm^{-1}	0.072
F(000)	588.0
Crystal size/mm^3	0.19 × 0.14 × 0.06
Radiation	MoKα (λ = 0.71073)
2Θ range for data collection/°	4.556 to 55.998
Index ranges	-12 ≤ h ≤ 12, -13 ≤ k ≤ 13, -21 ≤ l ≤ 21
Reflections collected	29340
Independent reflections	7327 [R_{int} = 0.0530, R_{sigma} = 0.0648]
Data/restraints/parameters	7327/0/397
Goodness-of-fit on F^2	1.034
Final R indexes [I>=2σ (I)]	R_1 = 0.0489, wR_2 = 0.1018
Final R indexes [all data]	R_1 = 0.0960, wR_2 = 0.1168
Largest diff. peak/hole / e Å^{-3}	0.24/-0.26

Crystals of **10** suitable for X-ray diffraction were grown by slow evaporation of a PhMe solution.

4.11. S3

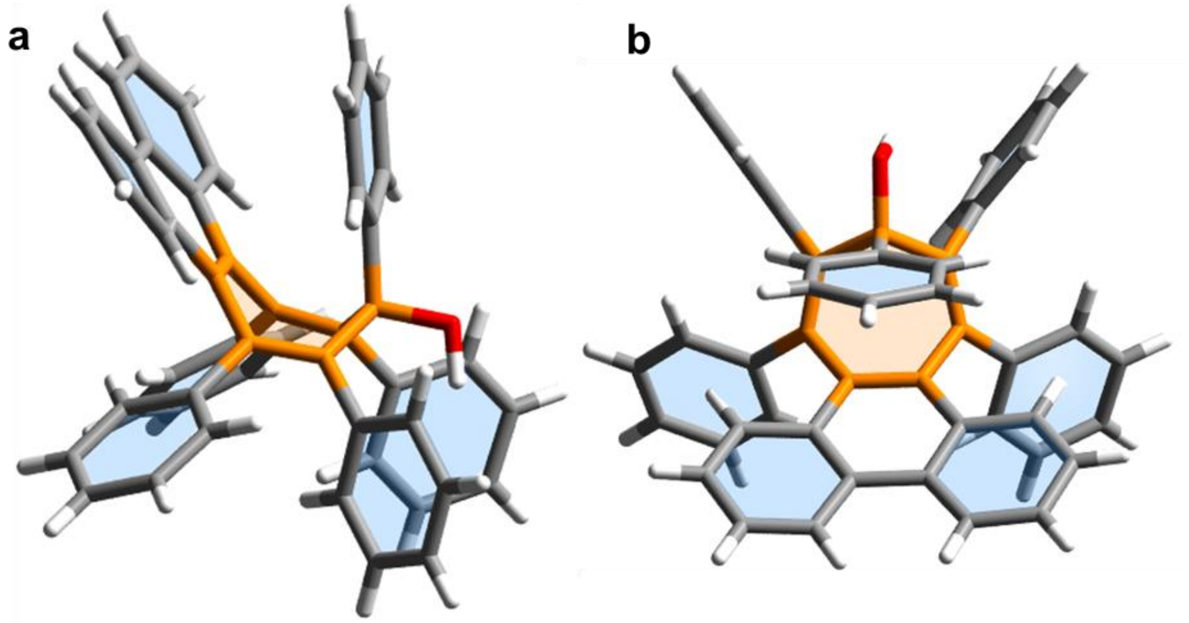

Fig. S64. Solid-state structure of **S3** viewed (**a**) side-on and (**b**) face-on to the seven-membered ring.

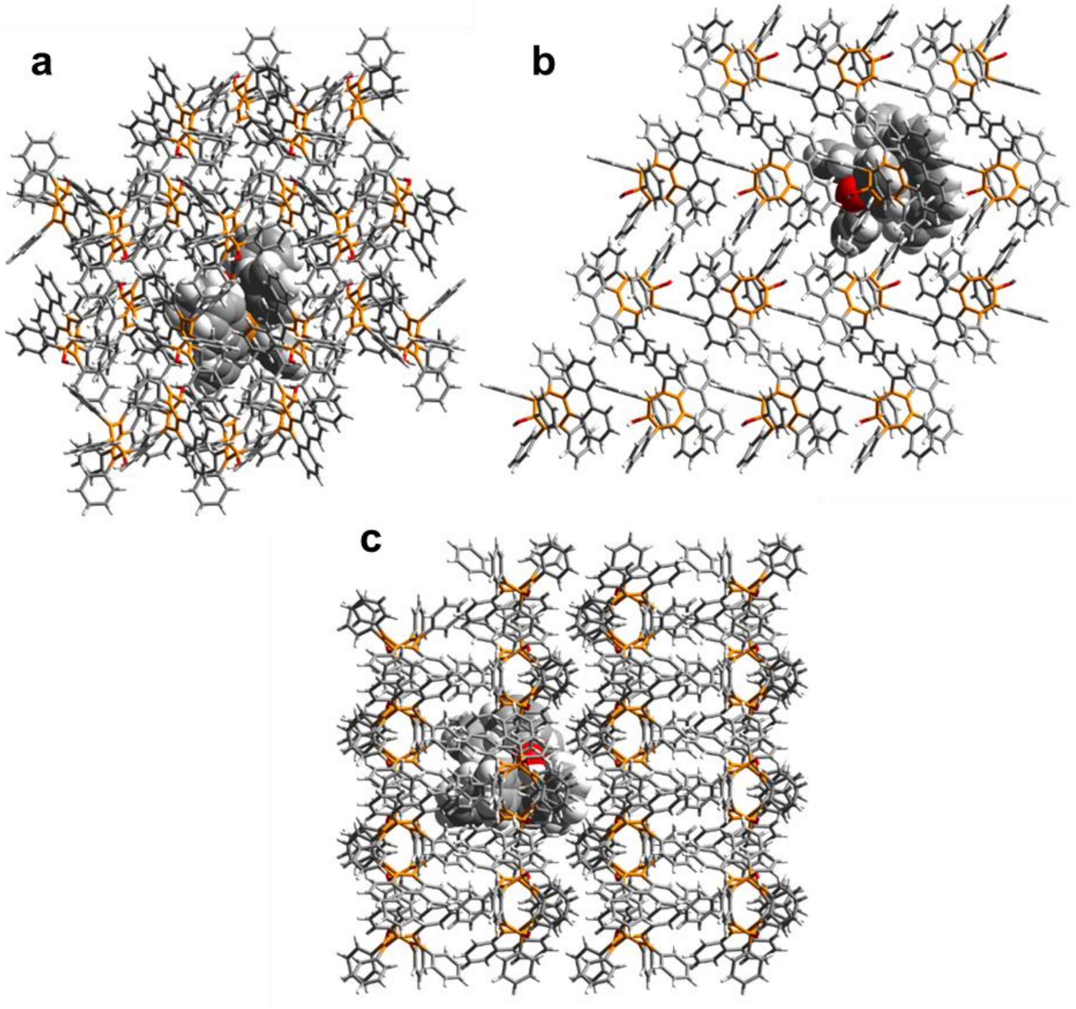

Fig. S65. Solid-state superstructure of **S3.** A central molecule (space filling representation) is shown embedded in a section of the lattice made up of 2×2×2 unit cells in order to illustrate the crystal packing. Projections are viewed along the crystallographic (**a**) *a*-, (**b**) *b*-, and (**c**) *c*-axes.

Table S12. Crystal data and structure refinement for **S3**.

	**S3**
CCDC Number	2141794
Empirical formula	$C_{49}H_{34}O$
Formula weight	638.76
Temperature/K	120.0
Crystal system	monoclinic
Space group	$P2_1/c$
a/Å	20.8941(13)
b/Å	9.4305(4)
c/Å	18.9622(10)
α/°	90
β/°	115.035(7)
γ/°	90
Volume/Å^3	3385.3(4)
Z	4
ρ_{calc}g/cm^3	1.253
μ/mm^{-1}	0.073
F(000)	1344.0
Crystal size/mm^3	0.54 × 0.23 × 0.22
Radiation	MoKα (λ = 0.71073)
2Θ range for data collection/°	4.872 to 57.994
Index ranges	-28 ≤ h ≤ 25, -9 ≤ k ≤ 12, -25 ≤ l ≤ 25
Reflections collected	34168
Independent reflections	8981 [R_{int} = 0.0717, R_{sigma} = 0.0781]
Data/restraints/parameters	8981/0/455
Goodness-of-fit on F^2	1.056
Final R indexes [I>=2σ (I)]	R_1 = 0.0611, wR_2 = 0.1299
Final R indexes [all data]	R_1 = 0.1037, wR_2 = 0.1519
Largest diff. peak/hole / e Å^{-3}	0.40/-0.27

Crystals of **S3** suitable for X-ray diffraction were grown by slow evaporation of a mixture of CH_2Cl_2–Et_2O.

4.12. 4·$SbCl_6$

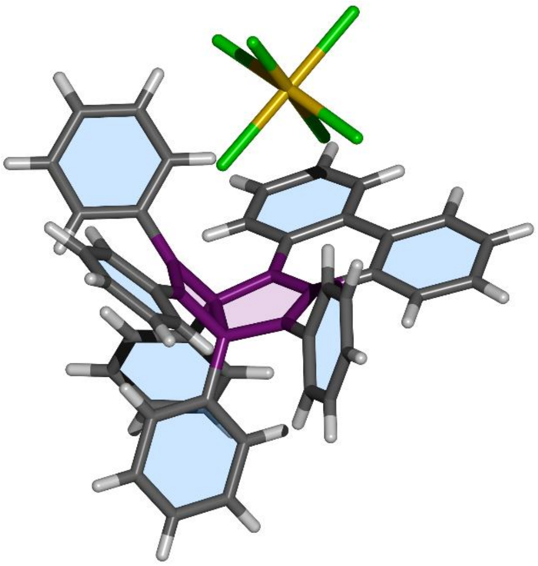

Fig. S66. Solid-state structure of **4**·$SbCl_6$ viewed side-on to the Dewar tropylium. The $SbCl_6$ counterion is included but the CH_2Cl_2 solvent has been omitted for clarity.

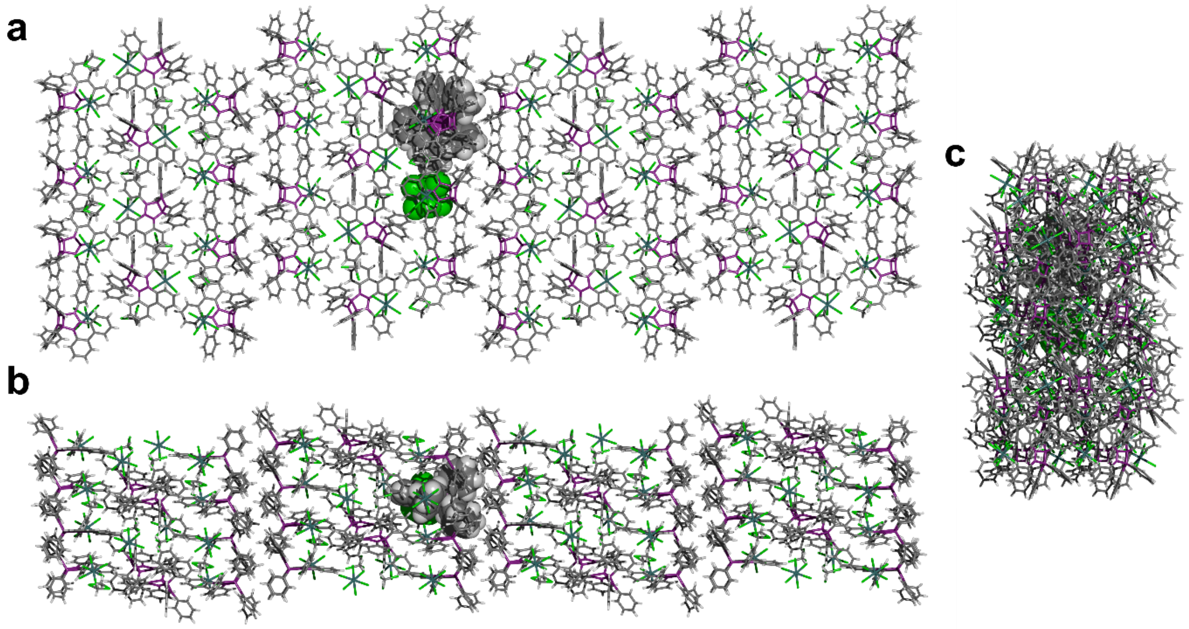

Fig. S67. Solid-state superstructure of **4**·$SbCl_6$**.** A central molecule (space filling representation) is shown embedded in a section of the lattice made up of 2×2×2 unit cells in order to illustrate the crystal packing. Projections are viewed along the crystallographic (**a**) *a*-, (**b**) *b*-, and (**c**) *c*-axes.

Table S13. Crystal data and structure refinement for **4**·$SbCl_6$.

	**4**·$SbCl_6$
CCDC Number	2141789
Empirical formula	$C_{49}H_{33}$ × $SbCl_6$ × 0.67 CH_2Cl_2
Formula weight	1012.82
Temperature/K	120
Crystal system	monoclinic
Space group	$P2_1/n$
a/Å	11.0794(6)
b/Å	19.5994(11)
c/Å	59.837(3)
α/°	90
β/°	93.9088(15)
γ/°	90
Volume/Å^3	12963.3(12)
Z	12
ρ_{calc}g/cm^3	1.557
μ/mm^{-1}	1.128
F(000)	6096.0
Crystal size/mm^3	0.323 × 0.133 × 0.029
Radiation	MoKα (λ = 0.71073)
2Θ range for data collection/°	4.092 to 50.054
Index ranges	-13 ≤ h ≤ 13, -23 ≤ k ≤ 23, -71 ≤ l ≤ 71
Reflections collected	187925
Independent reflections	22893 [R_{int} = 0.0843, R_{sigma} = 0.0545]
Data/restraints/parameters	22893/1467/1566
Goodness-of-fit on F^2	1.197
Final R indexes [I>=2σ (I)]	R_1 = 0.0724, wR_2 = 0.1408
Final R indexes [all data]	R_1 = 0.0958, wR_2 = 0.1483
Largest diff. peak/hole / e Å^{-3}	1.21/-1.50

Crystals of **4**·$SbCl_6$ suitable for X-ray diffraction were grown by slow evaporation of a mixture of CH_2Cl_2–hexane.

4.13. *anti*-11

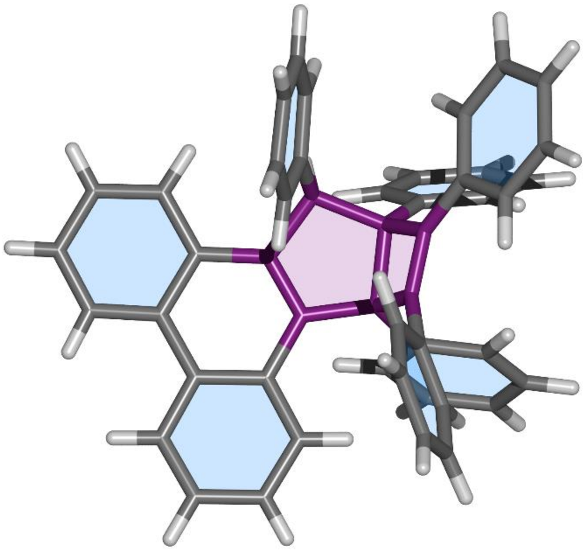

Fig. S68. Solid-state structure of *anti*-**11** viewed face-on to the bicyclo[3.2.0]heptadiene.

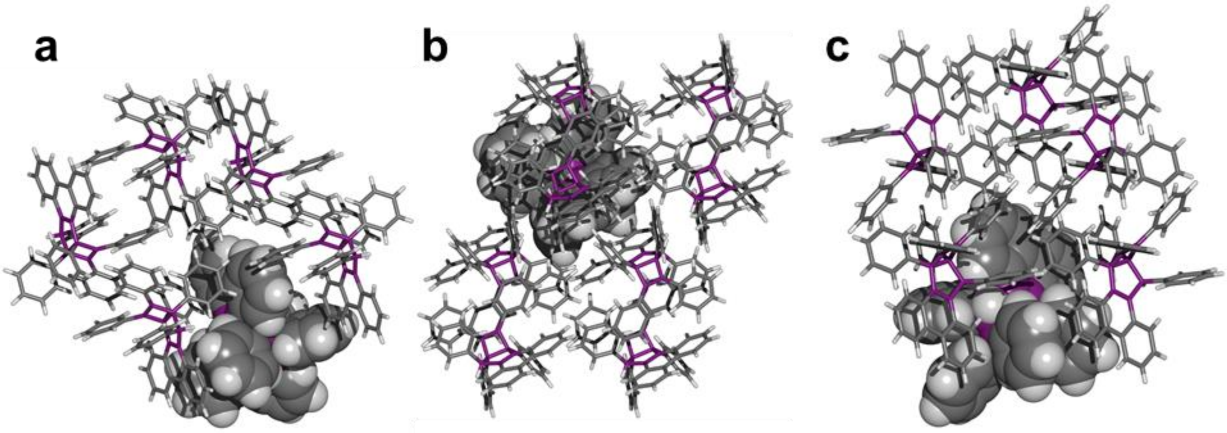

Fig. S69. Solid-state superstructure of *anti*-**11**. A central molecule (space filling representation) is shown embedded in a section of the lattice made up of 2×2×2 unit cells in order to illustrate the crystal packing. Projections are viewed along the crystallographic (**a**) *a*-, (**b**) *b*-, and (**c**) *c*-axes.

Table S14. Crystal data and structure refinement for *anti*-**11**.

	anti-**11**
Identification code	2182241
Empirical formula	$C_{49}H_{34}$
Formula weight	622.76
Temperature/K	120.00
Crystal system	triclinic
Space group	P-1
a/Å	11.4217(3)
b/Å	12.4870(4)
c/Å	13.5958(4)
α/°	104.5864(11)
β/°	105.3593(12)
γ/°	110.6846(10)
Volume/Å^3	1615.99(8)
Z	2
ρ_{calc}g/cm^3	1.280
μ/mm^{-1}	0.072
F(000)	656.0
Crystal size/mm^3	0.11 × 0.07 × 0.01
Radiation	Mo Kα (λ = 0.71073)
2Θ range for data collection/°	3.774 to 55
Index ranges	-14 ≤ h ≤ 14, -16 ≤ k ≤ 16, -17 ≤ l ≤ 17
Reflections collected	41725
Independent reflections	7421 [R_{int} = 0.0745, R_{sigma} = 0.0585]
Data/restraints/parameters	7421/0/578
Goodness-of-fit on F^2	1.058
Final R indexes [I>=2σ (I)]	R_1 = 0.0650, wR_2 = 0.1239
Final R indexes [all data]	R_1 = 0.0950, wR_2 = 0.1354
Largest diff. peak/hole / e Å^{-3}	0.29/-0.32

Crystals of *anti*-**11** suitable for X-ray diffraction were grown by slow evaporation of a mixture of CH_2Cl_2–hexane.

5. UV-Vis Absorption Spectroscopy

UV-Vis measurements were performed in anhydrous CH_2Cl_2. Spectra were measured in 10 mm path-length cuvette at room temperature. **2**·OTf and **4**·OTf were prepared *in situ* from their corresponding precursors: **S2**, and **S3** respectively. TfOH (10 µL) was added to a solution of the corresponding precursor (20 µM, 3 mL). The samples were sparged briefly with $N_{2\,(g)}$ to displace any TfOH·H_2O fumes formed when TfOH is in contact with air. The UV-vis spectrum of **1**·ICl_2 was measured by dissolving the compound in anhydrous CH_2Cl_2. The intensities were plotted as molar absorptivity ε defined by the formula:

$$\varepsilon = \frac{I}{c \cdot l}$$

where I is the measured intensity, c is the concentration of sample, and l is path length of the cuvette.

CH_2Cl_2 solutions of **1** and **2** are orange, whereas **4** is purple. These observations are consistent with the spectra shown in Figure S57, in which **1** and **2** absorb below 500 nm whereas the absorption of **4** extends to above 700 nm.

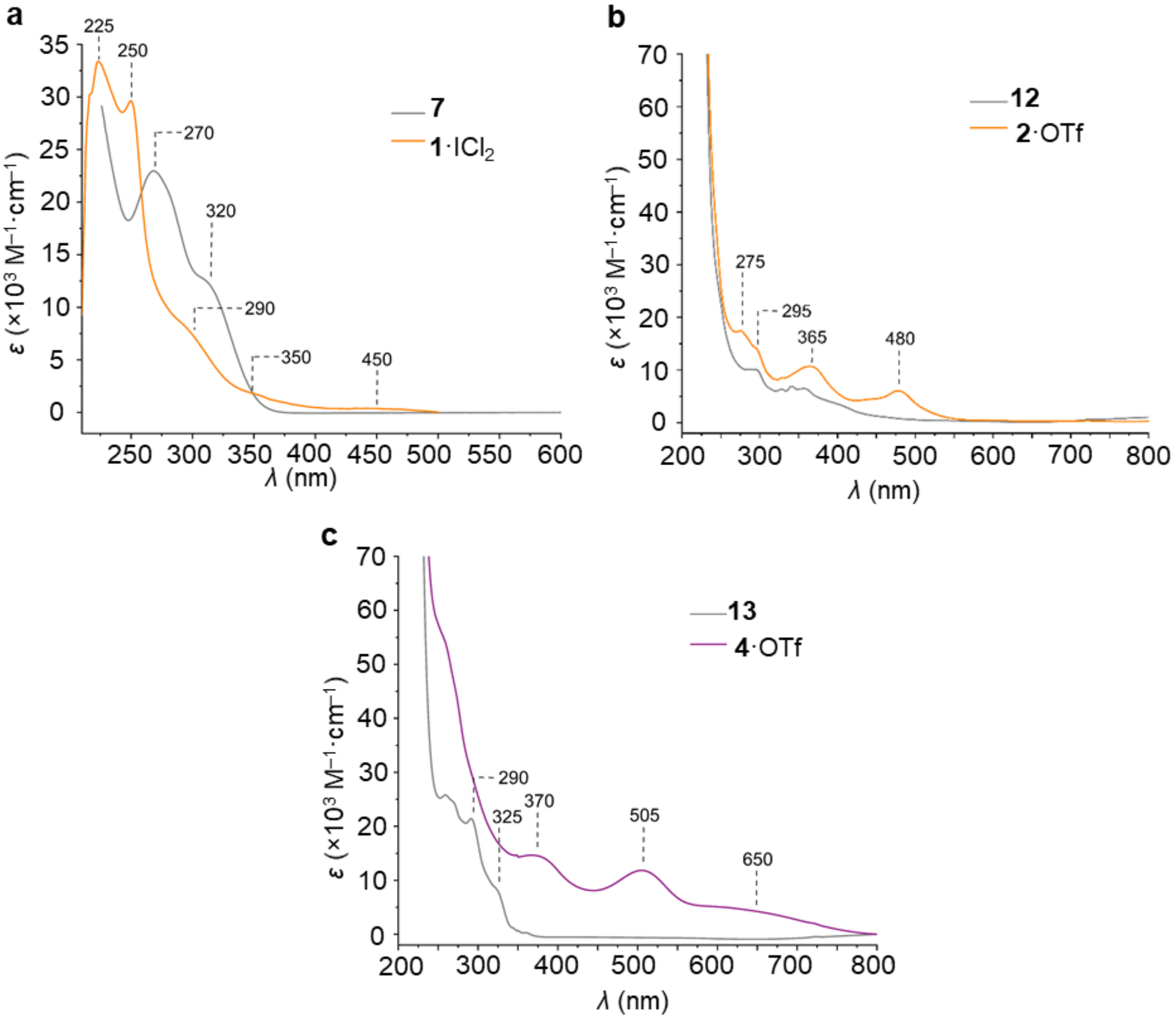

Fig. S70. UV-Vis absorption spectra of CH_2Cl_2 solutions a) **7** and **1**·ICl_2 b) **12** and **2**·OTf c) **13** and **4**·OTf. (*l* = 10 mm, *c* = 20 µM)

6. *In Silico* Modelling

6.1. General Methods

DFT calculations were carried out in Gaussian 16.[7] The minimum energy ground state (GS) and transition state (TS) geometries of all cations were optimized using the B3LYP functional and the D3 version of Grimme's dispersion correction with Becke–Johnson damping (GD3BJ) was included. Calculations were performed using the 6-31G(d) basis set using the integral equation formalism variant (IEFPCM) to approximate CH_2Cl_2 as the solvent. The tropylium and Dewar tropylium isomers of compound **4** (**4-TP** and **4** respectively) were also studied using a number of other functionals/basis sets (see section 6.2), and aromatic stabilization energies were also calculated using the M06-2X functional and 6-31G(d) basis set with the integral equation formalism variant (IEFPCM) to approximate CH_2Cl_2 as the solvent (see section 6.6).

Frequency calculations were carried out to confirm the lack of any negative vibrational frequencies for GS structures and to confirm their presence for TS structures (saddle points). Potential energy scans (PES) were carried out to predict the energetic penalty of twisting in **Naph**, fluoranthene, and **2**. Intrinsic reaction coordinate (IRC) calculations were performed to confirm that TS geometries identified lie on a potential surface linking the tropylium (**TP**), Möbius tropylium (**MT**), and Dewar tropylium (**DT**) GS structures. Aromatic stabilization energies (ASEs) were calculated using an isodesmic reaction, following the isomerization stabilization energy (ISE) method. The aromatic character of the **TP**-isomers of the cations synthesized in this work was assessed using nucleus-independent chemical shifts (NICS)[8], anisotropy of the induced current density (ACID) plots[9], and the electron density of the delocalized bonds (EDDB) index. DFT-optimized geometries were used for all aromaticity probes.

6.2. Basis Set and Functional Screen

We screened basis sets and functionals to find the combination that most accurately reproduces our experimental results, using the ~96:4 ratio of **DT**- and **TP**-derived products obtained in our hydride trapping experiment as the benchmark. The results are summarized below.

Table S15. Predicted free energy differences between the tropylium and Dewar tropylium isomers of compound **4** (**4-TP** and **4** respectively), using a range of functionals and basis sets. The integral equation formalism variant (IEFPCM) was used to approximate CH_2Cl_2 as the solvent.

Functional	Basis Set	Isomer	*G* / Hartrees	ΔG / kJ·mol^{-1}	**4**:**4-TP** [a]
ωB97X-D	6-31G(d)	**4-TP**	-1885.692947	–33.0	—
		**4**	-1885.705513		
	6-311G(d)	**4-TP**	-1886.036435	–29.1	—
		**4**	-1886.047512		
	6-311G(d,p)	**4-TP**	-1886.090614	–29.5	—
		**4**	-1886.101846		
M06-2X	6-31G(d)	**4-TP**	-1885.527902	–21.0	99.98:0.02
		**4**	-1885.5359		
	6-311G(d)	**4-TP**	-1885.925552	–14.6	99.7:0.03
		**4**	-1885.931109		
	6-311G(d,p)	**4-TP**	-1885.970401	–15.1	99.8:0.02
		**4**	-1885.976134		
CAM-B3LYP	6-31G(d)	**4-TP**	-1885.186164	–23.3	99.99:0.01
		**4**	-1885.19504		
	6-311G(d)	**4-TP**	-1885.564101	–16.4	99.9:0.01
		**4**	-1885.570339		
	6-311G(d,p)	**4-TP**	-1885.619595	–17.2	99.9:0.01
		**4**	-1885.62614		
B3LYP (GD3BJ)	6-31G(d)	**4-TP**	-1886.558425	–5.31	90:10
		**4**	-1886.560446		
	6-311G(d)	**4-TP**	-1886.928045	+1.25	38:62
		**4**	-1886.927568		
	6-311G(d,p)	**4-TP**	-1886.985344	+1.24	38:62
		**4**	-1886.98487		

[a] As predicted by the Boltzmann distribution of isomers. For details, see section 6.10.

The B3LYP functional with a GD3BJ empirical dispersion correction and 6-31G(d) basis set using CH_2Cl_2 as the solvent gave the closest isomeric distribution.

6.3. Intrinsic Reaction Coordinate (IRC) Calculations

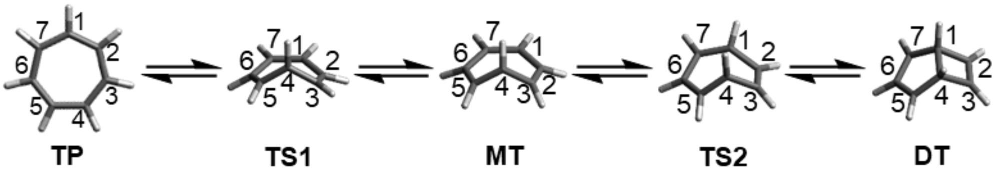

Fig. S71. Rearrangement of **TP**-$(CH)_7^+$ to the **DT** isomer *via* the **MT** intermediate.

Table S16. Relative energies (G_{rel} / kJ·mol^{-1}), d(C_1–C_4) / Å (internuclear distance between C_1 and C_4), and H_4–C_4–C_5–H_5 dihedral angles (Φ / °) of the key structures in the isomerism of **TP**-$(CH)_7^+$.

Structure	d(C_1–C_4) / Å	Φ / °	G_{rel} / kJ·mol^{-1} [a]
**TP**	3.14	0.00	0
**TS1**	2.65	113	302
**MT**	2.55	152	249
**TS2**	2.07	119	289
**DT**	1.55	52.8	219

[a] G_{rel} refers to the energy of each species relative to the energy of **TP-$C_7H_7^+$** (the lowest-energy structure).

Table S17. IRC Calculation for the rearrangement of the **DT** isomer of $(CH)_7^+$ to the **TP** isomer *via* a **MT** intermediate, showing the change in energy (E) as a function of d(C_1–C_4) / Å: and Φ / °.

Coordinate	d(C_1–C_4) / Å	Φ / °	E / Hartree	E / kJ·mol^{-1}	E_{rel} / kJ·mol^{-1} [a]
5.43658	1.54887463	53.20526639	-270.68535	-710684.3876	227.4037416
5.24923	1.54900413	54.67339977	-270.685305	-710684.2671	227.5242757
5.06167	1.54938376	56.17483063	-270.685195	-710683.979	227.8124007
4.8741	1.55003268	57.71142802	-270.685021	-710683.5234	228.2679643
4.68654	1.55095629	59.28161903	-270.684784	-710682.8994	228.8919695
4.49898	1.55216766	60.86767219	-270.684481	-710682.1037	229.6876746
4.31143	1.55368369	62.46900673	-270.68411	-710681.131	230.6603278
4.12389	1.55553397	64.07678141	-270.68367	-710679.9757	231.8156423
3.93637	1.55777253	65.68525878	-270.683158	-710678.6301	233.1612373
3.74886	1.56052861	67.28979810	-270.682569	-710677.0851	234.7062312
3.56138	1.56410036	68.91035564	-270.681899	-710675.3268	236.4645548
3.37396	1.56913063	70.59871246	-270.68114	-710673.3328	238.4585433
3.18668	1.57677668	72.43178201	-270.680276	-710671.0638	240.7275371
2.99957	1.58857722	74.48972007	-270.679277	-710668.4419	243.3494559
2.8125	1.60559948	76.78599549	-270.678098	-710665.3456	246.4458
2.62529	1.62756457	79.27120932	-270.676695	-710661.6625	250.128875
2.43793	1.65325202	81.87083506	-270.675057	-710657.3632	254.4281549
2.25048	1.68146341	84.54850224	-270.673216	-710652.5286	259.2627637
2.06297	1.71132515	87.28963521	-270.671232	-710647.3201	264.4712306
1.87545	1.74228235	90.08474503	-270.669182	-710641.9386	269.8527836
1.68791	1.77398411	92.91788949	-270.667144	-710636.5867	275.2046685
1.50036	1.8061947	95.78090221	-270.665185	-710631.4439	280.3474716
1.31281	1.83873332	98.66935933	-270.663363	-710626.6597	285.1316393
1.12526	1.87145955	101.58428469	-270.661723	-710622.3541	289.4372939
0.93771	1.90427539	104.53116306	-270.660301	-710618.6206	293.1707917
0.75016	1.93710311	107.49280474	-270.659124	-710615.5307	296.2606954
0.56262	1.96986824	110.45754753	-270.658212	-710613.1347	298.6566348
0.37508	2.00245236	113.42896746	-270.657573	-710611.4579	300.3334182
0.18756	2.0347103	116.37518556	-270.657205	-710610.4911	301.3002087
0	2.06583691	119.25053375	-270.657088	-710610.1844	301.60698
-0.18756	2.09697072	122.12356474	-270.657192	-710610.4564	301.3349913
-0.37507	2.12671961	124.89944386	-270.657489	-710611.2373	300.554039
-0.5626	2.15560633	127.63326464	-270.657957	-710612.4673	299.324008
-0.75013	2.18355018	130.27589566	-270.658579	-710614.098	297.6933073

-0.93767	2.21052017	132.82163089	-270.659336	-710616.0855	295.7058406
-1.12521	2.23657313	135.25642205	-270.660211	-710618.3841	293.4072232
-1.31276	2.26166541	137.54806823	-270.661188	-710620.9497	290.8416634
-1.5003	2.28582424	139.67969131	-270.66225	-710623.7377	288.0536528
-1.68784	2.30908279	141.64183481	-270.66338	-710626.7041	285.0872316
-1.87539	2.331481	143.42148129	-270.664559	-710629.8005	281.9908141
-2.06294	2.35313491	145.02142910	-270.665766	-710632.969	278.822379
-2.25048	2.37409373	146.44115137	-270.666977	-710636.1472	275.6441745
-2.43803	2.39441805	147.69081771	-270.668166	-710639.2705	272.5208482
-2.62558	2.41412237	148.77480495	-270.669312	-710642.2786	269.5127919
-2.81312	2.43320935	149.68862421	-270.670392	-710645.1146	266.6767767
-3.00067	2.45169208	150.42833907	-270.671386	-710647.7232	264.0681402
-3.1882	2.46960033	150.99838339	-270.672272	-710650.0504	261.7409495
-3.37573	2.48691342	151.40133727	-270.673032	-710652.0468	259.7445745
-3.56323	2.50352626	151.63916839	-270.673651	-710653.6716	258.1197497
-3.75068	2.51920137	151.72277104	-270.674117	-710654.8951	256.8962378
-3.93798	2.53342281	151.67561861	-270.674425	-710655.7024	256.0889228
-4.12443	2.54487658	151.55314418	-270.674577	-710656.1009	255.6904612
-3.38615	2.54754123	151.58405638	-270.67461	-710656.1885	255.6028509
-3.27384	2.54502689	152.10897250	-270.674569	-710656.0809	255.7104176
-3.16108	2.541865	152.65659251	-270.674479	-710655.8441	255.9472981
-3.04822	2.53873691	153.19562112	-270.674335	-710655.4678	256.3235506
-2.93533	2.5360063	153.71180979	-270.674137	-710654.9455	256.8458702
-2.82243	2.53391433	154.19455757	-270.67388	-710654.2716	257.5197074
-2.70954	2.53262992	154.62643024	-270.673563	-710653.4407	258.3506939
-2.59665	2.53227016	154.98699816	-270.673184	-710652.4456	259.3457295
-2.48377	2.53289746	155.24836734	-270.67274	-710651.2779	260.5134627
-2.37089	2.5345149	155.38079966	-270.672225	-710649.9271	261.8642089
-2.258	2.53707288	155.35395100	-270.671637	-710648.3823	263.4090373
-2.14512	2.54047515	155.13342133	-270.67097	-710646.6325	265.1588333
-2.03222	2.54460434	154.69630056	-270.670222	-710644.6689	267.1224894
-1.91932	2.54933423	154.02648291	-270.66939	-710642.484	269.307399
-1.80642	2.55452539	153.10929188	-270.668471	-710640.0715	271.7198213
-1.69351	2.5600486	151.94200311	-270.667466	-710637.4309	274.3604941
-1.58059	2.56580507	150.53403971	-270.666376	-710634.5697	277.2216773
-1.46767	2.57170556	148.88902594	-270.665206	-710631.4976	280.2937171
-1.35475	2.57766841	147.02043145	-270.663962	-710628.2322	283.5591722
-1.24182	2.58363277	144.94041231	-270.662655	-710624.801	286.9903279
-1.12889	2.58957657	142.67188202	-270.6613	-710621.244	290.5473868
-1.01595	2.59549478	140.23226785	-270.659916	-710617.6108	294.1805852
-0.90302	2.60137557	137.63842372	-270.658524	-710613.9547	297.836657
-0.79008	2.60721839	134.90007453	-270.657144	-710610.3327	301.4586078
-0.67714	2.61301397	132.02142709	-270.655804	-710606.8143	304.9770928
-0.56421	2.61876032	129.00844973	-270.654539	-710603.491	308.3003614
-0.45127	2.6245082	125.89065564	-270.653393	-710600.4823	311.3090505
-0.33834	2.6302312	122.68886513	-270.652415	-710597.9146	313.8767292
-0.22541	2.6358637	119.39127681	-270.651649	-710595.9047	315.8866649
-0.11258	2.6414673	116.00482482	-270.651148	-710594.589	317.2023896

0	2.6471038	112.53181934	-270.650968	-710594.1174	317.6740003
0.11234	2.65274972	109.07129384	-270.651162	-710594.6254	317.1659477
0.22522	2.65840043	105.53998346	-270.651779	-710596.247	315.5443155
0.33814	2.66405648	101.95264699	-270.652857	-710599.0768	312.7145043
0.45106	2.66971138	98.32199436	-270.654405	-710603.1391	308.6522966
0.56399	2.6754306	94.67333597	-270.656386	-710608.3423	303.4490833
0.67692	2.68131101	91.04127975	-270.658733	-710614.5022	297.2891326
0.78985	2.68738033	87.44248659	-270.661367	-710621.4178	290.3735735
0.90279	2.69362092	83.87914299	-270.664223	-710628.9184	282.8729479
1.01573	2.70003938	80.36206246	-270.667247	-710636.8561	274.9352475
1.12867	2.70663271	76.90413031	-270.670388	-710645.1041	266.6872209
1.24161	2.71341295	73.51731021	-270.673608	-710653.5584	258.2329192
1.35456	2.72037011	70.20618931	-270.676878	-710662.1436	249.6477942
1.4675	2.72748726	66.97191035	-270.680176	-710670.8011	240.990263
1.58045	2.73475842	63.82040768	-270.683481	-710679.48	232.3113945
1.6934	2.74217553	60.75379985	-270.686779	-710688.1379	223.6534328
1.80635	2.74973484	57.77699711	-270.690054	-710696.7378	215.0535682
1.9193	2.75743056	54.89231654	-270.693296	-710705.2484	206.5429548
2.03225	2.76525259	52.09989052	-270.696495	-710713.6469	198.1444949
2.14521	2.77319351	49.39876459	-270.699643	-710721.9139	189.8774071
2.25816	2.7812568	46.79184441	-270.702735	-710730.0301	181.7612567
2.37111	2.78943394	44.27910627	-270.705762	-710737.9782	173.8131593
2.48407	2.7977191	41.86158183	-270.70872	-710745.7432	166.0481407
2.59702	2.80610735	39.53830360	-270.711603	-710753.3126	158.4787796
2.70997	2.81459586	37.31143239	-270.714407	-710760.6746	151.1167988
2.82293	2.8231891	35.18429951	-270.717127	-710767.8182	143.9731179
2.93588	2.83188745	33.15878876	-270.719762	-710774.7353	137.0560123
3.04883	2.84068584	31.23609162	-270.722308	-710781.421	130.3703866
3.16179	2.84957688	29.41309088	-270.724766	-710787.8744	123.9169417
3.27474	2.85854353	27.68370799	-270.727137	-710794.0972	117.6941628
3.3877	2.86757418	26.04543905	-270.729419	-710800.0897	111.7016743
3.50065	2.87666286	24.49882464	-270.731613	-710805.8506	105.9407287
3.6136	2.88580489	23.04617436	-270.733719	-710811.3785	100.4128724
3.72656	2.89499157	21.68735396	-270.735736	-710816.6738	95.1175303
3.83951	2.90420848	20.42022630	-270.737665	-710821.7396	90.05171994
3.95246	2.91343782	19.24083407	-270.739509	-710826.5804	85.21092806
4.06541	2.92266697	18.14569152	-270.741269	-710831.2016	80.58978814
4.17836	2.93187134	17.13073573	-270.742948	-710835.6096	76.18173379
4.29131	2.94103063	16.18865320	-270.744549	-710839.8124	71.97893841
4.40426	2.95012328	15.31403119	-270.746074	-710843.8182	67.97313692
4.51721	2.95912684	14.50237382	-270.747528	-710847.6355	64.15580958
4.63016	2.96802346	13.74917298	-270.748914	-710851.2727	60.51861253
4.74311	2.97679684	13.05186997	-270.750234	-710854.7382	57.05314157
4.85606	2.98542517	12.40574344	-270.751491	-710858.0406	53.75076667
4.96901	2.99388858	11.80850558	-270.75269	-710861.1884	50.60291031
5.08196	3.00217101	11.25802300	-270.753834	-710864.1902	47.60117879
5.19491	3.0102513	10.75167036	-270.754924	-710867.0538	44.7375827
5.30786	3.01811144	10.28400383	-270.755965	-710869.7869	42.0044792

5.42081	3.02573209	9.84799912	-270.756959	-710872.397	39.39432263
5.53376	3.03310247	9.43798211	-270.75791	-710874.8915	36.89987183
5.64671	3.04020972	9.04946265	-270.758818	-710877.2766	34.51470748
5.75966	3.04705193	8.67932501	-270.759687	-710879.5582	32.23312172
5.87262	3.05362197	8.32435500	-270.760519	-710881.7414	30.04998697
5.98557	3.05991762	7.98180669	-270.761314	-710883.8305	27.96090025
6.09853	3.06594288	7.64968976	-270.762075	-710885.8292	25.96214648
6.21148	3.07169606	7.32809119	-270.762804	-710887.7409	24.05042016
6.32444	3.07717916	7.01667435	-270.7635	-710889.5683	22.22308791
6.4374	3.08239574	6.71447188	-270.764164	-710891.3135	20.47786818
6.55036	3.08734599	6.42057806	-270.764799	-710892.9786	18.81278133
6.66332	3.09203348	6.13400306	-270.765403	-710894.5653	17.22603415
6.77628	3.09645604	5.85274409	-270.765978	-710896.0754	15.71595945
6.88924	3.10061621	5.57532128	-270.766525	-710897.5104	14.28096354
7.0022	3.10451848	5.30023528	-270.767043	-710898.8718	12.91953677
7.11516	3.10816431	5.02595613	-270.767534	-710900.1611	11.63023249
7.22812	3.11155879	4.75288354	-270.767998	-710901.3797	10.41165129
7.34108	3.11470695	4.48200864	-270.768436	-710902.5289	9.262404306
7.45404	3.11761787	4.21252971	-270.768848	-710903.6101	8.181210279
7.567	3.12029572	3.94460947	-270.769234	-710904.6242	7.167192294
7.67996	3.12274951	3.67775547	-270.769595	-710905.5716	6.219754364
7.79293	3.12498563	3.41411963	-270.769931	-710906.4529	5.338473783
7.90589	3.12701186	3.15434344	-270.770241	-710907.2681	4.523219277
8.01885	3.12883979	2.89785566	-270.770526	-710908.0172	3.77418513
8.13181	3.13047869	2.64369638	-270.770786	-710908.6999	3.091457987
8.24478	3.13194088	2.39074374	-270.771021	-710909.3162	2.475129739
8.35774	3.13323399	2.13739018	-270.771231	-710909.8663	1.925084863
8.4707	3.1343685	1.88349708	-270.771415	-710910.3504	1.440976794
8.58367	3.13534656	1.62774910	-270.771575	-710910.7689	1.022456342
8.69663	3.13617373	1.36988649	-270.771709	-710911.1221	0.669211069
8.80959	3.13684946	1.10903124	-270.771819	-710911.4102	0.381172715
8.92256	3.13737309	0.84530537	-270.771904	-710911.6332	0.15818375
9.03552	3.13774175	0.57886811	-270.771964	-710911.7914	0

[a] E_{rel} refers to the energy of each species relative to the energy of **TP**-$(CH)_7^+$ (the lowest-energy structure).

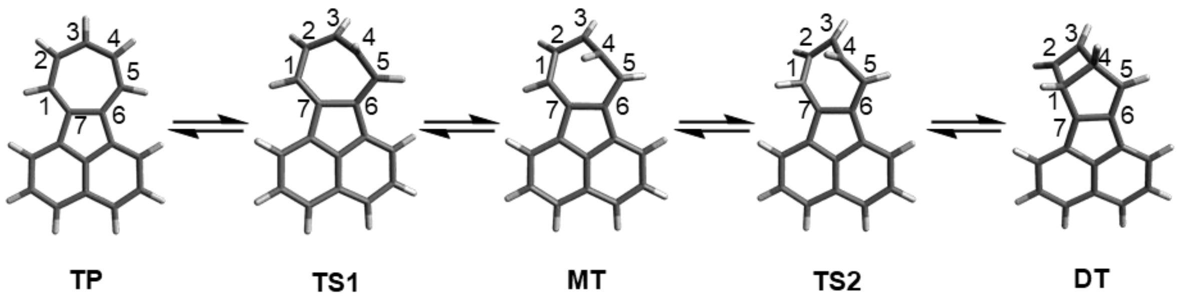

Fig. S72. Rearrangement of the **TP** isomer of acenaphthyltropylium (**Naph**) to the **DT** isomer *via* the **MT** intermediate.

Table S18. Relative energies (G_{rel} / kJ·mol^{-1}), d(C_1–C_4) / Å (internuclear distance between C_1 and C_4), and H_4–C_4–C_5–H_5 dihedral angles (Φ / °) of the key structures in the isomerism of **TP-Naph.**

Structure	d(C_1–C_4) / Å	Φ / °	G_{rel} / kJ·mol^{-1} [a]
**TP**	3.15	0.00	0
**TS1**	2.80	64.4	311
**MT**	2.58	153	259
**TS2**	2.10	117	316
**DT**	1.57	53.9	220

[a] G_{rel} refers to the energy of each species relative to the energy of **TP-$C_7H_7^+$** (the lowest-energy structure).

Table S19. IRC Calculation for the rearrangement of the **DT** isomer of **Naph** to the **TP** isomer *via* a **MT** intermediate, showing the change in energy (E) as a function of d(C_1–C_4): the bond distance between C_1 and C_4 and the H_4–C_4–C_5–H_5 dihedral angle (Φ / °). E_{rel} refers to the energy of each species relative to the energy of **TP- Naph** (the lowest-energy structure).

Coordinate	d(C_1–C_4) / Å	Φ / °	E / Hartree	E / kJ·mol^{-1}	E_{rel} / kJ·mol^{-1} [a]
-9.27357	1.57328596	60.71134970	-654.2493937	-1717731.78	227.6670688
-9.10176	1.573304803	60.69336773	-654.2493695	-1717731.72	227.7304877
-8.92994	1.573328918	60.67538807	-654.2493387	-1717731.64	227.8113164
-8.75813	1.573358331	60.65799525	-654.2493013	-1717731.54	227.9094654
-8.58631	1.573391101	60.64162618	-654.2492574	-1717731.43	228.0247564
-8.4145	1.573428979	60.62634172	-654.2492071	-1717731.29	228.1569556
-8.24268	1.573473586	60.61140598	-654.2491503	-1717731.14	228.3059291
-8.07087	1.573524656	60.59645420	-654.2490872	-1717730.98	228.4717688
-7.89905	1.573581359	60.58023502	-654.2490175	-1717730.8	228.6547871
-7.72724	1.573642408	60.56191917	-654.2489411	-1717730.59	228.8554173
-7.55542	1.573710093	60.54187066	-654.2488578	-1717730.38	229.0740979
-7.38361	1.573783959	60.51919801	-654.2487674	-1717730.14	229.3113092
-7.21179	1.573865797	60.49402951	-654.2486698	-1717729.88	229.5675895
-7.03998	1.573955966	60.46600057	-654.2485647	-1717729.61	229.8434297
-6.86816	1.574057329	60.43404177	-654.248452	-1717729.31	230.1393157
-6.69635	1.574170269	60.39826524	-654.2483315	-1717728.99	230.4557987
-6.52454	1.574293411	60.35767846	-654.2482029	-1717728.66	230.7935273
-6.35274	1.574432014	60.31162632	-654.2480658	-1717728.3	231.1533652
-6.18094	1.574590087	60.26027083	-654.2479199	-1717727.91	231.5363626
-6.00915	1.574769847	60.20232852	-654.2477647	-1717727.51	231.9438665
-5.83736	1.574976493	60.13705533	-654.2475995	-1717727.07	232.3776621
-5.66559	1.57521245	60.06409157	-654.2474234	-1717726.61	232.8400022
-5.49382	1.575475022	59.98146049	-654.2472353	-1717726.12	233.3339375
-5.32206	1.575767097	59.88825051	-654.2470336	-1717725.59	233.8635271
-5.15031	1.576083435	59.78481813	-654.2468164	-1717725.02	234.4337305
-4.97855	1.57642925	59.67542338	-654.2465815	-1717724.4	235.0503187
-4.8068	1.576805554	59.56541781	-654.2463266	-1717723.73	235.719619
-4.63505	1.57722388	59.46154309	-654.246049	-1717723	236.448408
-4.4633	1.577691317	59.37127634	-654.245746	-1717722.21	237.2440447
-4.29157	1.578218848	59.30323155	-654.2454142	-1717721.34	238.1150911

-4.11986	1.578821668	59.27161338	-654.2450495	-1717720.38	239.072695
-3.9482	1.579553194	59.29392693	-654.2446457	-1717719.32	240.1329533
-3.77661	1.580568615	59.39271882	-654.2441933	-1717718.13	241.3206717
-3.60511	1.582348901	59.60080686	-654.2436776	-1717716.78	242.6745134
-3.43376	1.586054928	59.96121050	-654.2430752	-1717715.19	244.2561409
-3.26266	1.593728364	60.53785766	-654.2423446	-1717713.28	246.174318
-3.09162	1.607148063	61.38971006	-654.2414153	-1717710.84	248.6142451
-2.92029	1.626043489	62.52540105	-654.2402059	-1717707.66	251.7896167
-2.74871	1.64883182	63.91493089	-654.2386703	-1717703.63	255.8211796
-2.577	1.674138999	65.52798285	-654.2368137	-1717698.75	260.6958483
-2.40524	1.701033686	67.33543105	-654.2346752	-1717693.14	266.310291
-2.23345	1.728888502	69.31358823	-654.2323135	-1717686.94	272.5110919
-2.06165	1.757319022	71.44595992	-654.2297991	-1717680.34	279.1125782
-1.88985	1.78609961	73.72129106	-654.2272079	-1717673.53	285.9159103
-1.71804	1.81510891	76.13620625	-654.224614	-1717666.72	292.7261265
-1.54623	1.844262613	78.68175978	-654.2220857	-1717660.09	299.3641965
-1.37443	1.873488544	81.34728763	-654.2196836	-1717653.78	305.6707919
-1.20262	1.902721791	84.11073222	-654.2174627	-1717647.95	311.501778
-1.03081	1.93189851	86.95352566	-654.2154718	-1717642.72	316.7288177
-0.859	1.960944492	89.85849698	-654.2137533	-1717638.21	321.2409127
-0.6872	1.989789341	92.82253823	-654.2123415	-1717634.5	324.947528
-0.5154	2.01839912	95.84391105	-654.2112582	-1717631.66	327.7917689
-0.3436	2.046682633	98.93440883	-654.2105065	-1717629.68	329.7653284
-0.17182	2.074566275	102.04807624	-654.2100725	-1717628.55	330.904819
0	2.101444877	105.19360065	-654.2099343	-1717628.18	331.2676815
0.17182	2.128326899	108.31953681	-654.210065	-1717628.53	330.92461
0.3436	2.154097523	111.40028248	-654.2104422	-1717629.52	329.9340797
0.51538	2.179237663	114.42326156	-654.2110445	-1717631.1	328.3527122
0.68718	2.203728471	117.35288906	-654.2118497	-1717633.21	326.2387436
0.85898	2.227626827	120.21524485	-654.2128318	-1717635.79	323.6601377
1.03078	2.251004204	123.02928919	-654.2139611	-1717638.75	320.6953128
1.20258	2.273806464	125.77213816	-654.2152089	-1717642.03	317.4192638
1.37438	2.296002877	128.41685171	-654.2165484	-1717645.55	313.9023488
1.54618	2.317560825	130.94594000	-654.2179544	-1717649.24	310.2109089
1.71798	2.338523139	133.36743052	-654.2193994	-1717653.03	306.4170142
1.88978	2.358931833	135.68611314	-654.220856	-1717656.86	302.5927949
2.06158	2.378768746	137.89785764	-654.2223005	-1717660.65	298.8003153
2.23338	2.397992289	139.99685890	-654.223715	-1717664.36	295.0865219
2.40518	2.416571236	141.98301621	-654.2250858	-1717667.96	291.4875101
2.57698	2.434533755	143.85563129	-654.2263977	-1717671.41	288.0431193
2.74878	2.451890226	145.61290866	-654.227635	-1717674.66	284.7945645
2.92058	2.468648086	147.24676915	-654.2287822	-1717677.67	281.7824229
3.09237	2.484851356	148.75141439	-654.229824	-1717680.4	279.0471665
3.26416	2.500479082	150.11597856	-654.2307488	-1717682.83	276.6192301
3.43594	2.515437535	151.33046125	-654.2315494	-1717684.93	274.5173257
3.6077	2.529633028	152.39085888	-654.2322219	-1717686.7	272.7516559

3.77945	2.542921837	153.29765300	-654.2327658	-1717688.13	271.3235913
3.95115	2.555118322	154.05368797	-654.2331829	-1717689.22	270.2283509
4.12273	2.565986747	154.65171761	-654.2334766	-1717689.99	269.4572258
4.29385	2.575032627	155.06207202	-654.2336544	-1717690.46	268.9905694
4.46104	2.581036482	155.20450086	-654.2337349	-1717690.67	268.7792482
4.62531	2.582611793	155.09820843	-654.233765	-1717690.75	268.7001104
-5.81302	2.582693738	154.98237921	-654.2337721	-1717690.77	268.6815454
-5.69899	2.582529307	154.92887209	-654.233756	-1717690.73	268.7238186
-5.58497	2.582351958	154.87954608	-654.2337354	-1717690.67	268.7779906
-5.47094	2.582171792	154.83632017	-654.2337102	-1717690.61	268.8441006
-5.35691	2.581997003	154.80078022	-654.2336804	-1717690.53	268.9221699
-5.24288	2.581837804	154.77521097	-654.2336461	-1717690.44	269.012193
-5.12886	2.581701577	154.76265349	-654.2336073	-1717690.34	269.1141885
-5.01484	2.581602102	154.76680921	-654.2335639	-1717690.22	269.2282507
-4.90082	2.581551065	154.79222947	-654.2335157	-1717690.1	269.3546055
-4.78681	2.581567749	154.84385794	-654.2334628	-1717689.96	269.4936493
-4.67281	2.581677635	154.92784865	-654.2334047	-1717689.8	269.6460307
-4.55883	2.581911739	155.05046849	-654.2333412	-1717689.64	269.8128025
-4.44485	2.582304942	155.21937773	-654.2332716	-1717689.45	269.9955977
-4.3309	2.582902651	155.44132199	-654.233195	-1717689.25	270.1967504
-4.21695	2.583753655	155.72049092	-654.2331102	-1717689.03	270.4194217
-4.10302	2.58490871	156.05764539	-654.2330156	-1717688.78	270.6676784
-3.98908	2.58640694	156.45153682	-654.2329095	-1717688.5	270.9463753
-3.87514	2.58828007	156.89322913	-654.2327898	-1717688.19	271.2606581
-3.76119	2.590547534	157.37084130	-654.2326545	-1717687.83	271.6157281
-3.64722	2.593223707	157.86986013	-654.2325019	-1717687.43	272.0165396
-3.53324	2.596314283	158.38088761	-654.2323301	-1717686.98	272.4675164
-3.41925	2.599825098	158.89905480	-654.2321378	-1717686.48	272.9723134
-3.30526	2.603763296	159.41855221	-654.2319241	-1717685.92	273.5335036
-3.19126	2.608142456	159.93563513	-654.2316882	-1717685.3	274.152733
-3.07726	2.612988248	160.44546865	-654.2314299	-1717684.62	274.8310624
-2.96326	2.618336204	160.93987555	-654.2311487	-1717683.88	275.5692979
-2.84926	2.624231129	161.40876932	-654.2308442	-1717683.08	276.3687758
-2.73527	2.630721135	161.84031626	-654.2305155	-1717682.22	277.2317934
-2.62129	2.637851718	162.22051668	-654.2301609	-1717681.29	278.162583
-2.50732	2.645643785	162.53195735	-654.2297778	-1717680.28	279.1686326
-2.39337	2.654071443	162.75446397	-654.2293615	-1717679.19	280.2614891
-2.27943	2.663041951	162.85777843	-654.2289062	-1717677.99	281.4569528
-2.1655	2.672398252	162.80825067	-654.2284043	-1717676.68	282.7746151
-2.05157	2.681932572	162.56660485	-654.2278474	-1717675.21	284.2369057
-1.93763	2.691414395	162.09255602	-654.2272263	-1717673.58	285.867567
-1.82368	2.700641893	161.35106295	-654.2265329	-1717671.76	287.687981
-1.70973	2.709481208	160.32361624	-654.2257615	-1717669.74	289.7134177
-1.59576	2.717854244	159.00095550	-654.2249086	-1717667.5	291.9526148
-1.48179	2.725729965	157.38599167	-654.223973	-1717665.04	294.4089381
-1.36781	2.733120845	155.48828802	-654.2229555	-1717662.37	297.080576

-1.25383	2.740032382	153.31610736	-654.221859	-1717659.49	299.9593869
-1.13983	2.746511558	150.89168376	-654.2206925	-1717656.43	303.0220379
-1.02584	2.752610807	148.23904763	-654.2194696	-1717653.22	306.2326883
-0.91184	2.758392413	145.38476284	-654.218209	-1717649.91	309.5424487
-0.79784	2.763915158	142.35629648	-654.2169313	-1717646.55	312.8970212
-0.68384	2.769217852	139.17352410	-654.2156604	-1717643.22	316.2337508
-0.56983	2.77434542	135.85959662	-654.2144302	-1717639.99	319.4637669
-0.45583	2.779381701	132.45100899	-654.2132872	-1717636.99	322.4646556
-0.34183	2.784358416	128.96596035	-654.2122828	-1717634.35	325.1016685
-0.22784	2.789285182	125.41449255	-654.2114743	-1717632.23	327.2244955
-0.11388	2.794199948	121.83262789	-654.2109293	-1717630.79	328.6553326
0	2.799255185	118.17934172	-654.2107298	-1717630.27	329.1790227
0.11374	2.804311469	114.53988763	-654.2109466	-1717630.84	328.6099403
0.2277	2.809521738	110.86954759	-654.2116331	-1717632.64	326.8073692
0.34169	2.814864811	107.16682458	-654.2128035	-1717635.72	323.7346362
0.45569	2.820336683	103.45631620	-654.2144263	-1717639.98	319.473904
0.56968	2.825906296	99.74830004	-654.2164384	-1717645.26	314.1911118
0.68368	2.831532846	96.05081719	-654.2187678	-1717651.37	308.0752668
0.79768	2.837206256	92.37916349	-654.2213444	-1717658.14	301.3103274
0.91168	2.842917692	88.74759610	-654.2241086	-1717665.4	294.0531251
1.02568	2.848654006	85.16727439	-654.2270126	-1717673.02	286.4285103
1.13969	2.854402267	81.64681212	-654.230019	-1717680.91	278.535194
1.2537	2.860165354	78.19543710	-654.2330971	-1717689	270.4537396
1.36771	2.865942593	74.82015158	-654.2362225	-1717697.2	262.2479467
1.48172	2.871727229	71.52351123	-654.2393769	-1717705.48	253.9659803
1.59574	2.877518248	68.30765573	-654.242545	-1717713.8	245.6483096
1.70975	2.883322229	65.17830160	-654.2457118	-1717722.12	237.3338158
1.82377	2.8891398	62.13831150	-654.2488651	-1717730.4	229.0547505
1.93779	2.894977186	59.18917276	-654.2519954	-1717738.61	220.8362792
2.05181	2.90082898	56.32834772	-654.2550948	-1717746.75	212.6986364
2.16583	2.906697782	53.55676169	-654.2581562	-1717754.79	204.6610253
2.27985	2.912594221	50.87662670	-654.2611723	-1717762.71	196.7423098
2.39387	2.918518793	48.28792291	-654.2641373	-1717770.49	188.9575973
2.50789	2.9244695	45.78989352	-654.2670465	-1717778.13	181.3195846
2.62191	2.930451501	43.38184855	-654.2698954	-1717785.61	173.8398974
2.73593	2.936468795	41.06403909	-654.2726797	-1717792.92	166.5296915
2.84995	2.942523628	38.83570632	-654.2753957	-1717800.05	159.3987416
2.96398	2.94861673	36.69633006	-654.27804	-1717806.99	152.456111
3.078	2.954745184	34.64659632	-654.2806093	-1717813.74	145.7105346
3.19202	2.960909277	32.68757760	-654.2831006	-1717820.28	139.1694112
3.30604	2.967105147	30.81901595	-654.285512	-1717826.61	132.8382778
3.42006	2.973329151	29.04017270	-654.2878419	-1717832.73	126.7212593
3.53409	2.979580043	27.35113243	-654.2900891	-1717838.63	120.8213512
3.64811	2.985851569	25.75101408	-654.292253	-1717844.31	115.1399241
3.76213	2.992135971	24.23931665	-654.2943337	-1717849.77	109.677083
3.87615	2.998420924	22.81431089	-654.2963314	-1717855.02	104.4319956

3.99017	3.004696832	21.47421271	-654.2982468	-1717860.05	99.40305518
4.10419	3.010948089	20.21729704	-654.3000809	-1717864.86	94.58780154
4.21821	3.017160343	19.04215542	-654.3018348	-1717869.47	89.98279006
4.33223	3.023321211	17.94675391	-654.3035101	-1717873.87	85.58427154
4.44625	3.029415761	16.92858436	-654.3051083	-1717878.06	81.38825257
4.56027	3.035429582	15.98468298	-654.306631	-1717882.06	77.3904221
4.67429	3.041347804	15.11127585	-654.3080801	-1717885.86	73.5857943
4.7883	3.047151143	14.30213929	-654.3094579	-1717889.48	69.9685458
4.90232	3.052828206	13.55120463	-654.3107666	-1717892.92	66.53238592
5.01634	3.058360945	12.85263716	-654.3120089	-1717896.18	63.27069577
5.13036	3.06373934	12.20190143	-654.3131874	-1717899.27	60.17658602
5.24437	3.068952088	11.59515509	-654.3143048	-1717902.21	57.24300723
5.35839	3.073988268	11.02765267	-654.3153637	-1717904.99	54.4628469
5.47241	3.078837625	10.49414617	-654.3163668	-1717907.62	51.82919472
5.58643	3.083491531	9.99099497	-654.3173166	-1717910.11	49.33542656
5.70044	3.087944253	9.51612764	-654.3182155	-1717912.47	46.97529372
5.81446	3.092192737	9.06792361	-654.3190658	-1717914.71	44.74302273
5.92848	3.096232857	8.64307102	-654.3198693	-1717916.82	42.63331796
6.04249	3.100061815	8.24028165	-654.3206281	-1717918.81	40.6411542
6.15651	3.103679873	7.85868702	-654.321344	-1717920.69	38.7615745
6.27053	3.107085817	7.49804448	-654.3220188	-1717922.46	36.98985297
6.38454	3.110278014	7.15683564	-654.3226543	-1717924.13	35.32131884
6.49855	3.113260601	6.83408127	-654.3232523	-1717925.7	33.75129609
6.61257	3.116035214	6.52780610	-654.3238146	-1717927.18	32.27499845
6.72658	3.118607302	6.23647785	-654.324343	-1717928.56	30.88752147
6.84059	3.120982957	5.95856506	-654.3248396	-1717929.87	29.58385307
6.9546	3.123169864	5.69292777	-654.3253061	-1717931.09	28.35887091
7.06861	3.125177027	5.43953217	-654.3257448	-1717932.24	27.20729248
7.18261	3.127017105	5.19827084	-654.3261574	-1717933.33	26.12379064
7.29661	3.12870179	4.96979888	-654.3265462	-1717934.35	25.10298574
7.41062	3.130242536	4.75407255	-654.3269133	-1717935.31	24.13936947
7.52462	3.131654773	4.55070402	-654.3272607	-1717936.22	23.22730228
7.63862	3.132947934	4.35882243	-654.3275905	-1717937.09	22.36121809
7.75263	3.134137822	4.17764238	-654.327905	-1717937.91	21.535669
7.86663	3.135230343	4.00566588	-654.328206	-1717938.7	20.74530161
7.98063	3.136236218	3.84141870	-654.3284955	-1717939.46	19.98519048
8.09464	3.137165146	3.68536629	-654.3287749	-1717940.2	19.25153651
8.20865	3.138024072	3.53841625	-654.3290451	-1717940.91	18.5421763
8.32267	3.138822383	3.40026103	-654.3293065	-1717941.59	17.85598087
8.43668	3.139562173	3.27049228	-654.3295594	-1717942.26	17.1919079
8.5507	3.140246223	3.14949112	-654.3298044	-1717942.9	16.54871554
8.66472	3.140879817	3.03696698	-654.3300419	-1717943.53	15.92511203
8.77874	3.141469794	2.93257219	-654.3302724	-1717944.13	15.31990802
8.89276	3.142019455	2.83607425	-654.3304963	-1717944.72	14.7320927
9.00678	3.142538669	2.74653373	-654.3307139	-1717945.29	14.16083641
9.12081	3.143030856	2.66356935	-654.3309254	-1717945.84	13.60540139

9.23483	3.143501849	2.58653334	-654.3311312	-1717946.39	13.06510499
9.34886	3.143954483	2.51440884	-654.3313315	-1717946.91	12.53923835
9.46288	3.14439272	2.44678208	-654.3315266	-1717947.42	12.02706368
9.57691	3.144818329	2.38289392	-654.3317167	-1717947.92	11.52783273
9.69094	3.145231851	2.32084577	-654.3319022	-1717948.41	11.04088387
9.80496	3.1456345	2.26055016	-654.3320832	-1717948.88	10.56577077
9.91899	3.146024665	2.20171819	-654.3322597	-1717949.35	10.10220986
10.03302	3.146402729	2.14458105	-654.332432	-1717949.8	9.65005413
10.14705	3.146766991	2.08858909	-654.3325998	-1717950.24	9.209264186
10.26108	3.147115327	2.03330717	-654.3327634	-1717950.67	8.779826905
10.3751	3.147449559	1.97873973	-654.3329227	-1717951.09	8.361679272
10.48913	3.14776402	1.92420454	-654.3330777	-1717951.5	7.954682139
10.60316	3.148059497	1.86947962	-654.3332285	-1717951.89	7.558654345
10.71719	3.148333554	1.81477119	-654.3333753	-1717952.28	7.17341473
10.83122	3.148584568	1.75950003	-654.333518	-1717952.65	6.798758505
10.94525	3.148819521	1.70402714	-654.3336567	-1717953.02	6.434436249
11.05927	3.149038537	1.64800710	-654.3337916	-1717953.37	6.08026155
11.1733	3.149242074	1.59123711	-654.3339227	-1717953.71	5.736097882
11.28733	3.149433296	1.53401631	-654.33405	-1717954.05	5.401813971
11.40136	3.149612448	1.47659433	-654.3341736	-1717954.37	5.077302171
11.51539	3.149780751	1.41971962	-654.3342935	-1717954.69	4.762509972
11.62942	3.14993852	1.36371528	-654.3344097	-1717954.99	4.457421621
11.74345	3.150083457	1.30924203	-654.3345222	-1717955.29	4.162055497
11.85748	3.150220597	1.25691646	-654.334631	-1717955.57	3.876466734
11.97151	3.150348091	1.20637676	-654.334736	-1717955.85	3.600734099
12.08554	3.150467286	1.15756046	-654.3348373	-1717956.12	3.334954734
12.19957	3.150580345	1.11029259	-654.3349347	-1717956.37	3.079212655
12.3136	3.150685033	1.06443856	-654.3350282	-1717956.62	2.833534118
12.42763	3.150785397	1.01974605	-654.335118	-1717956.85	2.597919123
12.54166	3.150879099	0.97551045	-654.3352039	-1717957.08	2.372344039
12.65569	3.150969493	0.93203191	-654.335286	-1717957.29	2.15677211
12.76972	3.151058935	0.88904941	-654.3353643	-1717957.5	1.951198086
12.88375	3.151146766	0.84643108	-654.3354388	-1717957.69	1.755629842
12.99778	3.151236033	0.80424834	-654.3355094	-1717957.88	1.570098884
13.11181	3.151325765	0.76221297	-654.3355763	-1717958.06	1.394639345
13.22584	3.151416393	0.71990749	-654.3356393	-1717958.22	1.229269602
13.33987	3.151508232	0.67748396	-654.3356984	-1717958.38	1.073916141
13.4539	3.151600571	0.63406329	-654.3357538	-1717958.52	0.928447688
13.56793	3.15168915	0.59028343	-654.3358055	-1717958.66	0.792769725
13.68196	3.151769621	0.54605105	-654.3358535	-1717958.78	0.666800861
13.79599	3.151837645	0.50175724	-654.3358978	-1717958.9	0.550433449
13.91002	3.151893929	0.45768598	-654.3359385	-1717959.01	0.443494209
14.02405	3.151935005	0.41385743	-654.3359757	-1717959.1	0.345864991
14.13808	3.151961952	0.37054430	-654.3360094	-1717959.19	0.25750904
14.25211	3.151978441	0.32725155	-654.3360395	-1717959.27	0.178502494
14.36614	3.151989796	0.28375758	-654.3360659	-1717959.34	0.109034389

14.48017	3.152002506	0.23953334	-654.3360886	-1717959.4	0.049419787
14.5942	3.152023497	0.19333270	-654.3361075	-1717959.45	0

[a] E_{rel} refers to the energy of each species relative to the energy of **TP-($C_7H_7^+$)** (the lowest-energy structure).

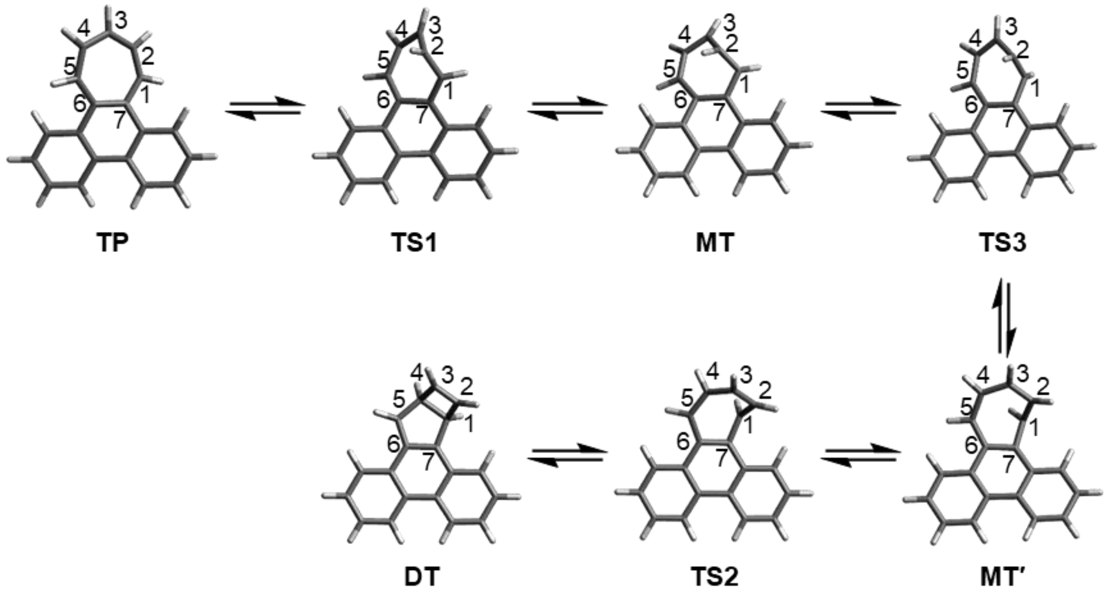

Fig. S73. Rearrangement of the **TP** isomer of phenanthrenyltropylium (**Phen**) to the **DT** isomer *via* the **MT** intermediate

Table S20. Relative energies (G_{rel} /·kJ mol^{-1}), d(C_1–C_4) / Å (internuclear distance between C_1 and C_4), and H_4–C_4–C_5–H_5 dihedral angles (Φ / °) of the key structures in the isomerism of **TP-Phen.**

Structure	d(C_1–C_4) / Å	Φ / °	G_{rel} / kJ·mol^{-1} [a]
**TP**	3.08	0.827	0
**TS1**	2.83	116	297
**MT**	3.03	165	232
**TS3**	2.77	175	261
**MT′**	2.54	156	209
**TS2**	2.11	107	250
**DT**	1.56	61.4	143

[a] G_{rel} refers to the energy of each species relative to the energy of **TP-Phen** (the lowest-energy structure).

Table S21. IRC Calculation for the rearrangement of the **DT** isomer of **Phen** to the **TP** isomer *via* a **MT** intermediate, showing the change in energy (E) as a function of d(C_1–C_4): the bond distance between C_1 and C_4 and the H_4–C_4–C_5–H_5 dihedral angle (Φ / °). E_{rel} refers to the energy of each species relative to the energy of **TP-Phen** (the lowest-energy structure).

Coordinate	d(C_1–C_4) / Å	Φ / °	E / Hartree	E / kJ·mol^{-1}	E_{rel} / kJ·mol^{-1} [a]
-8.10356	1.55901493	61.258760	-731.7042585	-1921089.53	150.324927
-7.93107	1.559032279	61.238090	-731.7042347	-1921089.47	150.3874034
-7.75858	1.559050507	61.213096	-731.7042056	-1921089.39	150.4638527
-7.58609	1.559070886	61.184464	-731.704171	-1921089.3	150.5546425
-7.4136	1.559095938	61.151959	-731.7041309	-1921089.2	150.6600484
-7.24111	1.559123022	61.114942	-731.7040851	-1921089.08	150.7801782

-7.06862	1.55915779	61.073857	-731.7040337	-1921088.94	150.9152313
-6.89612	1.55920636	61.028420	-731.7039764	-1921088.79	151.0657433
-6.72363	1.559269283	60.979142	-731.7039129	-1921088.62	151.2324337
-6.55114	1.559356097	60.926800	-731.7038428	-1921088.44	151.41635
-6.37866	1.559473492	60.871102	-731.7037657	-1921088.24	151.6190071
-6.2062	1.559633141	60.810383	-731.7036806	-1921088.01	151.8423163
-6.03376	1.559845717	60.739329	-731.7035867	-1921087.77	152.0887589
-5.86137	1.560123379	60.654792	-731.7034828	-1921087.49	152.3616114
-5.68906	1.560479698	60.552216	-731.7033667	-1921087.19	152.6664214
-5.51683	1.560928187	60.424305	-731.7032346	-1921086.84	153.0131922
-5.34463	1.561474519	60.270824	-731.7030808	-1921086.44	153.4171595
-5.17239	1.56210371	60.110580	-731.702898	-1921085.96	153.8971482
-5.00012	1.562776838	59.994605	-731.702679	-1921085.38	154.472004
-4.82784	1.563531386	59.907441	-731.7024172	-1921084.7	155.1593389
-4.65559	1.564400171	59.842527	-731.7021034	-1921083.87	155.9831578
-4.48334	1.565412834	59.828992	-731.7017243	-1921082.88	156.9784848
-4.31105	1.566656055	59.890240	-731.7012642	-1921081.67	158.1864669
-4.13872	1.568299108	60.043341	-731.7007069	-1921080.21	159.6497079
-3.96636	1.570675677	60.299537	-731.7000369	-1921078.45	161.4088113
-3.79401	1.57438969	60.678659	-731.6992385	-1921076.35	163.5049843
-3.62172	1.580379242	61.207638	-731.6982917	-1921073.86	165.9908995
-3.4495	1.589721482	61.919159	-731.6971666	-1921070.91	168.9447026
-3.27729	1.603122131	62.841425	-731.695823	-1921067.38	172.4725607
-3.10502	1.620506769	63.985496	-731.6942214	-1921063.18	176.6774932
-2.93266	1.641216211	65.342215	-731.6923425	-1921058.25	181.6103824
-2.76024	1.664437624	66.892920	-731.6901961	-1921052.61	187.2459525
-2.58777	1.689464338	68.618052	-731.6878179	-1921046.37	193.4898326
-2.41527	1.715761716	70.494609	-731.6852628	-1921039.66	200.1983054
-2.24276	1.742946875	72.507751	-731.6825956	-1921032.65	207.2008867
-2.07024	1.770751336	74.645260	-731.6798851	-1921025.54	214.3173675
-1.89772	1.798986939	76.902399	-731.677199	-1921018.49	221.3697703
-1.72519	1.827522439	79.265288	-731.6746011	-1921011.67	228.1905882
-1.55267	1.856264907	81.719431	-731.6721474	-1921005.22	234.6326909
-1.38014	1.885143695	84.257597	-731.6698843	-1920999.28	240.5744547
-1.20762	1.914092476	86.882004	-731.66785	-1920993.94	245.9156065
-1.03509	1.943058722	89.573371	-731.666076	-1920989.28	250.5731516
-0.86257	1.971968562	92.306788	-731.6645858	-1920985.37	254.4858214
-0.69005	2.000697702	95.083825	-731.663391	-1920982.23	257.6225719
-0.51754	2.029080056	97.918090	-731.6624917	-1920979.87	259.9836499
-0.34503	2.056968653	100.816542	-731.6618762	-1920978.26	261.599758
-0.17254	2.084302212	103.774393	-731.6615239	-1920977.33	262.5246324
0	2.110530607	106.788694	-731.6614121	-1920977.04	262.8182552
0.17254	2.136763551	109.784490	-731.6615165	-1920977.31	262.5441556
0.34503	2.161846439	112.796631	-731.661816	-1920978.1	261.7577081
0.51754	2.186310411	115.803804	-731.6622871	-1920979.33	260.5208482
0.69005	2.21010946	118.754960	-731.6629052	-1920980.96	258.8981474

0.86257	2.233179963	121.667128	-731.6636464	-1920982.9	256.9520901
1.03508	2.255492647	124.534086	-731.6644882	-1920985.11	254.7420177
1.20759	2.277033088	127.335725	-731.6654083	-1920987.53	252.3261849
1.38011	2.297811481	130.054161	-731.6663853	-1920990.09	249.7610924
1.55262	2.317819592	132.675112	-731.6673985	-1920992.75	247.1009988
1.72513	2.33706681	135.179657	-731.6684274	-1920995.46	244.3996008
1.89764	2.355574832	137.554905	-731.6694524	-1920998.15	241.7084502
2.07014	2.373349239	139.791407	-731.6704555	-1921000.78	239.0748164
2.24264	2.390352898	141.877202	-731.6714211	-1921003.32	236.5397491
2.41513	2.406533416	143.803350	-731.6723366	-1921005.72	234.1359096
2.58761	2.421838717	145.560962	-731.6731925	-1921007.97	231.888752
2.76008	2.43619416	147.142039	-731.6739823	-1921010.04	229.8151242
2.93253	2.449520992	148.544022	-731.6747032	-1921011.93	227.922354
3.10497	2.461742247	149.767050	-731.6753558	-1921013.65	226.2091286
3.27741	2.472815975	150.815549	-731.6759429	-1921015.19	224.6675243
3.44983	2.482733308	151.699628	-731.6764695	-1921016.57	223.2850961
3.62227	2.491529833	152.432687	-731.6769407	-1921017.81	222.047887
3.79471	2.49928286	153.030734	-731.6773614	-1921018.91	220.9434389
3.96718	2.506094707	153.513125	-731.677735	-1921019.89	219.9625627
4.13966	2.512092972	153.899740	-731.6780637	-1921020.76	219.09944
4.31216	2.51741868	154.204885	-731.678349	-1921021.51	218.3503192
4.48466	2.522201634	154.443262	-731.6785918	-1921022.14	217.7128452
4.65716	2.526557957	154.630714	-731.6787926	-1921022.67	217.1857603
4.82964	2.530566797	154.780623	-731.6789518	-1921023.09	216.767681
5.00204	2.53426339	154.903534	-731.6790707	-1921023.4	216.4555143
5.17409	2.537591275	155.012548	-731.6791519	-1921023.61	216.2423841
5.3448	2.540265171	155.130489	-731.6792015	-1921023.74	216.1122853
5.51486	2.541787592	155.273318	-731.6792314	-1921023.82	216.0338012
14.5362	2.541339968	155.368994	-731.6792342	-1921023.83	216.0262161
14.40524	2.541098917	155.386090	-731.6792145	-1921023.78	216.0780671
14.27427	2.54087152	155.407098	-731.679193	-1921023.72	216.1345338
14.14331	2.54066187	155.431354	-731.6791696	-1921023.66	216.1958261
14.01234	2.540466573	155.458253	-731.6791444	-1921023.59	216.2620385
13.88137	2.540282558	155.486476	-731.6791173	-1921023.52	216.3332552
13.75041	2.540111293	155.514384	-731.6790883	-1921023.45	216.4095234
13.61945	2.539951423	155.538698	-731.6790573	-1921023.36	216.4908167
13.48849	2.539801034	155.558348	-731.6790245	-1921023.28	216.576857
13.35752	2.53965942	155.573074	-731.6789901	-1921023.19	216.667169
13.22656	2.539524672	155.584679	-731.6789543	-1921023.09	216.7612039
13.09559	2.539397744	155.595593	-731.6789173	-1921023	216.8583421
12.96463	2.539280164	155.608388	-731.6788793	-1921022.9	216.9581662
12.83367	2.539172086	155.625304	-731.6788403	-1921022.8	217.0605502
12.7027	2.539076608	155.646446	-731.6788003	-1921022.69	217.1655834
12.57174	2.539003956	155.674168	-731.6787593	-1921022.58	217.2732131
12.44078	2.538943929	155.707904	-731.6787173	-1921022.47	217.3835708
12.30982	2.538897066	155.747599	-731.6786741	-1921022.36	217.4968348

12.17885	2.538858368	155.792994	-731.6786298	-1921022.24	217.6132075
12.04789	2.538826101	155.842921	-731.6785842	-1921022.12	217.732946
11.91693	2.538800594	155.896418	-731.6785372	-1921022	217.8562763
11.78596	2.538778043	155.951565	-731.6784888	-1921021.87	217.9833636
11.655	2.538763409	156.007205	-731.6784389	-1921021.74	218.1142789
11.52403	2.538752138	156.062678	-731.6783876	-1921021.61	218.2491141
11.39307	2.538745963	156.117719	-731.6783347	-1921021.47	218.387982
11.26211	2.538742567	156.174126	-731.6782802	-1921021.32	218.5309799
11.13114	2.538739696	156.232911	-731.6782241	-1921021.18	218.6783466
11.00018	2.538741761	156.295329	-731.6781661	-1921021.03	218.8305048
10.86922	2.538745392	156.361760	-731.6781062	-1921020.87	218.9879849
10.73825	2.538751439	156.432472	-731.6780439	-1921020.7	219.1513829
10.60729	2.538751178	156.503897	-731.6779792	-1921020.53	219.3212711
10.47633	2.538750982	156.578764	-731.6779118	-1921020.36	219.4981905
10.34536	2.538750842	156.654647	-731.6778416	-1921020.17	219.6825688
10.2144	2.538751584	156.730186	-731.6777685	-1921019.98	219.8746609
10.08344	2.538754379	156.805039	-731.6776923	-1921019.78	220.0745166
9.95247	2.538760064	156.879346	-731.6776133	-1921019.57	220.2819889
9.82151	2.538770204	156.953024	-731.6775315	-1921019.36	220.4967836
9.69055	2.538789318	157.027078	-731.677447	-1921019.14	220.7185675
9.55958	2.538822527	157.102664	-731.67736	-1921018.91	220.9469965
9.42862	2.538872416	157.180796	-731.6772706	-1921018.67	221.1817818
9.29766	2.538943236	157.262477	-731.6771788	-1921018.43	221.422766
9.1667	2.539036949	157.348906	-731.6770847	-1921018.19	221.6699358
9.03573	2.539153036	157.440013	-731.6769881	-1921017.93	221.9234252
8.90477	2.539292125	157.535377	-731.6768891	-1921017.67	222.1834626
8.77381	2.539449641	157.634440	-731.6767874	-1921017.41	222.4503368
8.64284	2.539621929	157.736848	-731.6766831	-1921017.13	222.7243734
8.51188	2.53980235	157.842657	-731.6765758	-1921016.85	223.0059504
8.38092	2.539988586	157.950903	-731.6764655	-1921016.56	223.2955273
8.24995	2.54017689	158.060976	-731.676352	-1921016.26	223.5936397
8.11899	2.540364309	158.172674	-731.676235	-1921015.95	223.9008415
7.98803	2.540551802	158.285690	-731.6761143	-1921015.64	224.2176029
7.85706	2.540736975	158.400018	-731.6759899	-1921015.31	224.5443096
7.7261	2.540917503	158.515565	-731.6758615	-1921014.97	224.8812636
7.59514	2.541091909	158.632408	-731.6757292	-1921014.63	225.2286934
7.46418	2.541257244	158.751408	-731.6755929	-1921014.27	225.5866908
7.33321	2.541413985	158.873000	-731.6754525	-1921013.9	225.955269
7.20225	2.541562642	158.997228	-731.6753081	-1921013.52	226.334428
7.07129	2.541699716	159.123892	-731.6751596	-1921013.13	226.7241913
6.94033	2.541828432	159.254375	-731.6750071	-1921012.73	227.12463
6.80936	2.541950921	159.389938	-731.6748505	-1921012.32	227.5358253
6.6784	2.542073977	159.530735	-731.6746897	-1921011.9	227.9578061
6.54744	2.542192141	159.677179	-731.6745249	-1921011.47	228.3905673
6.41648	2.54230445	159.831653	-731.6743559	-1921011.02	228.834198
6.28552	2.542411293	159.996029	-731.6741828	-1921010.57	229.2889084

6.15456	2.54251432	160.171121	-731.6740052	-1921010.1	229.755037
6.0236	2.54262151	160.358641	-731.6738231	-1921009.62	230.2330671
5.89264	2.542743269	160.559006	-731.6736363	-1921009.13	230.7235446
5.76169	2.542892244	160.772537	-731.6734446	-1921008.63	231.2270367
5.63073	2.543079903	161.000476	-731.6732476	-1921008.11	231.7441946
5.49977	2.543318203	161.243047	-731.6730451	-1921007.58	232.2758295
5.36882	2.543628766	161.501143	-731.6728367	-1921007.03	232.82296
5.23787	2.544026607	161.778197	-731.672622	-1921006.47	233.3867757
5.10692	2.544529853	162.077720	-731.6724003	-1921005.89	233.9687204
4.97597	2.545160229	162.402726	-731.6721711	-1921005.29	234.5705532
4.84502	2.545945002	162.758300	-731.6719335	-1921004.66	235.1943221
4.71408	2.546911563	163.146394	-731.6716867	-1921004.01	235.842201
4.58314	2.5480893	163.567394	-731.6714299	-1921003.34	236.5166185
4.45219	2.549514366	164.024357	-731.6711618	-1921002.64	237.2203759
4.32126	2.551226953	164.520699	-731.6708814	-1921001.9	237.9565398
4.19032	2.553258722	165.059956	-731.6705875	-1921001.13	238.7281427
4.05938	2.555639529	165.643930	-731.670279	-1921000.32	239.5380964
3.92844	2.558389222	166.274162	-731.669955	-1920999.47	240.3887505
3.7975	2.561522678	166.946559	-731.669615	-1920998.57	241.2815833
3.66656	2.565057266	167.649795	-731.6692585	-1920997.64	242.2176318
3.53561	2.56900451	168.379164	-731.668885	-1920996.66	243.1981484
3.40467	2.573370908	169.134507	-731.6684942	-1920995.63	244.2241728
3.27372	2.578147462	169.916355	-731.6680861	-1920994.56	245.2957759
3.14277	2.583322942	170.715482	-731.667661	-1920993.44	246.4118812
3.01182	2.588879088	171.529119	-731.6672196	-1920992.29	247.5705484
2.88087	2.594787676	172.355556	-731.6667633	-1920991.09	248.7688004
2.74991	2.601019067	173.189279	-731.6662935	-1920989.85	250.0020266
2.61896	2.607543485	174.029334	-731.6658127	-1920988.59	251.2644983
2.488	2.614331334	174.868689	-731.6653234	-1920987.31	252.5492185
2.35705	2.621362839	175.699261	-731.6648286	-1920986.01	253.848371
2.22609	2.628616024	176.511114	-731.6643311	-1920984.7	255.1543393
2.09513	2.636061678	177.301881	-731.6638339	-1920983.4	256.4599401
1.96417	2.643669334	178.072287	-731.6633397	-1920982.1	257.757218
1.83322	2.651402036	178.827222	-731.6628524	-1920980.82	259.0367318
1.70226	2.659243512	179.564502	-731.662376	-1920979.57	260.2875436
1.5713	2.667183801	180.270206	-731.6619149	-1920978.36	261.4982116
1.44034	2.675205629	180.941156	-731.6614729	-1920977.2	262.6585434
1.30938	2.683272032	181.579028	-731.6610538	-1920976.1	263.7588537
1.17843	2.69136192	182.178199	-731.660662	-1920975.07	264.787753
1.04747	2.699470827	182.732526	-731.6603015	-1920974.12	265.7342248
0.91651	2.707592835	183.246321	-731.6599759	-1920973.27	266.5891086
0.78556	2.715741795	183.703555	-731.6596882	-1920972.51	267.3444334
0.6546	2.723916921	184.114262	-731.6594407	-1920971.86	267.9941659
0.52365	2.732113711	184.484043	-731.6592351	-1920971.32	268.5340265
0.3927	2.74032901	184.806197	-731.6590726	-1920970.9	268.9606098
0.26175	2.748574511	185.069221	-731.6589547	-1920970.59	269.2702167

0.13082	2.756844685	185.283997	-731.6588826	-1920970.4	269.4593629
0	2.765062003	185.375933	-731.6588574	-1920970.33	269.5254967
-0.1309	2.773284736	185.466318	-731.6588784	-1920970.39	269.4704347
-0.26183	2.781501333	185.416684	-731.6589452	-1920970.56	269.2951379
-0.39277	2.789803882	185.308256	-731.6590559	-1920970.85	269.0044399
-0.52372	2.79818654	185.142475	-731.6592077	-1920971.25	268.6057866
-0.65468	2.80663488	184.941390	-731.659396	-1920971.74	268.1116544
-0.78563	2.815106881	184.716551	-731.6596161	-1920972.32	267.5336506
-0.91659	2.823546031	184.452438	-731.6598651	-1920972.98	266.8798985
-1.04754	2.831953266	184.149870	-731.66014	-1920973.7	266.1581774
-1.1785	2.840338461	183.805260	-731.6604376	-1920974.48	265.3766816
-1.30946	2.848719785	183.420311	-731.6607546	-1920975.31	264.5446107
-1.44041	2.857110302	182.998225	-731.661087	-1920976.18	263.6719339
-1.57137	2.865518359	182.539746	-731.6614308	-1920977.09	262.7692424
-1.70233	2.873910356	182.043853	-731.661782	-1920978.01	261.8470434
-1.83328	2.882264482	181.518706	-731.6621372	-1920978.94	260.9146076
-1.96423	2.890551815	180.975675	-731.6624932	-1920979.88	259.9797431
-2.09519	2.898740276	180.426065	-731.6628475	-1920980.81	259.0495653
-2.22614	2.906797779	179.873214	-731.6631973	-1920981.72	258.1311338
-2.35709	2.914683425	179.310139	-731.6635403	-1920982.63	257.2306582
-2.48804	2.922365674	178.733460	-731.6638747	-1920983.5	256.3527094
-2.61899	2.929825669	178.144722	-731.6641995	-1920984.36	255.4999549
-2.74995	2.937038646	177.543391	-731.6645144	-1920985.18	254.6732926
-2.8809	2.94398093	176.917291	-731.6648193	-1920985.98	253.8726411
-3.01185	2.950643834	176.266914	-731.6651143	-1920986.76	253.098242
-3.1428	2.957018839	175.629186	-731.6653982	-1920987.5	252.352789
-3.27374	2.963067604	175.021510	-731.6656698	-1920988.22	251.6396744
-3.40469	2.96873571	174.436340	-731.6659284	-1920988.9	250.9607017
-3.53563	2.973998255	173.865051	-731.6661741	-1920989.54	250.3156295
-3.66657	2.978862742	173.308424	-731.6664073	-1920990.15	249.7034626
-3.79751	2.983342182	172.768710	-731.6666283	-1920990.73	249.1230355
-3.92845	2.987452774	172.249202	-731.6668378	-1920991.28	248.573093
-4.05939	2.991204183	171.756325	-731.6670362	-1920991.8	248.0521308
-4.19033	2.994595886	171.287755	-731.6672244	-1920992.3	247.5581062
-4.32127	2.99763059	170.846627	-731.667403	-1920992.77	247.0890317
-4.4522	3.000303094	170.435014	-731.6675729	-1920993.21	246.6432008
-4.58314	3.00261483	170.054475	-731.6677345	-1920993.64	246.2188491
-4.71407	3.004586943	169.703139	-731.6678886	-1920994.04	245.8141231
-4.84501	3.006261212	169.381277	-731.6680359	-1920994.43	245.4274316
-4.97596	3.00767858	169.088779	-731.6681768	-1920994.8	245.0574986
-5.1069	3.008887377	168.822738	-731.6683117	-1920995.15	244.7032504
-5.23785	3.009928823	168.580130	-731.6684411	-1920995.49	244.3636131
-5.36881	3.010844195	168.355585	-731.6685653	-1920995.82	244.0374472
-5.49976	3.011665681	168.146519	-731.6686849	-1920996.13	243.7236002
-5.63072	3.012419088	167.949512	-731.6688001	-1920996.43	243.4210665
-5.76168	3.013128465	167.762191	-731.6689113	-1920996.73	243.1291397

-5.89264	3.013806372	167.585138	-731.6690186	-1920997.01	242.8474446
-6.0236	3.014458781	167.417592	-731.669122	-1920997.28	242.5758471
-6.15455	3.01509806	167.258247	-731.6692216	-1920997.54	242.3143578
-6.28551	3.015732735	167.105822	-731.6693173	-1920997.79	242.0630633
-6.41647	3.016364123	166.961787	-731.6694092	-1920998.03	241.8219873
-6.54743	3.016982126	166.827781	-731.6694971	-1920998.26	241.5909958
-6.67839	3.017584499	166.702992	-731.6695813	-1920998.49	241.3699576
-6.80935	3.018166851	166.587533	-731.6696618	-1920998.7	241.158702
-6.94031	3.018729509	166.481454	-731.6697387	-1920998.9	240.9569008
-7.07127	3.019282524	166.383041	-731.6698121	-1920999.09	240.7641261
-7.20223	3.019825037	166.290738	-731.6698822	-1920999.28	240.5799289
-7.33319	3.020356278	166.205674	-731.6699493	-1920999.45	240.4039154
-7.46415	3.020875499	166.126719	-731.6700133	-1920999.62	240.2357469
-7.59511	3.02139041	166.052951	-731.6700745	-1920999.78	240.0751634
-7.72607	3.021896508	165.983663	-731.6701328	-1920999.93	239.9219707
-7.85703	3.022395209	165.918557	-731.6701884	-1921000.08	239.7760165
-7.98799	3.022881347	165.856816	-731.6702413	-1921000.22	239.6371985
-8.11895	3.023353663	165.797038	-731.6702915	-1921000.35	239.5054535
-8.2499	3.023811717	165.738228	-731.670339	-1921000.47	239.3808132
-8.38086	3.024254947	165.680564	-731.6703836	-1921000.59	239.2634586
-8.51182	3.024679684	165.623726	-731.6704255	-1921000.7	239.1536208
-8.64278	3.025083526	165.568072	-731.6704643	-1921000.8	239.0515938
-8.77375	3.025468569	165.512773	-731.6705001	-1921000.9	238.9577926
-8.90471	3.025836872	165.457552	-731.6705325	-1921000.98	238.8726056
-9.03568	3.026192945	165.401980	-731.6705616	-1921001.06	238.7962981
-9.16664	3.026549842	165.344646	-731.6705872	-1921001.13	238.728933
-9.29761	3.026912564	165.285401	-731.6706095	-1921001.19	238.6704211
-9.42857	3.027283299	165.224796	-731.6706285	-1921001.24	238.6205629
6.34456	3.031358832	164.112202	-731.6705699	-1921001.08	238.7743542
6.23121	3.031695142	163.995960	-731.6705527	-1921001.04	238.8195443
6.11787	3.031946389	163.893320	-731.6705337	-1921000.99	238.8694524
6.00452	3.032098775	163.806579	-731.6705127	-1921000.93	238.9246168
5.89117	3.032198888	163.725110	-731.6704895	-1921000.87	238.985615
5.77783	3.032264016	163.644248	-731.6704638	-1921000.8	239.0528935
5.66448	3.032304922	163.561222	-731.6704357	-1921000.73	239.1268617
5.55114	3.032324379	163.474938	-731.6704048	-1921000.65	239.2079292
5.43781	3.032315039	163.382672	-731.6703711	-1921000.56	239.2964821
5.32448	3.032263403	163.284095	-731.6703343	-1921000.46	239.3929167
5.21116	3.032158404	163.179060	-731.6702944	-1921000.36	239.4977346
5.09785	3.031988531	163.066482	-731.6702511	-1921000.24	239.6115789
4.98454	3.031730538	162.947113	-731.670204	-1921000.12	239.7352294
4.87125	3.031337896	162.823320	-731.6701528	-1920999.99	239.869613
4.75798	3.030723991	162.693761	-731.6700971	-1920999.84	240.0157221
4.64474	3.029711582	162.562967	-731.6700367	-1920999.68	240.1744939
4.53153	3.028020309	162.473148	-731.6699707	-1920999.51	240.3475328
4.41832	3.025495821	162.467099	-731.6698979	-1920999.32	240.5388004

4.30508	3.02216871	162.545829	-731.6698158	-1920999.1	240.7544249
4.19182	3.018115925	162.693461	-731.669722	-1920998.86	241.0005156
4.07853	3.01339181	162.894943	-731.6696147	-1920998.57	241.2822265
3.96523	3.008029451	163.138982	-731.6694922	-1920998.25	241.6038923
3.85193	3.002052459	163.416749	-731.669353	-1920997.89	241.969375
3.73862	2.99548034	163.720187	-731.6691957	-1920997.47	242.382332
3.62531	2.988345529	164.042120	-731.669019	-1920997.01	242.8464233
3.51199	2.980689132	164.377822	-731.6688213	-1920996.49	243.3655214
3.39869	2.972566642	164.721063	-731.668601	-1920995.91	243.9438849
3.28538	2.964056986	165.065063	-731.6683563	-1920995.27	244.5863264
3.17209	2.95526054	165.400692	-731.668085	-1920994.56	245.298614
3.05881	2.946312143	165.715962	-731.6677843	-1920993.77	246.0881124
2.94554	2.937371975	165.994568	-731.6674505	-1920992.89	246.9643362
2.83228	2.928617158	166.220065	-731.6670791	-1920991.92	247.9395992
2.71903	2.920215471	166.373533	-731.666664	-1920990.83	249.0293235
2.60578	2.912310258	166.434224	-731.6661986	-1920989.6	250.2514255
2.49252	2.904995799	166.383676	-731.6656754	-1920988.23	251.6249479
2.37924	2.898311802	166.200229	-731.6650874	-1920986.69	253.1687341
2.26596	2.892258501	165.856448	-731.6644278	-1920984.96	254.9005217
2.15267	2.886804588	165.318112	-731.6636907	-1920983.02	256.8358644
2.03938	2.881894811	164.559269	-731.6628718	-1920980.87	258.9857236
1.92607	2.877457705	163.566688	-731.6619697	-1920978.5	261.3543631
1.81276	2.873416019	162.334384	-731.6609844	-1920975.91	263.9411868
1.69945	2.869696235	160.858897	-731.6599175	-1920973.11	266.7424535
1.58614	2.866237438	159.139720	-731.6587702	-1920970.1	269.7546004
1.47282	2.862996746	157.173962	-731.6575436	-1920966.88	272.97496
1.35951	2.859939703	154.965334	-731.6562406	-1920963.46	276.3959208
1.24618	2.857030264	152.533881	-731.6548692	-1920959.86	279.9965788
1.13286	2.854243281	149.898685	-731.6534413	-1920956.11	283.7455854
1.01953	2.851562254	147.073501	-731.6519708	-1920952.25	287.606349
0.9062	2.84897956	144.076629	-731.6504757	-1920948.32	291.5317865
0.79287	2.846492478	140.931414	-731.6489795	-1920944.4	295.4600308
0.67954	2.844107499	137.655087	-731.6475116	-1920940.54	299.313976
0.56621	2.841833872	134.265092	-731.6461082	-1920936.86	302.9985895
0.45288	2.839686563	130.787249	-731.6448135	-1920933.46	306.3978165
0.33955	2.837690073	127.234083	-731.6436772	-1920930.47	309.3813192
0.22623	2.835873868	123.613134	-731.6427589	-1920928.06	311.7922449
0.11295	2.834218763	119.959507	-731.6421334	-1920926.42	313.4346107
0	2.833018185	116.190636	-731.6419024	-1920925.81	314.0411091
-0.11322	2.831818269	112.437451	-731.6421819	-1920926.55	313.3070587
-0.22649	2.831113161	108.641332	-731.6430942	-1920928.94	310.911962
-0.3398	2.830698422	104.796940	-731.6446981	-1920933.15	306.7008885
-0.45311	2.830560799	100.924435	-731.6469439	-1920939.05	300.8045327
-0.56643	2.830658547	97.039675	-731.6496925	-1920946.27	293.5880256
-0.67975	2.830960005	93.160097	-731.6527975	-1920954.42	285.4358114
-0.79307	2.831461687	89.302858	-731.6561421	-1920963.2	276.6547295

-0.9064	2.832160069	85.487614	-731.6596406	-1920972.39	267.4692707
-1.01973	2.833058495	81.728435	-731.6632326	-1920981.82	258.0386112
-1.13306	2.834173673	78.038060	-731.6668736	-1920991.38	248.479087
-1.2464	2.83551811	74.424627	-731.6705341	-1921000.99	238.8685571
-1.35974	2.837100592	70.891364	-731.6741947	-1921010.6	229.2576439
-1.47307	2.838925801	67.441252	-731.6778412	-1921020.17	219.683619
-1.58641	2.840998838	64.077211	-731.6814608	-1921029.68	210.1803934
-1.69976	2.843333817	60.806524	-731.68504	-1921039.07	200.783167
-1.8131	2.845935394	57.632304	-731.6885686	-1921048.34	191.5189747
-1.92644	2.848805602	54.554413	-731.6920393	-1921057.45	182.406623
-2.03979	2.851948735	51.574436	-731.695445	-1921066.39	173.4649708
-2.15313	2.855365954	48.696486	-731.698778	-1921075.14	164.7140401
-2.26648	2.85905705	45.920494	-731.7020325	-1921083.69	156.1694396
-2.37982	2.863021593	43.243675	-731.7052042	-1921092.01	147.8420704
-2.49317	2.86725385	40.663529	-731.7082894	-1921100.11	139.7419408
-2.60651	2.871755102	38.182559	-731.7112829	-1921107.97	131.8823779
-2.71986	2.87652519	35.804857	-731.7141792	-1921115.58	124.2782
-2.83321	2.88156147	33.530974	-731.7169737	-1921122.91	116.941298
-2.94656	2.886856417	31.361753	-731.7196626	-1921129.97	109.8815727
-3.0599	2.892402795	29.297467	-731.7222429	-1921136.75	103.1069163
-3.17325	2.8981893	27.338213	-731.724713	-1921143.23	96.62164512
-3.2866	2.904200354	25.478123	-731.7270733	-1921149.43	90.42475886
-3.39994	2.910412049	23.711484	-731.7293251	-1921155.34	84.51277086
-3.51329	2.916808836	22.035277	-731.731469	-1921160.97	78.88380125
-3.62663	2.923377589	20.451584	-731.7335049	-1921166.32	73.53867707
-3.73998	2.930106138	18.966713	-731.7354318	-1921171.38	68.47945935
-3.85332	2.936987747	17.586601	-731.7372495	-1921176.15	63.70717726
-3.96666	2.94399908	16.311154	-731.7389585	-1921180.64	59.22016888
-4.08	2.951115114	15.139860	-731.7405605	-1921184.84	55.01417039
-4.19334	2.958310893	14.069119	-731.742058	-1921188.77	51.08235812
-4.30668	2.965553053	13.092690	-731.7434546	-1921192.44	47.41561108
-4.42002	2.972807367	12.201065	-731.7447544	-1921195.85	44.00315421
-4.53335	2.980040164	11.386102	-731.7459617	-1921199.02	40.83327516
-4.64669	2.987218778	10.641068	-731.7470812	-1921201.96	37.89394389
-4.76002	2.994309046	9.961981	-731.7481176	-1921204.68	35.17287307
-4.87335	3.001277816	9.344835	-731.7490757	-1921207.2	32.65755218
-4.98668	3.008088501	8.786324	-731.74996	-1921209.52	30.33575951
-5.10001	3.014714918	8.280421	-731.7507751	-1921211.66	28.19567246
-5.21333	3.021124296	7.820136	-731.7515255	-1921213.63	26.2255839
-5.32666	3.027285522	7.400115	-731.7522157	-1921215.44	24.41343492
-5.43998	3.033171847	7.015989	-731.7528504	-1921217.11	22.74705107
-5.5533	3.038753458	6.664321	-731.7534341	-1921218.64	21.21453097
-5.66663	3.044008799	6.340708	-731.7539711	-1921220.05	19.80451144
-5.77995	3.048918658	6.041434	-731.7544656	-1921221.35	18.50637498
-5.89327	3.053468324	5.764163	-731.7549212	-1921222.55	17.31024444
-6.00659	3.057647677	5.505781	-731.7553413	-1921223.65	16.20704347

-6.11991	3.061447169	5.263839	-731.7557293	-1921224.67	15.18837835
-6.23323	3.064868173	5.036346	-731.7560881	-1921225.61	14.24645659
-6.34655	3.067912462	4.821614	-731.7564204	-1921226.48	13.3740082
-6.45987	3.070592993	4.618754	-731.7567288	-1921227.29	12.56431975
-6.57319	3.072925647	4.426376	-731.7570156	-1921228.04	11.81128697
-6.68652	3.074934242	4.243785	-731.7572829	-1921228.75	11.10934641
-6.79984	3.07664435	4.069616	-731.7575328	-1921229.4	10.45348076
-6.91317	3.078087665	3.903216	-731.7577667	-1921230.02	9.839258162
-7.0265	3.079295107	3.743106	-731.7579863	-1921230.59	9.262753498
-7.13983	3.080299027	3.589578	-731.7581928	-1921231.14	8.720511609
-7.25316	3.081128608	3.442316	-731.7583875	-1921231.65	8.209476412
-7.3665	3.081812992	3.301553	-731.7585712	-1921232.13	7.726990903
-7.47983	3.082376015	3.167038	-731.758745	-1921232.59	7.270700007
-7.59317	3.082836449	3.039613	-731.7589096	-1921233.02	6.838463941
-7.70651	3.083212041	2.919501	-731.7590658	-1921233.43	6.428413352
-7.81985	3.083514792	2.806613	-731.7592142	-1921233.82	6.038925678
-7.93319	3.083754377	2.700516	-731.7593552	-1921234.19	5.66864091
-8.04653	3.083943265	2.601182	-731.7594894	-1921234.54	5.316406456
-8.15988	3.084087872	2.508121	-731.759617	-1921234.87	4.981237751
-8.27322	3.084194085	2.421361	-731.7597385	-1921235.19	4.662218497
-8.38656	3.084268693	2.340098	-731.7598542	-1921235.5	4.358519036
-8.49991	3.084315855	2.264371	-731.7599643	-1921235.79	4.069372721
-8.61326	3.084341068	2.193772	-731.7600692	-1921236.06	3.794044413
-8.7266	3.084348024	2.127992	-731.7601691	-1921236.32	3.531838353
-8.83995	3.084341164	2.066044	-731.7602642	-1921236.57	3.282140176
-8.9533	3.084322534	2.007657	-731.7603547	-1921236.81	3.044422154
-9.06664	3.084294369	1.951999	-731.7604409	-1921237.04	2.818190696
-9.17999	3.084257827	1.898887	-731.7605229	-1921237.25	2.602949581
-9.29334	3.084211867	1.848224	-731.7606008	-1921237.46	2.398210465
-9.40668	3.08415754	1.799189	-731.760675	-1921237.65	2.203524389
-9.52003	3.084095869	1.752183	-731.7607455	-1921237.84	2.018418763
-9.63338	3.084027512	1.707627	-731.7608125	-1921238.01	1.842465629
-9.74673	3.083952378	1.665067	-731.7608762	-1921238.18	1.675273789
-9.86007	3.083871403	1.624445	-731.7609367	-1921238.34	1.51651768
-9.97342	3.083785535	1.585863	-731.760994	-1921238.49	1.36594263
-10.08677	3.083695403	1.548998	-731.7610483	-1921238.63	1.2233491
-10.20012	3.083600506	1.513725	-731.7610997	-1921238.77	1.088595312
-10.31347	3.083502584	1.479656	-731.761148	-1921238.89	0.961536865
-10.42682	3.083399673	1.446503	-731.7611936	-1921239.01	0.842010977
-10.54017	3.083291598	1.414160	-731.7612363	-1921239.13	0.729873247
-10.65352	3.083177204	1.382048	-731.7612762	-1921239.23	0.625010777
-10.76687	3.083055034	1.350316	-731.7613134	-1921239.33	0.527347428
-10.88022	3.082926229	1.318620	-731.7613479	-1921239.42	0.436838567
-10.99358	3.082788658	1.287549	-731.7613797	-1921239.5	0.353452687
-11.10693	3.082643665	1.257146	-731.7614087	-1921239.58	0.277160908
-11.22028	3.08248767	1.227540	-731.7614351	-1921239.65	0.207918596

-11.33363	3.082324051	1.198678	-731.7614588	-1921239.71	0.14566274
-11.44698	3.082154765	1.170123	-731.7614799	-1921239.77	0.090325077
-11.56034	3.081979163	1.142070	-731.7614984	-1921239.81	0.041811087
-11.67369	3.081797958	1.114208	-731.7615143	-1921239.86	0

[a] E_{rel} refers to the energy of each species relative to the energy of **TP-($C_7H_7^+$)** (the lowest-energy structure).

6.4. Potential Energy Surface (PES) Scan

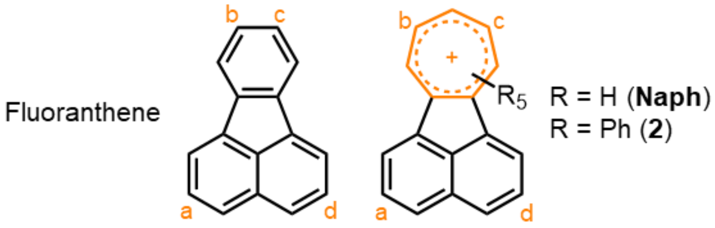

Fig. S74. Atom numbering for the torsional angle twisting θ(a–b–c–d) in fluoranthene, **Naph** (R=H), and **2** (R = Ph).

Table S22. PES scan of deforming naphthalene-annulated aromatic rings. The relative energy (E_{rel}) (compared to the lowest energy, planar conformer) is shown as a function of the torsional angle θ(a–b–c–d), which defines the end-to-end twist of the molecule.

	Fluoranthene	**Naph**	**2**
**θ(a–b–c–d) / °**		**E_{rel} / kJ·mol^{-1}**	
–25	38.33	27.99	5.48
–24	34.92	25.50	4.54
–23	31.72	23.16	3.73
–22	28.72	20.94	3.02
–21	25.91	18.85	2.41
–20	23.29	16.91	1.88
–19	20.84	15.10	1.41
–18	18.55	13.41	1.03
–17	16.42	11.84	0.73
–16	14.45	10.39	0.50
–15	12.62	9.05	0.32
–14	10.92	7.81	0.19
–13	9.36	6.69	1.01
–12	7.93	5.65	1.25
–11	6.63	4.71	1.12
–10	5.45	3.87	0.96
–9	4.39	3.11	0.83
–8	3.45	2.45	0.74
–7	2.62	1.87	0.65
–6	1.92	1.38	0.53
–5	1.32	0.96	0.41
–4	0.84	0.62	0.31

–3	0.47	0.35	0.21
–2	0.21	0.15	0.12
–1	0.05	0.04	0.05
0	0.00	0.00	0.00
1	0.05	0.04	0.05
2	0.21	0.15	0.12
3	0.47	0.35	0.21
4	0.84	0.62	0.31
5	1.32	0.96	0.41
6	1.92	1.38	0.53
7	2.62	1.87	0.65
8	3.45	2.45	0.74
9	4.39	3.11	0.83
10	5.45	3.87	0.96
11	6.63	4.71	1.12
12	7.93	5.65	1.25
13	9.36	6.69	1.01
14	10.92	7.81	0.19
15	12.62	9.05	0.32
16	14.45	10.39	0.50
17	16.42	11.84	0.73
18	18.55	13.41	1.03
19	20.84	15.10	1.41
20	23.29	16.91	1.88
21	25.91	18.85	2.41
22	28.72	20.94	3.02
23	31.72	23.16	3.73
24	34.92	25.50	4.54
25	38.33	27.99	5.48

6.5. Relative Stabilities of Tropylium Isomers

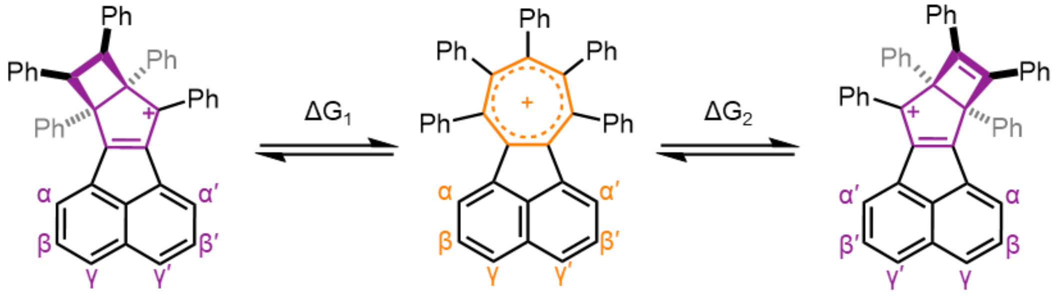

DT-1 **TP** **DT-2**

Fig. S75. Two possible isomers of asymmetrically substituted Dewar tropyliums (**DT-1** and **DT-2**) that form upon the rearrangement of the tropylium (**TP**) isomer.

Table S23. Calculated (B3LYP (GD3BJ) / 6-31G(d) – CH_2Cl_2) free energy differences of the **DT** isomers (**$\Delta G_{1,2}$**) relative to the **TP** isomer in a series of substituted analogues of **2**. The numbering pattern is outlined in **Fig. S63.** The number in bold denotes the lower energy **DT** isomer.

**Compound**	**ΔG_1 / kJ·mol^{-1}**	**ΔG_2 / kJ·mol^{-1}**	**Predicted Isomer**
**Naph**	219.5	—	**TP**
$(CH)_7^+$	218.6	—	**TP**
**Phen**	142.7	—	**TP**
β-NO_2	**79.9**	80.0	**TP**
β-Cl	78.9	**78.8**	**TP**
**2**	75.4	—	**TP**
β-Me	77.7	**74.7**	**TP**
β-OMe	**74.0**	75.5	**TP**
β-F	**72.9**	74.7	**TP**
γ-Cl	74.3	**72.5**	**TP**
γ-F	79.0	**70.9**	**TP**
γ-NO_2	**70.3**	81.3	**TP**
γ-OMe	83.9	**65.8**	**TP**
α-F	64.5	**62.3**	**TP**
α-NO_2	**59.2**	64.8	**TP**
α-NMe_2	**56.3**	64.5	**TP**
α-Cl	54.5	**54.4**	**TP**
α-^{t}Bu	**51.0**	53.9	**TP**
α-OMe	61.7	**48.9**	**TP**
α,β′-Me_2	51.6	**46.9**	**TP**
α-Me	51.8	**45.8**	**TP**
α,β-Me_2	52.8	**44.8**	**TP**
α,α′-Me_2	41.9	—	**TP**
α-Et	50.6	**41.8**	**TP**
α-^{i}Pr	53.7	**41.8**	**TP**
β,β′-Me_2	40.7	—	**TP**
α,α′-$(OMe)_2$	30.4	—	**TP**
α,α′-Cl_2	30.0	—	**TP**
**1**	26.1	—	**TP**
**3**	15.4	—	**TP**
α,α′-$^i Pr_2$	8.4	—	**TP**
**4**	–5.3	—	**DT**

6.6. Aromatic Stabilization Energy (ASE) Calculations

Aromatic stabilization energies (ASEs) of the parent cations of the compounds studied in this work were calculated using the indene–isoindene isomerization stabilization energy following a hyperhomodesmotic reaction,[10] as shown below.

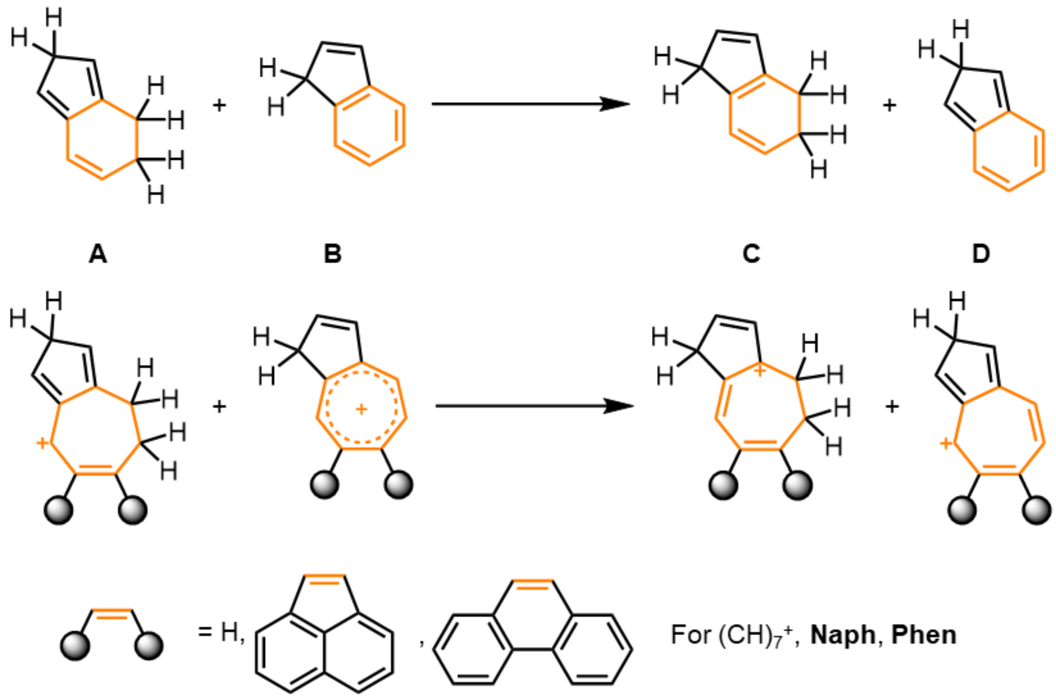

Fig. S76. Hyperhomodesmotic reactions used to predict the ASEs of $(CH)_6$, $(CH)_7^+$, **Naph**, and **Phen**.

DFT-optimized geometries were used for the calculation, and frequency calculations were performed to obtain the enthalpy change (ΔH) of the reaction, determined as:

$$\Delta H = [H(C) + H(D)] - [H(A) + H(B)]$$

The enthalpies of structures **A**–**D** for $(CH)_7^+$, **Naph**, and **Phen** are given in below.

Table S24. Calculated (B3LYP / 6-31G(d) GD3BJ – CH_2Cl_2) enthalpies (H) of the reagents and products in the hyperhomodesmotic reaction used to calculated aromatic stabilization energies (ASEs) of $(CH)_6$, $(CH)_7^+$, **Naph**, and **Phen**.

Structure	H / Hartree	$\Sigma(H)$ / kJ·mol^{-1}	ASE / kJ·mol^{-1}
$(CH)_6$-A	-348.802168	-1828533.043	–98.5
$(CH)_6$-B	-347.649191		
$(CH)_6$-C	-348.799555	-1828434.513	
$(CH)_6$-D	-347.614276		
$(CH)_7^+$-A	-387.297591	-2030682.617	–50.3

$(CH)_7^+$-B	-386.148464		
$(CH)_7^+$-C	-387.314453	-2030632.276	
$(CH)_7^+$-D	-386.112428		
**Naph**-A	-770.751992	-4044185.412	–55.1
**Naph**-B	-769.59667		
**Naph**-C	-770.762603	-4044130.266	
**Naph**-D	-769.565055		
**Phen**-A	-848.147453	-4450572.86	–50.7
**Phen**-B	-846.985992		
**Phen**-C	-848.162064	-4450522.196	
**Phen**-D	-846.952084		

Table S25. Calculated (M06-2X / 6-31G(d) – CH_2Cl_2) enthalpies (*H*) of the reagents and products in the hyperhomodesmotic reaction used to calculated aromatic stabilization energies (ASEs) of $(CH)_6$, $(CH)_7^+$, **Naph**, and **Phen**.

Structure	*H* / Hartree	Σ(*H*) / kJ·mol^{-1}	ASE / kJ·mol^{-1}
$(CH)_6$-A	-348.60346	-1827510.571	–104
$(CH)_6$-B	-347.45846		
$(CH)_6$-C	-348.601043	-1827406.349	
$(CH)_6$-D	-347.421181		
$(CH)_7^+$-A	-387.06396	-2029473.362	–56.0
$(CH)_7^+$-B	-385.921514		
$(CH)_7^+$-C	-387.080672	-2029417.323	
$(CH)_7^+$-D	-385.883458		
**Naph**-A	-770.324038	-4041955.467	–61.2
**Naph**-B	-769.175283		
**Naph**-C	-770.334106	-4041894.249	
**Naph**-D	-769.141898		
**Phen**-A	-847.675849	-4448113.651	–54.4
**Phen**-B	-846.520933		
**Phen**-C	-847.690569	-4448059.269	
**Phen**-D	-846.4855		

6.7. Nucleus-Independent Chemical Shifts (NICS) Values

NICS values were calculated 1 Å above and below the average plane generated by the seven C atoms forming the tropylium carbocycle using the Multiwfn software package.[11] Only the magnetic field in the *z*-direction (perpendicular to the plane of the studied ring) was considered. This corresponds to the *ZZ*-component of the magnetic shielding tensor (σ_{zz}).[12,13]

6.8. Anisotropy of the Induced Current (ACID) Plots

ACID plots were generated using version ACID-3.0.4. The magnetic field in the ACID plots was applied perpendicular to the plane of the tropylium and induced a clockwise, aromatic ring current in the conjugated system. An isovalue of 0.01 was used for all calculations. The molecular orbitals (determined from frequency calculations) used to visualize the plots are given below:

1: 150, 149, 146, 127, 102

2: 155, 154, 152 – 148, 144–142, 135, 131, 130, 123, 109, 98

3: 171 – 168, 166, 164–163, 160–157, 138, 128, 106, 105

4-TP: 162–160, 152–148, 131–129, 125, 124, 103

6.9. Electron Density of the Delocalized Bonds (EDDB^k)

EDDB^k calculations were run using the runEDDB program and MRAN 4.0.2. Population analysis was conducted using NBO v. 3.1. EDDB$_P^k$ plots of $(CH)_7^+$, **Naph**, and **Phen**, **1**–**3** and **4-TP** were used to assess the local aromaticity of the tropylium ring, as well as the global aromaticity of the larger polycyclic aromatic scaffold. The 'limit for uniformly delocalized electrons' output value was used for EDDB$_P^k$ analysis.

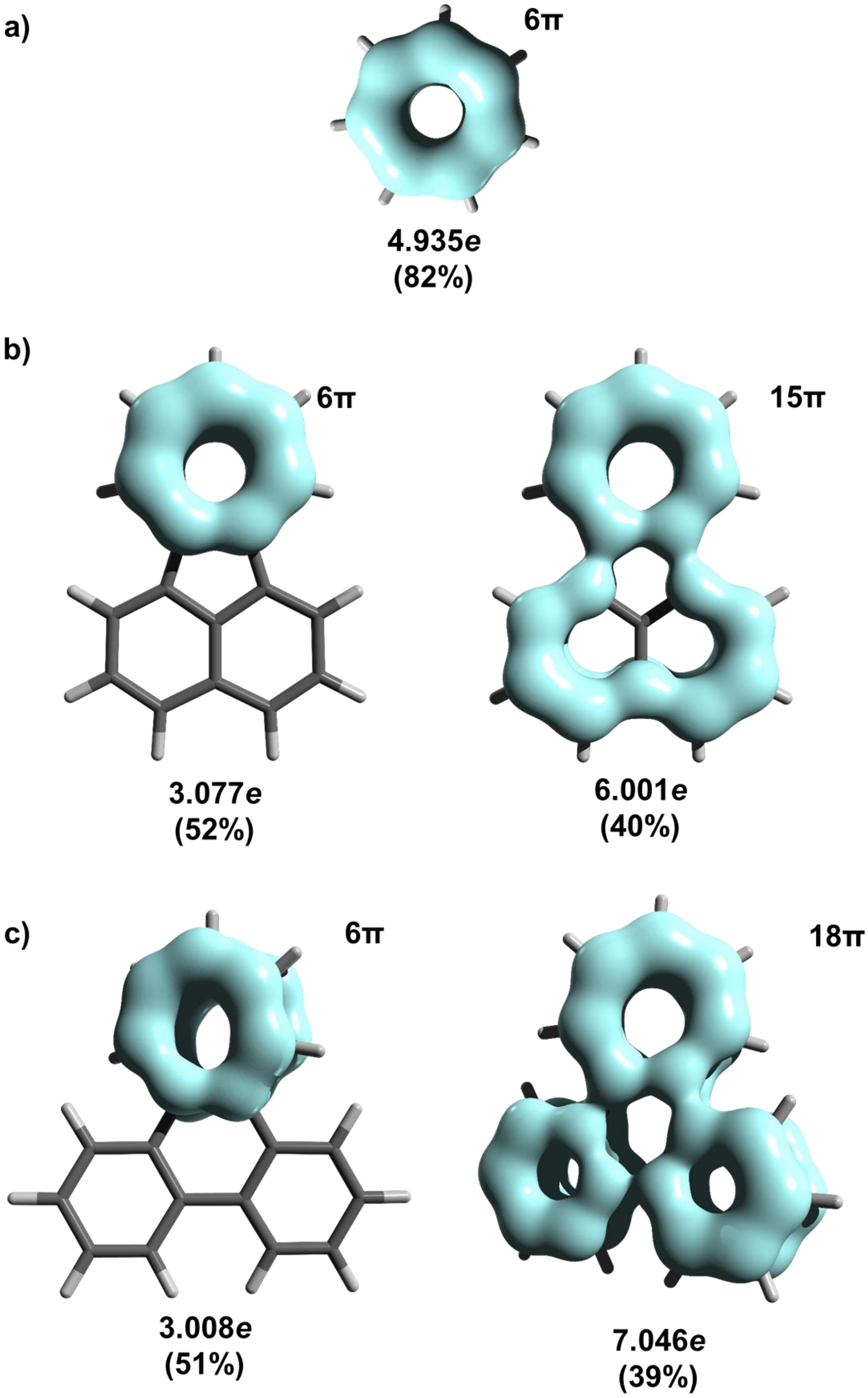

Fig. S77. π-EDDB$_P$^{k} plots and values of (a) $(CH)_7^+$, (b) **Naph**, and (c) **Phen**, showing delocalization pathways around the tropylium moiety (left) and the peripheral circuit (right). The effectiveness of delocalization (value in parentheses) was determined assuming the formal number of π-electrons in each structure. Isovalue = 0.008.

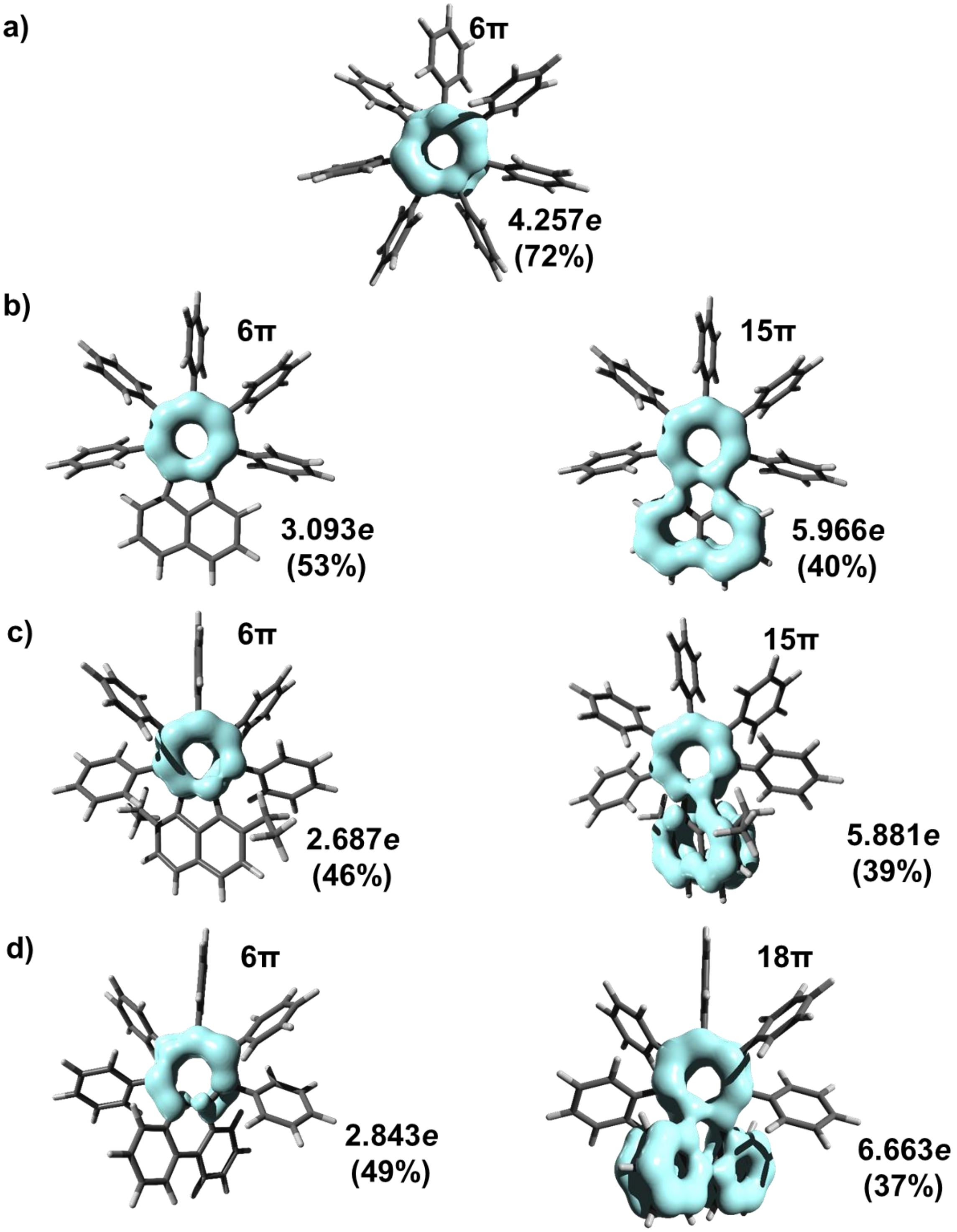

Fig. S78. ***π*-EDDB$_P$^{k}** plots and values of (a) **1**, (b) **2**, (c) **3**, and (d) **4-TP**, showing delocalization pathways around the tropylium moiety (left) and the peripheral circuit (right). The effectiveness of delocalization (value in parentheses) was determined assuming the formal number of *π*-electrons in each structure. Isovalue = 0.008.

6.10. Boltzmann Distribution of 4/4-TP

Using the ΔG of −5.3 kJ·mol^{-1} calculated for compound **4**, we calculated the probability of the higher energy tropylium state (**4-TP**) being populated. We applied the following equation to determine the Boltzmann distribution:

$$P_i = \frac{g_i e^{-\frac{\varepsilon_i}{kT}}}{\sum_i g_i e^{-\frac{\varepsilon_i}{kT}}}$$

where P_i is the probability of a state i, g_i is the degeneracy of a state i, ε_i is the energy of the state i relative to the lowest energy isomer (**4**), k is the Boltzmann constant (0.695 cm^{-1}·K^{-1}), and T is the temperature.

Two degenerate forms (i.e., g_i = 2) of the Dewar tropylium structure (**4** and **4′**) and two degenerate forms (*P* and *M* helical twist enantiomers) of **4-TP** are present at equilibrium.

The full set of parameters and the resulting probabilities are shown in Table S26 for **4**/**4-TP**.

Table S26. Probabilities of **4** and **4-TP** being occupied at rt.

	i	g_i	ε_i / J·mol^{-1}	ε_i / cm^{-1}	T / K	$g_i e^{-\frac{\varepsilon_i}{kT}}$	P_i
**4**	0	2	0	0	298	2	0.90
**4-TP**	1	2	5310	444	298	0.23	0.10

6.11. Optimized Geometries

Cartesian coordinates are given in Å, self-consistent field (SCF) free energies (G) and enthalpies (H) are given in Hartrees, and imaginary vibrational frequencies (v) are given in cm^{-1}.

$C_7H_7^+$-TP

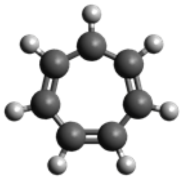

Table S27. Coordinates and energy for the optimized geometry of **$C_7H_7^+$-TP**.

G = –270.681568			
		**Coordinates / Å**	
**Atom**	**x**	**y**	**z**
C	-1.161811	-1.113980	-0.000209
C	-1.595468	0.213783	-0.000169
C	-0.827449	1.380472	-0.000028
C	0.146649	-1.602782	-0.000080
C	0.563472	1.507600	0.000132
C	1.344485	-0.884731	0.000133
C	1.529929	0.499481	0.000223
H	-1.945718	-1.865969	-0.000346
H	-2.671775	0.358793	-0.000263
H	-1.385483	2.312212	-0.000053
H	0.245933	-2.684276	-0.000149
H	0.944088	2.524683	0.000196
H	2.251946	-1.481216	0.000225
H	2.562166	0.836721	0.000373

$C_7H_7^+$-TS1

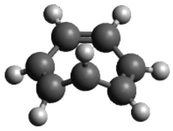

Table S28. Coordinates and energy for the optimized geometry of **$C_7H_7^+$-TS1**.

G = –270.566706<br>ν = –881.00			
		**Coordinates / Å**	
**Atom**	**x**	**y**	**z**
C	-0.076737	1.450806	0.137104
C	1.215599	0.888418	-0.297931
C	1.627959	-0.406240	-0.211263
C	0.636893	-1.055894	0.600079
C	-0.559962	-1.397987	-0.106546
C	-1.555044	-0.482164	-0.244852
C	-1.319684	0.879910	0.147412
H	-0.021048	2.505853	0.389692
H	1.970874	1.620861	-0.576624
H	2.627119	-0.761599	-0.438411
H	0.730205	-1.124879	1.689031
H	-0.498520	-2.282000	-0.747417

H	-2.448603	-0.739339	-0.805258
H	-2.174166	1.520007	0.344965

$C_7H_7^+$-MT

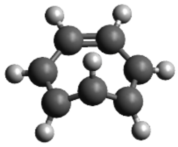

Table S29. Coordinates and energy for the optimized geometry of **$C_7H_7^+$-MT**.

G = –270.586913			
	Coordinates / Å		
Atom	x	y	z
C	0.711100	1.297106	0.086123
C	1.619747	0.236621	-0.196483
C	1.216863	-1.095759	-0.100699
C	0.000032	-1.094771	0.604488
C	-1.216784	-1.095812	-0.100759
H	-1.616672	-1.887583	-0.726216
C	-1.619771	0.236534	-0.196459
C	-0.711177	1.297068	0.086149
H	1.149205	2.291153	0.114398
H	2.554683	0.493986	-0.687503
H	1.616783	-1.887514	-0.726155
H	0.000000	-0.480920	1.504312
H	-2.554727	0.493862	-0.687462
H	-1.149342	2.291089	0.114461

$C_7H_7^+$-TS2

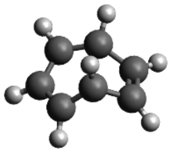

Table S30. Coordinates and energy for the optimized geometry of **$C_7H_7^+$-TS2**.

G = –270.571412<br>*v* = –407.48			
	Coordinates / Å		
Atom	x	y	z
C	0.193772	1.204739	0.236730
C	1.430445	0.657522	-0.427852
C	1.598198	-0.641806	-0.163728
C	0.391838	-0.801370	0.688370
C	-0.748751	-1.259817	-0.035105
H	-0.734821	-2.228586	-0.531097
C	-1.681417	-0.277903	-0.296988
C	-1.186899	1.005143	-0.000551
H	0.409225	2.103519	0.819452
H	2.118843	1.347854	-0.908090

H	2.332900	-1.370286	-0.479466
H	0.387785	-0.572053	1.752731
H	-2.639730	-0.453193	-0.772619
H	-1.857319	1.853703	0.113828

$C_7H_7^+$-DT

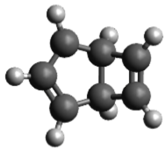

Table S31. Coordinates and energy for the optimized geometry of **$C_7H_7^+$-DT**.

G = –270.598324			
	**Coordinates / Å**		
**Atom**	**x**	**y**	**z**
C	0.972224	1.131988	-0.026765
H	1.271108	2.150139	-0.257231
C	1.717464	0.000266	-0.349430
H	2.679399	0.000404	-0.845824
C	0.972788	-1.131692	-0.026309
H	1.272074	-2.149765	-0.256597
C	-0.307762	0.774307	0.592357
C	-0.307542	-0.774411	0.592321
H	-0.540965	-1.305140	1.521176
H	-0.541046	1.304940	1.521295
C	-1.517575	0.662572	-0.391556
H	-2.109070	1.452466	-0.836126
C	-1.516901	-0.663021	-0.392172
H	-2.107675	-1.453098	-0.837376

Naph-TP

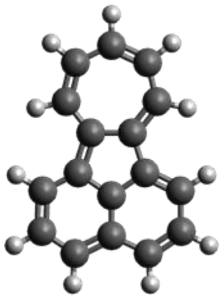

Table S32. Coordinates and energy for the optimized geometry of **Naph-TP**.

G = –654.146490			
	**Coordinates / Å**		
**Atom**	**x**	**y**	**z**

C	0.000000	0.726674	1.025022
C	0.000000	-0.726674	1.025022
C	0.000000	-1.584509	2.120280
C	0.000000	1.584509	2.120280
C	0.000000	-1.260517	3.478181
C	0.000000	1.260517	3.478181
C	0.000000	0.000000	4.080375
H	0.000000	-2.646475	1.889614
H	0.000000	2.646475	1.889614
H	0.000000	-2.107105	4.158715
H	0.000000	2.107105	4.158715
H	0.000000	0.000000	5.166854
C	0.000000	-1.166121	-0.367909
C	0.000000	0.000000	-1.162830
C	0.000000	-2.402709	-0.998491
C	0.000000	0.000000	-2.566148
C	0.000000	-2.434744	-2.414081
H	0.000000	-3.335232	-0.442269
C	0.000000	-1.278384	-3.186904
H	0.000000	-3.399787	-2.910000
H	0.000000	-1.353253	-4.270274
C	0.000000	1.166121	-0.367909
C	0.000000	2.402709	-0.998491
C	0.000000	2.434744	-2.414081
H	0.000000	3.335232	-0.442269
C	0.000000	1.278384	-3.186904
H	0.000000	3.399787	-2.910000
H	0.000000	1.353253	-4.270274

Naph-TS1

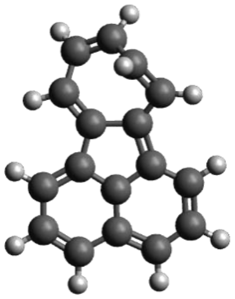

Table S33. Coordinates and energy for the optimized geometry of **Naph-TS1**.

G = –654.027867<br>v = –923.44			
		**Coordinates / Å**	
**Atom**	**x**	**y**	**z**
C	-3.062905	1.382781	0.091134
H	-4.141878	1.504708	0.092555

C	-2.239550	2.494828	0.197882
C	-0.250499	1.145043	0.084826
C	-1.097699	0.012347	-0.010014
H	-2.690996	3.477758	0.285314
C	-0.825568	2.399153	0.193975
C	1.133472	0.679404	-0.024264
C	-0.372634	-1.199211	-0.096498
C	-2.502058	0.080890	-0.021786
H	-0.225933	3.299903	0.276743
C	1.053392	-0.852252	-0.034757
C	2.203500	1.512243	-0.264159
C	-1.059775	-2.393852	-0.187778
C	-3.186739	-1.160889	-0.129091
C	2.056507	-1.720268	0.298500
C	3.099473	-0.899002	0.839906
C	3.512656	1.032644	-0.568711
H	-0.549038	-3.349348	-0.252197
C	-2.477385	-2.349908	-0.211602
H	-4.272325	-1.176086	-0.141789
C	3.876367	-0.211736	-0.142109
H	-3.022111	-3.284750	-0.294183
H	1.902285	-2.779322	0.476203
H	4.543547	-0.831780	-0.745458
H	3.185631	-0.703439	1.912639
H	1.994072	2.569573	-0.397878
H	4.113408	1.559481	-1.304675

Naph-MT

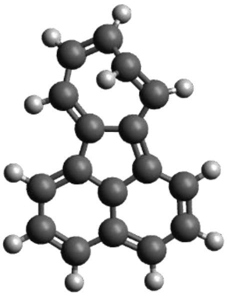

Table S34. Coordinates and energy for the optimized geometry of **Naph-MT**.

G = –654.047722			
		**Coordinates / Å**	
**Atom**	**x**	**y**	**z**
C	-3.060977	1.423633	-0.030289
H	-4.134962	1.572294	-0.090940
C	-2.215190	2.521503	0.080144

C	-0.259633	1.113835	0.150610
C	-1.130315	0.005288	0.035615
H	-2.644446	3.517555	0.103376
C	-0.808843	2.389035	0.167764
C	1.105881	0.586314	0.182157
C	-0.425989	-1.219670	0.022848
C	-2.527513	0.106922	-0.059428
H	-0.193676	3.278515	0.260839
C	0.981935	-0.887903	0.210970
C	2.231264	1.398476	-0.134283
C	-1.142172	-2.402536	-0.085545
C	-3.237390	-1.118720	-0.176999
C	2.120436	-1.695463	0.239676
C	3.190618	-0.870420	0.642770
C	3.554292	1.039992	-0.504283
H	-0.652070	-3.370909	-0.096033
C	-2.552890	-2.328164	-0.186535
H	-4.319679	-1.108877	-0.262640
C	4.035150	-0.259401	-0.295520
H	-3.115522	-3.251607	-0.274756
H	2.181029	-2.668691	-0.237577
H	4.726303	-0.747180	-0.976471
H	2.930600	-0.211848	1.469429
H	1.980028	2.442990	-0.304114
H	4.090422	1.731421	-1.149151

Naph-TS2

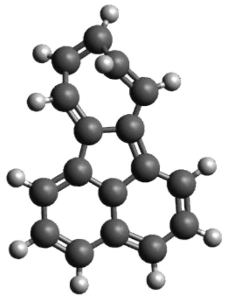

Table S35. Coordinates and energy for the optimized geometry of **Naph-TS2**.

G = –654.026160<br>*v* = –491.07			
		**Coordinates / Å**	
**Atom**	**x**	**y**	**z**
C	3.061527	1.449834	0.039367
H	4.133733	1.612768	0.098016
C	2.203362	2.541200	-0.058530
C	0.269063	1.110691	-0.133437
C	1.155155	0.000363	-0.027875

H	2.623554	3.541196	-0.073363
C	0.800014	2.394440	-0.138253
C	-1.067883	0.548346	-0.127503
C	0.472819	-1.242100	-0.009104
C	2.550596	0.124527	0.063585
H	0.168170	3.273874	-0.211605
C	-0.939821	-0.895935	-0.167023
C	-2.340354	1.176873	-0.077314
C	1.209912	-2.407321	0.084762
C	3.285159	-1.089675	0.168782
C	-2.139791	-1.536851	-0.353301
C	-3.204895	-0.636039	-0.695367
C	-3.553556	0.885806	0.757051
H	0.735699	-3.383553	0.097551
C	2.623652	-2.308057	0.176299
H	4.367645	-1.058716	0.245598
C	-4.119099	-0.277667	0.413600
H	3.201879	-3.222556	0.257381
H	-2.340128	-2.565259	-0.058893
H	-4.924663	-0.851842	0.853059
H	-3.313627	-0.205432	-1.688502
H	-2.334829	2.195821	-0.474661
H	-3.912580	1.633087	1.460973

Naph-DT

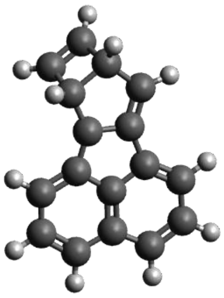

Table S36. Coordinates and energy for the optimized geometry of **Naph-DT**.

G = –731.540079			
		**Coordinates / Å**	
**Atom**	**x**	**y**	**z**
C	2.931707	1.559028	0.118548
H	3.985483	1.789161	0.246228
C	2.017884	2.610368	-0.027679
C	0.184041	1.064860	-0.217285
C	1.133051	-0.001736	-0.065176
H	2.388392	3.629350	-0.010337
C	0.642813	2.388516	-0.196587

C	-1.078681	0.440275	-0.358745
C	0.523343	-1.276995	-0.095256
C	2.510040	0.205878	0.103932
H	-0.039050	3.224721	-0.310095
C	-0.907384	-0.996757	-0.281922
C	-2.507746	0.785840	-0.488121
C	1.324620	-2.393402	0.044170
C	3.315779	-0.960942	0.244024
C	-2.124238	-1.616158	-0.323850
C	-3.222772	-0.615255	-0.468068
C	-3.273724	1.055182	0.828435
H	0.910934	-3.396446	0.028808
C	2.726031	-2.212914	0.212273
H	4.388905	-0.864615	0.376026
C	-3.885045	-0.128011	0.840250
H	3.349135	-3.094307	0.320805
H	-2.297329	-2.682260	-0.217856
H	-4.601082	-0.618256	1.488980
H	-3.920130	-0.834164	-1.283696
H	-2.748062	1.464291	-1.312269
H	-3.275504	1.935856	1.459751

Phen-TP

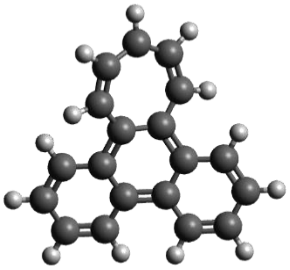

Table S37. Coordinates and energy for the optimized geometry of **Phen-TP**.

	G = –731.163764		
		**Coordinates / Å**	
**Atom**	**x**	**y**	**z**
C	1.004952	-0.706549	-0.134784
C	-0.256630	-1.434718	-0.042750
C	-0.273645	-2.846844	0.044880
C	-1.459694	-3.543489	0.180172
C	-2.672773	-2.844202	0.245852
C	-2.680635	-1.460750	0.184586
C	-1.487509	-0.728300	0.038850
C	-1.487508	0.728301	-0.038850
C	-0.256629	1.434718	0.042750
C	-0.273642	2.846844	-0.044880

C	-1.459691	3.543490	-0.180172
C	-2.672770	2.844204	-0.245852
C	-2.680634	1.460753	-0.184587
C	1.004953	0.706548	0.134784
C	2.146452	1.423534	0.551843
C	3.502197	1.114081	0.548251
C	4.132075	-0.000001	-0.000001
H	5.217963	-0.000001	-0.000001
C	3.502197	-1.114083	-0.548251
C	2.146451	-1.423536	-0.551842
H	0.653308	-3.405979	0.060199
H	-1.444705	-4.625287	0.258604
H	-3.607214	-3.382944	0.365114
H	-3.626833	-0.942803	0.272589
H	0.653311	3.405978	-0.060198
H	-1.444700	4.625288	-0.258603
H	-3.607210	3.382948	-0.365114
H	-3.626832	0.942806	-0.272590
H	1.933052	2.398389	0.972092
H	4.150848	1.874014	0.973631
H	4.150847	-1.874017	-0.973632
H	1.933051	-2.398392	-0.972090

Phen-TS1

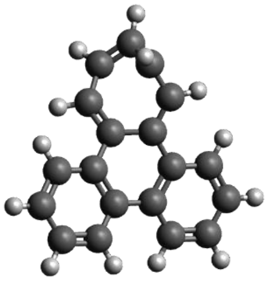

Table S38. Coordinates and energy for the optimized geometry of **Phen-TS1**.

	$G = -731.426826$		
	$v = -1026.93$		
		**Coordinates / Å**	
**Atom**	**x**	**y**	**z**
C	2.520337	1.646999	-0.296227
H	3.495082	1.202244	-0.453511
C	2.409962	3.031471	-0.326977
C	1.402130	0.826806	-0.073700
H	3.292295	3.635078	-0.514377
C	1.168742	3.646291	-0.122995
C	0.140173	1.456347	0.111605
C	1.523475	-0.639457	0.021420

H	1.077946	4.726881	-0.152450
C	0.049538	2.860794	0.096032
C	-1.028743	0.617349	0.352276
C	0.357142	-1.442930	0.026949
C	2.770829	-1.279708	0.105353
H	-0.911922	3.344239	0.232869
C	-0.950074	-0.786160	-0.053362
C	-2.257584	1.103208	0.721511
C	0.466319	-2.839339	0.115580
C	2.866882	-2.664840	0.183204
H	3.682445	-0.695129	0.127787
C	-2.023202	-1.389229	-0.663627
C	-3.379386	-0.904545	-0.932720
C	-3.406138	0.291021	0.946648
H	-0.432502	-3.445258	0.162985
C	1.711347	-3.451166	0.188612
H	3.844589	-3.130827	0.253815
C	-4.128586	0.025393	-0.256101
H	1.781409	-4.531076	0.268719
H	-1.841765	-2.366277	-1.098956
H	-5.187032	0.178986	-0.436695
H	-3.929281	-1.505183	-1.655575
H	-2.518356	2.144053	0.510319
H	-3.631881	-0.207575	1.894188

Phen-MT

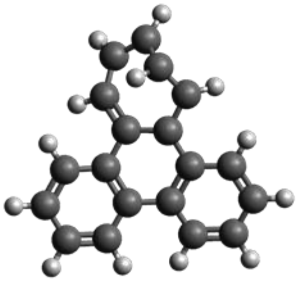

Table S39. Coordinates and energy for the optimized geometry of **Phen-MT**.

G = – 731.451809			
		**Coordinates / Å**	
**Atom**	**x**	**y**	**z**
C	2.813563	-1.115897	0.343821
H	3.662931	-0.479317	0.556391
C	3.002765	-2.487126	0.314999
C	1.545252	-0.547439	0.111798
H	3.990058	-2.896427	0.503646
C	1.929618	-3.346965	0.027460
C	0.448529	-1.424078	-0.122315

C	1.341644	0.895276	0.029123
H	2.084510	-4.419703	-0.018577
C	0.674483	-2.818175	-0.196623
C	-0.880712	-0.887564	-0.307473
C	0.024596	1.421054	-0.081401
C	2.425802	1.796494	0.025914
H	-0.146568	-3.485186	-0.432785
C	-1.127001	0.522321	-0.061371
C	-2.018880	-1.728511	-0.455451
C	-0.150251	2.814648	-0.230151
C	2.229012	3.161231	-0.097449
H	3.439572	1.423550	0.095715
C	-2.344823	1.071836	0.482997
C	-3.621325	0.527819	0.777990
C	-3.122198	-0.977275	-0.814162
H	-1.143558	3.218808	-0.389181
C	0.932108	3.675492	-0.238327
H	3.084171	3.829377	-0.104862
C	-4.033407	-0.685248	0.234932
H	0.775697	4.741144	-0.369034
H	-2.201345	2.060965	0.904373
H	-4.743355	-1.355812	0.708408
H	-4.199160	1.012069	1.560204
H	-2.081019	-2.701970	0.021546
H	-2.934575	-0.154845	-1.501720

Phen-TS3

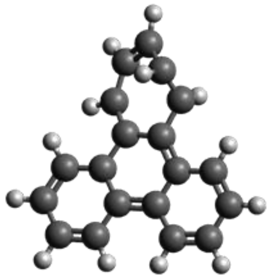

Table S40. Coordinates and energy for the optimized geometry of **Phen-TS3**.

	G = –731.440694<br>*v* = –267.71		
		**Coordinates / Å**	
**Atom**	**x**	**y**	**z**
C	-2.819562	1.247870	0.082523
H	-3.736450	0.674858	0.120814
C	-2.908258	2.632123	0.076798
C	-1.572240	0.600090	0.042850
H	-3.888787	3.096396	0.111656
C	-1.756893	3.448333	0.026899

C	-0.402193	1.433307	0.003426
C	-1.423361	-0.847427	0.014456
H	-1.854801	4.528140	0.021376
C	-0.520175	2.853956	-0.010019
C	0.868957	0.850303	-0.015261
C	-0.124183	-1.435352	0.007228
C	-2.552472	-1.693682	-0.033760
H	0.389395	3.444754	-0.048407
C	1.063535	-0.568831	0.078667
C	2.162964	1.549803	0.109168
C	-0.018003	-2.834569	-0.103082
C	-2.420189	-3.068655	-0.104138
H	-3.548053	-1.268999	-0.028223
C	2.321247	-1.140335	0.404627
C	3.712918	-0.718268	0.423913
C	3.116049	1.186567	-0.756189
H	0.954243	-3.304959	-0.187906
C	-1.143324	-3.642422	-0.148756
H	-3.304480	-3.696114	-0.142216
C	4.217272	0.416982	-0.153149
H	-1.029112	-4.718107	-0.233933
H	2.239495	-2.173304	0.727849
H	5.284787	0.602250	-0.233449
H	4.398251	-1.451860	0.840950
H	2.447013	1.768656	1.141876
H	2.835961	0.879532	-1.767599

Phen- MT′

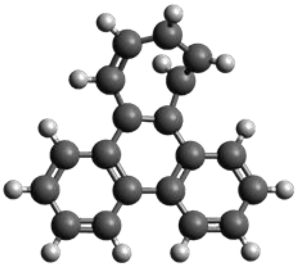

Table S41. Coordinates and energy for the optimized geometry of **Phen- MT′**.

G = –731.460396			
		**Coordinates / Å**	
**Atom**	**x**	**y**	**z**
C	2.112178	2.094707	0.312936
H	3.141714	1.877026	0.566118
C	1.707314	3.420543	0.278741
C	1.209898	1.049094	0.036319
H	2.428043	4.201202	0.500216
C	0.385156	3.767257	-0.052796

C	-0.132704	1.410733	-0.267257
C	1.602176	-0.360391	0.024285
H	0.086594	4.809236	-0.093297
C	-0.522407	2.768492	-0.337503
C	-1.074809	0.380181	-0.528560
C	0.627831	-1.384519	-0.171017
C	2.947473	-0.743401	0.202115
H	-1.541589	3.010834	-0.624224
C	-0.767987	-1.008364	-0.375341
C	-2.485052	0.446813	-0.701777
C	1.040076	-2.734646	-0.172046
C	3.329022	-2.073556	0.187708
H	3.710556	0.011531	0.341691
C	-1.833045	-1.948781	-0.176656
C	-3.163426	-1.795470	0.291908
C	-3.363331	0.632735	0.372272
H	0.319246	-3.527584	-0.328378
C	2.369391	-3.077018	-0.005194
H	4.373320	-2.335742	0.323063
C	-3.841742	-0.627234	0.739495
H	2.663799	-4.121162	-0.023870
H	-1.540527	-2.991318	-0.226121
H	-4.578802	-0.740836	1.530544
H	-3.657493	-2.738972	0.509068
H	-2.855637	-0.254845	-1.445945
H	-3.425300	1.497583	1.025359

Phen-TS2

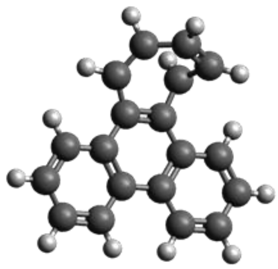

Table S42. Coordinates and energy for the optimized geometry of **Phen-TS2**.

	G = –731.444793<br>*v* = –438.72		
		Coordinates / Å	
Atom	x	y	z
C	1.460940	2.544260	0.218679
H	2.520933	2.630374	0.419544
C	0.701374	3.702827	0.176759
C	0.879473	1.277933	0.010903
H	1.183122	4.660546	0.346065
C	-0.676268	3.654959	-0.102103

C	-0.524436	1.238350	-0.247273
C	1.663386	0.041557	0.023697
H	-1.256634	4.569843	-0.153363
C	-1.275991	2.434466	-0.330259
C	-1.137587	-0.029994	-0.470483
C	1.019719	-1.216491	-0.151219
C	3.060890	0.047435	0.213731
H	-2.330580	2.383572	-0.584057
C	-0.416499	-1.232656	-0.335734
C	-2.553402	-0.266320	-0.628724
C	1.774688	-2.405140	-0.117410
C	3.789776	-1.129787	0.226882
H	3.588328	0.983324	0.348031
C	-1.261770	-2.363647	-0.261416
C	-2.644811	-2.277810	0.003646
C	-3.379467	-0.124421	0.591706
H	1.281677	-3.363321	-0.238080
C	3.146131	-2.365059	0.058936
H	4.864815	-1.093020	0.370908
C	-3.426105	-1.411405	0.947178
H	3.718765	-3.286576	0.071910
H	-0.868149	-3.352203	-0.483502
H	-3.988793	-1.870974	1.756057
H	-3.202387	-3.153964	-0.335048
H	-2.987123	-0.596224	-1.569938
H	-3.724200	0.774260	1.086487

Phen-DT

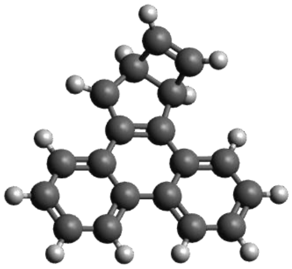

Table S43. Coordinates and energy for the optimized geometry of **Phen-DT**.

G = –731.485734			
	**Coordinates / Å**		
**Atom**	**x**	**y**	**z**
C	1.594358	2.478564	0.135064
H	2.665361	2.530654	0.279283
C	0.876487	3.665953	0.104908
C	0.955170	1.235894	-0.012271
H	1.407605	4.605139	0.223181
C	-0.520805	3.678766	-0.076108
C	-0.473025	1.253236	-0.196244

C	1.687602	-0.031718	0.022526
H	-1.059677	4.619321	-0.097726
C	-1.185366	2.484489	-0.226883
C	-1.149754	0.032823	-0.335350
C	0.988956	-1.264412	-0.101459
C	3.088416	-0.076627	0.178320
H	-2.259688	2.467273	-0.373526
C	-0.453489	-1.215790	-0.271440
C	-2.613967	-0.200897	-0.474656
C	1.690972	-2.478895	-0.069273
C	3.767220	-1.284264	0.210414
H	3.658295	0.838870	0.273780
C	-1.370451	-2.246310	-0.321634
C	-2.755546	-1.752868	-0.464918
C	-3.427944	-0.231633	0.837104
H	1.153274	-3.415689	-0.171272
C	3.068433	-2.492749	0.086876
H	4.845612	-1.290196	0.330491
C	-3.550600	-1.557701	0.851655
H	3.601730	-3.437367	0.110781
H	-1.126871	-3.298639	-0.220344
H	-4.023272	-2.287634	1.497611
H	-3.318601	-2.224092	-1.277488
H	-3.083529	0.334325	-1.304917
H	-3.760238	0.582876	1.470365

$(CH)_6$-A

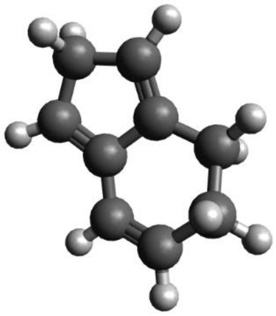

Table S44. Coordinates and energy for the optimized geometry of **$(CH)_6$-A**.

G = –348.642723, *H* = –348.60346			
		Coordinates / Å	
Atom	x	y	z
C	0.306672	-0.723316	-0.085954
C	1.563024	-1.191811	0.045583
C	2.526551	-0.031799	0.122482
C	1.625613	1.172291	0.020920
C	0.340309	0.757158	-0.086972

C	-0.909097	1.498770	-0.145450
C	-2.081380	0.863531	0.040007
C	-2.172561	-0.617626	0.326641
C	-1.007254	-1.419429	-0.293163
H	1.868524	-2.232362	0.068321
H	3.272041	-0.065542	-0.687571
H	3.108094	-0.029435	1.057610
H	1.974236	2.198015	0.067756
H	-0.877088	2.574338	-0.305075
H	-3.010092	1.430055	0.029622
H	-3.127640	-1.013860	-0.036631
H	-2.180814	-0.766377	1.417654
H	-1.190752	-1.499388	-1.374820
H	-0.987777	-2.442061	0.098583

(CH)$_6$-B

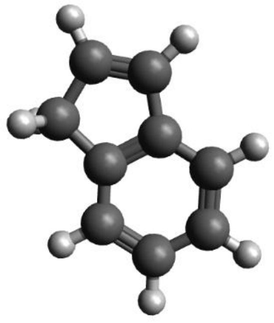

Table S45. Coordinates and energy for the optimized geometry of **(CH)$_6$-B**.

G = –347.496228, *H* = –347.45846			
		**Coordinates / Å**	
**Atom**	**x**	**y**	**z**
C	0.212177	0.723279	0.000000
C	1.599927	1.197978	0.000001
C	2.440203	0.143797	-0.000001
C	1.668486	-1.155672	0.000000
C	0.230344	-0.689908	0.000000
C	-0.955527	-1.411567	0.000000
C	-2.171791	-0.714512	0.000000
C	-2.191208	0.684563	0.000000
C	-0.999557	1.416446	0.000000
H	1.883939	2.245219	0.000002
H	3.523396	0.189388	-0.000002
H	1.905657	-1.773826	0.877723
H	1.905656	-1.773826	-0.877722
H	-0.946624	-2.498765	0.000000
H	-3.108020	-1.265581	0.000000
H	-3.143499	1.207835	0.000000
H	-1.018825	2.503131	0.000000

$(CH)_6$-C

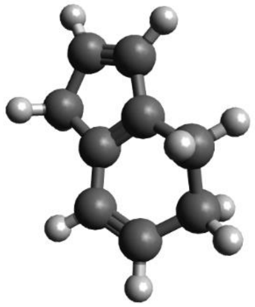

Table S46. Coordinates and energy for the optimized geometry of **$(CH)_6$-C**.

G = –348.640488, H = –348.601043			
		**Coordinates / Å**	
**Atom**	**x**	**y**	**z**
C	-0.282553	-0.679243	-0.064729
C	-1.649218	-1.193040	0.009925
C	-2.516601	-0.157400	0.091433
C	-1.746938	1.139875	0.061205
C	-0.314101	0.682797	-0.036692
C	0.897893	1.475026	-0.142648
C	2.096821	0.872459	-0.014470
C	2.191902	-0.603252	0.312481
C	1.012050	-1.421950	-0.251303
H	-1.906601	-2.247850	0.006732
H	-3.595500	-0.217406	0.173428
H	-1.935942	1.752798	0.955016
H	-2.036712	1.765070	-0.796902
H	0.824079	2.546451	-0.316578
H	3.016587	1.449692	-0.067973
H	2.210434	-0.710458	1.410563
H	3.139254	-1.019036	-0.047490
H	0.982853	-2.410669	0.220449
H	1.166012	-1.600228	-1.328465

$(CH)_6$-D

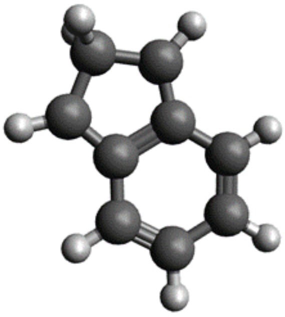

Table S47. Coordinates and energy for the optimized geometry of **$(CH)_6$-D**.

G = –347.459316, *H* = –347.421181			
		**Coordinates / Å**	
**Atom**	**x**	**y**	**z**
C	-0.253859	0.742058	-0.000002
C	-1.541984	1.186735	-0.000001
C	-2.461517	0.000000	0.000000
C	-1.541984	-1.186735	0.000000
C	-0.253859	-0.742058	-0.000002
C	1.007350	-1.442255	0.000000
C	2.163656	-0.726009	0.000001
C	2.163656	0.726009	0.000001
C	1.007349	1.442255	0.000000
H	-1.875441	2.217608	0.000001
H	-3.132002	0.000000	-0.874485
H	-3.131975	0.000000	0.874507
H	-1.875441	-2.217608	0.000001
H	1.019445	-2.528990	0.000000
H	3.121556	-1.238771	0.000001
H	3.121556	1.238771	0.000001
H	1.019445	2.528990	0.000000

$(CH)_7^+$-A

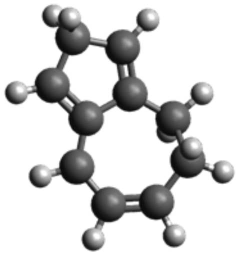

Table S48. Coordinates and energy for the optimized geometry of **$(CH)_7^+$-A**.

G = –387.339546, *H* = –387.297591			
		**Coordinates / Å**	
**Atom**	**x**	**y**	**z**
C	-0.564541	-0.746317	0.157931
C	-1.832302	-1.169972	-0.016986
C	-2.766045	-0.001453	-0.193179
C	-1.861057	1.169785	-0.086520
C	-0.562151	0.729378	0.099697
C	0.517615	1.629991	0.154008
H	0.248400	2.681754	0.218544
C	1.897809	1.354501	0.094449

C	2.494472	0.143764	-0.169405
C	1.884963	-1.193804	-0.400969
C	0.653702	-1.560134	0.452402
H	-2.163684	-2.201160	-0.011895
H	-3.559426	0.045179	0.569701
H	-3.299458	0.002474	-1.156703
H	-2.170419	2.204998	-0.176604
H	2.554795	2.212990	0.190553
H	3.578582	0.176158	-0.260387
H	2.662884	-1.950046	-0.259782
H	1.620299	-1.242647	-1.470601
H	0.922727	-1.444662	1.510333
H	0.430509	-2.619466	0.298264

$(CH)_7^+$-B

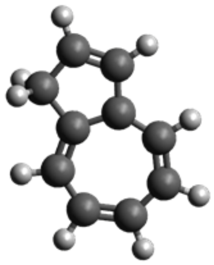

Table S49. Coordinates and energy for the optimized geometry of **$(CH)_7^+$-B**.

G = –386.188713, *H* = –386.148464			
	Coordinates / Å		
**Atom**	**x**	**y**	**z**
C	0.494666	0.733117	0.000000
C	-0.616594	1.591206	0.000000
C	-1.961358	1.249239	0.000000
C	-2.551546	-0.025161	0.000000
C	-1.935661	-1.270950	0.000000
C	-0.564888	-1.573431	0.000000
C	0.503287	-0.694468	0.000000
C	1.941465	-1.145863	0.000000
C	2.709544	0.135730	0.000000
C	1.869837	1.196283	0.000000
H	-0.392938	2.654502	0.000000
H	-2.655794	2.084365	0.000001
H	-3.637643	-0.035810	0.000000
H	-2.601702	-2.128708	0.000000
H	-0.314992	-2.631560	0.000000
H	2.165855	-1.770167	-0.876856
H	2.165855	-1.770166	0.876857
H	3.791711	0.180261	0.000000
H	2.147135	2.243071	0.000000

$(CH)_7^+$-C

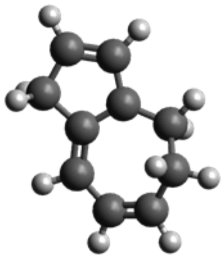

Table S50. Coordinates and energy for the optimized geometry of **$(CH)_7^+$-C**.

G = –387.356263 *H* = –387.314453			
		**Coordinates / Å**	
**Atom**	**x**	**y**	**z**
C	-0.539175	-0.697200	0.132832
C	0.663058	-1.542662	0.372095
C	1.878121	-1.149584	-0.473109
C	2.525737	0.141296	-0.077472
C	1.903091	1.317799	0.201626
C	0.502129	1.587321	0.102146
C	-0.557952	0.710722	-0.012547
C	-1.997353	1.142337	-0.170235
C	-2.740399	-0.142775	-0.102319
C	-1.878248	-1.188372	0.087163
H	0.413302	-2.591746	0.189440
H	0.910745	-1.469717	1.443806
H	1.589737	-1.079010	-1.535136
H	2.616658	-1.953320	-0.415358
H	3.613174	0.151500	-0.071255
H	2.527472	2.177767	0.423137
H	0.239875	2.644150	0.111636
H	-2.185494	1.671958	-1.115023
H	-2.316025	1.828841	0.627677
H	-3.817097	-0.220226	-0.198552
H	-2.146398	-2.233490	0.178545

$(CH)_7^+$-D

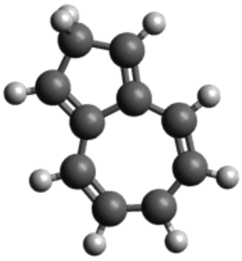

Table S51. Coordinates and energy for the optimized geometry of **$(CH)_7^+$-D**.

G = –386.153028, *H* = –386.112428			
		**Coordinates / Å**	
**Atom**	**x**	**y**	**z**

C	-0.516126	-0.746460	-0.000002
C	-1.818628	-1.177911	0.000001
C	-2.724378	0.000518	0.000003
C	-1.817845	1.178243	0.000001
C	-0.515460	0.745744	-0.000004
C	0.606385	1.613829	-0.000009
H	0.369939	2.675276	0.000017
C	1.949071	1.283086	0.000001
C	2.524730	-0.000126	0.000004
C	1.948466	-1.282948	0.000000
C	0.606113	-1.614175	-0.000003
H	-2.149631	-2.208437	0.000002
H	-3.403864	0.000762	0.869177
H	-3.403873	0.000762	-0.869163
H	-2.147544	2.209189	0.000001
H	2.649347	2.111600	0.000007
H	3.612462	-0.000412	0.000010
H	2.648516	-2.111695	0.000000
H	0.370685	-2.675831	-0.000004

Naph-A

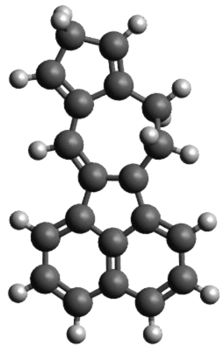

Table S52. Coordinates and energy for the optimized geometry of **Naph-A**.

G = –770.807648, *H* = –770.751992			
		**Coordinates / Å**	
**Atom**	**x**	**y**	**z**
C	3.409318	0.243503	0.021705
C	3.897577	1.579478	0.104274
C	3.001204	2.634253	0.142148
C	1.590335	2.443590	0.103227
C	1.097707	1.155362	0.018597
C	-0.247063	0.534675	-0.036690
C	-0.062974	-0.892076	-0.125133
C	-1.113395	-1.935581	-0.280510
C	-2.337926	-1.764931	0.645345
C	-3.234027	-0.634582	0.247851
C	-4.576821	-0.650853	0.127191
C	-5.094365	0.720050	-0.215229

C	-3.852695	1.542282	-0.271041
C	-2.762733	0.744758	-0.018337
C	-1.431949	1.244104	-0.046583
H	-1.345196	2.327970	-0.087407
C	2.016217	0.094991	-0.018284
C	1.343488	-1.156379	-0.105058
C	2.112917	-2.324930	-0.154023
C	3.512750	-2.202204	-0.114202
C	4.154569	-0.961250	-0.029930
H	4.966424	1.766650	0.135978
H	3.381026	3.648732	0.205165
H	0.937438	3.310545	0.140407
H	-0.665239	-2.919122	-0.115180
H	-1.454985	-1.924450	-1.326182
H	-1.981900	-1.617530	1.673149
H	-2.909530	-2.697286	0.639666
H	-5.216393	-1.513079	0.274068
H	-5.799228	1.111524	0.534603
H	-5.639587	0.755418	-1.170834
H	-3.821950	2.600141	-0.504947
H	1.658229	-3.308216	-0.217134
H	4.118308	-3.101369	-0.149557
H	5.239780	-0.925489	-0.003698

Naph-B

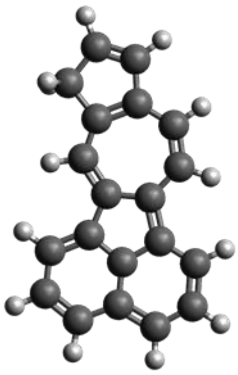

Table S53. Coordinates and energy for the optimized geometry of **Naph-B**.

G = –769.650878, *H* = –769.596670			
		**Coordinates / Å**	
**Atom**	**x**	**y**	**z**
C	3.376776	0.272329	-0.000001
C	3.807547	1.626895	0.000000
C	2.876459	2.658871	0.000001
C	1.479223	2.423086	-0.000001
C	1.032336	1.110178	-0.000002
C	-0.284897	0.471924	-0.000001
C	-0.078906	-0.957881	-0.000001
C	-1.037410	-1.974431	-0.000002

C	-2.422347	-1.868343	0.000000
C	-3.206070	-0.705123	0.000002
C	-4.661343	-0.719648	-0.000002
C	-5.135955	0.544995	0.000002
C	-4.008902	1.528725	0.000002
C	-2.777862	0.654633	0.000001
C	-1.493597	1.171706	-0.000001
C	1.988131	0.071110	-0.000003
C	1.366138	-1.196158	-0.000002
C	2.167570	-2.327895	0.000001
C	3.574500	-2.157564	0.000002
C	4.174835	-0.904143	0.000001
H	4.868895	1.856540	0.000001
H	3.228118	3.685319	0.000002
H	0.795662	3.266351	0.000001
H	-0.646620	-2.988488	-0.000003
H	-2.970173	-2.806698	0.000001
H	-5.246983	-1.631198	-0.000005
H	-6.179521	0.834954	0.000002
H	-4.030659	2.191444	0.876942
H	-4.030660	2.191439	-0.876942
H	-1.417218	2.256795	-0.000001
H	1.751561	-3.330577	0.000003
H	4.202478	-3.042373	0.000004
H	5.257767	-0.823106	0.000002

Naph-C

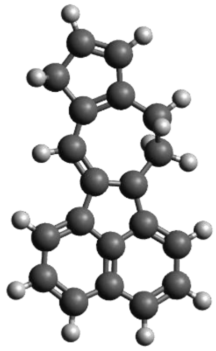

Table S54. Coordinates and energy for the optimized geometry of **Naph-C**.

G = –770.818431, H = –770.762603			
		**Coordinates / Å**	
**Atom**	**x**	**y**	**z**
C	-3.408885	0.266888	0.041774
C	-3.871439	1.611651	0.136039
C	-2.956543	2.650696	0.171542
C	-1.549060	2.436312	0.118347
C	-1.079874	1.140501	0.022638
C	0.250846	0.494790	-0.051194

C	0.048795	-0.909068	-0.130038
C	1.107092	-1.946379	-0.308944
C	2.322743	-1.760694	0.606252
C	3.191474	-0.604917	0.228814
C	4.628069	-0.634967	0.243013
C	5.115404	0.592960	-0.092933
C	3.992004	1.541823	-0.332768
C	2.766202	0.685461	-0.115142
C	1.463974	1.188048	-0.142671
C	-2.020371	0.095084	-0.011843
C	-1.368777	-1.163955	-0.110165
C	-2.154239	-2.315727	-0.158575
C	-3.556597	-2.171384	-0.109527
C	-4.178263	-0.925163	-0.012554
H	-4.936418	1.818675	0.178483
H	-3.318553	3.671260	0.243887
H	-0.881172	3.291902	0.155762
H	1.434202	-1.930452	-1.361081
H	0.680466	-2.938570	-0.137996
H	2.917853	-2.678147	0.606841
H	1.986903	-1.609794	1.644563
H	5.212231	-1.516301	0.479128
H	6.161081	0.861123	-0.184631
H	4.032490	1.979976	-1.340116
H	4.027749	2.390122	0.366230
H	1.380912	2.268421	-0.252269
H	-1.716505	-3.306655	-0.227465
H	-4.174043	-3.062722	-0.145976
H	-5.262525	-0.870593	0.022240

Naph-D

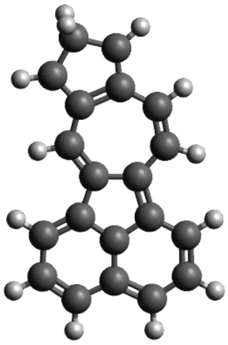

Table S55. Coordinates and energy for the optimized geometry of **Naph-D**.

G = –769.619528, H = –769.565055			
	**Coordinates / Å**		
**Atom**	**x**	**y**	**z**
C	-3.379796	0.244763	0.000020
C	-3.854168	1.586952	0.000025

C	-2.948452	2.635499	0.000006
C	-1.540943	2.432196	-0.000019
C	-1.058559	1.135988	-0.000022
C	0.282239	0.519245	-0.000051
C	0.093041	-0.932023	-0.000026
C	1.047784	-1.973837	-0.000032
C	2.416770	-1.873377	-0.000025
C	3.230839	-0.701356	-0.000006
C	4.599056	-0.726278	0.000035
C	5.124337	0.668169	0.000060
C	3.901660	1.519592	0.000028
C	2.789813	0.721978	-0.000026
C	1.456387	1.223826	-0.000088
H	1.374785	2.308837	0.000098
C	-1.985688	0.079587	-0.000003
C	-1.330897	-1.179348	-0.000010
C	-2.110273	-2.338140	0.000004
C	-3.510977	-2.199782	0.000027
C	-4.139736	-0.951894	0.000036
H	-4.921555	1.784788	0.000043
H	-3.320818	3.654776	0.000009
H	-0.880002	3.293404	-0.000034
H	0.641556	-2.980435	-0.000038
H	2.966516	-2.811476	-0.000024
H	5.218252	-1.614531	0.000054
H	5.768738	0.868320	-0.871604
H	5.768678	0.868309	0.871771
H	3.906362	2.602256	0.000032
H	-1.668419	-3.329142	0.000001
H	-4.124059	-3.094636	0.000041
H	-5.224661	-0.901025	0.000054

Phen-A

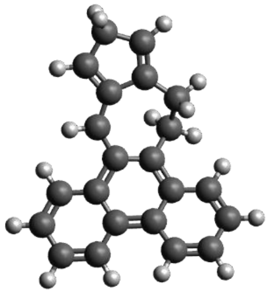

Table S56. Coordinates and energy for the optimized geometry of **Phen-A**.

G = –848.207783, *H* = –848.147453			
		**Coordinates / Å**	
**Atom**	**x**	**y**	**z**
C	-0.274897	0.634017	0.292033

C	0.927097	1.475640	0.164240
C	0.873698	2.878896	0.274007
C	2.016666	3.652253	0.154969
C	3.256810	3.042087	-0.077812
C	3.335734	1.665044	-0.172991
C	2.184763	0.855833	-0.052529
C	2.259595	-0.594549	-0.127314
C	1.118768	-1.387839	0.240931
C	1.238146	-2.809760	0.231887
C	2.405852	-3.426339	-0.153034
C	3.511846	-2.642378	-0.531274
C	3.436583	-1.259949	-0.515601
C	-0.125222	-0.762708	0.530516
C	-1.271379	-1.562081	1.088830
C	-2.356086	-1.905781	0.038272
C	-3.276340	-0.744517	-0.150227
C	-4.607340	-0.752814	-0.360787
C	-5.132308	0.651523	-0.487688
C	-3.909397	1.480038	-0.311984
C	-2.818114	0.663198	-0.103298
C	-1.523884	1.223259	0.070332
H	-1.512469	2.292225	-0.108408
H	-0.061266	3.378484	0.497740
H	1.948611	4.730270	0.259912
H	4.155490	3.643899	-0.165314
H	4.306532	1.208391	-0.318392
H	0.386718	-3.424474	0.490823
H	2.470387	-4.508725	-0.174130
H	4.434037	-3.123213	-0.841891
H	4.303570	-0.691168	-0.825108
H	-1.734584	-0.987793	1.897293
H	-0.898493	-2.476782	1.544904
H	-2.922001	-2.779613	0.373519
H	-1.872639	-2.183149	-0.906744
H	-5.232457	-1.634589	-0.434500
H	-5.898962	0.904404	0.260557
H	-5.604797	0.849825	-1.462493
H	-3.891222	2.563563	-0.336640

Phen-B

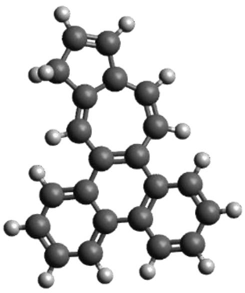

Table S57. Coordinates and energy for the optimized geometry of **Phen-B**.

G = –847.044566, H = –846.985992			
		**Coordinates / Å**	
**Atom**	**x**	**y**	**z**
C	0.363400	0.470653	0.015871
C	-0.747415	1.419734	-0.031373
C	-0.512690	2.810302	-0.154821
C	-1.555716	3.711605	-0.253930
C	-2.878705	3.248254	-0.247007
C	-3.135086	1.891749	-0.150024
C	-2.090077	0.953350	-0.038852
C	-2.354269	-0.475621	0.075205
C	-1.278551	-1.397437	-0.037869
C	-1.553795	-2.779413	0.096385
C	-2.840277	-3.241585	0.300766
C	-3.900979	-2.329778	0.393327
C	-3.655092	-0.971709	0.291482
C	0.092454	-0.916851	-0.204416
C	1.059398	-1.861426	-0.637690
C	2.439762	-1.834946	-0.705716
C	3.298318	-0.831242	-0.244897
C	4.745501	-0.915196	-0.206585
C	5.250804	0.221677	0.326260
C	4.153031	1.174436	0.681455
C	2.903171	0.432091	0.267698
C	1.645526	0.988958	0.330944
H	0.497606	3.192227	-0.226537
H	-1.345470	4.770639	-0.360393
H	-3.703418	3.947877	-0.337845
H	-4.163129	1.554745	-0.183169
H	-0.747454	-3.501803	0.092868
H	-3.019983	-4.305738	0.412147
H	-4.912407	-2.683430	0.565962
H	-4.483864	-0.284851	0.404557
H	0.636720	-2.785550	-1.011450
H	2.909703	-2.723322	-1.117927
H	5.306479	-1.775113	-0.551787
H	6.300279	0.437676	0.485511
H	4.251069	2.136545	0.159838
H	4.141371	1.412689	1.754907
H	1.640226	2.001770	0.716034

Phen-C

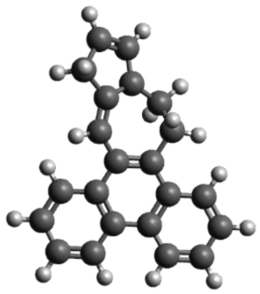

Table S58. Coordinates and energy for the optimized geometry of **Phen-C**.

G = –848.222453, *H* = –848.162064			
		**Coordinates / Å**	
**Atom**	**x**	**y**	**z**
C	-0.334529	0.433290	-0.134659
C	0.733856	1.425423	0.000499
C	0.448976	2.799166	0.171744
C	1.459336	3.737155	0.281155
C	2.800028	3.327130	0.244372
C	3.103612	1.984800	0.120520
C	2.089490	1.006721	0.005454
C	2.394711	-0.412182	-0.069998
C	1.331374	-1.375712	-0.062546
C	1.680789	-2.756825	-0.062886
C	2.992517	-3.175063	-0.104281
C	4.025279	-2.223902	-0.146862
C	3.725398	-0.874877	-0.125731
C	-0.039477	-0.941937	-0.091389
C	-1.150324	-1.961872	-0.204491
C	-2.274891	-1.790436	0.826683
C	-3.260749	-0.782783	0.366950
C	-4.683098	-0.850099	0.490095
C	-5.237565	0.246404	-0.108581
C	-4.176660	1.133592	-0.662745
C	-2.904000	0.404359	-0.297265
C	-1.636642	0.938207	-0.448252
H	-0.576438	3.138186	0.257106
H	1.210224	4.785384	0.412145
H	3.598716	4.056417	0.334745
H	4.144905	1.689781	0.131615
H	0.913619	-3.516860	-0.027494
H	3.223212	-4.235197	-0.104826
H	5.061349	-2.544677	-0.189538
H	4.542820	-0.166831	-0.156023
H	-0.763976	-2.970979	-0.108453
H	-1.577320	-1.904402	-1.216404
H	-1.861695	-1.469792	1.796710
H	-2.772669	-2.747768	1.005379

H	-5.217397	-1.661158	0.969098
H	-6.297670	0.456584	-0.188247
H	-4.228063	2.142534	-0.230113
H	-4.288110	1.264060	-1.748871
H	-1.636092	1.941374	-0.863540

Phen-D

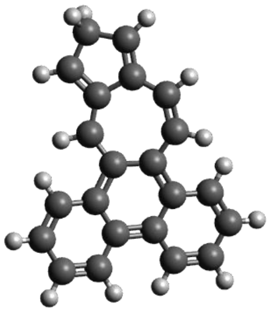

Table S59. Coordinates and energy for the optimized geometry of **Phen-D**.

G = –847.011020, H = –846.952084			
	**Coordinates / Å**		
**Atom**	**x**	**y**	**z**
C	-0.343853	0.521801	-0.059774
C	0.813806	1.431724	-0.082800
C	0.640004	2.822251	-0.230261
C	1.726677	3.681102	-0.283223
C	3.027786	3.168577	-0.211205
C	3.221655	1.802786	-0.093647
C	2.130001	0.913699	-0.024922
C	2.314410	-0.525020	0.103187
C	1.200481	-1.405538	-0.077623
C	1.414927	-2.807003	0.051733
C	2.662430	-3.322997	0.326964
C	3.752549	-2.451936	0.487961
C	3.574637	-1.082653	0.384736
C	-0.129346	-0.885504	-0.301385
C	-1.120514	-1.816860	-0.775127
C	-2.484089	-1.771436	-0.789008
C	-3.339725	-0.766439	-0.250537
C	-4.698040	-0.833274	-0.146528
C	-5.205296	0.416573	0.494667
C	-3.980976	1.228272	0.737149
C	-2.882945	0.529767	0.297307
C	-1.571425	1.064418	0.302507
H	-1.532780	2.076989	0.689519
H	-0.351825	3.239875	-0.355848
H	1.563549	4.746736	-0.407637
H	3.883118	3.833859	-0.268570
H	4.234373	1.420335	-0.077228

H	0.578895	-3.490350	-0.010476
H	2.794770	-4.393663	0.438436
H	4.737214	-2.849359	0.712629
H	4.427050	-0.435932	0.546483
H	-0.705100	-2.718525	-1.204551
H	-2.986711	-2.631580	-1.224285
H	-5.323668	-1.658435	-0.461872
H	-5.755139	0.215374	1.428775
H	-5.928763	0.952192	-0.141461
H	-3.973914	2.218617	1.175059

1-TP

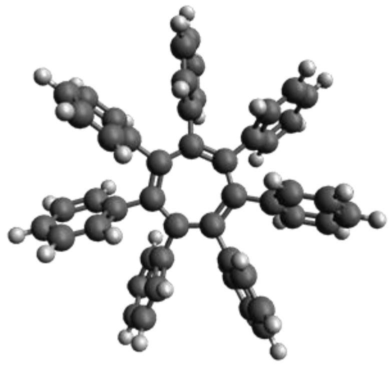

Table S60. Coordinates and energy for the optimized geometry of **1-TP**.

G = –1887.726030			
		**Coordinates / Å**	
**Atom**	**x**	**y**	**z**
C	1.029835	1.242096	-0.170062
C	-0.316803	1.590390	0.103282
C	-1.435577	0.728250	0.116199
C	-1.463087	-0.665828	-0.143011
C	-0.385951	-1.577147	-0.074199
C	0.975318	-1.280583	0.182127
C	1.620567	-0.034831	-0.015544
C	1.963810	2.359055	-0.525191
C	2.379917	3.307188	0.416560
H	2.023654	3.245232	1.438732
C	3.247560	4.328813	0.040811
H	3.570496	5.056348	0.779078
C	3.696138	4.420389	-1.279073
H	4.366994	5.222509	-1.571230
C	3.281603	3.477441	-2.220189
H	3.626482	3.541320	-3.247643
C	2.424961	2.443452	-1.843453
H	2.108389	1.704416	-2.572970

C	-0.610507	3.049335	0.292753
C	-0.613743	3.930859	-0.793949
H	-0.391023	3.561349	-1.789281
C	-0.899083	5.279494	-0.596459
H	-0.905898	5.956295	-1.445315
C	-1.170538	5.758933	0.686958
H	-1.387320	6.811743	0.839823
C	-1.162958	4.881743	1.772463
H	-1.372222	5.247797	2.772910
C	-0.890644	3.528471	1.576574
H	-0.894088	2.843981	2.418857
C	-2.768503	1.358673	0.395913
C	-3.425428	2.145872	-0.555340
H	-2.975447	2.305034	-1.529354
C	-4.655940	2.723922	-0.252583
H	-5.161026	3.330534	-0.998016
C	-5.235513	2.527245	1.002981
H	-6.192101	2.983723	1.238293
C	-4.582575	1.740904	1.952861
H	-5.026643	1.582013	2.930815
C	-3.355741	1.152043	1.648706
H	-2.847434	0.539512	2.387420
C	-2.812081	-1.236798	-0.467529
C	-3.341512	-1.008909	-1.742237
H	-2.778646	-0.422422	-2.462126
C	-4.581186	-1.543834	-2.091437
H	-4.980415	-1.369291	-3.085904
C	-5.304167	-2.296066	-1.165175
H	-6.270609	-2.710432	-1.435548
C	-4.782405	-2.512682	0.112393
H	-5.342545	-3.092374	0.839666
C	-3.539781	-1.989087	0.460321
H	-3.133370	-2.164209	1.450373
C	-0.742311	-3.028641	-0.207454
C	-1.030510	-3.553529	-1.471522
H	-0.995429	-2.909235	-2.344175
C	-1.361945	-4.900715	-1.608165
H	-1.577815	-5.303067	-2.593142
C	-1.419812	-5.725594	-0.483656
H	-1.682850	-6.773496	-0.590799
C	-1.138816	-5.200320	0.779490
H	-1.183872	-5.836380	1.658143
C	-0.794705	-3.857876	0.918305
H	-0.564146	-3.452628	1.897947
C	1.862076	-2.421810	0.578028
C	2.234373	-3.430437	-0.318043
H	1.881792	-3.400749	-1.342674
C	3.054549	-4.472021	0.106183

H	3.343524	-5.246732	-0.597358
C	3.499454	-4.523226	1.429576
H	4.132617	-5.341174	1.759516
C	3.130214	-3.519178	2.325026
H	3.472983	-3.550472	3.354696
C	2.321779	-2.465546	1.899101
H	2.040292	-1.679600	2.593307
C	3.120459	-0.079227	-0.068626
C	3.759950	-0.617090	-1.190313
H	3.167946	-1.000464	-2.015559
C	5.151834	-0.652718	-1.252088
H	5.639914	-1.065493	-2.129702
C	5.912990	-0.163344	-0.189146
H	6.997238	-0.195293	-0.235791
C	5.276074	0.367372	0.934315
H	5.861646	0.748001	1.765576
C	3.884740	0.415267	0.993017
H	3.390672	0.832705	1.864445

1-DT

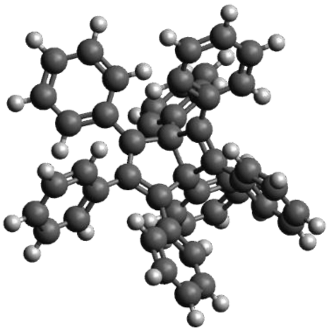

Table S61. Coordinates and energy for the optimized geometry of **1-DT**.

G = –1887.716081			
	Coordinates / Å		
**Atom**	**x**	**y**	**z**
C	-4.817226	2.320639	0.253372
H	-5.267833	3.247994	0.591305
C	-5.615334	1.311514	-0.296537
H	-6.685944	1.461319	-0.396155
C	-5.038276	0.104679	-0.703241
H	-5.656080	-0.684777	-1.118086
C	-3.673114	-0.094563	-0.558848
H	-3.243213	-1.042813	-0.850987
C	-2.840759	0.929797	-0.044928
C	-1.420397	0.702943	0.104318
C	-0.412318	1.682452	0.190298
C	-0.601876	3.125567	-0.087078
C	-1.092459	3.546758	-1.331501

H	-1.340477	2.808453	-2.088330
C	-1.253575	4.905006	-1.596687
H	-1.628103	5.224057	-2.564485
C	-0.934171	5.852308	-0.620542
H	-1.062960	6.910246	-0.827621
C	-0.448478	5.437347	0.621121
H	-0.200442	6.170310	1.382583
C	-0.278091	4.079348	0.886812
C	-3.446720	2.141839	0.368865
C	0.831594	1.091582	0.499461
C	0.682027	-0.383864	0.749641
C	-0.817908	-0.670119	0.215964
C	1.170812	-1.096146	-0.529978
C	-0.072750	-1.245744	-1.033746
C	2.133172	1.718410	0.421409
C	3.209679	1.229131	1.198650
H	3.037865	0.434138	1.910654
C	4.476289	1.785187	1.086191
H	5.285439	1.407820	1.702509
C	4.709035	2.815414	0.171952
H	5.703210	3.240738	0.075457
C	3.664164	3.295009	-0.626106
H	3.849219	4.080261	-1.351685
C	2.388414	2.767930	-0.496549
H	1.595256	3.132757	-1.134135
C	0.975194	-0.926536	2.121677
C	0.850682	-0.109380	3.252150
H	0.632985	0.948375	3.134530
C	1.006665	-0.644735	4.530088
H	0.912514	0.000789	5.398076
C	1.279971	-2.004042	4.691338
H	1.400092	-2.421639	5.686322
C	1.400417	-2.824131	3.567657
H	1.610292	-3.882982	3.684166
C	1.248018	-2.288553	2.290165
H	1.323918	-2.931635	1.419522
C	-1.612494	-1.671813	1.024557
C	-2.190697	-1.261679	2.235124
H	-2.113533	-0.224511	2.544796
C	-2.850517	-2.173251	3.053139
H	-3.285075	-1.837976	3.990111
C	-2.949886	-3.513716	2.671231
H	-3.465999	-4.225963	3.307989
C	-2.381798	-3.929559	1.468373
H	-2.452674	-4.968530	1.160465
C	-1.711773	-3.015535	0.652314
H	-1.270162	-3.359258	-0.274984
C	2.518430	-1.298478	-1.058044

C	2.762119	-1.127633	-2.433246
H	1.943101	-0.851083	-3.088758
C	4.043898	-1.292049	-2.949129
H	4.218354	-1.150079	-4.011510
C	5.104405	-1.628442	-2.102702
H	6.104580	-1.753979	-2.506503
C	4.873801	-1.798068	-0.736718
H	5.692993	-2.058065	-0.073058
C	3.592398	-1.631309	-0.215522
H	3.421348	-1.770420	0.845547
C	-0.601675	-1.818396	-2.268956
C	-1.599293	-1.155749	-3.004524
H	-1.984130	-0.203475	-2.657697
C	-2.069770	-1.696949	-4.199713
H	-2.834992	-1.171556	-4.762868
C	-1.557559	-2.906892	-4.671512
H	-1.930404	-3.330449	-5.599082
C	-0.562217	-3.571956	-3.948729
H	-0.160480	-4.512503	-4.313057
C	-0.085528	-3.033107	-2.758014
H	0.684463	-3.547533	-2.191740
H	0.104413	3.753741	1.849402
H	-2.844712	2.925429	0.807394

2-TP (twisted)

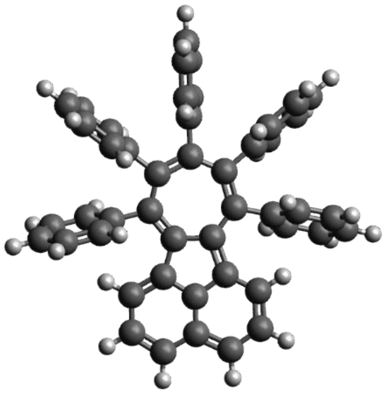

Table S62. Coordinates and energy for the optimized geometry of **2-TP (twisted)**.

G = –1809.171254			
	Coordinates / Å		
**Atom**	**x**	**y**	**z**
C	1.232920	-0.913144	0.017124
C	-0.072161	-1.461540	-0.001382
C	1.624930	0.446747	-0.049356
C	0.802743	1.585333	-0.087975
C	-0.642120	1.657030	0.084527
C	-1.572561	0.605036	0.048045
C	-1.316695	-0.786960	-0.018648

C	-0.998340	3.082303	0.215879
C	-2.156338	3.800838	0.516330
C	-2.102628	5.212469	0.553858
H	-3.097418	3.320250	0.734542
C	-0.940505	5.920091	0.287639
H	-3.012484	5.753761	0.791508
H	-0.942759	7.005835	0.308691
C	1.298385	2.967987	-0.220031
C	2.522293	3.567658	-0.519930
C	0.189348	3.816785	-0.002590
C	2.608942	4.977561	-0.558993
H	3.411304	2.995720	-0.736396
C	0.259346	5.223064	-0.003190
C	1.522533	5.797258	-0.294299
H	3.568275	5.425583	-0.796290
H	1.632558	6.877386	-0.316271
C	-3.017612	1.000372	0.048213
C	-3.791641	0.885006	1.207013
C	-3.590717	1.508327	-1.122931
C	-5.126946	1.287077	1.196094
H	-3.347394	0.491423	2.115386
C	-4.928959	1.897231	-1.134841
H	-2.986155	1.602127	-2.019721
C	-5.698090	1.792697	0.026045
H	-5.720549	1.203468	2.101246
H	-5.368128	2.287123	-2.047974
H	-6.738788	2.101952	0.019010
C	-2.536438	-1.664106	-0.073524
C	-3.253053	-1.792008	-1.267667
C	-2.964617	-2.355666	1.064467
C	-4.380178	-2.609869	-1.325461
H	-2.924790	-1.254720	-2.151665
C	-4.097236	-3.165406	1.007806
H	-2.410215	-2.260345	1.992460
C	-4.806305	-3.297251	-0.187698
H	-4.925408	-2.707506	-2.259423
H	-4.423343	-3.695258	1.897742
H	-5.686194	-3.931962	-0.232112
C	-0.146911	-2.963319	-0.003556
C	0.102634	-3.684005	1.168867
C	-0.467818	-3.649530	-1.179302
C	0.026969	-5.075481	1.165883
H	0.354112	-3.154977	2.082289
C	-0.533055	-5.041516	-1.183261
H	-0.664802	-3.092971	-2.089788
C	-0.288441	-5.758133	-0.010386
H	0.216861	-5.625550	2.082635
H	-0.777686	-5.565056	-2.102578

H	-0.343428	-6.842570	-0.012935
C	2.360193	-1.906153	0.076465
C	2.721421	-2.637966	-1.059621
C	3.057916	-2.101717	1.272686
C	3.768952	-3.554853	-0.998968
H	2.181767	-2.489469	-1.989291
C	4.099116	-3.026241	1.334441
H	2.782346	-1.532883	2.154982
C	4.458479	-3.753911	0.198676
H	4.044030	-4.115256	-1.887422
H	4.629999	-3.175385	2.269857
H	5.271653	-4.471915	0.246271
C	3.101826	0.698520	-0.046479
C	3.863918	0.507131	-1.203221
C	3.718657	1.149480	1.125766
C	5.232022	0.776913	-1.188913
H	3.385730	0.157832	-2.112470
C	5.088479	1.405801	1.141070
H	3.123640	1.302700	2.020799
C	5.846722	1.225425	-0.017629
H	5.817081	0.635033	-2.092371
H	5.561294	1.751763	2.055099
H	6.912616	1.431669	-0.008069

2-TP (planar)

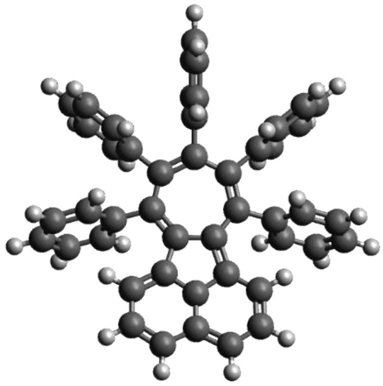

Table S63. Coordinates and energy for the optimized geometry of **2-TP (planar)**.

G = –1809.173404			
		**Coordinates / Å**	
**Atom**	**x**	**y**	**z**
C	1.440773	-0.029140	-0.237916
C	0.869269	1.254556	-0.076094
C	-0.498179	1.603875	0.027786
C	-1.608207	0.758471	-0.150386
C	-1.636509	-0.695757	-0.155455
C	-0.561417	-1.584843	0.024624

C	0.818876	-1.289124	-0.074266
C	2.923105	-0.058338	-0.480933
C	3.397497	-0.067247	-1.796184
H	2.693305	-0.053778	-2.623032
C	4.769467	-0.092901	-2.045745
H	5.129417	-0.099451	-3.070197
C	5.673400	-0.109873	-0.982928
H	6.741629	-0.129614	-1.176516
C	5.200153	-0.101329	0.331179
H	5.898455	-0.114595	1.162601
C	3.830026	-0.075689	0.582773
H	3.461280	-0.069395	1.603302
C	1.847469	2.388461	0.059544
C	2.458086	2.959881	-1.060979
H	2.228346	2.581559	-2.051362
C	3.356955	4.013882	-0.907460
H	3.822724	4.453728	-1.784149
C	3.658204	4.499523	0.366306
H	4.360856	5.318821	0.484715
C	3.053267	3.928133	1.487076
H	3.283229	4.299031	2.481350
C	2.148371	2.878821	1.334951
H	1.672350	2.439622	2.206189
C	-0.797214	3.038133	0.330220
C	-0.625491	4.044182	-0.627199
H	-0.258393	3.791251	-1.615615
C	-0.941967	5.365000	-0.314685
H	-0.815881	6.139411	-1.064974
C	-1.418255	5.690984	0.957695
H	-1.659849	6.721400	1.199791
C	-1.581686	4.690001	1.917047
H	-1.949839	4.937354	2.907960
C	-1.279693	3.365806	1.602514
H	-1.418596	2.582624	2.341617
C	-2.995204	1.228944	-0.324164
C	-3.620018	2.470119	-0.446100
H	-3.078501	3.401453	-0.384386
C	-5.014791	2.522083	-0.668505
H	-5.483616	3.496616	-0.756950
C	-5.793836	1.380684	-0.776938
H	-6.863449	1.461539	-0.945315
C	-5.190517	0.101651	-0.675154
C	-5.841346	-1.152475	-0.794283
H	-6.913018	-1.191186	-0.966154
C	-5.105494	-2.323547	-0.699631
H	-5.610094	-3.278675	-0.801745
C	-3.710269	-2.326489	-0.473751
H	-3.203421	-3.277900	-0.422698

C	-3.039778	-1.111112	-0.335896
C	-3.801891	0.074353	-0.444520
C	-0.917962	-3.006033	0.326533
C	-0.776329	-4.020575	-0.626981
H	-0.390543	-3.784752	-1.612515
C	-1.145504	-5.327552	-0.314384
H	-1.042251	-6.108018	-1.061880
C	-1.644636	-5.632086	0.954542
H	-1.926983	-6.652024	1.196880
C	-1.778442	-4.623130	1.910091
H	-2.164069	-4.853852	2.898361
C	-1.424012	-3.312090	1.595209
H	-1.539721	-2.522550	2.331586
C	1.751892	-2.459533	0.067360
C	2.341444	-3.059413	-1.049530
H	2.125896	-2.678669	-2.042195
C	3.200383	-4.145196	-0.889288
H	3.650231	-4.606954	-1.763018
C	3.482207	-4.634717	0.387500
H	4.154070	-5.478695	0.511121
C	2.897508	-4.035552	1.504489
H	3.112483	-4.409520	2.500957
C	2.032272	-2.954181	1.345668
H	1.572077	-2.492529	2.213771

2-DT

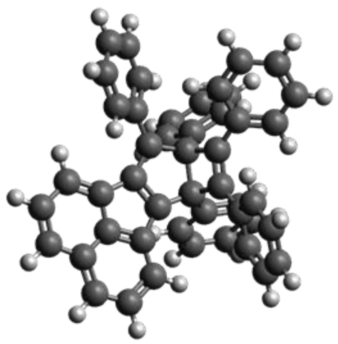

Table S64. Coordinates and energy for the optimized geometry of **2-DT**.

G = –1809.142545			
	**Coordinates / Å**		
**Atom**	**x**	**y**	**z**
C	4.530080	2.158673	-0.327814
C	5.554449	1.178022	-0.365969
C	3.227288	1.676782	-0.135219
H	6.584431	1.486215	-0.519780
C	5.264341	-0.179401	-0.202662
C	2.918492	0.291003	0.049689
H	6.077272	-0.896529	-0.236080

C	3.955947	-0.644726	0.015485
C	1.511160	0.235869	0.260838
C	2.072945	2.496423	-0.069629
H	3.767679	-1.703096	0.152063
C	0.960771	1.551310	0.158164
C	0.472220	-0.788142	0.499763
C	2.246571	3.865354	-0.167693
C	4.674224	3.570459	-0.449016
C	-0.422815	1.520001	0.256684
C	-0.887525	0.088065	0.502485
C	-0.065763	-1.380016	-0.826265
C	0.682437	-1.734875	1.656810
H	1.412802	4.554512	-0.095488
C	3.557918	4.383022	-0.360533
H	5.658058	4.003615	-0.600546
C	-1.315785	2.639206	0.017758
C	-1.229660	-0.691919	-0.805738
C	-1.774144	-0.107939	1.708803
C	0.630261	-2.275080	-1.743197
C	1.570242	-1.421161	2.692175
C	-0.079418	-2.906904	1.746112
H	3.677210	5.458424	-0.440690
C	-0.971440	3.582354	-0.976749
C	-2.551181	2.780165	0.688113
C	-2.442589	-0.725017	-1.620658
C	-1.365345	0.435284	2.936230
C	-2.943939	-0.869761	1.657147
C	1.647458	-3.127232	-1.277209
C	0.341595	-2.257221	-3.121142
H	2.159533	-0.511039	2.646669
C	1.699853	-2.267188	3.794135
C	0.046980	-3.749244	2.848179
H	-0.773116	-3.157142	0.950472
H	-0.056426	3.450305	-1.541402
C	-1.831259	4.627242	-1.289562
C	-3.392733	3.844645	0.387472
H	-2.834704	2.071433	1.453304
C	-3.240424	0.413962	-1.824459
C	-2.853774	-1.947107	-2.189976
H	-0.453015	1.022398	2.987024
C	-2.117423	0.225321	4.088272
C	-3.699540	-1.077698	2.813243
H	-3.270821	-1.301686	0.719195
H	1.872018	-3.164920	-0.217160
C	2.346520	-3.946657	-2.161552
C	1.043538	-3.075597	-4.000150
H	-0.424651	-1.588479	-3.497879
H	2.393760	-2.010029	4.588856

C	0.938198	-3.432885	3.876152
H	-0.550051	-4.654616	2.902521
H	-1.564428	5.327547	-2.074325
C	-3.040346	4.767101	-0.601722
H	-4.330684	3.951951	0.922507
H	-2.948162	1.362179	-1.395448
C	-4.401582	0.336760	-2.591004
C	-4.014372	-2.019780	-2.952957
H	-2.261334	-2.839388	-2.016492
H	-1.787678	0.650475	5.031466
C	-3.290436	-0.532488	4.029501
H	-4.608297	-1.669647	2.758470
H	3.124213	-4.603482	-1.783454
C	2.047590	-3.924770	-3.524631
H	0.812351	-3.046415	-5.060801
H	1.037814	-4.091192	4.733908
H	-3.708771	5.588254	-0.840947
H	-5.001597	1.229108	-2.742460
C	-4.792919	-0.876699	-3.158454
H	-4.317603	-2.970613	-3.380851
H	-3.878977	-0.696283	4.927176
H	2.593567	-4.562233	-4.213484
H	-5.700397	-0.934712	-3.751846

2-MT

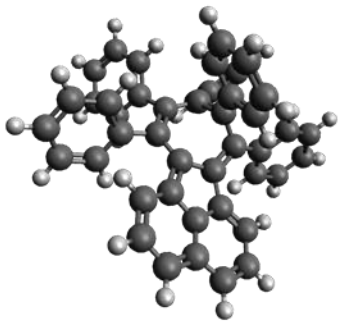

Table S65. Coordinates and energy for the optimized geometry of **2-MT**.

G = –1809.136004			
		**Coordinates / Å**	
**Atom**	**x**	**y**	**z**
C	-4.434356	-3.398753	-0.878815
H	-5.190196	-4.151601	-1.083044
C	-4.798159	-2.174348	-0.339886
C	-2.512010	-1.388536	-0.323271
C	-2.147821	-2.663917	-0.833070
H	-5.844359	-1.980897	-0.124187
C	-3.854482	-1.157980	-0.051494
C	-1.263790	-0.612905	-0.167369
C	-0.744641	-2.815689	-0.939832

C	-3.066648	-3.686847	-1.132182
H	-4.198467	-0.230818	0.386314
C	-0.162334	-1.580847	-0.375615
C	-1.185305	0.792400	-0.080415
C	-0.241727	-4.026854	-1.380325
C	-2.523680	-4.910228	-1.611899
C	1.104153	-1.370623	0.127761
C	1.035555	-0.264905	1.099770
C	0.033358	1.561733	-0.053489
H	0.823096	-4.206702	-1.468607
C	-1.152329	-5.059882	-1.726942
H	-3.188466	-5.727572	-1.876031
C	1.226482	0.975599	0.383696
H	-0.749355	-6.002155	-2.085337
C	-2.447326	1.590025	-0.124440
C	-2.799224	2.358664	0.991558
C	-3.270786	1.612360	-1.258216
C	-3.967265	3.122321	0.982757
H	-2.159186	2.349016	1.869366
C	-4.427212	2.387545	-1.273862
H	-3.001431	1.018074	-2.125819
C	-4.781614	3.140317	-0.150612
H	-4.236161	3.706771	1.857621
H	-5.053017	2.404183	-2.161124
H	-5.686051	3.741213	-0.161675
C	0.068371	2.930210	-0.627651
C	0.665966	3.968455	0.103038
C	-0.452532	3.208071	-1.900991
C	0.726497	5.258669	-0.418470
H	1.077875	3.759148	1.085534
C	-0.383532	4.495486	-2.426335
H	-0.894681	2.408036	-2.484135
C	0.202307	5.525230	-1.685052
H	1.183042	6.054528	0.162158
H	-0.783828	4.695486	-3.415635
H	0.252363	6.529702	-2.094515
C	2.589148	1.317368	-0.043631
C	3.648889	1.102363	0.856959
C	2.878560	1.785760	-1.339651
C	4.964564	1.358313	0.477900
H	3.433991	0.755197	1.862294
C	4.191927	2.038218	-1.713340
H	2.073661	1.933102	-2.049466
C	5.238375	1.826056	-0.807071
H	5.771700	1.192572	1.184388
H	4.406241	2.391914	-2.717062
H	6.262975	2.024145	-1.106667
C	0.503983	-0.376635	2.407500

C	0.150284	-1.644785	2.943688
C	0.357196	0.785408	3.212692
C	-0.377910	-1.732568	4.218403
H	0.283312	-2.533665	2.337338
C	-0.178917	0.684738	4.484868
H	0.675289	1.744533	2.820618
C	-0.551265	-0.570283	4.986255
H	-0.656282	-2.698001	4.626890
H	-0.297187	1.571555	5.097995
H	-0.961706	-0.646787	5.988196
C	2.360078	-2.004599	-0.253898
C	3.360589	-2.216468	0.712283
C	2.614106	-2.353873	-1.592719
C	4.576084	-2.790243	0.351418
H	3.173547	-1.945333	1.746555
C	3.831126	-2.924263	-1.949245
H	1.861412	-2.148949	-2.346853
C	4.813455	-3.145712	-0.978169
H	5.338600	-2.957388	1.105639
H	4.021681	-3.183858	-2.985897
H	5.764462	-3.586854	-1.260375

3-TP

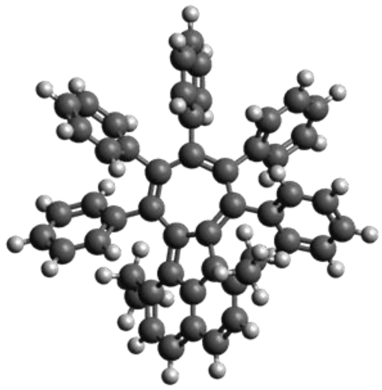

Table S66. Coordinates and energy for the optimized geometry of **3-TP**.

G = –1966.331489			
	**Coordinates / Å**		
**Atom**	**x**	**y**	**z**
C	1.154448	-1.254235	-0.220544
C	1.773388	-0.000575	-0.000055
C	-0.195471	-1.594746	0.066934
C	-1.254172	-0.683349	0.254966
C	-1.253736	0.684227	-0.255087
C	-0.194422	1.594920	-0.067080
C	1.155267	1.253476	0.220409
C	-2.643192	1.005617	-0.610939
C	-3.274355	1.966547	-1.415667
C	-4.696316	1.962196	-1.422038

C	-5.464903	1.043636	-0.728127
H	-5.204662	2.714788	-2.014188
H	-6.548720	1.100813	-0.768887
C	-2.643807	-1.003841	0.610890
C	-3.275530	-1.964412	1.415603
C	-3.436241	0.001155	0.000016
C	-4.697485	-1.959117	1.422111
C	-4.839273	0.001610	0.000070
C	-5.465526	-1.040016	0.728308
H	-5.206277	-2.711408	2.014259
H	-6.549377	-1.096485	0.769152
C	-0.544791	3.049149	-0.018961
C	0.109883	3.988874	-0.827762
C	-1.523153	3.489832	0.881754
C	-0.211870	5.339599	-0.741752
H	0.857471	3.657164	-1.539018
C	-1.837992	4.844729	0.975537
H	-2.028581	2.771817	1.519189
C	-1.184769	5.772799	0.163155
H	0.295347	6.054186	-1.382534
H	-2.591330	5.173199	1.684955
H	-1.431325	6.827833	0.233975
C	2.022813	2.358717	0.750248
C	1.799576	2.782909	2.065583
C	3.033856	2.966674	-0.002939
C	2.583904	3.791840	2.624041
H	1.011127	2.319406	2.651027
C	3.808891	3.981586	0.551823
H	3.214158	2.645428	-1.022586
C	3.589578	4.395008	1.868026
H	2.405439	4.106567	3.647887
H	4.586477	4.449006	-0.044754
H	4.198417	5.183351	2.300364
C	3.277168	-0.000990	-0.000051
C	3.979865	-0.031222	-1.208581
C	3.979862	0.028941	1.208489
C	5.373673	-0.029187	-1.207792
H	3.434187	-0.064070	-2.146276
C	5.373669	0.026205	1.207721
H	3.434185	0.062080	2.146174
C	6.073758	-0.001674	-0.000030
H	5.911689	-0.050701	-2.150744
H	5.911683	0.047463	2.150681
H	7.159616	-0.001946	-0.000023
C	2.021188	-2.360133	-0.750338
C	3.031246	-2.969376	0.003136
C	1.798113	-2.783713	-2.065899
C	3.805515	-3.984892	-0.551590

H	3.211365	-2.648682	1.022986
C	2.581695	-3.793242	-2.624321
H	1.010390	-2.319248	-2.651556
C	3.586415	-4.397657	-1.868034
H	4.582335	-4.453303	0.045208
H	2.403386	-4.107466	-3.648349
H	4.194662	-5.186474	-2.300342
C	-0.546796	-3.048747	0.018989
C	0.107562	-3.988858	0.827596
C	-1.525756	-3.488835	-0.881368
C	-0.215083	-5.339380	0.741747
H	0.855630	-3.657596	1.538558
C	-1.841497	-4.843533	-0.974986
H	-2.030950	-2.770517	-1.518647
C	-1.188580	-5.771991	-0.162799
H	0.291908	-6.054268	1.382372
H	-2.595295	-5.171548	-1.684125
H	-1.435835	-6.826869	-0.233494
C	-2.540012	2.918354	-2.328676
H	-2.527598	3.918179	-1.877375
H	-1.499230	2.604150	-2.406463
C	-2.541711	-2.916804	2.328424
H	-2.530045	-3.916596	1.877029
H	-1.500701	-2.603322	2.406084
C	-3.131850	-3.001514	3.744839
H	-4.135022	-3.437347	3.754565
H	-2.492536	-3.634803	4.368065
H	-3.187699	-2.010770	4.208214
C	-3.130302	3.003339	-3.745012
H	-4.133179	3.439852	-3.754628
H	-2.490649	3.636139	-4.368388
H	-3.186892	2.012593	-4.208292

3-DT

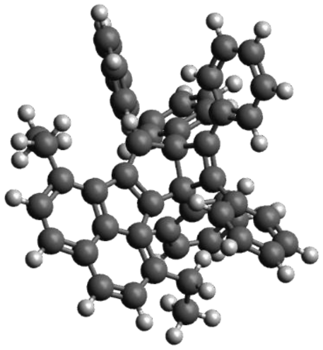

Table S67. Coordinates and energy for the optimized geometry of **3-DT**.

G = –1966.325641

	Coordinates / Å		
Atom	x	y	z
C	2.842616	3.721964	-0.390981
C	4.178941	3.300935	-0.611277
C	1.916409	2.706357	-0.121413
H	4.940117	4.039945	-0.845470
C	4.537041	1.957885	-0.535920
C	2.282600	1.317273	-0.011549
H	5.571643	1.687555	-0.721027
C	3.622940	0.926595	-0.224170
C	1.082273	0.634397	0.305037
C	0.533402	2.925629	0.099528
C	-0.015387	1.572053	0.314602
C	0.620361	-0.759781	0.490762
C	0.063243	4.240051	0.148368
C	2.340006	5.049974	-0.405262
C	-1.217621	0.896010	0.348977
C	-0.983505	-0.598541	0.514969
C	0.390380	-1.450388	-0.885570
C	1.257910	-1.557831	1.598225
C	1.005529	5.276530	-0.127813
H	3.005520	5.882432	-0.612317
C	-2.536514	1.428729	0.016852
C	-0.950520	-1.325978	-0.863591
C	-1.688692	-1.245721	1.678821
C	1.428489	-1.840658	-1.851482
C	1.919144	-0.916720	2.652700
C	1.115814	-2.949944	1.633941
H	0.641820	6.299342	-0.108570
C	-2.678922	2.117298	-1.202692
C	-3.674205	1.181771	0.806615
C	-2.015756	-1.648649	-1.806380
C	-1.630772	-0.626530	2.936353
C	-2.315238	-2.488895	1.561289
C	2.080793	-3.080722	-1.787941
C	1.841033	-0.898192	-2.809077
H	2.023544	0.163775	2.649390
C	2.446801	-1.654718	3.712010
C	1.640989	-3.688085	2.692793
H	0.574822	-3.454111	0.840906
H	-1.804513	2.287587	-1.822472
C	-3.937240	2.526533	-1.634670
C	-4.922635	1.629426	0.385278
H	-3.573813	0.660289	1.750152
C	-3.366192	-1.419014	-1.491138
C	-1.699400	-2.190056	-3.068549
H	-1.128537	0.330810	3.042811
C	-2.204108	-1.233594	4.050694

C	-2.889717	-3.097817	2.678594
H	-2.354576	-2.985917	0.598770
H	1.773259	-3.813611	-1.050671
C	3.128637	-3.368724	-2.661696
C	2.889467	-1.189300	-3.679888
H	1.337786	0.062438	-2.858841
H	2.959238	-1.141846	4.520451
C	2.312499	-3.043488	3.733517
H	1.522901	-4.767404	2.704802
H	-4.039588	3.032023	-2.589574
C	-5.060099	2.288712	-0.839405
H	-5.792690	1.454495	1.010112
H	-3.632968	-1.015658	-0.523372
C	-4.369167	-1.703933	-2.414386
C	-2.704723	-2.475465	-3.986244
H	-0.663319	-2.379054	-3.326544
H	-2.152715	-0.742198	5.017596
C	-2.838132	-2.472561	3.923994
H	-3.375328	-4.063165	2.571931
H	3.626585	-4.331891	-2.603694
C	3.538864	-2.423675	-3.604400
H	3.200352	-0.452094	-4.414084
H	2.723848	-3.619133	4.557135
H	-6.039948	2.616697	-1.172178
H	-5.405612	-1.511972	-2.153695
C	-4.043617	-2.230581	-3.665170
H	-2.444300	-2.889060	-4.956004
H	-3.285405	-2.947009	4.792218
H	4.358338	-2.649473	-4.280070
H	-4.825827	-2.451616	-4.385199
C	-1.338884	4.621870	0.549356
H	-1.458391	5.703150	0.429112
H	-2.064075	4.149201	-0.118051
C	4.106917	-0.493632	-0.129043
H	4.562321	-0.764503	-1.090030
H	3.267525	-1.171509	0.015315
C	5.128949	-0.701704	1.002111
H	5.450958	-1.747379	1.017231
H	6.015430	-0.073915	0.868861
H	4.684303	-0.469464	1.973888
C	-1.663139	4.232302	2.001969
H	-0.974929	4.720574	2.699697
H	-2.684062	4.535671	2.253649
H	-1.56525	3.200833	2.131464

4-TP

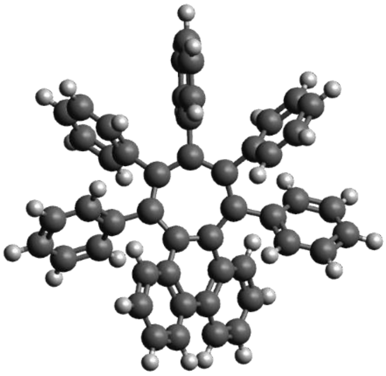

Table S68. Coordinates and energy for the optimized geometry of **4-TP**.

	G = –1886.558425		
		**Coordinates / Å**	
**Atom**	**x**	**y**	**z**
C	-0.985045	-1.257322	-0.158023
C	-1.616158	-0.000582	-0.000003
C	0.357412	-1.556940	0.177736
C	1.432710	-0.639361	0.322460
C	1.432362	0.640378	-0.322262
C	0.356336	1.557142	-0.178049
C	-0.985883	1.256582	0.157958
C	2.677911	1.021122	-0.989244
C	3.906630	0.441995	-0.580638
C	2.657811	1.860542	-2.123642
C	5.077476	0.749577	-1.293671
C	3.820806	2.140196	-2.819570
H	1.718421	2.265305	-2.474459
C	5.039562	1.592486	-2.394553
H	6.019331	0.301320	-0.999858
H	3.782161	2.771469	-3.701221
H	5.953392	1.810183	-2.938226
C	2.678218	-1.019268	0.989926
C	2.658186	-1.858866	2.124197
C	3.906685	-0.439132	0.582007
C	3.821039	-2.137686	2.820689
H	1.718924	-2.264403	2.474476
C	5.077398	-0.745842	1.295631
C	5.039575	-1.588917	2.396390
H	3.782451	-2.769122	3.702225
H	6.019046	-0.296791	1.002370
H	5.953288	-1.805942	2.940527
C	0.721651	3.009077	-0.252405
C	0.101643	3.874157	-1.163660
C	1.698193	3.512843	0.616863
C	0.461275	5.218170	-1.210241
H	-0.647913	3.490106	-1.846926

C	2.043180	4.862285	0.581488
H	2.180286	2.846897	1.325658
C	1.428842	5.717431	-0.334612
H	-0.016080	5.876642	-1.929385
H	2.793411	5.243327	1.267520
H	1.701853	6.767744	-0.367222
C	-1.831018	2.403812	0.627488
C	-2.848207	2.970235	-0.149484
C	-1.578203	2.915003	1.906070
C	-3.599680	4.032460	0.345907
H	-3.052606	2.579361	-1.139800
C	-2.340323	3.970731	2.405733
H	-0.785173	2.482922	2.509095
C	-3.351291	4.533503	1.626018
H	-4.381867	4.467957	-0.268470
H	-2.139972	4.353680	3.401831
H	-3.942149	5.358572	2.012216
C	-3.116857	-0.001066	0.000097
C	-3.819040	0.122866	1.203425
C	-3.819022	-0.125450	-1.203211
C	-5.212724	0.117804	1.202498
H	-3.273974	0.229593	2.135796
C	-5.212705	-0.121352	-1.202208
H	-3.273946	-0.231756	-2.135623
C	-5.912442	-0.002026	0.000168
H	-5.750919	0.210367	2.140932
H	-5.750891	-0.214268	-2.140613
H	-6.998240	-0.002413	0.000202
C	-1.829502	-2.405160	-0.627279
C	-1.576717	-2.916410	-1.905832
C	-2.846086	-2.972115	0.150096
C	-2.338289	-3.972722	-2.405087
H	-0.784112	-2.483947	-2.509142
C	-3.597014	-4.034921	-0.344887
H	-3.050427	-2.581232	1.140420
C	-3.348671	-4.536020	-1.624983
H	-2.137963	-4.355721	-3.401170
H	-4.378730	-4.470824	0.269803
H	-3.939106	-5.361535	-2.010875
C	0.724127	-3.008563	0.251343
C	0.104911	-3.874906	1.161938
C	1.701311	-3.510717	-0.618133
C	0.465901	-5.218580	1.207616
H	-0.645102	-3.492090	1.845390
C	2.047673	-4.859836	-0.583664
H	2.182818	-2.843769	-1.326383
C	1.434077	-5.716247	0.331747
H	-0.010868	-5.878034	1.926249

H	2.798396	-5.239629	-1.269851
H	1.708146	-6.766307	0.363640

4-DT

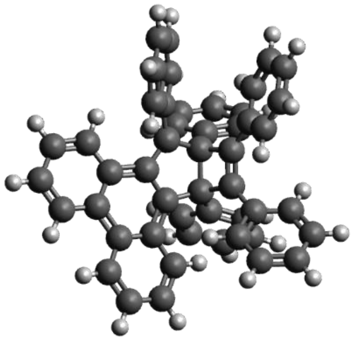

Table S69. Coordinates and energy for the optimized geometry of **4-DT**.

G = –1886.560446			
		**Coordinates / Å**	
**Atom**	**x**	**y**	**z**
C	4.957055	1.182381	-1.111024
H	5.544284	2.048170	-1.388078
C	5.534658	-0.070913	-1.233794
C	3.638090	1.339528	-0.643729
H	6.554764	-0.152879	-1.595605
C	4.818899	-1.233150	-0.892145
C	2.897419	0.150881	-0.328660
C	3.035251	2.655877	-0.448948
H	5.284078	-2.208794	-0.982784
C	3.520630	-1.122146	-0.447678
C	1.537464	0.279468	0.049845
C	1.671875	2.766865	-0.053478
C	3.789865	3.841201	-0.587395
H	2.960756	-2.009804	-0.185775
C	0.907605	1.540477	0.112754
C	0.569170	-0.828593	0.350194
C	1.144865	4.035388	0.261911
C	3.238693	5.081090	-0.317729
H	4.829825	3.791771	-0.883514
C	-0.479387	1.367577	0.335966
C	-0.779287	-0.042538	0.759830
C	-0.301026	-1.373463	-0.826342
C	1.059091	-1.848360	1.353492
H	0.129717	4.118252	0.626934
C	1.912271	5.178507	0.127552
H	3.844407	5.974886	-0.427642
C	-1.545020	2.277619	-0.014678
C	-1.441440	-0.763848	-0.435926
C	-1.264229	-0.261648	2.167994

C	0.015311	-2.317014	-1.894599
C	1.751292	-1.401794	2.489130
C	0.769571	-3.210628	1.229979
H	1.487985	6.145845	0.376529
C	-1.429548	3.071099	-1.181896
C	-2.763617	2.288465	0.699250
C	-2.776095	-0.623089	-1.014279
C	-0.951123	0.653215	3.180977
C	-1.930213	-1.446837	2.500365
C	1.223432	-2.239921	-2.608405
C	-0.912532	-3.321486	-2.232354
H	1.973080	-0.345184	2.603183
C	2.145249	-2.298244	3.478004
C	1.170901	-4.110968	2.219064
H	0.232620	-3.577543	0.363586
H	-0.514047	3.038450	-1.760505
C	-2.496971	3.842008	-1.613806
C	-3.814246	3.094740	0.281386
H	-2.864229	1.688954	1.594570
C	-3.922922	-0.530839	-0.207430
C	-2.920338	-0.538622	-2.411543
H	-0.422302	1.570950	2.939243
C	-1.310034	0.392332	4.503161
C	-2.284106	-1.709463	3.822324
H	-2.157825	-2.169892	1.724022
H	1.935049	-1.456413	-2.381097
C	1.496280	-3.144765	-3.632558
C	-0.634167	-4.224093	-3.253358
H	-1.843666	-3.390483	-1.679214
H	2.674819	-1.934515	4.353462
C	1.856662	-3.659023	3.345574
H	0.941192	-5.166280	2.105938
H	-2.408352	4.423594	-2.525391
C	-3.687808	3.864550	-0.877285
H	-4.738680	3.109904	0.848771
H	-3.829375	-0.593486	0.870534
C	-5.180684	-0.368570	-0.784412
C	-4.177647	-0.371652	-2.983228
H	-2.037671	-0.590621	-3.040308
H	-1.065062	1.112318	5.278104
C	-1.976566	-0.790076	4.827461
H	-2.799013	-2.633640	4.066489
H	2.431237	-3.069411	-4.179548
C	0.572470	-4.140167	-3.955376
H	-1.355235	-4.997612	-3.499586
H	2.164345	-4.359676	4.115971
H	-4.518394	4.477760	-1.212605
H	-6.058044	-0.301683	-0.148121

C	-5.312561	-0.286009	-2.171336
H	-4.272755	-0.300307	-4.062619
H	-2.255029	-0.994303	5.856811
H	0.789523	-4.847584	-4.749970
H	-6.293005	-0.152830	-2.618730

4-MT

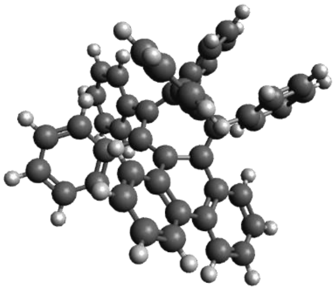

Table S70. Coordinates and energy for the optimized geometry of **4-MT**.

	G = –1886.532952		
		**Coordinates / Å**	
**Atom**	**x**	**y**	**z**
C	-2.194524	-3.885069	-2.540128
H	-3.228340	-4.210519	-2.578622
C	-1.278989	-4.418378	-3.441284
C	-1.806445	-2.920246	-1.599193
H	-1.602919	-5.158455	-4.166458
C	0.050444	-3.992417	-3.413254
C	-0.462172	-2.467244	-1.592814
C	-2.734115	-2.404500	-0.581017
H	0.772310	-4.399779	-4.114330
C	0.452292	-3.023176	-2.498453
C	-0.093169	-1.422775	-0.617573
C	-2.438237	-1.175940	0.062679
C	-3.891885	-3.100174	-0.204178
H	1.478098	-2.684359	-2.504442
C	-1.186619	-0.479805	-0.250839
C	1.063952	-1.310603	0.095614
C	-3.278177	-0.713320	1.094736
C	-4.725779	-2.616332	0.799438
H	-4.126771	-4.048279	-0.674766
C	-1.101584	0.929590	-0.222919
C	0.145344	1.655643	-0.130313
C	0.840817	-0.252544	1.116357
H	-3.023069	0.199480	1.618427
C	-4.410335	-1.425895	1.462882
H	-5.609906	-3.180046	1.080943

C	1.207180	1.018645	0.499189
H	-5.039385	-1.063068	2.269409
C	-2.340671	1.740471	-0.322133
C	-2.538988	2.824324	0.548118
C	-3.314754	1.460140	-1.293529
C	-3.694279	3.596757	0.462783
H	-1.788825	3.049031	1.299784
C	-4.461980	2.242304	-1.388422
H	-3.160497	0.632859	-1.978678
C	-4.657767	3.309096	-0.507128
H	-3.841809	4.424596	1.149744
H	-5.203161	2.021720	-2.150584
H	-5.555204	3.916177	-0.579080
C	0.296872	2.983524	-0.766704
C	0.901449	4.038584	-0.066770
C	-0.154612	3.203044	-2.077111
C	1.050708	5.287519	-0.664106
H	1.246398	3.875043	0.949514
C	0.001226	4.450736	-2.676050
H	-0.622217	2.390504	-2.623970
C	0.603111	5.496136	-1.971017
H	1.513159	6.098698	-0.109959
H	-0.347309	4.607895	-3.692301
H	0.720649	6.469711	-2.437290
C	2.643638	1.282978	0.351573
C	3.506573	0.883945	1.389957
C	3.196742	1.817218	-0.828796
C	4.886091	1.015764	1.255592
H	3.090285	0.473749	2.304564
C	4.573009	1.949841	-0.955942
H	2.546677	2.100781	-1.647249
C	5.421575	1.548669	0.083081
H	5.539392	0.701068	2.063020
H	4.990830	2.353842	-1.872807
H	6.497033	1.649329	-0.026433
C	0.195033	-0.418241	2.367216
C	-0.309916	-1.684002	2.769306
C	0.034931	0.700017	3.228125
C	-0.968465	-1.813496	3.979676
H	-0.192601	-2.537038	2.110299
C	-0.629776	0.556703	4.434496
H	0.446261	1.658939	2.932429
C	-1.132096	-0.696619	4.811012
H	-1.361321	-2.778040	4.282535
H	-0.751433	1.410451	5.092482
H	-1.645190	-0.805547	5.761247
C	2.400419	-1.922549	-0.028643
C	2.923919	-2.645005	1.055058

C	3.212919	-1.678726	-1.146381
C	4.220084	-3.153204	1.000930
H	2.312621	-2.810079	1.937029
C	4.514137	-2.172312	-1.190187
H	2.835883	-1.064018	-1.956753
C	5.017763	-2.916221	-0.120402
H	4.610362	-3.723788	1.838023
H	5.137953	-1.967683	-2.054665
H	6.032665	-3.300307	-0.156234

4-MT′

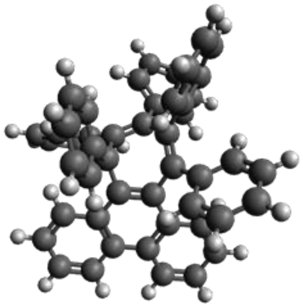

Table S71. Coordinates and energy for the optimized geometry of **4-MT′**.

G = –1886.547876			
		**Coordinates / Å**	
**Atom**	**x**	**y**	**z**
C	-4.860276	-1.887912	-0.062513
H	-5.403191	-2.583532	-0.690393
C	-5.554367	-1.203495	0.918978
C	-3.473644	-1.695089	-0.254588
H	-6.619821	-1.373304	1.038979
C	-4.890352	-0.294511	1.762686
C	-2.809902	-0.777047	0.610156
C	-2.714819	-2.378027	-1.293087
H	-5.439353	0.238118	2.532598
C	-3.534669	-0.088870	1.610881
C	-1.398426	-0.596498	0.452654
C	-1.307104	-2.163849	-1.410234
C	-3.336069	-3.247459	-2.219065
H	-3.016917	0.606926	2.261505
C	-0.649343	-1.262336	-0.491874
C	-0.602839	0.422180	1.180337
C	-0.597655	-2.793455	-2.460326
C	-2.616961	-3.866821	-3.223077
H	-4.401555	-3.430994	-2.156391
C	0.832017	-1.071363	-0.454236
C	1.444221	0.163786	-0.398180
C	-0.380893	1.588923	0.297228
H	0.462907	-2.607939	-2.568801

C	-1.237082	-3.630768	-3.351624
H	-3.123547	-4.527521	-3.919796
C	0.678067	1.431832	-0.534601
H	-0.674446	-4.101044	-4.152044
C	-0.025456	0.245162	2.445463
C	0.843176	1.241231	2.982959
C	-0.260456	-0.954604	3.180554
C	1.463453	1.030001	4.199699
H	1.016219	2.150457	2.418754
C	0.365256	-1.148977	4.395778
H	-0.931138	-1.702535	2.773114
C	1.226471	-0.160670	4.902551
H	2.132172	1.778524	4.609326
H	0.194493	-2.059021	4.959840
H	1.714392	-0.320748	5.858824
C	-1.476131	2.577012	0.238644
C	-2.046200	2.955878	-0.989173
C	-2.002507	3.101743	1.431276
C	-3.103482	3.858163	-1.018643
H	-1.660146	2.532869	-1.909576
C	-3.061314	4.007801	1.396513
H	-1.571124	2.815257	2.385434
C	-3.613129	4.387060	0.172689
H	-3.539462	4.144371	-1.970829
H	-3.454412	4.413428	2.323481
H	-4.442052	5.087664	0.143985
C	1.059359	2.408663	-1.573942
C	1.208872	3.769357	-1.268924
C	1.276138	1.969941	-2.889763
C	1.569834	4.675143	-2.263371
H	1.052215	4.106680	-0.249479
C	1.632197	2.879414	-3.883921
H	1.158952	0.915935	-3.125257
C	1.781066	4.233190	-3.572092
H	1.691772	5.725568	-2.016960
H	1.793280	2.532515	-4.900112
H	2.062395	4.941130	-4.345829
C	2.875056	0.321417	-0.004240
C	3.286283	-0.254414	1.212411
C	3.800018	1.089800	-0.728294
C	4.582049	-0.072777	1.687587
H	2.584812	-0.852412	1.782800
C	5.101046	1.259288	-0.254975
H	3.514313	1.536328	-1.671908
C	5.497158	0.684302	0.953629
H	4.875161	-0.523612	2.631094
H	5.807461	1.843419	-0.837443
H	6.510139	0.823538	1.319207

C	1.624989	-2.323335	-0.368743
C	1.164312	-3.381315	0.432858
C	2.822057	-2.490161	-1.086758
C	1.897763	-4.560695	0.541188
H	0.232206	-3.273502	0.977594
C	3.542462	-3.676276	-0.993348
H	3.175916	-1.690024	-1.726815
C	3.088013	-4.712686	-0.172253
H	1.535354	-5.363462	1.176177
H	4.458992	-3.794343	-1.563388
H	3.654480	-5.635978	-0.096941

β-NO_2-TP

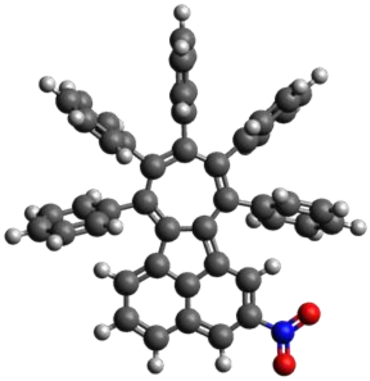

Table S72. Coordinates and energy for the optimized geometry of **β-NO_2-TP**.

G = –2013.678023			
	Coordinates / Å		
**Atom**	**x**	**y**	**z**
C	-1.896429	0.576431	-0.009182
C	-1.689692	-0.826298	0.015698
C	-0.938893	1.617032	0.059501
C	0.459943	1.502175	0.146517
C	1.269669	0.303131	0.031286
C	0.856122	-1.037819	0.050876
C	-0.469509	-1.541983	0.051509
C	2.683427	0.735867	-0.039961
C	3.898304	0.104792	-0.270004
C	5.064433	0.899221	-0.259520
C	5.084104	2.263716	-0.024202
H	6.025729	2.798239	-0.016789
C	1.382022	2.647727	0.299177
C	1.252131	4.009733	0.569226
C	2.689607	2.136426	0.152451
C	2.411908	4.817512	0.646666
C	3.855854	2.925686	0.193761
C	3.685174	4.311665	0.451066
H	4.551683	4.963412	0.500571
C	1.946076	-2.063138	0.105059

C	2.315443	-2.779588	-1.037776
C	2.620841	-2.279468	1.311956
C	3.362099	-3.698803	-0.974060
H	1.793506	-2.609874	-1.973605
C	3.658139	-3.208287	1.374750
H	2.337280	-1.715727	2.195475
C	4.034133	-3.915257	0.230916
H	3.652723	-4.244153	-1.866465
H	4.176791	-3.373049	2.314005
H	4.848853	-4.631041	0.277347
C	-0.586492	-3.039958	0.045923
C	-0.355089	-3.776217	1.212312
C	-0.919487	-3.707447	-1.137513
C	-0.462896	-5.165485	1.195816
H	-0.095738	-3.262621	2.132269
C	-1.016011	-5.097504	-1.154449
H	-1.100114	-3.137714	-2.043375
C	-0.790627	-5.829838	0.012483
H	-0.288327	-5.728050	2.107890
H	-1.269935	-5.606767	-2.079129
H	-0.869872	-6.912603	-0.000178
C	-2.935589	-1.666728	0.003631
C	-3.685219	-1.805760	-1.169067
C	-3.354812	-2.312717	1.171405
C	-4.839563	-2.585753	-1.174090
H	-3.363865	-1.303852	-2.075666
C	-4.516219	-3.082840	1.167339
H	-2.774464	-2.208955	2.082536
C	-5.260184	-3.223451	-0.005428
H	-5.411444	-2.692040	-2.090891
H	-4.837227	-3.574643	2.080566
H	-6.162796	-3.826919	-0.008811
C	-3.327960	1.025480	-0.089256
C	-4.155340	0.955065	1.036406
C	-3.839168	1.520781	-1.293177
C	-5.480577	1.378382	0.957619
H	-3.762038	0.569504	1.971477
C	-5.167888	1.933361	-1.372568
H	-3.198060	1.576561	-2.167147
C	-5.991087	1.865451	-0.247164
H	-6.114377	1.324311	1.837535
H	-5.558058	2.309334	-2.313467
H	-7.025212	2.190500	-0.308488
C	-1.483008	3.011261	0.010428
C	-2.079853	3.588078	1.135925
C	-1.368556	3.749370	-1.172984
C	-2.548562	4.900124	1.079584
H	-2.168079	3.015068	2.052858

C	-1.849902	5.056115	-1.230000
H	-0.899498	3.301268	-2.043594
C	-2.435454	5.635663	-0.102508
H	-3.003042	5.346234	1.958730
H	-1.761949	5.622249	-2.152057
H	-2.804112	6.655881	-0.144768
H	0.294778	4.479150	0.734549
H	4.008115	-0.948236	-0.466218
H	2.285665	5.873757	0.858507
N	6.346041	0.219862	-0.502929
O	6.323405	-0.993720	-0.708716
O	7.371421	0.900759	-0.487708

β-NO_2-DT

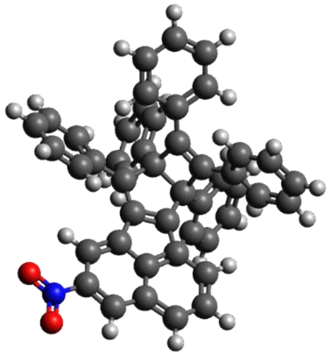

Table S73. Coordinates and energy for the optimized geometry of **β-NO_2-DT.**

G = –2013.647593			
	Coordinates / Å		
**Atom**	**x**	**y**	**z**
C	4.246209	-1.849043	-0.150074
C	4.266187	-3.269597	-0.209385
C	2.986854	-1.252335	-0.001964
C	3.086853	-4.005003	-0.105970
C	1.774441	-1.996801	0.120572
C	1.831007	-3.390029	0.069409
C	0.742311	-1.024884	0.304179
C	2.763628	0.148253	0.077263
C	1.301906	0.283894	0.246100
C	-0.723098	-1.001300	0.486196
C	3.858356	0.984422	0.042983
C	5.362270	-0.975089	-0.210159
C	0.311126	1.256849	0.326683
C	-1.045164	0.583542	0.503865
C	-1.462189	-1.005309	-0.875639
C	-1.297934	-1.836546	1.603946
C	5.136955	0.384888	-0.105544
C	0.501619	2.678656	0.129091

C	-1.780841	0.308269	-0.847115
C	-1.863724	1.055364	1.681836
C	-1.577148	-2.111617	-1.818468
C	-0.496329	-2.258629	2.670705
C	-2.667971	-2.126730	1.624762
C	1.478111	3.114058	-0.797000
C	-0.302151	3.646775	0.773621
C	-2.613654	1.169684	-1.683505
C	-1.245563	1.139875	2.938650
C	-3.228846	1.331638	1.573851
C	-1.499137	-3.443442	-1.373427
C	-1.706875	-1.865681	-3.198724
H	0.564332	-2.028435	2.679877
C	-1.052690	-2.966294	3.736618
C	-3.223817	-2.829362	2.691306
H	-3.298444	-1.800294	0.804461
H	2.050821	2.381651	-1.352636
C	1.647116	4.465570	-1.064297
C	-0.106506	4.998581	0.520292
H	-1.055003	3.337348	1.484525
C	-2.337315	2.536104	-1.862430
C	-3.756354	0.625052	-2.303604
H	-0.186209	0.920417	3.034133
C	-1.979342	1.501997	4.064452
C	-3.963743	1.696660	2.703950
H	-3.723661	1.264388	0.612778
H	-1.416276	-3.650897	-0.312470
C	-1.561787	-4.498852	-2.281442
C	-1.766994	-2.922620	-4.101055
H	-1.746079	-0.843293	-3.558435
H	-0.417761	-3.287547	4.556798
C	-2.417763	-3.253036	3.750442
H	-4.287424	-3.047708	2.693454
H	2.382448	4.781925	-1.796607
C	0.863036	5.412595	-0.397788
H	-0.716310	5.732733	1.036369
H	-1.466705	2.976958	-1.397626
C	-3.167585	3.328049	-2.652997
C	-4.582826	1.419782	-3.090683
H	-3.995144	-0.422242	-2.150753
H	-1.487919	1.562499	5.030758
C	-3.343620	1.783005	3.949641
H	-5.023660	1.911134	2.605649
H	-1.507591	-5.521307	-1.919881
C	-1.696383	-4.243247	-3.646839
H	-1.862150	-2.716424	-5.162946
H	-2.851728	-3.802737	4.580137
H	1.002085	6.469883	-0.599960

H	-2.934316	4.380301	-2.785031
C	-4.290817	2.775425	-3.269930
H	-5.460810	0.984318	-3.558113
H	-3.917246	2.066299	4.826931
H	-1.743761	-5.065820	-4.353983
H	-4.938105	3.395979	-3.882163
H	6.371932	-1.348732	-0.325880
H	5.212416	-3.787224	-0.331351
H	3.794002	2.060255	0.127773
H	0.936945	-3.995295	0.161765
H	3.137010	-5.087114	-0.151783
N	6.301315	1.283057	-0.154166
O	6.094734	2.492519	-0.051531
O	7.414789	0.779523	-0.294829

β-NO_2-DT′

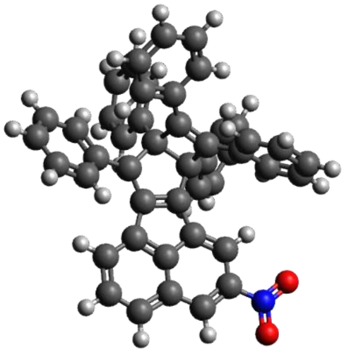

Table S74. Coordinates and energy for the optimized geometry of **β-NO_2-DT′**.

G = –2013.647570			
		**Coordinates / Å**	
**Atom**	**x**	**y**	**z**
C	3.764489	2.785892	-0.224425
C	4.908743	1.956857	-0.265612
C	2.532339	2.134624	-0.056028
C	4.766552	0.577753	-0.122506
C	2.403170	0.720758	0.109388
C	3.539000	-0.077540	0.078747
C	1.004074	0.479292	0.302426
C	1.279762	2.794135	0.005589
C	0.295277	1.707439	0.204696
C	0.115445	-0.678550	0.523717
C	1.280585	4.175876	-0.062872
C	3.728908	4.207699	-0.321538
C	-1.081975	1.488466	0.277111
C	-1.346555	0.006779	0.505566
C	-0.314684	-1.333792	-0.812958
C	0.441778	-1.586849	1.684086

C	2.516270	4.863979	-0.230629
C	-2.116967	2.464543	0.022700
C	-1.553748	-0.796169	-0.820841
C	-2.222583	-0.331649	1.688508
C	0.507117	-2.134194	-1.712222
C	1.235036	-1.136539	2.745738
C	-0.118500	-2.868732	1.747777
C	-1.865094	3.507362	-0.900521
C	-3.406233	2.364272	0.596921
C	-2.731979	-0.974689	-1.669022
C	-1.940545	0.264956	2.926769
C	-3.250212	-1.274397	1.604223
C	1.655633	-2.790319	-1.234502
C	0.203633	-2.213792	-3.084741
H	1.663286	-0.139343	2.721835
C	1.472097	-1.956983	3.848776
C	0.114857	-3.685870	2.851519
H	-0.738040	-3.225961	0.931949
H	-0.908342	3.552833	-1.405163
C	-2.860878	4.414750	-1.232397
C	-4.387234	3.294769	0.281043
H	-3.623180	1.574665	1.301814
C	-3.576675	0.091541	-2.021991
C	-3.046628	-2.268582	-2.129978
H	-1.139766	0.994392	3.004809
C	-2.677732	-0.072787	4.057915
C	-3.990441	-1.610689	2.739676
H	-3.479016	-1.747493	0.657146
H	1.890431	-2.758288	-0.176544
C	2.476235	-3.507932	-2.102378
C	1.025300	-2.932357	-3.947139
H	-0.668553	-1.698081	-3.471150
H	2.090629	-1.594275	4.664163
C	0.912346	-3.233484	3.905123
H	-0.324929	-4.677975	2.886727
H	-2.660862	5.191117	-1.963348
C	-4.121374	4.318602	-0.633171
H	-5.365835	3.218100	0.743053
H	-3.347110	1.095698	-1.693738
C	-4.697410	-0.130277	-2.820190
C	-4.167171	-2.485140	-2.925304
H	-2.409948	-3.100633	-1.846534
H	-2.447199	0.395597	5.009984
C	-3.707579	-1.013145	3.967198
H	-4.787873	-2.343377	2.659854
H	3.358484	-4.008569	-1.715347
C	2.164405	-3.581621	-3.460757
H	0.781399	-2.980284	-5.004225

H	1.095918	-3.872554	4.763483
H	-4.897015	5.034646	-0.885716
H	-5.335616	0.706182	-3.088780
C	-4.998036	-1.415762	-3.272859
H	-4.397271	-3.489257	-3.268450
H	-4.283741	-1.276985	4.848868
H	2.804431	-4.139141	-4.137807
H	-5.874489	-1.585480	-3.890856
H	4.649575	4.766026	-0.454463
H	5.899848	2.372189	-0.403533
H	0.367101	4.753984	0.015106
H	3.513720	-1.151601	0.198390
H	2.493997	5.946849	-0.288474
N	5.981166	-0.251806	-0.175476
O	7.059646	0.315969	-0.338512
O	5.844716	-1.468638	-0.055052

β-Cl-TP

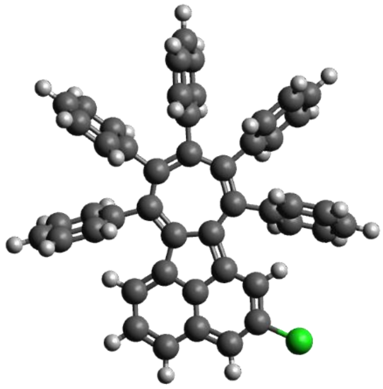

Table S75. Coordinates and energy for the optimized geometry of **β-Cl-TP**.

G = –2268.779623			
		**Coordinates / Å**	
**Atom**	**x**	**y**	**z**
C	-1.773541	0.560344	-0.007913
C	-1.553646	-0.839628	0.011166
C	-0.824835	1.610141	0.053750
C	0.575446	1.510257	0.128646
C	1.397712	0.317216	0.006958
C	0.993968	-1.027101	0.030689
C	-0.326341	-1.543383	0.039532
C	2.805315	0.762706	-0.073587
C	4.028665	0.142178	-0.309196
C	5.195436	0.938816	-0.308104
C	5.195447	2.303164	-0.071934
H	6.125464	2.860733	-0.066581
C	1.487077	2.662929	0.273876
C	1.345429	4.024219	0.541939
C	2.798685	2.161707	0.118989

C	2.500215	4.837932	0.612414
C	3.959485	2.956771	0.152718
C	3.777126	4.340192	0.410850
H	4.637971	5.000088	0.456888
C	2.092807	-2.043576	0.080210
C	2.464275	-2.756747	-1.063946
C	2.774380	-2.254120	1.284209
C	3.521138	-3.664610	-1.005299
H	1.936557	-2.592287	-1.997464
C	3.821969	-3.171511	1.342631
H	2.488872	-1.693004	2.168739
C	4.201354	-3.873569	0.196694
H	3.812941	-4.207557	-1.898874
H	4.346069	-3.331129	2.279786
H	5.023312	-4.581407	0.239722
C	-0.429792	-3.042730	0.036476
C	-0.189756	-3.774808	1.203706
C	-0.759713	-3.715814	-1.144587
C	-0.285636	-5.165037	1.190551
H	0.067286	-3.256848	2.121902
C	-0.844959	-5.106695	-1.158402
H	-0.947647	-3.149357	-2.051063
C	-0.610684	-5.834730	0.009495
H	-0.104125	-5.724152	2.103437
H	-1.097086	-5.620060	-2.081343
H	-0.681025	-6.918167	-0.000636
C	-2.791524	-1.692488	0.001473
C	-3.541805	-1.837704	-1.170008
C	-3.202603	-2.344641	1.168699
C	-4.688045	-2.629741	-1.174645
H	-3.227396	-1.330677	-2.076249
C	-4.355547	-3.127373	1.165225
H	-2.622023	-2.236101	2.079131
C	-5.099894	-3.274143	-0.006585
H	-5.260385	-2.740285	-2.090696
H	-4.669814	-3.624118	2.078162
H	-5.995952	-3.887348	-0.009596
C	-3.210787	0.995237	-0.070216
C	-4.022685	0.914206	1.065973
C	-3.743866	1.487177	-1.265923
C	-5.353373	1.323399	1.006028
H	-3.612606	0.531292	1.994936
C	-5.077748	1.886139	-1.326727
H	-3.115219	1.552614	-2.148209
C	-5.885236	1.807372	-0.190705
H	-5.974671	1.260779	1.894290
H	-5.484276	2.259963	-2.261576
H	-6.923476	2.121627	-0.237552

C	-1.385409	2.998350	0.010213
C	-1.978398	3.568655	1.140999
C	-1.294853	3.736177	-1.175340
C	-2.467765	4.873226	1.087572
H	-2.048064	2.996042	2.059825
C	-1.796693	5.035292	-1.229622
H	-0.828010	3.293644	-2.049986
C	-2.379107	5.608018	-0.097058
H	-2.919377	5.314101	1.970878
H	-1.727068	5.601018	-2.153550
H	-2.764058	6.622333	-0.137278
H	0.385441	4.486954	0.709813
H	4.132792	-0.912821	-0.504027
H	2.369462	5.893965	0.823816
Cl	6.723880	0.127068	-0.608703

β-Cl-DT

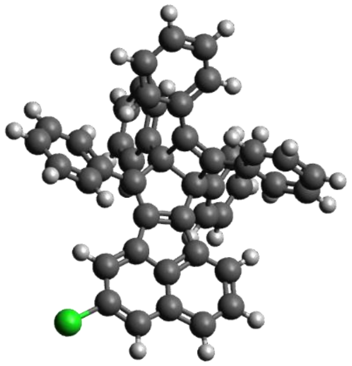

Table S76. Coordinates and energy for the optimized geometry of **β-Cl-DT**.

G = –2268.749575			
	Coordinates / Å		
**Atom**	**x**	**y**	**z**
C	4.515544	-1.433898	-0.180927
C	4.656491	-2.846522	-0.234128
C	3.208709	-0.953800	-0.027581
C	3.546151	-3.684676	-0.120286
C	2.065303	-1.801873	0.105566
C	2.242074	-3.186092	0.059811
C	0.955618	-0.923874	0.291173
C	2.867619	0.420282	0.047722
C	1.401037	0.430701	0.226047
C	-0.506480	-1.021664	0.480909
C	3.887490	1.349300	0.001845
C	5.553060	-0.463587	-0.252686
C	0.332873	1.313996	0.310058
C	-0.960840	0.531023	0.505491
C	-1.258097	-1.084177	-0.872517
C	-1.004830	-1.902349	1.600932

C	5.219646	0.874061	-0.152607
C	0.399799	2.747479	0.102367
C	-1.691621	0.195951	-0.832764
C	-1.792547	0.934658	1.699183
C	-1.288784	-2.193457	-1.818110
C	-0.166609	-2.256640	2.664246
C	-2.345764	-2.306030	1.627852
C	1.317043	3.256661	-0.845396
C	-0.467840	3.648844	0.759363
C	-2.613224	0.981418	-1.651040
C	-1.158445	1.074411	2.943089
C	-3.177840	1.095850	1.618743
C	-1.076346	-3.512877	-1.380206
C	-1.469659	-1.958120	-3.194479
H	0.871538	-1.940411	2.668285
C	-0.657547	-3.008396	3.732095
C	-2.836702	-3.053001	2.696197
H	-3.004973	-2.033097	0.810564
H	1.940555	2.570911	-1.406084
C	1.366746	4.616187	-1.122949
C	-0.392021	5.010876	0.494856
H	-1.177802	3.282071	1.487059
C	-2.465624	2.367917	-1.826953
C	-3.713922	0.338172	-2.252255
H	-0.082352	0.945943	3.016456
C	-1.897159	1.376797	4.083229
C	-3.917854	1.401297	2.763120
H	-3.684343	0.984819	0.667820
H	-0.951903	-3.712888	-0.321942
C	-1.057955	-4.567689	-2.290749
C	-1.448376	-3.014162	-4.099561
H	-1.612584	-0.943223	-3.549079
H	0.005673	-3.275795	4.549279
C	-1.993836	-3.408149	3.751789
H	-3.878395	-3.359370	2.702393
H	2.058132	4.987822	-1.872047
C	0.519620	5.498770	-0.445776
H	-1.050749	5.694774	1.019909
H	-1.630289	2.884310	-1.375136
C	-3.379621	3.084652	-2.596798
C	-4.624521	1.057939	-3.018465
H	-3.854106	-0.727024	-2.100373
H	-1.393152	1.480990	5.039323
C	-3.282203	1.542441	3.995964
H	-4.993780	1.526257	2.686189
H	-0.899635	-5.581175	-1.934528
C	-1.244069	-4.323166	-3.652141
H	-1.584535	-2.815734	-5.158479

H	-2.376787	-3.992405	4.583025
H	0.564443	6.562580	-0.656658
H	-3.245369	4.154375	-2.726589
C	-4.460168	2.435163	-3.195334
H	-5.468732	0.546536	-3.471032
H	-3.859733	1.779065	4.884473
H	-1.228167	-5.145118	-4.361431
H	-5.173100	2.996990	-3.791141
H	6.587117	-0.765269	-0.373670
H	5.643271	-3.282046	-0.359816
H	3.713991	2.414837	0.084802
H	1.405830	-3.867826	0.160648
H	3.694512	-4.758074	-0.162345
Cl	6.496443	2.076665	-0.219266

β-Cl-DT′

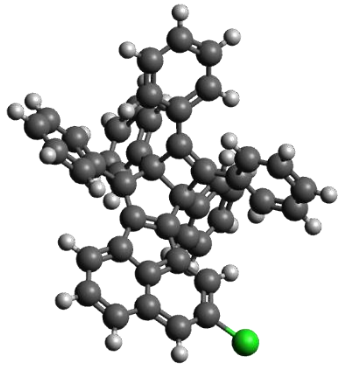

Table S77. Coordinates and energy for the optimized geometry of **β-Cl-DT′**.

G = –2268.749594			
		**Coordinates / Å**	
**Atom**	**x**	**y**	**z**
C	3.888737	2.734595	-0.207986
C	5.031373	1.898125	-0.228601
C	2.652529	2.093188	-0.042677
C	4.895815	0.516658	-0.071718
C	2.519472	0.681498	0.133232
C	3.657850	-0.121992	0.122179
C	1.122003	0.445640	0.313799
C	1.402475	2.759382	0.001915
C	0.414405	1.678596	0.203656
C	0.221525	-0.705764	0.519649
C	1.404973	4.138912	-0.089525
C	3.857308	4.155009	-0.323713
C	-0.958243	1.470142	0.275049
C	-1.239074	-0.013484	0.492598
C	-0.194193	-1.339713	-0.831661
C	0.527486	-1.633257	1.670047

C	2.645908	4.817766	-0.255415
C	-1.978346	2.471556	0.033429
C	-1.438242	-0.809416	-0.838361
C	-2.127206	-0.348113	1.667338
C	0.649513	-2.107007	-1.740645
C	1.342043	-1.219245	2.729983
C	-0.072087	-2.897856	1.725551
C	-1.737054	3.467122	-0.940915
C	-3.232520	2.448260	0.684420
C	-2.612535	-0.982778	-1.689056
C	-1.835546	0.225324	2.914418
C	-3.175260	-1.266514	1.568921
C	1.746898	-2.838820	-1.252488
C	0.424928	-2.070593	-3.130008
H	1.803820	-0.237361	2.710846
C	1.560048	-2.057538	3.823844
C	0.142081	-3.732645	2.819757
H	-0.707841	-3.226440	0.910211
H	-0.805218	3.457318	-1.493181
C	-2.713945	4.403267	-1.252893
C	-4.193202	3.406725	0.385776
H	-3.437702	1.697671	1.434524
C	-3.574737	0.029136	-1.851167
C	-2.817056	-2.217711	-2.337589
H	-1.017972	0.934623	3.003614
C	-2.584280	-0.108577	4.039078
C	-3.927192	-1.599538	2.697790
H	-3.409596	-1.725214	0.616195
H	1.924008	-2.890736	-0.184206
C	2.588728	-3.523032	-2.127268
C	1.268687	-2.754472	-3.999313
H	-0.404022	-1.491558	-3.522323
H	2.195564	-1.722445	4.638045
C	0.960103	-3.315830	3.872508
H	-0.329003	-4.710518	2.848614
H	-2.523735	5.143914	-2.022729
C	-3.941246	4.382209	-0.583319
H	-5.144989	3.389730	0.906510
H	-3.444521	0.982299	-1.358321
C	-4.692942	-0.179449	-2.655700
C	-3.935278	-2.421702	-3.138670
H	-2.098522	-3.018028	-2.196023
H	-2.345994	0.342422	4.997638
C	-3.635618	-1.023423	3.933379
H	-4.740434	-2.313299	2.606452
H	3.428150	-4.087442	-1.732548
C	2.353161	-3.483994	-3.502364
H	1.085319	-2.712196	-5.068811

H	1.128448	-3.968407	4.723842
H	-4.701503	5.119481	-0.821268
H	-5.421725	0.616847	-2.773776
C	-4.877583	-1.401753	-3.303484
H	-4.077719	-3.380425	-3.628156
H	-4.220931	-1.284567	4.809843
H	3.010478	-4.015398	-4.183861
H	-5.751721	-1.562860	-3.927069
H	4.780642	4.709808	-0.454777
H	6.020117	2.324358	-0.361716
H	0.491082	4.718972	-0.032995
H	3.610398	-1.195260	0.256053
H	2.630302	5.899895	-0.330716
Cl	6.339226	-0.475796	-0.104269

β-Me-TP

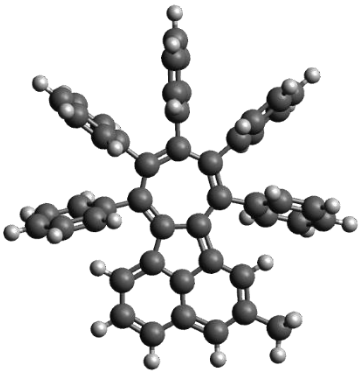

Table S78. Coordinates and energy for the optimized geometry of **β-Me-TP**.

G = –1848.470964			
	**Coordinates / Å**		
**Atom**	**x**	**y**	**z**
C	-1.613813	0.537456	-0.011650
C	-1.369339	-0.857092	-0.006009
C	-0.681932	1.603412	0.049309
C	0.720097	1.528576	0.099943
C	1.563355	0.348345	-0.041639
C	1.180448	-1.002877	-0.000828
C	-0.129845	-1.540012	0.028696
C	2.957944	0.816003	-0.148387
C	4.191161	0.224827	-0.409778
C	5.374215	1.010301	-0.429068
C	5.319497	2.375843	-0.177599
H	6.235327	2.960560	-0.177621
C	1.614575	2.694298	0.234063
C	1.457806	4.053739	0.511165
C	2.930288	2.213908	0.052479
C	2.601908	4.880719	0.565774
C	4.081448	3.021321	0.070674

C	3.883105	4.398659	0.339867
H	4.735425	5.070762	0.376254
C	2.297173	-2.000805	0.043708
C	2.657500	-2.728348	-1.094894
C	3.007768	-2.178908	1.235986
C	3.731978	-3.615752	-1.043840
H	2.106551	-2.590823	-2.019362
C	4.073496	-3.075537	1.287887
H	2.730263	-1.607907	2.116761
C	4.442166	-3.790432	0.146209
H	4.013747	-4.170019	-1.933800
H	4.619217	-3.210106	2.216718
H	5.277353	-4.483078	0.184028
C	-0.208661	-3.040834	0.065766
C	0.043816	-3.729368	1.256658
C	-0.528754	-3.759046	-1.091047
C	-0.031834	-5.120657	1.291738
H	0.295169	-3.175800	2.155724
C	-0.594613	-5.150571	-1.056787
H	-0.726251	-3.227477	-2.016214
C	-0.349253	-5.834964	0.135129
H	0.159311	-5.645436	2.222926
H	-0.840405	-5.698948	-1.961157
H	-0.404809	-6.919002	0.162346
C	-2.591732	-1.732039	-0.040957
C	-3.306083	-1.889461	-1.233042
C	-3.021130	-2.396535	1.112457
C	-4.434430	-2.706404	-1.272148
H	-2.975319	-1.374850	-2.129445
C	-4.155283	-3.205171	1.074361
H	-2.468126	-2.279140	2.038705
C	-4.863527	-3.364524	-0.118214
H	-4.978705	-2.826192	-2.204066
H	-4.483371	-3.712499	1.976599
H	-5.744826	-3.998111	-0.147988
C	-3.060040	0.947355	-0.048264
C	-3.849425	0.840160	1.101674
C	-3.625022	1.444475	-1.227138
C	-5.187602	1.228059	1.072754
H	-3.415185	0.453910	2.018222
C	-4.966001	1.822824	-1.257310
H	-3.015605	1.533429	-2.120558
C	-5.750346	1.717740	-0.107289
H	-5.790260	1.144703	1.972128
H	-5.396192	2.201665	-2.179514
H	-6.794104	2.016089	-0.130384
C	-1.268368	2.981747	0.027490
C	-1.851147	3.532197	1.173253

C	-1.214486	3.730481	-1.153411
C	-2.367112	4.827188	1.139280
H	-1.892583	2.951346	2.088687
C	-1.742668	5.019779	-1.188403
H	-0.754856	3.303927	-2.039779
C	-2.315230	5.572456	-0.040941
H	-2.810782	5.252402	2.034267
H	-1.701180	5.593771	-2.108979
H	-2.720833	6.579221	-0.066158
H	0.494350	4.502192	0.697623
C	6.685144	0.321771	-0.718728
H	6.838876	-0.526593	-0.042316
H	6.700626	-0.074469	-1.741094
H	7.529490	1.007055	-0.608555
H	4.292287	-0.830597	-0.612779
H	2.462125	5.934174	0.785093

β-Me-DT

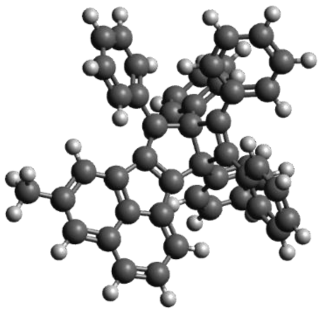

Table S79. Coordinates and energy for the optimized geometry of **β-Me-DT**.

G = –1848.441387			
		**Coordinates / Å**	
**Atom**	**x**	**y**	**z**
C	4.867838	-0.067806	-0.193117
C	5.382167	-1.388650	-0.239139
C	3.480644	0.039143	-0.032029
H	6.449568	-1.542930	-0.368852
C	4.540971	-2.499309	-0.113825
C	2.608959	-1.086266	0.112864
C	3.153819	-2.373524	0.071759
C	1.307435	-0.539940	0.300073
C	2.785811	1.271852	0.037840
C	1.371780	0.887247	0.225778
C	-0.075283	-1.026564	0.490506
C	3.525647	2.435635	-0.024289
C	5.596302	1.152861	-0.278252
C	0.106210	1.447179	0.307995
C	-0.931678	0.345780	0.501964
C	-0.774725	-1.298870	-0.864165

C	-0.322792	-2.001566	1.616269
C	4.946077	2.374299	-0.188600
H	6.674305	1.124257	-0.406368
C	-0.214673	2.847630	0.095501
C	-1.537214	-0.182084	-0.836604
C	-1.848727	0.518390	1.688856
C	-0.492321	-2.379246	-1.801963
C	0.588062	-2.130558	2.670883
C	-1.517791	-2.731450	1.656934
C	0.514538	3.570583	-0.874996
C	-1.273699	3.494187	0.769930
C	-2.625212	0.325913	-1.669173
C	-1.280251	0.804836	2.939395
C	-3.229516	0.328417	1.596264
C	0.045594	-3.596918	-1.348364
C	-0.689095	-2.200188	-3.184541
H	1.513171	-1.563325	2.663477
C	0.313293	-2.979004	3.744015
C	-1.793782	-3.575116	2.730300
H	-2.232981	-2.635396	0.846823
H	1.288923	3.067900	-1.441836
C	0.195302	4.891771	-1.161207
C	-1.568174	4.824892	0.496436
H	-1.843818	2.960711	1.517480
C	-2.879172	1.701021	-1.811576
C	-3.479557	-0.587866	-2.318811
H	-0.206345	0.944860	3.022753
C	-2.080478	0.907516	4.073447
C	-4.032187	0.433847	2.734427
H	-3.683780	0.098460	0.640259
H	0.190971	-3.755581	-0.285778
C	0.364431	-4.610831	-2.249738
C	-0.367327	-3.214496	-4.080566
H	-1.080966	-1.257476	-3.550763
H	1.030773	-3.068136	4.554295
C	-0.878492	-3.702883	3.777702
H	-2.724395	-4.134407	2.747286
H	0.747481	5.425016	-1.928233
C	-0.841017	5.525909	-0.469657
H	-2.372447	5.315708	1.034761
H	-2.242645	2.421785	-1.317634
C	-3.939325	2.147735	-2.597803
C	-4.537418	-0.137506	-3.101349
H	-3.313404	-1.652559	-2.192851
H	-1.626392	1.128167	5.034813
C	-3.462449	0.723337	3.973544
H	-5.104558	0.286404	2.647764
H	0.772688	-5.547213	-1.881358

C	0.158927	-4.424464	-3.617565
H	-0.520224	-3.058847	-5.144373
H	-1.093613	-4.362416	4.613031
H	-1.085223	6.560815	-0.687882
H	-4.115204	3.214399	-2.700520
C	-4.770660	1.233506	-3.246453
H	-5.186960	-0.856376	-3.591698
H	-4.088249	0.804016	4.857234
H	0.409039	-5.214002	-4.319737
H	-5.598356	1.584236	-3.855456
H	3.056695	3.411067	0.053550
C	5.714374	3.670062	-0.264739
H	5.387834	4.268797	-1.123221
H	5.549033	4.276679	0.633166
H	6.788250	3.491912	-0.363143
H	4.976564	-3.491840	-0.152112
H	2.533260	-3.255348	0.180153

β-Me-DT′

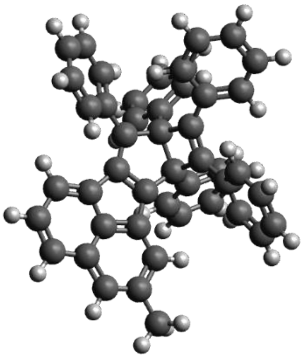

Table S80. Coordinates and energy for the optimized geometry of **β-Me-DT′**.

G = –1848.442516			
	**Coordinates / Å**		
**Atom**	**x**	**y**	**z**
C	4.117181	2.603007	-0.202677
C	5.231201	1.724858	-0.226744
C	2.864044	2.000536	-0.035759
H	6.226822	2.139423	-0.361241
C	5.095482	0.337347	-0.077398
C	2.695440	0.590228	0.136354
C	3.819259	-0.235889	0.118187
C	1.297166	0.391905	0.316943
C	1.633794	2.700789	0.011990
C	0.617972	1.646436	0.212317
C	0.362194	-0.734725	0.513995
C	1.673932	4.080602	-0.080560
C	4.125081	4.022565	-0.316758
C	-0.755908	1.474802	0.282378

C	-1.079925	-0.002380	0.492716
C	-0.072144	-1.346872	-0.840585
C	0.639970	-1.679711	1.657450
C	2.932846	4.722329	-0.247418
H	5.063845	4.551240	-0.449101
C	-1.749294	2.507912	0.045136
C	-1.303144	-0.785604	-0.840038
C	-1.974205	-0.314076	1.668773
C	0.752556	-2.126219	-1.757008
C	1.468173	-1.299609	2.719552
C	0.001068	-2.925298	1.705156
C	-1.488819	3.486052	-0.940409
C	-2.993140	2.532287	0.713526
C	-2.482515	-0.919243	-1.690934
C	-1.662168	0.245620	2.917199
C	-3.050527	-1.199221	1.570471
C	1.829416	-2.891375	-1.274177
C	0.534151	-2.068368	-3.146642
H	1.961442	-0.333168	2.705630
C	1.660345	-2.152042	3.807368
C	0.189837	-3.774488	2.793002
H	-0.645937	-3.227591	0.888456
H	-0.563350	3.442630	-1.502046
C	-2.438369	4.452439	-1.247202
C	-3.926744	3.519119	0.418498
H	-3.212619	1.795330	1.473264
C	-3.431448	0.110153	-1.817249
C	-2.708740	-2.131153	-2.374633
H	-0.822366	0.928548	3.006336
C	-2.418714	-0.067840	4.042549
C	-3.810469	-1.511870	2.699868
H	-3.300708	-1.647674	0.616853
H	2.003140	-2.956857	-0.206132
C	2.655444	-3.588225	-2.154063
C	1.362406	-2.764494	-4.021229
H	-0.277578	-1.462891	-3.535083
H	2.306636	-1.842791	4.623418
C	1.021353	-3.391301	3.847940
H	-0.311964	-4.737194	2.815335
H	-2.233265	5.180716	-2.025108
C	-3.656804	4.477438	-0.562456
H	-4.871453	3.537468	0.952156
H	-3.286521	1.044912	-1.294060
C	-4.556139	-0.057639	-2.622314
C	-3.833456	-2.294754	-3.175995
H	-2.002144	-2.946276	-2.260370
H	-2.164321	0.372503	5.001974
C	-3.498635	-0.948799	3.936524

H	-4.645923	-2.199519	2.608175
H	3.478287	-4.179259	-1.763118
C	2.425394	-3.528263	-3.529388
H	1.183758	-2.705243	-5.090746
H	1.169643	-4.054797	4.694607
H	-4.395743	5.237176	-0.797086
H	-5.274108	0.752140	-2.711906
C	-4.761366	-1.256768	-3.306178
H	-3.992280	-3.236420	-3.692884
H	-4.090253	-1.193833	4.813448
H	3.070283	-4.069763	-4.214862
H	-5.640414	-1.386384	-3.930240
H	0.776058	4.685527	-0.024751
H	3.727921	-1.308593	0.250894
H	2.949455	5.804604	-0.323170
C	6.307025	-0.560543	-0.119161
H	6.255877	-1.240719	-0.977260
H	7.230470	0.018107	-0.199730
H	6.366979	-1.179297	0.782857

β-OMe-TP

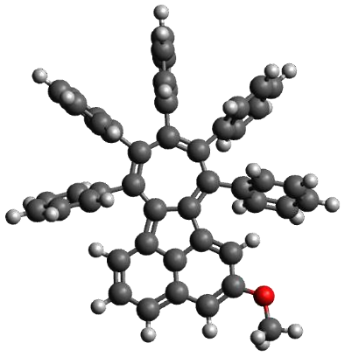

Table S81. Coordinates and energy for the optimized geometry of **β-OMe-TP**.

G = –1923.673190			
		**Coordinates / Å**	
**Atom**	**x**	**y**	**z**
C	1.742693	0.669399	0.018314
C	1.627347	-0.742436	0.004203
C	0.719728	1.645252	-0.069189
C	-0.669391	1.442354	-0.139211
C	-1.401327	0.191655	0.004262
C	-0.898417	-1.118771	-0.024437
C	0.456441	-1.535254	-0.046560
C	-2.835444	0.532513	0.096299
C	-4.004489	-0.168179	0.353846
C	-5.247918	0.523109	0.363130
C	-5.336170	1.887213	0.103159
H	-6.292219	2.396028	0.093867

C	-1.664012	2.521574	-0.292689
C	-1.624416	3.887146	-0.582128
C	-2.931876	1.927300	-0.115799
C	-2.838059	4.605729	-0.650765
C	-4.151794	2.626148	-0.145719
C	-4.072782	4.013577	-0.426647
H	-4.980291	4.608132	-0.473182
C	-1.917658	-2.216012	-0.064420
C	-2.215044	-2.960649	1.081192
C	-2.595123	-2.478974	-1.260220
C	-3.193061	-3.953450	1.032295
H	-1.690676	-2.755773	2.008748
C	-3.562758	-3.480647	-1.309703
H	-2.367329	-1.894295	-2.146319
C	-3.867791	-4.215620	-0.162188
H	-3.427105	-4.521623	1.927286
H	-4.082902	-3.681262	-2.241272
H	-4.627553	-4.990312	-0.198220
C	0.672149	-3.022396	-0.089794
C	0.485782	-3.724650	-1.284977
C	1.057495	-3.714455	1.063098
C	0.690267	-5.102672	-1.327774
H	0.186052	-3.191470	-2.181586
C	1.252184	-5.093559	1.021104
H	1.206330	-3.171903	1.990868
C	1.071730	-5.791046	-0.174810
H	0.549567	-5.637605	-2.262159
H	1.547606	-5.621753	1.922629
H	1.227732	-6.865061	-0.208029
C	2.924638	-1.500993	0.047573
C	3.644206	-1.594270	1.243325
C	3.419501	-2.119752	-1.105007
C	4.843068	-2.303305	1.286610
H	3.263648	-1.113220	2.138609
C	4.623401	-2.820136	-1.062501
H	2.862036	-2.051954	-2.033588
C	5.337187	-2.915975	0.133545
H	5.391350	-2.374479	2.221152
H	5.001657	-3.292808	-1.963925
H	6.273301	-3.465149	0.166834
C	3.143223	1.209986	0.098471
C	3.977921	1.181530	-1.023680
C	3.619906	1.747809	1.298293
C	5.274260	1.686797	-0.945963
H	3.612473	0.763241	-1.956055
C	4.919976	2.243773	1.377223
H	2.974108	1.773016	2.170223
C	5.749849	2.216501	0.255074

H	5.912928	1.663549	-1.823804
H	5.282448	2.653049	2.315391
H	6.761553	2.606022	0.315935
C	1.178673	3.071416	-0.055020
C	1.723255	3.660052	-1.200554
C	1.041774	3.825229	1.115804
C	2.117456	4.997428	-1.176738
H	1.829291	3.075800	-2.108594
C	1.448587	5.157953	1.140913
H	0.612620	3.367870	2.002056
C	1.982020	5.748184	-0.006745
H	2.531415	5.451503	-2.071801
H	1.342882	5.735997	2.053782
H	2.292746	6.788323	0.010460
H	-0.703746	4.417781	-0.767503
H	-4.041020	-1.226374	0.561069
H	-2.793405	5.665644	-0.879310
O	-6.309668	-0.276836	0.633778
C	-7.607944	0.314425	0.660669
H	-7.866489	0.740648	-0.315487
H	-8.296798	-0.495163	0.901188
H	-7.670314	1.093176	1.429523

β-OMe-DT

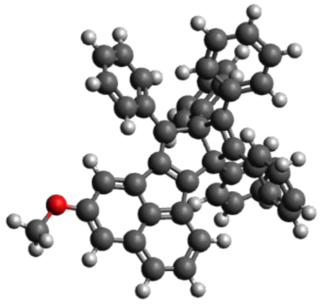

Table S82. Coordinates and energy for the optimized geometry of **β-OMe-DT**.

G = –1923.645000			
		**Coordinates / Å**	
**Atom**	**x**	**y**	**z**
C	4.538845	-1.241370	-0.121596
C	4.727850	-2.647146	-0.168097
C	3.210925	-0.817763	0.018471
C	3.648291	-3.530366	-0.062082
C	2.099031	-1.707553	0.143589
C	2.325535	-3.086918	0.103319
C	0.958039	-0.871818	0.314537
C	2.820180	0.544914	0.084553
C	1.352667	0.501059	0.249841
C	-0.502384	-1.022779	0.484122

C	3.803961	1.505450	0.040365
C	5.542916	-0.235110	-0.190781
C	0.252471	1.341563	0.319303
C	-1.015955	0.511995	0.497426
C	-1.228849	-1.116610	-0.880523
C	-0.984914	-1.918432	1.599226
C	5.171267	1.100887	-0.102775
C	0.271258	2.778881	0.112924
C	-1.714050	0.145641	-0.849383
C	-1.879145	0.887033	1.677853
C	-1.193086	-2.226459	-1.825584
C	-0.150420	-2.239632	2.675914
C	-2.310719	-2.370251	1.608225
C	1.169874	3.317806	-0.835024
C	-0.621483	3.651431	0.773840
C	-2.649780	0.895491	-1.684251
C	-1.269295	1.053897	2.930558
C	-3.268417	0.994168	1.577702
C	-0.950243	-3.537895	-1.379463
C	-1.333672	-1.997409	-3.207653
H	0.875835	-1.887180	2.693374
C	-0.629755	-3.005716	3.738924
C	-2.790265	-3.131749	2.671479
H	-2.967233	-2.123100	0.780641
H	1.816249	2.652440	-1.394417
C	1.176752	4.679184	-1.110270
C	-0.589602	5.015948	0.510982
H	-1.317663	3.261668	1.503023
C	-2.581083	2.293231	-1.814937
C	-3.685931	0.206817	-2.346844
H	-0.190156	0.967505	3.019417
C	-2.035385	1.330115	4.059247
C	-4.036251	1.273462	2.710425
H	-3.756531	0.860606	0.620039
H	-0.853005	-3.732959	-0.317450
C	-0.864411	-4.591289	-2.287768
C	-1.245110	-3.051883	-4.110594
H	-1.497392	-0.987587	-3.567905
H	0.030723	-3.247106	4.566410
C	-1.950870	-3.453489	3.740488
H	-3.820265	-3.475462	2.663345
H	1.855810	5.073349	-1.859264
C	0.303694	5.533933	-0.430988
H	-1.268290	5.677838	1.039030
H	-1.799923	2.845045	-1.311040
C	-3.505139	2.977158	-2.602336
C	-4.606975	0.893709	-3.130480
H	-3.768973	-0.868562	-2.229765

H	-1.549574	1.455751	5.022202
C	-3.424450	1.441964	3.951843
H	-5.115021	1.356377	2.617728
H	-0.683199	-5.598740	-1.925304
C	-1.012034	-4.353232	-3.655065
H	-1.350621	-2.857860	-5.173853
H	-2.324693	-4.049097	4.567855
H	0.314575	6.598963	-0.640426
H	-3.431406	4.056530	-2.695885
C	-4.519047	2.282825	-3.263809
H	-5.400424	0.346682	-3.630800
H	-4.023442	1.657977	4.831375
H	-0.943419	-5.174099	-4.362506
H	-5.240108	2.818633	-3.873687
H	6.580425	-0.523386	-0.301796
H	5.730713	-3.048223	-0.283256
H	3.601923	2.567588	0.112142
H	1.514318	-3.799178	0.196229
H	3.842423	-4.596896	-0.099999
O	6.034766	2.144009	-0.142909
C	7.427322	1.861570	-0.280910
H	7.629572	1.334480	-1.220289
H	7.926128	2.830452	-0.287918
H	7.791552	1.263899	0.562362

β-OMe-DT′

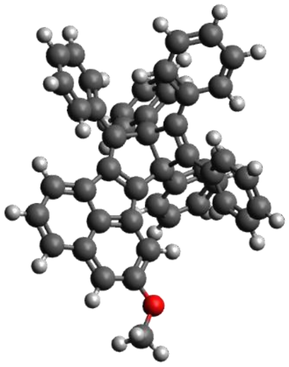

Table S83. Coordinates and energy for the optimized geometry of **β-OMe-DT′**.

G = –1923.644426			
		**Coordinates / Å**	
**Atom**	**x**	**y**	**z**
C	3.984219	2.544352	-0.206393
C	5.095432	1.663598	-0.210917
C	2.719988	1.961671	-0.048585
C	4.919317	0.279770	-0.048964
C	2.531332	0.554501	0.133839
C	3.635522	-0.288810	0.136414
C	1.126023	0.377937	0.307434

C	1.499881	2.678493	-0.016210
C	0.468067	1.639765	0.187315
C	0.174776	-0.732624	0.517011
C	1.556863	4.057126	-0.117356
C	4.009839	3.963237	-0.331241
C	-0.909936	1.490024	0.257786
C	-1.255710	0.021894	0.490210
C	-0.274890	-1.356744	-0.827549
C	0.439370	-1.667552	1.672043
C	2.826276	4.678941	-0.278235
C	-1.887222	2.533128	0.004681
C	-1.497047	-0.777150	-0.829750
C	-2.149465	-0.259166	1.674404
C	0.528898	-2.167426	-1.734873
C	1.264561	-1.281561	2.734431
C	-0.209431	-2.907487	1.730518
C	-1.606169	3.501632	-0.985338
C	-3.137688	2.576235	0.660561
C	-2.684274	-0.910211	-1.670526
C	-1.829185	0.321226	2.911200
C	-3.233520	-1.136750	1.595321
C	1.607451	-2.927657	-1.248259
C	0.285887	-2.149275	-3.121613
H	1.765038	-0.318966	2.712474
C	1.444671	-2.123025	3.832658
C	-0.032628	-3.745803	2.828805
H	-0.854367	-3.214296	0.913920
H	-0.676389	3.443324	-1.538279
C	-2.541387	4.476659	-1.307545
C	-4.056582	3.571815	0.350256
H	-3.373301	1.846648	1.422551
C	-3.603519	0.139980	-1.837377
C	-2.948247	-2.142976	-2.301224
H	-0.983403	0.998601	2.985287
C	-2.585453	0.035482	4.044101
C	-3.993084	-1.421515	2.732287
H	-3.490085	-1.600646	0.650840
H	1.799886	-2.962926	-0.181992
C	2.411146	-3.658559	-2.121093
C	1.091900	-2.879330	-3.989135
H	-0.527348	-1.548415	-3.513966
H	2.089119	-1.809486	4.648502
C	0.796337	-3.357071	3.883622
H	-0.541649	-4.704490	2.859173
H	-2.320797	5.197008	-2.088546
C	-3.765799	4.520463	-0.634295
H	-5.006174	3.604753	0.874406
H	-3.427721	1.092532	-1.357577

C	-4.737270	-0.029981	-2.629312
C	-4.081799	-2.308552	-3.089745
H	-2.263866	-2.972000	-2.155404
H	-2.324769	0.491596	4.994425
C	-3.673127	-0.837976	3.957354
H	-4.834634	-2.103545	2.655655
H	3.236166	-4.244412	-1.727134
C	2.156616	-3.638124	-3.493204
H	0.894451	-2.850747	-5.056609
H	0.935522	-4.012235	4.738280
H	-4.493501	5.287198	-0.880953
H	-5.432042	0.795696	-2.751016
C	-4.980782	-1.251093	-3.259607
H	-4.270085	-3.266457	-3.565231
H	-4.264565	-1.061313	4.840170
H	2.784199	-4.206175	-4.173230
H	-5.866956	-1.382106	-3.873210
H	4.956419	4.479147	-0.457809
H	6.090124	2.074520	-0.335733
H	0.667046	4.674335	-0.071537
H	3.555653	-1.360197	0.274538
H	2.859561	5.760346	-0.361775
O	5.931875	-0.616735	-0.042619
C	7.270036	-0.142149	-0.204997
H	7.901312	-1.029432	-0.169029
H	7.395045	0.360115	-1.170645
H	7.545944	0.539734	0.606970

β-F-TP

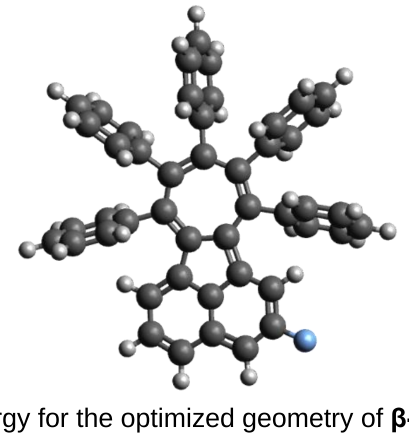

Table S84. Coordinates and energy for the optimized geometry of **β-F-TP**.

G = –1908.410652			
	**Coordinates / Å**		
**Atom**	**x**	**y**	**z**
C	-1.587327	0.575257	-0.006806
C	-1.380973	-0.826636	0.005668
C	-0.627679	1.615906	0.044502
C	0.772368	1.502908	0.099671

C	1.581765	0.301185	-0.030339
C	1.164805	-1.039017	-0.001615
C	-0.160370	-1.542359	0.019780
C	2.991751	0.731568	-0.129031
C	4.207021	0.098990	-0.376178
C	5.370786	0.890891	-0.394539
C	5.397699	2.252708	-0.167729
H	6.342109	2.785619	-0.182747
C	1.697125	2.646553	0.229794
C	1.570298	4.009704	0.495642
C	3.002055	2.132425	0.058058
C	2.732929	4.813078	0.546489
C	4.171295	2.918351	0.072186
C	4.002505	4.303650	0.327987
H	4.869098	4.956864	0.359117
C	2.252454	-2.067880	0.041085
C	2.605010	-2.790925	-1.102813
C	2.935910	-2.287888	1.242240
C	3.642889	-3.720458	-1.046043
H	2.075642	-2.619422	-2.034152
C	3.964254	-3.226911	1.299064
H	2.664847	-1.719276	2.126595
C	4.323164	-3.940889	0.153923
H	3.919001	-4.272439	-1.939109
H	4.488659	-3.395048	2.234606
H	5.129156	-4.666978	0.195935
C	-0.279538	-3.040729	0.016485
C	-0.038221	-3.776341	1.181252
C	-0.626881	-3.709392	-1.162061
C	-0.149203	-5.165428	1.167993
H	0.231704	-3.261851	2.097719
C	-0.727658	-5.099253	-1.176051
H	-0.816460	-3.140332	-2.066565
C	-0.491413	-5.830719	-0.010715
H	0.033756	-5.727137	2.079009
H	-0.993387	-5.609110	-2.097132
H	-0.573690	-6.913321	-0.020945
C	-2.627271	-1.667416	0.007669
C	-3.392044	-1.802517	-1.155588
C	-3.031849	-2.318369	1.177832
C	-4.546215	-2.583022	-1.149128
H	-3.082585	-1.297066	-2.064435
C	-4.192481	-3.089574	1.185653
H	-2.439677	-2.218013	2.081685
C	-4.951477	-3.225994	0.021997
H	-5.129786	-2.685756	-2.058993
H	-4.501353	-3.585440	2.100917
H	-5.853661	-3.830146	0.027665

C	-3.021341	1.023980	-0.046723
C	-3.816297	0.946622	1.101637
C	-3.568834	1.524830	-1.232205
C	-5.144059	1.367812	1.064043
H	-3.395031	0.557199	2.022846
C	-4.899863	1.935915	-1.270754
H	-2.953950	1.588034	-2.124257
C	-5.690287	1.860517	-0.122572
H	-5.751815	1.307725	1.961804
H	-5.317509	2.316599	-2.197914
H	-6.726284	2.184168	-0.152179
C	-1.177194	3.009085	0.012834
C	-1.745529	3.582363	1.154775
C	-1.102411	3.747995	-1.173075
C	-2.225811	4.890709	1.111826
H	-1.803283	3.009168	2.074115
C	-1.595237	5.050949	-1.216758
H	-0.654228	3.303337	-2.056343
C	-2.152873	5.626531	-0.073236
H	-2.658105	5.333755	2.003682
H	-1.537731	5.617444	-2.141067
H	-2.530739	6.643808	-0.105238
H	0.617001	4.481198	0.675871
H	4.319196	-0.957325	-0.564365
H	2.614693	5.871008	0.756108
F	6.533327	0.254461	-0.641002

β-F-DT

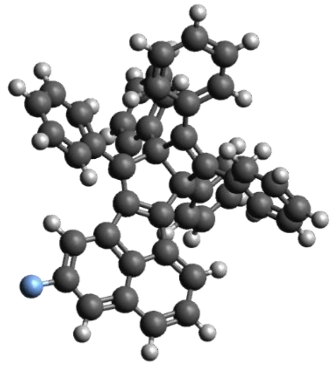

Table S85. Coordinates and energy for the optimized geometry of **β-F-DT**.

G = –1908.382896			
	**Coordinates / Å**		
**Atom**	**x**	**y**	**z**
C	4.870449	0.188024	-0.200553
C	5.448989	-1.108484	-0.244127
C	3.477952	0.229716	-0.040013
C	4.662637	-2.255104	-0.117109
C	2.664261	-0.937460	0.107850

C	3.269131	-2.195282	0.069098
C	1.334785	-0.457517	0.296241
C	2.715931	1.424665	0.027683
C	1.323942	0.968378	0.217431
C	-0.018131	-1.016320	0.495447
C	3.387759	2.629158	-0.037402
C	5.545589	1.436399	-0.288598
C	0.030563	1.463806	0.301995
C	-0.946204	0.308340	0.498481
C	-0.700859	-1.334794	-0.858134
C	-0.207548	-1.993181	1.630193
C	4.796305	2.589992	-0.197895
C	-0.361532	2.844648	0.090409
C	-1.518315	-0.257315	-0.840233
C	-1.877263	0.433642	1.680616
C	-0.361730	-2.404884	-1.789140
C	0.707503	-2.053688	2.687393
C	-1.356356	-2.793018	1.677602
C	0.332379	3.606765	-0.876692
C	-1.454931	3.434113	0.763199
C	-2.627398	0.191690	-1.678251
C	-1.330512	0.750239	2.933551
C	-3.245540	0.170176	1.581655
C	0.223422	-3.597494	-1.327377
C	-0.553255	-2.237053	-3.173838
H	1.596136	-1.430687	2.674907
C	0.482454	-2.904631	3.769962
C	-1.582779	-3.639370	2.760434
H	-2.074387	-2.749349	0.865481
H	1.130971	3.145864	-1.445547
C	-0.053455	4.910255	-1.160418
C	-1.816098	4.748548	0.492338
H	-1.998574	2.870357	1.508153
C	-2.953423	1.551568	-1.821278
C	-3.428320	-0.765936	-2.332910
H	-0.265812	0.945598	3.022447
C	-2.140332	0.810995	4.063755
C	-4.057810	0.233386	2.716101
H	-3.682773	-0.085024	0.624126
H	0.364712	-3.748630	-0.263114
C	0.593282	-4.598637	-2.223588
C	-0.180479	-3.238527	-4.064478
H	-0.981907	-1.312702	-3.545628
H	1.202172	-2.940652	4.582287
C	-0.663345	-3.699102	3.810281
H	-2.477786	-4.253783	2.782915
H	0.472242	5.473005	-1.924837
C	-1.122700	5.488494	-0.469665

H	-2.645659	5.196260	1.029701
H	-2.359016	2.305032	-1.323740
C	-4.031681	1.941828	-2.612740
C	-4.504503	-0.371811	-3.120618
H	-3.206500	-1.820313	-2.206588
H	-1.702950	1.055719	5.027004
C	-3.510046	0.553698	3.957515
H	-5.120311	0.028739	2.624648
H	1.037139	-5.516297	-1.849392
C	0.392549	-4.423938	-3.593649
H	-0.330240	-3.091768	-5.129965
H	-0.839752	-4.360621	4.653048
H	-1.418711	6.510248	-0.685591
H	-4.263571	2.997649	-2.715901
C	-4.809674	0.985008	-3.266113
H	-5.112340	-1.123758	-3.614748
H	-4.143344	0.601411	4.838229
H	0.682332	-5.203586	-4.291583
H	-5.651702	1.291640	-3.879220
H	6.620654	1.497577	-0.415359
H	6.522201	-1.210856	-0.372847
H	2.900463	3.594197	0.031722
H	2.692190	-3.105877	0.178925
H	5.144692	-3.225909	-0.153552
F	5.426160	3.778509	-0.265827

β-F-DT′

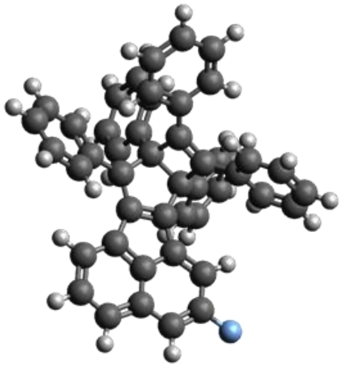

Table S86. Coordinates and energy for the optimized geometry of **β-F-DT′**.

G = –1908.382185			
	Coordinates / Å		
**Atom**	**x**	**y**	**z**
C	4.212949	2.482609	-0.264709
C	5.306166	1.583515	-0.285818
C	2.942873	1.910297	-0.089389
C	5.083294	0.218367	-0.120982

C	2.729596	0.507848	0.094478
C	3.822069	-0.358437	0.081315
C	1.322936	0.354510	0.286518
C	1.732986	2.646961	-0.039981
C	0.686432	1.626039	0.174736
C	0.357368	-0.740601	0.509782
C	1.810558	4.023653	-0.136962
C	4.258041	3.901925	-0.387045
C	-0.694698	1.499140	0.259347
C	-1.060145	0.037380	0.496333
C	-0.116654	-1.360839	-0.828485
C	0.619740	-1.676147	1.664714
C	3.086071	4.631563	-0.314405
C	-1.657473	2.555855	0.015469
C	-1.327606	-0.758847	-0.821220
C	-1.947002	-0.229176	1.689071
C	0.660664	-2.191901	-1.740388
C	1.464355	-1.301377	2.715721
C	-0.051087	-2.903679	1.735215
C	-1.368003	3.524940	-0.972247
C	-2.905252	2.611742	0.676490
C	-2.525111	-0.874178	-1.649989
C	-1.602882	0.344065	2.922755
C	-3.047718	-1.086502	1.620027
C	1.725594	-2.976225	-1.262020
C	0.403159	-2.172797	-3.124451
H	1.981178	-0.347602	2.685104
C	1.642056	-2.142126	3.814908
C	0.123288	-3.741032	2.834544
H	-0.711236	-3.201330	0.927455
H	-0.442617	3.455972	-1.531112
C	-2.292181	4.512330	-1.287133
C	-3.812120	3.620331	0.374213
H	-3.146965	1.882225	1.436517
C	-3.420285	0.195332	-1.824904
C	-2.822326	-2.108875	-2.261859
H	-0.743586	1.005124	2.988480
C	-2.352283	0.070796	4.063225
C	-3.800182	-1.358697	2.764737
H	-3.322733	-1.544328	0.677795
H	1.927310	-3.014054	-0.197440
C	2.501865	-3.729590	-2.140661
C	1.182104	-2.925090	-3.997659
H	-0.400076	-1.554471	-3.510207
H	2.301537	-1.837544	4.622041
C	0.971757	-3.363787	3.878006
H	-0.402839	-4.690045	2.874605
H	-2.066363	5.232332	-2.066868

C	-3.512985	4.569009	-0.607964
H	-4.759035	3.663562	0.902305
H	-3.217711	1.150502	-1.361238
C	-4.563954	0.041107	-2.605689
C	-3.965599	-2.258500	-3.039391
H	-2.155911	-2.951370	-2.110033
H	-2.073300	0.520829	5.011217
C	-3.456561	-0.782570	3.986918
H	-4.654824	-2.025098	2.696328
H	3.316014	-4.334728	-1.753285
C	2.233397	-3.707599	-3.510081
H	0.973949	-2.895704	-5.063046
H	1.108889	-4.018292	4.733466
H	-4.231802	5.345956	-0.848520
H	-5.239925	0.881218	-2.734097
C	-4.841009	-1.182651	-3.216702
H	-4.179826	-3.217969	-3.500473
H	-4.042631	-0.996200	4.875672
H	2.839661	-4.293237	-4.194466
H	-5.734874	-1.301218	-3.821594
H	5.209934	4.403874	-0.526411
H	6.324001	1.933884	-0.423060
H	0.930511	4.653445	-0.075930
H	3.733675	-1.429078	0.217407
H	3.130545	5.712591	-0.394909
F	6.147980	-0.601978	-0.145115

γ-Cl-TP

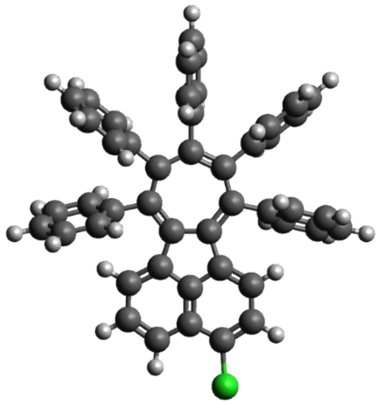

Table S87. Coordinates and energy for the optimized geometry of **γ-Cl-TP**.

G = –2268.780308			
		Coordinates / Å	
**Atom**	**x**	**y**	**z**
C	1.444013	-1.102261	-0.018520
C	1.818801	0.262688	-0.001148
C	0.146761	-1.667132	0.071317

C	-1.086598	-0.997464	0.125343
C	-1.345789	0.421857	-0.059537
C	-0.424704	1.482626	-0.032778
C	0.988152	1.409616	0.032401
C	-2.804896	0.588443	-0.183200
C	-3.665575	1.639923	-0.495685
C	-5.056591	1.413024	-0.520004
C	-5.598350	0.171555	-0.225011
C	-2.393765	-1.665599	0.283150
C	-2.820984	-2.950723	0.612371
C	-3.381939	-0.678626	0.059690
C	-4.206346	-3.221311	0.673159
C	-4.766612	-0.939606	0.081087
C	-5.166267	-2.260174	0.401298
H	-6.219892	-2.512343	0.440288
C	-1.006690	2.862786	-0.041756
C	-0.989738	3.636851	-1.206282
C	-1.592989	3.370588	1.122983
C	-1.568059	4.905918	-1.207563
H	-0.534433	3.242934	-2.109096
C	-2.158044	4.644517	1.122551
H	-1.610105	2.765098	2.023933
C	-2.152014	5.412101	-0.044103
H	-1.560501	5.498831	-2.116927
H	-2.607965	5.034050	2.030508
H	-2.598794	6.401537	-0.046616
C	1.698066	2.732433	0.104124
C	1.724293	3.443912	1.308018
C	2.334003	3.261529	-1.023766
C	2.388283	4.667074	1.385134
H	1.230430	3.036801	2.184725
C	2.988662	4.489060	-0.947585
H	2.317359	2.710678	-1.958462
C	3.020373	5.193632	0.257494
H	2.409339	5.208106	2.326322
H	3.476355	4.892912	-1.829653
H	3.534886	6.147921	0.317097
C	3.297742	0.531929	-0.023134
C	4.024011	0.370901	-1.207670
C	3.956714	0.946329	1.138959
C	5.393743	0.626146	-1.230011
H	3.516364	0.047712	-2.110686
C	5.328254	1.191385	1.117269
H	3.395639	1.075359	2.058658
C	6.050037	1.034441	-0.067444
H	5.947738	0.503557	-2.155772
H	5.831573	1.507304	2.026029
H	7.118030	1.229435	-0.084663

C	2.572470	-2.091837	-0.099921
C	3.372432	-2.350187	1.018164
C	2.826834	-2.764063	-1.299800
C	4.414673	-3.271306	0.936252
H	3.178816	-1.829626	1.950374
C	3.876121	-3.677685	-1.382875
H	2.206177	-2.568014	-2.168346
C	4.671376	-3.935149	-0.264941
H	5.027526	-3.467588	1.810809
H	4.069669	-4.189099	-2.320953
H	5.486604	-4.649470	-0.329039
C	0.086134	-3.163854	0.070713
C	0.404990	-3.896394	1.218485
C	-0.315802	-3.831903	-1.091359
C	0.310039	-5.287640	1.205633
H	0.715498	-3.379088	2.120166
C	-0.396647	-5.223066	-1.105528
H	-0.568415	-3.260304	-1.979152
C	-0.089460	-5.953242	0.044500
H	0.549637	-5.850592	2.102426
H	-0.705305	-5.735087	-2.011695
H	-0.159115	-7.036620	0.035903
H	-2.133203	-3.751508	0.835840
H	-3.314277	2.631365	-0.736209
H	-4.522709	-4.225863	0.933173
H	-5.718626	2.234942	-0.766037
Cl	-7.332529	-0.023858	-0.243280

γ-Cl-DT

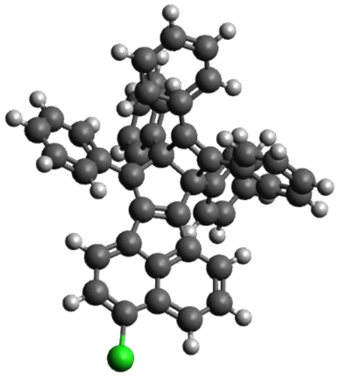

Table S88. Coordinates and energy for the optimized geometry of **γ-Cl-DT**.

G = –2268.751318			
	**Coordinates / Å**		
**Atom**	**x**	**y**	**z**
C	4.655970	0.143283	-0.064363
C	5.217363	-1.157920	-0.101573
C	3.260238	0.214341	0.063039
H	6.290609	-1.274626	-0.205494

C	4.411227	-2.292907	-0.000562
C	2.428768	-0.945392	0.183593
C	3.015692	-2.211306	0.151911
C	1.100206	-0.451209	0.340728
C	2.509726	1.415652	0.118953
C	1.109414	0.974258	0.270244
C	-0.263539	-0.996544	0.500813
C	3.203610	2.610734	0.079372
C	5.324726	1.402675	-0.126537
C	-0.181518	1.483450	0.323561
C	-1.175851	0.338426	0.487921
C	-0.915750	-1.300606	-0.870812
C	-0.493680	-1.976125	1.625640
C	4.617139	2.588528	-0.047345
C	-0.553769	2.868972	0.109289
C	-1.720527	-0.213697	-0.868264
C	-2.136002	0.467935	1.646358
C	-0.566432	-2.370924	-1.797738
C	0.389129	-2.045656	2.709306
C	-1.649018	-2.767799	1.636911
C	0.174827	3.629763	-0.833201
C	-1.660748	3.465160	0.753607
C	-2.803011	0.251850	-1.731706
C	-1.617909	0.766383	2.915833
C	-3.505099	0.226764	1.509164
C	-0.002537	-3.570159	-1.326705
C	-0.726254	-2.197881	-3.185807
H	1.281203	-1.427741	2.724845
C	0.126328	-2.897425	3.782623
C	-1.913238	-3.615094	2.710457
H	-2.342449	-2.717132	0.804072
H	0.985289	3.164134	-1.380896
C	-0.191321	4.937905	-1.121398
C	-2.001946	4.784215	0.479680
H	-2.230658	2.902660	1.479602
C	-3.105547	1.616290	-1.882438
C	-3.602268	-0.694247	-2.404879
H	-0.552925	0.943143	3.034703
C	-2.456454	0.831481	4.024636
C	-4.346194	0.294550	2.622149
H	-3.920666	-0.014238	0.538459
H	0.113883	-3.725271	-0.260008
C	0.377365	-4.572567	-2.217251
C	-0.343522	-3.200648	-4.070767
H	-1.138022	-1.268675	-3.564491
H	0.821327	-2.940329	4.615881
C	-1.025723	-3.683858	3.786885
H	-2.812824	-4.223174	2.704985

H	0.360720	5.499577	-1.867829
C	-1.274645	5.522463	-0.458341
H	-2.842424	5.236876	0.995495
H	-2.511760	2.361143	-1.371426
C	-4.159015	2.021933	-2.699170
C	-4.653636	-0.284761	-3.217881
H	-3.398891	-1.751696	-2.273085
H	-2.040979	1.061786	5.001047
C	-3.826751	0.596989	3.880111
H	-5.408949	0.107443	2.500938
H	0.804366	-5.495274	-1.835917
C	0.208048	-4.392591	-3.590861
H	-0.468632	-3.049868	-5.138873
H	-1.231704	-4.346098	4.622340
H	-1.555127	6.547933	-0.677348
H	-4.372881	3.080990	-2.807989
C	-4.935340	1.076323	-3.370612
H	-5.260634	-1.027930	-3.726126
H	-4.482439	0.648324	4.744079
H	0.505602	-5.173184	-4.284449
H	-5.758072	1.395016	-4.003449
H	2.704482	3.570278	0.147431
H	5.155173	3.528556	-0.084043
H	4.880039	-3.270146	-0.031592
H	2.421596	-3.113100	0.241433
Cl	7.062973	1.448505	-0.293515

γ-Cl-DT′

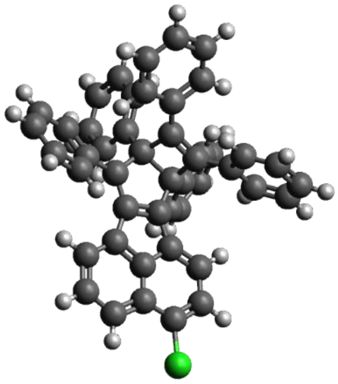

Table S89. Coordinates and energy for the optimized geometry of **γ-Cl-DT′**.

G = –2268.752017			
		**Coordinates / Å**	
**Atom**	**x**	**y**	**z**
C	4.424836	1.488375	-0.085998
C	5.293509	0.360602	-0.092810
C	3.063082	1.176975	0.045848

C	4.818903	-0.948856	0.041292
C	2.555383	-0.152622	0.198985
C	3.453320	-1.222523	0.197862
C	1.146882	-0.016047	0.346964
C	2.028004	2.147177	0.074087
C	0.787623	1.362327	0.239842
C	-0.031637	-0.889851	0.520686
C	2.384505	3.480636	0.003445
C	4.761033	2.867170	-0.180941
C	-0.589450	1.523186	0.283723
C	-1.257742	0.165115	0.482161
C	-0.578135	-1.377943	-0.843524
C	-0.006654	-1.875572	1.663441
C	3.759397	3.819193	-0.126694
C	-1.303976	2.762516	0.036727
C	-1.641026	-0.541019	-0.857039
C	-2.216655	0.074158	1.644914
C	0.048044	-2.331045	-1.752617
C	0.860095	-1.697657	2.747823
C	-0.917447	-2.939439	1.687996
C	-0.799515	3.654693	-0.936176
C	-2.520090	3.078283	0.682128
C	-2.806892	-0.392368	-1.723990
C	-1.800410	0.548735	2.898321
C	-3.467995	-0.536092	1.529732
C	0.904545	-3.332338	-1.260645
C	-0.135877	-2.222629	-3.144257
H	1.562265	-0.870105	2.753292
C	0.823585	-2.572492	3.834119
C	-0.956662	-3.810187	2.774526
H	-1.596914	-3.083292	0.854553
H	0.098894	3.394290	-1.482993
C	-1.486947	4.819460	-1.252311
C	-3.186541	4.259737	0.379014
H	-2.922968	2.411517	1.431419
C	-3.478028	0.834236	-1.868353
C	-3.308805	-1.519225	-2.406446
H	-0.826252	1.018254	3.000935
C	-2.625485	0.422589	4.012096
C	-4.295853	-0.660798	2.647640
H	-3.801854	-0.915991	0.571962
H	1.046458	-3.438289	-0.191028
C	1.546863	-4.208174	-2.133890
C	0.509195	-3.097895	-4.012051
H	-0.774425	-1.440033	-3.539452
H	1.503159	-2.421314	4.667477
C	-0.084973	-3.630792	3.851096
H	-1.667860	-4.630698	2.778724

H	-1.100494	5.480830	-2.020884
C	-2.677589	5.130351	-0.588814
H	-4.109993	4.499916	0.895801
H	-3.119424	1.710635	-1.346964
C	-4.599020	0.936275	-2.689451
C	-4.428769	-1.413025	-3.223934
H	-2.820687	-2.479576	-2.278914
H	-2.289533	0.794004	4.975561
C	-3.879225	-0.182887	3.889295
H	-5.266995	-1.135289	2.543135
H	2.198980	-4.979975	-1.736154
C	1.351714	-4.095156	-3.511229
H	0.360898	-2.997757	-5.083138
H	-0.114602	-4.311581	4.696452
H	-3.210429	6.044695	-0.830317
H	-5.099584	1.894378	-2.792776
C	-5.077567	-0.183154	-3.371712
H	-4.802504	-2.292438	-3.739731
H	-4.524138	-0.281555	4.757246
H	1.853723	-4.776545	-4.191389
H	-5.953295	-0.101801	-4.008451
H	1.646827	4.273477	0.048918
H	4.026523	4.868845	-0.186451
H	5.531408	-1.764972	0.031856
H	3.119627	-2.246827	0.313700
H	5.797042	3.167760	-0.285697
Cl	7.005107	0.602200	-0.265410

γ-F-TP

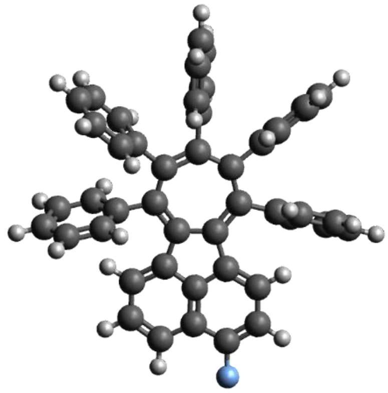

Table S90. Coordinates and energy for the optimized geometry of **γ-F-TP**.

G = –1908.413364			
	Coordinates / Å		
**Atom**	**x**	**y**	**z**
C	-1.254843	-1.097557	0.016775
C	-1.623090	0.267789	0.000835
C	0.041391	-1.667724	-0.064736
C	1.277840	-1.005359	-0.109202

C	1.544420	0.416000	0.071080
C	0.625057	1.479584	0.038534
C	-0.786674	1.411846	-0.029210
C	3.001119	0.578299	0.198716
C	3.871706	1.629036	0.502498
C	5.260450	1.394675	0.534238
C	5.778499	0.143100	0.255644
C	2.582402	-1.681368	-0.251859
C	3.014654	-2.970936	-0.563027
C	3.569933	-0.695297	-0.030578
C	4.400191	-3.246843	-0.609421
C	4.950526	-0.964564	-0.041393
C	5.361284	-2.284536	-0.341581
H	6.416699	-2.532091	-0.368087
C	1.212416	2.857684	0.046867
C	1.201483	3.631058	1.211849
C	1.798273	3.364041	-1.118704
C	1.784673	4.897953	1.212752
H	0.746472	3.238376	2.115374
C	2.368626	4.635642	-1.118692
H	1.810984	2.759060	-2.020091
C	2.368191	5.402590	0.048431
H	1.781305	5.490335	2.122517
H	2.818193	5.023952	-2.027380
H	2.818933	6.390250	0.050593
C	-1.491979	2.737385	-0.101183
C	-1.517750	3.448385	-1.305326
C	-2.124448	3.269409	1.027258
C	-2.177427	4.673929	-1.382124
H	-1.026535	3.039089	-2.182521
C	-2.775191	4.499086	0.951430
H	-2.108306	2.719005	1.962261
C	-2.806176	5.203241	-0.253904
H	-2.197839	5.214639	-2.323535
H	-3.260327	4.904989	1.833995
H	-3.317497	6.159279	-0.313248
C	-3.100934	0.543969	0.019137
C	-3.830384	0.388349	1.202407
C	-3.755874	0.959735	-1.144734
C	-5.198899	0.650547	1.221929
H	-3.325961	0.063873	2.106783
C	-5.126155	1.212028	-1.125958
H	-3.192440	1.084464	-2.063596
C	-5.850961	1.060663	0.057653
H	-5.755226	0.531986	2.146840
H	-5.626179	1.529215	-2.036120
H	-6.917959	1.261307	0.072634
C	-2.387938	-2.082912	0.087665

C	-3.175857	-2.340431	-1.039052
C	-2.659170	-2.751743	1.285713
C	-4.222642	-3.257390	-0.967667
H	-2.969389	-1.822111	-1.969796
C	-3.712458	-3.661671	1.358176
H	-2.048272	-2.555774	2.161229
C	-4.495625	-3.918299	0.231520
H	-4.826256	-3.452757	-1.848862
H	-3.918854	-4.170667	2.294858
H	-5.314131	-4.629582	0.287402
C	0.094936	-3.164843	-0.066276
C	-0.206976	-3.893145	-1.221186
C	0.473524	-3.837426	1.100937
C	-0.118452	-5.284900	-1.210129
H	-0.499593	-3.372111	-2.126745
C	0.548379	-5.228963	1.113076
H	0.712959	-3.268664	1.994183
C	0.258076	-5.955040	-0.043951
H	-0.344999	-5.844720	-2.112285
H	0.839237	-5.744540	2.023132
H	0.322946	-7.038742	-0.036882
H	2.328196	-3.773873	-0.783284
H	3.524379	2.624703	0.730773
H	4.714075	-4.255700	-0.855468
H	5.942775	2.202780	0.771304
F	7.104859	-0.040713	0.273629

γ-F-DT

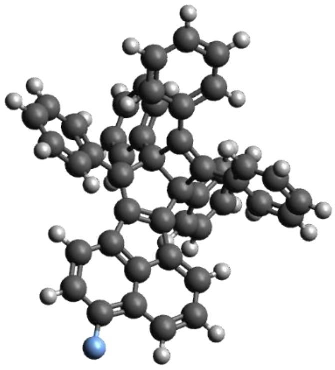

Table S91. Coordinates and energy for the optimized geometry of **γ-F-DT**.

G = –1908.383263			
		**Coordinates / Å**	
**Atom**	**x**	**y**	**z**
C	4.792621	0.742125	-0.201611
C	5.513174	-0.475478	-0.240268
C	3.402232	0.653772	-0.045568
H	6.590585	-0.457981	-0.364996
C	4.848960	-1.697435	-0.112265

C	2.717792	-0.594371	0.102820
C	3.456442	-1.779721	0.068566
C	1.344707	-0.256533	0.288301
C	2.522795	1.762055	0.018785
C	1.187902	1.161119	0.208028
C	0.056970	-0.953579	0.491191
C	3.077694	3.030008	-0.042905
C	5.302577	2.065582	-0.285194
C	-0.152613	1.517210	0.290958
C	-1.001774	0.269049	0.502477
C	-0.608982	-1.345635	-0.851176
C	-0.023556	-1.943543	1.628434
C	4.480729	3.170195	-0.200735
C	-0.693333	2.844177	0.068370
C	-1.531343	-0.357780	-0.825832
C	-1.924344	0.304641	1.697455
C	-0.186222	-2.386661	-1.780575
C	0.894906	-1.904740	2.683836
C	-1.078465	-2.863725	1.678798
C	-0.070750	3.681786	-0.885288
C	-1.863883	3.303147	0.713318
C	-2.692534	-0.032226	-1.652582
C	-1.398707	0.690782	2.939770
C	-3.257882	-0.103977	1.619223
C	0.555395	-3.492245	-1.327326
C	-0.459478	-2.273855	-3.157170
H	1.711004	-1.189618	2.669777
C	0.765956	-2.774942	3.766810
C	-1.209585	-3.729348	2.762172
H	-1.798094	-2.899663	0.867797
H	0.788127	3.316019	-1.434647
C	-0.598200	4.931657	-1.181293
C	-2.368680	4.566554	0.430650
H	-2.357114	2.679505	1.445328
C	-3.110435	1.291269	-1.873602
C	-3.444568	-1.080846	-2.219311
H	-0.360656	1.002098	3.011229
C	-2.195644	0.674277	4.080639
C	-4.057456	-0.117996	2.764265
H	-3.677680	-0.410880	0.669110
H	0.759623	-3.603031	-0.268366
C	1.000572	-4.462691	-2.222984
C	-0.011216	-3.244138	-4.047454
H	-1.011971	-1.415704	-3.523825
H	1.487392	-2.732793	4.577320
C	-0.286903	-3.688913	3.810000
H	-2.032364	-4.437537	2.786207
H	-0.123974	5.551631	-1.935030

C	-1.742945	5.382105	-0.516563
H	-3.257635	4.914708	0.946327
H	-2.549280	2.112893	-1.451017
C	-4.235246	1.557254	-2.652154
C	-4.567310	-0.810656	-2.994612
H	-3.146166	-2.107568	-2.034465
H	-1.774852	0.973960	5.035730
C	-3.530889	0.269944	3.995582
H	-5.093142	-0.435619	2.689113
H	1.566118	-5.313488	-1.854952
C	0.719273	-4.343074	-3.584746
H	-0.225815	-3.140069	-5.106873
H	-0.388583	-4.365538	4.653118
H	-2.150428	6.362608	-0.741957
H	-4.539162	2.586680	-2.816956
C	-4.967040	0.510868	-3.215340
H	-5.136740	-1.631410	-3.420276
H	-4.154101	0.257320	4.884657
H	1.067625	-5.098517	-4.282544
H	-5.845322	0.721165	-3.818143
H	2.472231	3.925713	0.032121
H	4.924930	4.157276	-0.256437
H	5.428629	-2.613285	-0.143609
H	2.978682	-2.746073	0.179313
F	6.624870	2.227155	-0.439603

γ-F-DT′

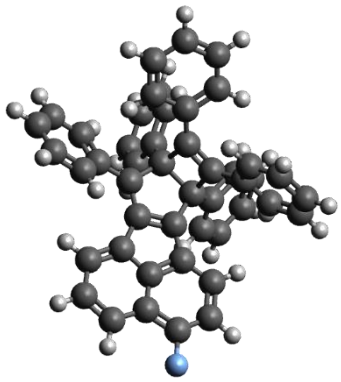

Table S92. Coordinates and energy for the optimized geometry of **γ-F-DT′**.

G = –1908.386378			
		Coordinates / Å	
Atom	x	y	z
C	4.517206	1.734569	-0.156472
C	5.432385	0.653433	-0.170754
C	3.178363	1.353089	-0.004368
C	5.046484	-0.680915	-0.029405
C	2.744049	-0.002644	0.157862
C	3.701030	-1.025431	0.145044

C	1.335916	0.063526	0.324818
C	2.099289	2.271081	0.035771
C	0.903161	1.424401	0.221027
C	0.203649	-0.867262	0.515003
C	2.394503	3.620202	-0.047063
C	4.796606	3.123531	-0.261753
C	-0.477598	1.514584	0.281367
C	-1.074959	0.124220	0.486598
C	-0.331638	-1.386924	-0.841190
C	0.291564	-1.843405	1.662638
C	3.749437	4.025877	-0.197868
C	-1.256663	2.717757	0.042599
C	-1.434402	-0.602767	-0.847114
C	-2.018077	-0.012629	1.657787
C	0.332812	-2.310183	-1.754090
C	1.162114	-1.615932	2.734687
C	-0.566290	-2.949799	1.705584
C	-0.813344	3.627710	-0.942865
C	-2.473215	2.978994	0.710052
C	-2.612074	-0.509976	-1.705918
C	-1.606295	0.466766	2.910671
C	-3.247977	-0.666294	1.550366
C	1.238861	-3.269425	-1.267010
C	0.135707	-2.211630	-3.144635
H	1.822632	-0.754697	2.725798
C	1.181165	-2.483634	3.827187
C	-0.550031	-3.813533	2.798276
H	-1.248667	-3.131557	0.881912
H	0.086316	3.407391	-1.505189
C	-1.560661	4.757663	-1.250334
C	-3.200820	4.126161	0.414457
H	-2.830047	2.297807	1.469795
C	-3.345650	0.681821	-1.838520
C	-3.061165	-1.656923	-2.391585
H	-0.646898	0.966603	3.006679
C	-2.414776	0.303411	4.031678
C	-4.059788	-0.828005	2.675354
H	-3.577581	-1.051072	0.593110
H	1.392697	-3.367553	-0.198242
C	1.916171	-4.114873	-2.143802
C	0.815673	-3.056433	-4.016021
H	-0.541075	-1.459960	-3.536259
H	1.862659	-2.293851	4.651050
C	0.325024	-3.584348	3.862648
H	-1.220566	-4.667422	2.817009
H	-1.219822	5.433033	-2.028336
C	-2.752168	5.015146	-0.566180
H	-4.124878	4.324808	0.947616

H	-3.028819	1.572143	-1.313532
C	-4.475695	0.731716	-2.652002
C	-4.190433	-1.602869	-3.201407
H	-2.524667	-2.592214	-2.272697
H	-2.081943	0.678584	4.994786
C	-3.647840	-0.344633	3.916477
H	-5.014600	-1.335795	2.576861
H	2.606514	-4.854661	-1.749695
C	1.707195	-4.012600	-3.519961
H	0.656164	-2.964263	-5.086225
H	0.338567	-4.259537	4.712914
H	-3.332201	5.901983	-0.801430
H	-5.024834	1.663798	-2.746327
C	-4.901370	-0.406566	-3.338132
H	-4.522776	-2.497246	-3.719824
H	-4.280126	-0.472327	4.789934
H	2.236260	-4.670411	-4.202836
H	-5.784194	-0.366103	-3.968983
H	1.620492	4.377410	0.004184
H	3.962349	5.087404	-0.265646
H	5.816989	-1.442643	-0.049078
H	3.421401	-2.065337	0.265010
H	5.817971	3.466539	-0.381376
F	6.727787	0.925889	-0.320888

γ-NO_2-TP

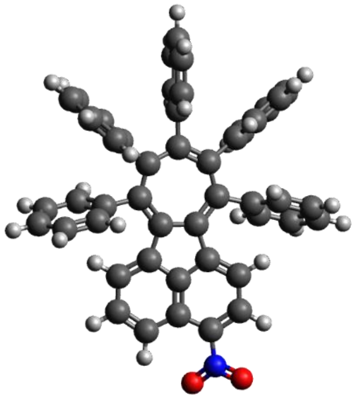

Table S93. Coordinates and energy for the optimized geometry of **γ-NO_2-TP**.

G = –2013.671259			
		**Coordinates / Å**	
**Atom**	**x**	**y**	**z**
C	-1.597170	-1.088843	0.025503
C	-1.955847	0.284163	-0.003690
C	-0.309973	-1.671117	-0.064847
C	0.931742	-1.012693	-0.133704
C	1.205139	0.396990	0.054352

C	0.304960	1.471998	0.031938
C	-1.112433	1.418520	-0.036643
C	2.675121	0.542267	0.178602
C	3.532124	1.579249	0.527707
C	4.918988	1.333927	0.550247
C	5.450453	0.103950	0.203933
C	2.229844	-1.694746	-0.313989
C	2.625455	-2.982472	-0.660330
C	3.238521	-0.726901	-0.093081
C	4.005651	-3.274045	-0.735773
C	4.622390	-1.004730	-0.135536
C	4.988904	-2.337187	-0.469495
H	6.031405	-2.614841	-0.521937
C	0.905524	2.843382	0.040612
C	0.878983	3.627415	1.198603
C	1.517708	3.332852	-1.118770
C	1.475690	4.887717	1.198921
H	0.403331	3.247892	2.096951
C	2.099845	4.599019	-1.119296
H	1.540407	2.720329	-2.014780
C	2.085577	5.375918	0.040958
H	1.462009	5.488103	2.103194
H	2.569559	4.974842	-2.022900
H	2.546224	6.358894	0.042953
C	-1.802266	2.750873	-0.111334
C	-1.802300	3.467410	-1.312683
C	-2.444501	3.284081	1.011170
C	-2.447907	4.700041	-1.392663
H	-1.303564	3.057184	-2.185077
C	-3.079234	4.521755	0.932067
H	-2.447582	2.729652	1.943806
C	-3.085737	5.231308	-0.270453
H	-2.449830	5.244905	-2.331793
H	-3.571308	4.929426	1.809852
H	-3.585239	6.193332	-0.332342
C	-3.431178	0.570557	0.006071
C	-4.170044	0.416894	1.183875
C	-4.073494	0.993575	-1.162229
C	-5.536687	0.688014	1.193029
H	-3.674931	0.087536	2.091494
C	-5.442453	1.253362	-1.153478
H	-3.502304	1.117198	-2.076390
C	-6.177097	1.103799	0.024186
H	-6.100825	0.571706	2.113377
H	-5.933462	1.575185	-2.066816
H	-7.242942	1.310565	0.031236
C	-2.737826	-2.061972	0.119378
C	-3.552750	-2.311018	-0.990163

C	-2.986830	-2.729305	1.323162
C	-4.605273	-3.218811	-0.895342
H	-3.362160	-1.795211	-1.925477
C	-4.047598	-3.628159	1.419009
H	-2.353837	-2.540822	2.184302
C	-4.857887	-3.876840	0.309973
H	-5.229398	-3.409150	-1.763122
H	-4.237870	-4.135302	2.359985
H	-5.681684	-4.580194	0.384030
C	-0.264830	-3.167776	-0.048099
C	-0.620778	-3.911311	-1.178024
C	0.160070	-3.824877	1.112245
C	-0.539564	-5.303002	-1.149299
H	-0.949414	-3.402571	-2.077980
C	0.226255	-5.216531	1.142413
H	0.440685	-3.245354	1.986410
C	-0.117691	-5.957816	0.010112
H	-0.807622	-5.874711	-2.032309
H	0.551901	-5.720220	2.047226
H	-0.059214	-7.041630	0.031136
H	1.920634	-3.768913	-0.881191
H	3.185976	2.565440	0.793853
H	4.302796	-4.282592	-1.002901
H	5.595902	2.132143	0.825249
N	6.915200	-0.000557	0.205993
O	7.436732	-0.865711	-0.499325
O	7.545564	0.795433	0.900969

γ-NO_2-DT

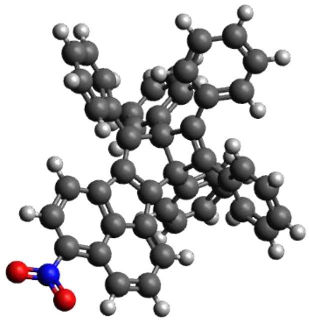

Table S94. Coordinates and energy for the optimized geometry of **γ-NO_2-DT**.

G = –2013.644489			
		**Coordinates / Å**	
**Atom**	**x**	**y**	**z**
C	4.526237	-0.144583	-0.020681
C	5.003532	-1.487400	-0.036000
C	3.135219	0.004543	0.098622

H	6.063771	-1.674979	-0.124708
C	4.126048	-2.564792	0.067915
C	2.233908	-1.100915	0.218841
C	2.735646	-2.399781	0.202892
C	0.932087	-0.531632	0.364047
C	2.443950	1.247875	0.152441
C	1.018889	0.887296	0.286340
C	-0.458798	-1.001633	0.515263
C	3.187133	2.410435	0.138523
C	5.243661	1.093731	-0.063753
C	-0.241822	1.470889	0.324159
C	-1.297286	0.380487	0.480706
C	-1.102076	-1.277449	-0.867705
C	-0.755981	-1.963707	1.638964
C	4.595250	2.311623	0.031622
C	-0.535661	2.871960	0.100994
C	-1.848275	-0.149258	-0.882516
C	-2.266454	0.568044	1.623222
C	-0.793134	-2.369174	-1.783571
C	0.111963	-2.085584	2.729838
C	-1.957147	-2.684030	1.639660
C	0.247594	3.591343	-0.830950
C	-1.621463	3.526793	0.725384
C	-2.892661	0.365611	-1.763617
C	-1.750160	0.843805	2.898505
C	-3.644482	0.402103	1.466567
C	-0.312777	-3.598165	-1.296801
C	-0.908057	-2.190186	-3.175303
H	1.039550	-1.522629	2.754047
C	-0.210991	-2.919568	3.800712
C	-2.281269	-3.512888	2.711068
H	-2.638760	-2.592418	0.800628
H	1.040240	3.082966	-1.366535
C	-0.044972	4.915597	-1.127710
C	-1.888076	4.860946	0.443505
H	-2.231604	2.997123	1.443130
C	-3.128521	1.742747	-1.919134
C	-3.722825	-0.543063	-2.451141
H	-0.678689	0.961443	3.031706
C	-2.599447	0.961060	3.994686
C	-4.496054	0.521931	2.567112
H	-4.058920	0.179766	0.491009
H	-0.233150	-3.757327	-0.227301
C	0.030272	-4.623116	-2.176526
C	-0.562698	-3.215961	-4.049154
H	-1.255277	-1.239461	-3.565198
H	0.472872	-3.003913	4.639929
C	-1.408611	-3.634582	3.794968

H	-3.215902	-4.065352	2.698006
H	0.547511	5.445493	-1.866185
C	-1.107213	5.557471	-0.483481
H	-2.711957	5.358703	0.944192
H	-2.510198	2.459448	-1.397147
C	-4.146652	2.196442	-2.754904
C	-4.738807	-0.085380	-3.282955
H	-3.571839	-1.608765	-2.315806
H	-2.185903	1.173231	4.975953
C	-3.978403	0.801777	3.831036
H	-5.565678	0.393199	2.431557
H	0.392832	-5.568201	-1.783575
C	-0.093572	-4.436669	-3.554115
H	-0.652055	-3.061036	-5.120208
H	-1.661431	-4.282599	4.628661
H	-1.329889	6.595597	-0.708904
H	-4.308956	3.264180	-2.867477
C	-4.953621	1.287352	-3.440613
H	-5.370583	-0.799641	-3.802209
H	-4.642457	0.893684	4.685148
H	0.175447	-5.234919	-4.239150
H	-5.748818	1.643582	-4.088400
H	2.733454	3.391173	0.209952
H	5.192330	3.213900	0.013229
H	4.536729	-3.568231	0.054495
H	2.085036	-3.261145	0.295426
N	6.702515	1.134375	-0.212699
O	7.269609	0.127159	-0.640963
O	7.284660	2.176894	0.083819

γ-NO_2-DT′

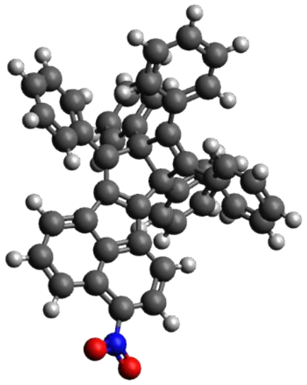

Table S95. Coordinates and energy for the optimized geometry of **γ-NO_2-DT′**.

G = –2013.640304			
		**Coordinates / Å**	
**Atom**	**x**	**y**	**z**
C	4.347696	1.415828	-0.089396

C	5.171352	0.254310	-0.128813
C	2.976536	1.129837	0.043537
C	4.671264	-1.038944	-0.015279
C	2.439332	-0.189739	0.161390
C	3.295488	-1.284169	0.133159
C	1.022609	-0.023228	0.328684
C	1.958950	2.118262	0.117420
C	0.700685	1.355650	0.262717
C	-0.165116	-0.882081	0.509230
C	2.339169	3.444672	0.114820
C	4.706162	2.797161	-0.113933
C	-0.683471	1.543395	0.311734
C	-1.367043	0.196758	0.497462
C	-0.747723	-1.355423	-0.846820
C	-0.136160	-1.871639	1.648822
C	3.721589	3.759355	-0.001770
C	-1.386452	2.782278	0.073556
C	-1.778192	-0.482463	-0.850524
C	-2.319621	0.100238	1.665698
C	-0.189848	-2.345868	-1.759614
C	0.741677	-1.701598	2.725575
C	-1.055345	-2.927920	1.679503
C	-0.829113	3.727856	-0.820901
C	-2.658408	3.047046	0.634897
C	-2.947558	-0.298303	-1.709404
C	-1.890304	0.556586	2.921289
C	-3.577653	-0.495937	1.549999
C	0.682016	-3.343341	-1.288113
C	-0.471004	-2.288159	-3.138002
H	1.449246	-0.878504	2.727301
C	0.708259	-2.577748	3.810675
C	-1.091661	-3.799613	2.765348
H	-1.743541	-3.065678	0.852231
H	0.107979	3.506477	-1.316003
C	-1.515290	4.891328	-1.137524
C	-3.323193	4.228339	0.335340
H	-3.104582	2.338783	1.318090
C	-3.456764	0.971726	-2.029817
C	-3.604496	-1.438107	-2.214693
H	-0.910909	1.015000	3.023762
C	-2.709115	0.423075	4.038757
C	-4.398851	-0.627323	2.671901
H	-3.922702	-0.857045	0.588950
H	0.892151	-3.414338	-0.226887
C	1.249997	-4.262422	-2.168275
C	0.099806	-3.206937	-4.012638
H	-1.126596	-1.512605	-3.518417
H	1.396279	-2.433140	4.638155

C	-0.208684	-3.628706	3.834021
H	-1.809411	-4.614276	2.775039
H	-1.088690	5.592648	-1.846837
C	-2.758597	5.150870	-0.550868
H	-4.288656	4.428609	0.787822
H	-2.961035	1.861190	-1.666747
C	-4.583974	1.096550	-2.839859
C	-4.729701	-1.308326	-3.022027
H	-3.228771	-2.423224	-1.957318
H	-2.363437	0.779128	5.004522
C	-3.968783	-0.169858	3.916769
H	-5.375337	-1.090704	2.567904
H	1.916292	-5.030042	-1.786429
C	0.961520	-4.197964	-3.532230
H	-0.122314	-3.146586	-5.073849
H	-0.236221	-4.310387	4.678673
H	-3.290057	6.066218	-0.791382
H	-4.959993	2.085844	-3.082746
C	-5.224670	-0.039214	-3.337044
H	-5.226081	-2.197046	-3.399813
H	-4.608557	-0.274019	4.787817
H	1.405433	-4.913045	-4.218134
H	-6.105070	0.061726	-3.964410
H	1.620644	4.250240	0.210904
H	4.010874	4.804823	-0.003940
H	5.369425	-1.865417	-0.040948
H	2.933449	-2.300805	0.222227
H	5.742225	3.088786	-0.209459
N	6.629868	0.371694	-0.295961
O	7.058721	1.344151	-0.915761
O	7.330651	-0.518026	0.181147

γ-OMe-TP

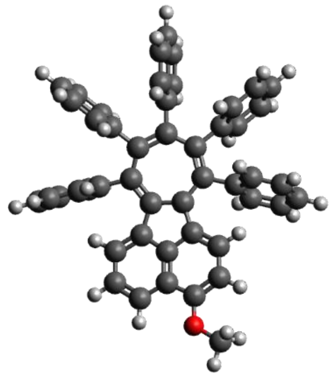

Table S96. Coordinates and energy for the optimized geometry of **γ-OMe-TP**.

G = –1923.679556			
		**Coordinates / Å**	
**Atom**	**x**	**y**	**z**

C	1.537265	-1.006422	-0.006393
C	1.796803	0.380546	-0.008250
C	0.286911	-1.678022	0.058363
C	-0.997138	-1.119670	0.107852
C	-1.378748	0.285430	-0.034804
C	-0.538161	1.416320	-0.008962
C	0.872744	1.458180	0.016886
C	-2.835769	0.336096	-0.133398
C	-3.799060	1.322325	-0.382231
C	-5.162082	0.991008	-0.404298
C	-5.613167	-0.313032	-0.168731
C	-2.243093	-1.898004	0.235672
C	-2.570545	-3.229579	0.505117
C	-3.307418	-0.984727	0.056595
C	-3.926540	-3.610926	0.553189
C	-4.661805	-1.356022	0.073883
C	-4.961226	-2.711248	0.329880
H	-5.993878	-3.040120	0.359137
C	-1.230322	2.745153	0.021178
C	-1.326357	3.531192	-1.131184
C	-1.805665	3.191667	1.216075
C	-2.002859	4.750123	-1.090037
H	-0.879865	3.185369	-2.057628
C	-2.471299	4.415463	1.257930
H	-1.734209	2.577087	2.108283
C	-2.575951	5.194615	0.103630
H	-2.080608	5.352190	-1.990162
H	-2.912060	4.757248	2.189432
H	-3.099646	6.145212	0.134279
C	1.477212	2.835336	0.040841
C	1.486388	3.579652	1.224910
C	2.031407	3.383866	-1.120502
C	2.050746	4.853853	1.249275
H	1.053157	3.159891	2.127179
C	2.587658	4.661424	-1.097293
H	2.028209	2.809098	-2.041064
C	2.601572	5.398872	0.088132
H	2.057801	5.420453	2.175591
H	3.012261	5.078779	-2.005406
H	3.038735	6.392650	0.106721
C	3.248575	0.772271	-0.039898
C	3.981156	0.660757	-1.226119
C	3.876863	1.252602	1.113824
C	5.324581	1.030029	-1.259364
H	3.497818	0.286309	-2.122712
C	5.222640	1.612759	1.081966
H	3.311428	1.344834	2.035394
C	5.949734	1.504900	-0.105018

H	5.882467	0.944153	-2.187005
H	5.701752	1.979954	1.984622
H	6.997368	1.789326	-0.130118
C	2.746379	-1.900340	-0.041202
C	3.517838	-2.084411	1.110871
C	3.106569	-2.557408	-1.222090
C	4.635451	-2.916571	1.082374
H	3.241534	-1.575245	2.028688
C	4.228997	-3.383038	-1.251994
H	2.509007	-2.419308	-2.117501
C	4.995458	-3.566354	-0.099634
H	5.225442	-3.054816	1.983454
H	4.502953	-3.883889	-2.175723
H	5.868234	-4.211971	-0.122641
C	0.357493	-3.174632	0.039631
C	0.702100	-3.892551	1.188922
C	0.058461	-3.859135	-1.143482
C	0.735710	-5.286269	1.156704
H	0.935117	-3.361367	2.105848
C	0.104289	-5.251755	-1.176741
H	-0.214581	-3.299063	-2.032636
C	0.437834	-5.968176	-0.025394
H	0.995994	-5.838507	2.054495
H	-0.125989	-5.776369	-2.098977
H	0.467484	-7.053232	-0.049118
H	-1.821768	-3.983646	0.691177
H	-3.535495	2.350386	-0.576745
H	-4.162504	-4.648420	0.765655
H	-5.873649	1.781668	-0.603904
O	-6.897872	-0.685161	-0.156289
C	-7.913961	0.299065	-0.393297
H	-8.858758	-0.238989	-0.331841
H	-7.878056	1.080769	0.371505
H	-7.798702	0.739588	-1.388261

γ-OMe-DT

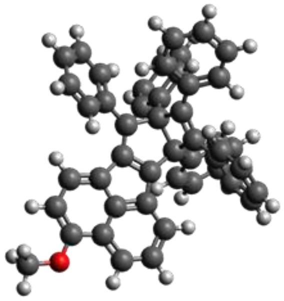

Table S97. Coordinates and energy for the optimized geometry of **γ-OMe-DT**.

G = –1923.647597

	Coordinates / Å		
Atom	x	y	z
C	4.649215	-0.210156	-0.076505
C	5.124655	-1.538030	-0.120809
C	3.263841	-0.042706	0.053779
H	6.188545	-1.720957	-0.226321
C	4.243055	-2.623328	-0.024027
C	2.354353	-1.145526	0.169448
C	2.860627	-2.450470	0.129400
C	1.067716	-0.564396	0.331749
C	2.602075	1.205576	0.117241
C	1.178899	0.862806	0.271055
C	-0.334103	-1.009913	0.487967
C	3.391608	2.345010	0.081694
C	5.433354	0.993691	-0.136488
C	-0.072440	1.458386	0.327522
C	-1.147232	0.388913	0.494610
C	-1.022355	-1.253022	-0.876978
C	-0.633953	-1.982006	1.604254
C	4.795778	2.231349	-0.047686
C	-0.347779	2.868819	0.115735
C	-1.747989	-0.112191	-0.856011
C	-2.078127	0.583608	1.667433
C	-0.765000	-2.339278	-1.815341
C	0.251654	-2.144959	2.675590
C	-1.851615	-2.674650	1.618585
C	0.430646	3.578526	-0.825943
C	-1.409355	3.539815	0.761644
C	-2.804031	0.435231	-1.705743
C	-1.522321	0.845155	2.929020
C	-3.462691	0.439164	1.550691
C	-0.263007	-3.571777	-1.360510
C	-0.950087	-2.154107	-3.198745
H	1.193921	-1.606885	2.688499
C	-0.070117	-2.989472	3.738770
C	-2.174649	-3.514411	2.681858
H	-2.547668	-2.552428	0.795470
H	1.208116	3.058453	-1.372540
C	0.156932	4.910146	-1.111279
C	-1.658992	4.879970	0.489553
H	-2.017685	3.016836	1.486070
C	-2.991752	1.818239	-1.871067
C	-3.693562	-0.447205	-2.351241
H	-0.446021	0.949333	3.030771
C	-2.337874	0.967470	4.050149
C	-4.280804	0.564585	2.675784
H	-3.907828	0.229674	0.585713
H	-0.127849	-3.735594	-0.297344

C	0.032582	-4.593347	-2.261185
C	-0.651615	-3.176077	-4.094163
H	-1.314224	-1.200855	-3.566193
H	0.628546	-3.104999	4.562103
C	-1.284339	-3.675846	3.745999
H	-3.122445	-4.044379	2.677899
H	0.747354	5.433351	-1.856449
C	-0.882906	5.567764	-0.447479
H	-2.466571	5.388697	1.005922
H	-2.325483	2.515727	-1.382998
C	-4.022945	2.302077	-2.673913
C	-4.722226	0.040104	-3.150620
H	-3.577157	-1.516559	-2.209130
H	-1.893234	1.168061	5.020312
C	-3.723190	0.828806	3.926048
H	-5.355795	0.452646	2.570229
H	0.413285	-5.540821	-1.891583
C	-0.160832	-4.400381	-3.629863
H	-0.794966	-3.015441	-5.158584
H	-1.535974	-4.332347	4.573509
H	-1.091950	6.610505	-0.665034
H	-4.147547	3.374161	-2.794090
C	-4.890370	1.418135	-3.317221
H	-5.399687	-0.655055	-3.637418
H	-4.360991	0.925074	4.799560
H	0.071158	-5.195881	-4.331551
H	-5.695350	1.798011	-3.939091
H	2.961465	3.337541	0.157271
H	5.378844	3.142661	-0.076520
H	4.647674	-3.628831	-0.060327
H	2.207139	-3.310916	0.214853
O	6.756779	0.813495	-0.268433
C	7.608392	1.963733	-0.329068
H	8.619428	1.572874	-0.435429
H	7.355754	2.586128	-1.193444
H	7.530234	2.551323	0.591156

γ-OMe-DT′

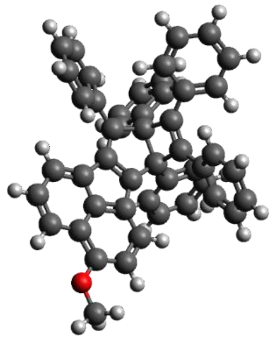

Table S98. Coordinates and energy for the optimized geometry of **γ-OMe-DT′**.

G = –1923.654501			
		**Coordinates / Å**	
**Atom**	**x**	**y**	**z**
C	4.305261	1.762237	-0.073703
C	5.268812	0.696379	-0.075926
C	2.971716	1.372026	0.057792
C	4.855676	-0.651845	0.062953
C	2.539923	0.009872	0.213210
C	3.516753	-1.000225	0.213505
C	1.140818	0.058513	0.354105
C	1.882535	2.278700	0.079729
C	0.691982	1.422161	0.247370
C	0.007421	-0.880308	0.514901
C	2.161808	3.632592	-0.005887
C	4.568028	3.151588	-0.178118
C	-0.683692	1.501667	0.292132
C	-1.279030	0.105218	0.480556
C	-0.508194	-1.385706	-0.851854
C	0.081155	-1.869129	1.652795
C	3.511587	4.049063	-0.135733
C	-1.469768	2.705634	0.051836
C	-1.619197	-0.611484	-0.860188
C	-2.231841	-0.042465	1.641838
C	0.180425	-2.286137	-1.770152
C	0.931594	-1.649760	2.742659
C	-0.770071	-2.981555	1.669092
C	-1.042909	3.602103	-0.950853
C	-2.671560	2.978055	0.738055
C	-2.785074	-0.511196	-1.734345
C	-1.835685	0.431631	2.901828
C	-3.457243	-0.702145	1.519583
C	1.089341	-3.242914	-1.283156
C	0.010274	-2.164894	-3.162464
H	1.587278	-0.784972	2.754010
C	0.937372	-2.530064	3.825291
C	-0.766987	-3.858430	2.751479
H	-1.437416	-3.157494	0.831912
H	-0.149912	3.375379	-1.521806
C	-1.792786	4.731570	-1.257993
C	-3.404155	4.122631	0.440293
H	-3.016218	2.306676	1.512345
C	-3.544637	0.668224	-1.825729
C	-3.199683	-1.636724	-2.474853
H	-0.880137	0.936498	3.009634
C	-2.654525	0.258347	4.013878
C	-4.279904	-0.873995	2.635224

H	-3.774660	-1.083985	0.556949
H	1.225192	-3.356390	-0.213473
C	1.793634	-4.064635	-2.161213
C	0.717163	-2.985815	-4.035425
H	-0.666536	-1.413260	-3.554131
H	1.603486	-2.345721	4.662976
C	0.088005	-3.636589	3.833552
H	-1.432305	-4.716680	2.748523
H	-1.462321	5.398043	-2.048291
C	-2.972447	4.999306	-0.558295
H	-4.318796	4.328342	0.987204
H	-3.257822	1.540100	-1.254268
C	-4.663433	0.729199	-2.653973
C	-4.318279	-1.572237	-3.298788
H	-2.645381	-2.565026	-2.387691
H	-2.333319	0.629992	4.982370
C	-3.883280	-0.395048	3.883028
H	-5.231145	-1.386068	2.524333
H	2.485389	-4.803029	-1.766741
C	1.610067	-3.940596	-3.539279
H	0.577661	-2.875780	-5.106790
H	0.091377	-4.321742	4.675983
H	-3.555535	5.884022	-0.794366
H	-5.232366	1.652183	-2.715391
C	-5.053418	-0.386764	-3.395770
H	-4.623465	-2.450702	-3.859525
H	-4.523786	-0.530362	4.749392
H	2.159928	-4.580022	-4.223259
H	-5.927539	-0.338026	-4.038125
H	1.376445	4.379315	0.026718
H	3.719015	5.111771	-0.205246
H	5.599484	-1.437549	0.060470
H	3.245202	-2.043549	0.327078
H	5.587417	3.504847	-0.282393
O	6.534062	1.070851	-0.209433
C	7.587575	0.089399	-0.216499
H	7.457160	-0.603167	-1.051992
H	8.505975	0.659750	-0.342391
H	7.608378	-0.452920	0.732506

α-F-TP

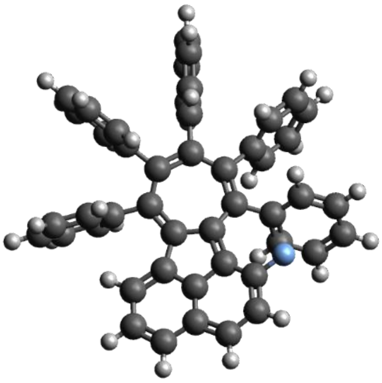

Table S99. Coordinates and energy for the optimized geometry of **α-F-TP**.

		G = –1908.408385	
		**Coordinates / Å**	
**Atom**	**x**	**y**	**z**
C	-1.500977	0.582322	-0.142708
C	-1.274102	-0.802812	-0.284173
C	-0.553729	1.602102	0.123118
C	0.836093	1.513157	-0.047515
C	1.628552	0.319292	-0.293067
C	1.248275	-1.018002	-0.031495
C	-0.074837	-1.516778	-0.027519
C	2.986361	0.759322	-0.621402
C	4.149222	0.186438	-1.129432
C	5.312896	0.952411	-1.348642
C	5.330349	2.314321	-1.116722
H	6.188223	0.435476	-1.725148
H	6.237073	2.883043	-1.297047
C	1.754684	2.668989	-0.079370
C	1.636123	4.046283	0.110730
C	3.022337	2.167313	-0.453517
C	2.769250	4.870527	-0.068841
H	0.706277	4.513862	0.393741
C	4.153448	2.974259	-0.674710
C	4.001136	4.364942	-0.454307
H	2.658910	5.937348	0.094700
H	4.848948	5.028845	-0.593376
C	2.345428	-1.968213	0.318895
C	2.589836	-3.144306	-0.403318
C	3.135886	-1.673733	1.438158
C	3.601396	-4.011989	-0.007144
H	2.001080	-3.367862	-1.284555
C	4.140963	-2.551931	1.841446
H	2.948353	-0.766308	2.004039
C	4.375459	-3.722673	1.120062
H	3.787455	-4.915661	-0.579327
H	4.736822	-2.320801	2.719090

H	5.158731	-4.406851	1.432151
C	-0.254146	-2.962210	0.344129
C	-0.126574	-3.304686	1.695528
C	-0.557935	-3.955845	-0.592209
C	-0.305025	-4.624949	2.105611
H	0.114536	-2.535285	2.422647
C	-0.726424	-5.276307	-0.181543
H	-0.660975	-3.697636	-1.639844
C	-0.603258	-5.614470	1.167657
H	-0.208927	-4.878469	3.156995
H	-0.956372	-6.040789	-0.917417
H	-0.739184	-6.643635	1.485875
C	-2.483473	-1.639255	-0.588581
C	-2.770303	-1.945223	-1.922529
C	-3.313227	-2.121914	0.427935
C	-3.877910	-2.732961	-2.237279
H	-2.125357	-1.569359	-2.711631
C	-4.416427	-2.911044	0.111203
H	-3.089044	-1.886176	1.463021
C	-4.701111	-3.219324	-1.221267
H	-4.093826	-2.966653	-3.275461
H	-5.054244	-3.285855	0.905982
H	-5.561504	-3.835031	-1.465327
C	-2.938131	1.020433	-0.187952
C	-3.647181	1.231393	0.999462
C	-3.570549	1.229796	-1.417117
C	-4.976334	1.647657	0.956194
H	-3.156923	1.072778	1.954614
C	-4.898544	1.652913	-1.458182
H	-3.022029	1.066129	-2.339203
C	-5.604366	1.862041	-0.272373
H	-5.520012	1.805856	1.882678
H	-5.380220	1.817487	-2.417285
H	-6.639026	2.189694	-0.305044
C	-1.102999	2.926505	0.546351
C	-1.021084	3.279255	1.897722
C	-1.677815	3.812250	-0.371567
C	-1.516578	4.509424	2.329679
H	-0.567900	2.591352	2.605067
C	-2.162765	5.044592	0.061697
H	-1.738004	3.538676	-1.419234
C	-2.084823	5.394883	1.412102
H	-1.454278	4.776002	3.380222
H	-2.602073	5.731108	-0.655389
H	-2.466962	6.354230	1.747234
F	4.220894	-1.101188	-1.473548

α-F-DT

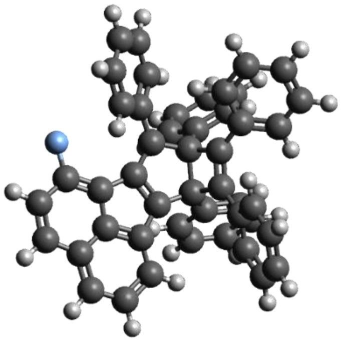

Table S100. Coordinates and energy for the optimized geometry of **α-F-DT**.

G = –1908.383827			
		**Coordinates / Å**	
**Atom**	**x**	**y**	**z**
C	4.766469	1.326618	-0.380663
C	5.617951	0.197965	-0.466537
C	3.405775	1.059368	-0.168669
C	5.116769	-1.100221	-0.326181
C	2.882149	-0.263224	-0.003913
C	3.757179	-1.352147	-0.084532
C	1.491726	-0.101392	0.248691
C	2.390392	2.043668	-0.047735
C	1.150810	1.286465	0.186989
C	0.310032	-0.955824	0.495193
C	2.819520	3.354888	-0.089873
C	5.140125	2.700150	-0.463611
C	-0.222678	1.465594	0.283772
C	-0.896532	0.119688	0.525146
C	-0.333659	-1.447518	-0.823869
C	0.389076	-1.931111	1.644980
C	4.181802	3.681042	-0.313403
C	-0.949534	2.693340	0.027187
C	-1.373328	-0.584306	-0.784481
C	-1.788474	0.054037	1.741650
C	0.192682	-2.440835	-1.752598
C	1.330125	-1.764957	2.667432
C	-0.544507	-2.970930	1.742046
C	-0.500268	3.540314	-1.010260
C	-2.146529	3.019432	0.703766
C	-2.588109	-0.426967	-1.583948
C	-1.287905	0.521717	2.966202
C	-3.060507	-0.522043	1.701150
C	1.075276	-3.440528	-1.306009
C	-0.125224	-2.382124	-3.122941
H	2.052541	-0.956672	2.615947
C	1.343308	-2.627338	3.764238

C	-0.533821	-3.829067	2.839126
H	-1.281375	-3.105640	0.957236
H	0.383972	3.269701	-1.574035
C	-1.229172	4.667608	-1.365756
C	-2.850084	4.167865	0.363930
H	-2.504287	2.385909	1.503211
C	-3.176346	0.826442	-1.824750
C	-3.214949	-1.577480	-2.103418
H	-0.296326	0.962745	3.009056
C	-2.049880	0.420653	4.126543
C	-3.825092	-0.621163	2.865632
H	-3.459757	-0.894171	0.765611
H	1.317568	-3.509563	-0.251415
C	1.615351	-4.361603	-2.201652
C	0.417779	-3.302727	-4.013311
H	-0.787977	-1.603753	-3.485023
H	2.080313	-2.485453	4.549023
C	0.411148	-3.661118	3.853985
H	-1.263651	-4.630789	2.899884
H	-0.887920	5.292893	-2.184239
C	-2.400265	4.989675	-0.674151
H	-3.757457	4.419153	0.903373
H	-2.712401	1.723998	-1.441210
C	-4.346247	0.926002	-2.575501
C	-4.383140	-1.473302	-2.851191
H	-2.782749	-2.552510	-1.903424
H	-1.648796	0.785220	5.067444
C	-3.324304	-0.151217	4.079074
H	-4.813190	-1.069215	2.819629
H	2.290108	-5.131025	-1.838563
C	1.288897	-4.296905	-3.557034
H	0.166724	-3.241191	-5.068034
H	0.419997	-4.331960	4.707753
H	-2.964358	5.876146	-0.946966
H	-4.782413	1.903949	-2.756105
C	-4.953800	-0.219668	-3.091141
H	-4.854333	-2.370980	-3.240117
H	-3.920198	-0.230096	4.983341
H	1.710955	-5.013691	-4.254923
H	-5.867547	-0.138893	-3.672126
H	4.445627	4.731885	-0.352140
H	5.802327	-1.937544	-0.395581
H	3.402366	-2.369034	0.035052
H	6.176542	2.974474	-0.630293
H	6.680636	0.342853	-0.637677
F	1.968349	4.373594	0.086410

α-F-DT′

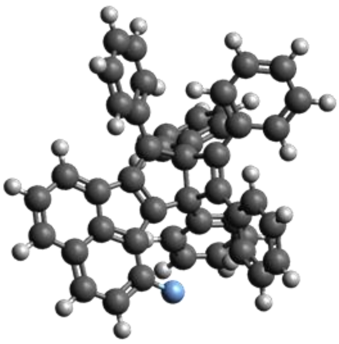

Table S101. Coordinates and energy for the optimized geometry of **α-F-DT′**.

	G = –1908.384643		
	**Coordinates / Å**		
**Atom**	**x**	**y**	**z**
C	4.371309	2.404033	-0.529449
C	5.454244	1.483200	-0.549627
C	3.099593	1.856298	-0.313149
C	5.249990	0.123958	-0.335762
C	2.851275	0.459957	-0.093074
C	3.963256	-0.382739	-0.095561
C	1.453649	0.334390	0.136531
C	1.907828	2.616669	-0.231064
C	0.850413	1.626784	0.045398
C	0.460070	-0.728565	0.416265
C	2.011801	3.993912	-0.344000
C	4.440950	3.816633	-0.675158
C	-0.524227	1.548870	0.237634
C	-0.917982	0.112823	0.551687
C	-0.198523	-1.321340	-0.859680
C	0.785651	-1.690459	1.534078
C	3.286412	4.574967	-0.574611
C	-1.478576	2.618538	0.033593
C	-1.372941	-0.671264	-0.712987
C	-1.666699	-0.092621	1.846052
C	0.388844	-2.219209	-1.846542
C	1.694535	-1.338532	2.538796
C	0.118187	-2.918022	1.623677
C	-1.226195	3.578974	-0.973250
C	-2.697993	2.683989	0.744914
C	-2.665737	-0.742665	-1.400972
C	-1.185568	0.522177	3.011612
C	-2.777570	-0.935866	1.929090
C	1.537733	-2.967033	-1.538582
C	-0.159770	-2.320235	-3.140142
H	2.209694	-0.384161	2.496373
C	1.939284	-2.199986	3.607572

C	0.359509	-3.777640	2.693454
H	-0.592490	-3.200971	0.854842
H	-0.327667	3.498654	-1.572835
C	-2.157883	4.568954	-1.255119
C	-3.611887	3.694773	0.475055
H	-2.912776	1.956657	1.515099
C	-3.408383	0.400138	-1.740395
C	-3.197218	-2.009938	-1.710675
H	-0.319474	1.175265	2.958346
C	-1.809557	0.301637	4.236207
C	-3.403777	-1.155037	3.157902
H	-3.159265	-1.421264	1.039210
H	1.970234	-2.901553	-0.549136
C	2.116517	-3.801404	-2.493032
C	0.422605	-3.153480	-4.089146
H	-1.033972	-1.733788	-3.399305
H	2.648720	-1.911545	4.377465
C	1.272270	-3.422738	3.688636
H	-0.165586	-4.726608	2.747282
H	-1.962796	5.282618	-2.048796
C	-3.348649	4.636018	-0.524369
H	-4.537142	3.744476	1.039560
H	-3.013360	1.384490	-1.531110
C	-4.643699	0.277517	-2.375086
C	-4.431299	-2.127904	-2.342749
H	-2.636003	-2.899195	-1.440986
H	-1.425887	0.783733	5.130356
C	-2.923362	-0.538967	4.312848
H	-4.267847	-1.810866	3.207988
H	3.002614	-4.374459	-2.236688
C	1.561425	-3.899932	-3.769526
H	-0.008635	-3.216994	-5.083840
H	1.461715	-4.094279	4.520639
H	-4.073333	5.414803	-0.740148
H	-5.201087	1.172499	-2.635149
C	-5.160842	-0.983105	-2.676025
H	-4.827345	-3.113205	-2.569593
H	-3.411611	-0.711003	5.267328
H	2.013064	-4.549937	-4.512952
H	-6.125365	-1.074785	-3.166252
H	3.350231	5.653738	-0.669614
H	6.082873	-0.569580	-0.335573
H	5.398820	4.295982	-0.850421
H	6.464021	1.843211	-0.721862
H	1.147062	4.640997	-0.252007
F	3.830944	-1.685790	0.152684

α-NO_2-TP

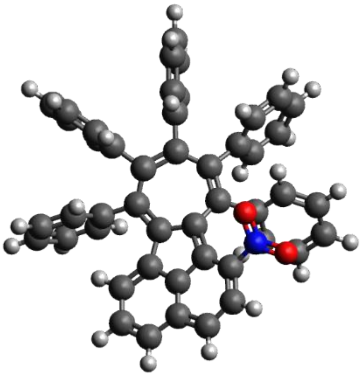

Table S102. Coordinates and energy for the optimized geometry of **α-NO_2-TP**.

G = –2012.658527			
	Coordinates / Å		
Atom	x	y	z
C	-1.766948	0.293858	-0.145451
C	-1.265909	-1.024703	-0.226809
C	-1.055644	1.479072	0.163531
C	0.329517	1.660435	0.048315
C	1.330208	0.651831	-0.221574
C	1.236375	-0.723163	0.095021
C	0.039191	-1.478473	0.096763
C	2.578394	1.357171	-0.559516
C	3.817174	1.033834	-1.106117
C	4.838036	1.996554	-1.226218
C	4.620075	3.319456	-0.875547
H	5.790532	1.683035	-1.634756
H	5.418216	4.045639	-0.988269
C	1.024276	2.968277	0.098282
C	0.661625	4.280534	0.374099
C	2.366703	2.725259	-0.280316
C	1.641525	5.303929	0.283710
H	-0.341139	4.558885	0.658729
C	3.344699	3.729205	-0.419225
C	2.945364	5.055764	-0.101465
H	1.340134	6.319419	0.518074
H	3.664970	5.865643	-0.167960
C	2.500508	-1.386770	0.536140
C	2.998010	-2.568027	-0.035849
C	3.196310	-0.803638	1.607377
C	4.163698	-3.147011	0.453687
H	2.490225	-3.015074	-0.879168
C	4.352459	-1.398177	2.109307
H	2.816815	0.106195	2.062078
C	4.839702	-2.570654	1.532280
H	4.545942	-4.050752	-0.010072
H	4.869173	-0.943589	2.948724

H	5.744340	-3.031938	1.916362
C	0.141156	-2.917375	0.512584
C	0.306283	-3.191946	1.875312
C	0.062724	-3.974360	-0.400699
C	0.381909	-4.510744	2.320505
H	0.377737	-2.372078	2.583318
C	0.150793	-5.290841	0.045803
H	-0.060127	-3.767118	-1.457555
C	0.306318	-5.562944	1.406700
H	0.502923	-4.713656	3.380216
H	0.096273	-6.104205	-0.671237
H	0.369510	-6.590168	1.752660
C	-2.279869	-2.091423	-0.524531
C	-2.477167	-2.487874	-1.850529
C	-3.016226	-2.696648	0.498513
C	-3.402483	-3.487798	-2.150918
H	-1.904107	-2.018048	-2.644321
C	-3.937347	-3.696757	0.195858
H	-2.859325	-2.391610	1.527954
C	-4.132115	-4.095435	-1.128696
H	-3.549102	-3.791422	-3.183033
H	-4.503009	-4.165306	0.995486
H	-4.850110	-4.875803	-1.361925
C	-3.251287	0.451450	-0.310363
C	-4.089360	0.533996	0.806584
C	-3.797528	0.538029	-1.594669
C	-5.462316	0.701754	0.638440
H	-3.666459	0.470271	1.803847
C	-5.171098	0.713235	-1.759856
H	-3.147544	0.473393	-2.461639
C	-6.005958	0.794934	-0.644447
H	-6.106461	0.761895	1.510415
H	-5.586721	0.784594	-2.760393
H	-7.075546	0.929800	-0.773724
C	-1.870804	2.669495	0.554937
C	-1.974059	2.991633	1.912458
C	-2.510127	3.464391	-0.402351
C	-2.715987	4.103863	2.310510
H	-1.472425	2.374466	2.651680
C	-3.244518	4.578670	-0.001081
H	-2.429628	3.211630	-1.454074
C	-3.349159	4.900237	1.354546
H	-2.795386	4.348169	3.365274
H	-3.735235	5.195565	-0.747461
H	-3.923768	5.767754	1.664170
N	4.085774	-0.272507	-1.708145
O	3.156140	-0.804045	-2.315603
O	5.218377	-0.734789	-1.606472

α-NO₂-DT

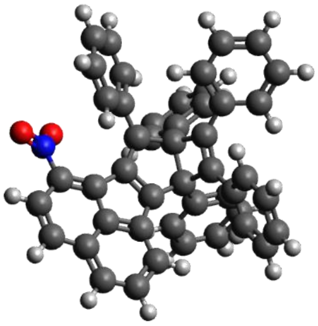

Table S103. Coordinates and energy for the optimized geometry of **α-NO_2-DT**.

G = –2012.637903			
	Coordinates / Å		
Atom	x	y	z
C	4.304333	-2.105139	-0.394631
C	4.229384	-3.522248	-0.505440
C	3.097964	-1.444412	-0.127507
C	3.022775	-4.194541	-0.329454
C	1.855883	-2.126786	0.066397
C	1.823242	-3.516741	-0.028240
C	0.893244	-1.109992	0.342718
C	2.957254	-0.039758	0.032518
C	1.514114	0.173986	0.261254
C	-0.560553	-1.017491	0.574609
C	4.133413	0.687284	-0.022220
C	5.467061	-1.295332	-0.513528
C	0.555183	1.179400	0.253188
C	-0.822219	0.574546	0.497155
C	-1.331809	-1.084766	-0.766828
C	-1.124051	-1.757987	1.761120
C	5.372388	0.069647	-0.325597
C	0.764840	2.560961	-0.132091
C	-1.603652	0.239511	-0.816242
C	-1.584721	1.171434	1.655469
C	-1.499707	-2.243361	-1.636730
C	-0.294015	-2.171417	2.809061
C	-2.505551	-1.965431	1.860715
C	1.620781	2.831700	-1.222052
C	0.084968	3.630687	0.487383
C	-2.438589	1.064507	-1.685619
C	-0.924834	1.326517	2.884317
C	-2.939345	1.500806	1.562927
C	-1.545547	-3.543811	-1.102989
C	-1.553901	-2.082095	-3.034213
H	0.777475	-2.004716	2.753872
C	-0.835406	-2.790082	3.936357

C	-3.045684	-2.578960	2.988474
H	-3.156319	-1.644343	1.054158
H	2.110767	2.010260	-1.734231
C	1.786129	4.130526	-1.682320
C	0.283201	4.931967	0.040535
H	-0.567062	3.440999	1.329023
C	-2.188239	2.432183	-1.892577
C	-3.561441	0.482895	-2.309770
H	0.126182	1.066842	2.969756
C	-1.607569	1.810030	3.996872
C	-3.622767	1.985928	2.679756
H	-3.466180	1.380284	0.624447
H	-1.518312	-3.684305	-0.028121
C	-1.656137	-4.650207	-1.943596
C	-1.662815	-3.189805	-3.868648
H	-1.497126	-1.085591	-3.458988
H	-0.179536	-3.106399	4.741829
C	-2.212133	-2.994808	4.029485
H	-4.118408	-2.733996	3.052770
H	2.429003	4.321952	-2.535031
C	1.124555	5.185431	-1.046338
H	-0.224777	5.751378	0.538420
H	-1.339749	2.904113	-1.418781
C	-3.019728	3.187363	-2.716635
C	-4.388900	1.240979	-3.130608
H	-3.785249	-0.563775	-2.133938
H	-1.084118	1.924836	4.941149
C	-2.961194	2.142662	3.897094
H	-4.675114	2.239337	2.593698
H	-1.698226	-5.647076	-1.515235
C	-1.715351	-4.477981	-3.327290
H	-1.699085	-3.049392	-4.944795
H	-2.634126	-3.475197	4.907012
H	1.263316	6.202671	-1.398597
H	-2.805442	4.240939	-2.868727
C	-4.119933	2.597160	-3.340163
H	-5.250643	0.776642	-3.600471
H	-3.494825	2.520156	4.764083
H	-1.799728	-5.340595	-3.981234
H	-4.768160	3.189041	-3.979209
H	6.252384	0.697980	-0.381587
H	3.005276	-5.275243	-0.415605
H	0.905245	-4.072086	0.122368
H	6.429752	-1.746870	-0.727950
H	5.129609	-4.087994	-0.725037
N	4.161955	2.114963	0.304374
O	3.380869	2.505131	1.170149
O	4.970378	2.824325	-0.291666

α-NO$_2$-DT′

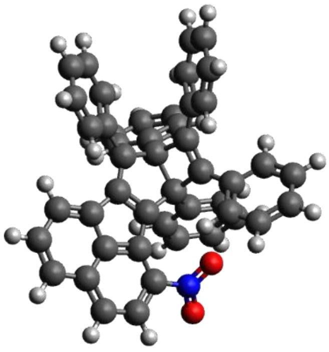

Table S104. Coordinates and energy for the optimized geometry of **α-NO$_2$-DT′**.

G = –2012.633752			
		**Coordinates / Å**	
**Atom**	**x**	**y**	**z**
C	3.672556	3.255451	-0.736035
C	4.925531	2.606137	-0.831832
C	2.556088	2.434477	-0.504553
C	5.019350	1.228102	-0.674247
C	2.608872	1.010247	-0.357762
C	3.880745	0.437306	-0.447256
C	1.252845	0.599058	-0.059458
C	1.240500	2.935297	-0.330480
C	0.425709	1.748032	-0.031461
C	0.491823	-0.633930	0.261770
C	1.046357	4.301638	-0.376108
C	3.436627	4.659650	-0.813925
C	-0.890611	1.419312	0.315458
C	-0.953203	-0.031633	0.738390
C	-0.367252	-1.282014	-0.878885
C	1.162064	-1.575549	1.230549
C	2.159872	5.151972	-0.632582
C	-2.059173	2.239843	0.140867
C	-1.558639	-0.836768	-0.428020
C	-1.317920	-0.330817	2.166764
C	0.019313	-2.145156	-1.990321
C	1.877321	-1.037912	2.310089
C	1.017826	-2.963081	1.136850
C	-2.079537	3.226409	-0.875181
C	-3.227532	2.029184	0.909708
C	-2.926765	-0.877167	-0.942700
C	-1.066884	0.605919	3.177149
C	-1.798032	-1.598527	2.514974
C	-0.769090	-3.268175	-2.305759
C	1.134595	-1.846361	-2.788926
H	1.974011	0.039154	2.411800
C	2.446365	-1.871445	3.269530

C	1.586987	-3.797787	2.097951
H	0.471294	-3.397203	0.308248
H	-1.215535	3.348226	-1.517021
C	-3.221303	3.978778	-1.102615
C	-4.359043	2.801741	0.690279
H	-3.224419	1.287199	1.696513
C	-4.034626	-0.926346	-0.079961
C	-3.149562	-0.827803	-2.331015
H	-0.684392	1.589776	2.921611
C	-1.304450	0.283763	4.512802
C	-2.032599	-1.920955	3.850292
H	-1.974914	-2.337063	1.740054
H	-1.628645	-3.503743	-1.686393
C	-0.447494	-4.069636	-3.396442
C	1.447748	-2.646760	-3.884828
H	1.749256	-0.987079	-2.551549
H	2.994641	-1.438620	4.100898
C	2.303029	-3.256716	3.166082
H	1.470613	-4.873728	2.008396
H	-3.234089	4.715484	-1.898870
C	-4.360214	3.774264	-0.314175
H	-5.244795	2.642739	1.295949
H	-3.880937	-0.970986	0.991989
C	-5.330203	-0.934566	-0.593251
C	-4.444859	-0.833359	-2.839304
H	-2.300293	-0.771544	-3.003756
H	-1.109402	1.020871	5.285828
C	-1.787289	-0.980778	4.853091
H	-2.404963	-2.908184	4.106203
H	-1.058392	-4.937142	-3.626868
C	0.661535	-3.760405	-4.189501
H	2.308707	-2.403472	-4.500030
H	2.744987	-3.908275	3.913851
H	-5.251771	4.367900	-0.490310
H	-6.176007	-0.976452	0.086450
C	-5.540084	-0.886980	-1.972136
H	-4.601403	-0.788485	-3.912964
H	-1.971793	-1.232165	5.893073
H	0.913965	-4.387855	-5.039014
H	-6.550318	-0.888961	-2.370322
H	1.990102	6.222463	-0.675835
H	5.981830	0.735680	-0.724746
H	4.266924	5.333526	-0.999300
H	5.825601	3.183722	-1.015324
H	0.070824	4.745464	-0.213664
N	4.099105	-1.004374	-0.308061
O	3.198922	-1.750147	-0.685955
O	5.172553	-1.375649	0.163237

α-NMe₂-TP

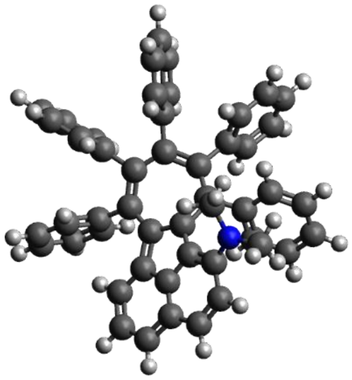

Table S105. Coordinates and energy for the optimized geometry of **α-NMe$_2$-TP**.

	G = –1942.118694		
		**Coordinates / Å**	
**Atom**	**x**	**y**	**z**
C	1.798934	0.270046	0.144343
C	1.295736	-1.035560	0.165573
C	1.098144	1.474553	-0.168590
C	-0.273966	1.670366	-0.080989
C	-1.282132	0.663472	0.268458
C	-1.206211	-0.716516	-0.095859
C	-0.029153	-1.472750	-0.157769
C	-2.486915	1.358984	0.617381
C	-3.734560	0.972870	1.178184
C	-4.819606	1.912357	1.048485
C	-4.625140	3.201450	0.615079
H	-5.804152	1.622989	1.393583
H	-5.468563	3.885703	0.598787
C	-0.981198	2.959265	-0.184490
C	-0.661677	4.258302	-0.592341
C	-2.305879	2.716511	0.244647
C	-1.670975	5.240671	-0.595949
H	0.329071	4.528446	-0.928002
C	-3.329527	3.674693	0.230243
C	-2.982346	4.968583	-0.207313
H	-1.416485	6.240637	-0.932234
H	-3.737882	5.747685	-0.253949
C	-2.501113	-1.326553	-0.550637
C	-3.030430	-2.522893	-0.047020
C	-3.208360	-0.656656	-1.562411
C	-4.242701	-3.019613	-0.519657
H	-2.497900	-3.076061	0.715331
C	-4.413824	-1.159374	-2.045577
H	-2.806892	0.262896	-1.975568
C	-4.941532	-2.339399	-1.518268
H	-4.639070	-3.941667	-0.105575
H	-4.939070	-0.628597	-2.833777
H	-5.884681	-2.730010	-1.888224
C	-0.144123	-2.893888	-0.630309

C	-0.311694	-3.127670	-1.999480
C	-0.077510	-3.980110	0.249438
C	-0.406111	-4.432041	-2.484234
H	-0.372476	-2.285686	-2.682431
C	-0.178626	-5.282376	-0.235033
H	0.054280	-3.804594	1.311720
C	-0.340495	-5.512282	-1.603255
H	-0.532434	-4.601984	-3.549312
H	-0.129302	-6.117869	0.456853
H	-0.415089	-6.528040	-1.979856
C	2.298155	-2.123986	0.429278
C	2.537288	-2.532025	1.744853
C	2.982990	-2.744814	-0.620229
C	3.446668	-3.556867	2.008508
H	2.007157	-2.049439	2.560705
C	3.890463	-3.768384	-0.356340
H	2.794262	-2.432738	-1.642249
C	4.123214	-4.178766	0.958425
H	3.624105	-3.868270	3.033669
H	4.415370	-4.246456	-1.178042
H	4.829396	-4.978036	1.162580
C	3.270645	0.436618	0.400020
C	4.192157	0.434792	-0.651808
C	3.721982	0.623382	1.710292
C	5.549788	0.617793	-0.395295
H	3.843217	0.294372	-1.669870
C	5.080844	0.807035	1.966535
H	3.007089	0.626891	2.527835
C	5.997482	0.805591	0.914185
H	6.257718	0.614460	-1.218809
H	5.421264	0.951830	2.987594
H	7.055344	0.949708	1.112669
C	1.935988	2.658197	-0.539306
C	2.175969	2.916761	-1.892879
C	2.458544	3.514772	0.434798
C	2.932715	4.026795	-2.269620
H	1.763999	2.252837	-2.647155
C	3.213419	4.623606	0.055960
H	2.271962	3.312495	1.484201
C	3.450958	4.882718	-1.296022
H	3.114272	4.222593	-3.322098
H	3.614706	5.286147	0.816848
H	4.038758	5.747399	-1.588909
C	-2.795043	-0.719871	2.687344
H	-3.178768	-0.910176	3.694695
H	-2.404900	-1.656437	2.283570
H	-1.983475	0.004590	2.749966
C	-5.207103	-0.724230	2.168712
H	-5.867312	-0.581764	1.312855
H	-5.099139	-1.797658	2.334279

H	-5.650637	-0.268574	3.063104
N	-3.885505	-0.175566	1.879263

α-NMe₂-DT

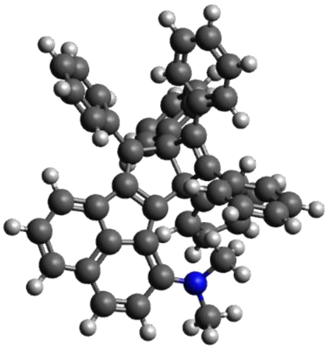

Table S106. Coordinates and energy for the optimized geometry of **α-NMe₂-DT**.

G = –1942.103339			
		**Coordinates / Å**	
**Atom**	**x**	**y**	**z**
C	3.279844	3.542993	-0.710834
C	4.608746	3.044849	-0.523637
C	2.258126	2.598478	-0.587676
H	5.450336	3.727207	-0.601691
C	4.850597	1.725444	-0.251859
C	2.449483	1.178307	-0.319132
C	3.818107	0.715607	-0.210529
C	1.162681	0.661913	-0.063892
C	0.893829	2.958045	-0.581945
C	0.190051	1.730384	-0.215394
C	0.466243	-0.591030	0.384420
C	0.544368	4.291162	-0.773953
C	2.910836	4.889006	-0.924071
C	-1.075424	1.348964	0.145576
C	-1.070728	-0.096075	0.618819
C	-0.168231	-1.440561	-0.764140
C	1.094062	-1.339625	1.536829
C	1.565431	5.243309	-0.964209
H	3.680450	5.647310	-1.034597
C	-2.280788	2.157439	-0.022831
C	-1.439696	-1.061402	-0.530180
C	-1.609398	-0.338650	2.005920
C	0.471677	-2.233742	-1.828409
C	2.023445	-0.721790	2.382212
C	0.698437	-2.653183	1.822588
C	-2.456391	2.855749	-1.233842
C	-3.290908	2.228051	0.955919
C	-2.722667	-1.354607	-1.162584
C	-1.189858	0.491027	3.056212
C	-2.441455	-1.424550	2.290138

C	0.922609	-3.546975	-1.627340
C	0.660974	-1.632307	-3.084409
H	2.319603	0.305438	2.202509
C	2.573492	-1.412094	3.462472
C	1.242818	-3.342948	2.903869
H	-0.050628	-3.132533	1.202564
H	-1.698055	2.778069	-2.005676
C	-3.612715	3.594604	-1.463695
C	-4.435271	2.986231	0.727741
H	-3.167552	1.708573	1.897460
C	-3.907375	-0.754209	-0.701899
C	-2.802323	-2.248346	-2.249716
H	-0.532217	1.330128	2.848085
C	-1.608332	0.247178	4.361606
C	-2.862737	-1.668754	3.598770
H	-2.762155	-2.082971	1.491011
H	0.784173	-4.022739	-0.663043
C	1.556093	-4.238973	-2.659452
C	1.295060	-2.326844	-4.113536
H	0.309082	-0.617027	-3.242174
H	3.298683	-0.916224	4.101009
C	2.190900	-2.727762	3.724041
H	0.923903	-4.361222	3.105565
H	-3.742840	4.109690	-2.410253
C	-4.603717	3.665268	-0.481754
H	-5.199944	3.044635	1.495969
H	-3.874352	-0.077372	0.141139
C	-5.128449	-1.020569	-1.316863
C	-4.024144	-2.513709	-2.859466
H	-1.904478	-2.732468	-2.614766
H	-1.276424	0.899037	5.164226
C	-2.449597	-0.834529	4.636893
H	-3.512581	-2.514455	3.803326
H	1.900120	-5.255246	-2.491869
C	1.746504	-3.631174	-3.902281
H	1.436051	-1.849523	-5.078682
H	2.620767	-3.266740	4.562953
H	-5.504246	4.245174	-0.658932
H	-6.029383	-0.540105	-0.947064
C	-5.192503	-1.898669	-2.399459
H	-4.065891	-3.203781	-3.697079
H	-2.777599	-1.024864	5.654399
H	2.241669	-4.172558	-4.702745
H	-6.144075	-2.106401	-2.879704
H	-0.492505	4.607605	-0.765837
H	1.292218	6.281810	-1.120308
H	5.878953	1.407537	-0.152004
C	5.528754	-0.955712	0.345450
H	5.468269	-1.969308	0.744894
H	6.235936	-0.948244	-0.492257

H	5.883452	-0.294621	1.136147
C	3.389787	-1.671458	-0.614128
H	3.009298	-2.307974	0.185176
H	2.567341	-1.280335	-1.201228
H	4.027231	-2.268926	-1.273975
N	4.183701	-0.569879	-0.085819

α-NMe₂-DT′

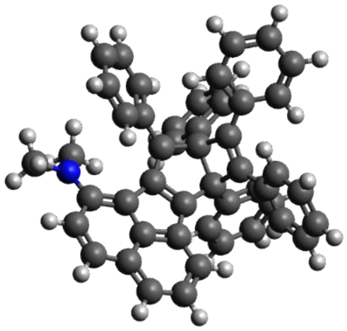

Table S107. Coordinates and energy for the optimized geometry of **α-NMe₂-DT′**.

	G = –1942.096248		
		Coordinates / Å	
**Atom**	**x**	**y**	**z**
C	-3.516571	3.189506	-0.488322
C	-3.066284	4.513240	-0.606888
C	-2.563348	2.223511	-0.112521
H	-3.764628	5.292299	-0.899782
C	-1.721209	4.855366	-0.359123
C	-1.181105	2.556558	0.116851
H	-1.418858	5.892072	-0.460192
C	-0.772989	3.900099	-0.001479
C	-0.532109	1.339044	0.393054
C	-2.815724	0.842741	0.038444
H	0.258266	4.186085	0.169158
C	-1.487414	0.260426	0.285968
C	0.849112	0.843211	0.602478
C	-4.155559	0.397167	-0.068444
C	-4.835031	2.692506	-0.730697
C	-0.821270	-0.950523	0.205571
C	0.666633	-0.759299	0.472468
C	1.644543	0.730292	-0.716141
C	1.580914	1.350421	1.822185
C	-5.124824	1.367306	-0.537258
H	-5.621692	3.373847	-1.040517
C	-1.398268	-2.208669	-0.245558
C	1.536895	-0.614102	-0.813032
C	1.204899	-1.582344	1.618300
C	2.172282	1.813575	-1.538583
C	0.892412	1.972436	2.869587
C	2.958947	1.131568	1.950467
H	-6.147456	1.041739	-0.675437

C	-2.333824	-2.181271	-1.300311
C	-1.023729	-3.451662	0.305547
C	2.122840	-1.606878	-1.715084
C	0.525053	-1.550925	2.845537
C	2.386272	-2.321567	1.515186
C	2.535011	3.042573	-0.959351
C	2.274036	1.668705	-2.935179
H	-0.176499	2.143140	2.788990
C	1.568404	2.371559	4.023495
C	3.632918	1.526180	3.103639
H	3.501977	0.648472	1.144866
H	-2.591580	-1.233134	-1.756814
C	-2.883531	-3.362476	-1.784894
C	-1.598575	-4.626304	-0.167554
H	-0.308779	-3.488669	1.116390
C	1.456386	-2.791559	-2.071167
C	3.420650	-1.387659	-2.219299
H	-0.386246	-0.967758	2.940160
C	1.015116	-2.249415	3.945280
C	2.875882	-3.024838	2.617983
H	2.927411	-2.352101	0.577406
H	2.473664	3.166613	0.116104
C	2.996972	4.091635	-1.752476
C	2.733744	2.719127	-3.723076
H	1.977675	0.733046	-3.396944
H	1.019618	2.853976	4.826985
C	2.939911	2.148476	4.144949
H	4.701025	1.349146	3.188048
H	-3.588600	-3.329555	-2.609293
C	-2.526637	-4.586591	-1.212463
H	-1.319564	-5.576099	0.277250
H	0.460560	-2.983735	-1.697886
C	2.061830	-3.717887	-2.918644
C	4.021556	-2.314989	-3.064247
H	3.956037	-0.488816	-1.931584
H	0.479897	-2.212087	4.889435
C	2.193816	-2.992127	3.833763
H	3.794219	-3.596651	2.522715
H	3.278763	5.031807	-1.287694
C	3.098420	3.934515	-3.135559
H	2.801105	2.593000	-4.799644
H	3.466520	2.458058	5.042747
H	-2.964834	-5.507671	-1.584137
H	1.527461	-4.624702	-3.186344
C	3.343500	-3.485097	-3.419029
H	5.023610	-2.129749	-3.439607
H	2.576860	-3.538802	4.690251
H	3.456419	4.752763	-3.753143
H	3.814055	-4.210502	-4.075911
C	-5.870914	-1.366285	-0.149655

H	-6.092163	-1.062451	-1.173608
H	-5.826322	-2.457650	-0.123041
H	-6.679490	-1.033934	0.515319
C	-3.990403	-1.585693	1.393134
H	-3.726913	-2.604378	1.100415
H	-3.104420	-1.077273	1.764710
H	-4.730173	-1.631776	2.203013
N	-4.569250	-0.855721	0.267216

α-Cl-TP

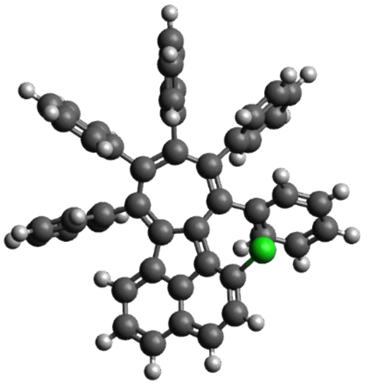

Table S108. Coordinates and energy for the optimized geometry of **α-Cl-TP**.

G = –2267.847031			
		**Coordinates / Å**	
**Atom**	**x**	**y**	**z**
C	-1.602418	0.548785	-0.138385
C	-1.322456	-0.825356	-0.295986
C	-0.694181	1.592652	0.166893
C	0.695387	1.544819	-0.014282
C	1.511184	0.379607	-0.291295
C	1.187873	-0.953792	0.076301
C	-0.113879	-1.500041	0.025011
C	2.838198	0.854887	-0.690648
C	3.961049	0.316435	-1.319335
C	5.101091	1.123903	-1.553457
C	5.118397	2.469729	-1.237133
H	5.965694	0.664328	-2.017903
H	6.010035	3.056740	-1.433564
C	1.591529	2.721523	-0.034097
C	1.451307	4.086608	0.204616
C	2.854905	2.256792	-0.467283
C	2.565774	4.938575	0.019543
H	0.520650	4.524892	0.530361
C	3.958322	3.092572	-0.709294
C	3.790703	4.472208	-0.428660
H	2.442077	5.996402	0.226457

H	4.621027	5.156606	-0.573566
C	2.306434	-1.767392	0.636439
C	2.624069	-3.072763	0.227825
C	3.048049	-1.187545	1.679847
C	3.646331	-3.778165	0.852466
H	2.088755	-3.527137	-0.594594
C	4.065657	-1.899586	2.311000
H	2.807813	-0.183207	2.014071
C	4.367397	-3.197897	1.899074
H	3.884618	-4.782356	0.516030
H	4.616751	-1.440266	3.125703
H	5.160604	-3.754762	2.388625
C	-0.278213	-2.944543	0.403070
C	-0.261917	-3.273267	1.762946
C	-0.468559	-3.950857	-0.549674
C	-0.439688	-4.595799	2.166608
H	-0.105526	-2.492210	2.500643
C	-0.637247	-5.273105	-0.144057
H	-0.480764	-3.700323	-1.604402
C	-0.626039	-5.598645	1.214014
H	-0.430057	-4.840668	3.224346
H	-0.779207	-6.048700	-0.890409
H	-0.761882	-6.629175	1.527775
C	-2.487607	-1.698235	-0.662230
C	-2.687058	-2.018047	-2.008705
C	-3.362848	-2.198752	0.306697
C	-3.755152	-2.833786	-2.383748
H	-2.005152	-1.631420	-2.760581
C	-4.425080	-3.017062	-0.069757
H	-3.205086	-1.954237	1.351758
C	-4.624422	-3.336261	-1.415224
H	-3.903809	-3.076730	-3.431579
H	-5.098277	-3.405611	0.688417
H	-5.453935	-3.973736	-1.705928
C	-3.050940	0.940589	-0.212874
C	-3.791426	1.125627	0.959688
C	-3.663043	1.133092	-1.455200
C	-5.132069	1.498919	0.888785
H	-3.316903	0.979398	1.924733
C	-5.002799	1.513531	-1.523562
H	-3.090257	0.988591	-2.365640
C	-5.740156	1.696314	-0.352609
H	-5.700380	1.636839	1.803656
H	-5.469071	1.665442	-2.492333
H	-6.783951	1.990466	-0.406812
C	-1.279750	2.891711	0.617039
C	-1.206899	3.218917	1.975546
C	-1.874400	3.782652	-0.283364

C	-1.731457	4.428211	2.431833
H	-0.737568	2.528388	2.669791
C	-2.388430	4.994120	0.174520
H	-1.928348	3.529414	-1.336515
C	-2.319596	5.318655	1.531808
H	-1.675971	4.674685	3.487668
H	-2.843115	5.684695	-0.528974
H	-2.724072	6.261892	1.885945
Cl	4.004690	-1.295380	-1.968674

α-Cl-DT

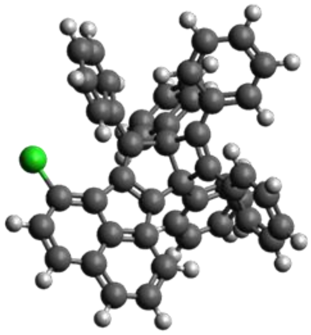

Table S109. Coordinates and energy for the optimized geometry of **α-Cl-DT**.

G = –2267.847031			
		**Coordinates / Å**	
**Atom**	**x**	**y**	**z**
C	4.853084	0.181624	-0.466591
C	5.430825	-1.107501	-0.586274
C	3.475563	0.222540	-0.211132
C	4.663157	-2.263848	-0.424609
C	2.684467	-0.955090	-0.019941
C	3.292025	-2.210150	-0.126250
C	1.372921	-0.497213	0.279378
C	2.707821	1.409134	-0.049655
C	1.333744	0.933046	0.204834
C	0.044985	-1.083928	0.550257
C	3.415589	2.599763	-0.064567
C	5.519845	1.436766	-0.550169
C	0.022279	1.382283	0.256840
C	-0.916684	0.211608	0.529054
C	-0.670583	-1.481360	-0.763523
C	-0.074954	-2.015671	1.731540
C	4.813791	2.602594	-0.339121
C	-0.461058	2.703506	-0.103178
C	-1.518921	-0.427672	-0.764396
C	-1.812430	0.389496	1.731296
C	-0.332354	-2.582588	-1.658347

C	0.897898	-2.035461	2.737106
C	-1.216261	-2.815724	1.871358
C	0.062837	3.307380	-1.265464
C	-1.511079	3.342002	0.589662
C	-2.674474	-0.066856	-1.583776
C	-1.229727	0.776580	2.947812
C	-3.182456	0.120095	1.687293
C	0.250984	-3.761229	-1.159713
C	-0.525398	-2.457790	-3.047192
H	1.782930	-1.412896	2.651465
C	0.736094	-2.846302	3.861230
C	-1.379299	-3.621510	2.995557
H	-1.977325	-2.803884	1.098242
H	0.848385	2.805152	-1.819325
C	-0.469475	4.500848	-1.737904
C	-2.006512	4.557937	0.133372
H	-1.914822	2.896048	1.488504
C	-3.080388	1.265776	-1.771843
C	-3.438902	-1.093057	-2.176073
H	-0.163507	0.977571	2.994039
C	-2.006246	0.898972	4.096552
C	-3.960773	0.244289	2.840128
H	-3.646995	-0.187136	0.758500
H	0.393745	-3.879217	-0.091288
C	0.618652	-4.790246	-2.025090
C	-0.154888	-3.486927	-3.906794
H	-0.954594	-1.544865	-3.445991
H	1.499863	-2.851514	4.633075
C	-0.402780	-3.640511	3.994340
H	-2.269187	-4.236435	3.090318
H	-0.079471	4.939869	-2.650358
C	-1.499321	5.132559	-1.036248
H	-2.799193	5.053655	0.684262
H	-2.519359	2.070860	-1.320501
C	-4.198787	1.562665	-2.547940
C	-4.554787	-0.791887	-2.949216
H	-3.156634	-2.127722	-2.013390
H	-1.541566	1.198054	5.031227
C	-3.377606	0.634195	4.045158
H	-5.024879	0.033326	2.791824
H	1.060926	-5.696797	-1.622969
C	0.417383	-4.657663	-3.399775
H	-0.305597	-3.373352	-4.976188
H	-0.529807	-4.270625	4.869427
H	-1.906948	6.070684	-1.399755
H	-4.491278	2.599314	-2.685890
C	-4.938487	0.538913	-3.141402
H	-5.132306	-1.596234	-3.394851

H	-3.984933	0.730060	4.940111
H	0.705811	-5.458952	-4.073327
H	-5.811360	0.773314	-3.743187
H	5.323848	3.558436	-0.365482
H	5.144172	-3.231170	-0.518322
H	2.725090	-3.122595	0.015279
H	6.585128	1.478766	-0.753059
H	6.491812	-1.198386	-0.798920
Cl	2.678363	4.130609	0.316883

α-Cl-DT′

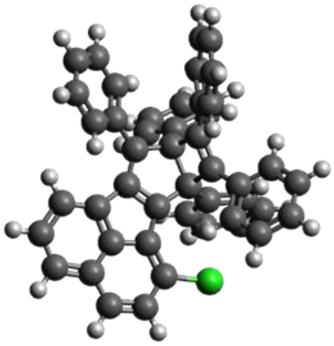

Table S110. Coordinates and energy for the optimized geometry of **α-Cl-DT′**.

G = –2267.847031			
		**Coordinates / Å**	
**Atom**	**x**	**y**	**z**
C	3.764162	3.157947	-0.304296
C	4.994088	2.453055	-0.343117
C	2.615151	2.377343	-0.117455
C	5.038868	1.071705	-0.185254
C	2.623718	0.949278	0.057693
C	3.871849	0.309860	0.023128
C	1.262960	0.571669	0.258880
C	1.306093	2.909557	-0.046082
C	0.440550	1.740348	0.176005
C	0.452764	-0.653115	0.476826
C	1.152203	4.282532	-0.137559
C	3.576178	4.563208	-0.418629
C	-0.902715	1.426235	0.300871
C	-1.061709	-0.071032	0.524075
C	0.010434	-1.300924	-0.864301
C	0.851405	-1.557439	1.615629
C	2.301448	5.094930	-0.328038
C	-2.001220	2.356117	0.101298
C	-1.264946	-0.859944	-0.804986
C	-1.866943	-0.473696	1.735346
C	0.819862	-1.996450	-1.867871

C	1.611698	-1.066721	2.683093
C	0.373920	-2.871863	1.670748
C	-1.905194	3.300046	-0.944886
C	-3.182956	2.311111	0.872570
C	-2.453773	-1.074131	-1.625252
C	-1.587592	0.136972	2.967269
C	-2.818915	-1.494615	1.682789
C	1.712017	-3.024643	-1.525053
C	0.722275	-1.602699	-3.215337
H	1.973661	-0.043112	2.665925
C	1.907263	-1.880294	3.776000
C	0.666592	-3.685363	2.763969
H	-0.235540	-3.256317	0.859626
H	-1.024700	3.307083	-1.576831
C	-2.959316	4.163762	-1.215528
C	-4.219705	3.200210	0.613729
H	-3.271990	1.601524	1.683713
C	-3.583047	-0.243891	-1.518209
C	-2.502577	-2.161176	-2.522152
H	-0.842681	0.925610	3.021046
C	-2.256949	-0.259510	4.121556
C	-3.490990	-1.891263	2.840806
H	-3.038035	-1.984696	0.741998
H	1.798739	-3.338673	-0.491831
C	2.478322	-3.649709	-2.507557
C	1.492158	-2.226619	-4.192775
H	0.043326	-0.799692	-3.484594
H	2.502677	-1.485318	4.593710
C	1.436912	-3.193283	3.819362
H	0.290258	-4.703570	2.790574
H	-2.882834	4.864341	-2.040675
C	-4.115743	4.122358	-0.431707
H	-5.114828	3.170504	1.226420
H	-3.581660	0.582208	-0.821120
C	-4.711289	-0.472419	-2.302793
C	-3.631218	-2.386396	-3.302331
H	-1.654794	-2.832248	-2.596181
H	-2.030023	0.222276	5.067817
C	-3.213764	-1.276437	4.061051
H	-4.230092	-2.684831	2.784677
H	3.160255	-4.447244	-2.228023
C	2.372486	-3.253839	-3.841853
H	1.410055	-1.907344	-5.227504
H	1.666486	-3.827970	4.669935
H	-4.935389	4.803508	-0.637709
H	-5.570010	0.185388	-2.207247
C	-4.739736	-1.539908	-3.200908
H	-3.650014	-3.229366	-3.986656

H	-3.736775	-1.586786	4.960567
H	2.973443	-3.740421	-4.604295
H	-5.620073	-1.718085	-3.811086
H	2.166764	6.168701	-0.403431
H	5.992716	0.558526	-0.213701
H	4.433964	5.211575	-0.567112
H	5.922893	2.995231	-0.492008
H	0.177600	4.751161	-0.063184
Cl	4.035066	-1.396891	0.242036

α-^{t}Bu-TP

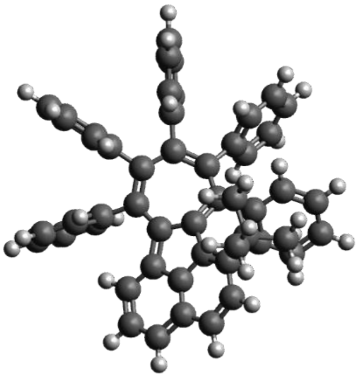

Table S111. Coordinates and energy for the optimized geometry of **α-^{t}Bu-TP**.

G = –1965.342396			
		**Coordinates / Å**	
**Atom**	**x**	**y**	**z**
C	1.847316	0.406943	0.137616
C	1.471418	-0.950751	0.098560
C	1.060285	1.517908	-0.258975
C	-0.334177	1.561874	-0.309322
C	-1.256638	0.525660	0.127649
C	-1.055215	-0.848345	-0.169541
C	0.203180	-1.494552	-0.246470
C	-2.548655	1.171665	0.403051
C	-3.708053	0.880422	1.143650
C	-4.822944	1.741976	0.910846
C	-4.776386	2.898590	0.157302
H	-5.762195	1.493401	1.390475
H	-5.670204	3.503238	0.036234
C	-1.151929	2.730403	-0.668378
C	-0.917041	3.937832	-1.321205
C	-2.466453	2.444010	-0.234921
C	-2.001082	4.818020	-1.538598
H	0.064090	4.215468	-1.679618
C	-3.543340	3.329502	-0.395828
C	-3.283518	4.538371	-1.088068

H	-1.814076	5.746399	-2.068261
H	-4.091628	5.241669	-1.266030
C	-2.276164	-1.621530	-0.578231
C	-2.604932	-2.886507	-0.068211
C	-3.078931	-1.079491	-1.595020
C	-3.695159	-3.592652	-0.568017
H	-2.011616	-3.325822	0.722581
C	-4.169622	-1.786839	-2.096167
H	-2.835590	-0.107883	-2.011949
C	-4.480346	-3.047942	-1.586174
H	-3.933409	-4.567824	-0.155111
H	-4.770542	-1.353087	-2.889404
H	-5.328121	-3.601815	-1.977581
C	0.212690	-2.922825	-0.708965
C	0.002482	-3.177881	-2.068618
C	0.437287	-3.991732	0.166169
C	0.018746	-4.487047	-2.548258
H	-0.180098	-2.350447	-2.747500
C	0.442269	-5.299315	-0.312898
H	0.605162	-3.799314	1.220344
C	0.235595	-5.550793	-1.671323
H	-0.141374	-4.673791	-3.605823
H	0.609984	-6.122099	0.375417
H	0.243896	-6.570716	-2.043710
C	2.582486	-1.930779	0.353018
C	2.969448	-2.198973	1.669488
C	3.243552	-2.564280	-0.704603
C	4.002558	-3.100390	1.926112
H	2.462419	-1.701459	2.490441
C	4.278876	-3.459480	-0.446778
H	2.939609	-2.362388	-1.726357
C	4.659574	-3.731890	0.869198
H	4.293732	-3.306042	2.951796
H	4.787301	-3.945466	-1.274032
H	5.465073	-4.432038	1.069267
C	3.259015	0.725057	0.536775
C	4.335908	0.552424	-0.338634
C	3.490637	1.219799	1.824737
C	5.629938	0.863626	0.073401
H	4.160732	0.173727	-1.339838
C	4.787515	1.524167	2.238347
H	2.655125	1.358932	2.504334
C	5.859946	1.346795	1.363539
H	6.459373	0.727213	-0.614018
H	4.957099	1.901075	3.242522
H	6.869799	1.584944	1.683807
C	1.792773	2.787571	-0.559724
C	2.435641	2.939480	-1.792767
C	1.815897	3.833144	0.369690
C	3.083963	4.136192	-2.099116

H	2.415662	2.128026	-2.513815
C	2.475600	5.022560	0.066337
H	1.313815	3.714229	1.324541
C	3.105498	5.178683	-1.170480
H	3.572768	4.251794	-3.061653
H	2.492838	5.828727	0.793335
H	3.613142	6.108382	-1.408774
C	-3.844023	-0.122136	2.298358
C	-4.310828	0.673738	3.548565
H	-5.290902	1.137031	3.413568
H	-4.382118	-0.011783	4.399546
H	-3.592291	1.460656	3.801831
C	-4.909706	-1.187817	1.963648
H	-4.650358	-1.747153	1.064649
H	-4.995486	-1.894554	2.796373
H	-5.892385	-0.730506	1.811218
C	-2.517932	-0.787746	2.701678
H	-2.146846	-1.485683	1.957124
H	-1.741688	-0.039396	2.893807
H	-2.672812	-1.353954	3.625596

α-^{t}Bu-DT

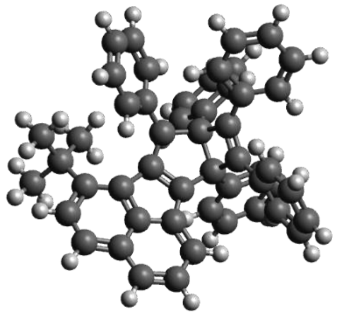

Table S112. Coordinates and energy for the optimized geometry of **α-^{t}Bu-DT**.

G = –1965.32847			
		Coordinates / Å	
**Atom**	**x**	**y**	**z**
C	-3.414152	3.015482	-1.164870
C	-2.942162	4.322132	-1.430623
C	-2.492386	2.109557	-0.620902
H	-3.614404	5.052437	-1.871973
C	-1.628413	4.694392	-1.127508
C	-1.147276	2.485234	-0.302153
H	-1.306390	5.708579	-1.337162
C	-0.720489	3.795622	-0.550980
C	-0.528223	1.317700	0.213859
C	-2.778447	0.755340	-0.274828
H	0.287712	4.112871	-0.313225
C	-1.452511	0.221242	0.135160
C	0.830892	0.896292	0.632815
C	-4.117757	0.351147	-0.286057

C	-4.725353	2.507470	-1.380702
C	-0.719976	-0.960348	0.100286
C	0.714460	-0.710004	0.533773
C	1.816800	0.772915	-0.548644
C	1.340919	1.469008	1.933103
C	-5.037467	1.242121	-0.940827
H	-5.483397	3.126687	-1.850081
C	-1.116012	-2.226890	-0.493311
C	1.736786	-0.571690	-0.636425
C	1.120420	-1.489288	1.761478
C	2.487165	1.830907	-1.295837
C	0.472332	2.086711	2.839563
C	2.688535	1.309663	2.281361
H	-6.059595	0.905021	-1.064106
C	-1.912401	-2.206491	-1.658348
C	-0.625126	-3.461792	-0.020547
C	2.461184	-1.560538	-1.444881
C	0.264904	-1.482058	2.873934
C	2.341256	-2.164953	1.845701
C	2.722763	3.087324	-0.709853
C	2.884147	1.620220	-2.629866
H	-0.576422	2.211227	2.587923
C	0.942164	2.541301	4.072336
C	3.156190	1.759031	3.513805
H	3.369466	0.830146	1.586055
H	-2.266072	-1.258203	-2.046438
C	-2.192306	-3.386215	-2.338497
C	-0.944061	-4.640162	-0.685250
H	-0.015684	-3.493172	0.872527
C	1.815195	-2.581124	-2.161146
C	3.865724	-1.475623	-1.506753
H	-0.681082	-0.951762	2.822201
C	0.625219	-2.138317	4.047574
C	2.700173	-2.824852	3.022471
H	3.012335	-2.182524	0.995831
H	2.439172	3.259465	0.322645
C	3.343593	4.102474	-1.435922
C	3.502022	2.637236	-3.350708
H	2.694341	0.660663	-3.098143
H	0.256076	3.019967	4.764714
C	2.284597	2.377188	4.413796
H	4.203076	1.627772	3.770518
H	-2.780952	-3.354780	-3.249680
C	-1.717230	-4.606465	-1.848930
H	-0.576834	-5.586743	-0.302463
H	0.736191	-2.645959	-2.157747
C	2.555486	-3.494436	-2.910857
C	4.600862	-2.390287	-2.255010
H	4.371962	-0.690045	-0.954097
H	-0.045336	-2.120997	4.901473

C	1.845977	-2.813641	4.125065
H	3.651017	-3.346867	3.074123
H	3.523378	5.065103	-0.966702
C	3.734886	3.881783	-2.757363
H	3.797771	2.461573	-4.380748
H	2.650934	2.729033	5.373501
H	-1.945847	-5.527551	-2.375699
H	2.038522	-4.274644	-3.461678
C	3.947386	-3.406713	-2.957426
H	5.683552	-2.313247	-2.286618
H	2.127908	-3.327056	5.039373
H	4.216856	4.673444	-3.323045
H	4.520412	-4.122912	-3.538620
C	-4.737711	-0.851536	0.443273
C	-5.903026	-0.321495	1.321617
H	-6.716230	0.112233	0.735137
H	-6.319623	-1.154715	1.896852
H	-5.547143	0.436932	2.027089
C	-3.765186	-1.556578	1.401149
H	-4.323010	-2.278943	2.005293
H	-2.989938	-2.110071	0.877664
H	-3.291391	-0.844991	2.085693
C	-5.301067	-1.869781	-0.571350
H	-4.505379	-2.309246	-1.177516
H	-5.804854	-2.680401	-0.033608
H	-6.030896	-1.407603	-1.243753

α-^{t}Bu-DT′

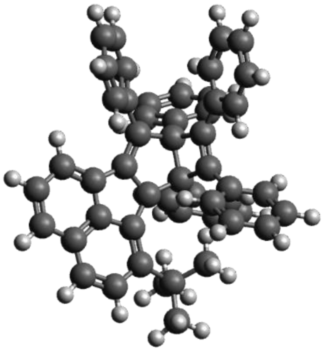

Table S113. Coordinates and energy for the optimized geometry of **α-^{t}Bu-DT′**.

G = –1965.32311			
		Coordinates / Å	
**Atom**	**x**	**y**	**z**
C	-5.721637	-0.919120	0.801406
C	-4.317870	-0.744022	0.178260
C	-3.389909	-1.675561	0.966893
C	-4.419953	-1.169501	-1.305543
C	-3.823870	0.710888	0.212628
C	-2.483656	1.188282	0.021563

C	-2.291315	2.630191	0.040124
C	-3.303980	3.588663	0.196374
C	-4.603781	3.074764	0.378476
C	-4.820157	1.706043	0.385842
C	-2.943896	4.962342	0.148289
C	-1.626485	5.324933	-0.063638
C	-0.610055	4.351623	-0.217997
C	-0.943076	3.008738	-0.143546
C	-0.222263	1.751489	-0.297036
C	1.090100	1.338922	-0.463268
C	2.277131	2.140990	-0.236310
C	3.486494	1.899879	-0.922590
C	4.618363	2.652690	-0.637396
C	4.579123	3.633463	0.357549
C	3.396881	3.864889	1.067220
C	2.254410	3.133922	0.769587
C	-1.150843	0.659785	-0.213851
C	-0.392629	-0.632837	-0.351593
C	0.283557	-1.186934	0.955117
C	-0.257143	-1.802806	2.175922
C	0.056375	-3.129499	2.506964
C	-0.391834	-3.681272	3.706287
C	-1.145904	-2.910389	4.593124
C	-1.450982	-1.583946	4.277104
C	-1.013551	-1.033442	3.074222
C	1.539934	-0.853496	0.608630
C	2.836054	-0.931533	1.281774
C	4.036339	-0.954894	0.551518
C	5.264257	-1.009005	1.207345
C	5.315757	-1.036380	2.601835
C	4.128550	-1.010026	3.338781
C	2.899460	-0.958381	2.687739
C	1.131584	-0.150763	-0.700389
C	1.684702	-0.625725	-2.019008
C	2.144311	-1.941363	-2.149401
C	2.547403	-2.429241	-3.390752
C	2.499786	-1.606375	-4.517973
C	2.044983	-0.292632	-4.395975
C	1.634061	0.192095	-3.155018
C	-0.925860	-1.636504	-1.345337
C	-1.253907	-1.189352	-2.633443
C	-1.625247	-2.092036	-3.626026
C	-1.687773	-3.457765	-3.340285
C	-1.370345	-3.909457	-2.059381
C	-0.981169	-3.004514	-1.070735
H	-5.937746	-1.990589	0.854191
H	-6.516170	-0.463995	0.202807
H	-5.771125	-0.518146	1.819305
H	-3.700039	-2.715323	0.821827
H	-3.435333	-1.450351	2.035263

H	-2.358070	-1.600867	0.652020
H	-4.759128	-2.209693	-1.361935
H	-3.465420	-1.096977	-1.821440
H	-5.146886	-0.541949	-1.832388
H	-5.444408	3.749502	0.512698
H	-5.839898	1.382309	0.528138
H	-3.712499	5.719400	0.269834
H	-1.358141	6.374841	-0.114482
H	0.411077	4.670430	-0.393436
H	3.523534	1.147888	-1.698969
H	5.536321	2.469678	-1.186296
H	5.470103	4.209901	0.586298
H	3.370459	4.607690	1.857836
H	1.351372	3.284639	1.348894
H	0.660419	-3.719743	1.824510
H	-0.146925	-4.710628	3.950045
H	-1.491536	-3.339493	5.528760
H	-2.030170	-0.978038	4.967439
H	-1.250937	-0.002708	2.828027
H	4.007648	-0.943195	-0.531090
H	6.181618	-1.028051	0.626527
H	6.273602	-1.075017	3.112040
H	4.160522	-1.023181	4.424265
H	1.982900	-0.921579	3.265490
H	2.171482	-2.587905	-1.278654
H	2.897913	-3.453269	-3.476674
H	2.816013	-1.986724	-5.484524
H	2.003352	0.355134	-5.266389
H	1.266053	1.210653	-3.072495
H	-1.208421	-0.129091	-2.861945
H	-1.867253	-1.728757	-4.620206
H	-1.980067	-4.163882	-4.111633
H	-1.417036	-4.969087	-1.826593
H	-0.730330	-3.368719	-0.081565

α-OMe-TP

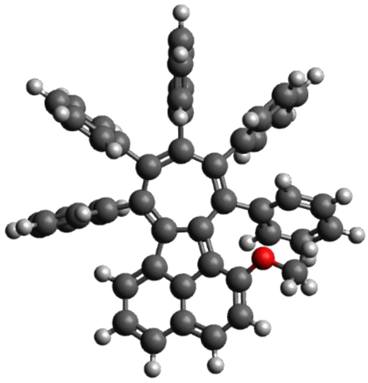

Table S114. Coordinates and energy for the optimized geometry of **α-OMe-TP**.

G = –1922.710864			
	**Coordinates / Å**		
**Atom**	**x**	**y**	**z**
C	-1.717508	0.271428	-0.161464
C	-1.214050	-1.038729	-0.234628
C	-1.009366	1.461891	0.154984
C	0.370624	1.659323	0.058488
C	1.394617	0.660244	-0.238916
C	1.295616	-0.729885	0.036526
C	0.105755	-1.485240	0.057411
C	2.618458	1.375490	-0.555134
C	3.865683	1.033400	-1.117355
C	4.865626	2.043981	-1.230884
C	4.620422	3.359035	-0.892743
H	5.839271	1.778348	-1.621615
H	5.409685	4.095785	-1.009080
C	1.044692	2.969756	0.113281
C	0.670690	4.281641	0.414061
C	2.381271	2.746633	-0.290815
C	1.627487	5.313633	0.311976
H	-0.327159	4.538665	0.734478
C	3.339255	3.765466	-0.429487
C	2.932611	5.078616	-0.101810
H	1.322837	6.324319	0.563119
H	3.641492	5.898200	-0.174282
C	2.564654	-1.421852	0.418272
C	3.045472	-2.554050	-0.254466
C	3.284320	-0.922287	1.511272
C	4.217306	-3.175783	0.162129
H	2.512641	-2.932424	-1.117967
C	4.456899	-1.549409	1.932374
H	2.917074	-0.048856	2.041062
C	4.926850	-2.676924	1.258752
H	4.581052	-4.048333	-0.372096
H	4.998034	-1.158099	2.788538
H	5.839144	-3.166741	1.585785
C	0.218878	-2.927158	0.467835
C	0.389276	-3.209852	1.827907
C	0.138866	-3.981370	-0.448347
C	0.472990	-4.530647	2.266897
H	0.458762	-2.393118	2.539886
C	0.231167	-5.300133	-0.009422
H	0.007142	-3.769401	-1.503482
C	0.395327	-5.579092	1.349158
H	0.600508	-4.738087	3.325119
H	0.172944	-6.110271	-0.730079
H	0.462469	-6.607916	1.689892
C	-2.225348	-2.115367	-0.511337

C	-2.461892	-2.507924	-1.831988
C	-2.920312	-2.737921	0.530122
C	-3.381574	-3.519812	-2.108836
H	-1.920931	-2.025781	-2.640887
C	-3.837050	-3.749363	0.252278
H	-2.732659	-2.437256	1.555723
C	-4.069113	-4.144042	-1.067415
H	-3.557517	-3.819785	-3.137581
H	-4.369886	-4.230191	1.067166
H	-4.783029	-4.933578	-1.282013
C	-3.200033	0.431179	-0.351223
C	-4.064662	0.468614	0.747535
C	-3.719360	0.566914	-1.642292
C	-5.434020	0.641811	0.555834
H	-3.663445	0.367829	1.750821
C	-5.089705	0.743791	-1.832670
H	-3.049452	0.538782	-2.496270
C	-5.949775	0.782263	-0.734315
H	-6.097531	0.668718	1.415022
H	-5.483117	0.851523	-2.839076
H	-7.016640	0.920250	-0.882246
C	-1.842967	2.641031	0.546993
C	-1.988634	2.934764	1.906844
C	-2.461356	3.451896	-0.410421
C	-2.749479	4.033612	2.306898
H	-1.502688	2.306019	2.646934
C	-3.216341	4.551952	-0.008028
H	-2.347969	3.222317	-1.464471
C	-3.361994	4.845095	1.350231
H	-2.859967	4.255820	3.363887
H	-3.690713	5.180472	-0.755467
H	-3.951973	5.701895	1.661129
O	4.040695	-0.205801	-1.572775
C	5.331310	-0.637311	-2.025547
H	5.631060	-0.087135	-2.921910
H	6.074975	-0.517386	-1.232950
H	5.210532	-1.694023	-2.255966

α-OMe-DT

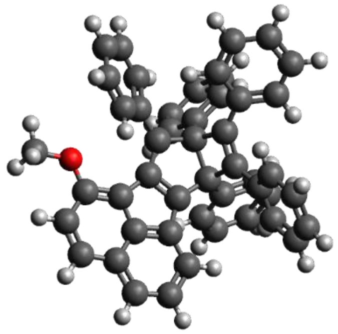

Table S115. Coordinates and energy for the optimized geometry of **α-OMe-DT**.

G = –1922.689245			
	Coordinates / Å		
**Atom**	**x**	**y**	**z**
C	4.682300	-1.296226	-0.361870
C	4.869658	-2.692415	-0.443774
C	3.366723	-0.854322	-0.142449
H	5.865263	-3.089586	-0.620092
C	3.794175	-3.581069	-0.293302
C	2.259401	-1.752340	0.027252
H	3.982812	-4.646990	-0.359178
C	2.490302	-3.133958	-0.049076
C	1.131826	-0.930994	0.272093
C	2.973553	0.496402	-0.027771
H	1.682268	-3.845037	0.077654
C	1.523237	0.449908	0.203243
C	-0.321345	-1.087885	0.501973
C	3.978437	1.466152	-0.065241
C	5.674659	-0.276857	-0.457687
C	0.413359	1.277391	0.279735
C	-0.840468	0.443629	0.526173
C	-1.115047	-1.198414	-0.821439
C	-0.749332	-1.973231	1.648112
C	5.328626	1.048588	-0.308091
H	6.712011	-0.544106	-0.633124
C	0.379385	2.701912	-0.005736
C	-1.604329	0.061819	-0.779770
C	-1.641964	0.839296	1.742776
C	-1.126448	-2.317717	-1.756432
C	0.139139	-2.295966	2.680123
C	-2.076110	-2.415267	1.731620
H	6.105907	1.800110	-0.363019
C	1.138623	3.187230	-1.091432
C	-0.454783	3.596851	0.698133
C	-2.588362	0.791545	-1.578722
C	-0.970057	1.020066	2.961435
C	-3.033547	0.958412	1.709566
C	-0.867755	-3.626036	-1.309972
C	-1.330888	-2.101506	-3.132590
H	1.167394	-1.951591	2.638900
C	-0.288005	-3.052043	3.772325
C	-2.503556	-3.166864	2.823730
H	-2.774490	-2.167618	0.939175
H	1.746382	2.497412	-1.663934
C	1.056532	4.521823	-1.469020
C	-0.503719	4.938032	0.335664
H	-1.040423	3.243600	1.535688
C	-2.534628	2.185269	-1.751718
C	-3.657319	0.081976	-2.162677

H	0.111156	0.923461	2.998916
C	-1.677343	1.321147	4.121915
C	-3.742353	1.262348	2.873912
H	-3.569234	0.814693	0.779187
H	-0.722311	-3.811723	-0.251709
C	-0.827845	-4.688284	-2.211339
C	-1.287929	-3.164587	-4.028798
H	-1.509000	-1.094508	-3.494005
H	0.414494	-3.294259	4.564281
C	-1.610444	-3.489205	3.848180
H	-3.535129	-3.502476	2.873064
H	1.623688	4.875090	-2.324233
C	0.242763	5.402651	-0.751197
H	-1.133018	5.621576	0.896465
H	-1.728785	2.753569	-1.310024
C	-3.506207	2.843873	-2.503001
C	-4.625408	0.743496	-2.910813
H	-3.727435	-0.990290	-2.012735
H	-1.143572	1.457006	5.057765
C	-3.068913	1.444797	4.081079
H	-4.823664	1.353986	2.832858
H	-0.633689	-5.693075	-1.848079
C	-1.037937	-4.462542	-3.572521
H	-1.442665	-2.980073	-5.087754
H	-1.943758	-4.077060	4.698152
H	0.186587	6.447426	-1.040914
H	-3.442868	3.920611	-2.629548
C	-4.552949	2.128725	-3.086335
H	-5.443093	0.179778	-3.349967
H	-3.622040	1.680517	4.985295
H	-1.005005	-5.290192	-4.274638
H	-5.310566	2.645199	-3.668028
O	3.631603	2.740377	0.147909
C	4.605173	3.787098	0.038139
H	5.375742	3.683371	0.807611
H	5.060675	3.792838	-0.956476
H	4.046259	4.709084	0.192740

α-OMe-DT′

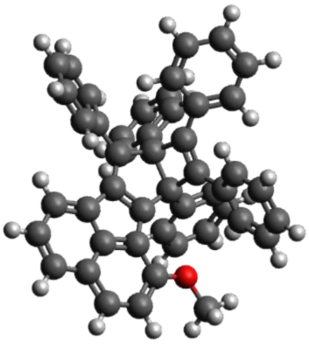

Table S116. Coordinates and energy for the optimized geometry of **α-OMe-DT′**.

G = –1922.694643			
	Coordinates / Å		
Atom	x	y	z
C	3.631989	3.272189	-0.316525
C	4.876974	2.578041	-0.348062
C	2.500616	2.473534	-0.123255
H	5.794507	3.138281	-0.503089
C	4.958762	1.204896	-0.179299
C	2.541648	1.048392	0.060461
C	3.802599	0.406407	0.040804
C	1.210264	0.633971	0.254790
C	1.178446	2.973088	-0.057014
C	0.343133	1.781096	0.171280
C	0.440713	-0.617855	0.457219
C	0.996733	4.344383	-0.165788
C	3.417086	4.668436	-0.444555
C	-0.981888	1.418841	0.297116
C	-1.091980	-0.087948	0.519130
C	0.014906	-1.265636	-0.884202
C	0.883232	-1.534364	1.571373
C	2.127693	5.175800	-0.361910
H	4.260086	5.335134	-0.599029
C	-2.120287	2.307376	0.103461
C	-1.280892	-0.885040	-0.804449
C	-1.870158	-0.511164	1.740528
C	0.863103	-1.923162	-1.875648
C	1.672984	-1.056423	2.622798
C	0.435755	-2.860512	1.608790
C	-2.064506	3.258932	-0.936880
C	-3.297534	2.211079	0.874472
C	-2.475593	-1.169481	-1.597038
C	-1.581353	0.098886	2.970633
C	-2.808866	-1.544914	1.698971
C	1.989695	-2.667359	-1.486356
C	0.592151	-1.773247	-3.249055
H	2.018871	-0.027584	2.615419
C	2.017804	-1.889455	3.686624
C	0.777091	-3.693240	2.672887
H	-0.183353	-3.240379	0.802785
H	-1.184736	3.305413	-1.568459
C	-3.151692	4.084117	-1.200386
C	-4.371250	3.058195	0.620465
H	-3.359434	1.490877	1.678775
C	-3.590473	-0.313613	-1.594915
C	-2.544370	-2.359370	-2.349741
H	-0.846786	0.897844	3.014624

C	-2.227347	-0.310763	4.133613
C	-3.458131	-1.954803	2.865654
H	-3.036078	-2.034030	0.759579
H	2.211371	-2.786868	-0.433763
C	2.813922	-3.255922	-2.443768
C	1.419823	-2.359639	-4.201581
H	-0.262077	-1.182167	-3.562203
H	2.633435	-1.502774	4.493494
C	1.570874	-3.211082	3.715599
H	0.422267	-4.719539	2.686008
H	-3.102550	4.793553	-2.020268
C	-4.305627	3.991431	-0.417570
H	-5.265114	2.985755	1.231752
H	-3.571180	0.596336	-1.011628
C	-4.724503	-0.622189	-2.343794
C	-3.678585	-2.664539	-3.094504
H	-1.706207	-3.047445	-2.336190
H	-1.992807	0.170733	5.078289
C	-3.171216	-1.340543	4.083977
H	-4.187584	-2.757937	2.817749
H	3.676251	-3.835113	-2.126454
C	2.533251	-3.105457	-3.802881
H	1.201134	-2.228279	-5.257229
H	1.838424	-3.860547	4.543790
H	-5.152321	4.640230	-0.619161
H	-5.572234	0.056548	-2.332465
C	-4.773476	-1.794714	-3.098708
H	-3.712967	-3.587288	-3.666130
H	-3.676571	-1.661045	4.990106
H	3.177880	-3.562797	-4.547605
H	-5.659102	-2.034959	-3.679320
H	0.012078	4.793842	-0.102179
H	1.972604	6.246022	-0.449870
H	5.932853	0.734132	-0.207543
O	3.823531	-0.902867	0.242911
C	5.068217	-1.623542	0.223494
H	5.536799	-1.543590	-0.760606
H	5.737603	-1.250103	1.002479
H	4.798247	-2.657970	0.427509

α,β′-Me$_2$-TP

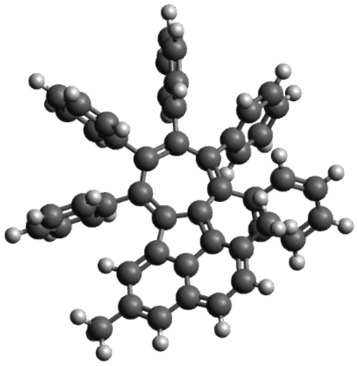

Table S117. Coordinates and energy for the optimized geometry of **α,β′-Me$_2$-TP**.

	G = –1886.81212		
	**Coordinates / Å**		
**Atom**	**x**	**y**	**z**
C	-0.523327	1.548123	-0.203440
C	-1.569395	0.601934	-0.134871
C	0.851068	1.325166	0.050149
C	1.522456	0.096802	0.033809
C	0.993092	-1.196962	-0.382589
C	-0.335533	-1.630074	-0.171402
C	-1.473165	-0.802254	0.035816
C	2.148032	-2.052277	-0.709999
C	2.356413	-3.242128	-1.425682
C	3.684469	-3.753380	-1.456044
C	4.770514	-3.124229	-0.875274
H	3.845261	-4.684979	-1.990569
H	5.757176	-3.574076	-0.934714
C	2.964686	-0.077408	0.257831
C	3.983198	0.700959	0.799869
C	3.289395	-1.364573	-0.223118
C	5.312202	0.205799	0.841488
C	4.602032	-1.860797	-0.253910
C	5.613633	-1.043433	0.308483
H	6.639931	-1.400467	0.321563
C	-0.551804	-3.103868	-0.023156
C	-1.487170	-3.802239	-0.799645
C	0.161216	-3.792773	0.967024
C	-1.708804	-5.158830	-0.586606
H	-2.029065	-3.285673	-1.582854
C	-0.064656	-5.151382	1.183973
H	0.881941	-3.260371	1.579171
C	-1.001340	-5.837471	0.409406
H	-2.430916	-5.687485	-1.200978
H	0.488107	-5.670249	1.961056
H	-1.178657	-6.895118	0.578993
C	-2.735604	-1.495002	0.465414

C	-2.809608	-1.977631	1.777129
C	-3.833153	-1.654511	-0.388116
C	-3.968194	-2.606339	2.231085
H	-1.957705	-1.861161	2.440070
C	-4.985471	-2.292774	0.063197
H	-3.784719	-1.280231	-1.404852
C	-5.058175	-2.767779	1.374668
H	-4.016183	-2.971447	3.252570
H	-5.828351	-2.416679	-0.609979
H	-5.959313	-3.261028	1.726329
C	-2.956525	1.182024	-0.167041
C	-3.523751	1.563559	-1.386495
C	-3.679798	1.358493	1.017280
C	-4.807041	2.107916	-1.421985
H	-2.960664	1.434380	-2.305350
C	-4.959301	1.908035	0.980600
H	-3.242556	1.057616	1.963735
C	-5.527320	2.282007	-0.239220
H	-5.241627	2.397033	-2.374166
H	-5.512933	2.042533	1.904970
H	-6.525819	2.707748	-0.267263
C	-0.908534	2.974699	-0.470627
C	-1.448762	3.795168	0.524956
C	-0.711823	3.488929	-1.756442
C	-1.797300	5.112027	0.233366
H	-1.598020	3.401837	1.524926
C	-1.066951	4.805959	-2.048366
H	-0.287526	2.854812	-2.529320
C	-1.611207	5.619977	-1.054472
H	-2.215727	5.741482	1.012979
H	-0.916480	5.193480	-3.051550
H	-1.887267	6.645559	-1.280396
C	1.684288	2.545253	0.295634
C	1.669011	3.152491	1.555282
C	2.507435	3.057341	-0.712981
C	2.489550	4.251545	1.810547
H	1.027888	2.754732	2.335912
C	3.317288	4.162712	-0.459379
H	2.519128	2.583035	-1.689171
C	3.316241	4.756698	0.804925
H	2.482088	4.711635	2.793899
H	3.953252	4.555794	-1.246630
H	3.954081	5.612387	1.004307
C	1.324644	-3.971514	-2.237116
H	0.875686	-4.790705	-1.666467
H	0.521941	-3.312852	-2.568369
H	1.799451	-4.408353	-3.121127
H	3.795760	1.684460	1.206958

C	6.384995	1.069950	1.457501
H	6.362063	2.081622	1.037273
H	6.236364	1.166713	2.539858
H	7.380537	0.650393	1.290420

α,β′-Me₂-DT

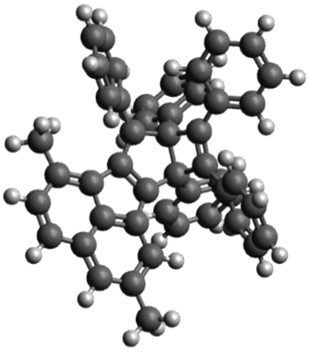

Table S118. Coordinates and energy for the optimized geometry of **α,β′-Me_2-DT**.

G = –1886.79523			
		Coordinates / Å	
**Atom**	**x**	**y**	**z**
C	4.086364	2.474099	-0.121571
C	5.183769	1.589109	-0.248451
C	2.831577	1.883752	0.074217
H	6.175828	2.001056	-0.414366
C	5.041111	0.194441	-0.159400
C	2.660197	0.467070	0.193693
C	3.773515	-0.370986	0.080736
C	1.276302	0.266790	0.427457
C	1.613363	2.594555	0.223292
C	0.596655	1.533099	0.390201
C	0.341378	-0.868174	0.565253
C	1.660093	3.984248	0.267687
C	4.106322	3.898244	-0.149758
C	-0.770590	1.338045	0.358404
C	-1.100322	-0.143674	0.516636
C	-0.037377	-1.435385	-0.825057
C	0.589306	-1.845941	1.686847
C	2.935678	4.599741	0.052846
H	5.041764	4.427145	-0.303681
C	-1.775212	2.334491	0.004712
C	-1.275173	-0.886748	-0.846641
C	-2.043680	-0.463298	1.650594
C	0.841092	-2.157142	-1.740376
C	1.415529	-1.509095	2.764667
C	-0.072942	-3.080348	1.695685
H	2.972056	5.685097	0.067599
C	-1.556955	3.106231	-1.153671

C	-2.986313	2.477267	0.709624
C	-2.421043	-0.993265	-1.744674
C	-1.786383	0.089361	2.914645
C	-3.115060	-1.347324	1.501161
C	1.841076	-3.015378	-1.248419
C	0.760925	-1.939036	-3.128707
H	1.927835	-0.552265	2.777950
C	1.582246	-2.393218	3.831364
C	0.090839	-3.961069	2.762254
H	-0.717119	-3.348956	0.864955
H	-0.634857	2.979491	-1.710936
C	-2.537278	3.980895	-1.609692
C	-3.945122	3.383399	0.266489
H	-3.161544	1.895987	1.605564
C	-3.463182	-0.049038	-1.733517
C	-2.522875	-2.093704	-2.620693
H	-0.948632	0.768326	3.044878
C	-2.593112	-0.227980	4.004066
C	-3.924190	-1.665023	2.594038
H	-3.321686	-1.792060	0.535554
H	1.912843	-3.199032	-0.182178
C	2.724250	-3.648286	-2.121496
C	1.645993	-2.571789	-3.996232
H	0.012025	-1.258066	-3.519128
H	2.227476	-2.118085	4.660372
C	0.920081	-3.620925	3.833839
H	-0.428455	-4.914691	2.755118
H	-2.370368	4.549143	-2.519154
C	-3.730576	4.126029	-0.897957
H	-4.867160	3.502951	0.826355
H	-3.426195	0.787813	-1.050786
C	-4.549880	-0.179961	-2.594989
C	-3.610035	-2.221132	-3.478090
H	-1.749663	-2.853744	-2.615803
H	-2.381344	0.207503	4.975974
C	-3.668125	-1.106871	3.845833
H	-4.754500	-2.352404	2.462388
H	3.485650	-4.313348	-1.724994
C	2.630283	-3.429873	-3.496910
H	1.574066	-2.388234	-5.064120
H	1.048809	-4.309071	4.663866
H	-4.491324	4.816162	-1.249131
H	-5.339139	0.565599	-2.574183
C	-4.626658	-1.260977	-3.474254
H	-3.669851	-3.076192	-4.144784
H	-4.298625	-1.355173	4.694272
H	3.320307	-3.921397	-4.176037
H	-5.475216	-1.361998	-4.144160

C	0.477968	4.864001	0.563526
H	0.009307	5.220048	-0.361372
H	-0.287893	4.338066	1.135934
H	0.796265	5.745202	1.128631
H	3.671995	-1.446922	0.172099
C	6.238186	-0.710846	-0.313980
H	6.135464	-1.338947	-1.206520
H	7.163610	-0.137284	-0.407711
H	6.336178	-1.381999	0.546174

α,β′-Me$_2$-DT′

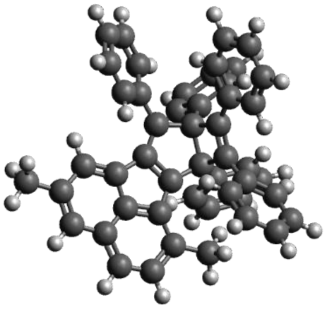

Table S119. Coordinates and energy for the optimized geometry of **α,β′-Me$_2$-DT′**.

G = – 1886.798695			
	Coordinates / Å		
**Atom**	**x**	**y**	**z**
C	-4.492769	1.492876	-0.185414
C	-5.403438	0.412026	-0.050576
C	-3.145017	1.131753	-0.280991
H	-6.467974	0.613279	0.030542
C	-4.957534	-0.906277	-0.016731
C	-2.669178	-0.225502	-0.275777
C	-3.596104	-1.277960	-0.121027
C	-1.258438	-0.130229	-0.439656
C	-2.095499	2.072393	-0.401588
C	-0.873459	1.253768	-0.469322
C	-0.077886	-1.024965	-0.500828
C	-2.419506	3.414740	-0.459491
C	-4.794541	2.881128	-0.236502
C	0.500572	1.395710	-0.463533
C	1.157626	0.020826	-0.475248
C	0.384389	-1.397762	0.933109
C	-0.060829	-2.090402	-1.566008
C	-3.784833	3.823535	-0.377915
H	-5.828670	3.206419	-0.168576
C	1.220379	2.649479	-0.286334
C	1.409186	-0.523307	0.966567
C	2.208840	-0.211443	-1.531411

C	-0.325709	-2.264723	1.884381
C	-0.906697	-2.011220	-2.678763
C	0.877132	-3.128147	-1.500095
C	0.721209	3.590050	0.639315
C	2.426159	2.932661	-0.961068
C	2.387814	-0.143950	1.978115
C	1.914176	0.119850	-2.862576
C	3.425609	-0.832361	-1.239470
C	-0.292755	-3.663740	1.785457
C	-1.136714	-1.666492	2.863861
H	-1.629973	-1.205203	-2.753674
C	-0.828552	-2.961179	-3.697290
C	0.959890	-4.073935	-2.519433
H	1.558841	-3.180094	-0.658208
H	-0.179812	3.361883	1.196752
C	1.410730	4.771043	0.888746
C	3.095617	4.128437	-0.724814
H	2.818577	2.230562	-1.684376
C	3.336583	0.864017	1.733578
C	2.395210	-0.788111	3.232072
H	0.965002	0.590116	-3.102360
C	2.825607	-0.154166	-3.878281
C	4.340673	-1.106181	-2.258128
H	3.662171	-1.107011	-0.218186
H	0.322208	-4.136128	1.028095
C	-1.056287	-4.447270	2.649747
C	-1.902870	-2.452897	3.722512
H	-1.167117	-0.583568	2.937905
H	-1.494564	-2.887461	-4.551783
C	0.104337	-3.995718	-3.620517
H	1.694642	-4.870732	-2.453962
H	1.027387	5.472976	1.622371
C	2.596422	5.046764	0.202929
H	4.012648	4.343106	-1.264140
H	3.359737	1.360824	0.772761
C	4.252683	1.231025	2.716460
C	3.312560	-0.419015	4.209749
H	1.678020	-1.575428	3.434726
H	2.583495	0.107458	-4.903956
C	4.044853	-0.768221	-3.577950
H	5.283555	-1.587097	-2.015519
H	-1.022049	-5.529247	2.565118
C	-1.866455	-3.845448	3.615172
H	-2.529528	-1.978981	4.472086
H	0.166853	-4.734507	-4.413793
H	3.131122	5.972187	0.392897
H	4.974218	2.015512	2.508891
C	4.243262	0.594190	3.958245

H	3.303507	-0.923454	5.171394
H	4.756354	-0.982835	-4.369587
H	-2.465097	-4.458343	4.282297
H	4.957213	0.880830	4.724560
H	-1.653657	4.175955	-0.567983
C	-4.102374	5.296802	-0.443940
H	-3.607548	5.842183	0.368218
H	-3.749574	5.732021	-1.386279
H	-5.177957	5.475870	-0.368287
H	-5.691571	-1.698097	0.096222
C	-3.210727	-2.721085	-0.015084
H	-3.092915	-2.993455	1.041275
H	-3.991207	-3.361720	-0.435560
H	-2.266435	-2.936027	-0.515401

α-Me-TP

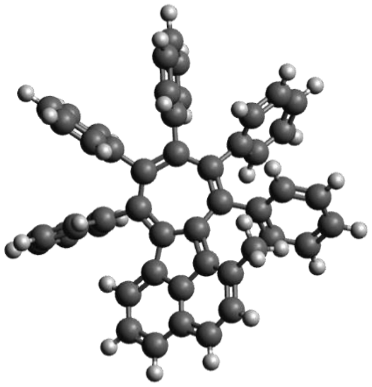

Table S120. Coordinates and energy for the optimized geometry of **α-Me-TP**.

G = –1847.531802			
		**Coordinates / Å**	
**Atom**	**x**	**y**	**z**
C	1.564772	-0.431072	-0.153345
C	1.219227	0.938480	-0.117679
C	0.723981	-1.534064	0.129041
C	-0.676596	-1.548121	0.132636
C	-1.572922	-0.488055	-0.312639
C	-1.331099	0.894839	-0.153063
C	-0.065884	1.517138	0.032969
C	-2.875257	-1.115550	-0.600597
C	-4.028504	-0.765434	-1.319346
C	-5.114581	-1.686068	-1.290373
C	-5.073030	-2.910065	-0.650175
H	-6.017573	-1.405660	-1.824545
H	-5.941362	-3.562125	-0.662338
C	-1.507041	-2.727196	0.414716
C	-1.295634	-3.969099	1.013414
C	-2.803991	-2.424905	-0.057132
C	-2.374872	-4.873968	1.111965

H	-0.335635	-4.258179	1.416030
C	-3.872062	-3.338004	-0.028201
C	-3.630734	-4.587192	0.594538
H	-2.203607	-5.830883	1.593877
H	-4.431624	-5.317203	0.665435
C	-2.530563	1.782878	-0.035561
C	-2.710107	2.906105	-0.855031
C	-3.471224	1.514199	0.967663
C	-3.804609	3.744966	-0.672364
H	-2.000037	3.113533	-1.646589
C	-4.565065	2.358203	1.154471
H	-3.337396	0.651717	1.612467
C	-4.733949	3.476262	0.336196
H	-3.934377	4.606508	-1.319905
H	-5.281014	2.143283	1.941753
H	-5.585083	4.134438	0.481918
C	-0.085985	2.971994	0.409284
C	-0.472263	3.313884	1.710270
C	0.282906	3.982493	-0.485810
C	-0.482448	4.648738	2.112812
H	-0.765835	2.533535	2.405670
C	0.261937	5.316003	-0.085720
H	0.584723	3.724342	-1.494983
C	-0.117307	5.653297	1.215575
H	-0.778206	4.901625	3.126537
H	0.544213	6.091947	-0.790798
H	-0.128537	6.693246	1.527600
C	2.380576	1.892138	-0.177890
C	2.985980	2.176056	-1.405744
C	2.870198	2.488711	0.988775
C	4.066090	3.055701	-1.467006
H	2.612350	1.707925	-2.310920
C	3.953322	3.362546	0.926408
H	2.397487	2.274226	1.941698
C	4.552785	3.650146	-0.301701
H	4.527199	3.273882	-2.425574
H	4.327719	3.819713	1.837384
H	5.395262	4.333488	-0.349760
C	3.003986	-0.764596	-0.423181
C	3.994652	-0.622174	0.553818
C	3.352538	-1.232179	-1.694676
C	5.319239	-0.936337	0.258597
H	3.728947	-0.260837	1.541316
C	4.680463	-1.539292	-1.991099
H	2.584185	-1.347403	-2.453456
C	5.666739	-1.392043	-1.015243
H	6.080921	-0.823219	1.024174
H	4.941208	-1.894615	-2.983534

H	6.700645	-1.631977	-1.244442
C	1.410900	-2.840989	0.379723
C	2.006500	-3.090766	1.620091
C	1.436417	-3.825523	-0.614137
C	2.608083	-4.324388	1.869952
H	1.985831	-2.326466	2.390501
C	2.049917	-5.051989	-0.366929
H	0.971833	-3.629895	-1.575479
C	2.630813	-5.306384	0.877513
H	3.059439	-4.516092	2.838595
H	2.068962	-5.810393	-1.143568
H	3.101665	-6.265135	1.072169
C	-4.185270	0.447518	-2.190548
H	-4.700657	1.254198	-1.659482
H	-3.226463	0.831332	-2.538323
H	-4.791312	0.190080	-3.064844

α-Me-DT

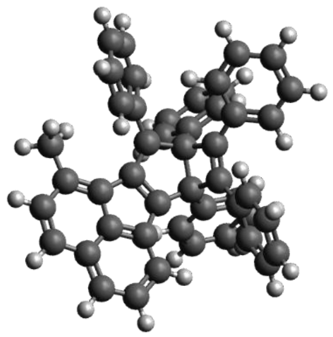

Table S121. Coordinates and energy for the optimized geometry of **α-Me-DT**.

G = –1847.515675			
		**Coordinates / Å**	
**Atom**	**x**	**y**	**z**
C	4.546097	1.868373	-0.250997
C	5.517672	0.848201	-0.386374
C	3.227233	1.444343	-0.032428
H	6.553980	1.118570	-0.567816
C	5.172673	-0.504988	-0.281160
C	2.866429	0.063377	0.097799
H	5.949944	-1.253720	-0.387533
C	3.857489	-0.918707	-0.027189
C	1.473480	0.052498	0.360564
C	2.116991	2.312380	0.135386
H	3.620444	-1.971900	0.068134
C	0.970946	1.398489	0.331261
C	0.398026	-0.944697	0.537368
C	2.347997	3.683808	0.172252
C	4.752836	3.277510	-0.286629
C	-0.410914	1.390575	0.343934

C	-0.932492	-0.031137	0.528911
C	-0.103992	-1.464783	-0.832160
C	0.546519	-1.939078	1.662261
C	3.689032	4.127107	-0.068008
H	5.747396	3.677455	-0.458044
C	-1.283039	2.510622	0.012473
C	-1.255766	-0.753070	-0.817975
C	-1.868332	-0.210929	1.699405
C	0.629151	-2.310688	-1.768500
C	1.438559	-1.706360	2.714865
C	-0.275105	-3.072948	1.703610
H	3.867219	5.198267	-0.058458
C	-0.998313	3.241657	-1.157977
C	-2.443147	2.815813	0.750904
C	-2.437607	-0.711907	-1.674296
C	-1.483840	0.298325	2.949295
C	-3.062212	-0.928801	1.595711
C	1.522601	-3.291988	-1.302495
C	0.516239	-2.103225	-3.156230
H	2.074510	-0.826789	2.704207
C	1.513906	-2.595427	3.787794
C	-0.202429	-3.958033	2.776648
H	-0.972552	-3.259614	0.893708
H	-0.118085	2.990332	-1.740197
C	-1.867016	4.236588	-1.592799
C	-3.285550	3.839659	0.328384
H	-2.667800	2.266359	1.655816
C	-3.332357	0.372836	-1.655001
C	-2.728366	-1.805786	-2.515457
H	-0.550356	0.845769	3.043238
C	-2.285126	0.101969	4.070733
C	-3.866046	-1.124548	2.720743
H	-3.368511	-1.338443	0.641086
H	1.616654	-3.469338	-0.236872
C	2.268007	-4.054516	-2.200099
C	1.264184	-2.865480	-4.048160
H	-0.148859	-1.330589	-3.527363
H	2.211998	-2.401804	4.596742
C	0.693492	-3.722993	3.822406
H	-0.845045	-4.833151	2.794618
H	-1.653426	4.773603	-2.511402
C	-3.008858	4.541959	-0.847958
H	-4.166509	4.083234	0.913542
H	-3.150412	1.211455	-0.998359
C	-4.457913	0.380177	-2.475598
C	-3.853796	-1.794906	-3.331927
H	-2.071988	-2.668750	-2.515465
H	-1.974062	0.501241	5.031423

C	-3.482583	-0.609931	3.958624
H	-4.792351	-1.683153	2.625183
H	2.947396	-4.813453	-1.823748
C	2.141576	-3.845386	-3.574330
H	1.169384	-2.689222	-5.115502
H	0.751051	-4.414845	4.657312
H	-3.681514	5.325369	-1.182772
H	-5.130855	1.232048	-2.449182
C	-4.721603	-0.698443	-3.320856
H	-4.060232	-2.647466	-3.972003
H	-4.109086	-0.763629	4.832056
H	2.724488	-4.438414	-4.272507
H	-5.600318	-0.691557	-3.958694
C	1.298635	4.713269	0.483948
H	0.867370	5.126582	-0.435447
H	0.479114	4.295339	1.070554
H	1.740801	5.545554	1.039685

α-Me-DT′

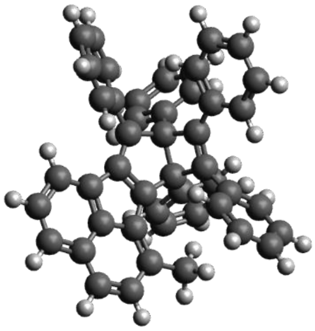

Table S122. Coordinates and energy for the optimized geometry of **α-Me-DT′**.

G = –1847.519046			
		**Coordinates / Å**	
**Atom**	**x**	**y**	**z**
C	-3.658360	3.205146	-0.419184
C	-4.906742	2.548664	-0.252748
C	-2.536679	2.367598	-0.456717
H	-5.820428	3.134937	-0.214375
C	-4.982461	1.164910	-0.133983
C	-2.601763	0.932772	-0.364014
C	-3.853916	0.311009	-0.178000
C	-1.256750	0.484507	-0.492167
C	-1.209594	2.837754	-0.597397
C	-0.382794	1.620183	-0.585734
C	-0.496417	-0.787826	-0.469398
C	-1.006650	4.200516	-0.737527
C	-3.423915	4.600847	-0.553253
C	0.944963	1.239603	-0.548111
C	1.040319	-0.279011	-0.467925

C	-0.214045	-1.214544	0.996391
C	-0.873429	-1.848207	-1.471280
C	-2.129757	5.068418	-0.714517
H	-4.259033	5.294368	-0.532688
C	2.079703	2.142164	-0.413708
C	1.063470	-0.786074	1.008616
C	1.933390	-0.950356	-1.480754
C	-1.202786	-1.687800	1.975827
C	-1.612927	-1.527797	-2.616267
C	-0.401893	-3.157316	-1.313610
C	1.961673	3.251731	0.449952
C	3.309108	1.915752	-1.067007
C	2.108265	-0.734263	2.023805
C	1.797369	-0.607454	-2.834451
C	2.820799	-1.968371	-1.123571
C	-1.696242	-3.000911	1.963508
C	-1.736310	-0.764373	2.891066
H	-1.973995	-0.514577	-2.762890
C	-1.890716	-2.501260	-3.575866
C	-0.674499	-4.128958	-2.273923
H	0.199340	-3.408708	-0.446728
H	1.036862	3.407633	0.993414
C	3.041489	4.101431	0.660486
C	4.375537	2.786423	-0.871612
H	3.415462	1.078292	-1.743423
C	3.370065	-0.181626	1.744145
C	1.864325	-1.239882	3.316967
H	1.100056	0.173200	-3.124468
C	2.544949	-1.262787	-3.808711
C	3.571729	-2.625021	-2.100762
H	2.927059	-2.254177	-0.083541
H	-1.298796	-3.719438	1.255937
C	-2.703344	-3.381112	2.849585
C	-2.746791	-1.146588	3.771694
H	-1.358158	0.253502	2.897919
H	-2.468793	-2.236848	-4.456243
C	-1.423224	-3.804975	-3.407349
H	-0.299653	-5.138766	-2.136914
H	2.943439	4.935691	1.347652
C	4.249318	3.875332	-0.004596
H	5.310301	2.612637	-1.394937
H	3.584691	0.198048	0.754105
C	4.352292	-0.118247	2.729613
C	2.848468	-1.175334	4.297231
H	0.900719	-1.679570	3.548130
H	2.428920	-0.986953	-4.852484
C	3.437226	-2.274864	-3.443553
H	4.259494	-3.412591	-1.807809

H	-3.077015	-4.400458	2.832251
C	-3.234763	-2.455603	3.750524
H	-3.154301	-0.422829	4.471155
H	-1.637935	-4.562905	-4.154622
H	5.090190	4.543191	0.154478
H	5.318793	0.317137	2.494246
C	4.095620	-0.611478	4.009660
H	2.643776	-1.566833	5.289237
H	4.020659	-2.786861	-4.202801
H	-4.023709	-2.753202	4.434658
H	4.861250	-0.561400	4.778027
H	-0.013951	4.617448	-0.864759
H	-1.960202	6.134531	-0.823436
H	-5.958283	0.708732	-0.000021
C	-4.032943	-1.162217	0.019405
H	-4.043015	-1.389039	1.093005
H	-4.988416	-1.494643	-0.396344
H	-3.227542	-1.742590	-0.430407

α,β-Me$_2$-TP

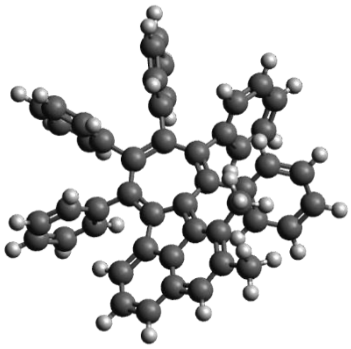

Table S123. Coordinates and energy for the optimized geometry of **α,β-Me$_2$-TP**.

G = –1886.809567			
		**Coordinates / Å**	
**Atom**	**x**	**y**	**z**
C	1.642260	-0.601913	-0.190355
C	1.455732	0.792941	-0.233721
C	0.690890	-1.589612	0.170788
C	-0.699296	-1.460129	0.117109
C	-1.464924	-0.280714	-0.268328
C	-1.065297	1.057041	-0.017317
C	0.269792	1.523930	0.048098
C	-2.827814	-0.725565	-0.581160
C	-3.924763	-0.167812	-1.252402
C	-5.148525	-0.916409	-1.274306
C	-5.224683	-2.194892	-0.737236
H	-6.169707	-2.729848	-0.774542
C	-1.663620	-2.560240	0.302244

C	-1.607061	-3.875340	0.762416
C	-2.911586	-2.069861	-0.142131
C	-2.790308	-4.648808	0.771844
C	-4.085778	-2.835652	-0.189020
C	-4.002870	-4.160766	0.306287
H	-4.887831	-4.790134	0.318770
C	-2.156449	2.027553	0.316339
C	-2.307245	3.261982	-0.332245
C	-3.036371	1.698752	1.357930
C	-3.309404	4.146238	0.054189
H	-1.652768	3.523492	-1.154254
C	-4.035999	2.587422	1.749606
H	-2.927527	0.749631	1.872775
C	-4.175517	3.814162	1.098974
H	-3.416794	5.093546	-0.464964
H	-4.701635	2.321209	2.564867
H	-4.954033	4.507351	1.402787
C	0.476389	2.950880	0.469136
C	0.301229	3.266353	1.821454
C	0.857022	3.955029	-0.427949
C	0.509448	4.569398	2.271745
H	0.000286	2.490340	2.518669
C	1.053921	5.258289	0.021692
H	0.998318	3.716392	-1.476160
C	0.884064	5.568940	1.372779
H	0.376403	4.801727	3.324039
H	1.343094	6.030883	-0.684266
H	1.043040	6.584558	1.722392
C	2.698768	1.603546	-0.473027
C	3.102773	1.870256	-1.784284
C	3.451777	2.096822	0.597370
C	4.245619	2.633870	-2.023390
H	2.520332	1.484262	-2.615498
C	4.593669	2.857396	0.356712
H	3.134112	1.896157	1.615443
C	4.992103	3.130140	-0.954029
H	4.550376	2.839669	-3.045113
H	5.171437	3.238592	1.193340
H	5.880914	3.725494	-1.140116
C	3.032730	-1.108135	-0.448643
C	3.955577	-1.259232	0.590686
C	3.398208	-1.454969	-1.753002
C	5.231670	-1.753089	0.326775
H	3.672034	-0.993266	1.603718
C	4.675543	-1.950179	-2.015419
H	2.682535	-1.337352	-2.561017
C	5.594524	-2.100897	-0.976175
H	5.942073	-1.867021	1.140068

H	4.950049	-2.217600	-3.031451
H	6.588741	-2.487113	-1.179747
C	1.243593	-2.923256	0.566478
C	1.486151	-3.178250	1.920055
C	1.503880	-3.912997	-0.386559
C	1.981111	-4.420393	2.318574
H	1.279002	-2.408982	2.657788
C	1.998668	-5.152653	0.014623
H	1.316466	-3.712249	-1.436122
C	2.235984	-5.409633	1.366997
H	2.165150	-4.613795	3.370957
H	2.197067	-5.918316	-0.729101
H	2.620283	-6.376521	1.677271
C	-3.857971	1.114352	-2.027988
H	-4.372099	1.930155	-1.507814
H	-2.830266	1.422365	-2.211924
H	-4.350836	0.988097	-2.997240
H	-0.694381	-4.324446	1.123764
H	-2.736338	-5.664864	1.149050
C	-6.375319	-0.313510	-1.914242
H	-6.584721	0.686620	-1.519036
H	-6.254796	-0.210278	-2.999681
H	-7.251739	-0.941068	-1.735470

α,β-Me$_2$-DT

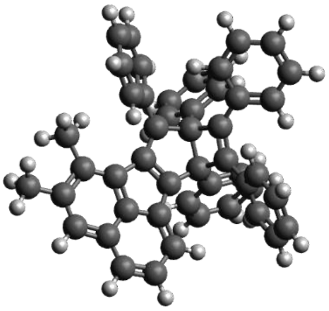

Table S124. Coordinates and energy for the optimized geometry of **α,β-Me$_2$-DT**.

G = –1886.792792			
		**Coordinates / Å**	
**Atom**	**x**	**y**	**z**
C	4.701686	-0.807081	-0.279639
C	5.006872	-2.180435	-0.433301
C	3.357323	-0.497380	-0.039254
H	6.031034	-2.481501	-0.634787
C	4.015000	-3.163415	-0.320719
C	2.336207	-1.492904	0.094808
C	2.677938	-2.844507	-0.045302
C	1.141344	-0.781295	0.370831
C	2.864898	0.818375	0.146793

C	1.409414	0.630879	0.345274
C	-0.295550	-1.077361	0.549573
C	3.770393	1.872224	0.195171
C	5.599692	0.295225	-0.325696
C	0.220925	1.337688	0.333536
C	-0.961491	0.393450	0.524167
C	-0.999223	-1.281074	-0.813726
C	-0.681412	-1.988314	1.688996
C	5.159567	1.585366	-0.085877
H	6.651947	0.118424	-0.528272
C	0.055675	2.741526	-0.022729
C	-1.614435	-0.075375	-0.814810
C	-1.855904	0.737381	1.690351
C	-0.828439	-2.399964	-1.734595
C	0.202877	-2.231992	2.745716
C	-1.970021	-2.535485	1.738961
C	0.721207	3.214152	-1.172033
C	-0.811541	3.605537	0.674634
C	-2.616249	0.550290	-1.674413
C	-1.263239	0.998549	2.935327
C	-3.249210	0.730896	1.588419
C	-0.559881	-3.693503	-1.252144
C	-0.856366	-2.189585	-3.126213
H	1.201086	-1.805835	2.727995
C	-0.191182	-3.014917	3.831465
C	-2.364342	-3.313668	2.824823
H	-2.663761	-2.348721	0.925872
H	1.372677	2.543760	-1.722444
C	0.506843	4.511100	-1.624183
C	-0.990694	4.913601	0.234962
H	-1.322104	3.256912	1.562697
C	-2.789008	1.944513	-1.728438
C	-3.475403	-0.262495	-2.442309
H	-0.180956	0.994753	3.026449
C	-2.050483	1.257014	4.054075
C	-4.038286	0.991242	2.710777
H	-3.723486	0.521813	0.637489
H	-0.542519	-3.874287	-0.183125
C	-0.342067	-4.748402	-2.136833
C	-0.636026	-3.245275	-4.005260
H	-1.037386	-1.191528	-3.510961
H	0.506632	-3.196062	4.643564
C	-1.475632	-3.557218	3.874766
H	-3.366302	-3.731478	2.849623
H	1.003643	4.855680	-2.525446
C	-0.344496	5.365664	-0.918841
H	-1.644432	5.579323	0.789255
H	-2.160716	2.590007	-1.131940

C	-3.764966	2.507853	-2.547555
C	-4.449000	0.303923	-3.257673
H	-3.381513	-1.341290	-2.384340
H	-1.577719	1.456065	5.011141
C	-3.443709	1.255715	3.944020
H	-5.120116	0.984259	2.616902
H	-0.143803	-5.742506	-1.747393
C	-0.379764	-4.529251	-3.514748
H	-0.655891	-3.065075	-5.075970
H	-1.783160	-4.165902	4.719741
H	-0.503693	6.381453	-1.266893
H	-3.876057	3.587653	-2.578689
C	-4.595330	1.693259	-3.318455
H	-5.101940	-0.339374	-3.839943
H	-4.059445	1.457266	4.815381
H	-0.207657	-5.351218	-4.203027
H	-5.356793	2.134440	-3.954504
C	3.377534	3.271393	0.575201
H	3.396910	3.943687	-0.291290
H	2.377597	3.307935	1.004595
H	4.081276	3.677493	1.308724
H	1.934913	-3.626971	0.053591
H	4.294394	-4.204589	-0.439677
C	6.153734	2.720049	-0.099864
H	5.841478	3.520927	-0.779550
H	6.262914	3.170245	0.894360
H	7.138264	2.368429	-0.417349

α,β-Me$_2$-DT′

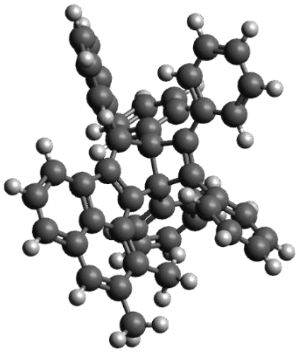

Table S125. Coordinates and energy for the optimized geometry of **α,β-Me$_2$-DT′**.

G = –1886.797954			
		Coordinates / Å	
**Atom**	**x**	**y**	**z**
C	-3.553309	3.089513	-0.541501
C	-4.780151	2.405375	-0.341915
C	-2.411241	2.283612	-0.569098
C	-4.853136	1.019447	-0.184917

C	-2.449463	0.852555	-0.443554
C	-3.682934	0.203519	-0.238786
C	-1.093189	0.430974	-0.550356
C	-1.095769	2.781256	-0.717377
C	-0.242117	1.582795	-0.667569
C	-0.305487	-0.820861	-0.466409
C	-0.926623	4.145534	-0.885540
C	-3.354751	4.487768	-0.705342
C	1.092154	1.232990	-0.592771
C	1.218727	-0.279488	-0.454334
C	-0.045797	-1.182356	1.022381
C	-0.641708	-1.929493	-1.429470
C	-2.072598	4.983739	-0.880181
C	2.206393	2.162649	-0.467124
C	1.222111	-0.727940	1.041487
C	2.146029	-0.969116	-1.422844
C	-1.050951	-1.636654	1.994811
C	-1.371645	-1.673203	-2.596399
C	-0.148187	-3.220793	-1.207877
C	2.051726	3.295731	0.358988
C	3.451457	1.939920	-1.091030
C	2.240856	-0.609737	2.077208
C	2.025457	-0.685168	-2.791528
C	3.052108	-1.948601	-1.009357
C	-1.455482	-2.977208	2.078306
C	-1.696394	-0.675586	2.791635
H	-1.752000	-0.674811	-2.789514
C	-1.617185	-2.692286	-3.516187
C	-0.389162	-4.238484	-2.128353
H	0.444646	-3.422240	-0.322390
H	1.114347	3.450425	0.880656
C	3.111436	4.172316	0.561969
C	4.497888	2.836419	-0.903773
H	3.585793	1.083979	-1.738795
C	3.500112	-0.048825	1.802844
C	1.970795	-1.056500	3.386627
H	1.312550	0.063610	-3.124466
C	2.806835	-1.359501	-3.725453
C	3.836957	-2.624340	-1.946079
H	3.146960	-2.189274	0.043159
H	-0.969130	-3.725707	1.463001
C	-2.487707	-3.346977	2.939663
C	-2.730283	-1.048459	3.648685
H	-1.386359	0.362919	2.725871
H	-2.188174	-2.478180	-4.414720
C	-1.127642	-3.978356	-3.284577
H	0.002033	-5.234165	-1.942254
H	2.984981	5.025410	1.220829

C	4.335431	3.949000	-0.073713
H	5.445327	2.664291	-1.404423
H	3.733515	0.288132	0.801504
C	4.455459	0.077441	2.808545
C	2.928174	-0.929118	4.387068
H	1.007771	-1.499855	3.613951
H	2.702340	-1.129414	-4.781480
C	3.718012	-2.332159	-3.304092
H	4.539037	-3.381339	-1.609637
H	-2.792506	-4.387586	2.996767
C	-3.130996	-2.384812	3.721248
H	-3.223854	-0.296186	4.256706
H	-1.317943	-4.772113	-4.000653
H	5.160679	4.637396	0.079638
H	5.420705	0.517740	2.577194
C	4.173603	-0.359290	4.103769
H	2.703983	-1.275919	5.391435
H	4.328023	-2.858931	-4.031671
H	-3.938408	-2.675358	4.386666
H	4.918436	-0.260239	4.887685
H	-1.930687	6.051433	-1.011642
H	-4.206330	5.161260	-0.695157
H	-5.701985	2.979633	-0.300713
H	0.055083	4.585634	-1.020110
C	-3.800793	-1.276661	-0.052161
H	-4.128794	-1.502866	0.970315
H	-4.552546	-1.695145	-0.730431
H	-2.859590	-1.792404	-0.222487
C	-6.196353	0.375052	0.049545
H	-6.445975	-0.333178	-0.749228
H	-6.208241	-0.187421	0.990279
H	-6.987673	1.126685	0.093451

α,α′-Me$_2$-TP

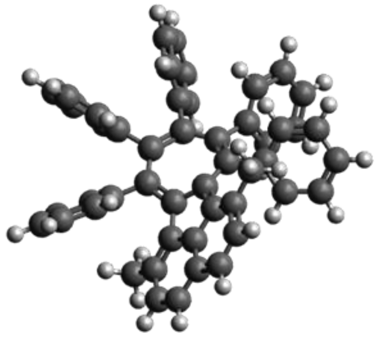

Table S126. Coordinates and energy for the optimized geometry of **α,α′-Me$_2$-TP**.

G = –1886.804624
**Coordinates / Å**

Atom	x	y	z
C	1.015242	1.250886	0.130657
C	1.615645	-0.023786	0.001013
C	-0.329920	1.589999	-0.180691
C	-1.405469	0.687293	-0.295741
C	-1.424890	-0.646611	0.296038
C	-0.376080	-1.580345	0.181264
C	0.978577	-1.280477	-0.129338
C	-2.819297	-0.927757	0.667447
C	-3.467583	-1.826526	1.528176
C	-4.888439	-1.781845	1.557057
C	-5.644924	-0.899557	0.806718
H	-5.396829	-2.478809	2.216617
H	-6.729154	-0.927284	0.862495
C	-2.791189	1.009093	-0.666766
C	-3.413315	1.926458	-1.527176
C	-3.598752	0.052235	0.000432
C	-4.834889	1.923339	-1.555441
C	-5.002342	0.072740	0.000728
C	-5.616564	1.063450	-0.804950
H	-5.322806	2.634853	-2.214919
H	-6.699542	1.122749	-0.860404
C	-0.739631	-3.029780	0.245204
C	-0.098215	-3.905662	1.132002
C	-1.708067	-3.534950	-0.632123
C	-0.422673	-5.258600	1.144860
H	0.641634	-3.521635	1.824969
C	-2.027193	-4.891935	-0.624785
H	-2.201896	-2.866726	-1.329835
C	-1.386354	-5.756913	0.263997
H	0.074347	-5.923699	1.844200
H	-2.773532	-5.272068	-1.315525
H	-1.635677	-6.813628	0.270812
C	1.828969	-2.433351	-0.579967
C	1.595905	-2.946456	-1.861595
C	2.833846	-3.000660	0.212195
C	2.364204	-4.003601	-2.348612
H	0.812184	-2.514666	-2.476914
C	3.592623	-4.063430	-0.270670
H	3.022697	-2.609874	1.205544
C	3.363433	-4.566057	-1.553703
H	2.178196	-4.387543	-3.347168
H	4.365387	-4.498317	0.356026
H	3.959714	-5.391738	-1.930210
C	3.119331	-0.045430	0.001173
C	3.823522	0.057159	1.204939
C	3.820383	-0.167945	-1.202579
C	5.217157	0.035185	1.204230

H	3.279825	0.161555	2.138542
C	5.214089	-0.185040	-1.201819
H	3.273941	-0.257639	-2.136090
C	5.915641	-0.084596	0.001199
H	5.756374	0.113281	2.143482
H	5.750941	-0.278422	-2.141034
H	7.001376	-0.099669	0.001201
C	1.899050	2.378984	0.580005
C	2.918459	2.917228	-0.213913
C	1.682912	2.898817	1.861845
C	3.708341	3.957688	0.267599
H	3.094333	2.521464	-1.207648
C	2.482069	3.933518	2.347486
H	0.888199	2.489773	2.478621
C	3.495839	4.466834	1.550940
H	4.492257	4.370089	-0.360449
H	2.308725	4.322753	3.346270
H	4.116295	5.275008	1.926358
C	-0.650824	3.049456	-0.245465
C	0.015460	3.905236	-1.133512
C	-1.603399	3.583594	0.632068
C	-0.268965	5.267155	-1.147294
H	0.743137	3.498728	-1.826586
C	-1.882393	4.949392	0.623782
H	-2.116111	2.930716	1.330719
C	-1.216930	5.794334	-0.266172
H	0.246893	5.916792	-1.847524
H	-2.616754	5.351984	1.314634
H	-1.435007	6.857930	-0.273713
C	-2.784212	-2.768368	2.478003
H	-2.697880	-3.771869	2.048765
H	-1.782530	-2.429053	2.741863
H	-3.374348	-2.852735	3.395926
C	-2.703566	2.847951	-2.477602
H	-2.588067	3.848613	-2.048607
H	-1.712281	2.479941	-2.742235
H	-3.291767	2.949080	-3.395061

α,α′-Me$_2$-DT

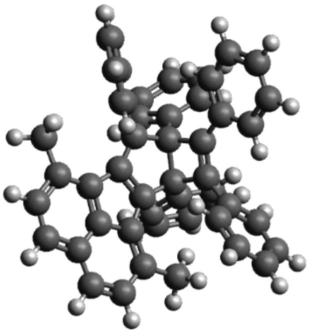

Table S127. Coordinates and energy for the optimized geometry of **α,α′-Me$_2$-DT**.

	G = –1886.804624		
		**Coordinates / Å**	
**Atom**	**x**	**y**	**z**
C	-3.636146	3.153159	-0.164658
C	-4.875912	2.505273	0.061963
C	-2.522836	2.314172	-0.297112
H	-5.777339	3.099743	0.181529
C	-4.964483	1.116970	0.129762
C	-2.604107	0.876352	-0.254344
H	-5.936559	0.666635	0.304524
C	-3.854438	0.257785	-0.025508
C	-1.281597	0.415140	-0.478665
C	-1.202936	2.777247	-0.523616
C	-0.393141	1.547226	-0.580476
C	-0.539544	-0.868552	-0.506087
C	-0.991373	4.141389	-0.712099
C	-3.397510	4.549018	-0.291215
C	0.921551	1.135863	-0.518661
C	1.000867	-0.383318	-0.515358
C	-0.232718	-1.354545	0.937689
C	-0.949323	-1.888046	-1.537267
C	-2.122634	5.001804	-0.567300
H	-4.219497	5.251970	-0.195201
C	2.083153	1.974512	-0.236089
C	1.054184	-0.954980	0.934526
C	1.873453	-0.992260	-1.582782
C	-1.203999	-1.852769	1.922303
C	-1.706935	-1.516514	-2.654685
C	-0.492589	-3.208002	-1.436639
H	-1.960665	6.067628	-0.697802
C	2.018635	2.836010	0.875552
C	3.283455	1.876062	-0.964102
C	2.123064	-0.961003	1.927323
C	1.731583	-0.547934	-2.905913
C	2.749101	-2.046071	-1.309584
C	-1.683044	-3.170843	1.897007
C	-1.731692	-0.950102	2.861343
H	-2.057641	-0.494074	-2.755841
C	-2.016960	-2.450441	-3.643207
C	-0.797705	-4.140247	-2.425962
H	0.121188	-3.499329	-0.591373
H	1.100591	2.894294	1.450968
C	3.138568	3.563930	1.264564
C	4.387142	2.636652	-0.589711
H	3.340202	1.223289	-1.826101
C	3.401969	-0.460375	1.627588

C	1.883496	-1.467753	3.220680
H	1.038179	0.257337	-3.131244
C	2.464490	-1.137609	-3.932525
C	3.483909	-2.637448	-2.338926
H	2.857750	-2.410229	-0.294484
H	-1.288750	-3.873449	1.171806
C	-2.671381	-3.576244	2.793010
C	-2.722492	-1.357717	3.752901
H	-1.364828	0.071697	2.878672
H	-2.608571	-2.146588	-4.501607
C	-1.564189	-3.765464	-3.531473
H	-0.434406	-5.159289	-2.333221
H	3.086860	4.203873	2.139671
C	4.322897	3.470333	0.530146
H	5.303205	2.571432	-1.168162
H	3.613724	-0.080024	0.637325
C	4.404621	-0.447195	2.594075
C	2.888649	-1.454860	4.181770
H	0.906191	-1.866157	3.468425
H	2.344882	-0.783267	-4.951927
C	3.345984	-2.184852	-3.650687
H	4.162234	-3.454340	-2.111616
H	-3.034602	-4.599123	2.765193
C	-3.197082	-2.671338	3.718028
H	-3.125562	-0.649997	4.471080
H	-1.804061	-4.492798	-4.301150
H	5.193515	4.045928	0.828682
H	5.383166	-0.048994	2.343197
C	4.152525	-0.941107	3.874771
H	2.686435	-1.846255	5.174359
H	3.917970	-2.645612	-4.450365
H	-3.971092	-2.988647	4.410309
H	4.934088	-0.929691	4.628551
C	0.332430	4.745327	-1.089274
H	0.890243	5.060297	-0.199751
H	0.962185	4.038298	-1.632276
H	0.180257	5.631396	-1.712587
C	-4.044518	-1.223058	0.090236
H	-4.034648	-1.513568	1.148008
H	-5.013372	-1.519244	-0.322441
H	-3.257930	-1.784614	-0.413026

α-Et-TP

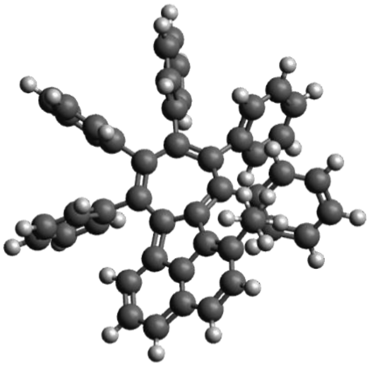

Table S128. Coordinates and energy for the optimized geometry of **α-Et-TP**.

G = –1886.804036			
	Coordinates / Å		
Atom	x	y	z
C	1.678138	0.415679	0.150597
C	1.321135	-0.949743	0.106771
C	0.862872	1.526308	-0.178000
C	-0.535229	1.556897	-0.223935
C	-1.454750	0.501262	0.182629
C	-1.224698	-0.882529	-0.000373
C	0.040925	-1.515702	-0.124703
C	-2.760241	1.135164	0.426563
C	-3.939926	0.778924	1.096772
C	-5.034879	1.682931	0.993760
C	-4.965342	2.907655	0.355259
H	-5.971123	1.398837	1.461698
H	-5.841461	3.547749	0.310375
C	-1.348783	2.743182	-0.531314
C	-1.111680	3.988003	-1.113307
C	-2.665204	2.442754	-0.114921
C	-2.187125	4.892688	-1.255775
H	-0.135275	4.280093	-1.471788
C	-3.734486	3.350683	-0.194234
C	-3.466402	4.602306	-0.801456
H	-1.994668	5.851928	-1.724974
H	-4.264426	5.330694	-0.911236
C	-2.431816	-1.745249	-0.211687
C	-2.680576	-2.889830	0.559193
C	-3.314644	-1.421485	-1.250756
C	-3.785502	-3.692607	0.295678
H	-2.017452	-3.142018	1.377838
C	-4.417318	-2.230881	-1.520575
H	-3.128747	-0.542599	-1.859556
C	-4.655860	-3.368467	-0.748436
H	-3.968710	-4.570290	0.907683
H	-5.085949	-1.972721	-2.335975

H	-5.514619	-3.999109	-0.957445
C	0.028669	-2.964149	-0.523381
C	-0.285465	-3.280502	-1.850026
C	0.334082	-3.992814	0.374845
C	-0.288014	-4.608583	-2.274383
H	-0.528963	-2.485193	-2.547867
C	0.320158	-5.319548	-0.047715
H	0.581314	-3.754502	1.403524
C	0.012502	-5.631571	-1.373955
H	-0.527328	-4.841816	-3.307521
H	0.552414	-6.109970	0.659527
H	0.006883	-6.666308	-1.702993
C	2.466352	-1.914223	0.246513
C	2.988287	-2.191544	1.513634
C	3.025031	-2.526291	-0.880463
C	4.053856	-3.080248	1.652810
H	2.561790	-1.710532	2.388233
C	4.093554	-3.408850	-0.740096
H	2.616718	-2.317159	-1.863717
C	4.609300	-3.690111	0.526944
H	4.450016	-3.293052	2.641164
H	4.521908	-3.877982	-1.620716
H	5.440492	-4.380283	0.635715
C	3.106664	0.738355	0.482578
C	4.136114	0.589201	-0.452230
C	3.404396	1.204792	1.767345
C	5.449198	0.895664	-0.102208
H	3.908856	0.229526	-1.449933
C	4.720582	1.504590	2.118527
H	2.605640	1.325735	2.493144
C	5.745799	1.350572	1.184680
H	6.241562	0.777348	-0.835153
H	4.942030	1.859530	3.120596
H	6.770620	1.584846	1.456566
C	1.575450	2.819154	-0.429102
C	2.186853	3.046195	-1.666175
C	1.609468	3.812186	0.555820
C	2.813864	4.266010	-1.921596
H	2.158893	2.275024	-2.429614
C	2.247348	5.025016	0.302741
H	1.132635	3.634217	1.514546
C	2.844994	5.256797	-0.938224
H	3.278045	4.440134	-2.887499
H	2.272564	5.790505	1.072214
H	3.335286	6.204873	-1.137157
C	-4.078532	-0.425322	1.994497
H	-4.701920	-1.180148	1.499563
H	-3.099160	-0.879257	2.140234

C	-4.668616	-0.094240	3.375347
H	-5.700456	0.263068	3.314366
H	-4.666908	-0.994867	3.997395
H	-4.073490	0.672825	3.881914

α-Et-DT

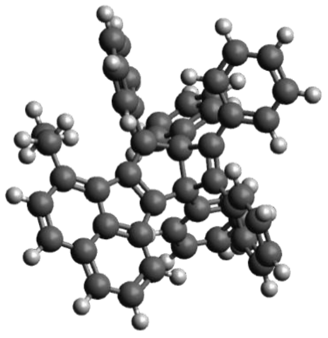

Table S129. Coordinates and energy for the optimized geometry of **α-Et-DT**.

G = –1886.790248			
	**Coordinates / Å**		
**Atom**	**x**	**y**	**z**
C	4.667632	1.293623	-0.539467
C	5.538758	0.178284	-0.576660
C	3.317771	1.020852	-0.270243
H	6.592213	0.332106	-0.792528
C	5.073454	-1.119129	-0.331109
C	2.836098	-0.301428	0.005175
H	5.776300	-1.944276	-0.365952
C	3.730268	-1.379364	-0.025713
C	1.455182	-0.157662	0.283110
C	2.294293	2.000519	-0.189194
H	3.398470	-2.390387	0.179442
C	1.073741	1.220056	0.113965
C	0.300834	-1.028511	0.582765
C	2.654063	3.343648	-0.300070
C	5.005299	2.664028	-0.720855
C	-0.302055	1.335099	0.151439
C	-0.941824	-0.004251	0.503965
C	-0.283946	-1.637426	-0.716842
C	0.388734	-1.915929	1.799977
C	4.027148	3.627972	-0.587758
H	6.029957	2.948803	-0.938478
C	-1.093394	2.478745	-0.291283
C	-1.370465	-0.829522	-0.750517
C	-1.851141	0.033706	1.708184
C	0.349325	-2.635439	-1.572893
C	1.325042	-1.664539	2.808859
C	-0.530652	-2.959363	1.968628

H	4.305201	4.672035	-0.694287
C	-0.836767	2.994023	-1.576769
C	-2.162035	3.001325	0.461144
C	-2.571355	-0.763810	-1.577524
C	-1.386715	0.649095	2.880853
C	-3.102389	-0.587406	1.716889
C	1.155699	-3.647502	-1.021223
C	0.231585	-2.552959	-2.973177
H	2.037806	-0.853410	2.699516
C	1.347363	-2.446992	3.964084
C	-0.510552	-3.737739	3.123536
H	-1.262362	-3.159485	1.192891
H	-0.028895	2.573607	-2.166897
C	-1.649657	3.991184	-2.107891
C	-2.943881	4.026464	-0.063029
H	-2.362108	2.618013	1.453360
C	-3.390526	0.379260	-1.602936
C	-2.961642	-1.884666	-2.339170
H	-0.408057	1.120071	2.891030
C	-2.166330	0.655263	4.034366
C	-3.883884	-0.581229	2.874186
H	-3.470827	-1.079743	0.825414
H	1.253054	-3.728687	0.055732
C	1.808958	-4.561068	-1.846670
C	0.887852	-3.465881	-3.793051
H	-0.363778	-1.759446	-3.412382
H	2.080745	-2.240052	4.737736
C	0.429667	-3.485002	4.125317
H	-1.229005	-4.543600	3.239768
H	-1.461410	4.360782	-3.110718
C	-2.700178	4.513548	-1.350192
H	-3.752684	4.439328	0.531276
H	-3.135356	1.239893	-1.001306
C	-4.536471	0.415343	-2.393746
C	-4.107677	-1.845150	-3.125483
H	-2.367293	-2.790502	-2.300908
H	-1.793479	1.136206	4.933655
C	-3.421422	0.040643	4.033284
H	-4.855097	-1.066855	2.865694
H	2.420608	-5.341489	-1.403970
C	1.677185	-4.474964	-3.233596
H	0.790459	-3.385137	-4.871571
H	0.446016	-4.093729	5.024308
H	-3.327562	5.298323	-1.760950
H	-5.148911	1.311991	-2.403442
C	-4.897527	-0.691809	-3.162784
H	-4.391127	-2.719131	-3.704260
H	-4.031136	0.044777	4.931733

H	2.188209	-5.186037	-3.875587
H	-5.791864	-0.662595	-3.777858
C	1.714982	4.495840	-0.052494
H	2.229202	5.430534	-0.296679
H	0.850914	4.428501	-0.718097
C	1.225256	4.548918	1.405458
H	2.067686	4.656961	2.096411
H	0.551892	5.400182	1.544820
H	0.679995	3.640241	1.676049

α-Et-DT′

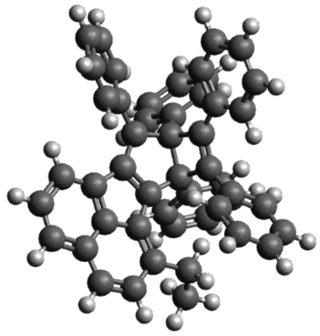

Table S130. Coordinates and energy for the optimized geometry of **α-Et-DT′**.

G = –1886.793151			
		**Coordinates / Å**	
**Atom**	**x**	**y**	**z**
C	-3.174792	3.583041	-0.426389
C	-4.472619	3.072930	-0.152655
C	-2.151375	2.629207	-0.480731
H	-5.313032	3.757649	-0.081773
C	-4.686775	1.712303	0.038755
C	-2.364226	1.216148	-0.313289
C	-3.660526	0.738862	-0.036951
C	-1.080533	0.620995	-0.466189
C	-0.789746	2.946302	-0.701178
C	-0.095827	1.648161	-0.655380
C	-0.462544	-0.721674	-0.385975
C	-0.451867	4.271807	-0.917820
C	-2.801466	4.937910	-0.639016
C	1.183891	1.125930	-0.635362
C	1.117734	-0.387943	-0.467704
C	-0.169647	-1.087627	1.096377
C	-0.998786	-1.788490	-1.304106
C	-1.475315	5.255206	-0.885622
H	-3.555815	5.718122	-0.610709
C	2.414323	1.902212	-0.585493
C	1.145826	-0.805151	1.036430

C	1.888083	-1.213890	-1.466375
C	-1.169685	-1.387660	2.132415
C	-1.713805	-1.446605	-2.458307
C	-0.704069	-3.135510	-1.063826
C	2.449389	3.064800	0.213350
C	3.587311	1.503394	-1.259569
C	2.227356	-0.796618	2.013590
C	1.742011	-0.933149	-2.833180
C	2.661559	-2.309984	-1.076614
C	-1.700947	-2.673836	2.308813
C	-1.677679	-0.325687	2.900268
H	-1.934335	-0.405181	-2.671138
C	-2.143771	-2.434123	-3.344456
C	-1.131270	-4.122802	-1.949427
H	-0.119276	-3.408429	-0.192463
H	1.567587	3.353311	0.773917
C	3.622632	3.799374	0.340515
C	4.748784	2.259751	-1.148893
H	3.576636	0.622194	-1.887131
C	3.536659	-0.437746	1.649831
C	1.969653	-1.144340	3.355062
H	1.130139	-0.092629	-3.147669
C	2.370722	-1.725891	-3.789227
C	3.293044	-3.104701	-2.035544
H	2.771957	-2.548573	-0.025085
H	-1.319294	-3.499865	1.719557
C	-2.723934	-2.890840	3.231215
C	-2.701563	-0.546359	3.819701
H	-1.268106	0.670681	2.764233
H	-2.699298	-2.151787	-4.233781
C	-1.856750	-3.775905	-3.090922
H	-0.894346	-5.163167	-1.748031
H	3.641290	4.676523	0.979315
C	4.773537	3.403367	-0.345768
H	5.639530	1.953718	-1.688112
H	3.759318	-0.180718	0.622671
C	4.555292	-0.410839	2.599344
C	2.990173	-1.116936	4.299267
H	0.966817	-1.430926	3.651597
H	2.248701	-1.496488	-4.843510
C	3.151037	-2.815405	-3.392014
H	3.893604	-3.951638	-1.717527
H	-3.127655	-3.890605	3.359671
C	-3.230129	-1.828924	3.983480
H	-3.087597	0.282644	4.405264
H	-2.191352	-4.545430	-3.779973
H	5.687455	3.981655	-0.252429
H	5.559258	-0.126278	2.299092

C	4.286683	-0.747519	3.926863
H	2.775322	-1.384554	5.329543
H	3.641745	-3.434481	-4.136940
H	-4.030308	-2.000579	4.697163
H	5.080797	-0.725770	4.667152
H	0.571542	4.574306	-1.109696
H	-1.200455	6.291122	-1.054585
H	-5.693870	1.374786	0.261692
C	-3.990502	-0.711323	0.182144
H	-4.434086	-0.812826	1.180982
H	-3.080145	-1.308856	0.188283
C	-4.961488	-1.270538	-0.871872
H	-5.173898	-2.321610	-0.654047
H	-5.910645	-0.725641	-0.879077
H	-4.522566	-1.212778	-1.871857

α-^{i}Pr-TP

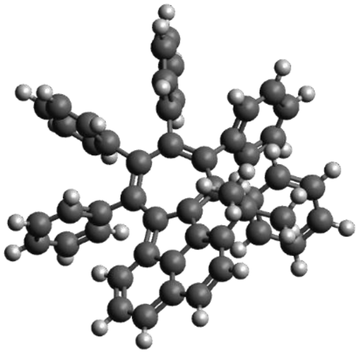

Table S131. Coordinates and energy for the optimized geometry of **α-^{i}Pr-TP**.

	G = –1927.045377		
		Coordinates / Å	
**Atom**	**x**	**y**	**z**
C	1.785990	0.285835	0.161326
C	1.294197	-1.031115	0.214045
C	1.081733	1.454607	-0.226822
C	-0.303509	1.634218	-0.180394
C	-1.314130	0.643894	0.162909
C	-1.206771	-0.739684	-0.139511
C	-0.006128	-1.486102	-0.141432
C	-2.547302	1.367108	0.490978
C	-3.739022	1.052166	1.161227
C	-4.721592	2.078834	1.243702
C	-4.534668	3.363092	0.767377
H	-5.660264	1.844813	1.733796
H	-5.328370	4.098324	0.862354
C	-0.998203	2.927765	-0.328840
C	-0.640576	4.211996	-0.736332

C	-2.325460	2.716780	0.108287
C	-1.610950	5.239150	-0.700789
H	0.350891	4.452631	-1.088502
C	-3.285879	3.737428	0.206964
C	-2.900277	5.026573	-0.236142
H	-1.323273	6.229653	-1.037971
H	-3.614352	5.844234	-0.206744
C	-2.455825	-1.420293	-0.610606
C	-2.921607	-2.636665	-0.088375
C	-3.137397	-0.845564	-1.694092
C	-4.020951	-3.274165	-0.653185
H	-2.426703	-3.084017	0.764042
C	-4.234834	-1.489374	-2.264858
H	-2.787846	0.093592	-2.110753
C	-4.675314	-2.709278	-1.750845
H	-4.368372	-4.212771	-0.233038
H	-4.738198	-1.039039	-3.114790
H	-5.526879	-3.213927	-2.196838
C	-0.083506	-2.920772	-0.580982
C	-0.224123	-3.179913	-1.949155
C	0.001342	-3.990165	0.317345
C	-0.275966	-4.493458	-2.413617
H	-0.299725	-2.351549	-2.646787
C	-0.059899	-5.301627	-0.147728
H	0.111618	-3.796122	1.378409
C	-0.195942	-5.557393	-1.514005
H	-0.381151	-4.683201	-3.477537
H	0.000476	-6.124115	0.558526
H	-0.239347	-6.580545	-1.874925
C	2.308922	-2.093193	0.531001
C	2.523444	-2.457022	1.863514
C	3.033268	-2.725704	-0.484503
C	3.448713	-3.453017	2.177633
H	1.963021	-1.964648	2.652622
C	3.956318	-3.720049	-0.168970
H	2.862998	-2.447908	-1.519580
C	4.164946	-4.087873	1.162384
H	3.606939	-3.731551	3.215201
H	4.512142	-4.208611	-0.963607
H	4.883429	-4.864576	1.406380
C	3.248895	0.475360	0.445296
C	4.192592	0.470343	-0.586658
C	3.666527	0.687747	1.762802
C	5.541152	0.676015	-0.302336
H	3.868883	0.310894	-1.610025
C	5.016480	0.896327	2.045271
H	2.934015	0.694880	2.564374
C	5.956209	0.891328	1.013621

H	6.267251	0.669854	-1.109694
H	5.331509	1.063036	3.070989
H	7.007073	1.054235	1.233160
C	1.916775	2.630460	-0.624446
C	2.167587	2.847365	-1.983241
C	2.433039	3.513251	0.329632
C	2.929699	3.944702	-2.385636
H	1.761420	2.162108	-2.721211
C	3.192214	4.609293	-0.075769
H	2.237915	3.341503	1.382767
C	3.440978	4.827911	-1.433005
H	3.121023	4.108898	-3.441659
H	3.588342	5.293371	0.668422
H	4.032658	5.682636	-1.746299
C	-3.979247	-0.229023	1.927896
H	-3.165218	-0.918122	1.708813
C	-3.921482	0.062443	3.442277
H	-4.720076	0.746705	3.748093
H	-4.041792	-0.871661	4.001613
H	-2.962700	0.511330	3.721853
C	-5.304151	-0.906650	1.544136
H	-5.378734	-1.052401	0.464373
H	-5.371960	-1.886887	2.026463
H	-6.164516	-0.314397	1.873616

α-^{i}Pr-DT

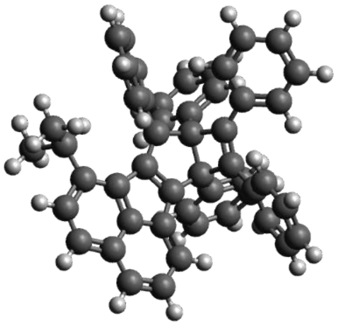

Table S132. Coordinates and energy for the optimized geometry of **α-^{i}Pr-DT**.

G = –1927.024917			
	Coordinates / Å		
**Atom**	**x**	**y**	**z**
C	-3.953621	2.623095	-0.410415
C	-3.715073	4.014290	-0.506579
C	-2.835691	1.816047	-0.152383
H	-4.544518	4.684041	-0.715627
C	-2.432750	4.548354	-0.326446
C	-1.523595	2.354213	0.052031
H	-2.295952	5.621414	-0.403250
C	-1.326844	3.738845	-0.034188

C	-0.690973	1.243006	0.330459
C	-2.872239	0.402918	-0.014952
H	-0.348328	4.178119	0.120398
C	-1.459844	0.030272	0.239734
C	0.744560	0.985641	0.560319
C	-4.110010	-0.232723	-0.047829
C	-5.201671	1.945760	-0.522436
C	-0.614709	-1.063146	0.262311
C	0.822555	-0.624761	0.527245
C	1.516039	0.922702	-0.782256
C	1.402074	1.685684	1.723788
C	-5.254752	0.581996	-0.332198
H	-6.107442	2.505989	-0.732379
C	-0.948542	-2.429727	-0.126434
C	1.652348	-0.424648	-0.780186
C	1.474291	-1.282237	1.718999
C	1.771199	2.031766	-1.696237
C	0.636630	2.215113	2.768891
C	2.799272	1.736915	1.809232
H	-6.221081	0.091999	-0.384977
C	-1.598085	-2.621343	-1.361604
C	-0.562966	-3.551457	0.631752
C	2.370724	-1.380498	-1.617296
C	0.764008	-1.340056	2.927920
C	2.787515	-1.757339	1.679301
C	2.009377	3.328566	-1.206554
C	1.698327	1.837715	-3.088583
H	-0.447161	2.171957	2.724883
C	1.256674	2.793963	3.876759
C	3.417991	2.311752	2.917039
H	3.400839	1.325054	1.005697
H	-1.878501	-1.757111	-1.954289
C	-1.835861	-3.906219	-1.837186
C	-0.839590	-4.833059	0.164218
H	-0.071386	-3.415782	1.586382
C	2.085447	-2.757159	-1.581040
C	3.415313	-0.934784	-2.453358
H	-0.252995	-0.961149	2.973819
C	1.352849	-1.875108	4.070567
C	3.377433	-2.294160	2.825443
H	3.355312	-1.708367	0.758277
H	2.073850	3.494562	-0.136967
C	2.187819	4.395574	-2.085444
C	1.874901	2.906538	-3.961886
H	1.486315	0.847584	-3.478258
H	0.649242	3.201762	4.679215
C	2.648611	2.843700	3.954452
H	4.502098	2.345353	2.968563

H	-2.315170	-4.042376	-2.801175
C	-1.462976	-5.014648	-1.073243
H	-0.559321	-5.692946	0.764149
H	1.313334	-3.131382	-0.924038
C	2.791559	-3.651092	-2.382463
C	4.119001	-1.830801	-3.250364
H	3.679193	0.116636	-2.465026
H	0.790433	-1.913184	4.998641
C	2.663728	-2.356792	4.021676
H	4.398433	-2.661057	2.779253
H	2.379054	5.388936	-1.690637
C	2.122496	4.189322	-3.464382
H	1.810681	2.740519	-5.033139
H	3.131678	3.293644	4.816546
H	-1.658731	-6.017271	-1.440237
H	2.547997	-4.708623	-2.343483
C	3.805829	-3.193415	-3.224627
H	4.919998	-1.467300	-3.887105
H	3.124495	-2.774580	4.911706
H	2.259446	5.021879	-4.147796
H	4.355971	-3.891688	-3.848139
C	-4.319758	-1.695658	0.278670
H	-3.341056	-2.149738	0.435086
C	-5.016010	-2.452409	-0.864955
H	-4.478499	-2.331944	-1.809992
H	-5.055556	-3.521049	-0.630513
H	-6.043516	-2.102437	-1.011159
C	-5.103094	-1.837339	1.598537
H	-5.198701	-2.896792	1.859270
H	-4.588508	-1.326004	2.418662
H	-6.111433	-1.417675	1.514738

α-^{i}Pr-DT′

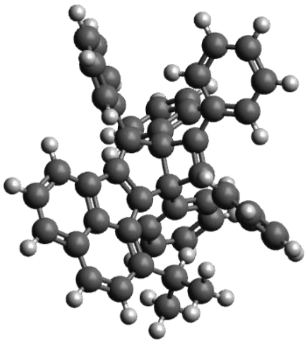

Table S133. Coordinates and energy for the optimized geometry of **α-^{i}Pr-DT′**.

G = –1927.029469			
		**Coordinates / Å**	
**Atom**	**x**	**y**	**z**

C	3.126735	3.687194	-0.305256
C	4.442038	3.153516	-0.346408
C	2.101207	2.751248	-0.123345
H	5.287851	3.820795	-0.486271
C	4.668545	1.788500	-0.213180
C	2.321569	1.337019	0.037920
C	3.637702	0.835720	-0.022915
C	1.029818	0.775510	0.258024
C	0.731289	3.094306	-0.051835
C	0.042367	1.814287	0.172549
C	0.401946	-0.547384	0.514847
C	0.381100	4.431096	-0.145631
C	2.742540	5.051255	-0.413997
C	-1.238924	1.312304	0.291998
C	-1.181125	-0.190895	0.525451
C	0.081469	-1.297466	-0.806311
C	0.915240	-1.338057	1.692444
C	1.403916	5.397765	-0.328776
H	3.499364	5.816438	-0.556376
C	-2.461171	2.072681	0.074053
C	-1.237983	-1.013644	-0.796519
C	-1.939647	-0.699511	1.726778
C	0.994983	-1.926748	-1.770770
C	1.613973	-0.708832	2.729239
C	0.592412	-2.694537	1.820944
C	-2.497013	2.998132	-0.990703
C	-3.624662	1.880047	0.848362
C	-2.358371	-1.341591	-1.671944
C	-1.757445	-0.057449	2.961019
C	-2.742852	-1.840763	1.666790
C	1.749948	-3.067545	-1.455526
C	1.109380	-1.369131	-3.056303
H	1.852732	0.347502	2.659788
C	2.004243	-1.426170	3.860165
C	0.980733	-3.412024	2.950477
H	0.016648	-3.184187	1.042983
H	-1.622703	3.119999	-1.619809
C	-3.661841	3.700192	-1.278073
C	-4.777151	2.606921	0.570657
H	-3.613480	1.184847	1.676956
C	-3.624052	-0.760068	-1.481044
C	-2.197507	-2.271219	-2.719948
H	-1.124289	0.823032	3.020650
C	-2.376605	-0.542237	4.109446
C	-3.365327	-2.326271	2.818750
H	-2.883438	-2.356315	0.724301
H	1.681616	-3.503821	-0.465754
C	2.576868	-3.651400	-2.414545

C	1.943878	-1.950665	-4.008831
H	0.532052	-0.482580	-3.300828
H	2.548565	-0.922069	4.653147
C	1.692510	-2.781216	3.972865
H	0.722699	-4.463517	3.032615
H	-3.681521	4.389179	-2.116330
C	-4.803115	3.511761	-0.494281
H	-5.660064	2.464021	1.185353
H	-3.779709	-0.062489	-0.669569
C	-4.687264	-1.074572	-2.324016
C	-3.262237	-2.583292	-3.558219
H	-1.237433	-2.750063	-2.870543
H	-2.225420	-0.035142	5.057627
C	-3.185503	-1.680087	4.040968
H	-3.988752	-3.213196	2.756455
H	3.146722	-4.540122	-2.160424
C	2.674825	-3.098162	-3.692098
H	2.020716	-1.509557	-4.998168
H	1.996522	-3.340534	4.852416
H	-5.710358	4.065808	-0.714087
H	-5.654596	-0.609031	-2.160494
C	-4.510942	-1.983075	-3.368370
H	-3.119456	-3.301042	-4.360573
H	-3.669242	-2.059368	4.936052
H	3.321071	-3.554779	-4.435626
H	-5.339825	-2.228347	-4.025571
H	-0.651421	4.754868	-0.079211
H	1.119723	6.442207	-0.403427
H	5.692502	1.434400	-0.260441
C	3.978180	-0.631144	0.076968
H	3.048478	-1.189165	0.203698
C	4.871011	-0.919514	1.297476
H	5.050112	-1.997242	1.369559
H	5.841955	-0.420577	1.210228
H	4.395116	-0.591309	2.225291
C	4.641263	-1.116097	-1.225650
H	4.827831	-2.191689	-1.161850
H	3.994466	-0.935888	-2.088344
H	5.599248	-0.613165	-1.395614

β,β′-Me$_2$-TP

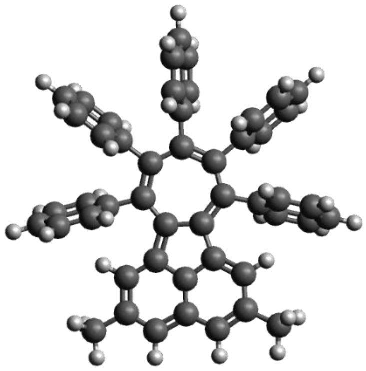

Table S134. Coordinates and energy for the optimized geometry of **β,β′-Me$_2$-TP**.

	G = –1887.770119		
	**Coordinates / Å**		
**Atom**	**x**	**y**	**z**
C	-1.142644	-1.275708	0.040951
C	-1.754677	0.000144	0.000274
C	0.234829	-1.601543	-0.015559
C	1.331962	-0.725833	-0.068977
C	1.332041	0.725643	0.069156
C	0.235058	1.601526	0.015304
C	-1.142468	1.275899	-0.040946
C	2.738127	1.155147	0.188691
C	3.400086	2.352262	0.451600
C	4.818987	2.391309	0.487046
C	5.566070	1.242283	0.251638
H	6.651400	1.294413	0.266774
C	2.738045	-1.155513	-0.188122
C	3.399969	-2.352696	-0.450820
C	3.526947	-0.000234	0.000434
C	4.818882	-2.391913	-0.485725
C	4.931199	-0.000318	0.000706
C	5.566018	-1.242991	-0.249990
H	6.651347	-1.295255	-0.264702
C	0.562158	3.063492	-0.013442
C	0.424480	3.850801	1.133978
C	1.042821	3.636587	-1.195921
C	0.781765	5.198463	1.101585
H	0.051912	3.406610	2.051226
C	1.387733	4.986524	-1.229321
H	1.154179	3.021561	-2.083526
C	1.264762	5.768386	-0.078626
H	0.682858	5.802228	1.998429
H	1.758436	5.424961	-2.150746
H	1.541106	6.818057	-0.101796
C	-2.078311	2.450245	-0.114422
C	-2.246820	3.137269	-1.320890

C	-2.783314	2.866504	1.019698
C	-3.118313	4.222502	-1.394636
H	-1.698724	2.818916	-2.201852
C	-3.647035	3.957644	0.946988
H	-2.655963	2.334904	1.957164
C	-3.819430	4.636551	-0.260794
H	-3.247005	4.744734	-2.337926
H	-4.187094	4.274904	1.833965
H	-4.496139	5.483803	-0.317781
C	-3.258343	0.000249	0.000830
C	-3.962285	-0.264856	1.179988
C	-3.963370	0.265458	-1.177656
C	-5.355833	-0.259766	1.181053
H	-3.418428	-0.473556	2.095563
C	-5.356915	0.260635	-1.177360
H	-3.420395	0.474069	-2.093769
C	-6.056956	0.000510	0.002187
H	-5.892881	-0.461577	2.102962
H	-5.894819	0.462539	-2.098749
H	-7.142781	0.000624	0.002710
C	-2.078668	-2.449942	0.113934
C	-2.783770	-2.865453	-1.020399
C	-2.247253	-3.137625	1.320013
C	-3.647627	-3.956530	-0.948297
H	-2.656396	-2.333314	-1.957557
C	-3.118876	-4.222791	1.393159
H	-1.699118	-2.819832	2.201152
C	-3.820064	-4.636116	0.259093
H	-4.187758	-4.273204	-1.835440
H	-3.247616	-4.745542	2.336154
H	-4.496869	-5.483322	0.315612
C	0.561614	-3.063596	0.012520
C	0.424146	-3.850190	-1.135409
C	1.041701	-3.637508	1.194836
C	0.781085	-5.197964	-1.103687
H	0.052005	-3.405361	-2.052523
C	1.386284	-4.987545	1.227554
H	1.152899	-3.023045	2.082850
C	1.263531	-5.768699	0.076353
H	0.682338	-5.801177	-2.000920
H	1.756559	-5.426616	2.148850
H	1.539611	-6.818452	0.099003
H	2.871175	-3.274007	-0.643103
C	5.491757	3.710529	0.777587
H	5.141027	4.488447	0.090038
H	5.261452	4.051374	1.794094
H	6.578126	3.634005	0.684091
H	2.871309	3.273639	0.643643

C	5.491602	-3.711184	-0.776155
H	5.139716	-4.489403	-0.089544
H	5.262526	-4.051335	-1.793176
H	6.577874	-3.635065	-0.681209

β,β′-Me₂-DT

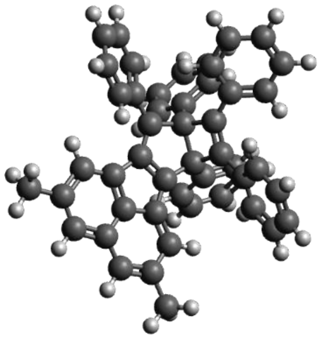

Table S135. Coordinates and energy for the optimized geometry of **β,β′-Me₂-DT**.

G = –1887.741719			
		**Coordinates / Å**	
**Atom**	**x**	**y**	**z**
C	4.515332	1.397934	-0.133202
C	5.386053	0.279048	-0.167762
C	3.155435	1.106649	0.015676
H	6.453346	0.444347	-0.289621
C	4.920445	-1.038878	-0.043850
C	2.649875	-0.222964	0.159971
C	3.543065	-1.294973	0.132219
C	1.243790	-0.078723	0.330365
C	2.133427	2.084848	0.071116
C	0.889783	1.305485	0.246703
C	0.061173	-0.947160	0.503096
C	2.507203	3.412395	0.006841
C	4.862954	2.776110	-0.220216
C	-0.484452	1.471569	0.308746
C	-1.159530	0.114241	0.492933
C	-0.500406	-1.410605	-0.863491
C	0.091314	-1.953653	1.627648
C	3.887408	3.759453	-0.143795
H	5.904617	3.059598	-0.338925
C	-1.194249	2.720351	0.089432
C	-1.559972	-0.569145	-0.852659
C	-2.106104	0.012114	1.664679
C	0.116958	-2.349532	-1.793263
C	0.981802	-1.810942	2.697774
C	-0.835241	-3.003997	1.649488
C	-0.696231	3.622746	-0.876720
C	-2.397225	3.037065	0.757691
C	-2.731941	-0.398432	-1.707172

C	-1.669388	0.458324	2.921519
C	-3.366282	-0.580312	1.553445
C	0.974430	-3.360666	-1.323471
C	-0.072750	-2.215778	-3.181901
H	1.698440	-0.996019	2.703630
C	0.952437	-2.706296	3.767550
C	-0.867290	-3.895228	2.719474
H	-1.532636	-3.121062	0.826730
H	0.193579	3.363739	-1.437965
C	-1.377322	4.798820	-1.164493
C	-3.058012	4.228703	0.481948
H	-2.795112	2.362499	1.502784
C	-3.405092	0.831151	-1.812068
C	-3.238245	-1.505822	-2.417625
H	-0.687744	0.912885	3.020575
C	-2.482877	0.322099	4.042590
C	-4.182824	-0.714840	2.678595
H	-3.715868	-0.938432	0.592861
H	1.122067	-3.484870	-0.256656
C	1.612502	-4.220769	-2.215198
C	0.567833	-3.075645	-4.068333
H	-0.711632	-1.425073	-3.560109
H	1.650711	-2.582306	4.589918
C	0.027630	-3.750418	3.782189
H	-1.591084	-4.704699	2.721711
H	-0.995251	5.469060	-1.927661
C	-2.555623	5.109795	-0.479682
H	-3.971822	4.468751	1.015778
H	-3.044081	1.692365	-1.267425
C	-4.531569	0.955647	-2.622527
C	-4.363675	-1.377326	-3.224388
H	-2.749190	-2.469214	-2.320447
H	-2.130856	0.671148	5.008711
C	-3.745788	-0.264939	3.923692
H	-5.161145	-1.175078	2.577029
H	2.265947	-5.000000	-1.834360
C	1.411735	-4.082446	-3.589466
H	0.415151	-2.955713	-5.136792
H	0.003553	-4.447184	4.614645
H	-3.083769	6.032342	-0.699449
H	-5.033486	1.915890	-2.695072
C	-5.013978	-0.144220	-3.333322
H	-4.740479	-2.242195	-3.762123
H	-4.381806	-0.371294	4.797306
H	1.910486	-4.751529	-4.284122
H	-5.893897	-0.045468	-3.961792
H	3.196231	-2.316639	0.243237
H	1.777068	4.212430	0.071966

C	4.253379	5.220915	-0.219680
H	3.764955	5.703670	-1.074346
H	3.926810	5.753517	0.681129
H	5.332973	5.356574	-0.323273
C	5.885325	-2.198040	-0.087879
H	5.647426	-2.870798	-0.919648
H	6.915543	-1.855251	-0.211195
H	5.829625	-2.788267	0.833677

α,α′-(OMe)$_2$-TP

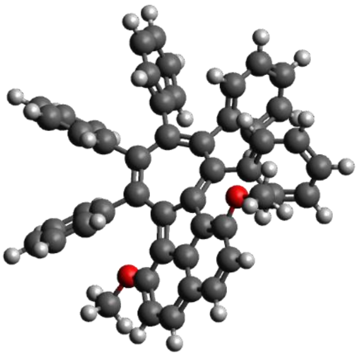

Table S136. Coordinates and energy for the optimized geometry of **α,α′-(OMe)$_2$-TP**.

G = –2038.171979			
		Coordinates / Å	
Atom	x	y	z
C	-1.265157	1.157548	0.168177
C	-0.000935	1.771704	-0.000111
C	-1.601928	-0.201587	-0.055602
C	-0.700175	-1.274049	-0.217072
C	0.701343	-1.273321	0.217037
C	1.602031	-0.199971	0.055526
C	1.263916	1.158802	-0.168364
C	1.052695	-2.656459	0.506450
C	2.097561	-3.317306	1.188128
C	2.121113	-4.739570	1.191377
C	1.116188	-5.490972	0.608465
H	2.936694	-5.251778	1.684722
H	1.171756	-6.575009	0.644480
C	-1.050083	-2.657568	-0.506497
C	-2.094209	-3.319538	-1.188225
C	0.001734	-3.458140	-0.000032
C	-2.116217	-4.741830	-1.191516
C	0.002502	-4.863192	-0.000059
C	-1.110494	-5.492166	-0.608613
H	-2.931246	-5.254895	-1.684890
H	-1.164876	-6.576265	-0.644684
C	3.054504	-0.549333	-0.025365

C	3.993223	-0.007725	0.862350
C	3.487581	-1.419255	-1.032398
C	5.340556	-0.333407	0.745598
H	3.663017	0.655392	1.653766
C	4.839262	-1.741483	-1.152796
H	2.766331	-1.834423	-1.729341
C	5.768675	-1.200822	-0.263742
H	6.058083	0.088493	1.443014
H	5.164404	-2.410003	-1.944356
H	6.821483	-1.450020	-0.357188
C	2.393914	2.060363	-0.582740
C	2.875228	1.962383	-1.892965
C	2.968437	2.991812	0.289232
C	3.909241	2.790564	-2.328639
H	2.437309	1.235883	-2.570807
C	4.006520	3.813125	-0.143500
H	2.600397	3.074995	1.305885
C	4.478383	3.717666	-1.454701
H	4.269749	2.708550	-3.349750
H	4.446227	4.529459	0.544090
H	5.285086	4.361589	-1.792072
C	-0.001667	3.276451	-0.000124
C	-0.094412	3.980550	1.204633
C	0.090464	3.980668	-1.204861
C	-0.092808	5.374284	1.204276
H	-0.174242	3.435678	2.139927
C	0.087706	5.374401	-1.204457
H	0.170723	3.435904	-2.140183
C	-0.002830	6.074954	-0.000077
H	-0.163161	5.912078	2.145076
H	0.157605	5.912283	-2.145239
H	-0.003274	7.160863	-0.000056
C	-2.396021	2.058018	0.582576
C	-2.971487	2.988949	-0.289331
C	-2.877194	1.959515	1.892813
C	-4.010359	3.809222	0.143482
H	-2.603609	3.072533	-1.306014
C	-3.912018	2.786644	2.328559
H	-2.438552	1.233400	2.570594
C	-4.482098	3.713232	1.454688
H	-4.450791	4.525156	-0.544061
H	-4.272421	2.704216	3.349674
H	-5.289429	4.356339	1.792111
C	-3.054048	-0.552327	0.025480
C	-3.993356	-0.011752	-0.862234
C	-3.486219	-1.422354	1.032814
C	-5.340388	-0.338576	-0.745206
H	-3.663823	0.651473	-1.653839

C	-4.837600	-1.745730	1.153483
H	-2.764512	-1.836676	1.729784
C	-5.767604	-1.206111	0.264415
H	-6.058386	0.082532	-1.442615
H	-5.162052	-2.414320	1.945268
H	-6.820183	-1.456199	0.358074
O	2.987817	-2.578741	1.853140
O	-2.985251	-2.581952	-1.853270
C	-4.137237	-3.198751	-2.440773
H	-4.715298	-3.732701	-1.681438
H	-4.727951	-2.374249	-2.835565
H	-3.846584	-3.876115	-3.249355
C	4.140555	-3.194239	2.440525
H	4.719125	-3.727567	1.681142
H	4.730394	-2.369067	2.835224
H	3.850743	-3.871907	3.249153

α,α′-(OMe)$_2$-DT

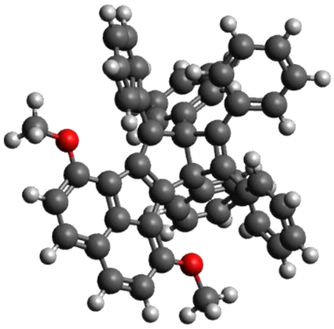

Table S137. Coordinates and energy for the optimized geometry of **α,α′-(OMe)$_2$-DT**.

	G = –2038.160385		
		**Coordinates / Å**	
**Atom**	**x**	**y**	**z**
C	3.170429	3.508576	-0.276882
C	4.490495	2.997544	-0.363601
C	2.159226	2.562680	-0.061609
H	5.315979	3.681434	-0.539878
C	4.771206	1.642337	-0.222564
C	2.407049	1.152787	0.093883
H	5.799909	1.313627	-0.292286
C	3.748095	0.694124	0.022141
C	1.160022	0.550827	0.314738
C	0.786889	2.861822	0.052727
C	0.131475	1.566685	0.266290
C	0.582844	-0.805159	0.486120
C	0.412955	4.210649	0.010407
C	2.753924	4.862639	-0.368546
C	-1.123954	1.000452	0.341353
C	-1.009617	-0.510422	0.536163

C	0.274898	-1.482751	-0.871460
C	1.146438	-1.669163	1.588036
C	1.419726	5.195706	-0.223750
H	3.485654	5.646211	-0.540090
C	-2.390826	1.678955	0.092300
C	-1.064342	-1.300427	-0.804863
C	-1.737188	-1.056345	1.739809
C	1.227710	-1.980000	-1.862834
C	1.889639	-1.110821	2.633337
C	0.859972	-3.039806	1.618185
H	1.132995	6.238178	-0.276497
C	-2.485691	2.526698	-1.028559
C	-3.538175	1.454037	0.879046
C	-2.191269	-1.733352	-1.628989
C	-1.547876	-0.432906	2.982592
C	-2.530236	-2.204289	1.670467
C	2.408193	-2.634678	-1.472777
C	0.999948	-1.754822	-3.233425
H	2.113087	-0.048893	2.630619
C	2.345631	-1.906465	3.684249
C	1.312108	-3.835346	2.669056
H	0.278593	-3.482954	0.816395
H	-1.613568	2.677570	-1.654028
C	-3.698194	3.118441	-1.364598
C	-4.739794	2.076291	0.555103
H	-3.479658	0.812567	1.748346
C	-3.448244	-1.108102	-1.555143
C	-2.043881	-2.839110	-2.491339
H	-0.924255	0.453949	3.048935
C	-2.150178	-0.941785	4.129864
C	-3.135436	-2.714215	2.821230
H	-2.677336	-2.704910	0.721203
H	2.597389	-2.810711	-0.421404
C	3.327323	-3.064088	-2.428345
C	1.922743	-2.180904	-4.184217
H	0.101380	-1.231556	-3.543862
H	2.923403	-1.456503	4.486334
C	2.058351	-3.271669	3.706206
H	1.080965	-4.896446	2.676506
H	-3.763476	3.748042	-2.246419
C	-4.826655	2.900362	-0.570370
H	-5.612917	1.911380	1.178521
H	-3.597357	-0.274992	-0.883101
C	-4.510584	-1.550532	-2.340874
C	-3.107015	-3.278593	-3.272723
H	-1.092969	-3.357870	-2.536300
H	-1.993973	-0.448240	5.084493
C	-2.949601	-2.085709	4.051842

H	-3.751190	-3.606073	2.751343
H	4.230593	-3.577083	-2.110928
C	3.089707	-2.839238	-3.785519
H	1.735357	-1.992708	-5.237294
H	2.412564	-3.891918	4.524200
H	-5.771409	3.368985	-0.828594
H	-5.470472	-1.047279	-2.271128
C	-4.345370	-2.632541	-3.206177
H	-2.972381	-4.133070	-3.929416
H	-3.420976	-2.483443	4.945616
H	3.808794	-3.171824	-4.528251
H	-5.174710	-2.977145	-3.816630
O	-0.878027	4.506064	0.210745
O	3.950712	-0.604056	0.204828
C	5.279885	-1.144359	0.128251
H	5.165173	-2.209785	0.318447
H	5.698176	-0.983824	-0.868711
H	5.919814	-0.693974	0.891663
C	-1.337012	5.856573	0.082147
H	-0.896394	6.493505	0.855208
H	-1.105865	6.253995	-0.910859
H	-2.416842	5.802172	0.215005

α,α′-Cl_2-TP

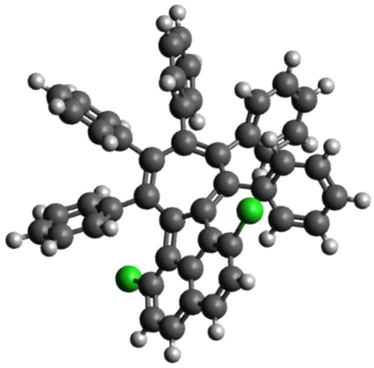

Table S138. Coordinates and energy for the optimized geometry of **α,α′-Cl_2-TP**.

G = –2728.366213			
	Coordinates / Å		
Atom	x	y	z
C	1.125556	-1.263541	-0.176646
C	1.744364	-0.002207	0.000132
C	-0.226916	-1.595295	0.111169
C	-1.282600	-0.674636	0.266519
C	-1.281032	0.677894	-0.266182
C	-0.222954	1.595850	-0.111464
C	1.128686	1.260672	0.176556
C	-2.672478	0.990713	-0.627492
C	-3.339204	1.903208	-1.445519

C	-4.755074	1.911025	-1.495108
C	-5.515454	1.012009	-0.771078
H	-5.239010	2.643376	-2.130536
H	-6.598543	1.056980	-0.822635
C	-2.674590	-0.984019	0.628457
C	-3.343094	-1.894959	1.446766
C	-3.477726	0.004361	0.000724
C	-4.758945	-1.899315	1.497145
C	-4.881896	0.006066	0.001102
C	-5.517490	-0.998357	0.773590
H	-5.244351	-2.630519	2.132773
H	-6.600660	-1.040665	0.825713
C	-0.578801	3.046133	-0.086984
C	0.070105	3.977466	-0.910401
C	-1.550157	3.493676	0.818163
C	-0.248847	5.328291	-0.830591
H	0.808586	3.637967	-1.626972
C	-1.862465	4.849610	0.903490
H	-2.046038	2.782718	1.471139
C	-1.213507	5.769326	0.079431
H	0.253548	6.037798	-1.480707
H	-2.608009	5.185093	1.617792
H	-1.456612	6.825499	0.145135
C	2.001747	2.382529	0.658421
C	1.788069	2.857285	1.958130
C	3.009027	2.957001	-0.125509
C	2.580092	3.883651	2.471542
H	1.002019	2.419965	2.566340
C	3.789980	3.990681	0.384114
H	3.180743	2.596504	-1.133322
C	3.581475	4.454428	1.685171
H	2.410403	4.237858	3.483819
H	4.563886	4.433035	-0.235775
H	4.195462	5.257033	2.082412
C	3.247989	-0.004115	0.000443
C	3.950032	-0.080057	-1.206487
C	3.949707	0.070072	1.207672
C	5.343755	-0.078977	-1.205093
H	3.405107	-0.146900	-2.142696
C	5.343420	0.065542	1.206868
H	3.404554	0.138307	2.143647
C	6.043499	-0.007585	0.001034
H	5.881858	-0.135614	-2.146451
H	5.881264	0.120851	2.148453
H	7.129298	-0.008931	0.001266
C	1.995809	-2.387503	-0.658650
C	3.001585	-2.964660	0.125251
C	1.780913	-2.861614	-1.958401

C	3.779803	-4.000373	-0.384429
H	3.174243	-2.604713	1.133095
C	2.570234	-3.890024	-2.471868
H	0.996001	-2.422206	-2.566575
C	3.570083	-4.463500	-1.685510
H	4.552524	-4.444815	0.235444
H	2.399636	-4.243706	-3.484176
H	4.181940	-5.267719	-2.082774
C	-0.586751	-3.044585	0.085871
C	0.059695	-3.978318	0.908506
C	-1.559527	-3.488765	-0.819419
C	-0.262995	-5.328196	0.827726
H	0.799184	-3.641372	1.625242
C	-1.875596	-4.843766	-0.905712
H	-2.053551	-2.775933	-1.471763
C	-1.229014	-5.765885	-0.082467
H	0.237530	-6.039574	1.477239
H	-2.622195	-5.176658	-1.620124
H	-1.475040	-6.821334	-0.148932
Cl	-2.524825	-2.977943	2.529712
Cl	-2.518990	2.984078	-2.529121

α,α′-Cl$_2$-DT

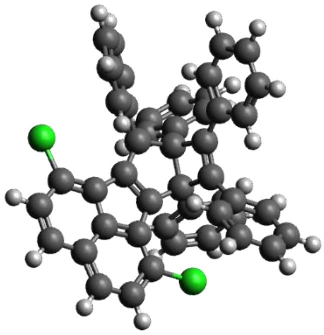

Table S139. Coordinates and energy for the optimized geometry of **α,α′-Cl$_2$-DT**.

G = –2728.354797			
		**Coordinates / Å**	
**Atom**	**x**	**y**	**z**
C	-2.963477	3.658924	0.047697
C	-4.302241	3.230141	0.234895
C	-2.008360	2.649064	-0.125001
H	-5.086709	3.965459	0.385591
C	-4.638964	1.880023	0.226156
C	-2.327849	1.248900	-0.165282
H	-5.670152	1.581574	0.372255
C	-3.669157	0.880149	0.021026
C	-1.102140	0.572472	-0.410055
C	-0.619375	2.873975	-0.324130
C	-0.033754	1.528984	-0.450420

C	-0.613495	-0.818614	-0.515842
C	-0.219152	4.197008	-0.435425
C	-2.492203	4.999620	-0.002132
C	1.193888	0.884342	-0.439486
C	0.987956	-0.620845	-0.532104
C	-0.383440	-1.425909	0.899787
C	-1.225158	-1.687962	-1.582199
C	-1.158187	5.249634	-0.251311
H	-3.181436	5.826663	0.132823
C	2.485916	1.470964	-0.125977
C	0.954053	-1.268890	0.888136
C	1.714349	-1.321797	-1.650961
C	-1.419330	-1.792415	1.876935
C	-1.902808	-1.120982	-2.667518
C	-1.041876	-3.074747	-1.545500
H	-0.800872	6.270104	-0.323758
C	2.574371	2.322926	0.993252
C	3.658640	1.133837	-0.831145
C	2.014741	-1.514086	1.859314
C	1.663083	-0.772053	-2.940476
C	2.348723	-2.551650	-1.459078
C	-2.073001	-3.032146	1.844658
C	-1.818468	-0.830682	2.820284
H	-2.037693	-0.044853	-2.721283
C	-2.406680	-1.927415	-3.687352
C	-1.540861	-3.881277	-2.566193
H	-0.489586	-3.521019	-0.726074
H	1.677007	2.564613	1.552601
C	3.810188	2.798379	1.415670
C	4.883674	1.652400	-0.428756
H	3.599421	0.492889	-1.701539
C	3.366662	-1.308204	1.535117
C	1.692051	-1.956051	3.158278
H	1.156319	0.174925	-3.103149
C	2.250936	-1.435018	-4.014601
C	2.937327	-3.216286	-2.536381
H	2.382177	-2.995439	-0.470677
H	-1.776631	-3.779140	1.117169
C	-3.108633	-3.302151	2.738114
C	-2.856162	-1.103483	3.709669
H	-1.311657	0.129522	2.846664
H	-2.934272	-1.472532	-4.520325
C	-2.229175	-3.310621	-3.638756
H	-1.389708	-4.955694	-2.523557
H	3.871600	3.428149	2.297211
C	4.965352	2.471046	0.702110
H	5.779384	1.409610	-0.991308
H	3.638774	-0.983822	0.539987

C	4.364985	-1.517447	2.483206
C	2.692829	-2.167288	4.100733
H	0.655130	-2.125089	3.425376
H	2.205076	-0.997653	-5.007329
C	2.892116	-2.660494	-3.814569
H	3.428488	-4.170735	-2.372814
H	-3.607666	-4.266043	2.705925
C	-3.505738	-2.338768	3.667586
H	-3.157485	-0.351692	4.433045
H	-2.620585	-3.939464	-4.432701
H	5.928337	2.854396	1.024835
H	5.402543	-1.344519	2.213829
C	4.033133	-1.945308	3.769496
H	2.427285	-2.504361	5.098299
H	3.350254	-3.178547	-4.651576
H	-4.316343	-2.550914	4.358283
H	4.811418	-2.107299	4.509181
Cl	-4.164483	-0.773583	0.007967
Cl	1.406590	4.643566	-0.865368

α,α′-^{i}Pr$_2$-TP

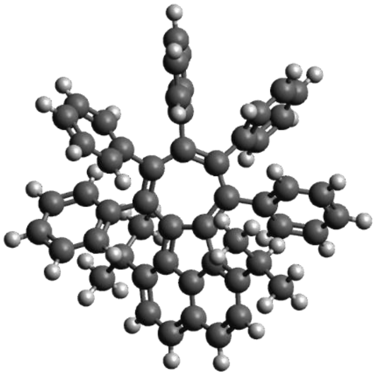

Table S140. Coordinates and energy for the optimized geometry of **α,α′-^{i}Pr$_2$-TP**.

G = –2044.914917			
	**Coordinates / Å**		
**Atom**	**x**	**y**	**z**
C	1.057196	1.387981	0.316475
C	1.844860	0.257693	-0.000992
C	-0.324228	1.556620	0.023804
C	-1.252981	0.526073	-0.228109
C	-1.062012	-0.847975	0.226909
C	0.113473	-1.585100	-0.023076
C	1.396176	-1.045210	-0.315835
C	-2.390869	-1.367290	0.588910
C	-2.889953	-2.415022	1.393557
C	-4.302494	-2.572253	1.421779
C	-5.186145	-1.761329	0.732243
H	-4.716760	-3.372601	2.019808

H	-6.253682	-1.954297	0.784293
C	-2.672413	0.662634	-0.592247
C	-3.437085	1.534569	-1.397926
C	-3.313490	-0.461078	-0.002242
C	-4.838980	1.301028	-1.427858
C	-4.704289	-0.654277	-0.003187
C	-5.468885	0.279767	-0.739194
H	-5.454932	1.958296	-2.026590
H	-6.548569	0.174515	-0.792253
C	-0.017212	-3.068782	-0.122814
C	0.798260	-3.920572	0.638088
C	-0.954873	-3.625126	-1.002320
C	0.665587	-5.301940	0.531936
H	1.515021	-3.497835	1.332364
C	-1.081340	-5.008736	-1.113187
H	-1.579911	-2.973308	-1.603567
C	-0.275220	-5.849796	-0.344043
H	1.291526	-5.950064	1.137287
H	-1.808188	-5.428515	-1.801666
H	-0.377670	-6.927428	-0.427419
C	2.381631	-1.979885	-0.954896
C	2.152218	-2.341302	-2.288304
C	3.503169	-2.491412	-0.291356
C	3.037620	-3.189885	-2.952297
H	1.279106	-1.954088	-2.805091
C	4.379359	-3.348791	-0.951189
H	3.689632	-2.220065	0.741322
C	4.152641	-3.697523	-2.284762
H	2.852167	-3.455304	-3.988760
H	5.241722	-3.743726	-0.422647
H	4.840434	-4.362110	-2.798869
C	3.333541	0.465286	-0.004180
C	4.041972	0.491938	1.201105
C	4.016579	0.632214	-1.212935
C	5.422822	0.681791	1.196749
H	3.509602	0.372163	2.139397
C	5.396852	0.826542	-1.215171
H	3.467310	0.602015	-2.148678
C	6.103072	0.850580	-0.010887
H	5.965978	0.699390	2.136799
H	5.919755	0.957346	-2.157769
H	7.178552	1.000138	-0.013479
C	1.748495	2.554467	0.960341
C	2.691571	3.354072	0.303615
C	1.423342	2.835922	2.293252
C	3.297621	4.415993	0.969403
H	2.951131	3.146487	-0.728033
C	2.040832	3.891712	2.963194

H	0.686734	2.223774	2.805112
C	2.978202	4.686067	2.302215
H	4.022318	5.032396	0.446088
H	1.785371	4.093385	3.999122
H	3.456374	5.511526	2.820889
C	-0.856432	2.947798	0.124882
C	-0.303678	3.991694	-0.632998
C	-1.913245	3.224789	1.001687
C	-0.809989	5.283727	-0.526320
H	0.502599	3.782680	-1.326174
C	-2.414293	4.520630	1.113022
H	-2.337910	2.425940	1.600405
C	-1.867318	5.551581	0.347077
H	-0.383940	6.079369	-1.129452
H	-3.230368	4.724049	1.799446
H	-2.261422	6.559743	0.430849
C	-2.024339	-3.286728	2.286783
H	-1.341518	-3.838654	1.641733
C	-2.839893	2.608253	-2.290887
H	-2.328744	3.322233	-1.646047
C	-3.860232	3.413304	-3.104503
H	-4.589157	3.918891	-2.463015
H	-3.329733	4.184836	-3.671270
H	-4.402810	2.788481	-3.823143
C	-2.789270	-4.334590	3.104253
H	-3.355279	-5.020156	2.465435
H	-2.069855	-4.932607	3.672341
H	-3.479714	-3.876892	3.821960
C	-1.164798	-2.415861	3.226235
H	-1.801354	-1.835282	3.903646
H	-0.513905	-3.054761	3.831907
H	-0.532132	-1.715921	2.674566
C	-1.779862	2.001498	-3.233363
H	-2.237989	1.270874	-3.909737
H	-1.326960	2.792293	-3.840181
H	-0.980465	1.498200	-2.683548

α,α′-^{i}Pr$_2$-DT

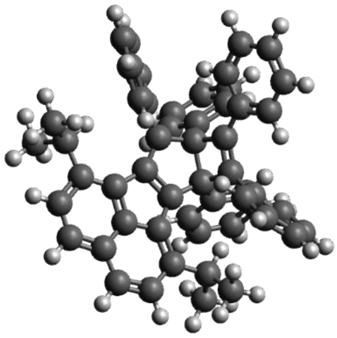

Table S141. Coordinates and energy for the optimized geometry of **α,α′-^{i}Pr$_2$**.

	G = –2044.911718		
		Coordinates / Å	
**Atom**	**x**	**y**	**z**
C	1.375716	4.386346	-0.585891
C	2.784184	4.476906	-0.698218
C	0.862313	3.115961	-0.296101
H	3.241695	5.434977	-0.928981
C	3.597081	3.362115	-0.511584
C	1.693936	1.958011	-0.083425
H	4.669703	3.489740	-0.606814
C	3.097891	2.079007	-0.198767
C	0.801085	0.905782	0.246245
C	-0.517729	2.837725	-0.127854
C	-0.556929	1.391844	0.172358
C	0.847336	-0.546401	0.561182
C	-1.426311	3.894483	-0.172150
C	0.431753	5.441848	-0.706840
C	-1.447595	0.343492	0.267501
C	-0.717797	-0.949750	0.589140
C	0.903881	-1.427201	-0.719371
C	1.665220	-0.959111	1.759874
C	-0.906923	5.186747	-0.493907
H	0.769056	6.447214	-0.939969
C	-2.861175	0.359546	-0.103543
C	-0.397334	-1.784267	-0.684083
C	-1.192699	-1.656171	1.834676
C	1.998925	-1.622750	-1.681948
C	1.996476	-0.031276	2.754398
C	2.002101	-2.304532	1.951643
H	-1.606627	6.013382	-0.554642
C	-3.195407	0.825397	-1.388988
C	-3.871240	-0.152646	0.730789
C	-1.250690	-2.628743	-1.515279
C	-1.382300	-0.908487	3.006514
C	-1.360740	-3.042511	1.879064
C	3.157670	-2.348900	-1.365791
C	1.867478	-1.079557	-2.971886
H	1.723901	1.012550	2.634613
C	2.673477	-0.435178	3.905142
C	2.678350	-2.709349	3.100825
H	1.715041	-3.040159	1.208250
H	-2.412847	1.206684	-2.036627
C	-4.510080	0.758498	-1.838340
C	-5.189961	-0.180544	0.285551
H	-3.624843	-0.503863	1.724892
C	-2.626653	-2.752159	-1.252665

C	-0.707110	-3.353993	-2.595400
H	-1.236530	0.167616	2.987343
C	-1.747272	-1.535099	4.195273
C	-1.726514	-3.670893	3.070890
H	-1.204207	-3.635968	0.986119
H	3.279657	-2.765408	-0.372660
C	4.146298	-2.551053	-2.328346
C	2.860617	-1.278729	-3.929221
H	0.974616	-0.512603	-3.217577
H	2.925460	0.299161	4.664400
C	3.020942	-1.774953	4.080162
H	2.933238	-3.756670	3.231871
H	-4.753558	1.099460	-2.839483
C	-5.510828	0.261247	-1.000774
H	-5.968232	-0.556360	0.942123
H	-3.064434	-2.227640	-0.414674
C	-3.438166	-3.547963	-2.057334
C	-1.520810	-4.149921	-3.394675
H	0.353731	-3.292376	-2.805254
H	-1.890624	-0.942947	5.094082
C	-1.922667	-2.921002	4.230157
H	-1.855043	-4.748974	3.089793
H	5.032349	-3.124640	-2.073331
C	3.999904	-2.021757	-3.611426
H	2.742964	-0.856488	-4.922759
H	3.549258	-2.090078	4.974839
H	-6.538437	0.220059	-1.348073
H	-4.499280	-3.620722	-1.838519
C	-2.891141	-4.246556	-3.134303
H	-1.083956	-4.699357	-4.223323
H	-2.207254	-3.411174	5.156299
H	4.771565	-2.181974	-4.358318
H	-3.523743	-4.866497	-3.762537
C	-2.894010	3.766727	0.177711
H	-3.103076	2.716983	0.381708
C	4.054920	0.927200	-0.007022
H	3.471141	0.023502	0.176582
C	4.961479	1.158825	1.216422
H	5.604589	0.284866	1.363065
H	5.604684	2.033961	1.074842
H	4.373787	1.304025	2.126487
C	-3.205588	4.551144	1.466913
H	-3.045132	5.626304	1.331933
H	-4.253314	4.398949	1.747622
H	-2.574684	4.214009	2.295919
C	4.890160	0.696122	-1.278943
H	5.525036	-0.183881	-1.143778
H	4.248910	0.520673	-2.146199

H	5.537950	1.553194	-1.491297
C	-3.805485	4.214834	-0.977147
H	-3.577274	3.673107	-1.899496
H	-4.851630	4.020217	-0.719526
H	-3.701841	5.287021	-1.176205

6.12. Additional Data

A two-dimensional format of the IRC in Fig. 1f of the manuscript is reproduced below, to follow the reaction coordinate as a function of the C_1–C_4 internuclear distance (Fig. S79a) or H_5–C_5–C_4–H_4 dihedral angle (Fig. S79b).

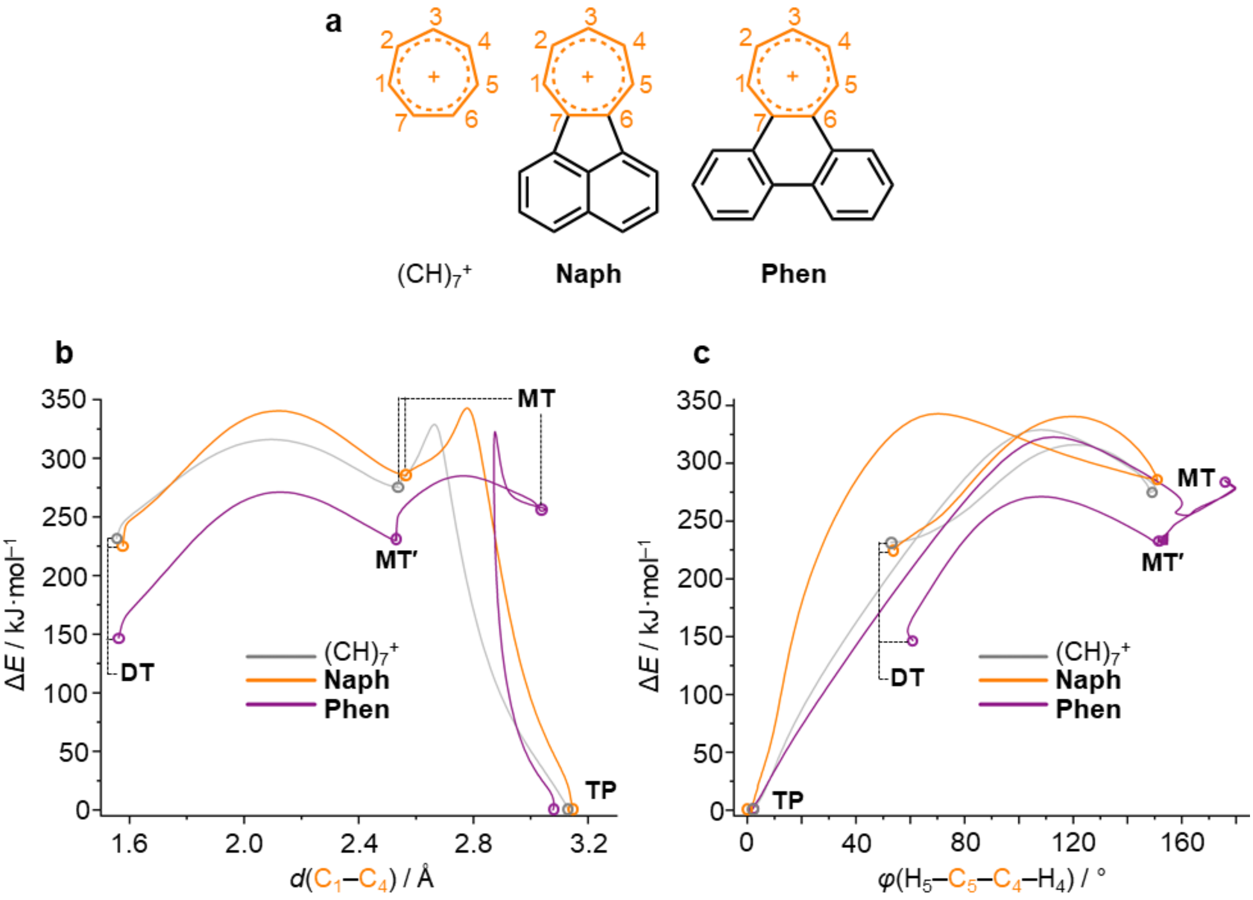

Fig. S79. Two-dimensional representations of data from Fig. 1f in the manuscript. (a) Structural formulae of $(CH)_7^+$, **Naph**, and **Phen**. (b) IRC of the rearrangements of $(CH)_7^+$, **Naph**, and **Phen**, plotted against the C_1–C_4 internuclear distance *d*. (c) IRC of the rearrangements of $(CH)_7^+$, **Naph**, and **Phen**, plotted against the H_5–C_5–C_4–H_4 dihedral angles, φ.

7. References

1. Dolomanov, O. V., Bourhis, L. J., Gildea, R. J., Howard, J. A. K. & Puschmann, H. *OLEX2* : a complete structure solution, refinement and analysis program. *J. Appl. Crystallogr.* **42**, 339–341 (2009).

2. Sheldrick, G. M. A short history of SHELX. *Acta Cryst. A* **64**, 112–122 (2008).

3. Friedrich, L. E. & Cormier, R. A. Attempted epoxidation of triphenylcyclopropene. *J. Org. Chem.* **35**, 450–454 (1970).

4. Breslow, R. & Chang, H. W. Heptaphenylcycloheptatrienyl anion. *J. Am. Chem. Soc.* **87**, 2200–2203 (1965).

5. Wooi, G. Y. & White, J. M. Structural manifestations of the cheletropic reaction. *Org. Biomol. Chem.* **3**, 972–974 (2005).

6. Turley, A. T. *et al.* Extended conjugation attenuates the quenching of aggregation-induced emitters by photocyclization pathways. *Angew. Chem. Int. Ed.* **61**, e202202193 (2022).

7. Hancock, E. N., Kuker, E. L., Tantillo, D. J. & Brown, M. K. Lessons in strain and stability: enantioselective synthesis of (+)-[5]-ladderanoic acid. *Angew. Chem. Int. Ed.* **59**, 436–441 (2020).

8. von Ragué Schleyer, P., Maerker, C., Dransfeld, A., Jiao, H. & Van Eikema Hommes, N. J. R. Nucleus-independent chemical shifts: a simple and efficient aromaticity probe. *J. Am. Chem. Soc.* **118**, 6317–6318 (1996).

9. Geuenich, D., Hess, K., Köhler, F. & Herges, R. Anisotropy of the induced current density (ACID), a general method to quantify and visualize electronic delocalization. *Chem. Rev.* **105** 3758–3772 (2005).

10. Wannere, C. S. *et al.* On the stability of large [4n]annulenes. *Org. Lett.* **5**, 2983–2986 (2003).

11. Lu, T. & Chen, F. Multiwfn: A multifunctional wavefunction analyzer. *J. Comput. Chem.* **33**, 580–592 (2012).

12. von Ragué Schleyer, P. *et al.* Dissected nucleus-independent chemical shift analysis of π-aromaticity and antiaromaticity. *Org. Lett.* **3**, 2465–2468 (2001).

13. Fallah-Bagher-Shaidaei, H., Wannere, C. S., Corminboeuf, C., Puchta, R. & Schleyer, P. V. R. Which NICS aromaticity index for planar π rings is best? *Org. Lett.* **8**, 863–866 (2006).
